# Kinetic trapping of 2,4,6-tris(4-pyridyl)benzene and ZnI_2_ into M_12_L_8_ poly-[*n*]-catenanes using solution and solid-state processes

**DOI:** 10.1038/s41598-023-32661-x

**Published:** 2023-04-05

**Authors:** Javier Martí-Rujas, Stefano Elli, Antonino Famulari

**Affiliations:** 1 Dipartimento di Chimica Materiali e Ingegneria Chimica, ‘‘Giulio Natta’’, Politecnico di Milano, Via L. Mancinelli 7, 20131 Milan, Italy; 2 Center for Nano Science and Technology@Polimi, Istituto Italiano di Tecnologia, Via Pascoli 70/3, 20133 Milan, Italy; 3 INSTM, Consorzio Interuniversitario Nazionale per la Scienza e Tecnologia dei Materiali, Florence, Italy

**Keywords:** Chemistry, Materials science

## Abstract

Here, we show that in a supramolecular system with more than 20 building blocks forming large icosahedral **M**_**12**_**L**_**8**_ metal–organic cages (**MOCs**), using the *instant synthesis method,* it is possible to *kinetically trap* and control the formation of interlocking **M**_**12**_**L**_**8**_ nanocages, giving rare **M**_**12**_**L**_**8**_ TPB-ZnI_2_ poly-[*n*]-catenane. The catenanes are obtained in a one-pot reaction, selectively as amorphous (**a1**) or crystalline states, as demonstrated by powder X-ray diffraction (powder XRD), thermogravimetric (TG) analysis and ^1^H NMR. The 300 K **M**_**12**_**L**_**8**_ poly-[*n*]-catenane single crystal X-ray diffraction (SC-XRD) structure including nitrobenzene (**1**) indicates strong guest binding with the large **M**_**12**_**L**_**8**_ cage (i.e., internal volume *ca*. 2600 Å^3^), allowing its structural resolution. Conversely, slow self-assembly (5 days) leads to a mixture of the **M**_**12**_**L**_**8**_ poly-[*n*]-catenane and a new TPB-ZnI_2_ (**2**) coordination polymer (i.e., thermodynamic product), as revealed by SC-XRD. The neat grinding solid-state synthesis also yields amorphous **M**_**12**_**L**_**8**_ poly-[*n*]-catenane (**a1′**), but not coordination polymers, selectively in 15 min. The dynamic behavior of the **M**_**12**_**L**_**8**_ poly-[*n*]-catenanes demonstrated by the *amorphous-to-crystalline* transformation upon the uptake of *ortho*-, *meta*- and *para*-xylenes shows the potential of **M**_**12**_**L**_**8**_ poly-[*n*]-catenanes as functional materials in molecular separation. Finally, combining SC-XRD of **1** and DFT calculations specific for the solid-state, the role of the guests in the stability of the 1D chains of **M**_**12**_**L**_**8**_ nanocages is reported. Energy interactions such as interaction energies (**E**), lattice energies (E*), host–guest energies (**E**_**host-guest**_) and guest-guest energies (**E**_**guest-guest**_) were analysed considering the X-ray structure with and without the nitrobenzene guest. Not only the synthetic control achieved in the synthesis of the **M**_**12**_**L**_**8**_** MOCs** but also their dynamic behavior either in the crystalline or amorphous phase are sufficient to raise scientific interest in areas ranging from fundamental to applied sides of chemistry and material sciences.

## Introduction

Poly-[*n*]-catenanes^1–4^ self-assembled interlocked metal organic cages (**MOCs**)^5^ are classified as mechanically interlocked materials (**MIMs**) and are receiving considerable interest not only due to their potential applications but also due to their aesthetic synthetic aspects. Despite advances in the synthesis of catenanes^6^, the formation of catenanes made of interlocked MOCs is still very challenging^7^. Interestingly, the combination of properties of MOFs and MOCs^8–11^ can be achieved with poly-[*n*]-catenanes formed by the interlocking of large icosahedral **M**_**12**_**L**_**8**_ nanocages via the *mechanical bond*^12^. The long extension along one direction of the mechanical bond confers poly-[*n*]-catenanes the strength, stability and dynamic behavior of a metal–organic material similar to those observed in MOFs. As quoted by Stoddart, the strength of a mechanical bond is comparable to the weakest “*participating*” chemical bond in the sense that its cleavage will allow the system components parts to dissociate^13^.

To date, most of the reported polycatenanes self-assembled from interlocked nanocages have used harsh conditions at high temperatures using solvothermal reactions under thermodynamic control^14–16^. Recently, it has been reported that the self-assembly of *tris*-pyridyl-benzene (**TPB**)**,** a very unexplored exo-tridentate ligand in the area of MOC and MOF chemistries^17^, and ZnCl_2_ or ZnBr_2_ with various aromatic solvents yields large single crystals of isostructural **M**_**12**_**L**_**8**_ poly-[*n*]-catenanes where icosahedral **M**_**12**_**L**_**8**_ nanocages with internal voids of *ca*. 2600 Å^3^ are interlocked by means of mechanical bonds. There are only four reported crystal structures of **M**_**12**_**L**_**8**_ poly-[*n*]-catenanes with **TPB** ligands, including different guests^18–21^ and six structures, without X-ray-determined guests, considering the analogous 2,4,6-tris-(4-pyridyl)pyridine (**TPP**) ligand^12,22^. However, to the best of our knowledge, there is no report of **TPB**-ZnI_2_ poly-[*n*]-catenane synthesized using nonconventional approaches such as *instant synthesis* or in the *solid-state* upon mechanochemical means^23^ under ambient conditions. It is known that small changes in one of the building blocks (metal ions, organic ligands and solvents) constituting a **MOC** can result in different products, particularly when there are a large number of molecules and/or ions as starting components. Thus, replacing the halide in the metal node of a **MOC** can be sufficient to give different structures or different behavior and properties in a family of isostructural materials^24^.

**Figure 1:**
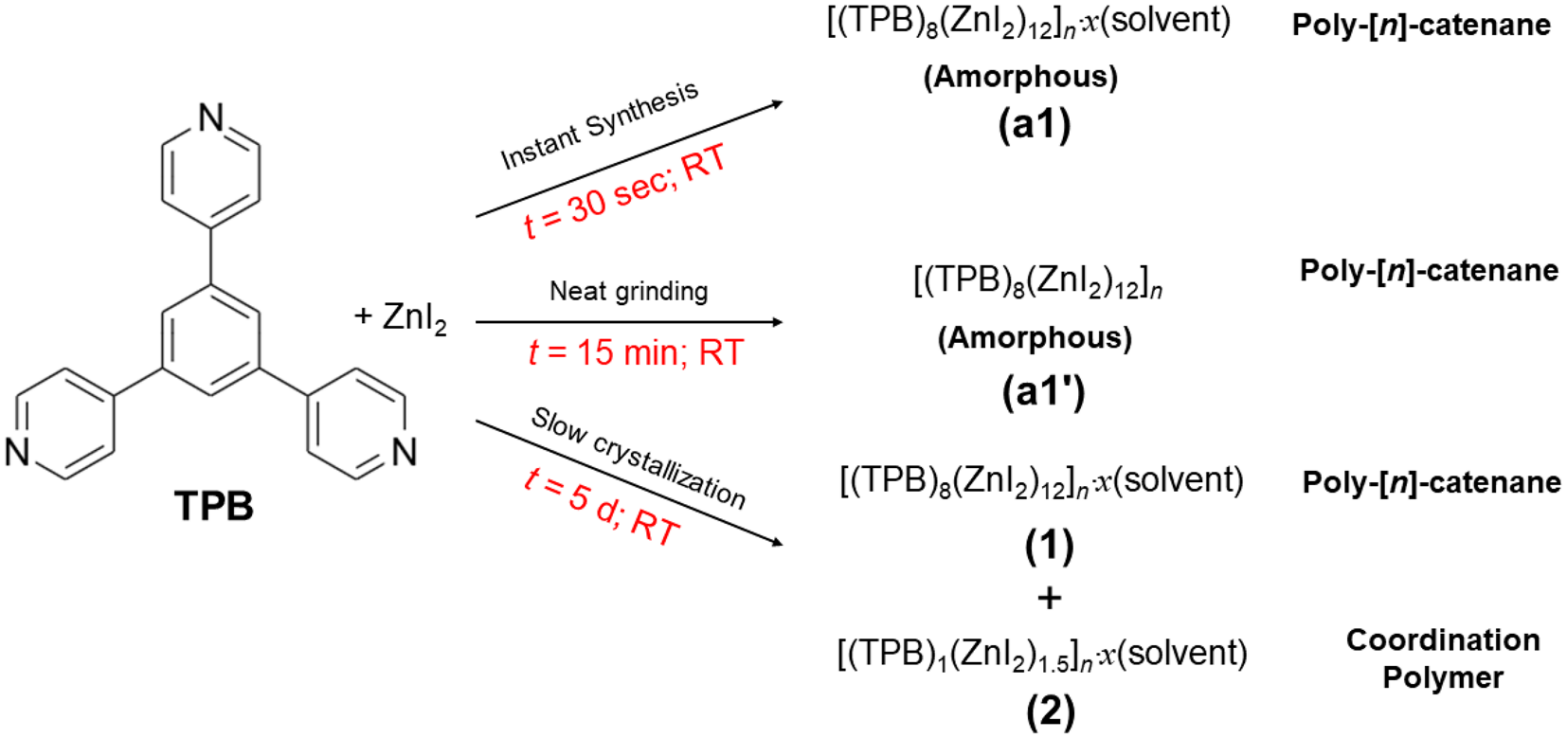
Self-assembly of **TPB** and ZnI_2_ using *instant synthesis*, *neat grinding* and *slow crystallization* approaches at room temperature conditions yielded **M**_**12**_**L**_**8**_ poly-[*n*]-catenane (**a1**, **a1′** and **1**) and coordination polymer (**2**).

Herein, we show that in a supramolecular system with more than 20 building blocks forming large icosahedral **M**_**12**_**L**_**8**_ nanocages, by using the *instant synthesis method*^25^, it is possible to *kinetically trap* and hence control the formation of interlocking **M**_**12**_**L**_**8**_ nanocages in rare **M**_**12**_**L**_**8**_ TPB-ZnI_2_ poly-[*n*]-catenane both as crystalline (**1**) and amorphous (**a1**) phases. Conversely, slow self-assembly (i.e., *ca.* 5 days) leads to a statistical mixture including a new coordination polymer TPB-ZnI_2_ (**2**) (i.e., thermodynamic product) and poly-[*n*]-catenane **1** (Fig. 1). In previous reports, the terminal ZnX_2_ ligand X was Cl or Br, whereas here it is I, which has a different size and polarizability compared to Cl and Br. Since ZnI_2_ occupies the vertices of the **M**_**12**_**L**_**8**_ icosahedron, it has a direct effect on the van der Waals interactions among adjacent cages and therefore can affect the poly-[*n*]-catenane’s dynamic behaviour. Thus, its single crystal X-ray structure (SC-XRD) is crucial for the structure–function correlation in **M**_**12**_**L**_**8**_** TPB** isostructural poly-[*n*]-catenanes. Additionally, solid-state synthesis by neat grinding yields amorphous **M**_**12**_**L**_**8**_ poly-[*n*]-catenane (**a1′**) but not coordination polymers in a selective manner in a short time (15 min). The dynamic behavior of the **M**_**12**_**L**_**8**_ poly-[*n*]-catenane chains demonstrated by the *amorphous-to-crystalline* (**a1′ → 1**) transformation upon the uptake of *ortho*-, *meta*- and *para*-xylenes and other aromatic molecules shows the potential of **M**_**12**_**L**_**8**_ poly-[*n*]-catenanes to be used in functional applications (i.e., molecular separation). Finally, combining structural single crystal X-ray diffraction (SC-XRD) data and DFT calculations specific for the solid-state, the role of the guest molecules in the stability of the 1D chains of **M**_**12**_**L**_**8**_ nanocages has been studied. Various energy interactions, such as interaction energies (**E**), lattice energies (E*), host–guest energies (**E**_**host-guest**_) and guest-guest energies (**E**_**guest-guest**_), were analysed considering **1** without solvent and including ordered nitrobenzene. DFT analysis considering guest molecules has been performed for the first time in the structural analysis of **M**_**12**_**L**_**8**_ poly-[*n*]-catenanes, providing important structural information regarding the dynamic behavior and guest inclusion/exchange properties in systems that do not have continuous channels.

## Results and discussion

### Synthesis of M_12_L_8_ poly-[***n***]-catenane using non-conventional crystallization method: instant synthesis of TPB and ZnI_2_ using aromatic solvents

Using the extremely fast crystallization method, *instant synthesis*^26,27^ at room temperature conditions in the preparation of mechanically interlocked large **M**_**12**_**L**_**8**_ cages is uncommon. The instant synthesis method allows selective synthesis using *kinetic control*, with which it is possible to obtain the poly-[*n*]-catenane of interlocked **M**_**12**_**L**_**8**_ nanocages as a homogeneous phase (i.e., *selectively*). Although we have demonstrated instant synthesis using **TPB** and ZnCl_2_ or ZnBr_2_, using ZnI_2_ has not been reported. Changing one of the building blocks in the self-assembling components (i.e., metal ions and organic ligands) can have a drastic effect on the final products. For instance, it can lead to different and unexpected structures, particularly in self-assembling systems where the building blocks are more than twenty components, as it is in the poly-[*n*]-catenanes self-assembled of **M**_**12**_**L**_**8**_ icosahedral nanocages including solvent.

**Figure 2:**
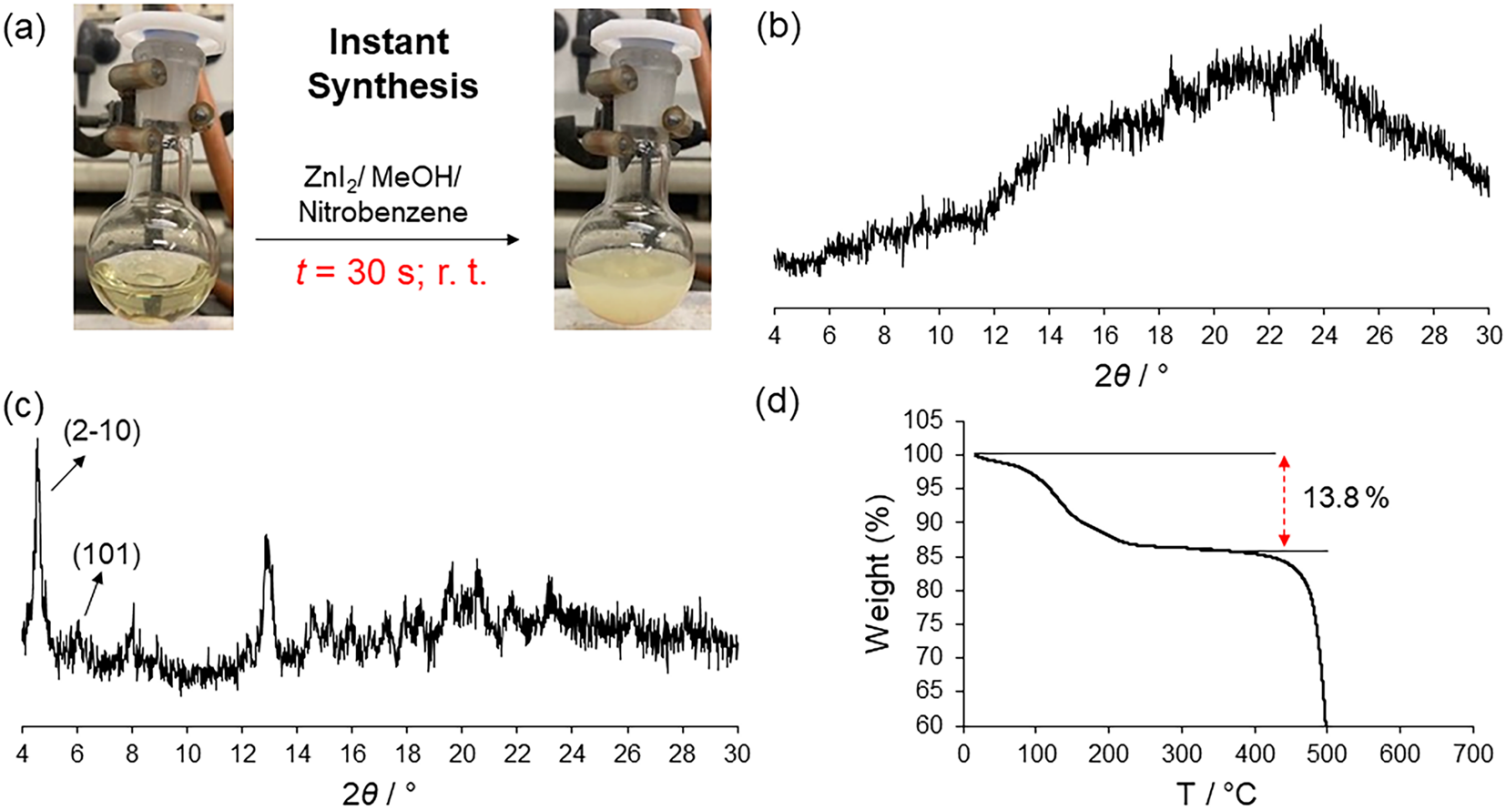
(**a**) Pictures taken during the instant synthesis experiments in the preparation of the **TPB**-ZnI_2_ poly-[*n*]-catenane at room temperature conditions before adding ZnI_2_ (left) and after complexation (right). (**b**) Experimental powder XRD pattern of the solid product (**a1**) obtained from the instant synthesis of **TPB** and ZnI_2_ using nitrobenzene as the templating aromatic solvent. The diffraction pattern shows that the material is amorphous. (**c**) The peak intensities observed in the powder XRD denote the formation of a crystalline phase. The first two peaks with Miller indices of (2–10) and (101) are highlighted. (**d**) TGA data carried out on **a1** immersed in MeOH/toluene showing the weight loss corresponding to *ca*. 14% of the total weight.

The addition of a methanolic solution of ZnI_2_ into the vigorous stirring light-yellow solution of **TPB** in nitrobenzene instantaneously formed a suspension that was stirred for 5 min (Fig. 2a). After filtration, the solid was very difficult to dry using pump filtration, and the sample was dried by flowing dry N_2_ for 30 min (Fig. S5). Powder XRD analysis revealed that the new product is amorphous (hereafter named **a1**) with a very large bump covering the 10–30 range in 2*θ*/° Bragg diffraction angles and with the absence of sharp Bragg reflections (Fig. 2b). The diffuse scattering shows that there is short-range ordering that is different from the starting crystalline materials (Fig. 2). This differs from what was observed in the chloride and bromide versions of poly-[*n*]-catenanes, which upon instant synthesis using the same solvents, always yield crystalline phases. Notably, the structure of the amorphous phase is not known, but it can be inferred via an *amorphous*-*to*-*crystalline* phase transition if **a1** uptakes aromatic guest molecules producing the **M**_**12**_**L**_**8**_ poly-[*n*]-catenane periodic structure.

Therefore, **a1** was immersed in a toluene/methanol (4 ml:1 ml) solution in a round bottom flask and stirred for 12 h (overnight experiment). Then, the suspension was filtered, and the solid was analysed by powder XRD. The diffractogram showed that the amorphous phase **a1** transformed to a crystalline material, as sharp Bragg reflections were observed (Fig. 2c). The experimental powder XRD pattern corresponds to that of a poly-[*n*]-catenane. This indicates that the product obtained upon instant synthesis is the amorphous poly-[*n*]-catenane of interlocked **M**_**12**_**L**_**8**_ cages. It is important to recall that **a1** does not dissolve in toluene/methanol followed by recrystallization, yielding polycatenane, but traps the solvent in the **M**_**12**_**L**_**8**_ cages rearranged in an ordered manner.

Using TG analysis, we quantified the included guest molecules, which amount to 1.3 guest molecules in the asymmetric unit (considering only toluene) (13.8% weight loss) and 7.8 guests per **M**_**12**_**L**_**8**_ nanocage (Fig. 2d). ^1^H NMR of the sample analysed by TGA did not show any presence of methanol, thus corroborating that the weight loss corresponds to toluene (Fig. S6). The TG also provides information about the stability of the structure, and from 250 to 400 °C, there is no weight loss, indicating that the **M**_**12**_**L**_**8**_ poly-[*n*]-catenane is fully evacuated, and after *ca*. 400 °C, the material decomposes. Therefore, using nitrobenzene as a templating solvent does not lead to a crystalline poly-[*n*]-catenane or to another crystalline phase (*vide ante*) using the instant synthesis method, as observed by the repetition of three experiments. This behavior is different from that observed using ZnCl_2_ and ZnBr_2_, which formed crystalline **M**_**12**_**L**_**8**_ poly-[*n*]-catenane under the same experimental conditions.

*Instant synthesis* was also carried out using chlorobenzene and toluene as templating solvents following the same protocol as in nitrobenzene synthesis (see ESI). The obtained product in both cases is crystalline, but the quantity of product is small (low yields). Interestingly, the sample obtained with chlorobenzene remained crystalline after 1 month of synthesis (Fig. S9), with 19% weight loss of guest molecules included, as shown by TG (Fig. S10). However, the sample obtained using toluene as a template after one month was amorphous and included very few guests (6% weight loss with a poorly defined weight loss release step from TG (Fig. S8)). Clearly, these two examples of **TPB**-ZnI_2_ poly-[*n*]-catenanes have different stabilities depending on the solvent.

### Single crystal X-ray structure at room temperature of M_12_L_8_ poly-[*n*]-catenane using TPB and ZnI_2_ using nitrobenzene

The proof that the poly-[*n*]-catenane can be formed using **TPB** and ZnI_2_ using slow crystallization experiments has been carried out by preparing single crystals by self-assembling **TPB** and ZnI_2_. Pristine single crystals of **TPB**-ZnI_2_ poly-[*n*]-catenane were obtained by layering diffusion of Zn(II) to a solution of **TPB** in nitrobenzene. In a crystallization tube, as a bottom layer, **TPB** was dissolved in a nitrobenzene/MeOH mixture, and then as a middle layer, methanol was added until a clear biphasic solution was formed (Fig. S4). As the top layer, a methanolic solution of ZnI_2_ was added dropwise in such a way that no precipitate was observed during layering. The crystallization tube was left standing, and after 5 days, large crystals attached in the middle layer of the tube were obtained. A single crystal of good quality was selected and mounted for SC-XRD analysis. Importantly, the crystals covered in the mineral oil used to mount in the loop did not crack even after being in contact with the atmosphere for more than one hour. Thus, we attempted the structure solution at room temperature.

**Figure 3:**
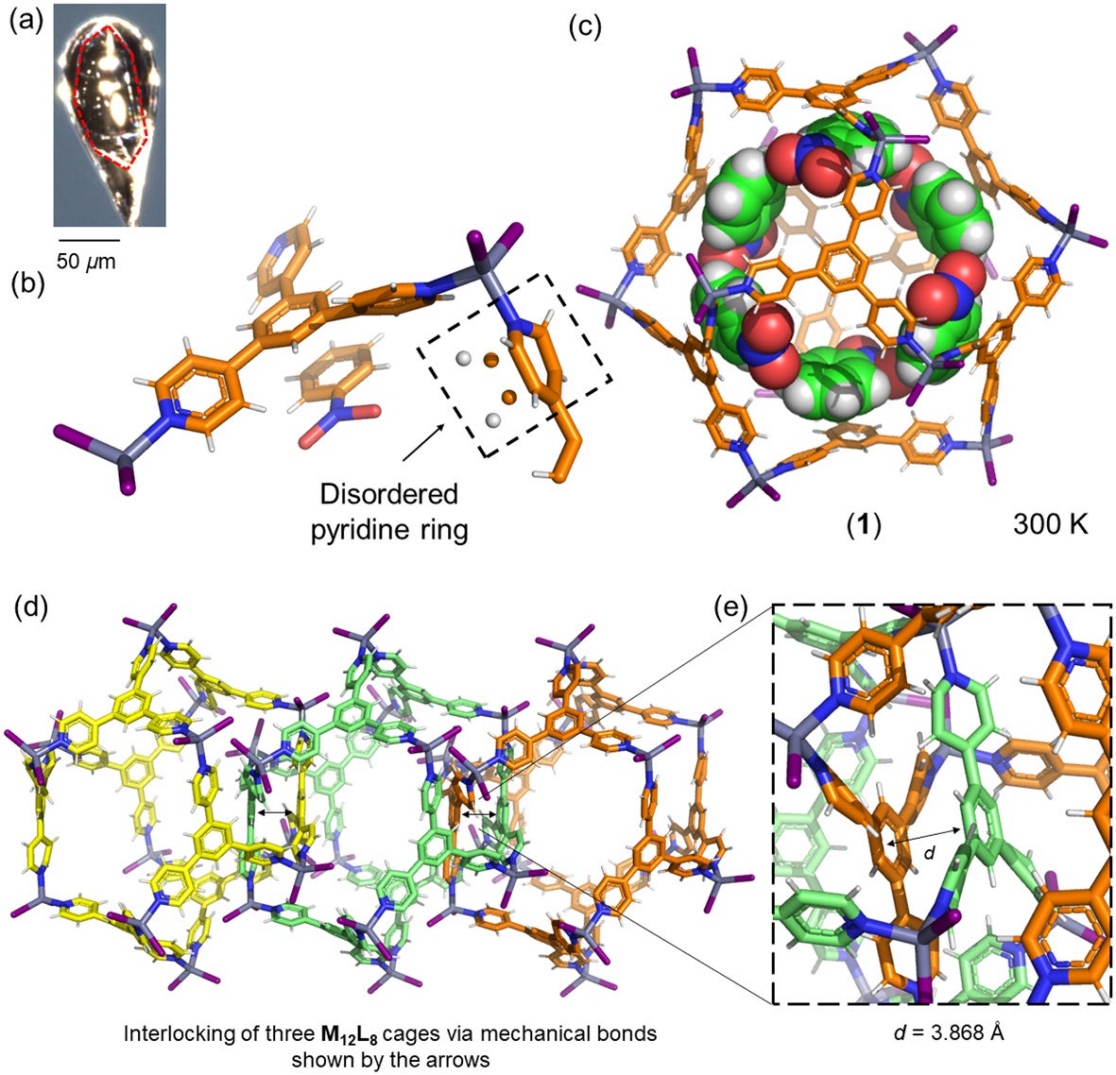
(**a**) A photo of a single crystal that was used for SC-XRD structure solution of **1**. (**b**) Asymmetric unit content showing the disordered pyridine ring and the included guest nitrobenzene. (**c**) Assembly of the **M**_**12**_**L**_**8**_ nanocage including the six ordered guest molecules having good electrostatic interactions with the cage. Color code host: Carbon: orange; Nitrogen: blue; Hydrogen: white; Zinc: gray; Iodide: Purple. Color code guest: Carbon: green; Nitrogen: blue; Hydrogen: white; Oxygen: red. (**d**) View of three **M**_**12**_**L**_**8**_ nanocages linked by the mechanical bond. (**e**) Expanded view displaying the aromatic-aromatic distance among **TPB** ligands in the interlocked cages. To show the three different **M**_**12**_**L**_**8**_ nanocages, the Carbon atoms are yellow, green and orange.

The single crystal X-ray structure (300 K) was solved in the trigonal space group *R*-3 with the following unit cell parameters: *a* = 38.6805(7) Å; *b* = 38.6805(7) Å; *c* = 16.0202(3) Å; *α* = *β* = 90° and *γ* = 120°; *V* = 20,757.9(8) Å^3^. Crystallographic analysis revealed the formula [(ZnI_2_)_12_(TPB)_8_]_6_(C_6_H_5_NO_2_) (**1**) with two ZnI_2_, one **TPB** ligand and one-third of a second **TPB** ligand in the asymmetric unit (Fig. 3b). The structure of **1** corresponds to a poly-[*n*]-catenane with interlocked **M**_**12**_**L**_**8**_ icosahedral cages. The large cages include one nitrobenzene in the asymmetric unit that can be resolved by X-ray crystallography (Fig. 3b). Reports of guest inclusion in small voids are widely reported due to efficient host–guest interactions, but the binding and precise three-dimensional (3D) resolution of guests in large cavities is very challenging due to the lack of good host–guest and/or guest-guest noncovalent interactions^28^. The simulated powder XRD pattern matches well with the reported **M**_**12**_**L**_**8**_ poly-[*n*]-catenane structures, indicating that the sample is isostructural (Fig. S1).

The **M**_**12**_**L**_**8**_ cage framework is formed of twelve Zn(II) metal centers at the vertices of the icosahedron with a tetrahedral geometry with three Zn-N bonds (2.046 Å, 2.058 Å, and 2.052 Å) and four Zn-I bonds ranging from 2.523 to 2.563 Å. The icosahedron is defined as “*opened icosahedrons*”^20^ due to the large windows formed by the absence of 12 of the 20 **TPB** ligands forming the faces (triangles) of the icosahedron. This large window allows good interpenetration of **M**_**12**_**L**_**8**_ cages and thus mechanical bond formation, resulting in 1D chains of interlocked icosahedral cages. The 1D chains extend along the crystallographic *c*-axis and pack in the crystalline state in such a way that the interactions among the rods are through weak C–H···I–Zn electrostatic interactions.

The *mechanical bond* among the cages takes place because of the efficient *π*-stacking between the benzenic core of the **TPB** ligand (*d* = 3.868 Å) of adjacent cages. The good benzene-benzene interactions among **TPB** ligands are one of the key factors contributing to the formation under *kinetic control*^18^ of the poly-[*n*]-catenanes of interlocked **M**_**12**_**L**_**8**_ nanocages through mechanical bonding. This aspect is favourable from the enthalpic point of view. It is known that switching from benzene to triazine core in the exotridentate ligand (i.e., 2,4,6-tri(4-pyridyl)-1,3,5-triazine (**TPT**)), the outcome of the self-assembly is a completely different structure that does not form the **M**_**12**_**L**_**8**_ interlocked cages but a 3D coordination polymer with doubly interpenetrated (10,3-*b*) networks^29^.

**Figure 4:**
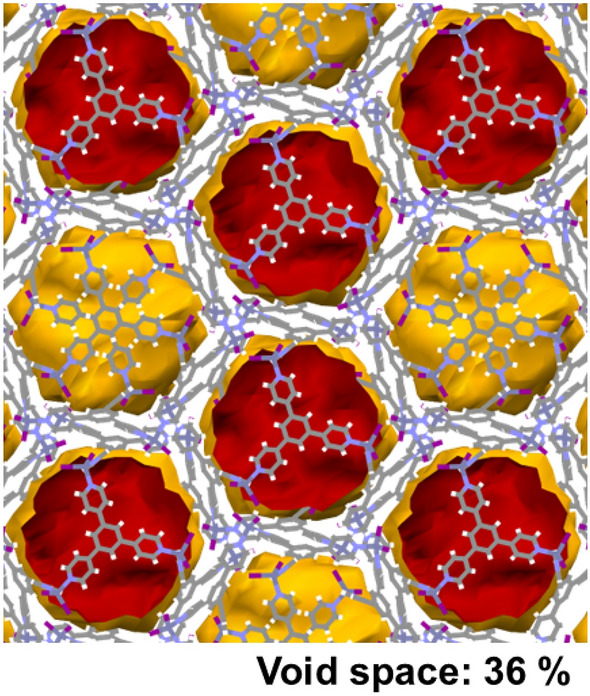
Picture showing the isolated voids in **1**. The nitrobenzene guest molecules were removed manually to show the available space occupied by the guest molecules, which amounted to 36% of the total cell volume. X-ray data measured at room temperature. Color code: Carbon: gray; Nitrogen: blue; Hydrogen: white; Zinc: gray; Iodide: Purple.

The packing of the 1D chains of interlocked **M**_**12**_**L**_**8**_ cages does not lead to a porous structure but an array of isolated cages with large internal volumes of *ca*. 2600 Å^3^ (Fig. 4). The volume occupied by the solvent molecules corresponds to 36% of the total cell volume (7553 Å^3^), similar to the other reported isostructural structures^18–21^. Interestingly, the interchain interactions for the zinc iodide catenane have an important role in the structure stabilization. In fact, terminal ligand I in the Zn metal interacts with the aromatic rings of **TPB** via C–H···I interactions with distances of 3.169 Å and 3.145 Å from adjacent chains.

Even though the crystal structure has been solved at room temperature, the **M**_**12**_**L**_**8**_ cages can be resolved with reasonably small atomic thermal parameters. One of the four pyridine rings in the asymmetric unit is disordered over two positions, which is important to explain the guest exchange reactions despite not having continuous channels (Fig. 4)^20^. Pyridine ring motion along with the dynamic behavior of the 1D chains of interlocked **M**_**12**_**L**_**8**_ cages has been proposed to facilitate guest exchange and the dynamic behavior of **M**_**12**_**L**_**8**_ poly-[*n*]-catenane systems^20^.

**Figure 5:**
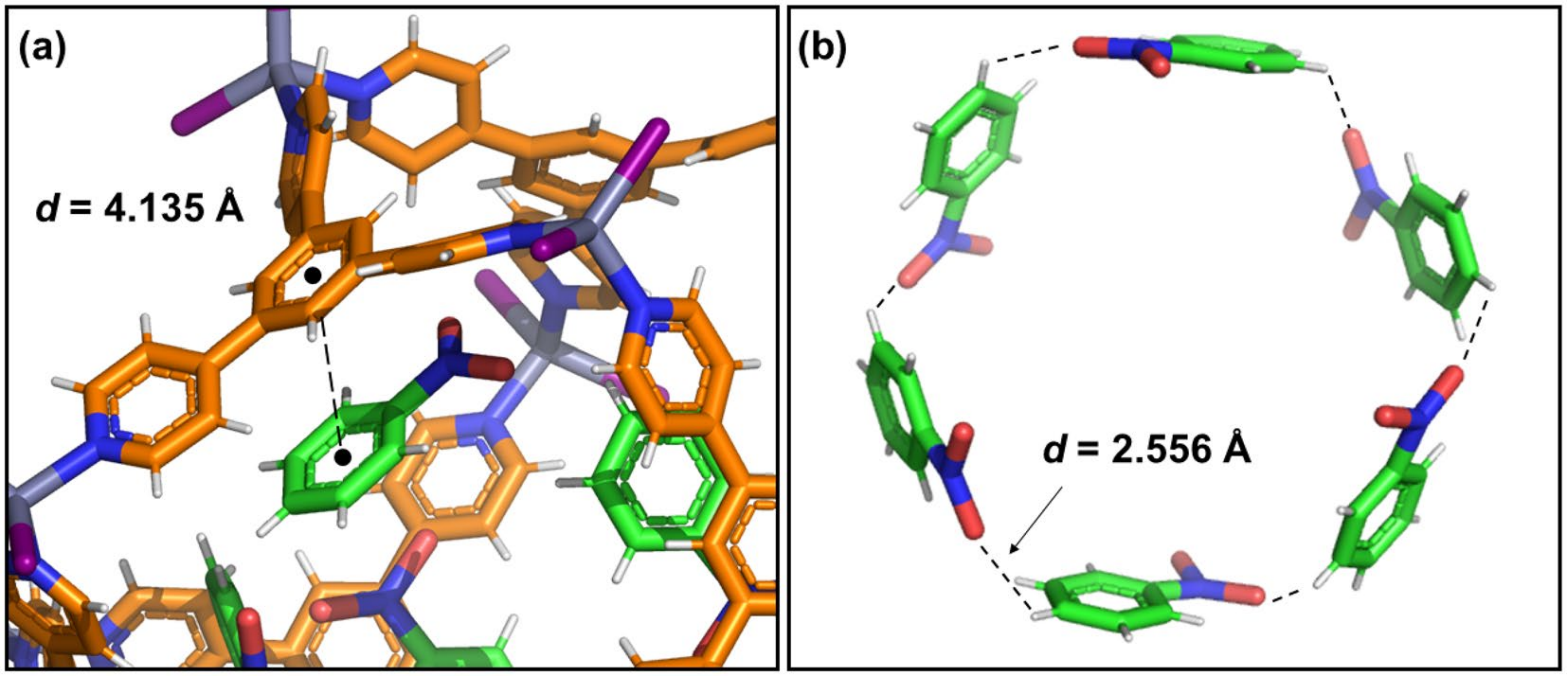
Crystal structure of poly-[*n*]-catenane (**1**) showing the host–guest interactions among the benzene ring of **TPB** and the benzene ring of nitrobenzene. Benzene-host ···benzene-guest is depicted by the black dashed line (**a**). Guest-guest interactions within the **M**_**12**_**L**_**8**_ nanocages shown as dashed lines (**b**). Color code as in Fig. 3.

The nitrobenzene guest interacts with the **TPB** host framework via aromatic-aromatic interactions. The host–guest distance among the centroids of the ring in nitrobenzene and the benzene ring of the **TPB** ligand is 4.135 Å (Fig. 5a). Nitrobenzene is oriented with the part of the guest molecule that is more electropositive towards the more electronegative part of the coordinated **TPB** ligand according to the maps of electrostatic potential (MEPs) for the nitrobenzene guest and **TPB** host, as shown in Fig. 3^21^.

There are additional electrostatic interactions involving the nitrobenzene guests among the aromatic C–H and one oxygen atom from the –NO_2_ group. The C–H···O distance is 2.556 Å, which, from an enthalpic point of view, contributes to the stabilization of the **M**_**12**_**L**_**8**_ cage (Fig. 5b). The oxygen that is engaged in the electrostatic interaction is shorter in the -NO_2_ group, indicating its involvement in the stabilization of the guests within the **M**_**12**_**L**_**8**_ nanocage.

### Screening of single crystals of self-assembling TPB and ZnI_2_ in various aromatic solvents

To gauge more structural information on the self-assembly of **TPB** and ZnI_2_ and to compare the results from *instant synthesis*, several slow crystallization experiments were carried out with **TPB** and ZnI_2_ with various aromatic solvents acting as templating agents (Fig. S4). The solvents screened were 1,2-dichlorobenzene, toluene and chlorobenzene. Interestingly, in all the crystallization tubes, the **M**_**12**_**L**_**8**_ poly-[*n*]-catenanes are formed (as indicated by the unit cells obtained, which were all trigonal with lattice parameters similar to those of **1**), but in all cases, different crystal structures were identified, as evidenced by the different unit cells.

**Figure 6:**
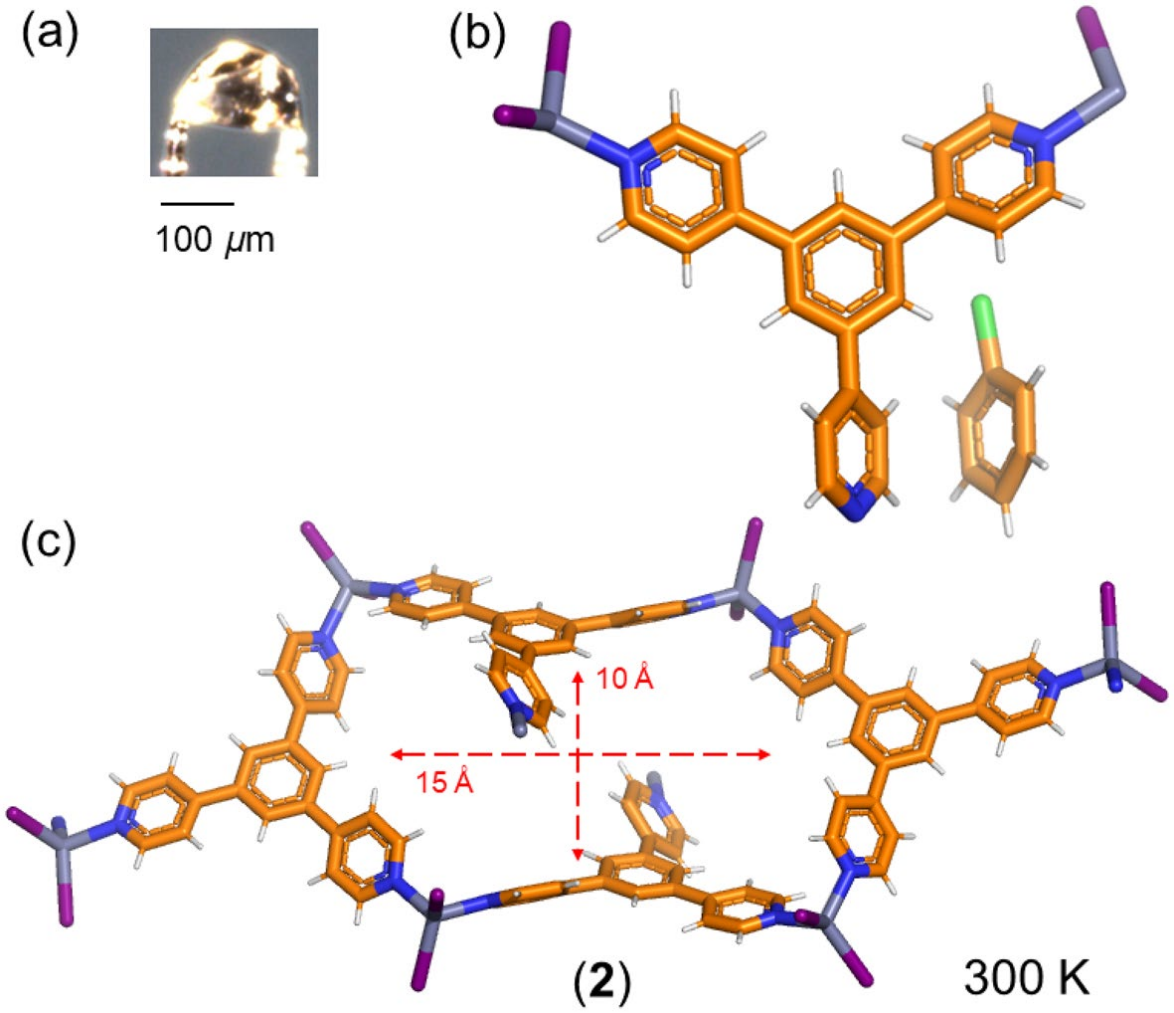
(**a**) A photo of a single crystal that was used for SC-XRD of **2.** (**b**) View of the asymmetric unit with one **TPB** ligand 1.5 ZnI_2_ and one chlorobenzene guest molecule. (**c**) The minimum circuits are formed of four **TPB** ligands and four metal centers. The crystal structure is determined at room temperature and does not show disorder.

Slow crystallization by the layering diffusion method using chlorobenzene as the templating solvent gave, after 5 days, a mixture of two macroscopically different crystals: (i) **M**_**12**_**L**_**8**_ poly-[*n*]-catenane, which was confirmed by the unit cell parameters as block-like crystals, and (ii) a new colourless crystalline phase (**2**) easily distinguishable due to its thin-plate habit. Crystals of **2** (covered with mineral oil) are stable at room temperature for several hours; therefore, due to their thermal stability, SC-XRD was recorded at room temperature (300 K). The thin plates (Fig. 6a) have lattice parameters and space group symmetry different from those of **1**: *a* = 24.1055(4) Å, *b* = 14.4843(2) Å, *c* = 18.2201(2) Å, *β* = 100.8630(10), and *V* = 6247.57(15) Å^3^, crystallizing in the monoclinic system in the *C*2/*c* space group (Table S2).

In the asymmetric unit, there is one **TPB** ligand and one and a half ZnI_2_, as one of the Zn atoms sits at a special position on a twofold axis. There is also one chlorobenzene guest molecule (Fig. 6b). The chemical formula, according to the SC-XRD data, is [(**TPB**)_1_(ZnI_2_)_1.5_]_*n*_·(C_6_H_5_Cl) (**2**). The minimum metal–ligand circuit is formed by four **TPB** ligands and four Zn(II) metal centers expanding along the *c*-axis, which leads to infinite 1D channels, including chlorobenzene. The smallest **TPB**-ZnI_2_ circuits form pockets of rectangular shape with windows of *ca*. 10 Å × 15 Å dimensions (Fig. 6c).

**Figure 7:**
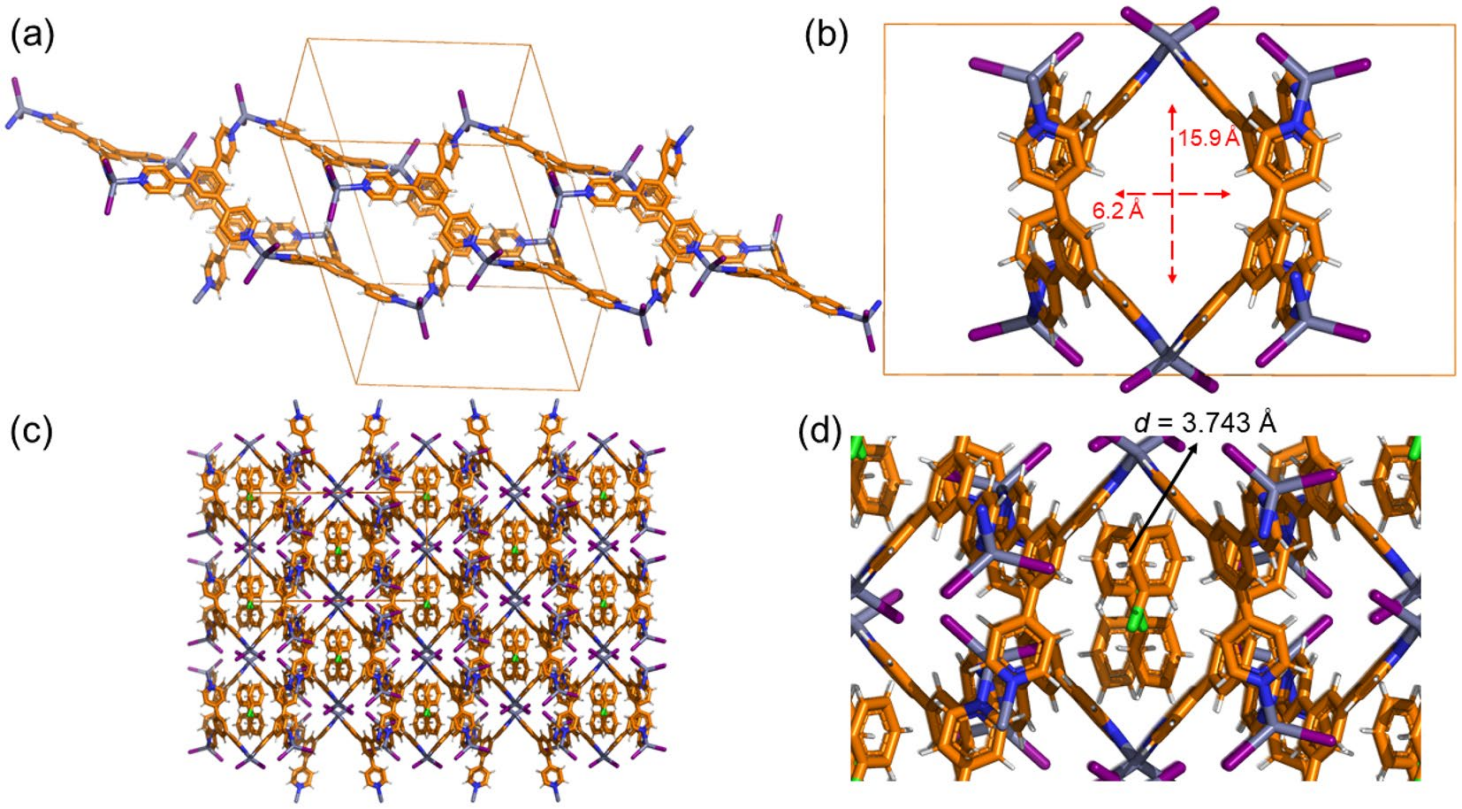
Single crystal X-ray structure of **2** depicting (**a**) the propagation of the tubular structure formed by the circuits; (**b**) view of the tube structure along the *c*-axis; (**c**) the packing of the tubular structures viewed along the *c*-axis including the guest molecules; and (**d**) expanded view of the 1D channel structure (tubular) including the solvent dichlorobenzene molecules.

The minimum circuits expand through the other two Zn-N coordination bonds to give a tubular structure expanding along the *c*-axis (Fig. 7a,b). The tubes are large enough to form 1D channels of dimensions *ca*. 16 Å × 6 Å. Adjacent 1D chains expand along the *a*- and *b*-axes via aromatic (pyridine) C–H···I interactions (*d* = 3.045 Å). The included guest does not form a particular interaction with the host structure but guest-guest interactions through *π*–*π* electrostatic contacts with distances among the centroids of each chlorobenzene of 3.743 Å (Fig. 7c,d). The close guest-guest interactions contribute to the stabilization of the whole structure. Therefore, **2** can be regarded as a coordination polymer with a 1D channel structure with void space (void volumes are calculated using a spherical probe of 1.2 Å diameter)^30^ corresponding to 30% of the total unit cell volume (Fig. S3).

Importantly, if toluene is used as an aromatic templating solvent instead of chlorotoluene, a mixture of crystals is also obtained where the **M**_**12**_**L**_**8**_ poly-[*n*]-catenane (large and stable blocks) coexists with a coordination polymer (thin plates) with unit cell parameters similar to those of the crystal including chlorobenzene: *a* = 29.721(4) Å, *b* = 13.3437(15) Å, *c* = 17.2043(15) Å, *β* = 99.804(17), *V* = 6723 Å^3^. Details of this structure along with other coordination polymers will be reported elsewhere.

### Room temperature kinetic control in the synthesis of M_12_L_8_ poly-[*n*]-catenane using TPB and ZnI_2_ using nitrobenzene

**Figure 8:**
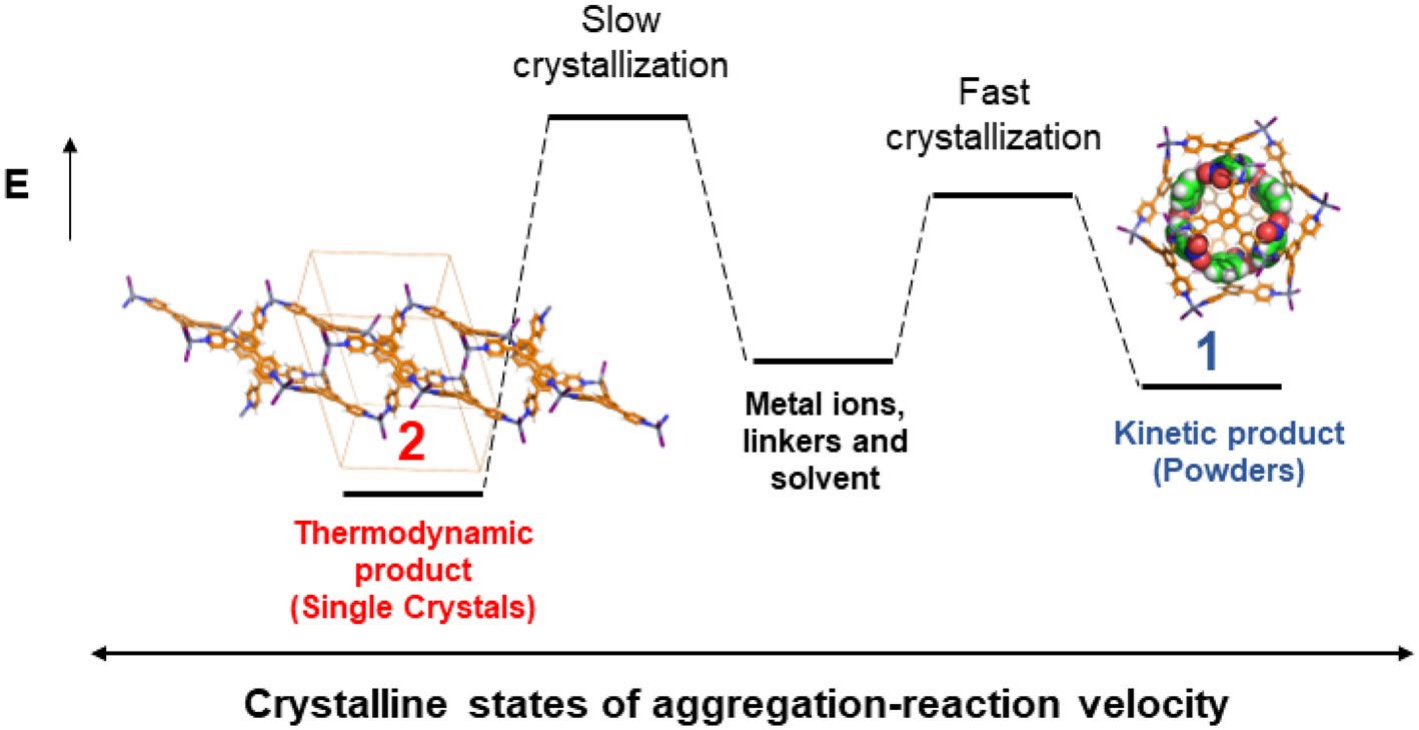
Kinetic control for the synthesis of **M**_**12**_**L**_**8**_ poly-[*n*]-catenane **1** using *instant synthesis*. Slow crystallization leads to the formation of thermodynamic structures such as coordination polymer **2**.

Our attempts to obtain coordination polymer **2** or any of the other coordination polymers observed using instant synthesis were unsuccessful. The **M**_**12**_**L**_**8**_ poly-[*n*]-catenane as *crystalline* or *amorphous* phases were always obtained instead of coordination polymers. This clearly demonstrates that *instant synthesis* crystallization is an effective method to prepare 1D **M**_**12**_**L**_**8**_ poly-[*n*]-catenane in a selective manner, as it does not allow the error-checking process^31^. The error-checking mechanism is possible due to the labile nature of the coordination bond. During slow crystallization (stratification method), the formation of alternative structures such as the abovementioned coordination polymers or other potential structures can take place because the coordination bond can be broken and reformed until the most thermodynamically stable structure is self-assembled (i.e., error checking). It is important to see that crystal structures **1** and **2** are completely different because the self-assembly process follows different crystallization conditions (Fig. 8). Kinetic products tend to form structures that have large voids^32^, as in **1**, while thermodynamic products form denser structures with smaller channels/pores, as in **2**. This can also be seen by the different densities of 1.690 g/cm^3^ and 1.904 g/cm^3^ in **1** and **2,** respectively, and the difference in void space (36% vs. 30%). Another important aspect is that *kinetic products* tend to be dynamic and hence can perform guest exchange/inclusion reactions but can also undergo further transformations towards more stable structures upon external stimuli (i.e., upon heating) following *crystal-to-amorphous-to-crystal* transformations^33^. Thus, the crystallization process has a direct effect on the final structure formed and is therefore very important to gain control. The ability to direct at will the product formed in a chemical reaction has a crucial role both in chemical synthesis and material sciences.

### Solid-state synthesis (neat grinding) of amorphous M_12_L_8_ poly-[*n*]-catenane using TPB and ZnI_2_

The solid-state synthesis of poly-[*n*]-catenanes is not common and even less common in systems such as **M**_**12**_**L**_**8**_ interlocked cages. While it has been demonstrated that it is possible to obtain **TPB**-ZnBr_2_ poly-[*n*]-catenane as an amorphous material, neither the **TPB**-ZnI_2_ nor **TPB**-ZnCl_2_ structures have yet been reported in the solid state following a solvent-free approach. Here, we report, for the first time, the synthesis of **TPB**-ZnI_2_ poly-[*n*]-catenane by means of *neat grinding*.

**TPB** and ZnI_2_ were mixed in a 1:1.5 molar ratio (**TPB** 30 mg: ZnI_2_ 47 mg) and ground using a mortar and pestle for 15 min. During the grinding process, the reagent and product mixtures were solid at all times. Product **a1′** (74 mg) was washed with a mixture of methanol (4 ml) and chloroform (4 ml) and left to equilibrate for 1 day. The weight after the washing process was 60 mg.

The yellowish solid **a1′** (Fig. S11) was analysed by powder XRD, which showed two broad bumps denoting that there is no *long-range* order but *only short-range* ordering, as in **a1** (Fig. S12). To corroborate that the **M**_**12**_**L**_**8**_ poly-[*n*]-catenane was formed, amorphous phase **a1′** was immersed in a mixture of MeOH/toluene and stirred for 4 h (see ESI). Then, the solid was filtered and immediately checked again by powder XRD. The similarity among the experimental powder XRD of the new crystalline phase (Fig. S13a) and the simulated single crystal XRD of **1** clearly indicates that **M**_**12**_**L**_**8**_ poly-[*n*]-catenane is obtained (Fig. S13b). The *amorphous*-to-*crystalline* transformation also occurs if **a1′** is exposed to vapours of methanol and aromatic guests (Fig. S14). The same synthetic approach was used for the solid-state synthesis of the TPB-ZnCl_2_ amorphous **M**_**12**_**L**_**8**_ poly-[*n*]-catenane (see ESI).

### Inclusion of xylene isomers by amorphous (a1′) TPB-ZnI_2_ M_12_L_8_ poly-[*n*]-catenane

Because toluene can be included in the **M**_**12**_**L**_**8**_ cages of the amorphous phase, our interest geared towards the inclusion of toluene derivatives such as the three isomers of xylenes by the uptake of **a1'** using heterogeneous solid–liquid reactions. The inclusion and separation of xylenes is an interesting topic in material sciences, as from an industrial point of view, it is relevant. Some reports have focused on the use of MOFs and discrete complexes (0D) for such separation^34–36^. To the best of our knowledge, the inclusion of xylenes in poly-[*n*]-catenanes, and in particular in **M**_**12**_**L**_**8**_ poly-[*n*]-catenanes, has not yet been reported. Thus, an amorphous polycatenane synthesized by neat grinding was used to adsorb *o*-xylene, *m*-xylene and *p*-xylene molecules using heterogeneous solid–liquid reactions at room temperature.

**Figure 9:**
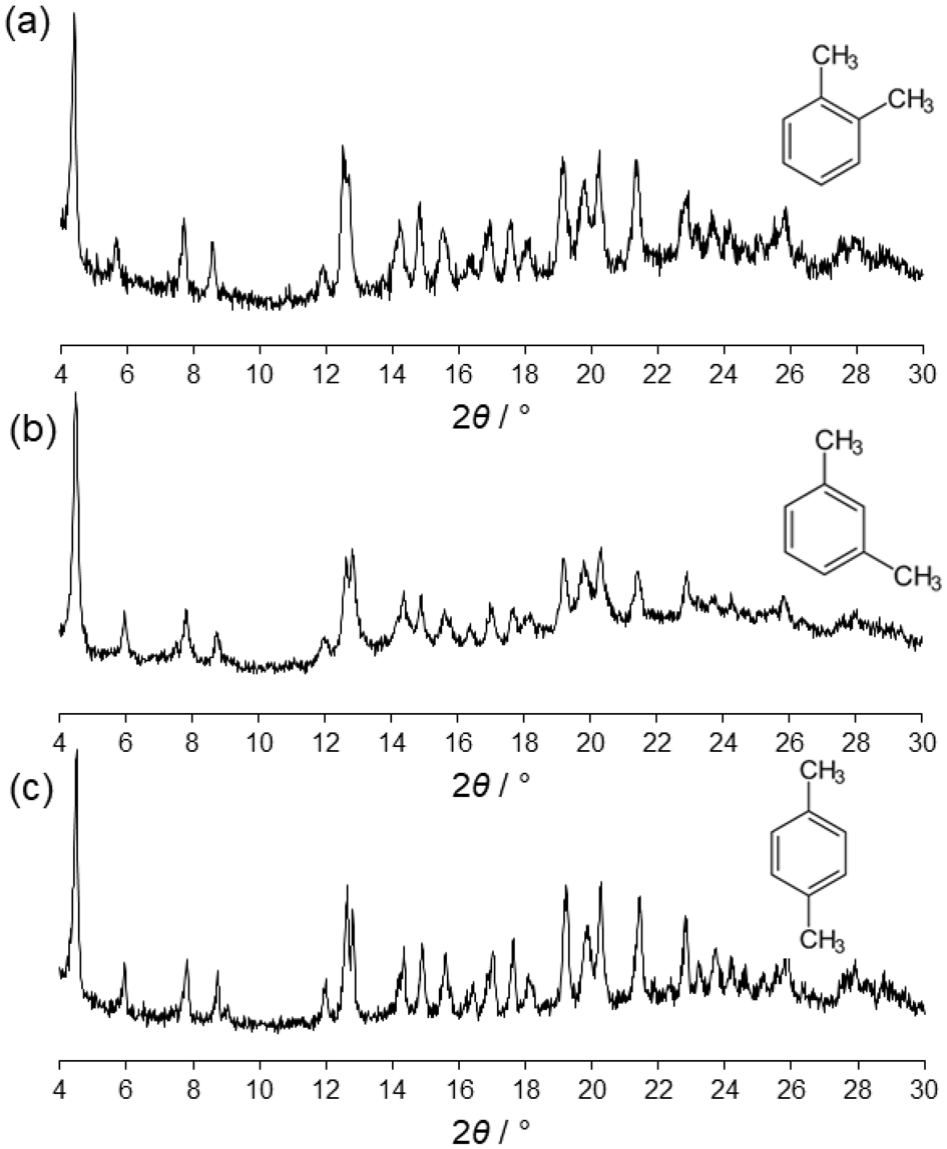
Experimental powder XRD patterns measured at room temperature of **a1′** exposed to *o*-xylene (**a**), **a1′** exposed to *m*-xylene (**b**) and **a1′** exposed to *p*-xylene.

In a typical experiment, 30 mg of **a1′** was immersed in 4 ml of *o*-xylene and 1 ml of methanol. The suspension was stirred overnight, filtered, and analysed by powder XRD analysis. As observed in the diffractograms, **a1′** transformed from an *amorphous* to a *crystalline* phase whose diffractogram corresponds to that of the **M**_**12**_**L**_**8**_ *poly*-[*n*]-catenanes (Fig. 9a). The same behavior is observed when *m*-xylene and *p*-xylene are used instead of *o*-xylene in the presence of amorphous **M**_**12**_**L**_**8**_ *poly*-[*n*]-catenane (Fig. 9b,c). The *amorphous*-*to*-*crystalline* transformation is a valid tool to differentiate if guest inclusion takes place (Fig. S18). Thus, the **a1′** phase can take up the three isomers, *para*-, *ortho*- and *meta*- xylenes, that are included in the large **M**_**12**_**L**_**8**_ nanocages.

Instant synthesis was also attempted using *o*-xylene, *m*-xylene and *p*-xylene. However, because of the poor solubility of **TPB** in xylenes, chloroform and methanol had to be used to obtain a homogenous solution of **TPB** for instant synthesis. The instant synthesis produced a low quantity of crystalline materials (≈ 10 mg). Thus, we retain that the use of xylenes as templating solvents in the *instant synthesis* is not ideal. The inclusion of xylenes in the **M**_**12**_**L**_**8**_ nanocages is much more efficient using amorphous phases **a1** or **a1′,** and from an industrial point of view, amorphous poly-[*n*]-catenane obtained in the solid state is preferable.

### Solid-state QM density functional theory (DFT) calculations of interaction energies among M_12_L_8_ nanocages, lattice energies, host–guest energies, and guest-guest energies

The availability of the room temperature structure of **1,** including nitrobenzene, allows DFT calculations to be carried out, giving insights into the structural stability and intrinsic local dynamic behavior of the **TPB**-ZnI_2_ **M**_**12**_**L**_**8**_ poly-[*n*]-catenane system. Therefore, DFT calculations specific for solid crystalline states have been carried out considering the energy interactions among **M**_**12**_**L**_**8**_ cages, among the 1D chains, and host–guest energy interactions. DFT calculations were carried out at the PBE/DNP level (where PBE is the functional of Perdew, Burke and Ernzerhof, DNP states for a standard numerical basis set inserted into the Dmol^3^ package, roughly comparable to the 6-31G** Gaussian set)^37^. The strategy adopted here was shown to be good in several recent studies of crystalline systems such as molecules, polymers, and hybrid metal–organic materials^38–44^. The explicit van der Waals contribution, according to the approach proposed by Grimme, was determined^45,46^.

It is important to define the model *system* to be adopted in DFT calculations. First, the “*interaction energies*” (***E***) among interlocked and noninterlocked **M**_**12**_**L**_**8**_ nanocages, which can give us a view of the stabilities of the 1D ribbons and of individual (i.e., isolated) **M**_**12**_**L**_**8**_ cages, are considered. The second aspect is the “*lattice energy*” (E*), which considers the energy of a single **M**_**12**_**L**_**8**_ cage or a 1D chain of interlocked cages *immersed* within the crystalline lattice (**1**). The third point considers the interaction energy among the **M**_**12**_**L**_**8**_ host and the guest and guest-guest interactions, referred to hereafter as ***E***_***host-guest***_ and ***E***_***guest-guest***_, respectively**.**

#### Interaction energy calculation (*E*)

***E*** can be analysed considering the interaction energies of two *close* interacting dimers: two interlocked **M**_**12**_**L**_**8**_ cages or two first neighbour cages that are not mechanically linked but that are both stable. The interaction energy into **1** considering the interlocked **M**_**12**_**L**_**8**_ cages is *ca.* 89 kcal/mol, which is almost 178 kcal/mol per 2 cages, and this interaction appears to be strongly affected by the aromatic ring interactions (*d* = 3.868 Å) from the central **TPB** ring. The interaction energies among the noninterlocked **M**_**12**_**L**_**8**_ cages that interact via van der Waals interactions correspond to 47 kcal/mol, or 23.5 kcal/mol per cage.

#### Lattice energy calculation (E*)

The average energy required to extract a single **M**_**12**_**L**_**8**_ cage immersed in the crystalline structure is very high: approximately 505 kcal/mol. In a similar way, if we consider the polycatenated chain along the *c*-axis (i.e., mechanically interlocked) and calculate the energy required to remove a single infinite chain of **M**_**12**_**L**_**8**_ interlocked nanocages from the crystalline structure, the calculated E* is 398 kcal/mol, indicating that the model of an infinite 1D chain “*immersed*” in the structure is a good model explaining the high stability of the poly-[*n*]-catenane architecture.

Because nitrobenzene has an important role in templating poly-[*n*]-catenane, we carried out the same sort of calculations but included the six crystallographic nitrobenzene guest molecules in the **M**_**12**_**L**_**8**_ cages. In this way, it is possible to compare the previously calculated energies against the empty model not including guest molecules. The interaction of the **M**_**12**_**L**_**8**_ cages with the solvent molecules, the ***E***_***host-guest***_*,* is also important in this case. The ***E***_***host-guest***_ is approximately 46 kcal/mol for each solvent molecule, which is comparable to the energies observed among the noninterlocked cages interacting via electrostatic and van der Waals interactions (i.e., the interactions among neighboring 1D chains). The ***E***_***guest-guest***_ interactions (Fig. 5b) are, as expected, much lower, 4.3 kcal/mol, but not negligible. Thus, the ***E***_***host-guest***_ and ***E***_***guest-guest***_ interaction energies of aromatic guests are very important for the stabilization of the whole poly-[*n*]-catenane structure. While the lattice energy (E*) values for a single cage and a single chain are higher and the interaction energies (***E***) for interlocked cages are also higher (Table 1), the interaction energy of noninterlocked cages shows a lower interaction energy, from 47 kcal/mol without guest to 27 kcal/mol including guests.

**Table 1:** Computed DFT energy interactions in **1** without (**TPB-ZnI**_**2**_) and with (**TPB-ZnI**_**2**_**(NB)**) included nitrobenzene (NB) solvent.

	TPB-ZnI_2_	TPB-ZnI_2_(NB)
Interaction energies (**E**)	Interlocked: 89 kcal/mol Noninterlocked: 47 kcal/mol	Interlocked: 111 kcal/mol Noninterlocked: 27 kcal/mol
Lattice energy (E*)	Single cage: 505 kcal/mol Chain: 398 kcal/mol	Single cage: 556 kcal/mol Chain: 417 kcal/mol
Host–guest energies **E**_**host-guest**_	TPB-NB: n.a.	TPB-NB: 46 kcal/mol

#### Interaction energies among 1D chains of interlocked M_12_L_8_ nanocages and the role of the included guest nitrobenzene

Among catenanes formed of molecular rings, 1D catenanes are very interesting because of their dynamic behavior due to their high conformational degrees of freedom through rotations, translations and rocking motions of the molecular rings^47^. However, when the chains are formed of interlocked **MOCs** such as the **M**_**12**_**L**_**8**_ large nanocages, the translation, rotation and rocking motions are somehow limited by the *free window* space left after mechanical bond formation. In the **M**_**12**_**L**_**8**_ poly-[*n*]-catenanes, the *free window* space is small, and therefore, the 1D rods are quite stable. Thermodynamically, the interlocking process is favoured from the enthalpic but not from the entropic point of view. We note that the 1D chains of interlaced **M**_**12**_**L**_**8**_ nanocages are not completely rigid, as it has been observed that guest molecules can be exchanged^20^, and stability studies demonstrated that included guest molecules come out from the cages under vacuum conditions^12^.

From the DFT calculations, it has been seen that almost all the interactions increase in the presence of guest molecules. This confirms that when the solvent is included in the **M**_**12**_**L**_**8**_ cages, the structure gains stability within the 1D chains. In particular, the energy interactions increase in single cages, the interlocked cages, and the chain of interlocked cages gaining stability, but the interactions among non-interlocked **M**_**12**_**L**_**8**_ cages, that is, among neighbouring chains, become weaker. In our opinion, the enhancement in structural stability brought by the 6 included and ordered guest interactions per **M**_**12**_**L**_**8**_ cage influences the electrostatic interactions (i.e., lowering) among chains for structure **1,** as observed by DFT. This has a direct effect on the guest exchange/inclusion properties and the dynamic behavior of the 1D chains of interlocked cages, which might help to explain the guest inclusion/exchange observed in these systems formed of large, interlocked cages.

It has been reported that the relative displacement among 1D chains of interlocked **M**_**12**_**L**_**8**_ cages considering two main directions, one perpendicular to the chain propagation (*c*-axis) and the second parallel to the chain extension (i.e., sliding chains), has a low energy cost. While a 1 Å displacement orthogonal to the 1D rod direction has an energy penalty of 10 kcal/mol, in the sliding direction, the energy penalty is 0.2 kcal/mol per 2 Å translation. These calculations were carried out considering a **TPB**-ZnBr_2_ model system of two **M**_**12**_**L**_**8**_ cages belonging to different chains (i.e., noninterpenetrated)^20^. The DFT results reported herein go in that direction, as the van der Waals interactions among neighboring chains are much weaker when the solvent is included in the DFT calculations.

## Conclusions

The self-assembly of poly-[*n*]-catenanes formed by the interlocking of large icosahedral **M**_**12**_**L**_**8**_ nanocages using **TPB** and ZnI_2_ as building blocks has been reported for the first time using three different crystallization methods. Fast crystallization by means of one-pot *instant synthesis* using nitrobenzene as the templating solvent gives amorphous poly-[*n*]-catenane phases. Compared to the other experiments using the same solvents where **TPB** is self-assembled with ZnCl_2_ or ZnBr_2_ using instant synthesis that yields crystalline poly-[*n*]-catenane, the **TPB**-ZnI_2_ system using nitrobenzene as the templating solvent yields an amorphous phase (**a1**). Importantly, fast crystallization takes place under *kinetic control*, homogeneously producing **M**_**12**_**L**_**8**_ poly-[*n*]-catenane. Instead, the slow crystallization by layering solution allows the formation of large single crystals of interlocked **M**_**12**_**L**_**8**_ poly-[*n*]-catenane including nitrobenzene (**1**), whose structure has been determined at room temperature conditions allowing unambiguous resolution of the guest nitrobenzene. In contrast to *kinetic* control, under *thermodynamic* conditions, the products are not obtained selectively but as mixtures. Slow crystallization under thermodynamic control using different templating solvents yields a mixture of the **M**_**12**_**L**_**8**_ poly-[*n*]-catenane and 1D coordination polymers (**2**), including aromatic guest molecules that yield 1D channels. The **M**_**12**_**L**_**8**_ poly-[*n*]-catenanes have also been synthesized in the solid-state in the absence of solvent as an amorphous phase by *neat grinding*. In the presence of aromatic guest molecules, the amorphous phase **a1′** becomes crystalline following an *amorphous-to-crystalline* transformation by using suitable guests such as *o*-DCB, toluene, *o*-xylene, *m*-xylene or *p*-xylene. Solid-state DFT calculations were carried out to analyze the stability of **M**_**12**_**L**_**8**_ poly-[*n*]-catenane **1** with and without nitrobenzene guests. Considering that the MOC cavity is considerably larger than the guest molecule, the host–guest interactions are highly specific, allowing its binding within the cage. The DFT analysis allowed us to rationalize the importance of the guest effect in the stabilization of the 1D chains of interlocked cages. The lattice energies of interlocked and noninterlocked **M**_**12**_**L**_**8**_ nanocages as well as host–guest and guest-guest energy interactions were calculated. The results suggest that while interactions within the 1D chains are enhanced by the presence of guests, the electrostatic interactions among the chains are less intense. The lower local van der Waals interchain interactions observed in **1** add additional proof to the dynamic behavior described in isostructural **M**_**12**_**L**_**8**_ poly-[*n*]-catenanes allowing 1D chain displacement and guest exchange properties in structures that do not contain channels.

The guest uptake ability by the amorphous phase of the poly-[*n*]-catenane formed in the solid-state shows good potential for functional applications of this class of mechanically interlocked materials in areas such as molecular separation of xylene isomers but can also find applications for including other guests such as drugs for drug delivery purposes.

## Supplementary Information

Supplementary Information 1.

Supplementary Information 2.

Supplementary Information 3.

Supplementary Information 4.

Supplementary Information 5.

## Data Availability

All data generated or analyzed during this study are included in this published article (and its Supplementary Information files). The single-crystal X-ray datasets **1** and **2** generated and analysed during the current study are available from The Cambridge Crystallographic Data Centre (CCDC) repository (https://www.ccdc.cam.ac.uk). The accession codes for **1** and **2** are 2233049 and 2233048, respectively.
