# Supplementary material for: Kinetic trapping of 2,4,6-tris(4-pyridyl)benzene and ZnI2 into M12L8 poly-[n]-catenanes using solution and solid-state processes
Source: Sci Rep. 2023 Apr 5;13:5605. doi: 10.1038/s41598-023-32661-x (PMC10076325; doi:10.1038/s41598-023-32661-x)

checkCIF/PLATON report

Structure factors have been supplied for datablock(s) shelx

THIS REPORT IS FOR GUIDANCE ONLY. IF USED AS PART OF A REVIEW PROCEDURE FOR PUBLICATION, IT SHOULD NOT REPLACE THE EXPERTISE OF AN EXPERIENCED CRYSTALLOGRAPHIC REFEREE.

No syntax errors found. CIF dictionary Interpreting this report

Datablock: shelx

Bond precision:	C-C = 0.0201 A	Wavelength=1.54184	
Cell:	a=24.1055(4)	b=14.4843(2)	c=18.2201(2)
	alpha=90	beta=100.863(1)	gamma=90
Temperature:	304 K		

	Calculated	Reported
Volume	6247.58(15)	6247.57(15)
Space group	C 2/c	C 2/c
Hall group	-C 2yc	-C 2yc
Moiety formula	C42 H30 I6 N6 Zn3, 2(C6 H5 Cl)	?
Sum formula	C54 H40 Cl2 I6 N6 Zn3	C54 H40 Cl2 I6 N6 Zn3
Mr	1801.39	1801.33
Dx,g cm-3	1.915	1.915
Z	4	4
Mu (mm-1)	25.704	25.704
F000	3392.0	3392.0
F000'	3377.51	
h,k,lmax	30,18,22	30,17,22
Nref	6583	6171
Tmin,Tmax	0.173,0.214	0.193,1.000
Tmin'	0.051	

Correction method= # Reported T Limits: Tmin=0.193 Tmax=1.000
AbsCorr = MULTI-SCAN

Data completeness= 0.937 Theta(max)= 76.599

R(reflections)= 0.0797(4954) wR2(reflections)= 0.2560(6171)

S = 1.105 Npar= 286

The following ALERTS were generated. Each ALERT has the format
test-name_ALERT_alert-type_alert-level.
Click on the hyperlinks for more details of the test.

Alert level B

```
PLAT342_ALERT_3_B Low Bond Precision on  C-C Bonds ...............    0.02015 Ang.
```

Alert level C

```
SHFSU01_ALERT_2_C  The absolute value of parameter shift to su ratio > 0.05
            Absolute value of the parameter shift to su ratio given   0.065
            Additional refinement cycles may be required.
PLAT080_ALERT_2_C Maximum Shift/Error ...........................       0.06 Why ?
PLAT084_ALERT_3_C High wR2 Value (i.e. > 0.25) ..................       0.26 Report
PLAT202_ALERT_3_C Isotropic non-H Atoms in Anion/Solvent .........         7 Check
              Cl30    C2      C3      C4      C5      C12               etc.
PLAT234_ALERT_4_C Large Hirshfeld Difference C14      --C15      .       0.16 Ang.
PLAT241_ALERT_2_C High   'MainMol' Ueq as Compared to Neighbors of        C16 Check
PLAT242_ALERT_2_C Low    'MainMol' Ueq as Compared to Neighbors of        Zn4 Check
PLAT242_ALERT_2_C Low    'MainMol' Ueq as Compared to Neighbors of        Zn5 Check
PLAT242_ALERT_2_C Low    'MainMol' Ueq as Compared to Neighbors of        N12 Check
PLAT242_ALERT_2_C Low    'MainMol' Ueq as Compared to Neighbors of        C15 Check
PLAT243_ALERT_4_C High   'Solvent' Ueq as Compared to Neighbors of         C4 Check
PLAT243_ALERT_4_C High   'Solvent' Ueq as Compared to Neighbors of        C18 Check
PLAT244_ALERT_4_C Low    'Solvent' Ueq as Compared to Neighbors of         C2 Check
PLAT244_ALERT_4_C Low    'Solvent' Ueq as Compared to Neighbors of         C3 Check
PLAT244_ALERT_4_C Low    'Solvent' Ueq as Compared to Neighbors of         C5 Check
PLAT244_ALERT_4_C Low    'Solvent' Ueq as Compared to Neighbors of        C12 Check
PLAT250_ALERT_2_C Large U3/U1 Ratio for Average U(i,j) Tensor ....        2.5 Note
PLAT601_ALERT_2_C Unit Cell Contains Solvent Accessible VOIDS of .         38 Ang**3
PLAT906_ALERT_3_C Large K Value in the Analysis of Variance ......      4.247 Check
PLAT911_ALERT_3_C Missing FCF Refl Between Thmin & STh/L=    0.600         34 Report
PLAT971_ALERT_2_C Check Calcd Resid. Dens.  1.37Ang From I3             1.89 eA-3
PLAT971_ALERT_2_C Check Calcd Resid. Dens.  1.00Ang From I1             1.80 eA-3
PLAT971_ALERT_2_C Check Calcd Resid. Dens.  0.91Ang From I2             1.80 eA-3
PLAT972_ALERT_2_C Check Calcd Resid. Dens.  1.01Ang From I3            -1.75 eA-3
PLAT977_ALERT_2_C Check Negative Difference Density on H3        .      -0.38 eA-3
```

Alert level G

```
PLAT002_ALERT_2_G Number of Distance or Angle Restraints on AtSite          7 Note
PLAT004_ALERT_5_G Polymeric Structure Found with Maximum Dimension          1 Info
PLAT083_ALERT_2_G SHELXL Second Parameter in WGHT  Unusually Large      30.49 Why ?
PLAT172_ALERT_4_G The CIF-Embedded .res File Contains DFIX Records          8 Report
PLAT173_ALERT_4_G The CIF-Embedded .res File Contains DANG Records          2 Report
PLAT174_ALERT_4_G The CIF-Embedded .res File Contains FLAT Records          1 Report
PLAT232_ALERT_2_G Hirshfeld Test Diff (M-X)  I2       --Zn4      .        5.8 s.u.
PLAT794_ALERT_5_G Tentative Bond Valency for Zn4       (II)      .       2.03 Info
PLAT794_ALERT_5_G Tentative Bond Valency for Zn5       (II)      .       2.06 Info
PLAT860_ALERT_3_G Number of Least-Squares Restraints .............         13 Note
PLAT883_ALERT_1_G No Info/Value for _atom_sites_solution_primary .     Please Do !
PLAT912_ALERT_4_G Missing # of FCF Reflections Above STh/L=  0.600        364 Note
PLAT941_ALERT_3_G Average HKL Measurement Multiplicity ...........        4.8 Low
PLAT965_ALERT_2_G The SHELXL WEIGHT Optimisation has not Converged     Please Check
```

```
PLAT978_ALERT_2_G Number C-C Bonds with Positive Residual Density.          0 Info
```

```
   0 ALERT level A = Most likely a serious problem - resolve or explain
   1 ALERT level B = A potentially serious problem, consider carefully
  25 ALERT level C = Check. Ensure it is not caused by an omission or oversight
  15 ALERT level G = General information/check it is not something unexpected

   1 ALERT type 1 CIF construction/syntax error, inconsistent or missing data
  19 ALERT type 2 Indicator that the structure model may be wrong or deficient
   7 ALERT type 3 Indicator that the structure quality may be low
  11 ALERT type 4 Improvement, methodology, query or suggestion
   3 ALERT type 5 Informative message, check
```

It is advisable to attempt to resolve as many as possible of the alerts in all categories. Often the minor alerts point to easily fixed oversights, errors and omissions in your CIF or refinement strategy, so attention to these fine details can be worthwhile. In order to resolve some of the more serious problems it may be necessary to carry out additional measurements or structure refinements. However, the purpose of your study may justify the reported deviations and the more serious of these should normally be commented upon in the discussion or experimental section of a paper or in the "special_details" fields of the CIF. checkCIF was carefully designed to identify outliers and unusual parameters, but every test has its limitations and alerts that are not important in a particular case may appear. Conversely, the absence of alerts does not guarantee there are no aspects of the results needing attention. It is up to the individual to critically assess their own results and, if necessary, seek expert advice.

Publication of your CIF in IUCr journals

A basic structural check has been run on your CIF. These basic checks will be run on all CIFs submitted for publication in IUCr journals (*Acta Crystallographica*, *Journal of Applied Crystallography*, *Journal of Synchrotron Radiation*); however, if you intend to submit to *Acta Crystallographica Section C* or *E* or *IUCrData*, you should make sure that full publication checks are run on the final version of your CIF prior to submission.

Publication of your CIF in other journals

Please refer to the *Notes for Authors* of the relevant journal for any special instructions relating to CIF submission.

PLATON version of 28/11/2022; check.def file version of 28/11/2022

Datablock shelx - ellipsoid plot

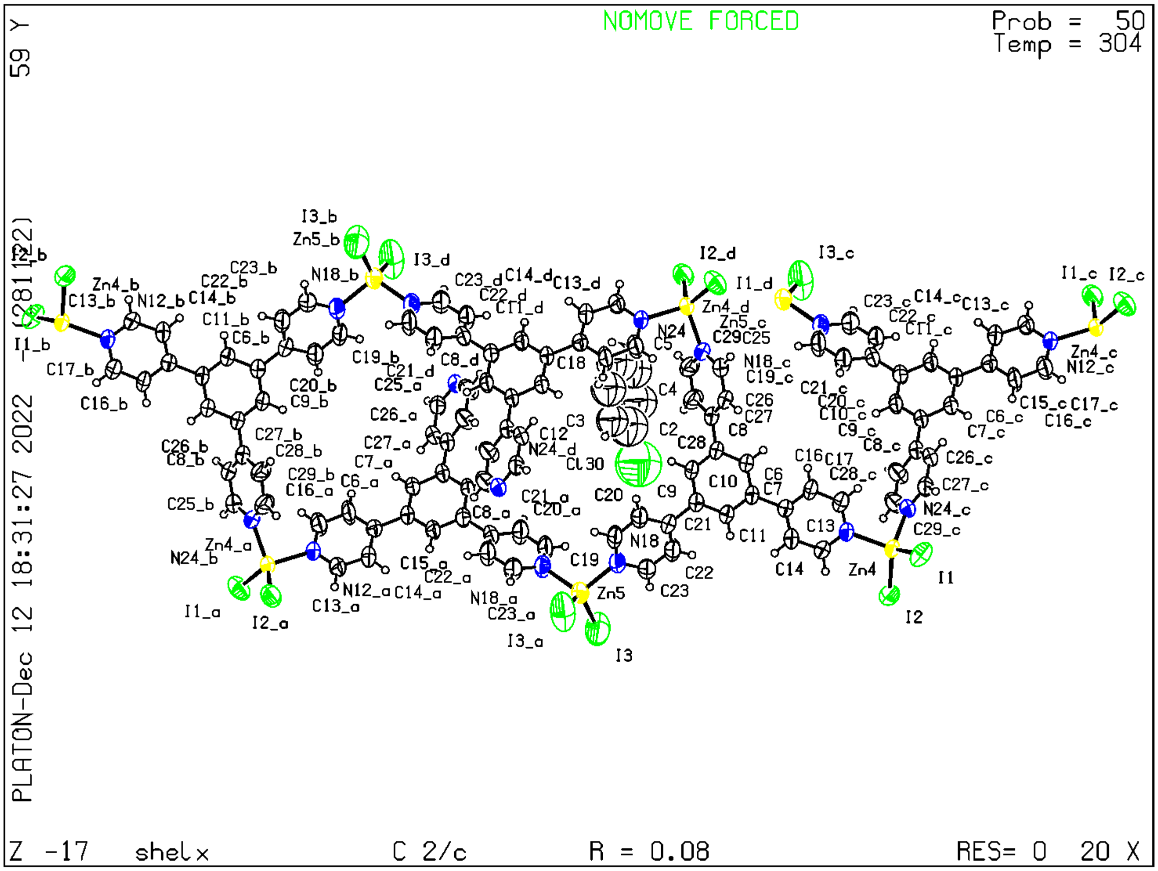

Supplement: Supplementary file 1 — Supplementary Information 1. [file 41598_2023_32661_MOESM1_ESM.pdf]
