# Supplementary material for: Kinetic trapping of 2,4,6-tris(4-pyridyl)benzene and ZnI2 into M12L8 poly-[n]-catenanes using solution and solid-state processes
Source: Sci Rep. 2023 Apr 5;13:5605. doi: 10.1038/s41598-023-32661-x (PMC10076325; doi:10.1038/s41598-023-32661-x)
Supplement: Supplementary file 3 — Supplementary Information 3. [file 41598_2023_32661_MOESM3_ESM.docx]

“Kinetic Trapping of 2,4,6-Tris(4-pyridyl)benzene and ZnI_2_ into M_12_L_8_ poly-[*n*]-catenanes using solution and solid-state processes”

Javier Martí-Rujas*^,†,⁋^, Stefano Elli^†^, Antonino Famulari^†#^

† Dipartimento di Chimica Materiali e Ingegneria Chimica ‘‘Giulio Natta’’, Politecnico di Milano, Via L. Mancinelli 7, 20131 Milan, Italy.

⁋ Center for Nano Science and Technology@Polimi, Istituto Italiano di Tecnologia, Via Pascoli 70/3, 20133 Milano, Italy.

# INSTM Consorzio Interuniversitario Nazionale per la Scienza e Tecnologia dei Materiali

*E-mail: [javier.marti@polimi.it](mailto:javier.marti@polimi.it)

**Contents**

**Materials and methods**

**Single crystal XRD of 1 and 2**

**Powder X-Ray Diffraction Experiments**

**TG experiments**

**Density Functional Theory Calculations**

**Slow crystallization experiments using TPB and ZnI_2_ in various aromatic solvents: nitrobenzene, toluene, chlorobenzene.**

**Instant synthesis using TPB and ZnI_2_ in various aromatic solvents: nitrobenzene, toluene, chlorobenzene.**

**Solid-state synthesis of amorphous poly-[*n*]-catenane (a1').**

**Gas-solid phase *amorphous-to-crystalline* transformation using a1'.**

**Amorphous-to-crystalline transformation by uptake of xylenes.**

**References**

**Materials and Methods.**

**Single crystal XRD of 1 and 2.**

The single crystal XRD experiments were carried out at the NeXt GAME Laboratory of the Politecnico di Milano, co-founded by Regione Lombardia.

Single crystal X-ray data of poly-[*n*]-catenane **1** and **2** were recorded using a XtaLAB Synergy-S, Dualflex, HyPix-6000HE diffractometer. Single block-shaped crystals of **1** and plate-like crystals of **2** were obtained in the same crystallization tube using the three-layering method as shown in Figure S1. A suitable crystal of each sample **1** and **2** were selected and mounted on a suitable support on a XtaLAB Synergy-S, Dualflex, HyPix-6000HE diffractometer. The crystal was kept at a steady T ≈ 300 K during data collection. The structure was solved with the ShelXT^[[1]](#endnote-1)^ structure solution program using the Intrinsic Phasing solution method and by using Olex2^[[2]](#endnote-2)^ as the graphical interface. The model was refined with version 2014/7 of ShelXL 2014/7^1^ using Least Squares minimization. The structures were solved and the space group *R*-3 for **1** and *C*2/*c* for **2** determined by the ShelXT^1^ structure solution program using Intrinsic Phasing and refined by Least Squares using version 2014/7 of ShelXL 2014/7.^1^ All non-hydrogen atoms were refined anisotropically. Hydrogen atom positions were calculated geometrically and refined using the riding model.

Crystal Data (**1**). C_204_H_150_I_24_N_30_O_12_Zn_12_, Mr = 7043.57, trigonal, *R*-3 (No. 148), *a* = 38.6805(7) Å, *b* = 38.6805(7) Å, *c* = 16.0202(3) Å, *α* = 90°, *β* = 90°, *γ* = 120°, V = 20757.9(8) Å^3^, T = 303(2) K, *Z* = 3. Table S1 contains further crystallographic information.

The reference CCDC code for **1** is 2233049.

Crystal Data (**2**). C_54_H_40_Cl_2_I_6_N_6_Zn_3_, Mr = 1801, monoclinic, *C*2/*c* (No. 15), *a* = 24.1055(4) Å, *b* = 14.4843(2) Å, *c* = 18.2201(2) Å, *α* = 90°, *β* = 100.8630(10)°, *γ* = 90°, V = 6247.57(15) Å^3^, T = 303(2) K, *Z* = 4. Table S2 contains further crystallographic information.

The reference CCDC code for **2** is 2233048.

**Powder X-Ray diffraction experiments.**

All the powder X-ray diffraction experiments were carried out using a Bruker D2-Phaser diffractometer equipped with Cu radiation (*λ* = 1.54184 Å) using Bragg-Brentano geometry. The experiments were performed at room temperature.

**TG experiments.**

Thermogravimetric analysis was carried out using a Perkin Elmer Thermal Analysis instrument at the Laboratorio Analisi Chimiche at the Dipartimento di Chimica, Materiali ed Ingegneria Chimica, Politecnico di Milano. The analyzed microcrystalline samples were heated within the temperature range from 30 °C to 700 °C using a heating rate of 10 °C/min under N_2_.

**Density Functional Theory**

Molecular modelling studies are performed in the gas phase. The calculations rely on the gradient corrected GGA PBE functional.^[[3]](#endnote-3)^,^[[4]](#endnote-4)^ A numerical double zeta numerical basis set centered on atoms (including polarisation functions on all atoms), roughly comparable with the usual 6-31G** gaussian basis, has been employed. Explicit van der Waals corrections^[[5]](#endnote-5)^ were also used to improve the description of van der Waals intra-particle interactions.^[[6]](#endnote-6)^,^[[7]](#endnote-7)^ The DMol^3^ package^[[8]](#endnote-8)^ was employed for all the calculations.

The geometries of the molecules have been extracted from experimental single crystal X-ray determined structures and optimised. Electrostatic Potentials have been plotted by employing the above mentioned DFT electron density.

**Slow crystallization experiments using TPB and ZnI_2_ in various aromatic solvents: nitrobenzene, toluene, chlorobenzene.**

**Crystallization using nitrobenzene:** In a typical layer diffusion experiment 7.5 mg of TPB were dissolved in a mixture of nitrobenzene/methanol (3:2 ml) forming the bottom layer in the crystallization tube. Then 3 ml of methanol as a middle layer were added. The last layer, the methanolic solution of ZnI_2_ (11.5 mg ZnI_2_ / 1 ml methanol), was added on top dropwise. The crystallization tube was left to stand for 5 days in the lab.

**
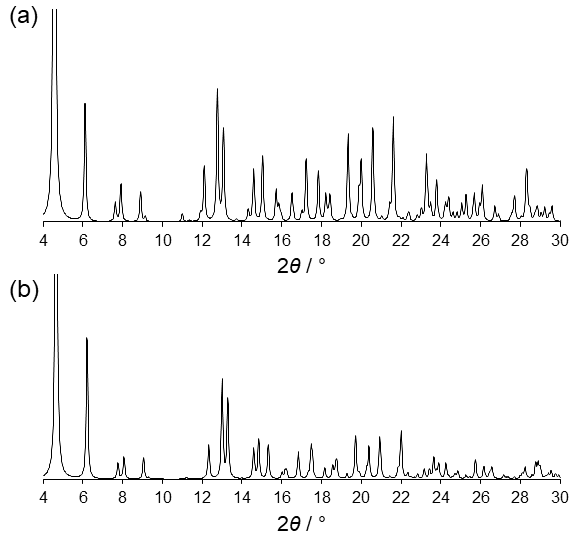
**

**Figure S1. (a)** Simulated powder XRD pattern of TPB-ZnI_2_ synthesized using slow crystallization (layering) method with nitrobenzene as templating solvent. The data was recorded at room temperature. The structure is the poly-[*n*]-catenane **1**. (b) Simulated powder XRD pattern of the TPB-ZnBr_2_ **M_12_L_8_** poly-[*n*]-catenane including *para*-chlorotoluene as guest whose SC-XRD structure was measured at 100 K.^[[9]](#endnote-9)^

**Crystallization using chlorobenzene:** In a typical layer diffusion experiment 7.5 mg of TPB were dissolved in a mixture of chlorobenzene/methanol (3:2 ml) forming the bottom layer in the crystallization tube. Then 3 ml of methanol as a middle layer were added. The last layer, the methanolic solution of ZnI_2_, was added on top dropwise. The crystallization tube was left to stand for 5 days in the lab.

**
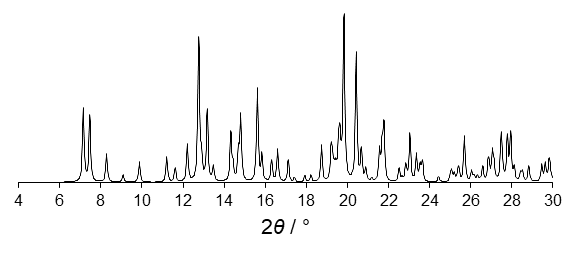
**

**Figure S2.** Simulated powder XRD pattern of TPB-ZnI_2_ synthesized using slow crystallization (layering) method using chlorobenzene as templating agent. The data was recorded at room temperature.


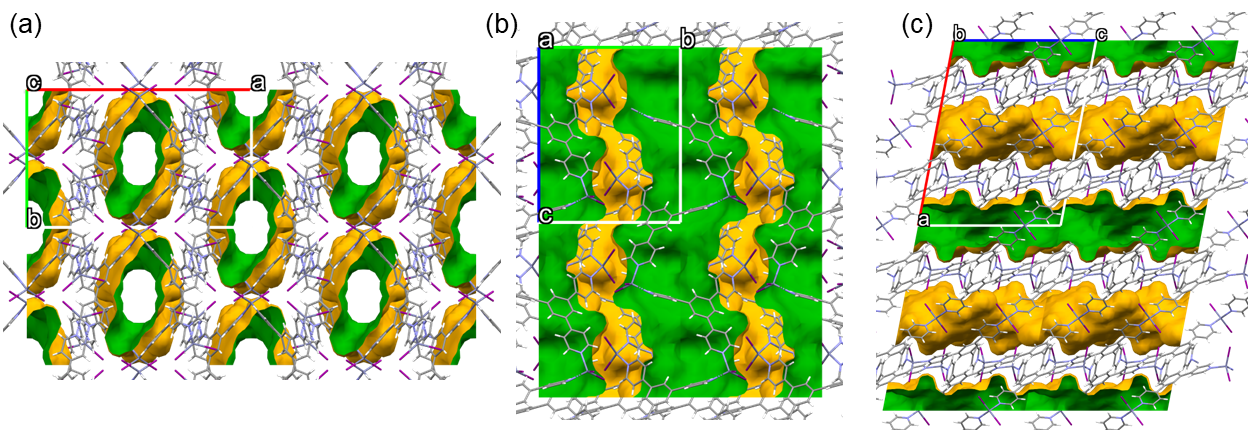


**Figure S3.** Picture showing the 1D channels viewed along the three crystallographic directions of **2**. The void space corresponds to 30 % of the total unit cell volume.

**Crystallization using toluene:** In a typical layer diffusion experiment 7.5 mg of TPB were dissolved in a mixture of toluene/methanol (7:3 ml) forming the bottom layer in the crystallization tube. Then 3 ml of methanol as a middle layer were added. The last layer, the methanolic solution of ZnI_2_, was added on top dropwise. The crystallization tube was left to stand for 5 days in the lab.


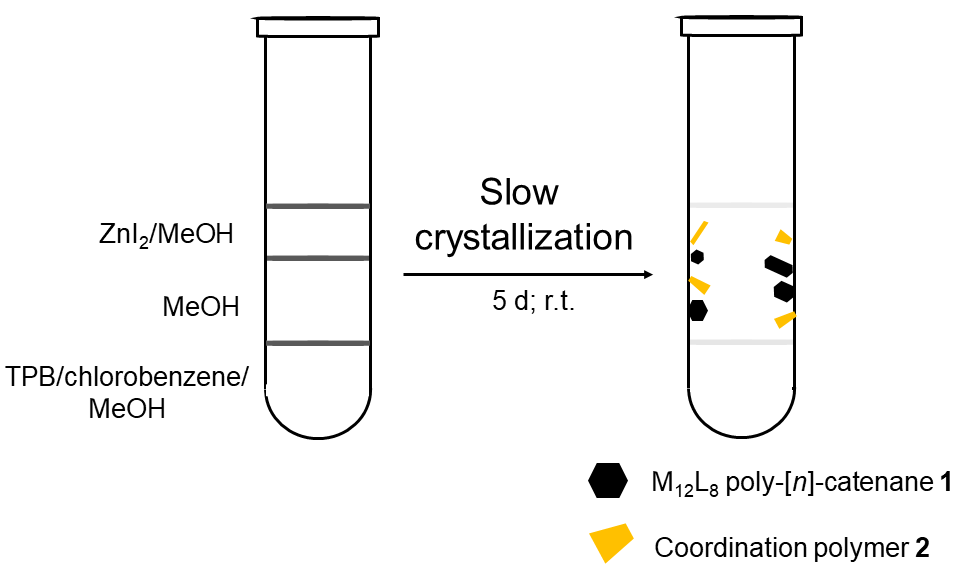


**Figure S4.** Cartoon showing the slow crystallization carried out by triple layering with a bottom TPB solution including the templating solvent chlorobenzene, the middle layer of MeOH separating the metal that is added as a top layer. All the three layers are deposited dropwise to avoid rapid mixing of the self-assembling components. The same methodology was used using nitrobenzene and *o*-dichlorobenzene.

**Instant synthesis using TPB and ZnI_2_ in various aromatic solvents: nitrobenzene, toluene, chlorobenzene.**

Instant precipitation of the **M_12_L_8_** poly-[*n*]-catenane was carried out using the following aromatic solvents: nitrobenzene, toluene and chlorobenzene.

**Fast crystallization using nitrobenzene as aromatic solvent:** The fast crystallization using nitrobenzene as aromatic solvent was carried out by dissolving TPB (30 mg) in a mixture nitrobenzene/methanol (6/3 mL) which was kept stirring vigorously at room temperature. Then the ZnI_2_ (48 mg) dissolved in methanol was added to the stirring TPB solution. Immediately a yellowish precipitate was formed.

**
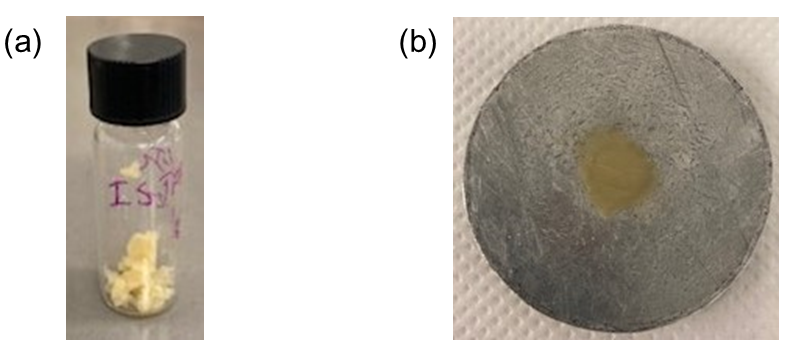
**

**Figure S5.** (a) Vial containing the “sticky” product after filtration the suspension generated upon instant synthesis. (b) Actual view of the sample mounted and measured by powder XRD.

**
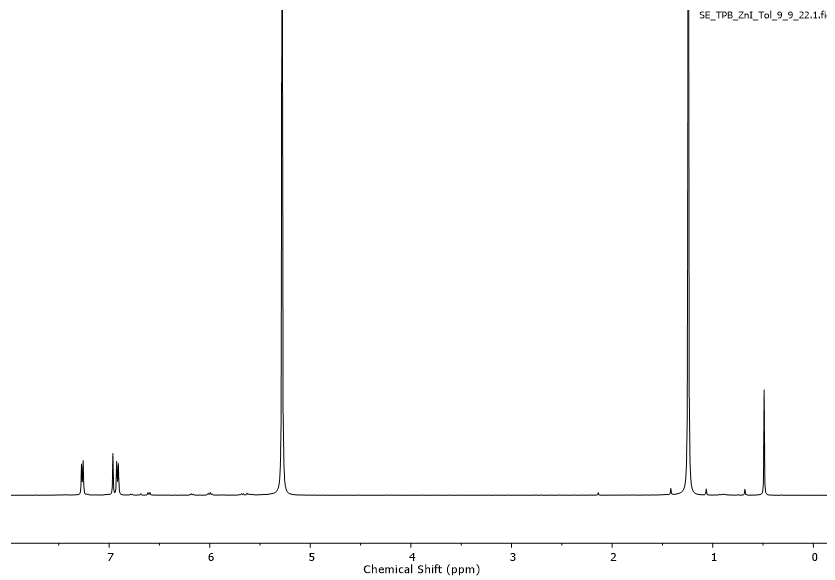
**

**Figure S6.** ^1^H NMR of the sample **a1** obtained by instant synthesis after being immersed in toluene/methanol. The ^1^H NMR plot shows that no nitrobenzene nor methanol are present but toluene.

**Fast crystallization using toluene as aromatic solvent:** The fast crystallization using toluene as aromatic solvent was carried out by dissolving TPB (30 mg) in a mixture toluene/methanol (24/10 mL) which was kept stirring vigorously at room temperature. Then the ZnI_2_ (48 mg) dissolved in methanol was added to the stirring TPB solution. Immediately a precipitate was formed. Upon filtration the solid obtained was 20 mg. The powder XRD pattern was measured right after the synthesis (Figure S11a) and one month later (Figure S11b). Clearly, the sample is not able to maintain its crystallinity.

**
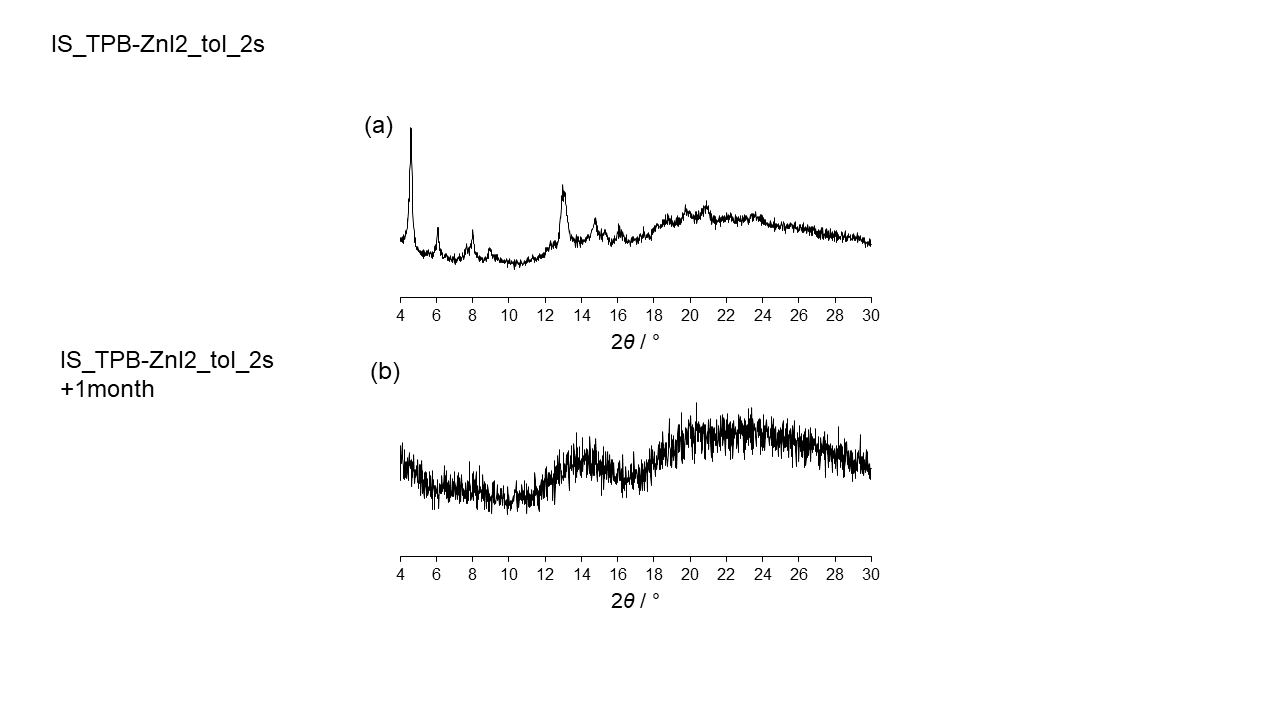
**

**Figure S7.** (a) Experimental powder XRD pattern of the solid product obtained from the instant synthesis of TPB and ZnI_2_ using toluene as templating aromatic solvent measured after the synthesis (*i.e.*, within 1 h after filtration). (b) The same sample measured after one month kept in the vial.

**
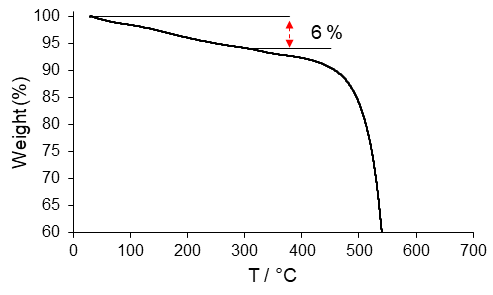
**

**Figure S8.** TG corresponding to the sample obtained upon instant synthesis using toluene as templating agent which was left stored in the vial for one month. The reaction was carried out at room temperature.

**Fast crystallization using chlorobenzene as aromatic solvent:** The fast crystallization using chlorobenzene as aromatic solvent was carried out by dissolving TPB (30 mg) in a mixture chlorobenzene/methanol (10/7 mL) which was kept stirring vigorously at room temperature. Then the ZnI_2_ (48 mg) dissolved in methanol was added to the stirring TPB solution. Immediately a yellowish precipitate was formed. Upon filtration the solid obtained was 52 mg. (a) The powder XRD diffraction pattern measured after the synthesis and filtration of the solid shows the crystalline poly-[*n*]-catenane (Figure S13a). The same sample measured after one month in the closed vial shows that the material retains its crystallinity (Figure S13b).

**
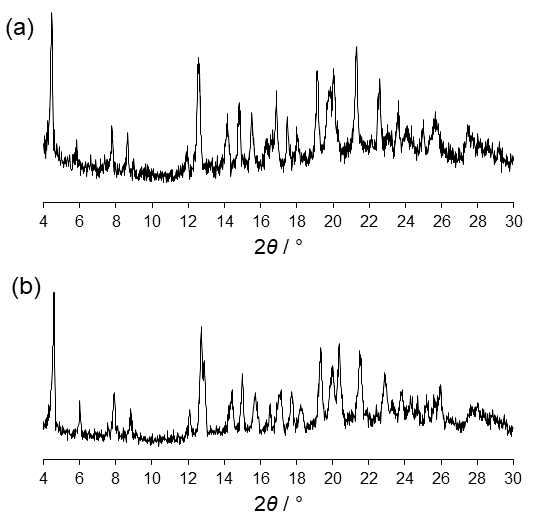
**

**Figure S9.** (a) Experimental powder XRD pattern of the solid product obtained from the instant synthesis of TPB and ZnI_2_ using chlorobenzene as templating aromatic solvent measured after the synthesis (*i.e.*, within 1 h after filtration). (b) The same sample measured after one month kept in the vial at room temperature.

**
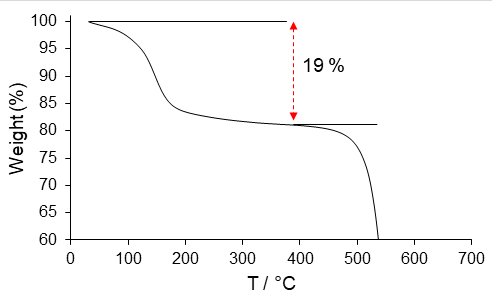
**

**Figure S10.** TG corresponding to the sample obtained upon instant synthesis using chlorobenzene as templating agent measured after one month of its synthesis. The sample used is the same used to do the powder XRD shown in Figure S13b.

**Solid-state synthesis of amorphous poly-[*n*]-catenane (a1').**

**
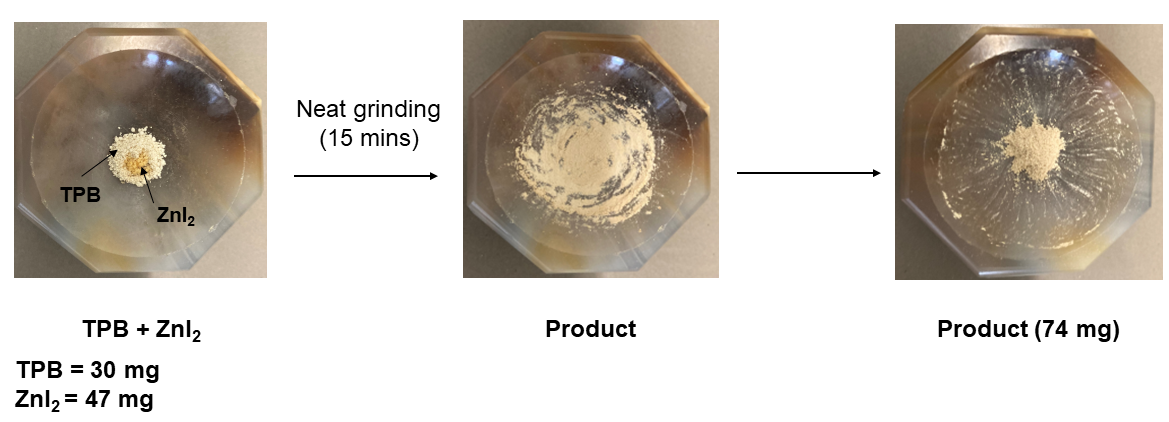
**

**Figure S11.** Images of the mortar taken during the neat grinding experiment. The actual product after removing it from the mortar and transferred to a vial was 74 mg.


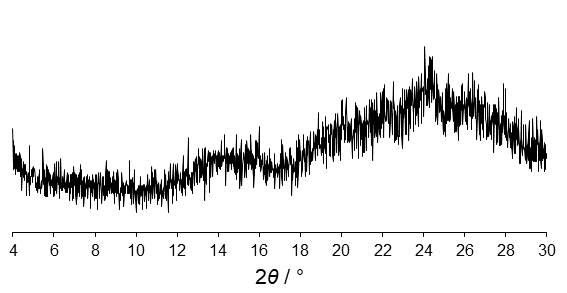


**Figure S12.** Experimental powder XRD pattern of the amorphous phase (**a1'**) obtained upon neat grinding **TPB** and ZnI_2_.


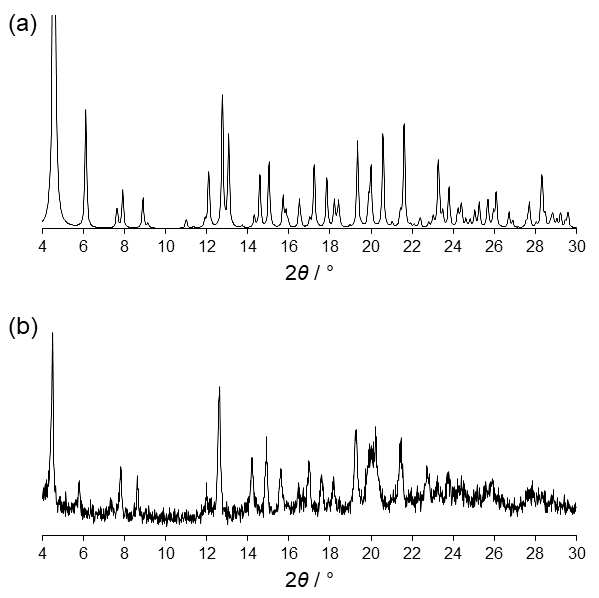


**Figure S13.** (a) Simulated powder XRD pattern of **1** at room temperature. (b) Experimental powder XRD pattern obtained after immersing the amorphous phase **a1'** obtained upon neat grinding in toluene/methanol overnight measured at room temperature.

**Gas-solid phase *amorphous-to-crystalline* transformation using a1'**.

The proof that the neat grinding of **TPB** and ZnI_2_ produces amorphous poly-[*n*]-catenane and that uptakes guest molecules not following a recrystallization process is demonstrated by the following experiment: a gas-solid reaction in which **a1’** is exposed to vapours of *p*-xylene and methanol (5ml : 5ml) for 20 h. After the gas-solid reaction, the sample was analyzed by powder XRD. The diffractograms in Figure S14 clearly shows the transformation towards a crystalline phase of poly-[*n*]-catenane. In particular Figure S14b shows how the poly-[*n*]-catenane is forming. A similar guest inclusion via gas-solid reaction is reported for the TPB-ZnBr_2_ exposed to methanol and dichlorobenzene.^[[10]](#endnote-10)^

**
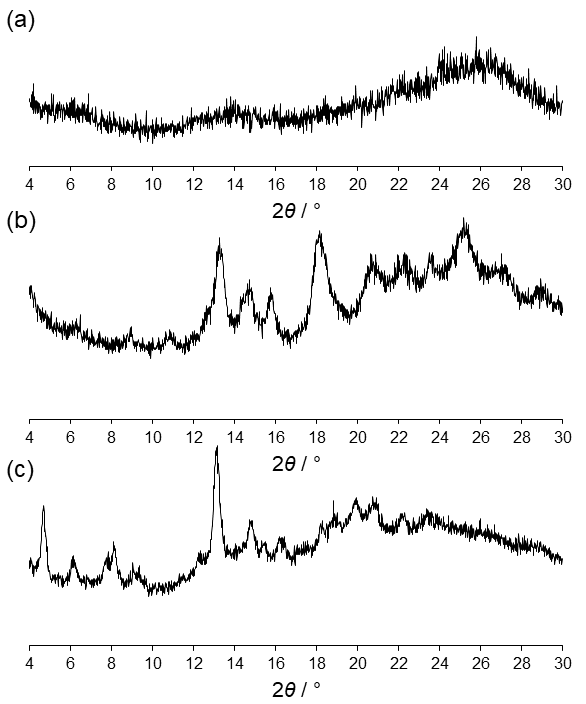
Figure S14.** Powder XRD pattern of **a1'** obtained by neat grinding (a). Powder diffractogram of **a1'** obtained after exposing it in a sealed chamber with 5 ml of *p*-xylene and 5 ml of methanol for 20 h (b). Powder XRD of **a1'** immersed in a mixture *p*-xylene/methanol (8 ml / 2 ml) at room temperature (c).

***Amorphous*-to-*crystalline* transformation by uptake of xylenes.**

**Uptake of *o*-xylene by a1' in a solid-liquid heterogeneous reaction.** In a round bottom flask containing 4ml of *o*-xylene and 1ml of methanol were introduced 30 mg of **a1'** obtained via neat grinding. The suspension was left stirring overnight. Then the solid was filtered and mounted for powder XRD analysis. The obtained diffractogram shown in Figure S15 demonstrates that the sample is crystalline.

**
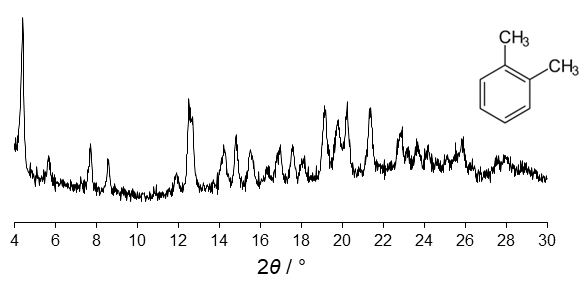
**

**Figure S15.** Powder XRD pattern obtained after immersing the amorphous phase **a1'** in *o*-xylene/methanol measured at room temperature.

**Uptake of *m*-xylene by a1' in a solid-liquid heterogeneous reaction.** In a round bottom flask containing 4ml of *m*-xylene and 1ml of methanol were introduced 30 mg of **a1'** obtained via neat grinding. The suspension was left stirring overnight. Then the solid was filtered and subjected to powder XRD analysis (Figure S16). The obtained diffractogram demonstrates that the sample is crystalline and the structural transformation can be considered as an *amorphous*-to-*crystalline* process.

**
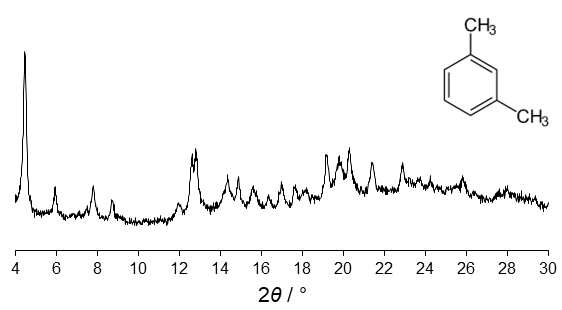
**

**Figure S16.**  Powder XRD pattern obtained after immersing the amorphous phase **a1’** in *m*-xylene/methanol measured at room temperature.

**Uptake of *p*-xylene by a1’ in a solid-liquid heterogeneous reaction.** In a round bottom flask containing 4ml of *p*-xylene and 1ml of methanol were introduced 30 mg of amorphous phase **a1’** obtained via neat grinding. The suspension was left stirring overnight. Then the solid was filtered and subjected to powder XRD analysis (Figure S17). The obtained diffractogram demonstrates that the sample is crystalline and it is very similar to the diffractogram obtained after exposing **a1'** to *m*-xylene and *o*-xylene.


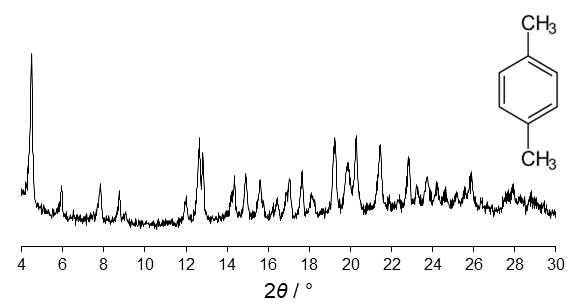


**Figure S17.** Powder XRD pattern obtained after immersing the amorphous phase **a1'** in *p*-xylene/methanol measured at room temperature.

**
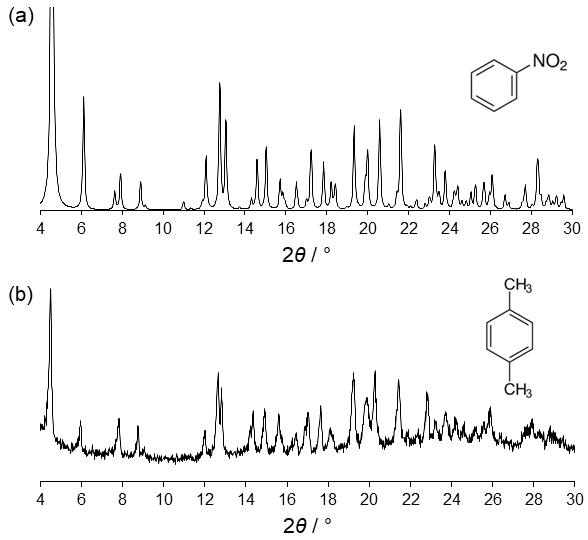
**

**Figure S18.** (a) Simulated powder XRD pattern of **1** from single crystal XRD data at room temperature. (b) Experimental powder XRD data obtained after immersing the amorphous phase **a1'** in *para*-xylene/methanol also recorded at room temperature.

**Solid-state synthesis of amorphous M_12_L_8_ poly-[*n*]-catenane using TPB and ZnCl_2_.**

Here we also report the solid-state synthesis of the **M_12_L_8_** poly-[*n*]-catenane in the amorphous state upon grinding **TPB** and ZnCl_2_. The powder XRD pattern has the profile typical of the amorphous poly-[*n*]-catenanes of **M_12_L_8_** interlocked cages with two broad bumps as in the bromide and iodide amorphous solids. The formation of the poly-[*n*]-catenane is corroborated by the guest uptake (1,2-dichlorobenzene) which induces the transformation from amorphous to crystalline via an a*morphous-to-crystalline* transformation. The powder XRD pattern after guest uptake matches with those of the TPB-ZnCl_2_ poly-[*n*]-catenane including similar aromatic molecules. See ESI for further information.

**
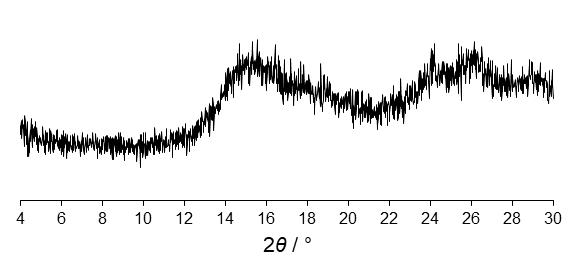
**

**Figure S19.** Powder XRD pattern obtained after grinding **TPB** and ZnCl_2_. The product clearly shows that is amorphous.


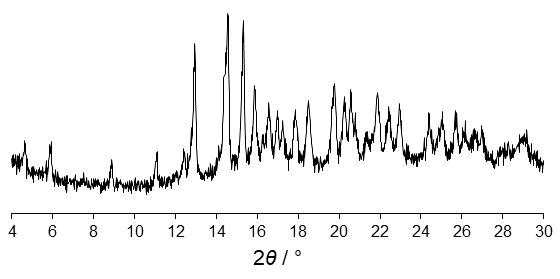


**Figure S20.** Powder XRD pattern obtained after immersing in *o*-DCB/methanol the amorphous phase obtained from grinding **TPB** and ZnCl_2_. The product clearly shows that became crystalline following an *amorphous*-*to*-*crystalline* transformation.

**Table S1**. Crystal data and structure refinement for the poly-[*n*]-catenane **1**.

Identification code **1**

Empirical formula C_204_ H_150_ I_24_ N_30_ O_12_ Zn_12_

Formula weight 7043.57

Temperature 303(2) K

Wavelength 1.54184 Å

Crystal system Trigonal

Space group *R* -3 :H

Unit cell dimensions *a* = 38.6805(7) Å *α* = 90°.

*b* = 38.6805(7) Å *β* = 90°.

*c* = 16.0202(3) Å *γ* = 120°.

Volume 20757.9(8) Å3

Z 3

Density (calculated) 1.690 Mg/m3

Absorption coefficient 22.539 mm-1

F(000) 9936

Crystal size 0.100 x 0.080 x 0.050 mm3

Theta range for data collection 3.058 to 76.440°.

Index ranges -42<=h<=36, -46<=k<=48, -19<=l<=16

Reflections collected 42589

Independent reflections 9032 [R(int) = 0.0780]

Completeness to theta = 67.684° 99.6 %

Absorption correction "cylinder"

Max. and min. transmission 0.1295 and 0.0323

Refinement method Full-matrix least-squares on F2

Data / restraints / parameters 9032 / 92 / 386

Goodness-of-fit on F2 1.052

Final R indices [I>2sigma(I)] R1 = 0.0753, wR2 = 0.2362

R indices (all data) R1 = 0.0887, wR2 = 0.2561

Extinction coefficient n/a

Largest diff. peak and hole 2.457 and -0.990 e.Å-3

**Table S2**. Crystal data and structure refinement for the coordination polymer **2**.

Identification code **2**

Empirical formula C_54_ H_40_ Cl_2_ I_6_ N_6_ Zn_3_

Formula weight 1801.33

Temperature 304(2) K

Wavelength 1.54184 Å

Crystal system Monoclinic

Space group *C*2/*c*

Unit cell dimensions *a* = 24.1055(4) Å *α* = 90°.

*b* = 14.4843(2) Å *β* = 100.8630(10)°.

*c* = 18.2201(2) Å *γ* = 90°.

Volume 6247.57(15) Å3

Z 4

Density (calculated) 1.915 Mg/m3

Absorption coefficient 25.704 mm-1

F(000) 3392

Crystal size 0.100 x 0.070 x 0.060 mm3

Theta range for data collection 3.577 to 76.599°.

Index ranges -30<=h<=30, -17<=k<=17, -22<=l<=14

Reflections collected 29701

Independent reflections 6171 [R(int) = 0.0526]

Completeness to theta = 67.684° 99.4 %

Absorption correction "multi-scan"

Max. and min. transmission 1.0 and 0.19306

Refinement method Full-matrix least-squares on F2

Data / restraints / parameters 6171 / 13 / 286

Goodness-of-fit on F2 1.105

Final R indices [I>2sigma(I)] R1 = 0.0797, wR2 = 0.2443

R indices (all data) R1 = 0.0900, wR2 = 0.2560

Extinction coefficient n/a

Largest diff. peak and hole 1.908 and -1.688 e.Å-3

**References**

1. Sheldrick, G. M., Crystal structure refinement with SHELXL. *Acta Cryst*. **C71***,* 3–8 (2015). [↑](#endnote-ref-1)
2. Dolomanov, O. V.; Bourhis, L. J.; Gildea, R. J.; Howard, J. A. K.; Puschmann, H. Olex2: A complete structure solution, refinement and analysis program. *J. Appl. Cryst*. **42**, 339-341 (2009). [↑](#endnote-ref-2)
3. Perdew, J. P.; Burke, K. Ernzerhof, M. E. Generalized Gradient Approximation Made Simple. *Phys. Rev. Lett.* ***77***, 3865–3868 (1996). [↑](#endnote-ref-3)
4. Perdew, J. P.; Burke, K.; Ernzerhof, M. E. Generalized Gradient Approximation Made Simple. *Phys. Rev. Lett.* ***78***, 1396–1396 (1997). [↑](#endnote-ref-4)
5. Grimme, S. Semiempirical hybrid density functional with perturbative second-order correlation. *J. Chem. Phys*.**124**, 34108 (2006). [↑](#endnote-ref-5)
6. Baggioli, A.; Meille, S. V.; Raos, G.; Po, R.; Brinkmann, M. Famulari, A. Intramolecular CH/π interactions in alkylaromatics: Monomer conformations for poly(3-alkylthiophene) atomistic models. *Int. J. Quantum Chem*. **113**, 2154-2162 (2013). [↑](#endnote-ref-6)
7. Baggioli, A.; Famulari, A. On the inter-ring torsion potential of regioregular P3HT: a first principles reexamination with explicit side chains. *Phys. Chem. Chem. Phys*. **16**, 3983-3994 (1014). [↑](#endnote-ref-7)
8. Delley, B. From molecules to solids with the DMol^3^ approach. *J. Chem. Phys.* **113**, 7756-7764 (2000). [↑](#endnote-ref-8)
9. Martí-Rujas, J.; Ma, S.; Famulari, A. Experimental X-ray and DFT structural analyses of M_12_L_8_ poly-[*n*]-catenanes using exotridentate ligands. *Inorg. Chem.* **61**, 10863-10871 (2022). [↑](#endnote-ref-9)
10. Martí-Rujas, J.; Elli, S.; Sacchetti, A.; F. Castiglione, *Dalton Trans*. **51**, 53-58 (2022). [↑](#endnote-ref-10)
