# Supplementary material for: Kinetic trapping of 2,4,6-tris(4-pyridyl)benzene and ZnI2 into M12L8 poly-[n]-catenanes using solution and solid-state processes
Source: Sci Rep. 2023 Apr 5;13:5605. doi: 10.1038/s41598-023-32661-x (PMC10076325; doi:10.1038/s41598-023-32661-x)
Supplement: Supplementary file 4 — Supplementary Information 4. [file 41598_2023_32661_MOESM4_ESM.docx]

data_shelx

_audit_creation_method 'SHELXL-2017/1'

_shelx_SHELXL_version_number '2017/1'

_chemical_name_systematic ?

_chemical_name_common ?

_chemical_melting_point ?

_chemical_formula_moiety ?

_chemical_formula_sum

'C54 H40 Cl2 I6 N6 Zn3'

_chemical_formula_weight 1801.33

loop_

_atom_type_symbol

_atom_type_description

_atom_type_scat_dispersion_real

_atom_type_scat_dispersion_imag

_atom_type_scat_source

'C' 'C' 0.0181 0.0091

'International Tables Vol C Tables 4.2.6.8 and 6.1.1.4'

'H' 'H' 0.0000 0.0000

'International Tables Vol C Tables 4.2.6.8 and 6.1.1.4'

'Cl' 'Cl' 0.3639 0.7018

'International Tables Vol C Tables 4.2.6.8 and 6.1.1.4'

'I' 'I' -0.3257 6.8362

'International Tables Vol C Tables 4.2.6.8 and 6.1.1.4'

'N' 'N' 0.0311 0.0180

'International Tables Vol C Tables 4.2.6.8 and 6.1.1.4'

'Zn' 'Zn' -1.5491 0.6778

'International Tables Vol C Tables 4.2.6.8 and 6.1.1.4'

_space_group_crystal_system monoclinic

_space_group_IT_number 15

_space_group_name_H-M_alt 'C 2/c'

_space_group_name_Hall '-C 2yc'

_shelx_space_group_comment

;

The symmetry employed for this shelxl refinement is uniquely defined

by the following loop, which should always be used as a source of

symmetry information in preference to the above space-group names.

They are only intended as comments.

;

loop_

_space_group_symop_operation_xyz

'x, y, z'

'-x, y, -z+1/2'

'x+1/2, y+1/2, z'

'-x+1/2, y+1/2, -z+1/2'

'-x, -y, -z'

'x, -y, z-1/2'

'-x+1/2, -y+1/2, -z'

'x+1/2, -y+1/2, z-1/2'

_cell_length_a 24.1055(4)

_cell_length_b 14.4843(2)

_cell_length_c 18.2201(2)

_cell_angle_alpha 90

_cell_angle_beta 100.8630(10)

_cell_angle_gamma 90

_cell_volume 6247.57(15)

_cell_formula_units_Z 4

_cell_measurement_temperature 304(2)

_cell_measurement_reflns_used 14211

_cell_measurement_theta_min 3.5560

_cell_measurement_theta_max 76.075

_exptl_crystal_description "plate"

_exptl_crystal_colour "colorless"

_exptl_crystal_density_meas ?

_exptl_crystal_density_method ?

_exptl_crystal_density_diffrn 1.915

_exptl_crystal_F_000 3392

_exptl_transmission_factor_min ?

_exptl_transmission_factor_max ?

_exptl_crystal_size_max 0.100

_exptl_crystal_size_mid 0.070

_exptl_crystal_size_min 0.060

_exptl_absorpt_coefficient_mu 25.704

_shelx_estimated_absorpt_T_min 0.183

_shelx_estimated_absorpt_T_max 0.308

_exptl_absorpt_correction_type "multi-scan"

_exptl_absorpt_correction_T_min 0.19306

_exptl_absorpt_correction_T_max 1.0

_exptl_absorpt_process_details "CrysAlisPro 1.171.42.55a (Rigaku Oxford Diffraction, 2022) Empirical absorption correction using spherical harmonics, implemented in SCALE3 ABSPACK scaling algorithm"

_exptl_absorpt_special_details ?

_diffrn_ambient_temperature 304(2)

_diffrn_radiation_wavelength 1.54184

_diffrn_radiation_type CuK\a

_diffrn_source 'micro-focus sealed X-ray tube'

_diffrn_measurement_device_type 'PhotonJet (Cu) X-ray Source'

_diffrn_measurement_method ?

_diffrn_detector_area_resol_mean ?

_diffrn_reflns_number 29701

_diffrn_reflns_av_unetI/netI 0.0348

_diffrn_reflns_av_R_equivalents 0.0526

_diffrn_reflns_limit_h_min -30

_diffrn_reflns_limit_h_max 30

_diffrn_reflns_limit_k_min -17

_diffrn_reflns_limit_k_max 17

_diffrn_reflns_limit_l_min -22

_diffrn_reflns_limit_l_max 14

_diffrn_reflns_theta_min 3.577

_diffrn_reflns_theta_max 76.599

_diffrn_reflns_theta_full 67.684

_diffrn_measured_fraction_theta_max 0.937

_diffrn_measured_fraction_theta_full 0.994

_diffrn_reflns_Laue_measured_fraction_max 0.937

_diffrn_reflns_Laue_measured_fraction_full 0.994

_diffrn_reflns_point_group_measured_fraction_max 0.937

_diffrn_reflns_point_group_measured_fraction_full 0.994

_reflns_number_total 6171

_reflns_number_gt 4954

_reflns_threshold_expression 'I > 2\s(I)'

_reflns_Friedel_coverage 0.000

_reflns_Friedel_fraction_max .

_reflns_Friedel_fraction_full .

_reflns_special_details

;

Reflections were merged by SHELXL according to the crystal

class for the calculation of statistics and refinement.

_reflns_Friedel_fraction is defined as the number of unique

Friedel pairs measured divided by the number that would be

possible theoretically, ignoring centric projections and

systematic absences.

;

_computing_data_collection ?

_computing_cell_refinement ?

_computing_data_reduction ?

_computing_structure_solution ?

_computing_structure_refinement 'SHELXL-2017/1 (Sheldrick, 2017)'

_computing_molecular_graphics ?

_computing_publication_material ?

_refine_special_details ?

_refine_ls_structure_factor_coef Fsqd

_refine_ls_matrix_type full

_refine_ls_weighting_scheme calc

_refine_ls_weighting_details

'w=1/[\s^2^(Fo^2^)+(0.1480P)^2^+30.4862P] where P=(Fo^2^+2Fc^2^)/3'

_atom_sites_solution_primary ?

_atom_sites_solution_secondary ?

_atom_sites_solution_hydrogens geom

_refine_ls_hydrogen_treatment constr

_refine_ls_extinction_method none

_refine_ls_extinction_coef .

_refine_ls_number_reflns 6171

_refine_ls_number_parameters 286

_refine_ls_number_restraints 13

_refine_ls_R_factor_all 0.0900

_refine_ls_R_factor_gt 0.0797

_refine_ls_wR_factor_ref 0.2560

_refine_ls_wR_factor_gt 0.2443

_refine_ls_goodness_of_fit_ref 1.105

_refine_ls_restrained_S_all 1.104

_refine_ls_shift/su_max 0.065

_refine_ls_shift/su_mean 0.003

loop_

_atom_site_label

_atom_site_type_symbol

_atom_site_fract_x

_atom_site_fract_y

_atom_site_fract_z

_atom_site_U_iso_or_equiv

_atom_site_adp_type

_atom_site_occupancy

_atom_site_site_symmetry_order

_atom_site_calc_flag

_atom_site_refinement_flags_posn

_atom_site_refinement_flags_adp

_atom_site_refinement_flags_occupancy

_atom_site_disorder_assembly

_atom_site_disorder_group

I1 I 0.84187(3) 0.11266(6) 0.14685(4) 0.0968(3) Uani 1 1 d . . . . .

I2 I 0.66219(4) 0.04094(5) 0.10376(5) 0.0978(3) Uani 1 1 d . . . . .

I3 I 0.42455(7) -0.03287(14) 0.65663(8) 0.1843(8) Uani 1 1 d . . . . .

Zn4 Zn 0.74051(6) 0.15592(8) 0.14879(6) 0.0697(4) Uani 1 1 d . . . . .

Zn5 Zn 0.500000 0.04915(13) 0.750000 0.0793(5) Uani 1 2 d S T P . .

N18 N 0.5394(4) 0.1328(6) 0.6852(4) 0.0741(19) Uani 1 1 d . . . . .

N24 N 0.7217(4) 0.7171(5) 0.5983(4) 0.0681(18) Uani 1 1 d . . . . .

N12 N 0.7316(4) 0.2010(5) 0.2543(4) 0.0721(19) Uani 1 1 d . . . . .

C7 C 0.7016(4) 0.3989(6) 0.4779(4) 0.0612(18) Uani 1 1 d . . . . .

H7 H 0.724587 0.433335 0.452537 0.073 Uiso 1 1 calc R U . . .

C27 C 0.6969(4) 0.5345(6) 0.5597(4) 0.0624(19) Uani 1 1 d . . . . .

C21 C 0.6003(4) 0.2369(6) 0.6007(5) 0.067(2) Uani 1 1 d . . . . .

C8 C 0.6825(4) 0.4377(6) 0.5382(4) 0.0615(18) Uani 1 1 d . . . . .

C11 C 0.6532(4) 0.2586(6) 0.4944(4) 0.0628(19) Uani 1 1 d . . . . .

H11 H 0.642593 0.198845 0.478991 0.075 Uiso 1 1 calc R U . . .

C26 C 0.7500(4) 0.5695(6) 0.5615(5) 0.068(2) Uani 1 1 d . . . . .

H26 H 0.778552 0.531986 0.550267 0.081 Uiso 1 1 calc R U . . .

C9 C 0.6483(4) 0.3862(6) 0.5762(4) 0.0633(19) Uani 1 1 d . . . . .

H9 H 0.634237 0.412804 0.615371 0.076 Uiso 1 1 calc R U . . .

C10 C 0.6350(4) 0.2950(6) 0.5561(4) 0.0613(18) Uani 1 1 d . . . . .

C22 C 0.5648(5) 0.1708(8) 0.5680(5) 0.084(3) Uani 1 1 d . . . . .

H22 H 0.560682 0.160098 0.516963 0.101 Uiso 1 1 calc R U . . .

C28 C 0.6552(4) 0.5940(7) 0.5759(6) 0.081(3) Uani 1 1 d . . . . .

H28 H 0.618519 0.573050 0.574562 0.097 Uiso 1 1 calc R U . . .

C6 C 0.6869(4) 0.3093(6) 0.4550(4) 0.0610(18) Uani 1 1 d . . . . .

C25 C 0.7607(4) 0.6609(6) 0.5802(5) 0.069(2) Uani 1 1 d . . . . .

H25 H 0.796606 0.684153 0.580220 0.083 Uiso 1 1 calc R U . . .

C15 C 0.7054(4) 0.2696(6) 0.3860(4) 0.0613(18) Uani 1 1 d . . . . .

C29 C 0.6698(5) 0.6860(8) 0.5943(6) 0.084(3) Uani 1 1 d . . . . .

H29 H 0.642036 0.726306 0.603947 0.101 Uiso 1 1 calc R U . . .

C23 C 0.5347(5) 0.1194(8) 0.6101(6) 0.084(3) Uani 1 1 d . . . . .

H23 H 0.510353 0.074017 0.586724 0.101 Uiso 1 1 calc R U . . .

C19 C 0.5742(5) 0.1979(9) 0.7153(5) 0.092(3) Uani 1 1 d . . . . .

H19 H 0.577945 0.208442 0.766313 0.110 Uiso 1 1 calc R U . . .

C13 C 0.7100(6) 0.1508(7) 0.2994(6) 0.095(4) Uani 1 1 d . . . . .

H13 H 0.702264 0.089374 0.286665 0.114 Uiso 1 1 calc R U . . .

C16 C 0.7323(6) 0.3211(8) 0.3422(6) 0.101(4) Uani 1 1 d . . . . .

H16 H 0.742979 0.381236 0.356054 0.121 Uiso 1 1 calc R U . . .

C20 C 0.6056(5) 0.2518(8) 0.6757(5) 0.086(3) Uani 1 1 d . . . . .

H20 H 0.629739 0.297226 0.699594 0.104 Uiso 1 1 calc R U . . .

C17 C 0.7442(6) 0.2853(8) 0.2769(6) 0.098(4) Uani 1 1 d . . . . .

H17 H 0.762254 0.323023 0.247415 0.117 Uiso 1 1 calc R U . . .

C14 C 0.6975(6) 0.1826(8) 0.3665(6) 0.093(3) Uani 1 1 d . . . . .

H14 H 0.683397 0.141761 0.397801 0.111 Uiso 1 1 calc R U . . .

Cl30 Cl 0.4908(10) 0.4438(14) 0.5996(12) 0.395(10) Uiso 1 1 d D . . . .

C3 C 0.5536(10) 0.5488(18) 0.7058(14) 0.184(9) Uiso 1 1 d D . . . .

H3 H 0.567593 0.496608 0.732836 0.220 Uiso 1 1 calc R U . . .

C2 C 0.4945(14) 0.6259(18) 0.5994(19) 0.229(14) Uiso 1 1 d D . . . .

H2 H 0.468714 0.621356 0.554580 0.275 Uiso 1 1 calc R U . . .

C5 C 0.5143(14) 0.713(2) 0.626(2) 0.236(14) Uiso 1 1 d D . . . .

H5 H 0.501802 0.765960 0.598238 0.283 Uiso 1 1 calc R U . . .

C12 C 0.5704(14) 0.637(2) 0.726(2) 0.222(13) Uiso 1 1 d D . . . .

H12 H 0.598077 0.641167 0.769182 0.267 Uiso 1 1 calc R U . . .

C18 C 0.5527(16) 0.722(3) 0.693(2) 0.30(2) Uiso 1 1 d D . . . .

H18 H 0.565317 0.778107 0.713660 0.357 Uiso 1 1 calc R U . . .

C4 C 0.5127(13) 0.546(2) 0.6393(16) 0.29(2) Uiso 1 1 d D . . . .

loop_

_atom_site_aniso_label

_atom_site_aniso_U_11

_atom_site_aniso_U_22

_atom_site_aniso_U_33

_atom_site_aniso_U_23

_atom_site_aniso_U_13

_atom_site_aniso_U_12

I1 0.1050(5) 0.1039(6) 0.0796(5) -0.0293(4) 0.0123(4) 0.0155(4)

I2 0.1197(6) 0.0827(5) 0.1028(6) -0.0223(4) 0.0514(5) -0.0171(4)

I3 0.1737(12) 0.2508(19) 0.1433(10) -0.0732(11) 0.0676(9) -0.1168(13)

Zn4 0.1156(9) 0.0540(6) 0.0481(5) -0.0032(4) 0.0373(6) 0.0052(6)

Zn5 0.0904(12) 0.0805(11) 0.0786(11) 0.000 0.0452(9) 0.000

N18 0.084(5) 0.084(5) 0.063(4) -0.006(4) 0.035(4) -0.015(4)

N24 0.102(5) 0.056(4) 0.053(3) -0.008(3) 0.032(3) -0.002(4)

N12 0.119(6) 0.060(4) 0.046(3) 0.001(3) 0.038(4) 0.003(4)

C7 0.082(5) 0.060(4) 0.049(4) -0.007(3) 0.030(4) -0.005(4)

C27 0.087(5) 0.062(5) 0.045(4) -0.003(3) 0.028(4) 0.001(4)

C21 0.088(5) 0.067(5) 0.054(4) -0.013(4) 0.033(4) -0.001(4)

C8 0.087(5) 0.060(4) 0.044(3) -0.008(3) 0.029(3) -0.001(4)

C11 0.085(5) 0.065(5) 0.044(3) -0.009(3) 0.024(3) -0.008(4)

C26 0.093(6) 0.062(5) 0.057(4) -0.009(4) 0.037(4) 0.002(4)

C9 0.084(5) 0.068(5) 0.045(4) -0.011(3) 0.031(4) -0.009(4)

C10 0.072(5) 0.067(5) 0.050(4) -0.004(3) 0.025(3) -0.003(4)

C22 0.100(7) 0.104(8) 0.054(4) -0.010(5) 0.031(5) -0.015(6)

C28 0.080(6) 0.075(6) 0.096(7) -0.024(5) 0.040(5) -0.007(5)

C6 0.085(5) 0.064(4) 0.041(3) -0.002(3) 0.026(3) 0.001(4)

C25 0.091(6) 0.066(5) 0.060(4) -0.005(4) 0.036(4) -0.011(4)

C15 0.086(5) 0.056(4) 0.046(3) -0.004(3) 0.021(3) -0.005(4)

C29 0.091(7) 0.074(6) 0.099(7) -0.023(5) 0.046(6) -0.003(5)

C23 0.107(7) 0.082(6) 0.070(5) -0.015(5) 0.035(5) -0.030(6)

C19 0.125(9) 0.107(8) 0.053(5) -0.020(5) 0.043(5) -0.031(7)

C13 0.174(12) 0.061(5) 0.064(5) -0.014(4) 0.057(7) -0.014(6)

C16 0.160(11) 0.089(7) 0.073(6) -0.034(5) 0.075(7) -0.039(7)

C20 0.108(7) 0.100(8) 0.057(5) -0.014(5) 0.033(5) -0.031(6)

C17 0.147(10) 0.085(7) 0.078(6) -0.030(5) 0.064(7) -0.033(7)

C14 0.155(10) 0.078(6) 0.061(5) -0.002(5) 0.059(6) -0.005(6)

_geom_special_details

;

All esds (except the esd in the dihedral angle between two l.s. planes)

are estimated using the full covariance matrix. The cell esds are taken

into account individually in the estimation of esds in distances, angles

and torsion angles; correlations between esds in cell parameters are only

used when they are defined by crystal symmetry. An approximate (isotropic)

treatment of cell esds is used for estimating esds involving l.s. planes.

;

loop_

_geom_bond_atom_site_label_1

_geom_bond_atom_site_label_2

_geom_bond_distance

_geom_bond_site_symmetry_2

_geom_bond_publ_flag

I1 Zn4 2.5291(16) . ?

I2 Zn4 2.5351(15) . ?

I3 Zn5 2.5392(15) . ?

Zn4 N24 2.068(7) 6_565 ?

Zn4 N12 2.080(6) . ?

Zn5 N18 2.046(7) 2_656 ?

Zn5 N18 2.046(7) . ?

N18 C19 1.312(14) . ?

N18 C23 1.366(13) . ?

N24 C25 1.330(12) . ?

N24 C29 1.319(13) . ?

N12 C13 1.278(12) . ?

N12 C17 1.306(13) . ?

C7 C6 1.389(12) . ?

C7 C8 1.388(10) . ?

C27 C26 1.372(13) . ?

C27 C28 1.398(13) . ?

C27 C8 1.479(11) . ?

C21 C22 1.346(14) . ?

C21 C20 1.366(12) . ?

C21 C10 1.525(11) . ?

C8 C9 1.389(11) . ?

C11 C10 1.385(10) . ?

C11 C6 1.392(11) . ?

C26 C25 1.378(13) . ?

C9 C10 1.392(12) . ?

C22 C23 1.370(14) . ?

C28 C29 1.403(14) . ?

C6 C15 1.522(10) . ?

C15 C14 1.314(14) . ?

C15 C16 1.346(13) . ?

C19 C20 1.381(14) . ?

C13 C14 1.390(12) . ?

C16 C17 1.376(12) . ?

Cl30 C4 1.686(18) . ?

C3 C12 1.369(18) . ?

C3 C4 1.411(14) . ?

C2 C4 1.396(18) . ?

C2 C5 1.407(19) . ?

C5 C18 1.392(19) . ?

C12 C18 1.399(19) . ?

loop_

_geom_angle_atom_site_label_1

_geom_angle_atom_site_label_2

_geom_angle_atom_site_label_3

_geom_angle

_geom_angle_site_symmetry_1

_geom_angle_site_symmetry_3

_geom_angle_publ_flag

N24 Zn4 N12 94.6(3) 6_565 . ?

N24 Zn4 I1 110.2(2) 6_565 . ?

N12 Zn4 I1 111.1(3) . . ?

N24 Zn4 I2 110.7(2) 6_565 . ?

N12 Zn4 I2 107.7(2) . . ?

I1 Zn4 I2 119.68(5) . . ?

N18 Zn5 N18 107.4(5) 2_656 . ?

N18 Zn5 I3 108.2(3) 2_656 . ?

N18 Zn5 I3 104.0(2) . . ?

N18 Zn5 I3 104.0(2) 2_656 2_656 ?

N18 Zn5 I3 108.2(3) . 2_656 ?

I3 Zn5 I3 124.21(13) . 2_656 ?

C19 N18 C23 116.5(8) . . ?

C19 N18 Zn5 121.0(6) . . ?

C23 N18 Zn5 122.3(7) . . ?

C25 N24 C29 118.9(8) . . ?

C25 N24 Zn4 123.3(7) . 6_566 ?

C29 N24 Zn4 117.0(6) . 6_566 ?

C13 N12 C17 115.3(8) . . ?

C13 N12 Zn4 122.8(6) . . ?

C17 N12 Zn4 121.7(6) . . ?

C6 C7 C8 120.8(7) . . ?

C26 C27 C28 118.2(8) . . ?

C26 C27 C8 121.9(8) . . ?

C28 C27 C8 119.8(8) . . ?

C22 C21 C20 119.1(9) . . ?

C22 C21 C10 121.6(7) . . ?

C20 C21 C10 119.3(8) . . ?

C9 C8 C7 119.6(7) . . ?

C9 C8 C27 120.6(6) . . ?

C7 C8 C27 119.8(7) . . ?

C10 C11 C6 121.5(8) . . ?

C27 C26 C25 119.4(8) . . ?

C8 C9 C10 120.5(7) . . ?

C11 C10 C9 118.9(7) . . ?

C11 C10 C21 120.7(7) . . ?

C9 C10 C21 120.4(6) . . ?

C21 C22 C23 119.9(9) . . ?

C29 C28 C27 118.5(9) . . ?

C7 C6 C11 118.6(7) . . ?

C7 C6 C15 120.2(7) . . ?

C11 C6 C15 121.1(7) . . ?

N24 C25 C26 122.8(9) . . ?

C14 C15 C16 115.5(8) . . ?

C14 C15 C6 122.5(8) . . ?

C16 C15 C6 122.0(8) . . ?

N24 C29 C28 122.1(9) . . ?

C22 C23 N18 122.1(9) . . ?

N18 C19 C20 124.0(8) . . ?

N12 C13 C14 123.9(9) . . ?

C15 C16 C17 120.5(10) . . ?

C21 C20 C19 118.4(10) . . ?

N12 C17 C16 123.4(10) . . ?

C15 C14 C13 120.8(9) . . ?

C12 C3 C4 113(3) . . ?

C4 C2 C5 121(4) . . ?

C18 C5 C2 120(4) . . ?

C3 C12 C18 130(4) . . ?

C5 C18 C12 114(4) . . ?

C2 C4 C3 122(3) . . ?

C2 C4 Cl30 118(2) . . ?

C3 C4 Cl30 120(2) . . ?

_refine_diff_density_max 1.908

_refine_diff_density_min -1.688

_refine_diff_density_rms 0.165

_shelx_res_file

;

TITL jm48_auto_a.res in C2/c

shelx.res

created by SHELXL-2017/1 at 17:53:21 on 12-Dec-2022

CELL 1.54184 24.1055 14.4843 18.2201 90.000 100.863 90.000

ZERR 4.00 0.0004 0.0002 0.0002 0.000 0.001 0.000

LATT 7

SYMM - X, Y, 1/2 - Z

SFAC C H CL I N ZN

UNIT 216 160 8 24 24 12

MERG 2

DFIX 1.410 0.020 C3 C4

DFIX 1.410 0.020 C2 C5

DFIX 1.410 0.020 C5 C18

DFIX 1.410 0.020 C18 C12

DFIX 1.410 0.020 C12 C3

DFIX 1.410 0.020 C3 C4

DFIX 1.410 0.020 C2 C4

DFIX 1.700 0.020 CL30 C4

FLAT 0.100 C3 C2 C5 C12 C18 C4

DANG 2.700 0.040 C2 CL30

DANG 2.700 0.040 C3 CL30

FMAP 2

PLAN 17

SIZE 0.060 0.070 0.100

ACTA

L.S. 10

TEMP 31.00

WGHT 0.148000 30.486200

FVAR 0.79630

I1 4 0.841866 0.112655 0.146847 11.00000 0.10503 0.10389 =

0.07962 -0.02934 0.01233 0.01545

I2 4 0.662192 0.040945 0.103757 11.00000 0.11971 0.08268 =

0.10285 -0.02228 0.05144 -0.01706

I3 4 0.424545 -0.032872 0.656629 11.00000 0.17367 0.25084 =

0.14328 -0.07318 0.06765 -0.11677

ZN4 6 0.740508 0.155919 0.148786 11.00000 0.11561 0.05402 =

0.04812 -0.00323 0.03729 0.00523

ZN5 6 0.500000 0.049149 0.750000 10.50000 0.09036 0.08046 =

0.07864 0.00000 0.04525 0.00000

N18 5 0.539419 0.132783 0.685182 11.00000 0.08376 0.08362 =

0.06307 -0.00588 0.03451 -0.01476

N24 5 0.721730 0.717130 0.598261 11.00000 0.10180 0.05594 =

0.05322 -0.00750 0.03180 -0.00168

N12 5 0.731571 0.200989 0.254328 11.00000 0.11873 0.05996 =

0.04616 0.00056 0.03761 0.00264

C7 1 0.701619 0.398909 0.477888 11.00000 0.08196 0.06013 =

0.04852 -0.00688 0.03008 -0.00537

AFIX 43

H7 2 0.724587 0.433335 0.452537 11.00000 -1.20000

AFIX 0

C27 1 0.696896 0.534466 0.559718 11.00000 0.08705 0.06159 =

0.04476 -0.00346 0.02808 0.00056

C21 1 0.600314 0.236938 0.600691 11.00000 0.08780 0.06656 =

0.05447 -0.01308 0.03337 -0.00088

C8 1 0.682452 0.437702 0.538182 11.00000 0.08717 0.05965 =

0.04420 -0.00760 0.02905 -0.00090

C11 1 0.653182 0.258552 0.494406 11.00000 0.08468 0.06487 =

0.04361 -0.00861 0.02389 -0.00756

AFIX 43

H11 2 0.642593 0.198845 0.478991 11.00000 -1.20000

AFIX 0

C26 1 0.749995 0.569521 0.561540 11.00000 0.09256 0.06195 =

0.05731 -0.00863 0.03735 0.00181

AFIX 43

H26 2 0.778552 0.531986 0.550267 11.00000 -1.20000

AFIX 0

C9 1 0.648323 0.386178 0.576195 11.00000 0.08431 0.06817 =

0.04459 -0.01056 0.03067 -0.00939

AFIX 43

H9 2 0.634237 0.412804 0.615371 11.00000 -1.20000

AFIX 0

C10 1 0.635043 0.295001 0.556106 11.00000 0.07190 0.06714 =

0.05008 -0.00414 0.02484 -0.00301

C22 1 0.564800 0.170847 0.568027 11.00000 0.09970 0.10416 =

0.05425 -0.00955 0.03091 -0.01485

AFIX 43

H22 2 0.560682 0.160098 0.516963 11.00000 -1.20000

AFIX 0

C28 1 0.655171 0.593983 0.575921 11.00000 0.07981 0.07516 =

0.09642 -0.02390 0.03958 -0.00725

AFIX 43

H28 2 0.618519 0.573050 0.574562 11.00000 -1.20000

AFIX 0

C6 1 0.686898 0.309283 0.454972 11.00000 0.08462 0.06373 =

0.04051 -0.00208 0.02644 0.00147

C25 1 0.760654 0.660862 0.580224 11.00000 0.09056 0.06581 =

0.05960 -0.00473 0.03556 -0.01077

AFIX 43

H25 2 0.796606 0.684153 0.580220 11.00000 -1.20000

AFIX 0

C15 1 0.705362 0.269613 0.386037 11.00000 0.08551 0.05647 =

0.04550 -0.00353 0.02141 -0.00517

C29 1 0.669810 0.685980 0.594277 11.00000 0.09087 0.07383 =

0.09869 -0.02323 0.04582 -0.00296

AFIX 43

H29 2 0.642036 0.726306 0.603947 11.00000 -1.20000

AFIX 0

C23 1 0.534731 0.119441 0.610067 11.00000 0.10712 0.08212 =

0.06996 -0.01463 0.03538 -0.03049

AFIX 43

H23 2 0.510353 0.074017 0.586724 11.00000 -1.20000

AFIX 0

C19 1 0.574197 0.197925 0.715262 11.00000 0.12459 0.10728 =

0.05316 -0.02024 0.04344 -0.03119

AFIX 43

H19 2 0.577945 0.208442 0.766313 11.00000 -1.20000

AFIX 0

C13 1 0.710027 0.150820 0.299443 11.00000 0.17365 0.06075 =

0.06355 -0.01356 0.05707 -0.01381

AFIX 43

H13 2 0.702264 0.089374 0.286665 11.00000 -1.20000

AFIX 0

C16 1 0.732331 0.321070 0.342164 11.00000 0.16014 0.08940 =

0.07341 -0.03433 0.07453 -0.03937

AFIX 43

H16 2 0.742979 0.381236 0.356054 11.00000 -1.20000

AFIX 0

C20 1 0.605585 0.251844 0.675714 11.00000 0.10844 0.10045 =

0.05746 -0.01353 0.03345 -0.03055

AFIX 43

H20 2 0.629739 0.297226 0.699594 11.00000 -1.20000

AFIX 0

C17 1 0.744245 0.285265 0.276937 11.00000 0.14745 0.08463 =

0.07817 -0.03027 0.06356 -0.03325

AFIX 43

H17 2 0.762254 0.323023 0.247415 11.00000 -1.20000

AFIX 0

C14 1 0.697506 0.182593 0.366453 11.00000 0.15460 0.07757 =

0.06085 -0.00248 0.05905 -0.00531

AFIX 43

H14 2 0.683397 0.141761 0.397801 11.00000 -1.20000

AFIX 0

CL30 3 0.490770 0.443832 0.599593 11.00000 0.39526

C3 1 0.553604 0.548796 0.705815 11.00000 0.18356

AFIX 43

H3 2 0.567593 0.496608 0.732836 11.00000 -1.20000

AFIX 0

C2 1 0.494463 0.625908 0.599379 11.00000 0.22893

AFIX 43

H2 2 0.468714 0.621356 0.554580 11.00000 -1.20000

AFIX 0

C5 1 0.514322 0.713394 0.625742 11.00000 0.23556

AFIX 43

H5 2 0.501802 0.765960 0.598238 11.00000 -1.20000

AFIX 0

C12 1 0.570401 0.636884 0.726217 11.00000 0.22239

AFIX 43

H12 2 0.598077 0.641167 0.769182 11.00000 -1.20000

AFIX 0

C18 1 0.552650 0.721524 0.692862 11.00000 0.29734

AFIX 43

H18 2 0.565317 0.778107 0.713660 11.00000 -1.20000

AFIX 0

C4 1 0.512739 0.545833 0.639298 11.00000 0.28699

HKLF 4

REM jm48_auto_a.res in C2/c

REM R1 = 0.0797 for 4954 Fo > 4sig(Fo) and 0.0900 for all 6171 data

REM 286 parameters refined using 13 restraints

END

WGHT 0.1566 31.2944

REM Highest difference peak 1.908, deepest hole -1.688, 1-sigma level 0.165

Q1 1 0.4727 -0.0812 0.6887 11.00000 0.05 1.91

Q2 1 0.6322 0.0577 0.0647 11.00000 0.05 1.82

Q3 1 0.8334 0.0821 0.1818 11.00000 0.05 1.78

Q4 1 0.6601 0.0957 0.0852 11.00000 0.05 1.18

Q5 1 0.6992 0.1572 0.1356 11.00000 0.05 1.14

Q6 1 0.4241 0.0326 0.6277 11.00000 0.05 1.13

Q7 1 0.4123 0.0323 0.6700 11.00000 0.05 1.12

Q8 1 0.6504 0.0519 0.1280 11.00000 0.05 0.94

Q9 1 0.8430 0.1735 0.1528 11.00000 0.05 0.90

Q10 1 0.7046 0.0425 0.1208 11.00000 0.05 0.89

Q11 1 0.5687 0.2292 0.5861 11.00000 0.05 0.69

Q12 1 0.7390 0.1045 0.1404 11.00000 0.05 0.66

Q13 1 0.5270 0.4421 0.6773 11.00000 0.05 0.64

Q14 1 0.7476 0.1567 0.1330 11.00000 0.05 0.57

Q15 1 0.4969 -0.0001 0.7858 11.00000 0.05 0.56

Q16 1 0.6516 0.1986 0.3368 11.00000 0.05 0.56

Q17 1 0.4705 0.5402 0.5806 11.00000 0.05 0.55

;

_shelx_res_checksum 6013

_shelx_hkl_file

;

0 0 -2 27150.9 1249.00 2

0 0 -2 26970.7 1249.62 1

0 0 -3 40.4178 34.7354 2

0 0 -3 31.4949 34.6582 1

0 0 -4 692389. 31929.0 2

0 0 -4 701899. 31929.8 1

0 0 -5 67.9784 52.5307 1

0 0 -5 58.5914 51.1792 2

0 0 -6 4225.28 208.823 5

0 0 -6 4013.09 217.417 4

0 0 -6 4220.74 227.708 2

0 0 -6 3859.55 225.340 1

0 0 -7 37.2443 30.1535 5

0 0 -7 8.65287 45.5903 4

0 0 -7 45.0504 62.1653 2

0 0 -8 35285.1 1693.53 4

0 0 -8 37093.3 1683.90 5

0 0 -8 35598.7 1705.15 2

0 0 -9 62.3574 57.1778 4

0 0 -9 28.9046 41.1033 5

0 0 -9 46.7040 52.6845 17

0 0 -9 67.7599 88.6841 2

0 0 -10 45493.4 2123.64 5

0 0 -10 45622.0 2154.26 2

0 0 -10 45500.7 2114.12 17

0 0 -11 15.4104 47.7974 5

0 0 -11 21.3550 66.7657 17

0 0 -11 69.7588 98.3302 2

0 0 -12 12091.6 598.181 7

0 0 -12 12186.6 662.146 2

0 0 -12 12021.9 621.109 5

0 0 -12 12567.5 592.293 10

0 0 -12 13271.9 627.026 23

0 0 -12 12384.5 601.149 9

0 0 -12 12298.4 614.553 17

0 0 -12 12739.9 591.745 8

0 0 -13 12.9898 48.7389 7

0 0 -13-27.7615 101.478 2

0 0 -13 76.4818 58.9311 5

0 0 -13-1.97590 35.8879 10

0 0 -13-14.7530 87.5533 23

0 0 -13 104.705 54.2246 9

0 0 -13 48.5155 83.7493 17

0 0 -13 77.9549 37.5022 8

0 0 -14 2879.59 178.100 7

0 0 -14 3168.16 168.732 8

0 0 -14 3035.36 167.852 10

0 0 -14 3140.67 217.118 23

0 0 -14 2997.44 203.597 5

0 0 -14 3300.99 209.133 17

0 0 -14 3043.53 254.813 2

0 0 -15 142.333 100.286 17

0 0 -15 23.6557 101.305 23

0 0 -15-31.5152 61.6884 5

0 0 -15-11.7217 130.685 2

0 0 -15 97.2899 60.5262 7

0 0 -15-6.83485 40.0389 8

0 0 -16 5669.60 358.777 23

0 0 -16 6092.68 360.880 5

0 0 -16 6173.82 316.155 8

0 0 -16 6816.92 361.107 17

0 0 -16 6972.80 438.176 2

0 0 -17-43.0223 67.1179 5

0 0 -17 20.0781 46.5737 8

0 0 -17 21.1114 135.143 28

0 0 -17-79.8624 129.562 2

0 0 -17 6.09261 112.556 23

0 0 -18 394.157 58.4712 8

0 0 -18 247.637 141.168 28

0 0 -19 26.1555 46.9374 8

0 0 -19 120.780 133.762 28

0 0 -20 5397.99 274.244 8

0 0 -21 16.9652 38.8144 8

0 0 -22 15.0507 36.6552 8

0 2 0 94050.5 4314.19 13

0 -2 -1 130262. 5869.12 12

0 2 -1 123177. 5870.08 13

0 -2 -1 115902. 5865.58 2

0 2 -1 120178. 5865.92 2

0 -2 -1 125223. 5863.77 5

0 2 -1 128684. 5863.94 5

0 -2 -1 143332. 5865.80 4

0 2 -1 138685. 5865.97 4

0 2 -2 82.0493 22.6117 4

0 -2 -2 72.2105 21.7996 4

0 -2 -2 87.0667 29.0897 2

0 2 -2 41.0033 27.9396 1

0 2 -2 43.0066 26.0017 2

0 -2 -2 52.4211 28.2393 1

0 2 -2 75.8221 13.3659 5

0 -2 -2 68.1500 12.5486 5

0 -2 -3 35685.9 1749.64 2

0 2 -3 36850.7 1750.70 2

0 2 -3 34587.1 1744.82 13

0 -2 -3 37448.1 1750.91 1

0 2 -3 35351.5 1750.39 1

0 -2 -3 39432.3 1743.79 5

0 -2 -3 41953.9 1749.77 4

0 2 -3 39510.6 1749.15 4

0 2 -3 39570.2 1744.02 5

0 -2 -4 66265.9 3071.18 2

0 2 -4 64307.8 3070.93 2

0 2 -4 61370.6 3061.39 13

0 -2 -4 66025.3 3071.45 1

0 2 -4 64292.2 3071.59 1

0 -2 -4 70236.6 3061.95 5

0 -2 -4 70425.5 3068.71 4

0 2 -4 67091.1 3061.75 5

0 2 -4 69775.0 3068.30 4

0 -2 -5 310922. 14315.6 5

0 2 -5 316830. 14316.0 5

0 2 -5 306491. 14322.8 4

0 -2 -5 332520. 14324.6 4

0 -2 -5 301043. 14326.6 2

0 2 -5 303684. 14327.7 2

0 2 -6 278.076 62.7868 2

0 -2 -6 256.078 58.0611 2

0 2 -6 324.597 52.6249 4

0 -2 -6 252.019 51.0504 4

0 2 -6 194.312 33.0664 5

0 -2 -6 143.064 31.9095 5

0 -2 -7 4513.45 260.121 2

0 2 -7 4614.47 261.394 2

0 2 -7 4359.02 237.519 5

0 2 -7 4517.62 248.278 4

0 -2 -7 4638.74 249.241 4

0 -2 -7 4092.01 235.360 5

0 2 -8 1865.03 126.453 4

0 -2 -8 1573.38 124.306 4

0 -2 -8 1864.87 113.845 5

0 2 -8 1952.99 114.462 5

0 -2 -8 1596.72 140.017 2

0 2 -8 1780.44 139.456 2

0 2 -9 42114.0 1928.98 5

0 -2 -9 40735.3 1928.14 5

0 -2 -9 42802.9 1924.72 17

0 2 -9 39384.4 1952.52 2

0 -2 -9 40451.7 1954.38 2

0 -2 -10 889.596 120.521 2

0 2 -10 845.621 115.349 2

0 2 -10 838.635 88.0458 17

0 -2 -10 827.151 88.7287 17

0 -2 -10 803.030 60.4809 15

0 -2 -10 957.272 81.4960 5

0 2 -10 930.930 81.5345 5

0 -2 -11 15228.4 730.914 15

0 2 -11 14480.8 789.775 2

0 -2 -11 15169.1 792.263 2

0 -2 -11 14664.9 753.948 5

0 2 -11 13933.9 753.258 5

0 2 -11 16002.3 751.263 17

0 -2 -11 17135.2 756.145 17

0 2 -12 2245.57 146.699 5

0 -2 -12 1762.81 139.765 5

0 2 -12 1923.22 167.878 28

0 2 -12 2148.40 114.055 8

0 -2 -12 1976.34 113.070 8

0 2 -12 2155.73 114.614 10

0 -2 -12 2107.99 114.225 10

0 2 -12 1939.14 145.858 17

0 -2 -12 2233.34 150.658 17

0 -2 -12 1795.86 112.740 15

0 -2 -12 2415.49 193.787 2

0 2 -12 1902.05 179.234 2

0 -2 -12 1925.58 134.315 26

0 -2 -12 1891.06 124.619 9

0 2 -12 1913.22 125.521 9

0 2 -12 2129.30 169.951 23

0 -2 -12 1881.27 146.777 23

0 2 -12 1790.70 111.354 24

0 2 -12 1820.38 122.428 7

0 -2 -12 2005.15 123.432 7

0 -2 -13 2921.45 184.467 5

0 2 -13 3019.63 187.874 5

0 2 -13 2923.13 150.113 10

0 -2 -13 2852.20 149.701 10

0 2 -13 2701.71 204.177 23

0 -2 -13 2316.78 179.053 23

0 2 -13 2374.40 144.585 24

0 2 -13 2354.49 156.429 7

0 -2 -13 2440.47 156.810 7

0 -2 -13 2907.93 149.126 8

0 2 -13 2958.26 150.612 8

0 2 -13 2631.38 206.232 28

0 -2 -13 2772.10 188.964 17

0 2 -13 2553.11 181.239 17

0 -2 -13 2975.20 239.448 2

0 2 -13 2714.81 232.547 2

0 -2 -14 2802.76 187.457 5

0 2 -14 2624.03 185.778 5

0 -2 -14 2755.90 151.079 10

0 2 -14 2883.53 152.462 10

0 2 -14 3172.04 226.592 28

0 2 -14 2676.40 193.948 17

0 2 -14 2489.40 161.449 7

0 -2 -14 2659.67 162.126 7

0 2 -14 2746.95 151.109 8

0 -2 -14 2815.12 150.275 8

0 -2 -14 2673.34 242.843 2

0 2 -14 2081.32 229.746 2

0 2 -14 2498.92 146.270 24

0 2 -14 2510.74 205.538 23

0 -2 -14 2587.71 186.172 23

0 -2 -15 11798.7 599.327 5

0 2 -15 11499.5 597.445 5

0 2 -15 11843.5 627.059 28

0 -2 -15 12189.2 596.984 28

0 2 -15 12242.5 591.168 17

0 -2 -15 11371.8 563.533 7

0 -2 -15 11810.8 553.446 8

0 2 -15 11640.2 553.013 8

0 2 -15 11399.9 564.331 7

0 2 -15 11354.4 654.225 2

0 -2 -15 11831.5 658.923 2

0 -2 -15 11776.1 589.466 23

0 2 -15 10320.0 545.984 24

0 2 -15 12089.9 612.970 23

0 2 -16 420.655 89.8883 5

0 -2 -16 482.626 86.3127 5

0 2 -16 171.827 151.318 28

0 -2 -16 293.444 117.744 28

0 2 -16 429.624 114.141 17

0 2 -16 448.064 59.5576 8

0 -2 -16 489.936 58.3368 8

0 2 -16 520.150 165.663 2

0 -2 -16 305.728 146.799 2

0 -2 -16 357.170 105.215 23

0 2 -17 379.200 165.516 28

0 -2 -17 552.353 132.261 28

0 2 -17 286.641 110.154 17

0 -2 -17 437.965 57.3370 8

0 2 -17 402.394 58.9367 8

0 -2 -17 448.880 113.521 23

0 2 -17 386.377 86.7710 5

0 -2 -17 331.284 82.2656 5

0 -2 -18-25.3666 95.7130 23

0 -2 -18 99.9369 48.3090 8

0 2 -18 70.4688 46.7663 8

0 -2 -18-6.04702 117.445 28

0 -2 -19 3768.07 197.313 8

0 2 -19 3786.71 197.762 8

0 2 -20 145.040 47.5491 8

0 -2 -20 64.3704 43.7581 8

0 2 -21 249.679 46.4556 8

0 -2 -21 398.659 49.9874 8

0 -2 -22 162.249 37.7778 8

0 2 -22 208.282 38.8420 8

0 4 0 3158.54 164.533 13

0 -4 -1 55307.7 2719.13 12

0 4 -1 59783.6 2721.56 13

0 4 1 62827.4 2722.40 13

0 4 -2 3523.76 171.192 5

0 -4 -2 3554.32 170.940 5

0 -4 -2 3671.77 176.443 2

0 4 -2 3538.99 176.218 2

0 -4 -2 4076.02 178.732 12

0 4 -3 97862.2 4261.15 5

0 -4 -3 94524.4 4260.60 5

0 4 -3 86686.9 4266.62 2

0 -4 -3 88419.8 4266.38 2

0 4 -4 7447.65 361.678 13

0 4 -4 7165.68 362.574 2

0 -4 -4 7080.77 361.346 2

0 -4 -4 7435.90 351.975 5

0 4 -4 7759.74 352.872 5

0 4 -5 19970.6 1012.14 13

0 -4 -5 21182.1 1019.03 2

0 4 -5 21267.8 1019.80 2

0 4 -5 23015.1 1008.11 5

0 -4 -5 22117.0 1007.05 5

0 4 -6 39822.9 1836.99 13

0 -4 -6 38446.3 1830.08 5

0 4 -6 39779.1 1831.66 5

0 -4 -6 41492.2 1848.14 2

0 4 -6 39116.4 1847.22 2

0 -4 -7 1753.93 101.377 5

0 4 -7 1747.43 101.699 5

0 -4 -7 1509.90 122.711 2

0 -4 -8 1420.66 80.0738 15

0 -4 -8 991.021 109.509 2

0 4 -8 922.865 109.678 2

0 4 -8 1561.36 98.8437 17

0 -4 -8 852.887 80.6971 5

0 4 -8 828.454 80.4162 5

0 -4 -9 19340.6 894.287 15

0 -4 -9 18883.5 934.468 2

0 4 -9 18315.1 932.465 2

0 4 -9 19973.7 908.199 17

0 -4 -9 18296.8 906.154 5

0 4 -9 18700.9 907.525 5

0 -4 -9 19343.2 893.940 21

0 -4 -10 26544.4 1225.80 15

0 -4 -10 25514.8 1273.23 2

0 4 -10 24503.3 1271.24 2

0 -4 -10 26753.4 1225.79 21

0 4 -10 27725.9 1241.28 17

0 -4 -10 25469.2 1241.08 5

0 4 -10 25722.6 1242.18 5

0 -4 -11 4192.44 232.221 21

0 4 -11 4097.72 231.970 7

0 -4 -11 4641.74 233.259 7

0 -4 -11 4624.17 224.191 8

0 4 -11 4795.57 224.927 8

0 -4 -11 4450.50 237.822 26

0 4 -11 4762.94 253.885 5

0 -4 -11 4237.01 249.577 5

0 -4 -11 4312.06 227.545 18

0 4 -11 4304.63 250.800 17

0 -4 -11 4369.27 231.356 15

0 -4 -11 4125.57 287.342 2

0 4 -11 4141.86 283.462 2

0 -4 -11 4560.69 224.192 10

0 -4 -11 4624.01 236.775 9

0 4 -11 4800.33 225.329 10

0 4 -11 4298.56 235.228 9

0 4 -11 4730.31 297.968 28

0 4 -11 4555.30 229.479 24

0 4 -12 53.2127 39.8829 24

0 4 -12 31.6182 47.0180 5

0 -4 -12-4.48883 50.5264 5

0 -4 -12 207.426 71.3695 23

0 4 -12 69.9710 31.1258 8

0 -4 -12 18.2542 30.3651 8

0 -4 -12 153.937 64.8192 26

0 -4 -12 110.219 39.3568 18

0 -4 -12 88.2818 44.6222 15

0 4 -12-8.29331 91.7387 2

0 -4 -12-66.0489 103.785 2

0 4 -12 34.6220 30.9832 10

0 -4 -12 77.0646 32.1756 10

0 4 -12 14.6734 44.9919 7

0 -4 -12 114.435 45.7282 7

0 4 -12 292.491 138.303 28

0 -4 -13 6520.33 422.496 2

0 4 -13 7004.15 427.521 2

0 -4 -13 6986.80 360.896 26

0 -4 -13 6662.36 373.743 5

0 4 -13 6670.46 375.028 5

0 -4 -13 6302.20 340.248 18

0 -4 -13 7363.24 342.024 8

0 -4 -13 7270.11 353.757 7

0 4 -13 6898.74 351.507 7

0 4 -13 7304.12 341.986 8

0 -4 -13 7084.98 369.141 23

0 4 -13 6804.93 344.568 24

0 -4 -13 7116.36 348.141 15

0 -4 -14 13058.3 669.541 15

0 -4 -14 13806.3 707.579 5

0 4 -14 14440.1 709.176 5

0 -4 -14 13617.3 688.978 26

0 4 -14 13363.7 668.613 24

0 -4 -14 14287.2 696.482 23

0 4 -14 14355.9 763.007 2

0 -4 -14 13786.7 765.109 2

0 4 -14 14678.8 667.511 8

0 -4 -14 14952.1 667.955 8

0 4 -15 409.248 77.6624 5

0 -4 -15 464.647 85.2798 5

0 -4 -15 319.851 90.7196 23

0 4 -15 286.000 53.2939 24

0 -4 -15 300.330 141.365 2

0 4 -15 299.323 131.991 2

0 4 -15 419.331 51.3345 8

0 -4 -15 346.280 49.9981 8

0 -4 -16 967.448 75.1986 8

0 4 -16 977.315 77.5765 8

0 4 -16 848.230 74.3445 24

0 -4 -16 906.956 116.180 23

0 -4 -16 830.385 106.236 5

0 4 -16 758.198 104.761 5

0 -4 -16 941.124 166.127 2

0 4 -16 1007.92 176.015 2

0 4 -17 5972.61 271.798 8

0 -4 -17 5608.60 270.209 8

0 -4 -17 5227.14 310.088 28

0 -4 -17 4696.69 296.730 23

0 4 -17 4622.05 263.721 24

0 -4 -18 3321.74 180.663 8

0 4 -18 3619.92 183.497 8

0 -4 -18 2945.59 223.458 28

0 -4 -18 2975.85 207.844 23

0 -4 -19 88.8933 40.1448 8

0 4 -19 128.482 44.1794 8

0 -4 -19 14.5220 104.213 28

0 -4 -19 126.658 74.2917 23

0 -4 -20 89.9067 40.2849 8

0 4 -20 184.796 43.3041 8

0 -4 -20 149.867 96.9362 28

0 4 -21 33.0596 34.5874 8

0 6 0 67.4747 36.5099 13

0 6 1 3584.99 183.635 13

0 6 -1 3433.02 182.503 13

0 -6 -1 3045.15 172.323 12

0 -6 -2 2497.55 132.817 12

0 6 2 2881.59 153.098 13

0 6 -2 2614.09 151.702 13

0 6 3 13629.2 654.050 13

0 -6 -4 8796.33 438.020 5

0 6 -4 9657.26 439.604 5

0 6 -4 9226.12 457.892 13

0 6 -4 8889.58 448.524 2

0 -6 -4 9342.25 449.677 2

0 -6 -5 777.464 57.0545 5

0 6 -5 788.812 58.1661 5

0 6 -5 876.402 78.2858 13

0 6 5 1285.97 81.4225 13

0 -6 -5 1227.06 70.3088 15

0 -6 -5 1020.94 81.8262 2

0 6 -5 863.154 75.7850 2

0 6 -6 1286.41 101.596 17

0 6 -6 1577.53 108.318 13

0 6 6 1490.55 101.128 13

0 -6 -6 1361.26 111.020 2

0 6 -6 1357.17 110.072 2

0 6 -6 1849.09 95.8762 5

0 -6 -6 1637.84 91.6336 5

0 6 -7 2526.81 142.346 5

0 -6 -7 2415.62 140.517 5

0 6 -7 2378.03 155.027 13

0 6 7 2854.92 149.421 13

0 6 -7 2683.19 151.587 17

0 -6 -7 2740.11 145.307 21

0 6 -7 2344.81 162.863 2

0 -6 -7 2457.01 164.432 2

0 -6 -8 2513.04 183.429 2

0 6 -8 2231.64 181.018 2

0 -6 -8 3504.21 161.673 15

0 -6 -8 3976.99 166.047 21

0 6 -8 2417.84 157.706 5

0 -6 -8 2172.70 154.048 5

0 6 -8 3509.78 173.737 17

0 6 -9 16972.2 809.028 17

0 -6 -9 16672.2 797.507 21

0 6 -9 15996.2 833.973 2

0 -6 -9 15945.3 832.822 2

0 -6 -9 17056.0 798.601 15

0 -6 -9 17704.3 799.944 26

0 -6 -9 16227.3 804.914 5

0 6 -9 17403.6 808.263 5

0 -6 -10 3808.57 258.871 2

0 6 -10 3492.92 255.710 2

0 -6 -10 4092.02 218.156 21

0 6 -10 4105.67 213.095 24

0 -6 -10 4024.63 217.122 15

0 6 -10 4255.85 229.560 5

0 -6 -10 4153.10 227.449 5

0 6 -10 4443.56 234.747 17

0 6 -11 9601.42 479.935 17

0 -6 -11 8991.42 462.722 15

0 6 -11 9081.69 517.416 2

0 -6 -11 9988.05 523.793 2

0 -6 -11 9828.27 466.345 21

0 -6 -11 9500.06 449.446 8

0 6 -11 9607.17 460.397 24

0 6 -11 9674.99 479.442 5

0 -6 -11 9420.84 477.715 5

0 -6 -11 9035.47 459.374 18

0 6 -12 2177.23 154.226 17

0 -6 -12 2031.70 128.218 15

0 6 -12 1935.16 186.662 2

0 -6 -12 1811.89 182.697 2

0 -6 -12 2108.40 133.945 21

0 6 -12 2003.43 110.833 8

0 -6 -12 1815.52 108.703 8

0 6 -12 1994.85 122.397 24

0 -6 -12 1872.19 137.321 5

0 6 -12 1863.52 137.891 5

0 -6 -12 1931.54 123.290 18

0 -6 -12 2024.26 135.770 26

0 -6 -13 10758.8 535.686 15

0 -6 -13 10756.9 607.765 2

0 6 -13 10792.2 609.069 2

0 -6 -13 11579.2 523.651 8

0 6 -13 11677.9 524.174 8

0 -6 -13 10326.7 547.784 23

0 6 -13 10148.6 530.021 24

0 6 -13 11095.1 560.628 5

0 -6 -13 11005.9 559.479 5

0 6 -13 11510.7 558.733 17

0 -6 -13 10600.7 530.519 18

0 -6 -13 10332.5 542.821 26

0 -6 -14 1903.69 129.658 15

0 6 -14 1776.41 198.031 2

0 -6 -14 1825.00 122.310 18

0 -6 -14 1645.77 143.725 5

0 6 -14 1710.04 145.643 5

0 -6 -14 1949.41 146.684 26

0 6 -14 2235.56 118.156 8

0 -6 -14 2047.78 115.435 8

0 6 -14 2050.63 126.510 24

0 -6 -14 1994.75 145.755 23

0 6 -14 2102.74 162.834 17

0 6 -15 3777.75 221.859 17

0 -6 -15 2917.63 205.838 5

0 6 -15 3001.59 178.492 24

0 -6 -15 2884.50 198.027 23

0 6 -15 3556.47 174.287 8

0 -6 -15 3196.97 171.300 8

0 6 -15 2655.84 253.142 2

0 -6 -15 3180.49 203.350 26

0 6 -16 120.921 97.7952 17

0 6 -16 46.3950 39.4439 8

0 -6 -16 79.1827 38.5195 8

0 6 -16 45.0472 48.1923 24

0 -6 -16-36.7049 70.0246 23

0 -6 -16 110.638 95.1774 28

0 6 -17 1452.84 140.906 17

0 6 -17 1313.90 86.5529 8

0 6 -17 1129.36 88.5948 24

0 -6 -17 1217.21 114.858 23

0 -6 -17 1146.13 131.740 28

0 6 -18 227.735 89.4652 17

0 6 -18 211.389 43.9679 8

0 -6 -18 134.167 69.1466 23

0 -6 -18 318.263 99.8306 28

0 -6 -19 861.878 115.192 28

0 6 -19 933.172 67.5420 8

0 -6 -20 49.1870 81.3741 28

0 8 0 3675.36 202.403 13

0 -8 0 3263.38 185.300 12

0 -8 0 3372.59 175.691 21

0 8 1 14761.0 664.257 13

0 8 -1 14708.6 665.124 13

0 -8 -1 13197.5 636.255 21

0 -8 1 12937.8 634.293 21

0 -8 -2 347.306 47.1382 15

0 8 2 346.785 61.8710 13

0 8 -2 320.946 60.3791 13

0 -8 -2 294.929 45.4981 21

0 -8 -3 26711.3 1250.27 15

0 8 3 26541.1 1269.69 13

0 -8 -3 27114.4 1249.66 21

0 -8 -4 8164.95 402.188 15

0 -8 -4 8473.12 402.991 21

0 8 4 8150.10 420.396 13

0 -8 -5 12710.7 604.604 15

0 8 -5 11960.8 606.906 17

0 -8 -5 13220.8 605.060 21

0 8 -5 12443.8 624.830 13

0 8 5 12918.6 621.009 13

0 -8 -6 22846.6 1059.17 21

0 8 -6 23517.4 1055.00 24

0 8 -6 21106.3 1051.67 16

0 -8 -6 23012.4 1059.84 15

0 8 -6 21338.7 1061.41 17

0 -8 -6 23658.4 1058.84 26

0 8 -6 22336.2 1082.12 13

0 8 6 23135.5 1073.37 13

0 8 -7 7401.79 343.970 24

0 8 -7 6484.86 342.441 16

0 -8 -7 7065.97 349.423 15

0 8 -7 6752.83 352.820 17

0 8 7 6593.34 354.950 13

0 8 -7 6334.24 366.374 13

0 -8 -7 7784.51 349.294 26

0 8 -8 2844.92 180.560 17

0 -8 -8 3004.67 177.130 25

0 -8 -8 3266.04 174.554 26

0 -8 -8 3159.68 175.292 21

0 8 -8 2894.87 164.548 24

0 8 -9 71.0693 61.8441 17

0 -8 -9 113.106 52.9045 26

0 -8 -9 118.231 54.0598 25

0 8 -9 70.3075 42.2088 24

0 -8 -9 143.506 58.5414 21

0 -8 -10 906.849 83.6733 18

0 8 -10 1095.65 82.0434 24

0 -8 -10 934.321 87.4399 15

0 8 -10 1019.49 101.538 17

0 -8 -10 1007.71 92.1616 26

0 -8 -10 935.163 89.0264 25

0 -8 -10 875.651 88.7841 21

0 -8 -11 6232.89 331.911 18

0 8 -11 6095.40 329.578 24

0 8 -11 6466.96 348.291 17

0 -8 -11 7112.79 341.222 26

0 -8 -11 6459.51 337.306 15

0 -8 -11 7020.49 342.509 21

0 -8 -12 847.270 87.1415 18

0 8 -12 920.832 109.418 17

0 -8 -12 1062.08 100.622 26

0 -8 -12 911.788 93.8003 15

0 -8 -12 908.552 97.6808 21

0 -8 -13 129.610 52.4093 18

0 8 -13 172.428 87.4688 17

0 -8 -13 270.897 73.6505 23

0 -8 -13 152.014 52.8997 20

0 -8 -13 171.190 74.9579 21

0 8 -14 3980.93 209.276 24

0 -8 -14 3586.10 218.511 23

0 -8 -14 3587.86 208.648 18

0 8 -14 3994.83 235.672 17

0 -8 -15 265.042 73.2957 23

0 8 -15 329.245 57.3133 24

0 8 -15 395.525 90.3561 17

0 8 -16-30.0666 46.2962 24

0 -8 -16 4.10312 85.3835 28

0 8 -16-4.09726 78.6895 17

0 8 -17 567.643 63.4896 24

0 -8 -17 575.354 97.0331 28

0 8 -17 608.267 95.5613 17

0 8 -18 434.041 54.9609 24

0 8 -18 484.423 86.4959 17

0 8 -18 486.085 55.3624 22

0 -8 -18 390.847 86.3577 28

0 -8 -19 123.492 70.5744 28

0 -10 0 12690.4 623.544 21

0 -10 0 13248.8 623.451 18

0 10 0 14063.8 662.088 13

0 -10 -1 1951.75 120.310 21

0 -10 1 1828.78 115.671 21

0 10 1 2437.65 154.261 13

0 10 -1 2335.77 155.628 13

0 -10 -1 2069.38 119.748 18

0 -10 1 2016.81 115.226 18

0 -10 -2 16.6479 42.6439 18

0 10 2 52.8843 54.5184 13

0 10 -2 60.7005 56.4402 13

0 -10 2 82.1325 37.6311 21

0 -10 -2 32.7123 45.7554 21

0 -10 -3 5368.67 269.878 21

0 10 3 5185.83 296.960 13

0 10 -3 5290.68 299.726 13

0 -10 -3 5199.61 267.737 18

0 10 -3 5223.04 262.199 24

0 -10 -4 4391.02 231.029 21

0 10 -4 4492.57 223.366 24

0 10 4 4424.41 255.301 13

0 -10 -4 4099.46 228.875 18

0 -10 -5 2005.87 128.668 21

0 10 -5 2258.37 119.857 24

0 -10 -5 2203.92 128.150 26

0 10 -5 1616.31 125.013 17

0 -10 -5 1772.32 124.096 18

0 10 5 1984.39 142.703 13

0 10 -5 2177.43 153.866 13

0 10 -6 1398.97 125.747 13

0 10 6 1227.70 108.268 13

0 -10 -6 1122.79 96.4949 18

0 10 -6 1343.62 90.1760 24

0 10 -6 1552.30 96.1070 16

0 10 -6 1122.01 99.2435 17

0 -10 -6 1347.91 100.427 26

0 -10 -6 1311.36 102.771 21

0 -10 -7 1094.57 94.1542 18

0 10 -7 1337.20 90.1849 24

0 10 -7 1090.91 95.0356 16

0 10 -7 1097.67 99.5187 17

0 -10 -7 1391.08 100.868 26

0 -10 -7 1188.77 102.991 21

0 10 -8 435.423 76.6111 17

0 -10 -8 416.332 70.9974 18

0 -10 -8 565.218 74.7199 26

0 10 -8 571.805 79.0222 16

0 10 -8 529.279 60.0821 24

0 10 -9 1015.51 102.021 17

0 -10 -9 1302.63 102.748 26

0 10 -9 1148.16 89.3690 24

0 10 -10 2009.11 123.525 24

0 -10 -10 1829.43 134.173 26

0 10 -10 1754.21 135.005 17

0 -10 -10 1648.52 127.262 18

0 -10 -10 1922.38 134.767 21

0 10 -11 134.939 71.0980 17

0 -10 -11 221.821 67.7752 18

0 -10 -11 246.747 77.8583 21

0 -10 -11 211.739 72.2974 26

0 10 -11 187.703 54.4835 24

0 10 -12 609.194 88.9067 17

0 -10 -12 554.023 78.2253 18

0 -10 -12 617.083 89.6076 21

0 -10 -12 678.604 93.1187 26

0 10 -12 714.224 73.3317 24

0 10 -13 468.880 81.0820 17

0 -10 -13 391.847 71.3590 18

0 -10 -13 567.262 91.9982 26

0 10 -13 449.046 67.3977 24

0 -10 -14 720.426 104.650 26

0 10 -14 716.814 91.7907 17

0 -10 -14 854.567 83.9552 18

0 -10 -15 84.6544 72.5264 28

0 -10 -16 12.0311 66.8591 28

0 10 -16 2.26762 47.2970 24

0 -10 -17 20.0602 57.4021 28

0 10 -17 42.2674 43.8358 24

0 10 -18 16.0505 38.3789 22

0 12 0 8530.69 444.891 13

0 -12 0 7675.88 396.363 21

0 -12 0 8271.63 398.362 18

0 -12 -1 486.812 62.6320 21

0 -12 1 368.181 53.6686 21

0 12 -1 620.731 93.0327 13

0 12 1 434.524 85.6701 13

0 -12 -1 398.288 60.6772 18

0 -12 -2 29.2269 53.9491 21

0 12 2 54.3000 62.4402 13

0 12 -2 59.8668 68.8338 13

0 -12 -2 53.6626 49.0972 18

0 12 -3 81.4009 71.1604 13

0 12 3-23.3844 62.1754 13

0 -12 -3 11.2455 49.8093 18

0 -12 -3 78.3993 49.4663 26

0 -12 -3-25.1305 55.3085 21

0 -12 -4 1743.90 127.354 21

0 12 -4 1860.91 116.946 24

0 12 4 1755.55 145.666 13

0 -12 -4 2068.49 124.342 26

0 12 -4 2026.60 125.995 16

0 -12 -4 1642.16 123.756 18

0 -12 -5 370.422 72.0379 21

0 12 -5 396.533 56.3760 24

0 12 5 478.829 74.8765 13

0 12 -5 553.914 70.8802 16

0 -12 -5 448.307 70.9420 18

0 -12 -5 363.495 67.0589 26

0 -12 -6 3990.39 228.465 21

0 -12 -6 3985.55 228.559 18

0 12 -6 4155.45 231.901 16

0 -12 -6 4519.57 229.263 26

0 12 -6 3963.04 219.272 24

0 -12 -7 903.441 98.3477 21

0 -12 -7 897.345 96.4607 18

0 12 -7 1004.14 102.971 16

0 12 -7 912.209 82.6816 24

0 12 -8 1988.13 130.240 24

0 -12 -8 1931.73 138.315 18

0 -12 -8 2223.88 141.282 26

0 -12 -8 2002.11 142.484 21

0 -12 -9 327.600 78.8164 21

0 -12 -9 305.077 72.8576 26

0 -12 -9 362.351 73.7795 18

0 12 -9 432.042 65.3022 24

0 -12 -10 290.046 76.4395 26

0 12 -10 404.688 62.5745 24

0 -12 -11 26.1082 66.9907 26

0 -12 -11 10.5730 72.5993 21

0 12 -11 102.161 55.3768 24

0 -12 -12 62.3549 55.5573 20

0 12 -12 98.9117 54.0605 24

0 -12 -12 102.787 60.8663 18

0 -12 -12 112.792 73.5047 26

0 12 -13 58.5344 51.6532 24

0 -12 -13 102.376 56.8210 18

0 -12 -13 112.871 73.3036 26

0 -12 -14 558.456 71.5345 18

0 12 -14 625.417 66.1630 24

0 12 -16-7.92051 37.3972 22

0 12 -17 20.8102 33.1473 22

0 -14 0 3548.28 193.320 21

0 -14 -1 58.6296 52.6549 21

0 -14 -1 42.0251 54.2492 18

0 14 -2 100.419 62.1325 16

0 -14 -2 125.622 58.1394 18

0 -14 -2 29.6406 52.4461 21

0 -14 -3 1780.89 126.182 18

0 -14 -3 1578.34 121.018 21

0 -14 -4 2073.47 145.951 18

0 -14 -4 2013.85 142.546 21

0 -14 -5 383.810 73.0121 21

0 -14 -5 340.047 70.1928 18

0 -14 -5 264.905 60.4476 26

0 14 -5 338.424 83.1185 16

0 -14 -6 12.8409 58.1604 18

0 -14 -6 87.7740 56.2668 26

0 -14 -6-18.3685 61.1952 21

0 -14 -7 481.286 76.7023 18

0 -14 -7 443.924 77.5188 21

0 -14 -7 505.560 71.2552 26

0 -14 -8 846.037 84.2903 26

0 -14 -8 751.809 86.0961 18

0 -14 -8 852.015 90.8289 21

0 -14 -9 422.982 71.4479 18

0 -14 -9 489.984 73.0920 26

0 -14 -9 441.473 76.7076 21

0 -14 -10 52.2351 58.5085 18

0 -14 -10-79.0498 61.1137 26

0 -14 -10 26.5932 52.0484 20

0 -14 -11 371.459 67.3965 18

0 -14 -11 177.312 60.0803 20

0 -14 -11 404.767 70.7002 26

0 14 -12 24.5749 37.0307 22

0 14 -13 124.486 37.3025 22

0 -16 0 815.091 76.8377 20

0 16 0 1034.93 98.8943 16

0 16 1 283.903 70.8762 16

0 16 -1 260.313 77.2854 16

0 16 -2 48.1776 73.8096 16

0 -16 -3 575.557 69.8173 21

0 16 3 663.067 80.9154 16

0 16 -3 764.522 101.854 16

0 -16 -4 731.717 78.5016 21

0 16 4 984.039 87.6942 16

0 -16 -5 459.541 68.2211 20

0 -16 -5 503.288 68.9540 21

0 16 6 114.204 53.0039 16

0 -16 -6 98.9229 53.5855 20

0 16 7 112.387 50.0064 16

0 -16 -7 33.9093 52.0313 20

0 16 8-1.92822 43.5196 16

1 1 -22 184.291 41.5473 8

1 1 -21 578.666 59.5661 8

1 1 -20 17.3672 45.4141 8

1 -1 -20 193.718 133.819 28

1 1 -19-28.5214 47.2121 8

1 -1 -18 619.398 129.496 23

1 1 -18 775.966 169.304 28

1 1 -18 762.360 74.3795 8

1 1 -17 3228.01 177.343 8

1 1 -17 3015.29 257.444 28

1 1 -17 3170.06 239.327 23

1 -1 -17 3306.11 232.383 23

1 1 -17 3439.25 230.015 17

1 1 -17 2839.64 215.189 5

1 -1 -17 2836.94 215.374 5

1 -1 -17 3306.34 292.702 2

1 -1 -16 177.773 137.162 2

1 1 -16 171.171 49.4900 8

1 1 -16 293.757 138.840 28

1 1 -16 284.632 120.915 23

1 -1 -16 92.3255 105.659 23

1 -1 -16 398.718 74.8940 7

1 1 -16 143.110 69.7516 5

1 1 -16 116.063 107.037 17

1 1 -15 314.571 106.662 17

1 -1 -15 322.444 106.499 17

1 1 -15 362.727 52.7223 8

1 1 -15 347.024 133.704 28

1 1 -15 376.926 129.535 23

1 -1 -15 157.072 108.761 23

1 1 -15 347.929 80.2955 5

1 -1 -15 319.433 136.921 2

1 -1 -15 255.505 64.3920 7

1 -1 -14 376.360 66.5086 7

1 1 -14 494.060 54.0735 8

1 1 -14 355.124 123.880 28

1 1 -14 425.596 124.868 23

1 -1 -14 455.732 107.091 23

1 -1 -14 369.927 71.3062 9

1 1 -14 543.612 84.2328 5

1 1 -14 384.472 93.1805 17

1 -1 -14 407.689 103.004 17

1 -1 -14 495.026 138.008 2

1 -1 -13 5020.86 273.384 17

1 1 -13 4709.17 270.909 17

1 -1 -13 4495.25 249.749 7

1 1 -13 4773.73 241.861 8

1 1 -13 5467.61 300.091 28

1 1 -13 4689.59 275.133 5

1 1 -13 4857.42 242.948 10

1 -1 -13 4530.61 252.600 9

1 -1 -13 4806.02 284.520 23

1 1 -13 5089.90 295.470 23

1 -1 -13 4875.16 328.528 2

1 -1 -12 5181.33 298.283 17

1 1 -12 5151.99 297.450 17

1 -1 -12 5086.05 281.819 7

1 1 -12 6255.78 311.091 5

1 1 -12 5548.90 315.427 28

1 -1 -12 5466.93 275.135 10

1 1 -12 5945.01 276.734 10

1 -1 -12 5060.92 283.248 9

1 1 -12 5750.24 321.463 23

1 -1 -12 5068.93 307.502 23

1 1 -12 6298.50 277.313 8

1 -1 -12 5707.70 351.034 2

1 -1 -11 6655.89 402.158 2

1 1 -11 7618.44 368.410 5

1 -1 -11 7087.44 361.457 17

1 1 -11 6657.91 356.932 17

1 -1 -10 11037.5 596.470 2

1 -1 -10 10814.3 577.129 4

1 1 -10 12105.1 568.655 5

1 -1 -10 11469.0 560.638 17

1 1 -10 11827.5 559.618 17

1 -1 -9 1457.27 145.142 2

1 1 -9 1575.19 112.332 5

1 -1 -9 1605.72 129.062 4

1 -1 -9 2016.67 117.719 17

1 1 -9 1941.58 115.314 17

1 -1 -8 9024.69 467.746 2

1 -1 -8 8970.25 453.944 4

1 1 -8 9107.32 442.350 5

1 -1 -7 35923.8 1647.30 2

1 -1 -7 33572.7 1636.51 4

1 1 -7 35688.8 1628.95 5

1 -1 -6 95580.6 4563.14 2

1 -1 -6 99470.4 4557.88 4

1 1 -6 100600. 4549.56 5

1 -1 -5 52701.1 2435.67 2

1 -1 -5 51520.7 2412.36 11

1 -1 -5 55607.7 2431.18 4

1 1 -5 54303.4 2423.69 5

1 -1 -4 27032.1 1263.50 2

1 1 -4 27029.5 1250.40 13

1 -1 -4 27868.2 1259.86 4

1 1 -4 26835.2 1253.90 5

1 -1 -3 63624.6 2973.67 2

1 1 -3 62686.4 2964.61 13

1 1 -3 66331.1 2967.41 5

1 -1 -3 67175.4 2971.69 4

1 1 -2 20930.0 985.200 13

1 1 -2 21212.0 985.122 5

1 -1 -2 22957.2 988.609 4

1 -1 -2 20844.1 989.415 2

1 1 -1 19963.7 935.093 3

1 -1 -1 21473.0 936.181 4

-1 1 1 21690.4 934.501 13

1 -1 -1 18604.0 936.742 2

1 1 -1 19652.9 934.176 5

-1 1 0 47591.6 2183.02 13

-1 -1 -1 5808.56 274.079 1

-1 -1 -2 217829. 10117.8 1

-1 -1 -2 223508. 10117.0 2

-1 -1 -3 64073.7 2963.29 2

-1 -1 -3 67469.0 2961.46 4

-1 -1 -3 61070.7 2963.81 1

-1 -1 -4 1619.22 102.694 2

1 -1 4 1686.42 87.3360 11

-1 -1 -4 1450.69 105.280 1

-1 -1 -4 1709.75 100.138 4

-1 -1 -5 38817.9 1657.48 4

-1 1 -5 35976.3 1647.79 5

-1 -1 -5 33847.9 1660.42 1

1 -1 5 34530.9 1653.43 11

-1 -1 -5 34781.1 1659.08 2

-1 -1 -6 63819.6 3097.66 2

-1 1 -6 68852.1 3083.68 5

-1 -1 -6 69424.0 3094.03 4

-1 -1 -6 63835.4 3099.22 1

-1 -1 -7 18669.1 922.305 2

-1 1 -7 19522.5 903.122 5

-1 -1 -7 19121.4 925.015 1

-1 1 -8 1479.08 100.031 5

-1 -1 -8 1462.20 131.567 2

-1 1 -9 5971.51 320.774 5

-1 1 -9 6569.71 319.737 17

-1 -1 -9 6457.19 318.904 17

-1 -1 -9 5795.14 348.795 2

-1 1 -10 4289.05 204.315 5

-1 1 -10 2930.64 193.073 17

-1 -1 -10 3068.85 192.701 17

-1 -1 -10 3776.10 238.933 2

-1 -1 -11 16.2778 29.0611 15

-1 1 -11 67.8026 48.3442 5

-1 1 -11 31.8946 68.6195 17

-1 -1 -11 104.425 70.9751 17

-1 -1 -11 129.871 103.007 2

-1 -1 -12 2140.17 197.632 2

-1 1 -12 2398.59 128.242 10

-1 -1 -12 2087.30 134.072 7

-1 1 -12 2515.53 128.524 8

-1 -1 -12 2116.84 156.510 23

-1 1 -12 1837.67 162.651 23

-1 1 -12 2457.17 158.739 5

-1 -1 -12 2218.25 159.992 17

-1 1 -12 1990.46 154.829 17

-1 1 -12 2150.63 169.249 28

-1 -1 -13 12097.3 677.686 2

-1 1 -13 13129.0 600.801 10

-1 -1 -13 12033.4 606.615 7

-1 1 -13 13041.4 599.372 8

-1 1 -13 11550.0 593.743 24

-1 1 -13 13008.7 640.385 23

-1 -1 -13 12865.8 630.682 23

-1 1 -13 11689.0 631.049 5

-1 -1 -13 12666.4 629.095 17

-1 1 -13 12974.3 629.375 17

-1 1 -13 13633.7 646.787 28

-1 -1 -13 13711.3 636.114 28

-1 -1 -14 11378.7 665.608 2

-1 1 -14 12042.7 577.090 10

-1 -1 -14 11714.6 585.969 7

-1 1 -14 12232.1 576.472 8

-1 1 -14 12033.9 618.133 23

-1 -1 -14 11889.1 609.739 23

-1 1 -14 11198.0 613.734 5

-1 1 -14 12098.0 609.558 17

-1 -1 -14 12865.4 615.845 17

-1 1 -14 12137.1 629.631 28

-1 -1 -14 12245.9 616.034 28

-1 -1 -15 47.1437 138.252 2

-1 1 -15 84.1490 45.6902 10

-1 1 -15 39.3393 42.0172 8

-1 1 -15 112.213 104.999 23

-1 -1 -15 105.326 96.6074 23

-1 1 -15 98.3264 107.978 17

-1 -1 -15 223.387 110.850 17

-1 1 -15 25.4802 62.4076 5

-1 -1 -15 17.0711 104.852 28

-1 1 -15 6.71838 119.046 28

-1 -1 -16 176.758 118.582 28

-1 1 -16 216.417 142.516 28

-1 1 -16 180.435 50.1090 8

-1 1 -16 128.359 113.176 23

-1 -1 -16-32.7231 97.8419 23

-1 1 -16 237.590 114.003 17

-1 -1 -16 178.915 147.270 2

-1 1 -16 240.884 77.1052 5

-1 1 -17 5664.88 273.382 8

-1 -1 -17 4632.99 302.124 23

-1 -1 -17 4889.52 316.050 5

-1 1 -17 5256.75 320.720 5

-1 1 -17 5186.60 341.980 28

-1 -1 -17 4714.51 323.530 28

-1 1 -17 5789.83 321.952 17

-1 1 -18 3169.94 178.293 8

-1 1 -19 249.489 51.9125 8

-1 1 -20 62.7860 45.3486 8

-1 1 -21 592.989 58.9945 8

-1 1 -22 751.765 58.8209 8

1 3 -22 301.510 40.6496 8

1 -3 -21 451.959 52.2241 8

1 3 -21 514.324 52.8971 8

1 -3 -20 785.473 134.534 28

1 3 -20 817.156 69.3939 8

1 -3 -20 795.504 69.1623 8

1 3 -19 78.0774 45.2771 8

1 -3 -19 60.3537 41.0695 8

1 -3 -19 90.6335 92.2292 23

1 -3 -19 154.891 116.237 28

1 -3 -18 763.829 71.9425 8

1 3 -18 788.287 70.9626 8

1 -3 -18 863.984 123.043 23

1 -3 -18 880.960 143.141 28

1 3 -18 763.630 128.620 17

1 3 -17 544.911 61.8682 8

1 -3 -17 523.948 62.3116 8

1 -3 -17 510.025 113.958 23

1 -3 -17 419.004 122.082 28

1 -3 -16 6078.73 371.644 28

1 3 -16 6883.82 374.921 5

1 -3 -16 6642.61 372.608 5

1 3 -16 7308.60 329.074 8

1 -3 -16 6824.40 327.189 8

1 3 -16 6499.02 435.230 2

1 -3 -16 6833.30 443.888 2

1 -3 -16 6461.67 365.253 23

1 3 -16 5651.72 320.122 24

1 3 -15 96.6030 153.086 28

1 3 -15 68.1252 39.2358 24

1 -3 -15 94.0277 99.0255 23

1 3 -15 112.479 63.2337 5

1 -3 -15 31.2176 61.2651 5

1 3 -15 84.3246 41.1206 8

1 -3 -15 99.7172 40.6346 8

1 -3 -15 195.023 65.0681 7

1 3 -15 183.956 66.0729 7

1 3 -15 197.453 129.037 2

1 -3 -15 11.6365 128.206 2

1 -3 -14 1005.95 75.7720 15

1 3 -14 809.868 166.453 2

1 -3 -14 852.011 165.670 2

1 3 -14 1191.99 179.256 28

1 3 -14 953.024 107.762 5

1 -3 -14 950.477 103.776 5

1 -3 -14 978.301 87.2542 7

1 -3 -14 1002.66 74.3650 8

1 3 -14 1007.82 74.9939 8

1 3 -14 931.618 88.0355 7

1 -3 -14 1106.66 122.438 23

1 3 -14 1084.79 160.594 23

1 3 -14 918.015 72.9697 24

1 -3 -14 1065.15 107.754 26

1 -3 -13 626.145 68.2928 7

1 3 -13 554.685 68.0581 7

1 -3 -13 620.544 59.6665 15

1 -3 -13 439.438 132.281 2

1 3 -13 628.374 136.995 2

1 3 -13 692.687 141.806 28

1 -3 -13 610.711 70.0256 9

1 3 -13 603.839 71.7379 9

1 -3 -13 472.052 77.8535 5

1 3 -13 494.436 78.5080 5

1 -3 -13 447.143 52.3033 8

1 3 -13 502.786 52.0715 8

1 -3 -13 715.659 97.4824 23

1 3 -13 763.832 133.767 23

1 3 -13 577.563 56.2932 24

1 -3 -13 725.205 85.4215 26

1 3 -12 58561.1 2522.02 23

1 3 -12 51289.6 2465.27 24

1 -3 -12 56469.9 2495.93 23

1 -3 -12 53376.5 2474.91 9

1 3 -12 51382.4 2474.33 9

1 -3 -12 52397.4 2464.28 8

1 3 -12 55496.7 2465.03 8

1 -3 -12 52760.9 2477.98 26

1 -3 -12 52822.1 2472.73 7

1 3 -12 51519.1 2472.03 7

1 -3 -12 51849.5 2467.09 15

1 -3 -12 54907.8 2465.21 10

1 3 -12 55194.6 2465.50 10

1 3 -12 51647.2 2536.75 2

1 -3 -12 51654.7 2538.36 2

1 -3 -12 52248.6 2496.49 5

1 3 -12 56078.6 2498.88 5

1 3 -12 55875.9 2521.78 28

1 3 -11 6914.74 344.232 5

1 -3 -11 7132.01 342.371 5

1 -3 -11 6081.58 311.705 18

1 3 -11 6655.70 363.473 23

1 -3 -11 6264.54 315.266 15

1 -3 -11 6551.43 379.205 2

1 3 -11 6428.53 380.004 2

1 -3 -10 9744.13 470.829 5

1 3 -10 10185.1 473.980 5

1 3 -10 9339.06 465.138 17

1 3 -10 9503.17 503.603 2

1 -3 -10 9948.85 506.493 2

1 -3 -10 9650.20 450.804 15

1 -3 -10 9098.69 451.829 21

1 -3 -10 8971.66 446.903 18

1 -3 -9 19903.5 951.560 15

1 -3 -9 19180.4 951.737 21

1 3 -9 20454.2 963.246 17

1 -3 -9 21525.2 968.556 5

1 3 -9 22613.8 971.112 5

1 3 -9 19909.8 980.100 4

1 -3 -9 21209.6 982.328 4

1 3 -9 20842.1 997.342 2

1 -3 -9 20541.1 994.088 2

1 3 -8 62195.7 2720.11 17

1 -3 -8 61422.6 2724.12 5

1 3 -8 63380.3 2725.78 5

1 3 -8 58646.3 2735.53 4

1 -3 -8 61627.1 2737.56 4

1 3 -8 59564.4 2747.43 2

1 -3 -8 59389.7 2745.77 2

1 -3 -8 54668.6 2705.14 11

1 -3 -7 3310.83 171.980 5

1 3 -7 3552.22 173.957 5

1 -3 -7 3015.65 182.362 4

1 3 -7 3113.28 184.070 4

1 -3 -7 3267.89 195.505 2

1 3 -7 2873.45 190.122 2

1 -3 -6 8105.35 393.234 4

1 3 -6 7853.80 392.948 4

1 -3 -6 7953.39 383.304 5

1 3 -6 8561.49 384.750 5

1 -3 -6 7473.22 397.802 2

1 3 -6 7887.19 400.938 2

1 3 -6 7645.53 383.665 13

1 -3 -5 29742.7 1271.34 4

1 3 -5 28026.4 1269.94 4

1 3 -5 27927.2 1262.18 5

1 -3 -5 27035.1 1261.22 5

1 3 -5 25923.2 1273.30 2

1 -3 -5 26541.4 1273.00 2

1 3 -5 25715.3 1262.74 13

1 3 -4 5835.39 259.357 4

1 -3 -4 4934.01 249.870 5

1 -3 -4 5518.12 258.286 4

1 3 -4 5563.69 251.580 5

1 -3 -4 4812.85 260.023 2

1 3 -4 5011.96 261.263 2

1 3 -4 4872.80 254.048 13

1 -3 -3 105877. 4935.49 5

1 -3 -3 121722. 4941.71 4

1 3 -3 112308. 4940.75 4

1 3 -3 107323. 4935.89 5

1 3 -3 100880. 4941.86 2

1 -3 -3 103959. 4941.99 2

1 -3 -3 106134. 4940.59 12

1 3 -3 104006. 4941.80 13

1 -3 -2 52104.2 2246.58 4

1 -3 -2 47083.4 2243.24 5

1 3 -2 48990.0 2243.82 5

1 3 -2 50604.7 2246.61 4

1 -3 -2 47365.0 2248.52 2

1 3 -2 44481.8 2247.91 2

-1 3 2 53172.2 2251.25 13

1 -3 -2 48999.3 2253.33 12

1 -3 -1 113727. 5102.75 4

1 3 -1 107481. 5102.74 4

-1 3 1 116403. 5111.80 13

-1 3 0 118925. 5456.47 13

-1 3 -1 105417. 4942.24 1

-1 -3 -1 101303. 4941.12 1

-1 3 -1 105042. 4941.34 2

-1 -3 -1 109987. 4941.34 2

-1 -3 -1 111388. 4939.32 12

-1 3 -1 117874. 4949.98 13

-1 -3 -2 10196.2 485.605 1

-1 3 -2 10252.8 486.424 1

-1 3 -2 10640.0 485.501 2

-1 -3 -2 10276.9 484.838 2

-1 3 -3 26107.4 1178.24 2

-1 -3 -3 25279.7 1177.39 2

-1 3 -3 25796.0 1170.38 5

-1 -3 -3 25407.7 1170.25 5

-1 -3 -3 24748.8 1177.92 1

-1 3 -3 25593.8 1179.28 1

-1 3 -3 24345.6 1175.92 13

-1 -3 -4 212335. 9547.50 2

-1 3 -4 205657. 9547.35 2

-1 -3 -4 206756. 9537.42 5

-1 3 -4 211233. 9537.84 5

-1 -3 -4 210724. 9548.24 1

-1 3 -4 201697. 9548.30 1

-1 3 -4 207049. 9542.87 13

-1 -3 -5 22228.8 1088.99 1

-1 3 -5 22764.2 1090.39 1

-1 3 -5 22705.4 1089.66 2

-1 -3 -5 23027.8 1089.15 2

-1 3 -5 24256.1 1076.99 5

-1 -3 -5 23129.7 1076.03 5

-1 3 -6 115.131 30.2493 5

-1 -3 -6 124.725 31.0904 5

-1 3 -6 138.646 60.1068 2

-1 -3 -6 101.755 60.4895 2

-1 3 -6 178.160 62.0299 1

-1 -3 -6 128.916 66.4375 1

-1 -3 -7 11906.0 598.654 2

-1 3 -7 12189.6 599.324 2

-1 3 -7 12654.6 579.240 5

-1 -3 -7 12569.6 578.716 5

1 -3 7 11463.2 568.971 11

-1 3 -8 190922. 9147.47 2

-1 -3 -8 196767. 9148.65 2

-1 -3 -8 197906. 9125.42 5

-1 3 -8 205295. 9126.51 5

-1 3 -9 9353.24 527.081 2

-1 -3 -9 9658.65 531.176 2

-1 -3 -9 10543.5 488.113 15

-1 -3 -9 9411.97 501.449 5

-1 3 -9 9947.60 503.038 5

-1 -3 -10 3783.35 207.855 15

-1 -3 -10 4227.18 261.617 2

-1 3 -10 4228.90 264.828 2

-1 -3 -10 4167.21 227.337 5

-1 3 -10 4791.57 232.005 5

-1 -3 -10 3864.54 208.410 21

-1 -3 -10 4345.76 235.592 17

-1 3 -11 81.8160 85.1646 23

-1 -3 -11 47.4656 45.9637 5

-1 3 -11 47.6038 47.6364 5

-1 -3 -11 53.4145 44.5227 21

-1 -3 -11 11.7699 77.0470 17

-1 -3 -11 54.0787 39.5823 15

-1 3 -11-21.5292 91.3843 2

-1 -3 -11 64.4094 87.9270 2

-1 3 -11 92.1702 31.9098 10

-1 -3 -11 73.9363 31.5951 10

-1 3 -11 63.0176 30.7387 8

-1 -3 -11 76.7638 29.3037 8

-1 3 -11 85.2898 97.1911 28

-1 -3 -11 78.9638 55.1609 26

-1 -3 -11 89.4985 44.3284 7

-1 3 -11 17.3926 41.5658 7

-1 3 -12 23774.5 1085.55 28

-1 -3 -12 20812.7 1026.91 15

-1 -3 -12 22515.0 1040.13 26

-1 3 -12 20347.3 1053.99 5

-1 -3 -12 19898.2 1050.66 5

-1 -3 -12 22527.7 1047.10 23

-1 3 -12 23756.5 1073.67 23

-1 3 -12 22866.4 1052.00 17

-1 -3 -12 22398.0 1033.38 7

-1 3 -12 21689.5 1024.13 8

-1 -3 -12 21823.7 1023.64 8

-1 3 -12 20841.7 1030.97 7

-1 -3 -12 22613.7 1025.28 10

-1 3 -12 23100.1 1025.94 10

-1 3 -12 20260.3 1093.11 2

-1 -3 -12 21314.8 1096.10 2

-1 3 -13 7034.29 388.409 28

-1 -3 -13 5974.80 315.584 15

-1 -3 -13 6361.80 334.498 26

-1 -3 -13 6404.61 339.851 23

-1 3 -13 6133.09 314.340 24

-1 3 -13 6310.01 324.386 7

-1 3 -13 6268.10 313.464 8

-1 -3 -13 6488.42 325.247 7

-1 -3 -13 6480.68 313.381 8

-1 3 -13 5547.74 389.928 2

-1 -3 -13 6343.58 396.422 2

-1 3 -13 6571.68 348.094 17

-1 -3 -13 6352.08 314.179 10

-1 3 -13 6576.57 315.023 10

-1 -3 -13 6344.56 347.827 5

-1 3 -13 6070.10 347.392 5

-1 3 -14 560.774 153.058 28

-1 3 -14 587.266 85.2617 5

-1 -3 -14 403.912 79.8918 5

-1 -3 -14 536.836 93.3730 23

-1 3 -14 519.497 59.1497 24

-1 -3 -14 599.395 59.2791 8

-1 3 -14 518.546 56.1974 8

-1 -3 -14 527.759 60.8114 15

-1 -3 -14 551.402 143.585 2

-1 3 -14 518.056 133.289 2

-1 -3 -14 711.986 59.6877 10

-1 3 -14 628.026 57.7411 10

-1 3 -14 717.552 118.993 17

-1 3 -15 103.146 108.964 17

-1 -3 -15 23.3389 119.558 2

-1 3 -15 34.8470 122.423 2

-1 3 -15 31.6659 62.0120 5

-1 -3 -15 6.35270 58.8052 5

-1 -3 -15 122.962 98.4137 28

-1 -3 -15 97.7745 87.3139 23

-1 3 -15 49.3242 41.7184 24

-1 -3 -15 70.0020 41.4575 8

-1 3 -15 15.7669 40.9760 8

-1 3 -16 1384.87 153.341 17

-1 -3 -16 1429.03 192.614 2

-1 3 -16 1106.11 189.432 2

-1 -3 -16 1221.42 124.517 5

-1 3 -16 1109.30 123.777 5

-1 3 -16 1163.82 85.6923 24

-1 -3 -16 1230.39 129.164 23

-1 -3 -16 1230.87 90.3170 8

-1 3 -16 1281.64 91.3486 8

-1 -3 -16 1506.38 144.503 28

-1 3 -17 1929.71 111.999 8

-1 -3 -17 1712.46 111.029 8

-1 -3 -17 1335.61 140.171 23

-1 3 -17 1657.54 165.508 17

-1 -3 -17 1378.21 155.024 28

-1 3 -18 1260.00 88.6817 8

-1 -3 -18 1078.32 86.7646 8

-1 -3 -18 1063.28 118.706 23

-1 3 -18 1400.20 150.527 17

-1 -3 -18 1295.74 151.967 28

-1 -3 -19 1906.37 120.408 8

-1 3 -19 2123.40 122.813 8

-1 -3 -19 1836.47 170.580 28

-1 3 -20 82.8517 41.4447 8

-1 -3 -20 46.1820 42.2474 8

-1 -3 -20 31.1328 98.8017 28

-1 3 -21 410.792 49.0297 8

-1 -3 -21 394.735 47.4270 8

1 5 -21 298.871 39.3252 8

1 5 -20 471.045 49.9615 8

1 -5 -20 366.703 101.932 28

1 -5 -19-69.4127 100.190 28

1 -5 -19 31.8675 74.2137 23

1 5 -19 53.2243 40.7102 8

1 5 -18 2.12639 40.9686 8

1 -5 -18 84.4315 40.1072 8

1 -5 -18 40.2443 107.261 28

1 5 -18 75.9959 44.4182 22

1 -5 -18-4.26131 79.6979 23

1 5 -18 192.683 99.4194 17

1 5 -17 3913.57 254.254 17

1 5 -17 3699.03 214.049 24

1 -5 -17 3971.00 248.912 23

1 -5 -17 4166.80 215.423 8

1 5 -17 4391.33 217.387 8

1 -5 -17 4673.28 266.636 28

1 5 -16 4323.06 273.024 5

1 -5 -16 4318.89 272.753 5

1 5 -16 4396.63 330.546 2

1 5 -16 4579.70 272.928 17

1 5 -16 4074.76 232.175 24

1 -5 -16 4335.00 266.804 23

1 -5 -16 4570.69 232.097 8

1 5 -16 4870.62 233.785 8

1 -5 -15 3688.97 223.839 26

1 -5 -15 3691.94 230.752 5

1 5 -15 3434.71 228.105 5

1 -5 -15 3466.60 287.410 2

1 5 -15 3250.12 279.086 2

1 5 -15 3648.89 233.257 17

1 5 -15 3363.22 193.449 24

1 -5 -15 3663.75 228.951 23

1 5 -15 3778.02 191.538 8

1 -5 -15 3504.81 189.343 8

1 -5 -14 134.226 77.5856 26

1 -5 -14 190.454 62.2802 5

1 5 -14 118.714 59.3835 5

1 5 -14 72.6338 109.038 2

1 -5 -14 103.628 124.813 2

1 5 -14 107.868 88.6742 17

1 -5 -14 113.380 44.3034 18

1 -5 -14 216.241 42.7908 8

1 5 -14 158.331 42.1646 8

1 -5 -14 54.8133 82.2182 23

1 5 -14 114.139 48.1033 24

1 -5 -13 9561.12 470.284 18

1 -5 -13 9111.58 473.444 15

1 5 -13 9271.18 470.593 24

1 -5 -13 10017.8 498.216 23

1 -5 -13 9595.59 501.620 5

1 5 -13 9629.99 502.085 5

1 -5 -13 9542.75 485.568 26

1 5 -13 9638.74 497.057 17

1 -5 -13 10050.1 477.465 7

1 5 -13 9533.20 477.014 7

1 5 -13 10061.1 466.203 8

1 -5 -13 10134.5 466.098 8

1 -5 -13 9648.97 553.043 2

1 5 -13 9405.94 550.486 2

1 -5 -12 5899.41 302.606 18

1 -5 -12 6054.68 296.055 8

1 -5 -12 6315.54 308.138 7

1 5 -12 5904.78 306.230 7

1 5 -12 6194.72 296.634 8

1 5 -12 5856.09 322.706 17

1 -5 -12 5848.47 306.027 15

1 5 -12 5913.59 302.440 24

1 5 -12 6345.61 329.866 5

1 -5 -12 5916.30 326.820 5

1 -5 -12 5909.51 313.773 26

1 -5 -12 6122.21 312.336 21

1 -5 -12 6329.40 377.461 2

1 5 -12 6270.43 375.597 2

1 -5 -11 11756.2 566.417 18

1 5 -11 12021.1 559.360 8

1 -5 -11 12399.4 569.035 7

1 5 -11 11230.1 566.553 7

1 -5 -11 11456.9 558.260 8

1 5 -11 11852.8 585.031 17

1 -5 -11 12606.2 570.479 15

1 5 -11 12038.0 565.908 24

1 5 -11 12200.5 589.342 5

1 -5 -11 11504.4 586.318 5

1 -5 -11 11972.6 574.047 26

1 -5 -11 11818.3 570.867 21

1 5 -11 11379.8 569.820 9

1 -5 -11 11954.2 570.546 9

1 5 -11 11341.6 623.565 2

1 -5 -11 11702.4 626.300 2

1 -5 -10 4705.08 251.929 15

1 5 -10 5117.16 249.210 24

1 -5 -10 4767.73 255.290 26

1 5 -10 4874.47 264.430 17

1 5 -10 4927.78 302.578 2

1 -5 -10 5199.19 302.854 2

1 -5 -10 4672.56 251.717 21

1 -5 -10 5384.66 266.891 5

1 5 -10 5561.98 270.096 5

1 -5 -10 4702.58 248.919 18

1 -5 -9 27002.1 1236.78 21

1 5 -9 26166.1 1248.55 5

1 -5 -9 25821.6 1247.41 5

1 5 -9 27317.1 1247.12 17

1 5 -9 25058.5 1273.75 2

1 -5 -9 25224.1 1273.02 2

1 -5 -9 27246.9 1237.18 15

1 -5 -8 2906.31 170.694 21

1 5 -8 3724.08 182.099 5

1 -5 -8 3443.74 178.456 5

1 -5 -8 3080.33 170.679 15

1 -5 -8 3577.27 210.109 2

1 5 -8 3323.95 206.683 2

1 5 -8 3085.32 179.692 17

1 -5 -7 66894.9 3014.36 15

1 -5 -7 66168.4 3013.55 21

1 -5 -7 63262.6 3018.25 5

1 5 -7 68141.7 3020.56 5

1 5 -7 63780.7 3039.17 2

1 -5 -7 65356.6 3038.92 2

1 5 -7 64275.7 3018.95 17

1 5 -7 61747.1 3025.43 13

-1 5 7 67035.6 3015.71 13

1 -5 -6 23796.7 1077.68 5

1 5 -6 24846.2 1079.18 5

1 -5 -6 21600.6 1092.01 2

1 5 -6 21984.2 1093.15 2

-1 5 6 24075.6 1080.25 13

1 5 -6 20911.0 1083.55 13

1 -5 -5 302.410 31.3600 5

1 5 -5 403.362 35.4491 5

1 -5 -5 285.652 54.8530 2

1 5 -5 280.993 51.1308 2

-1 5 5 168.120 36.3739 13

1 5 -5 168.035 43.0603 13

1 5 -4 2050.94 100.634 5

1 -5 -4 1876.81 98.5798 5

1 -5 -4 1794.93 112.094 2

1 5 -4 1757.07 111.548 2

1 5 -4 1804.97 112.912 13

-1 5 4 1800.20 109.399 13

1 5 -3 2526.66 126.757 5

1 -5 -3 2513.17 125.978 5

-1 5 3 2749.84 141.716 13

1 -5 -3 2487.68 134.586 12

-1 5 2 33153.6 1492.52 13

1 -5 -2 31140.8 1491.19 12

-1 5 1 17262.7 771.001 13

1 5 -1 16974.4 774.386 13

1 5 0 50086.8 2224.82 13

-1 5 0 46318.3 2220.32 13

-1 -5 -1 21515.1 1016.26 12

-1 5 -1 23483.0 1032.52 13

-1 5 -2 7884.11 357.331 13

-1 -5 -2 7465.42 336.069 5

-1 5 -2 7481.07 336.284 5

-1 -5 -2 6791.39 339.550 2

-1 5 -2 6686.47 339.666 2

-1 -5 -3 93198.2 4222.69 5

-1 5 -3 95566.0 4223.09 5

-1 -5 -3 88935.6 4228.97 2

-1 5 -3 87370.0 4229.15 2

-1 5 -4 60403.1 2661.94 5

-1 -5 -4 59526.3 2661.50 5

-1 5 -4 57033.8 2674.41 13

-1 5 -4 55275.0 2670.65 2

-1 -5 -4 54239.3 2669.63 2

-1 5 -5 95896.5 4228.56 5

-1 -5 -5 93013.9 4227.36 5

-1 5 -5 89248.8 4239.06 13

-1 -5 -5 90125.7 4239.72 2

-1 5 -5 88449.4 4239.64 2

-1 5 -6 107.512 40.2810 13

-1 -5 -6 132.486 30.7587 5

-1 5 -6 104.674 30.0301 5

-1 -5 -6 157.274 56.2820 2

-1 5 -6 180.016 58.7729 2

-1 5 -7 33004.4 1694.51 2

-1 -5 -7 34343.6 1695.28 2

-1 -5 -7 36825.1 1672.35 15

-1 5 -7 37942.0 1682.48 17

-1 -5 -7 37785.4 1672.85 21

-1 -5 -7 34981.6 1676.85 5

-1 5 -7 35515.8 1677.79 5

-1 5 -8 40416.8 1898.60 5

-1 -5 -8 39709.4 1897.79 5

-1 -5 -8 41019.8 1890.88 15

-1 5 -8 42501.9 1902.35 17

-1 -5 -8 39700.9 1920.65 2

-1 5 -8 37976.5 1919.43 2

-1 -5 -8 42421.0 1891.27 21

-1 5 -9 8582.63 416.193 5

-1 -5 -9 7913.13 413.877 5

-1 -5 -9 8194.63 405.178 15

-1 5 -9 8601.32 420.632 17

-1 -5 -9 7764.38 439.572 2

-1 5 -9 7986.53 442.301 2

-1 -5 -9 8732.83 405.580 21

-1 -5 -10 6914.14 364.934 5

-1 5 -10 7645.57 370.406 5

-1 5 -10 7336.25 371.442 17

-1 -5 -10 7106.13 354.130 15

-1 -5 -10 7165.89 353.567 21

-1 5 -10 6699.13 396.338 2

-1 -5 -10 6860.88 399.044 2

-1 -5 -11 13325.8 613.983 8

-1 5 -11 13216.6 613.996 8

-1 -5 -11 13020.9 629.806 26

-1 5 -11 13072.4 643.035 17

-1 -5 -11 12812.0 624.721 15

-1 5 -11 11571.9 673.425 2

-1 -5 -11 11980.0 676.072 2

-1 5 -11 12728.8 641.993 5

-1 -5 -11 12728.2 640.855 5

-1 -5 -11 12706.0 624.764 21

-1 -5 -11 13381.5 614.695 10

-1 5 -11 13221.4 615.000 10

-1 -5 -12 2712.50 164.138 18

-1 -5 -12 3096.76 158.911 8

-1 5 -12 3076.23 159.279 8

-1 -5 -12 2880.63 179.434 26

-1 -5 -12 2868.39 187.313 5

-1 5 -12 2822.14 187.864 5

-1 5 -12 2989.77 201.459 17

-1 -5 -12 3017.59 171.548 15

-1 5 -12 2837.77 227.824 2

-1 -5 -12 3258.16 241.384 2

-1 5 -12 2976.95 166.209 24

-1 -5 -12 2938.33 183.973 23

-1 -5 -12 3069.59 174.577 21

-1 5 -12 3149.90 159.726 10

-1 -5 -12 3060.52 159.225 10

-1 -5 -13 7854.68 403.773 15

-1 5 -13 7810.09 479.179 2

-1 -5 -13 7967.24 477.698 2

-1 -5 -13 8207.58 393.540 8

-1 5 -13 8331.45 394.640 8

-1 -5 -13 8534.61 419.887 23

-1 5 -13 8166.74 401.402 24

-1 -5 -13 8151.47 428.869 5

-1 5 -13 8159.60 430.710 5

-1 5 -13 8580.03 434.269 17

-1 -5 -13 7616.86 397.782 18

-1 -5 -13 8006.75 415.796 26

-1 5 -14 6435.90 409.518 2

-1 -5 -14 6755.78 412.124 2

-1 5 -14 6985.43 310.155 8

-1 -5 -14 6483.02 307.629 8

-1 5 -14 6115.56 313.345 24

-1 -5 -14 5802.30 330.329 23

-1 -5 -14 6102.74 345.170 5

-1 5 -14 6241.76 347.676 5

-1 -5 -14 6108.86 316.382 15

-1 -5 -14 5453.65 307.801 18

-1 -5 -14 5729.99 331.378 26

-1 -5 -15 140.761 66.0680 5

-1 5 -15 79.7999 59.2304 5

-1 -5 -15 94.4524 41.6308 8

-1 5 -15 103.480 40.6011 8

-1 -5 -15 87.2623 50.8131 15

-1 5 -15 53.6340 46.2756 24

-1 -5 -15 78.8310 77.7678 23

-1 -5 -15-91.1978 126.628 2

-1 5 -15 79.5182 115.865 2

-1 5 -16 203.574 46.6313 8

-1 -5 -16 218.082 45.8430 8

-1 5 -16 252.777 51.5474 24

-1 -5 -16 246.416 79.9600 23

-1 -5 -16 189.733 99.7105 28

-1 -5 -17 774.308 68.2898 8

-1 5 -17 772.200 69.1001 8

-1 -5 -17 925.964 100.770 23

-1 5 -17 761.717 70.4805 24

-1 -5 -17 1018.04 122.961 28

-1 5 -18 816.899 66.3106 8

-1 -5 -18 691.247 63.6934 8

-1 -5 -18 587.174 85.3408 23

-1 -5 -18 776.734 116.534 28

-1 -5 -19 115.366 92.6471 28

-1 5 -19 101.334 40.1466 8

-1 -5 -20-14.1402 83.6036 28

-1 5 -20 6.27281 34.2064 8

1 -7 -20 231.007 77.5198 28

1 -7 -19 350.309 91.1557 28

1 7 -18 7.65785 78.9204 17

1 7 -18 41.4033 43.3256 22

1 -7 -18 4.12700 90.9781 28

1 -7 -18-65.1277 64.3816 23

1 7 -17 2403.64 168.273 17

1 -7 -17 2376.95 176.397 28

1 -7 -17 2255.74 157.507 23

1 7 -17 2325.43 137.434 24

1 -7 -16 461.344 90.4302 23

1 7 -16 556.605 66.0715 24

1 7 -16 578.753 98.7363 17

1 -7 -15 2356.68 168.434 26

1 -7 -15 2182.15 165.519 23

1 7 -15 2424.90 142.780 24

1 7 -15 2458.29 175.739 17

1 -7 -15 2305.66 143.165 18

1 -7 -14 11.3714 73.2982 26

1 -7 -14 51.7565 75.0292 23

1 7 -14 52.9535 46.8319 24

1 -7 -14 1.89266 32.4862 8

1 -7 -14-22.2948 48.6467 18

1 7 -14 131.386 90.2726 17

1 7 -13 4225.04 244.374 17

1 7 -13 4120.72 218.568 24

1 -7 -13 4061.04 232.644 26

1 -7 -13 4164.16 220.483 18

1 -7 -13 3710.24 214.986 20

1 7 -12 7359.20 447.975 2

1 7 -12 7735.64 404.398 17

1 -7 -12 8181.53 397.042 21

1 7 -12 7401.34 384.414 24

1 -7 -12 7901.55 391.428 15

1 -7 -12 7781.68 387.829 18

1 -7 -11 11380.5 537.040 21

1 7 -11 10508.0 585.014 2

1 -7 -11 10961.0 546.841 5

1 7 -11 11227.1 547.426 17

1 -7 -11 10957.8 535.066 15

1 -7 -11 10498.6 529.810 18

1 -7 -10 187.396 50.4784 15

1 -7 -10 104.443 64.6349 25

1 -7 -10 110.450 41.6767 5

1 7 -10 93.3397 77.8829 2

1 -7 -10 163.284 48.2477 18

1 7 -10 209.989 68.8479 17

1 -7 -10 141.055 57.9223 21

1 -7 -10 133.263 57.6908 26

1 -7 -9 14575.4 695.273 15

1 -7 -9 15347.9 696.020 21

1 7 -9 15128.6 690.507 24

1 -7 -9 13830.3 698.876 5

1 7 -9 13514.6 727.372 2

1 7 -9 14307.6 701.399 17

1 -7 -9 14129.9 691.303 18

1 -7 -9 13976.8 701.232 25

1 -7 -9 15275.9 696.496 26

1 7 -8 7713.71 433.492 13

1 7 -8 7091.20 437.405 2

1 -7 -8 7838.98 417.126 5

1 -7 -8 9590.57 420.034 26

1 7 -8 8763.47 423.801 17

1 -7 -8 8773.00 418.465 21

1 7 -8 8921.49 412.740 24

1 7 -7 8228.50 424.699 17

-1 7 7 8925.22 426.250 13

1 7 -7 8029.09 436.731 13

1 -7 -7 9117.98 422.045 5

1 -7 -6 439.471 51.9704 15

1 7 -6 455.184 59.4638 17

-1 7 6 301.710 47.9065 13

1 7 -6 269.047 56.1476 13

1 -7 -6 530.855 56.3761 21

1 -7 -5 2256.61 131.705 21

-1 7 5 2457.69 142.142 13

1 7 -5 2395.25 147.838 13

1 -7 -5 2227.02 130.042 15

1 -7 -4 3872.88 194.254 21

-1 7 4 3837.19 205.988 13

1 7 -4 3522.65 209.316 13

1 -7 -4 3691.61 192.245 15

1 -7 -3 11993.9 511.518 21

-1 7 3 11444.0 527.474 13

1 -7 -3 10551.8 509.432 15

1 -7 -2 13938.3 687.795 12

-1 7 2 15534.4 701.494 13

1 7 -2 14574.2 703.974 13

-1 7 1 4308.86 229.034 13

1 -7 -1 4196.53 228.088 12

1 7 -1 4125.15 230.282 13

-1 -7 0 124.741 31.2849 12

-1 7 0 73.2472 43.9041 13

1 7 0 163.084 49.4636 13

1 7 1 69260.0 3128.33 13

-1 7 -1 65950.9 3123.54 13

1 7 2 1791.66 120.961 13

-1 -7 -2 1959.37 105.165 21

-1 -7 -3 49822.2 2299.95 21

1 7 3 50944.0 2320.50 13

-1 -7 -3 49350.5 2298.78 15

-1 -7 -4 622.501 58.9080 21

1 7 4 513.181 67.1631 13

-1 -7 -4 608.739 56.2222 15

1 7 5 119189. 5191.89 13

-1 7 -5 107135. 5188.65 13

-1 -7 -5 112061. 5171.87 15

-1 -7 -6 7899.83 373.695 15

-1 7 -6 7486.85 367.730 16

-1 7 -6 7123.54 379.479 17

-1 7 -6 7360.95 385.641 2

-1 7 -6 7384.81 390.094 13

1 7 6 8019.62 389.757 13

-1 -7 -6 7909.63 369.210 5

1 7 7 5984.08 294.047 13

-1 -7 -7 5491.58 279.805 21

-1 7 -7 5295.97 298.613 2

-1 7 -7 5389.60 278.572 16

-1 -7 -7 5679.54 280.720 15

-1 7 -7 5302.59 289.035 17

-1 -7 -7 5612.87 278.161 5

-1 -7 -8 14228.1 676.846 21

-1 -7 -8 14382.4 678.922 5

-1 7 -8 13267.6 701.867 2

-1 -7 -8 14858.3 677.098 26

-1 7 -8 13723.8 685.635 17

-1 7 -8 14075.0 672.184 24

-1 7 -9 21257.4 1022.78 17

-1 7 -9 20117.0 1044.52 2

-1 -7 -9 21756.7 1012.49 21

-1 -7 -9 20560.9 1011.10 15

-1 -7 -9 22401.1 1013.14 26

-1 -7 -9 21124.4 1016.28 5

-1 7 -9 22064.8 1007.73 24

-1 7 -10 363.068 82.9193 17

-1 -7 -10 354.666 53.1010 5

-1 -7 -10 270.328 56.3610 15

-1 7 -10 305.983 88.3996 2

-1 -7 -10 371.229 61.6359 26

-1 -7 -10 330.063 62.8722 21

-1 7 -11 4938.22 281.522 17

-1 7 -11 4495.59 309.642 2

-1 -7 -11 4878.93 259.613 18

-1 -7 -11 4967.90 272.976 5

-1 -7 -11 5210.20 267.701 26

-1 -7 -11 4915.66 263.003 15

-1 -7 -11 5006.88 265.690 21

-1 -7 -12 1983.84 129.765 18

-1 7 -12 2042.31 129.096 24

-1 7 -12 2053.04 161.454 17

-1 -7 -12 2206.23 136.727 15

-1 -7 -12 1882.62 138.616 21

-1 -7 -13 2524.11 151.747 18

-1 7 -13 2583.91 150.246 24

-1 -7 -13 2203.07 158.273 23

-1 -7 -13 2443.77 135.781 8

-1 7 -13 2492.69 182.490 17

-1 -7 -13 2476.26 156.385 15

-1 -7 -13 2740.94 166.252 21

-1 -7 -14 77.7624 51.3360 18

-1 7 -14 172.619 96.2056 17

-1 -7 -14 109.184 36.8123 8

-1 -7 -14 100.162 59.7349 15

-1 -7 -14 78.4149 65.4585 23

-1 7 -14 169.511 51.7371 24

-1 -7 -15 80.8691 66.1655 23

-1 7 -15 93.9851 50.2506 24

-1 7 -15 149.257 96.5676 17

-1 7 -16 389.813 58.1973 24

-1 -7 -16 349.090 94.1923 28

-1 7 -16 327.161 103.016 17

-1 7 -17 41.4832 44.3448 24

-1 -7 -17 28.1331 81.4494 28

-1 7 -17 4.37752 89.3702 17

-1 -7 -18 294.419 87.3283 28

-1 7 -18 296.415 90.8344 17

-1 -7 -19 46.5784 72.0514 28

1 -9 -19 51.4581 59.8900 28

1 9 -19 60.0742 41.0157 22

1 -9 -18 248.059 78.1509 28

1 9 -18 258.620 47.6034 22

1 -9 -17 284.817 82.5234 28

1 9 -17 315.675 52.5178 24

1 9 -16 448.232 80.1408 17

1 -9 -16 628.308 98.1530 28

1 9 -16 558.818 64.0843 24

1 9 -15 146.567 75.1394 17

1 -9 -15 88.8330 55.2016 18

1 9 -15 15.5899 47.3900 24

1 -9 -15 90.7130 66.9619 23

1 9 -14 588.881 89.5442 17

1 -9 -14 609.256 74.7035 18

1 -9 -14 502.899 83.8821 23

1 -9 -13 244.718 64.4321 18

1 9 -13 268.391 79.6832 17

1 -9 -13 206.872 58.0605 20

1 -9 -13 260.847 81.9727 26

1 9 -12 4078.36 228.419 24

1 -9 -12 4527.32 245.293 26

1 9 -12 3980.15 242.963 17

1 -9 -12 4493.11 246.046 21

1 -9 -12 4379.76 236.638 18

1 9 -11 3218.12 181.587 24

1 -9 -11 3417.10 195.998 26

1 9 -11 3098.05 194.917 17

1 -9 -11 3200.59 193.885 21

1 -9 -11 3028.24 185.314 18

1 9 -10 3908.11 212.693 24

1 -9 -10 3760.80 224.445 25

1 -9 -10 4369.61 227.694 26

1 9 -10 3785.69 224.933 17

1 -9 -10 4243.40 226.096 21

1 -9 -10 3790.49 217.746 18

1 9 -9 904.126 94.1694 17

1 -9 -9 916.233 96.5462 25

1 -9 -9 1054.42 92.6046 26

1 9 -9 1045.30 79.6798 24

1 -9 -9 967.073 89.1127 18

1 9 -8 1388.34 110.397 17

1 -9 -8 1584.27 110.411 26

1 -9 -8 1489.05 117.770 25

1 9 -8 1432.47 98.4347 24

1 9 -7 1464.36 132.981 13

1 -9 -7 1851.73 121.948 21

1 9 -7 1787.25 110.088 24

1 9 -7 1587.29 109.011 16

1 -9 -7 1685.86 118.272 15

1 9 -7 1524.74 116.667 17

1 -9 -7 1849.52 132.296 25

1 -9 -7 2213.22 122.908 26

1 -9 -7 1847.40 116.160 18

1 -9 -6 1516.28 112.167 21

1 9 -6 1863.73 103.812 24

1 9 -6 1704.15 102.246 16

1 9 -6 1424.97 108.131 17

1 9 -6 1544.72 130.456 13

-1 9 6 1730.91 120.090 13

1 -9 -6 1638.07 108.647 18

1 -9 -5 1725.24 110.920 21

1 9 -5 1522.51 106.129 17

1 9 -5 1692.77 98.4243 24

1 9 -5 1424.16 124.647 13

-1 9 5 1528.89 117.199 13

1 -9 -5 1652.73 104.678 18

-1 9 4 23.6055 50.2752 13

1 -9 -4 115.012 47.2452 21

1 -9 -4 14.1943 42.1486 18

1 -9 -3 8385.43 388.725 21

-1 9 3 7440.11 407.323 13

1 -9 -3 7749.05 385.678 18

1 9 -2 31.4699 58.2909 13

-1 9 2 40.0487 47.1444 13

-1 -9 2-13.3168 40.8149 21

1 -9 -2-5.59334 40.1016 21

1 -9 -2 31.9970 36.2015 18

-1 -9 1 5985.11 295.457 21

1 -9 -1 6377.85 296.590 21

1 -9 -1 5839.68 292.887 18

1 9 -1 5395.00 321.236 13

-1 9 1 5902.61 320.759 13

-1 -9 0 25205.8 1148.71 21

1 -9 0 24438.3 1145.58 21

1 -9 0 23303.7 1143.62 18

1 9 0 26595.7 1182.37 13

-1 9 0 25880.5 1176.73 13

1 -9 1 9233.27 454.512 21

-1 -9 -1 9923.70 460.607 21

1 -9 1 8591.48 490.930 12

1 9 1 11035.2 496.182 13

-1 9 -1 10610.7 490.786 13

1 -9 1 9130.67 452.971 18

-1 -9 -2 2705.29 146.817 15

1 9 2 2837.03 173.601 13

-1 9 -2 2616.28 169.971 13

-1 -9 -2 2386.82 146.653 21

-1 -9 -3 152.135 46.7906 15

1 9 3 227.668 58.1371 13

-1 9 -3 239.564 59.3296 13

-1 -9 -3 189.742 47.3005 21

-1 -9 -4 11428.2 536.440 15

-1 -9 -4 10840.2 534.678 21

1 9 4 10955.1 557.330 13

-1 9 -5 10070.6 487.000 24

-1 -9 -5 10439.7 496.591 15

-1 9 -5 10031.9 486.814 16

-1 -9 -5 10601.7 491.487 26

-1 -9 -5 9622.53 492.528 21

-1 9 -5 10134.0 520.350 13

1 9 5 10219.7 514.368 13

-1 -9 -6 837.770 78.9206 21

-1 9 -6 934.794 67.9555 24

-1 9 -6 783.587 69.1015 16

-1 -9 -6 703.039 75.8643 15

-1 9 -6 721.877 82.5553 17

-1 -9 -6 1087.23 78.2289 26

-1 9 -6 622.981 85.6849 13

1 9 6 805.718 84.3390 13

-1 9 -7 1473.69 92.1010 24

-1 -9 -7 1100.72 95.8385 15

-1 9 -7 1190.27 94.4706 16

-1 9 -7 1293.92 105.056 17

-1 -9 -7 1436.67 99.1358 26

1 9 7 1177.77 101.211 13

-1 9 -8 6211.72 353.360 17

-1 -9 -8 7184.87 346.607 26

-1 -9 -8 7045.23 349.215 15

-1 9 -8 6586.58 352.249 16

-1 9 -8 6870.59 341.493 24

-1 9 -9 1505.21 100.506 24

-1 -9 -9 1472.40 111.369 15

-1 9 -9 1205.46 118.355 17

-1 -9 -9 1378.05 113.816 21

-1 -9 -9 1404.78 109.353 26

-1 -9 -10 145.399 62.3404 18

-1 9 -10 160.541 82.6893 17

-1 -9 -10 204.228 68.6115 26

-1 9 -10 196.568 51.2431 24

-1 -9 -10 229.251 71.7228 21

-1 -9 -11 2410.97 152.720 18

-1 9 -11 2407.02 147.019 24

-1 -9 -11 2313.35 155.696 15

-1 9 -11 2489.00 169.950 17

-1 -9 -11 2447.54 160.989 21

-1 -9 -11 2381.07 157.775 26

-1 9 -12 446.950 92.5165 17

-1 -9 -12 289.475 66.6987 18

-1 -9 -12 367.541 80.8825 26

-1 -9 -12 323.017 79.7091 21

-1 9 -12 311.705 60.8657 24

-1 -9 -13 113.487 56.1418 18

-1 9 -13 205.211 84.9481 17

-1 -9 -13 190.554 79.5887 26

-1 -9 -13 262.046 76.9975 21

-1 -9 -14 117.451 80.2614 26

-1 9 -14 16.7758 80.2390 17

-1 -9 -14 32.4616 56.0885 18

-1 9 -15 64.9955 51.1093 24

-1 -9 -15 29.1933 70.2417 28

-1 9 -15 119.770 81.2526 17

-1 9 -16 9.58676 46.0904 24

-1 -9 -16 6.71985 70.7323 28

-1 9 -16-14.6778 76.4448 17

-1 9 -17 459.377 57.4056 24

-1 -9 -17 445.824 77.7046 28

-1 9 -17 477.124 83.5857 17

-1 9 -18 14.4099 40.6383 24

-1 -9 -18 33.4185 61.2070 28

-1 9 -18 47.6165 40.6952 22

1 11 -17 84.6028 41.6701 22

1 -11 -16 257.494 64.6895 28

1 11 -16 193.618 46.1241 22

1 11 -15 336.110 58.0578 24

1 -11 -15 223.121 73.4854 28

1 -11 -14 1127.78 95.9455 18

1 -11 -14 1141.93 113.428 26

1 11 -14 1101.63 89.9894 24

1 -11 -13 1604.44 120.965 18

1 -11 -13 1877.49 138.779 26

1 11 -13 1683.45 117.667 24

1 11 -12 2827.41 164.120 24

1 -11 -12 2791.13 180.380 26

1 -11 -12 2786.78 171.347 18

1 -11 -12 2821.42 181.019 21

1 -11 -12 2773.32 167.866 20

1 -11 -11 131.369 65.4406 18

1 -11 -11 201.805 75.3036 26

1 11 -11 119.606 51.9885 24

1 -11 -11 180.062 73.9163 21

1 -11 -10 1492.25 116.349 26

1 -11 -10 1109.56 103.311 25

1 11 -10 1327.40 99.2982 24

1 11 -9 1174.19 103.449 17

1 11 -9 1505.97 103.799 24

1 -11 -9 1422.43 120.835 21

1 -11 -9 1394.60 114.948 25

1 -11 -9 1624.43 117.896 26

1 11 -8 2792.15 158.455 24

1 -11 -8 3016.47 171.275 26

1 -11 -8 2410.81 167.497 25

1 -11 -8 2587.02 163.873 18

1 -11 -8 2550.69 167.437 21

1 11 -7 1300.43 93.8517 24

1 11 -7 1261.23 103.769 16

1 -11 -7 1462.06 109.483 26

1 -11 -7 1139.18 112.396 25

1 -11 -7 1300.98 106.374 18

1 -11 -7 1409.23 110.718 21

1 -11 -6 8032.96 419.592 21

1 11 -6 8345.32 412.367 24

1 11 -6 8336.46 416.361 16

1 -11 -6 7978.82 431.576 25

1 -11 -6 9189.97 421.400 26

1 11 -6 8092.65 455.968 13

1 -11 -6 8407.59 419.465 18

1 -11 -5 2088.02 137.247 21

1 11 -5 2218.05 126.555 24

1 -11 -5 2383.03 137.845 26

1 11 -5 2169.55 172.209 13

-1 11 5 1791.92 146.079 13

1 -11 -5 1986.55 134.226 18

1 -11 -4 151.967 51.6929 18

1 11 -4 181.205 42.1321 24

-1 11 4 144.078 56.7265 13

1 -11 -4 108.498 59.5422 21

1 11 -3 213.329 70.8596 13

-1 11 3 235.113 67.2222 13

1 -11 -3 217.698 59.4367 21

1 -11 -3 213.955 54.0902 18

-1 -11 2 2326.74 143.377 21

1 -11 -2 2674.72 150.308 21

1 -11 -2 2644.17 149.891 18

-1 11 2 2843.85 182.002 13

1 11 -2 2634.34 189.040 13

1 -11 -1 1018.28 82.0220 21

-1 -11 1 1012.22 79.6486 21

-1 -11 1 1073.15 77.1017 18

1 -11 -1 1048.02 80.4740 18

1 11 -1 745.590 103.572 13

-1 11 1 1012.61 106.543 13

1 -11 0 6271.99 328.018 21

-1 -11 0 6374.68 331.246 21

1 11 0 7725.50 378.468 13

-1 11 0 7286.33 370.070 13

1 -11 0 6546.58 329.314 18

-1 -11 0 6727.78 330.188 18

1 11 1 92.7315 72.5467 13

-1 11 -1 117.280 61.4542 13

1 -11 1 110.564 41.1185 21

-1 -11 -1 122.518 51.8138 21

-1 -11 -1 117.427 45.9558 18

-1 -11 -2 7644.40 388.373 21

1 11 2 8101.48 425.707 13

-1 11 -2 7918.46 423.159 13

-1 -11 -2 7799.78 386.685 18

-1 -11 -3 468.515 59.8566 26

-1 -11 -3 479.525 66.0598 18

1 11 3 438.320 78.6864 13

-1 11 -3 503.200 87.8239 13

-1 -11 -3 471.073 69.5040 21

-1 -11 -4 3452.66 211.515 21

-1 11 -4 3854.41 201.643 24

-1 11 -4 4006.96 208.281 16

-1 -11 -4 4147.75 208.442 26

-1 -11 -4 3576.05 209.231 18

1 11 4 3816.56 236.185 13

-1 -11 -5 178.036 62.8050 21

-1 11 -5 265.421 46.6529 24

-1 11 -5 280.267 57.2793 16

-1 -11 -5 228.935 58.8143 18

-1 11 -5 186.420 58.3024 17

-1 -11 -5 261.307 58.7994 26

1 11 5 199.645 64.3082 13

1 11 6 1753.59 139.122 13

-1 -11 -6 1903.37 129.052 18

-1 11 -6 1939.54 117.650 24

-1 11 -6 1897.92 127.241 16

-1 11 -6 1507.83 125.112 17

-1 -11 -6 2081.48 126.301 26

-1 -11 -6 1789.07 130.758 21

-1 11 -7 82.8420 47.6652 24

-1 11 -7 92.2267 64.0872 16

-1 -11 -7 24.5889 60.1994 18

-1 11 -7-22.5278 60.2028 17

-1 -11 -7 52.1514 59.1131 26

-1 -11 -7 61.7510 68.9265 21

-1 -11 -8 4163.39 229.200 18

-1 11 -8 4257.54 222.827 24

-1 11 -8 3806.65 240.476 16

-1 11 -8 3721.39 228.385 17

-1 -11 -8 3835.19 232.066 21

-1 -11 -8 4597.58 230.287 26

-1 11 -9 345.041 73.8437 17

-1 -11 -9 379.843 74.6949 26

-1 11 -9 434.871 62.8896 24

-1 11 -10 576.220 81.7966 17

-1 -11 -10 517.135 79.6307 26

-1 -11 -10 404.661 79.0878 21

-1 11 -10 358.566 60.4071 24

-1 11 -11 379.214 78.9930 17

-1 -11 -11 263.395 70.3624 18

-1 -11 -11 381.323 79.9038 26

-1 -11 -11 367.536 83.2903 21

-1 11 -11 449.501 66.2147 24

-1 -11 -12 107.110 61.2120 18

-1 -11 -12 94.3022 72.2936 26

-1 -11 -12 113.903 72.6648 21

-1 11 -12 123.032 52.8219 24

-1 11 -13 128.214 56.7431 24

-1 -11 -13 160.264 79.3994 26

-1 -11 -13 212.182 60.5056 18

-1 11 -14 325.872 59.2593 24

-1 -11 -14 233.325 64.4114 28

-1 -11 -14 254.657 62.9781 18

-1 11 -15 192.412 53.9278 24

1 13 -15 51.4018 36.8251 22

1 13 -14 842.817 66.4875 22

1 -13 -13 251.061 71.9914 26

1 -13 -12 855.389 83.2868 18

1 -13 -12 684.535 77.7922 20

1 -13 -12 665.006 87.8709 26

1 -13 -11 451.809 69.0599 20

1 -13 -11 546.079 81.0616 26

1 13 -11 510.922 66.5526 24

1 -13 -10 1418.77 114.099 18

1 -13 -10 1565.08 118.663 26

1 -13 -10 1354.11 118.336 21

1 13 -10 1538.80 108.405 24

1 -13 -9 1001.53 95.3301 18

1 -13 -9 1061.35 99.3298 26

1 -13 -9 1080.50 106.139 21

1 13 -9 1208.87 93.2913 24

1 -13 -8 305.740 72.2181 18

1 -13 -8 188.325 69.7585 21

1 13 -8 214.993 56.8594 24

1 -13 -8 260.844 70.9277 26

1 -13 -7 238.998 71.3556 18

1 -13 -7 211.009 67.6547 26

1 -13 -7 252.295 71.1634 21

1 13 -7 246.463 58.7234 24

1 13 -6 1975.43 128.074 24

1 -13 -6 2101.38 134.514 26

1 -13 -6 1847.74 131.850 21

1 -13 -6 1843.79 134.741 18

1 13 -6 2035.30 142.081 16

1 13 -5-31.3039 71.9735 13

1 -13 -5 34.5629 60.1010 18

1 -13 -5 54.0239 54.7666 26

1 -13 -5 5.98088 60.9789 21

1 13 -5 44.7216 60.0519 16

-1 13 4 742.854 94.0612 13

1 -13 -4 559.716 76.1606 18

1 -13 -4 809.961 77.2552 26

1 -13 -4 628.061 78.8869 21

1 13 -4 669.910 80.6306 16

1 -13 -3-2.98200 52.1333 18

1 -13 -3-24.8936 58.2287 21

1 -13 -3 28.2655 51.8081 26

1 13 -3-15.1152 69.9148 13

-1 13 3-17.9836 59.8983 13

-1 13 2 799.635 102.933 13

1 13 -2 814.684 113.327 13

1 -13 -2 764.716 76.8810 21

1 -13 -2 823.905 80.5126 18

1 -13 -1 25.2189 49.5082 18

-1 -13 1 12.1331 47.0843 21

-1 13 1 64.8316 62.1061 13

1 13 -1 108.516 71.9396 13

1 -13 -1 34.8773 50.8232 21

-1 -13 0 183.063 54.4782 18

-1 -13 0 176.393 54.2275 21

-1 13 0 288.580 80.7447 13

1 13 0 294.737 82.8781 13

1 -13 0 213.917 51.4917 21

-1 13 -1 48.0162 66.7745 13

1 13 1-34.2278 69.1013 13

-1 -13 -1 30.2166 54.2688 21

-1 -13 -1 25.4398 50.0789 18

1 13 2 1361.38 129.816 13

-1 13 -2 1383.78 131.242 13

-1 -13 -2 1188.40 93.7341 18

1 13 3 882.697 106.216 13

-1 -13 -3 866.529 91.0959 21

-1 13 -3 1070.05 92.5800 16

-1 -13 -3 841.162 87.8475 18

-1 -13 -4 549.039 78.8994 21

1 13 4 351.466 79.7182 13

-1 13 -4 563.332 78.0522 16

-1 -13 -4 594.019 76.4660 18

-1 -13 -4 580.262 67.5492 26

-1 -13 -5 700.051 87.4308 21

-1 13 -5 766.131 88.3087 16

-1 13 -5 775.913 72.7525 24

-1 -13 -5 651.492 81.3734 18

-1 -13 -5 729.504 77.1382 26

-1 -13 -6 158.341 58.2716 26

-1 13 -6 56.3499 70.2415 16

-1 -13 -6 124.113 64.7636 18

-1 -13 -6 215.171 73.1343 21

-1 13 -6 89.1973 50.0948 24

-1 -13 -7 46.6057 66.4055 21

-1 13 -7 47.9928 50.3966 24

-1 -13 -7 107.726 58.9056 26

-1 -13 -7 16.4744 61.9137 18

-1 13 -8 2128.32 129.630 24

-1 -13 -8 1859.70 136.231 18

-1 -13 -8 2152.70 136.200 26

-1 -13 -8 1779.92 140.567 21

-1 -13 -9 77.8330 72.9887 21

-1 -13 -9 102.897 64.9325 26

-1 -13 -9 41.7863 61.4457 18

-1 13 -9 33.8898 51.0633 24

-1 -13 -10 73.3826 67.1295 26

-1 -13 -10 46.8439 63.5397 18

-1 13 -10 16.6594 49.9308 24

-1 -13 -11 34.6799 64.6091 26

-1 13 -11 53.5090 51.5696 24

-1 -13 -11 56.1135 71.9550 21

-1 -13 -12 236.117 62.7781 18

-1 13 -12 228.544 54.4406 24

-1 -13 -13 20.5071 55.0125 18

-1 13 -13 11.8166 46.7498 24

-1 13 -15 68.1855 35.8847 22

1 -15 -10 178.423 57.7823 20

1 -15 -9 939.975 88.2058 20

1 -15 -9 1148.37 90.1764 26

-1 15 9 1217.15 82.2381 16

1 -15 -8 501.220 74.0804 18

1 -15 -8 356.718 62.8536 26

-1 15 8 449.350 57.7883 16

1 -15 -7 170.919 62.9854 18

1 -15 -7 362.447 68.9440 21

1 -15 -7 287.348 58.7170 26

1 -15 -6 1246.04 97.1142 26

1 -15 -6 1497.29 111.334 18

1 -15 -6 1430.46 106.945 21

1 -15 -5 197.011 62.5383 21

1 -15 -5 273.051 69.3203 18

1 -15 -4 239.613 60.4964 21

1 -15 -4 262.284 67.3850 18

1 15 -4 294.357 79.3800 16

1 -15 -3 152.062 56.1063 21

1 15 -3 169.885 72.4553 16

1 -15 -2 2250.97 147.120 21

1 15 -2 3122.51 172.744 16

1 15 -1 151.644 65.2640 16

-1 15 0 76.2557 62.2558 16

-1 -15 -1 258.173 56.7611 21

-1 15 -1 209.727 70.9499 16

-1 -15 -2 1173.00 96.3741 21

-1 15 -2 1510.83 117.280 16

-1 -15 -2 1260.78 104.868 18

-1 -15 -3 126.184 62.3810 18

-1 15 -3 216.390 76.2839 16

-1 -15 -3 139.080 58.9364 21

-1 -15 -4 91.6501 62.9662 18

-1 15 -4 49.1182 76.5580 16

-1 -15 -4 30.7569 55.6497 21

-1 -15 -5 102.474 63.4795 21

-1 -15 -5 153.874 63.9783 18

-1 -15 -6 111.442 62.9042 21

-1 -15 -6 129.888 63.1383 18

-1 -15 -7 193.162 65.6142 21

-1 -15 -7 111.588 61.4170 18

-1 -15 -8 47.8822 59.9779 21

-1 -15 -8 81.4591 59.5327 18

-1 -15 -9 17.3077 51.7343 20

-1 -15 -9 83.2340 58.2441 18

-1 -15 -10 63.6410 52.6107 20

-1 -15 -10 38.9694 56.4139 18

1 -17 -5 128.783 54.5593 20

-1 17 5 129.243 53.7841 16

1 -17 -4 139.118 56.8960 20

-1 17 4 208.138 63.0516 16

1 -17 -3 815.570 85.3403 20

-1 17 3 1498.12 102.985 16

1 -17 -2 46.1300 54.6389 20

-1 17 1 798.370 91.7245 16

1 17 -1 661.115 95.2229 16

-1 17 0 85.3834 72.9198 16

1 17 0 19.7495 71.2940 16

-1 17 -1 1051.36 109.588 16

1 17 1 949.139 96.8087 16

-1 -17 -2-6.82652 49.0087 20

1 17 2 85.3314 62.4532 16

-1 -17 -3 61.8007 53.2627 20

-1 -17 -4 45.3658 52.7192 20

1 17 4 112.741 60.0288 16

1 17 5 350.759 62.5034 16

2 0 -22 172.000 43.9521 8

2 0 -21 21.7849 43.0242 8

2 0 -20 461.674 59.8625 8

2 0 -19 28.8987 48.8256 8

2 0 -19 93.1773 132.818 28

2 0 -18 1060.20 82.5558 8

2 0 -18 692.483 166.375 28

2 0 -18 834.048 144.490 23

2 0 -17-113.018 118.498 23

2 0 -17 85.0849 70.5707 5

2 0 -17 52.4206 143.731 2

2 0 -17 10.0339 45.0709 8

2 0 -17-14.8015 135.331 28

2 0 -16 407.830 135.369 28

2 0 -16 587.648 95.1457 5

2 0 -16 444.936 153.274 2

2 0 -16 292.405 135.892 23

2 0 -16 496.683 60.1435 8

2 0 -16 386.095 75.7370 7

2 0 -15 31.1128 54.3942 7

2 0 -15-10.1454 59.5160 9

2 0 -15-6.47736 113.430 28

2 0 -15 12.6798 62.0434 5

2 0 -15-35.6198 135.057 2

2 0 -15-52.2232 117.683 23

2 0 -15-14.5945 101.942 17

2 0 -15 20.3144 38.9994 8

2 0 -14 12310.4 611.570 7

2 0 -14 11744.4 612.820 9

2 0 -14 13165.5 652.049 28

2 0 -14 13175.6 656.592 23

2 0 -14 12305.6 630.798 17

2 0 -14 12659.2 697.468 2

2 0 -14 13267.4 604.787 8

2 0 -14 13007.3 646.358 5

2 0 -13 7.66236 44.4589 7

2 0 -13 44.1683 49.3227 9

2 0 -13 15.1010 55.2155 5

2 0 -13 46.8269 107.793 2

2 0 -13 113.685 97.7600 23

2 0 -13 56.9930 78.4253 17

2 0 -13 42.3332 35.4336 8

2 0 -12 57492.2 2749.96 17

2 0 -12 56672.1 2737.32 7

2 0 -12 55661.6 2774.41 4

2 0 -12 63476.2 2766.71 5

2 0 -12 60036.4 2806.58 2

2 0 -12 59845.6 2769.71 23

2 0 -12 63163.1 2733.20 10

2 0 -12 55065.0 2745.76 27

2 0 -12 62995.6 2732.55 8

2 0 -12 55623.6 2739.00 9

2 0 -11 30.9530 44.7321 5

2 0 -11 28.1624 90.4669 2

2 0 -11 88.7913 62.0972 17

2 0 -11 5.09224 61.9164 4

2 0 -10 873.285 119.595 2

2 0 -10 999.052 65.5303 6

2 0 -10 978.556 86.0792 17

2 0 -10 858.911 96.5490 4

2 0 -10 1048.82 85.0792 5

2 0 -9 35.6144 76.0045 2

2 0 -9 48.5579 49.8515 17

2 0 -9 34.5568 26.9723 6

2 0 -9 2.81685 38.2677 5

2 0 -9-3.85478 52.9329 4

2 0 -8 352752. 15706.2 5

2 0 -8 336995. 15715.8 4

2 0 -8 332104. 15725.8 2

2 0 -7 39.7044 57.5027 2

2 0 -7 35.7862 28.5473 5

2 0 -7 2.79926 40.3741 4

2 0 -6 15978.4 721.291 5

2 0 -6 14884.0 727.931 4

2 0 -6 14750.8 736.099 2

2 0 -5-16.7940 48.9196 2

2 0 -4 545106. 24973.3 2

2 0 -3 43.7359 26.3689 11

2 0 -3 53.4501 37.7468 2

2 0 -2 50060.7 2144.64 3

2 0 -2 51870.1 2146.34 12

2 0 -2 48716.5 2148.13 2

2 0 -1 80.7655 21.0812 12

2 0 -1 218.894 20.2948 3

2 0 0 50093.5 2320.54 11

2 0 1 60.8384 49.7885 11

2 0 2 36752.8 1963.08 11

-2 0 -2 48300.6 1963.29 1

2 0 3-19.1915 36.9491 11

-2 0 -3 77.5197 41.4186 2

-2 0 -3 85.7821 47.6063 1

-2 0 -4 63552.0 2976.60 1

-2 0 -4 65214.1 2974.21 2

-2 0 -5 23.9249 52.1576 2

-2 0 -5 145.354 56.2554 1

-2 0 -6 10946.1 530.841 1

-2 0 -6 10399.0 527.575 2

-2 0 -7 130.649 64.1003 2

-2 0 -7 69.4039 71.1497 1

-2 0 -7 6.47505 30.6108 5

-2 0 -8 132055. 6125.31 1

-2 0 -8 132413. 6099.46 5

-2 0 -8 133424. 6123.30 2

-2 0 -9 55.4234 45.0796 5

-2 0 -9 50.3240 85.8013 2

-2 0 -9 17.1006 89.1542 1

-2 0 -9 29.5551 57.3183 17

-2 0 -10 9396.87 440.034 17

-2 0 -10 8230.47 441.576 5

-2 0 -10 8533.51 480.229 2

-2 0 -11 52.1063 69.0729 28

-2 0 -11 29.3411 31.8532 10

-2 0 -11 35.8786 30.5466 8

-2 0 -11 15.0450 97.2410 2

-2 0 -11-23.2746 69.6802 17

-2 0 -11 4.41077 39.9566 7

-2 0 -11 4.16167 45.8338 5

-2 0 -12 22272.1 1084.30 23

-2 0 -12 20821.0 1128.13 2

-2 0 -12 23794.8 1059.95 10

-2 0 -12 22157.8 1082.55 17

-2 0 -12 22158.4 1089.96 5

-2 0 -12 21626.8 1065.20 7

-2 0 -12 23433.5 1058.46 8

-2 0 -12 22390.2 1086.98 28

-2 0 -13 16.2302 37.2265 8

-2 0 -13 134.116 121.549 2

-2 0 -13 14.6667 91.8001 28

-2 0 -13-45.6239 82.1321 23

-2 0 -13-8.77146 89.0978 17

-2 0 -13 165.513 61.9014 7

-2 0 -13 34.0750 37.2594 10

-2 0 -13-5.29921 55.8609 5

-2 0 -14 782.236 97.2691 5

-2 0 -14 582.190 155.166 2

-2 0 -14 781.609 69.8963 10

-2 0 -14 928.380 128.287 17

-2 0 -14 860.505 125.518 28

-2 0 -14 845.601 70.1900 8

-2 0 -14 699.414 113.618 23

-2 0 -15 62.7642 90.5218 23

-2 0 -15-37.7593 106.415 17

-2 0 -15-47.7215 137.827 2

-2 0 -15 14.8670 44.3207 10

-2 0 -15 45.8282 68.0364 5

-2 0 -15-2.38725 41.4588 8

-2 0 -16 13207.5 611.521 8

-2 0 -16 12397.6 719.925 2

-2 0 -16 13466.2 654.364 17

-2 0 -16 12389.3 658.390 5

-2 0 -16 11320.5 640.154 23

-2 0 -17 35.5031 48.0391 8

-2 0 -17-18.0395 110.430 17

-2 0 -18 14.8740 50.1734 8

-2 0 -19 69.6743 47.8038 8

-2 0 -20 1802.28 112.069 8

-2 0 -21 10.4173 38.0758 8

-2 0 -22 52.8045 32.4849 8

2 -2 -22-27.7462 36.8246 8

2 2 -22-1.54699 36.4922 8

2 -2 -21 39.7829 39.8120 8

2 2 -21 18.9154 40.5684 8

2 2 -20 299.830 53.6471 8

2 -2 -20 302.795 52.7070 8

2 -2 -19 2269.69 189.350 23

2 -2 -19 2416.93 139.889 8

2 2 -19 2331.84 139.563 8

2 -2 -18 284.528 118.546 23

2 2 -18 308.598 55.7689 8

2 -2 -18 316.533 55.8110 8

2 2 -17 1855.53 228.328 28

2 -2 -17 2350.06 134.384 8

2 2 -17 2345.64 133.896 8

2 2 -17 2221.82 237.280 2

2 -2 -17 2166.11 240.179 2

2 -2 -17 1783.97 179.534 23

2 -2 -17 1901.88 167.734 5

2 2 -17 2094.73 170.811 5

2 2 -17 2013.95 181.578 17

2 2 -16 951.031 175.172 28

2 2 -16 1186.19 179.002 23

2 -2 -16 1158.49 151.031 23

2 2 -16 1190.51 105.828 7

2 -2 -16 1261.96 105.826 7

2 -2 -16 1399.49 93.2861 8

2 2 -16 1285.76 91.4341 8

2 -2 -16 1246.58 186.268 2

2 2 -16 1256.39 186.334 2

2 2 -16 1096.95 123.424 5

2 -2 -16 1123.90 125.282 5

2 2 -16 1188.28 139.786 17

2 2 -15-4.65609 97.5669 17

2 -2 -15 37.4469 56.4326 7

2 2 -15 25.1041 56.4823 7

2 2 -15-9.37240 34.1088 24

2 -2 -15 140.053 104.870 23

2 2 -15 82.8604 132.566 23

2 2 -15 46.9425 133.081 28

2 -2 -15-64.7056 60.0318 9

2 2 -15 37.5270 63.5875 9

2 2 -15-58.5853 125.719 2

2 -2 -15-23.4391 135.391 2

2 -2 -15 15.6346 42.1762 8

2 2 -15 6.71264 37.4527 8

2 -2 -15-37.7974 67.2918 5

2 2 -15-12.6176 67.0892 5

2 2 -14 190.203 57.8057 7

2 -2 -14 169.323 56.6895 7

2 -2 -14 155.953 59.7013 9

2 2 -14 124.819 59.8110 9

2 2 -14 203.906 123.230 28

2 2 -14 200.452 38.5955 24

2 -2 -14 85.8930 95.4905 23

2 2 -14 170.387 119.579 23

2 2 -14 224.380 125.607 2

2 -2 -14 138.658 120.196 2

2 -2 -14 251.406 70.9318 5

2 2 -14 229.255 71.6158 5

2 -2 -14 222.882 43.3280 8

2 2 -14 192.850 45.3023 8

2 2 -13 15677.6 773.785 7

2 -2 -13 16136.5 774.510 7

2 -2 -13 15799.4 783.305 26

2 2 -13 15551.2 776.058 9

2 -2 -13 16030.5 776.819 9

2 2 -13 16310.9 821.812 28

2 -2 -13 16077.7 784.848 27

2 2 -13 14763.9 762.523 24

2 2 -13 18125.6 830.089 23

2 -2 -13 17055.0 805.251 23

2 2 -13 16554.8 852.943 2

2 -2 -13 17669.1 858.823 2

2 2 -13 16924.2 805.919 5

2 -2 -13 16721.8 804.881 5

2 2 -13 17111.3 767.586 8

2 -2 -13 17409.0 767.677 8

2 -2 -12 29717.0 1416.59 17

2 -2 -12 29543.5 1398.81 7

2 2 -12 28237.1 1397.68 7

2 -2 -12 28658.3 1406.78 27

2 2 -12 29868.2 1436.47 28

2 -2 -12 31353.6 1392.14 8

2 2 -12 31751.7 1392.72 8

2 -2 -12 28793.9 1400.61 9

2 2 -12 28231.3 1399.68 9

2 2 -12 31897.6 1444.04 23

2 -2 -12 30787.5 1426.23 23

2 -2 -12 28931.9 1404.50 26

2 2 -12 32075.9 1427.06 5

2 -2 -12 31265.6 1425.56 5

2 -2 -12 30493.5 1468.06 2

2 2 -12 30573.7 1468.36 2

2 -2 -11 1871.79 165.078 2

2 2 -11 1836.80 161.464 2

2 -2 -11 1126.98 82.2872 15

2 -2 -11 1200.58 112.354 17

2 -2 -11 1656.18 113.990 5

2 2 -11 2003.41 118.199 5

2 -2 -11 1425.60 128.187 4

2 2 -11 1324.01 125.408 4

2 -2 -10 2329.85 148.332 17

2 2 -10 2639.98 189.197 2

2 -2 -10 2371.24 182.970 2

2 2 -10 2323.13 147.528 5

2 -2 -10 2460.63 164.771 4

2 2 -10 2104.08 159.859 4

2 -2 -10 2269.32 144.006 5

2 -2 -9 73960.8 3304.50 17

2 2 -9 71450.8 3299.85 17

2 2 -9 66126.9 3317.49 4

2 -2 -9 75236.3 3308.84 5

2 -2 -9 69410.3 3319.23 4

2 2 -9 73337.7 3309.60 5

2 2 -9 70902.0 3289.27 6

2 -2 -9 71477.4 3289.32 6

2 2 -9 70530.0 3335.50 2

2 -2 -9 71566.5 3335.30 2

2 2 -8 12594.6 638.543 4

2 2 -8 14601.6 631.532 5

2 -2 -8 13209.6 628.199 5

2 -2 -8 12460.4 637.823 4

2 -2 -8 12966.6 652.163 2

2 2 -8 12179.0 648.946 2

2 2 -7 76413.7 3264.72 5

2 -2 -7 76126.3 3263.92 5

2 2 -7 73213.8 3273.46 4

2 -2 -7 73263.8 3273.70 4

2 -2 -7 62847.5 3252.26 11

2 -2 -7 71322.9 3281.00 2

2 2 -7 72503.5 3282.71 2

2 -2 -6 1691.92 95.8573 5

2 2 -6 1816.87 97.5782 5

2 -2 -6 1700.60 107.472 4

2 2 -6 1776.76 108.484 4

2 -2 -6 1591.90 85.9223 11

2 2 -6 1585.25 90.5904 13

2 2 -6 1651.58 117.434 2

2 -2 -6 1451.56 113.423 2

2 2 -5 100127. 4666.05 4

2 -2 -5 102227. 4666.55 4

2 -2 -5 98561.0 4658.78 5

2 2 -5 101734. 4659.88 5

2 2 -5 93893.4 4655.46 13

-2 2 5 106217. 4648.40 13

2 2 -5 97430.5 4671.31 2

2 -2 -5 99001.0 4671.01 2

2 -2 -4 70593.6 3346.49 5

2 2 -4 72605.3 3352.28 4

2 2 -4 74149.4 3347.29 5

2 -2 -4 72967.4 3352.60 4

-2 2 4 76707.2 3340.35 13

2 2 -4 69273.2 3345.91 13

2 -2 -4 68995.8 3355.48 2

2 2 -4 70744.2 3356.78 2

2 2 -3 133844. 6125.45 4

2 -2 -3 136646. 6125.81 4

2 2 -3 132274. 6121.47 5

2 -2 -3 129469. 6121.01 5

2 2 -3 121681. 6127.92 2

2 -2 -3 128442. 6128.64 2

-2 2 3 144204. 6118.74 13

2 2 -3 131486. 6123.75 13

2 -2 -3 132811. 6128.67 12

2 2 -2 82159.6 3711.27 4

2 2 -2 80594.4 3707.91 5

2 -2 -2 77921.0 3707.49 5

2 -2 -2 83209.6 3711.29 4

2 2 -2 79880.7 3709.71 3

2 -2 -2 80686.2 3709.84 3

2 -2 -2 82161.3 3720.17 12

-2 2 2 86900.4 3708.13 13

2 -2 -2 76451.2 3713.27 2

2 2 -2 77060.1 3713.60 2

-2 2 1 70855.9 3244.96 13

2 -2 -1 68514.0 3244.53 3

2 2 -1 73044.1 3245.48 3

-2 2 0 78472.2 3601.14 13

-2 2 -1 182295. 8355.22 13

-2 2 -2 26665.0 1235.08 1

-2 -2 -2 26573.4 1234.54 1

-2 2 -3 119481. 5522.20 2

-2 -2 -3 123371. 5522.54 2

-2 -2 -3 121233. 5523.65 1

-2 2 -3 116717. 5523.38 1

-2 -2 -4 82112.7 3707.26 2

-2 2 -4 78669.6 3706.47 2

-2 2 -4 79473.9 3709.55 1

-2 -2 -4 81531.0 3709.33 1

-2 2 -5 3972.14 226.348 2

-2 -2 -5 4074.56 226.377 2

-2 -2 -5 4379.96 230.392 1

-2 2 -5 3932.57 227.891 1

2 -2 5 4441.51 209.362 11

-2 -2 -6 11537.0 578.692 5

-2 2 -6 12491.2 580.072 5

-2 -2 -6 11858.3 595.666 2

-2 2 -6 11520.4 595.042 2

-2 2 -6 12109.1 598.036 1

-2 -2 -6 12031.1 597.558 1

2 -2 6 13543.2 586.793 11

-2 2 -7 10501.2 566.633 1

-2 -2 -7 11203.3 568.911 1

-2 -2 -7 11454.4 569.457 2

-2 2 -7 10560.6 565.693 2

-2 2 -7 10578.9 545.358 5

-2 -2 -7 11644.4 547.136 5

-2 2 -8 73086.3 3325.29 17

-2 2 -8 70755.2 3352.66 2

-2 -2 -8 72873.3 3354.01 2

-2 2 -8 68813.6 3352.87 1

-2 -2 -8 73733.6 3355.88 1

-2 -2 -8 71260.1 3327.47 5

-2 2 -8 72846.3 3328.44 5

-2 2 -9 630.213 104.873 2

-2 -2 -9 617.936 103.014 2

-2 2 -9 685.496 70.6829 5

-2 -2 -9 865.180 75.1553 5

-2 -2 -9 736.586 82.5967 17

-2 2 -9 651.226 77.4100 17

-2 -2 -10 34711.3 1598.44 5

-2 2 -10 34075.7 1598.51 5

-2 2 -10 32629.2 1589.97 17

-2 -2 -10 35273.6 1593.20 17

-2 -2 -10 33833.1 1630.32 2

-2 2 -10 33016.4 1629.85 2

-2 2 -11 16560.4 799.744 17

-2 -2 -11 17578.3 803.783 17

-2 -2 -11 15281.0 776.937 15

-2 2 -11 15294.2 839.746 2

-2 -2 -11 16165.0 844.662 2

-2 2 -11 16791.3 777.951 10

-2 -2 -11 17062.0 777.845 10

-2 2 -11 16417.2 805.925 5

-2 -2 -11 15898.1 803.258 5

-2 -2 -11 16473.5 790.487 26

-2 2 -11 15690.5 782.411 7

-2 -2 -11 16504.0 784.012 7

-2 2 -11 16702.8 776.717 8

-2 -2 -11 17444.5 777.084 8

-2 2 -11 16810.6 809.684 23

-2 2 -11 17436.6 815.532 28

-2 -2 -12 1806.04 117.415 15

-2 2 -12 1935.65 191.020 2

-2 -2 -12 1802.11 186.931 2

-2 2 -12 2168.94 119.051 10

-2 -2 -12 2221.83 118.715 10

-2 -2 -12 2143.32 117.747 8

-2 -2 -12 2057.92 130.354 7

-2 2 -12 2139.87 117.184 8

-2 2 -12 2014.77 129.285 7

-2 -2 -12 1714.26 139.451 23

-2 2 -12 2005.33 158.682 23

-2 2 -12 1857.80 149.539 17

-2 -2 -12 2065.00 153.892 17

-2 2 -12 1880.90 169.869 28

-2 -2 -12 2134.79 148.500 5

-2 2 -12 1933.43 143.505 5

-2 -2 -13 2530.76 155.911 15

-2 -2 -13 2795.16 238.044 2

-2 2 -13 2816.44 237.999 2

-2 -2 -13 3100.52 159.789 10

-2 2 -13 3259.04 160.989 10

-2 2 -13 2957.08 196.252 17

-2 -2 -13 3058.00 202.361 17

-2 2 -13 2975.24 203.063 23

-2 -2 -13 2624.25 183.976 23

-2 -2 -13 2649.94 189.595 5

-2 2 -13 2777.49 189.544 5

-2 -2 -13 2848.36 158.509 8

-2 2 -13 2878.77 157.599 8

-2 2 -13 2960.96 219.870 28

-2 -2 -13 2928.25 190.782 28

-2 2 -14 2601.67 232.191 2

-2 -2 -14 2441.80 231.765 2

-2 -2 -14 2453.77 140.839 10

-2 2 -14 2389.07 140.571 10

-2 -2 -14 2544.43 195.579 17

-2 2 -14 2878.58 193.990 17

-2 -2 -14 2328.97 170.476 23

-2 2 -14 2071.03 135.913 24

-2 -2 -14 2561.40 179.400 5

-2 2 -14 2385.05 176.641 5

-2 2 -14 2588.10 141.948 8

-2 -2 -14 2666.22 143.090 8

-2 -2 -14 2409.79 181.948 28

-2 2 -14 2362.39 206.719 28

-2 -2 -15 4474.89 333.826 2

-2 2 -15 4170.40 327.434 2

-2 2 -15 4584.04 228.848 10

-2 -2 -15 4498.79 227.938 10

-2 -2 -15 3983.84 255.984 23

-2 2 -15 3782.97 220.829 24

-2 2 -15 4639.77 272.077 5

-2 -2 -15 3810.85 262.808 5

-2 -2 -15 4073.35 267.610 28

-2 2 -15 4455.50 300.871 28

-2 2 -15 4720.67 229.070 8

-2 -2 -15 4398.07 228.407 8

-2 2 -15 4491.87 274.255 17

-2 2 -16 265.578 145.350 2

-2 -2 -16 508.547 153.262 2

-2 2 -16 257.335 46.0291 24

-2 -2 -16 339.656 94.1997 23

-2 -2 -16 353.349 84.3612 5

-2 2 -16 413.835 87.9425 5

-2 2 -16 433.914 58.8788 8

-2 -2 -16 465.064 58.2168 8

-2 2 -16 410.759 125.383 17

-2 -2 -16 228.144 115.728 28

-2 -2 -17 466.132 100.521 23

-2 -2 -17 653.378 67.8844 8

-2 2 -17 608.739 66.5472 8

-2 2 -17 809.373 126.924 17

-2 -2 -17 516.526 121.794 28

-2 2 -18 761.305 72.4203 8

-2 -2 -18 689.247 69.5837 8

-2 2 -18 768.889 132.881 17

-2 -2 -18 588.196 126.859 28

-2 -2 -19 1403.75 99.8356 8

-2 2 -19 1601.57 101.853 8

-2 -2 -19 1426.21 159.196 28

-2 2 -20 183.421 47.4699 8

-2 -2 -20 171.684 45.4907 8

-2 -2 -21 593.956 56.5795 8

-2 2 -21 632.141 56.7035 8

-2 2 -22-6.84053 29.2044 8

2 4 -21 105.052 39.0928 8

2 -4 -21 27.7877 94.5766 28

2 -4 -20 194.865 80.7073 23

2 4 -20 112.101 42.1003 8

2 -4 -20 171.313 43.2610 8

2 -4 -20 33.1159 104.703 28

2 -4 -19 82.1223 92.2242 23

2 4 -19 139.652 46.7557 8

2 -4 -19 83.6522 43.2929 8

2 -4 -19 74.2004 111.163 28

2 -4 -18 4267.08 275.875 23

2 -4 -18 4681.68 239.264 8

2 4 -18 4711.77 239.981 8

2 -4 -18 4454.45 292.914 28

2 -4 -17 189.193 102.032 23

2 4 -17 174.099 43.2534 24

2 -4 -17 231.036 120.523 28

2 -4 -17 204.569 46.9084 8

2 4 -17 230.370 50.8998 8

2 4 -17 129.116 102.138 17

2 -4 -16 1418.39 146.026 23

2 4 -16 1232.97 86.6435 24

2 -4 -16 971.684 115.722 5

2 4 -16 1241.40 122.342 5

2 4 -16 1060.09 176.071 2

2 -4 -16 991.771 182.254 2

2 -4 -16 1176.44 86.3897 8

2 4 -16 1167.89 85.0273 8

2 4 -16 1425.05 142.689 17

2 -4 -15 3281.72 222.381 26

2 4 -15 3496.41 196.944 24

2 -4 -15 3889.23 241.447 23

2 -4 -15 3603.31 235.475 5

2 4 -15 4116.05 243.088 5

2 4 -15 3513.09 294.930 2

2 -4 -15 3694.09 297.140 2

2 -4 -15 3674.41 210.453 7

2 4 -15 3895.49 198.589 8

2 -4 -15 3850.76 197.227 8

2 4 -15 3631.87 210.247 7

2 4 -15 3776.51 235.298 17

2 4 -14 9653.76 464.485 17

2 -4 -14 9110.05 471.590 23

2 4 -14 8517.57 432.424 24

2 4 -14 8023.05 519.183 28

2 -4 -14 9386.63 455.735 26

2 4 -14 8756.85 469.935 5

2 -4 -14 8739.89 470.190 5

2 -4 -14 8899.10 442.521 7

2 4 -14 8708.94 442.227 7

2 4 -14 9446.69 432.565 8

2 -4 -14 8628.65 430.499 8

2 -4 -14 8470.88 524.081 2

2 4 -14 8223.12 520.174 2

2 4 -13 218.758 46.1609 24

2 4 -13 253.192 60.8293 9

2 -4 -13 260.558 60.3084 9

2 -4 -13 204.101 92.0392 23

2 4 -13 246.539 67.2906 5

2 -4 -13 233.612 63.9323 5

2 -4 -13 229.531 39.6336 8

2 4 -13 211.993 41.3123 8

2 -4 -13 135.848 42.4398 18

2 4 -13 296.663 85.2358 17

2 -4 -13 171.602 45.8696 15

2 4 -13 190.765 147.240 28

2 -4 -13 188.090 70.4106 26

2 -4 -13 217.614 54.5034 7

2 4 -13 242.009 55.9980 7

2 4 -13 257.647 122.594 2

2 -4 -13 75.8431 116.026 2

2 4 -12 1700.85 102.873 24

2 4 -12 1671.04 111.909 9

2 -4 -12 1660.74 110.865 9

2 -4 -12 1582.77 96.7608 8

2 4 -12 1621.18 98.0749 8

2 4 -12 1654.58 129.262 5

2 -4 -12 1455.80 122.483 5

2 -4 -12 1688.52 102.296 18

2 4 -12 1699.31 132.248 17

2 -4 -12 1621.62 104.570 15

2 4 -12 1544.13 177.500 28

2 -4 -12 2135.86 125.770 27

2 -4 -12 1874.45 118.843 26

2 4 -12 1532.37 108.269 7

2 -4 -12 1680.30 110.074 7

2 -4 -12 1772.89 178.049 2

2 4 -12 1637.75 177.645 2

2 4 -11 19962.1 930.367 24

2 -4 -11 19202.2 935.374 21

2 4 -11 20831.5 926.886 8

2 -4 -11 20080.7 926.151 8

2 -4 -11 18854.8 929.017 18

2 -4 -11 19929.5 955.016 5

2 4 -11 21618.1 958.702 5

2 4 -11 19101.1 944.768 17

2 -4 -11 19853.5 933.047 15

2 -4 -11 21716.4 944.663 27

2 -4 -11 19687.2 993.801 2

2 4 -11 19352.5 991.358 2

2 4 -11 19038.6 934.942 9

2 -4 -11 20034.3 936.590 9

2 -4 -11 19704.2 939.003 26

2 4 -11 19392.0 933.652 7

2 -4 -11 19703.1 933.768 7

2 4 -10 19648.5 952.944 5

2 4 -10 17602.9 961.955 4

2 -4 -10 18278.3 950.093 5

2 -4 -10 18833.9 964.388 4

2 -4 -10 20788.3 933.780 18

2 -4 -10 20665.1 937.671 21

2 4 -10 20999.4 947.677 17

2 4 -10 18841.3 982.941 2

2 -4 -10 18610.5 982.737 2

2 -4 -10 21175.6 936.529 15

2 -4 -9 29197.0 1385.04 5

2 -4 -9 29843.9 1399.72 4

2 4 -9 31093.7 1387.65 5

2 4 -9 28332.8 1397.26 4

2 -4 -9 29061.1 1369.58 18

2 -4 -9 31064.1 1372.74 15

2 4 -9 28974.1 1413.18 2

2 -4 -9 29800.8 1414.90 2

2 -4 -9 28332.4 1371.58 21

2 4 -9 29785.2 1381.05 17

2 4 -8 1698.13 104.137 17

2 -4 -8 1702.55 93.5175 15

2 -4 -8 1755.29 93.9125 21

2 -4 -8 1548.51 114.149 4

2 4 -8 1275.12 96.1915 5

2 4 -8 1496.99 113.150 4

2 -4 -8 1294.79 95.3127 5

2 -4 -8 1397.14 127.540 2

2 4 -8 1212.01 125.986 2

2 -4 -7 84375.1 3930.52 2

2 4 -7 81318.8 3930.41 2

2 4 -7 87015.9 3912.35 5

2 -4 -7 84936.0 3910.96 5

2 -4 -7 87477.9 3922.65 4

2 4 -7 82179.4 3920.48 4

-2 4 7 88322.1 3899.57 13

2 4 -7 78498.5 3911.59 13

2 -4 -6 8243.17 430.863 2

2 4 -6 8869.91 434.264 2

2 4 -6 8957.75 415.742 5

2 -4 -6 8137.45 412.467 5

2 -4 -6 8547.42 422.895 4

2 4 -6 9022.21 424.560 4

-2 4 6 9273.75 408.709 13

2 4 -6 7648.06 414.961 13

2 4 -5 6766.20 358.043 2

2 -4 -5 6848.87 358.410 2

2 -4 -5 7605.19 352.795 4

2 4 -5 6953.95 343.992 5

2 4 -5 7052.49 351.035 4

2 -4 -5 6572.22 342.469 5

2 4 -5 7191.82 350.146 13

-2 4 5 8123.23 344.250 13

2 -4 -4 22623.0 1087.46 2

2 4 -4 21942.3 1087.26 2

2 -4 -4 23022.2 1084.76 12

-2 4 4 25668.0 1080.55 13

2 4 -4 21751.5 1084.02 13

2 -4 -4 22769.0 1076.32 5

2 4 -4 22915.0 1081.74 4

2 4 -4 23239.6 1077.14 5

2 -4 -4 24660.3 1082.84 4

2 4 -3 10041.0 489.762 13

-2 4 3 11046.4 485.235 13

2 -4 -3 10319.8 491.745 12

2 -4 -3 10541.7 482.043 4

2 -4 -3 9678.27 478.085 5

2 4 -3 10080.3 481.723 4

2 4 -3 10225.4 479.187 5

-2 4 2 13387.6 599.243 13

2 -4 -2 12722.6 612.057 12

2 4 -2 12010.3 590.679 4

2 -4 -2 13090.0 591.160 4

2 -4 -1 3668.64 169.687 4

2 4 -1 3350.18 168.877 4

-2 4 0 39073.6 1752.80 13

-2 -4 0 35391.0 1740.18 1

-2 4 0 36055.3 1741.24 1

2 -4 0 38555.2 1739.70 3

2 4 0 41449.1 1741.20 3

-2 4 -1 17662.0 731.991 13

-2 -4 -1 15000.0 720.035 1

-2 4 -1 15475.9 721.238 1

-2 -4 -1 15590.5 719.887 2

-2 4 -1 15222.3 719.921 2

-2 4 -2 85659.0 3847.06 13

-2 4 -2 83738.3 3842.51 1

-2 -4 -2 81458.2 3840.75 1

-2 -4 -2 84858.3 3840.59 2

-2 4 -2 83008.4 3840.83 2

-2 -4 -3 2355.63 130.434 1

-2 4 -3 2372.64 129.654 1

-2 -4 -3 2337.51 127.490 2

-2 4 -3 2255.38 127.663 2

-2 4 -4 7744.76 385.358 1

-2 -4 -4 7035.10 382.284 1

-2 4 -4 7231.32 380.548 13

-2 4 -4 8472.03 372.600 5

-2 -4 -4 8320.37 372.238 5

-2 4 -4 7347.16 381.694 2

-2 -4 -4 7780.19 382.437 2

-2 -4 -5 92527.0 4393.82 2

-2 4 -5 92835.1 4393.91 2

-2 4 -5 96720.2 4381.07 5

-2 -4 -5 99108.5 4381.33 5

-2 4 -5 90841.0 4394.82 1

-2 -4 -5 96141.0 4395.69 1

-2 -4 -6 79423.1 3706.22 2

-2 4 -6 77871.1 3706.36 2

-2 4 -6 77939.3 3707.08 1

-2 -4 -6 79524.1 3706.22 1

-2 4 -6 83131.0 3689.36 5

-2 -4 -6 80291.8 3688.48 5

-2 4 -7 2046.31 123.789 17

2 -4 7 1791.11 108.349 11

-2 4 -7 1935.41 140.697 2

-2 -4 -7 1695.12 133.571 2

-2 4 -7 1939.81 113.044 5

-2 -4 -7 1716.33 110.769 5

-2 -4 -8 313.315 80.2628 2

-2 4 -8 427.744 87.7596 2

-2 -4 -8 266.376 45.7159 21

-2 -4 -8 391.332 51.0502 5

-2 4 -8 431.682 51.7122 5

-2 4 -9 48280.3 2403.31 2

-2 -4 -9 51285.0 2406.14 2

-2 -4 -9 50512.9 2360.72 21

-2 -4 -9 52290.2 2377.83 5

-2 4 -9 52800.5 2378.07 5

-2 -4 -10 39355.3 1865.92 15

-2 -4 -10 40273.7 1918.25 2

-2 4 -10 40190.6 1918.22 2

-2 4 -10 43417.8 1887.43 5

-2 -4 -10 41812.3 1885.33 5

-2 -4 -10 38565.8 1864.31 21

-2 -4 -10 40572.3 1870.23 26

-2 4 -11 133.915 88.6042 2

-2 -4 -11-7.42760 86.5331 2

-2 -4 -11 67.5611 45.4300 15

-2 -4 -11 104.033 52.2601 26

-2 -4 -11 57.4882 45.1627 5

-2 4 -11 61.6838 47.2764 5

-2 -4 -11 98.9193 54.3239 23

-2 4 -11 31.8003 29.6679 10

-2 -4 -11 94.8026 30.7020 10

-2 -4 -11 174.906 31.3635 8

-2 4 -11 27.0261 28.2021 8

-2 -4 -11 92.7688 45.8275 21

-2 4 -12 1534.94 164.973 2

-2 -4 -12 1221.65 160.293 2

-2 -4 -12 1444.66 102.951 15

-2 -4 -12 1528.53 117.632 26

-2 -4 -12 1613.45 119.983 23

-2 4 -12 1614.68 94.7439 10

-2 -4 -12 1651.45 94.6073 10

-2 -4 -12 1550.18 93.4689 8

-2 4 -12 1521.23 93.5614 8

-2 -4 -12 1491.80 105.380 21

-2 4 -12 1300.01 116.779 5

-2 -4 -12 1247.04 116.229 5

-2 4 -13 14372.8 700.855 5

-2 -4 -13 14605.0 700.398 5

-2 4 -13 14869.7 663.008 10

-2 -4 -13 14196.6 661.950 10

-2 -4 -13 13582.0 681.233 23

-2 4 -13 13192.9 663.803 24

-2 -4 -13 14310.8 748.828 2

-2 4 -13 13521.3 746.482 2

-2 -4 -13 12314.0 658.763 18

-2 -4 -13 13154.5 666.421 15

-2 4 -13 14904.3 662.159 8

-2 -4 -13 14685.0 661.445 8

-2 -4 -14 5518.87 327.395 28

-2 -4 -14 6107.47 338.532 5

-2 4 -14 6144.55 339.092 5

-2 -4 -14 5393.86 317.831 23

-2 4 -14 5638.85 300.094 24

-2 -4 -14 6264.99 298.125 8

-2 4 -14 6361.28 299.028 8

-2 -4 -14 5445.10 301.075 15

-2 4 -14 5977.05 341.030 17

-2 4 -14 5541.45 389.475 2

-2 -4 -14 5552.67 390.424 2

-2 4 -14 6047.65 297.960 10

-2 -4 -14 6084.84 298.245 10

-2 -4 -15 343.273 51.2550 8

-2 4 -15 376.181 53.6520 8

-2 -4 -15 465.752 86.8023 23

-2 4 -15 419.753 58.0686 24

-2 4 -15 460.675 122.514 17

-2 -4 -15 522.716 62.6132 15

-2 -4 -15 272.517 77.3411 5

-2 4 -15 322.307 75.2664 5

-2 -4 -15 638.925 107.470 28

-2 4 -15 303.902 139.297 2

-2 -4 -15 411.306 146.420 2

-2 4 -16 1776.65 165.937 17

-2 4 -16 1518.68 204.499 2

-2 -4 -16 1538.59 133.730 23

-2 4 -16 1506.45 105.698 24

-2 -4 -16 1662.93 149.587 28

-2 -4 -16 1750.63 107.763 8

-2 4 -16 1708.15 108.172 8

-2 -4 -16 1559.58 141.705 5

-2 4 -16 1707.55 147.905 5

-2 -4 -17 374.094 81.9739 23

-2 4 -17 346.748 112.249 17

-2 4 -17 312.975 52.4868 8

-2 -4 -17 317.796 51.4809 8

-2 -4 -17 286.221 103.866 28

-2 4 -18 190.339 107.563 17

-2 -4 -18 150.997 96.7367 28

-2 4 -18 112.058 46.0785 8

-2 -4 -18 219.435 46.7077 8

-2 -4 -19 138.056 97.6986 28

-2 4 -19 28.0344 41.1596 8

-2 -4 -19 73.7151 42.2160 8

-2 4 -20 587.389 56.3551 8

-2 4 -21 1.27607 29.0588 8

2 -6 -21 214.249 80.5770 28

2 -6 -20 3.73331 87.8086 28

2 -6 -19 283.336 101.571 28

2 6 -19 266.938 45.1974 8

2 -6 -19 362.101 81.8535 23

2 -6 -18 61.6448 82.2471 23

2 -6 -18 202.789 105.603 28

2 6 -18 90.1477 46.4727 22

2 6 -18 240.398 91.0063 17

2 6 -18 151.223 41.4537 8

2 -6 -17-13.7108 88.5290 23

2 6 -17 37.7715 42.7004 24

2 6 -17 97.5043 86.3183 17

2 6 -17 70.0108 41.0456 8

2 -6 -17 107.870 104.757 28

2 6 -16 176.877 93.3516 17

2 -6 -16 134.509 41.2247 8

2 6 -16 126.031 41.4939 8

2 -6 -16 131.657 92.4206 23

2 6 -16 143.749 49.2261 24

2 -6 -15 2842.57 204.317 5

2 -6 -15 3258.71 206.858 23

2 6 -15 3194.37 175.367 24

2 6 -15 3204.84 204.933 17

2 -6 -15 2685.63 171.583 18

2 6 -15 3302.07 169.020 8

2 -6 -15 3144.94 167.041 8

2 -6 -15 2992.51 197.612 26

2 -6 -14 94.0347 75.9678 26

2 6 -14-22.7086 56.4854 5

2 -6 -14 61.4400 57.2648 5

2 6 -14 83.4843 45.0928 24

2 6 -14 30.1676 86.9223 17

2 -6 -14 10.3837 47.0770 18

2 -6 -14 73.0605 83.7538 23

2 6 -14 63.6945 35.6787 8

2 -6 -14 72.3490 36.8408 8

2 -6 -13 928.111 148.224 2

2 6 -13 696.875 139.447 2

2 -6 -13 1015.81 95.2052 26

2 -6 -13 644.272 87.4840 5

2 6 -13 770.291 92.1450 5

2 6 -13 853.289 75.2023 24

2 6 -13 894.196 104.671 17

2 -6 -13 959.747 82.7193 7

2 -6 -13 760.186 62.4014 8

2 6 -13 814.324 65.0291 8

2 6 -13 1025.97 85.8732 7

2 -6 -13 815.770 75.5170 18

2 -6 -12 642.373 63.3649 18

2 -6 -12 440.388 57.4292 20

2 -6 -12 593.750 50.7231 8

2 6 -12 613.914 65.4480 7

2 6 -12 660.887 53.0210 8

2 6 -12 594.120 89.0165 17

2 -6 -12 524.414 68.4297 15

2 -6 -12 522.208 77.4167 26

2 6 -12 551.330 75.5741 5

2 -6 -12 525.771 72.2801 5

2 6 -12 588.401 61.0937 24

2 -6 -12 741.582 82.1298 21

2 -6 -12 488.657 118.363 2

2 6 -12 526.150 117.942 2

2 -6 -11 1339.64 96.9035 21

2 -6 -11 1260.60 76.7928 8

2 6 -11 1247.34 88.8477 7

2 -6 -11 1300.18 91.1168 18

2 -6 -11 1414.46 101.917 27

2 6 -11 1389.46 112.605 17

2 -6 -11 1252.59 91.8501 15

2 6 -11 1310.35 88.5964 24

2 -6 -11 1091.88 97.6985 5

2 6 -11 1026.06 97.5858 5

2 -6 -11 1438.09 103.444 26

2 6 -11 1166.55 91.0708 9

2 -6 -11 1172.88 142.457 2

2 6 -11 1033.37 138.893 2

2 -6 -10 2714.47 154.247 15

2 6 -10 2617.86 147.810 24

2 -6 -10 2510.59 164.922 25

2 6 -10 2440.60 162.688 17

2 6 -10 2452.33 195.438 2

2 -6 -10 2662.62 198.820 2

2 -6 -10 2552.03 153.000 21

2 6 -10 3185.33 169.965 5

2 -6 -10 2975.39 165.222 5

2 -6 -10 2564.29 149.563 18

2 6 -9 4088.48 203.148 5

2 -6 -9 3774.69 199.518 5

2 6 -9 3295.96 198.530 17

2 -6 -9 3278.29 189.177 21

2 -6 -9 3541.70 191.233 15

2 6 -9 3653.72 233.072 2

2 -6 -9 3997.28 235.716 2

2 -6 -9 3396.05 187.726 18

2 6 -8 364.237 58.8868 13

2 -6 -8 429.516 50.1132 5

2 6 -8 373.750 62.5734 17

2 -6 -8 478.672 57.0062 21

2 -6 -8 483.464 55.2487 15

2 -6 -8 600.709 87.2559 2

2 6 -8 568.313 88.8709 2

2 -6 -8 437.714 52.0554 18

2 6 -7 7775.85 372.432 5

2 -6 -7 7496.45 370.643 5

2 6 -7 6910.33 379.887 13

-2 6 7 7713.13 368.189 13

2 6 -7 7946.03 373.262 17

2 -6 -7 7309.16 393.491 2

2 6 -7 7211.40 392.490 2

2 -6 -6 2238.76 125.779 5

2 6 -6 2297.74 127.259 5

-2 6 6 2510.76 131.518 13

2 6 -6 2082.55 137.259 13

2 6 -6 2104.03 146.067 2

2 -6 -5 859.471 62.5298 15

2 -6 -5 1017.35 66.2148 21

2 6 -5 811.330 74.9883 13

-2 6 5 851.438 66.5754 13

2 6 -5 777.869 59.9192 5

2 -6 -5 757.280 57.4554 5

2 6 -4 290.155 51.1174 13

-2 6 4 339.278 46.4852 13

2 -6 -4 280.789 41.9579 12

2 -6 -3 37508.2 1795.79 12

-2 6 3 39905.0 1797.28 13

2 -6 -2 7344.20 385.904 12

-2 6 2 7964.55 379.953 13

2 6 -1 15215.0 693.539 13

-2 6 1 14896.5 685.600 13

-2 6 0 9874.04 407.354 13

2 6 0 9962.27 415.033 13

-2 6 -1 77496.4 3575.38 13

-2 -6 -2 5927.45 269.865 5

-2 6 -2 5313.51 271.614 2

-2 6 -3 555.626 47.8552 2

-2 -6 -3 631.321 50.5971 2

-2 -6 -3 653.000 39.3721 5

-2 -6 -4 13008.0 659.477 2

-2 6 -4 13511.8 660.609 2

-2 6 -4 13788.8 650.439 5

-2 -6 -4 13249.3 649.689 5

-2 -6 -5 107771. 4728.24 21

-2 -6 -5 107554. 4727.57 15

-2 6 -5 96214.2 4736.39 2

-2 -6 -5 96068.2 4735.88 2

-2 6 -5 104088. 4744.51 13

-2 6 -5 106662. 4725.36 5

-2 -6 -5 98736.1 4723.78 5

-2 -6 -6 438.125 50.0481 15

-2 -6 -6 255.108 59.5357 2

-2 6 -6 243.297 60.7711 2

-2 -6 -6 352.579 38.8572 5

-2 6 -6 283.804 38.0262 5

-2 6 -7 31890.2 1495.54 5

-2 -6 -7 32189.1 1495.49 5

-2 -6 -7 33564.3 1493.57 21

-2 6 -7 31378.3 1496.40 16

-2 6 -7 29594.8 1513.08 2

-2 6 -7 34084.8 1505.92 17

-2 -6 -7 31068.7 1514.67 2

-2 -6 -8 5446.49 272.621 15

-2 6 -8 5446.98 276.153 5

-2 -6 -8 5258.42 274.470 5

2 -6 8 5182.04 277.024 11

-2 -6 -8 5782.67 274.121 21

-2 -6 -8 5071.05 299.485 2

-2 6 -8 4548.55 295.368 2

-2 6 -8 5683.37 287.622 17

-2 6 -9 8647.28 480.531 2

-2 -6 -9 8323.71 478.278 2

-2 6 -9 9769.52 455.944 5

-2 -6 -9 9178.32 453.786 5

-2 6 -9 8732.78 461.981 17

-2 -6 -9 8847.01 445.708 15

-2 -6 -9 8982.14 445.512 21

-2 -6 -9 9939.02 448.881 26

-2 -6 -10 1091.77 67.5724 8

-2 -6 -10 1283.16 94.2192 26

-2 6 -10 1156.41 115.527 17

-2 6 -10 972.997 89.0502 5

-2 -6 -10 1001.22 87.0360 5

-2 -6 -10 1086.47 85.0780 15

-2 6 -10 899.559 121.203 2

-2 -6 -10 860.378 123.377 2

-2 -6 -10 1098.84 68.3959 10

-2 -6 -10 1239.43 90.1061 21

-2 6 -11 7345.56 372.491 17

-2 -6 -11 7246.68 349.230 15

-2 6 -11 6190.10 394.244 2

-2 -6 -11 6909.01 402.147 2

-2 6 -11 6710.40 361.467 5

-2 -6 -11 6838.15 361.422 5

-2 -6 -11 6713.05 332.140 8

-2 -6 -11 7204.75 349.545 21

-2 -6 -11 6720.20 332.864 10

-2 -6 -12 85.4171 56.1800 15

-2 6 -12-34.5412 96.6700 2

-2 -6 -12 43.0611 99.1169 2

-2 6 -12 6.85874 29.1299 8

-2 -6 -12 18.6882 30.4972 8

-2 6 -12 50.5919 42.3509 24

-2 6 -12-45.9413 88.5964 17

-2 -6 -12 20.9682 58.2433 21

-2 6 -12 37.6445 46.5452 5

-2 -6 -12-4.66744 44.9504 5

-2 -6 -13 4485.62 253.466 15

-2 -6 -13 4657.38 324.748 2

-2 6 -13 4409.93 324.040 2

-2 -6 -13 4716.50 258.551 21

-2 6 -13 4725.21 249.127 24

-2 -6 -13 4172.26 256.122 23

-2 6 -13 5027.50 278.311 5

-2 -6 -13 4266.66 270.540 5

-2 6 -13 5111.61 289.758 17

-2 6 -13 4995.33 240.870 8

-2 -6 -13 5048.48 240.552 8

-2 -6 -13 4652.81 248.511 18

-2 -6 -14 548.743 81.6582 23

-2 6 -14 483.783 66.0288 24

-2 -6 -14 504.361 63.7379 18

-2 6 -14 577.703 114.831 17

-2 -6 -14 635.649 75.0237 15

-2 -6 -14 446.671 76.4241 5

-2 -6 -14 537.593 53.7668 8

-2 6 -14 494.552 52.8399 8

-2 6 -14 488.376 136.359 2

-2 6 -15 2071.83 133.218 24

-2 -6 -15 2067.03 122.269 8

-2 6 -15 2158.74 123.413 8

-2 6 -15 2342.39 182.964 17

-2 -6 -15 2193.72 163.953 28

-2 -6 -15 1989.29 136.654 15

-2 6 -16 33.2493 45.9923 24

-2 -6 -16 87.6955 85.1473 28

-2 -6 -16 22.6629 36.1753 8

-2 6 -16 41.7773 38.3937 8

-2 6 -17 517.908 58.7072 24

-2 6 -17 530.402 56.0601 8

-2 -6 -17 394.626 92.0429 28

-2 6 -18 199.465 42.1928 8

-2 -6 -18 277.879 90.2932 28

-2 6 -19 12.3389 34.1453 8

-2 -6 -19-10.1221 77.5106 28

2 -8 -20 68.5464 63.9412 28

2 8 -19 35.2832 43.0498 22

2 -8 -19 100.300 78.4112 28

2 8 -18 928.703 71.3846 22

2 -8 -18 804.867 106.944 28

2 -8 -17 170.754 91.5803 28

2 8 -17 261.637 77.3474 17

2 8 -17 316.482 53.6737 24

2 -8 -17 290.041 77.8819 23

2 8 -16 1054.23 109.802 17

2 -8 -16 1039.54 117.227 26

2 8 -16 1116.55 86.2037 24

2 -8 -16 1008.57 111.401 23

2 -8 -15 836.434 83.0171 18

2 8 -15 796.801 100.775 17

2 -8 -15 877.520 105.283 23

2 8 -15 893.185 80.0953 24

2 -8 -15 924.220 110.149 26

2 -8 -14 967.796 84.1566 18

2 8 -14 739.521 102.449 17

2 -8 -14 936.221 111.482 23

2 8 -14 884.954 80.2371 24

2 -8 -14 873.470 106.694 26

2 -8 -13-17.7403 51.9525 18

2 8 -13 19.4889 73.7107 17

2 8 -13-32.3197 46.9328 24

2 -8 -13-2.86982 50.5919 20

2 -8 -12 427.361 79.6823 21

2 -8 -12 450.770 65.6830 18

2 8 -12 393.473 81.6903 17

2 -8 -11 3282.80 188.362 21

2 -8 -11 2857.26 176.862 18

2 -8 -11 3375.84 191.227 26

2 -8 -11 3079.32 194.165 25

2 8 -11 3112.41 189.455 17

2 -8 -10 323.656 75.3671 25

2 8 -10 307.367 50.5090 24

2 -8 -10 202.618 56.2243 18

2 8 -10 355.365 70.3185 17

2 -8 -10 347.648 68.8007 21

2 -8 -10 210.442 67.1372 26

2 8 -9 8182.07 395.366 24

2 -8 -9 7905.03 401.592 21

2 8 -9 7987.92 405.000 17

2 -8 -9 7917.53 398.706 18

2 -8 -9 7567.24 411.328 25

2 -8 -9 8568.94 404.803 26

2 8 -8 1098.56 80.2755 24

2 8 -8 1207.24 95.2615 17

2 -8 -8 1282.90 109.015 25

2 -8 -8 1097.00 93.0002 26

2 8 -8 1189.08 108.821 13

2 -8 -7 1042.41 103.612 25

2 8 -7 1103.94 78.6853 24

2 -8 -7 1304.35 88.4046 15

2 8 -7 1043.94 87.3280 17

2 -8 -7 1119.07 86.5129 21

2 8 -7 1090.41 101.626 13

2 -8 -6 1416.73 95.9838 18

2 8 -6 1532.01 91.2240 24

2 8 -6 1270.15 96.0463 17

-2 8 6 1607.47 103.398 13

2 8 -6 1542.36 116.575 13

2 -8 -6 1341.71 98.0379 21

2 -8 -6 1381.09 100.338 15

2 -8 -5 19582.5 894.695 21

-2 8 5 19054.8 907.053 13

2 8 -5 17866.3 915.886 13

2 -8 -5 19005.2 893.088 18

2 -8 -4 2450.32 138.417 21

-2 8 4 2666.64 153.862 13

2 8 -4 2270.69 158.578 13

2 -8 -4 2451.88 137.314 18

2 -8 -3 2821.55 139.244 21

-2 8 3 2472.33 152.604 13

2 -8 -3 2621.85 136.994 18

2 -8 -2 6917.65 340.338 21

2 -8 -2 6779.57 352.051 12

-2 8 2 7283.43 361.935 13

2 8 -2 7173.61 367.824 13

2 -8 -2 7019.12 339.240 18

2 -8 -1 38983.8 1718.33 21

2 -8 -1 37331.5 1716.79 18

2 8 -1 36843.8 1746.44 13

-2 8 1 35641.2 1737.85 13

2 -8 -1 34186.0 1742.68 12

2 -8 0 188.941 36.0025 21

2 -8 0 121.814 76.8536 12

-2 8 0 173.310 52.1578 13

2 8 0 296.038 59.7080 13

2 -8 0 252.395 36.4464 18

-2 -8 -1 612.507 61.5381 21

-2 -8 -1 592.673 54.5237 15

-2 8 -1 529.948 69.6247 13

-2 -8 -2 37777.1 1770.06 21

-2 -8 -2 37279.7 1767.84 15

2 8 2 40831.5 1799.06 13

-2 8 -2 38738.8 1790.12 13

-2 -8 -3 22734.7 1041.41 21

-2 -8 -3 21829.2 1039.52 15

-2 8 -3 21297.0 1058.82 13

2 8 3 23166.4 1067.58 13

-2 -8 -4 276.162 52.5289 21

-2 -8 -4 228.706 46.9911 15

2 8 4 141.452 56.9787 13

-2 -8 -5 5549.27 304.569 21

2 8 5 6811.08 332.952 13

-2 8 -5 6336.50 328.880 13

-2 8 -5 6168.33 299.045 16

-2 -8 -5 5992.92 304.943 15

-2 8 -6 17790.2 832.597 24

-2 8 -6 16321.6 832.409 16

-2 -8 -6 17696.4 838.237 15

-2 -8 -6 19126.8 836.758 26

2 8 6 17920.1 859.081 13

-2 -8 -7 6799.01 334.179 26

-2 8 -7 6808.48 330.496 24

-2 8 -7 6449.50 335.718 16

-2 -8 -7 6452.06 335.841 15

-2 8 -7 6217.73 345.691 17

-2 -8 -7 6827.49 336.785 21

2 8 7 7087.05 354.049 13

-2 8 -8 148.870 41.3107 24

-2 8 -8 103.944 62.2286 16

-2 -8 -8 199.541 57.5307 15

-2 8 -8 118.267 71.9454 17

-2 -8 -8 152.250 58.0966 21

-2 -8 -8 156.015 51.9818 26

-2 8 -9 726.618 98.6100 17

-2 -8 -9 649.731 78.6366 21

-2 8 -9 725.616 68.5415 24

-2 -8 -9 748.841 79.3099 26

-2 8 -10 5036.65 294.978 17

-2 -8 -10 5209.92 279.231 21

-2 -8 -10 5437.13 277.917 26

-2 8 -10 5138.46 269.588 24

-2 8 -11 218.944 52.7954 24

-2 -8 -11 220.796 59.5971 18

-2 8 -11 124.105 83.9227 17

-2 -8 -11 204.373 66.0452 15

-2 -8 -11 167.242 67.8421 21

-2 -8 -11 171.143 67.2246 26

-2 -8 -12 1903.39 130.252 18

-2 8 -12 1978.20 163.085 17

-2 -8 -12 1974.49 141.112 21

-2 -8 -12 2022.77 141.235 26

-2 -8 -12 1819.42 135.219 15

-2 -8 -13 44.8203 59.4125 15

-2 8 -13 33.3800 90.0170 17

-2 -8 -13 161.825 58.8831 18

-2 -8 -13 122.216 78.4366 26

-2 -8 -13 73.4353 67.5593 21

-2 8 -14 203.777 56.0887 24

-2 8 -14 290.353 96.2305 17

-2 -8 -14 319.446 62.3612 18

-2 8 -15 837.392 113.117 17

-2 8 -15 536.998 70.4617 24

-2 -8 -15 823.096 98.7032 28

-2 8 -16 603.330 107.306 17

-2 8 -16 706.388 70.7697 24

-2 -8 -16 803.550 98.9778 28

-2 8 -17 227.370 90.1362 17

-2 8 -17 175.104 49.3919 24

-2 -8 -17 206.005 74.1750 28

-2 8 -18 138.938 79.8815 17

-2 8 -18 127.191 41.7474 24

-2 -8 -18 69.7026 69.1385 28

2 10 -19 63.4438 34.1369 22

2 10 -18 747.582 62.7067 22

2 -10 -18 647.680 80.0229 28

2 -10 -17 956.216 98.5287 28

2 -10 -16 88.2698 77.1973 28

2 10 -16 127.357 49.4235 24

2 -10 -15 999.270 97.6828 23

2 -10 -14 140.035 69.5329 23

2 -10 -14 66.3548 81.6030 26

2 -10 -14 120.787 60.8021 18

2 10 -13 596.095 69.4554 24

2 -10 -13 547.502 74.8395 18

2 -10 -13 500.937 70.6820 20

2 -10 -13 499.327 91.5181 26

2 10 -12 1045.89 86.7549 24

2 -10 -12 1111.81 108.580 21

2 10 -12 868.573 93.1993 17

2 -10 -12 955.039 92.6479 18

2 -10 -12 947.839 89.2160 20

2 -10 -12 1125.63 110.697 26

2 10 -11 3675.70 192.252 24

2 -10 -11 3479.45 204.470 21

2 10 -11 2969.93 193.242 17

2 -10 -11 3184.82 194.861 18

2 -10 -11 3655.36 207.047 26

2 -10 -10 216.513 67.7264 18

2 10 -10 270.229 65.8155 17

2 -10 -10 390.986 80.1783 25

2 10 -10 226.375 53.4971 24

2 -10 -10 315.490 77.5286 26

2 10 -9 1258.11 103.088 17

2 10 -9 1528.97 102.507 24

2 -10 -9 1515.63 116.455 26

2 -10 -9 1420.69 124.944 25

2 -10 -8 192.245 65.0051 26

2 -10 -8 123.710 73.1718 25

2 10 -8 237.692 49.2593 24

2 -10 -8 109.459 54.8775 18

2 10 -8 114.799 52.7562 17

2 10 -8 160.275 69.7735 13

2 -10 -8 152.881 63.8832 21

2 -10 -7 4945.09 272.877 25

2 -10 -7 4812.08 251.575 26

2 10 -7 4592.28 242.452 24

2 10 -7 4622.57 244.726 16

2 -10 -7 4501.20 248.661 18

2 10 -7 4634.28 282.840 13

2 -10 -7 4694.60 252.534 21

2 10 -6 7094.40 346.982 24

2 -10 -6 6868.73 353.540 18

-2 10 6 6858.47 366.070 13

2 10 -6 7156.52 387.666 13

2 -10 -6 7039.13 354.917 21

2 10 -5 973.589 71.9856 24

2 -10 -5 849.590 78.3785 18

2 10 -5 975.370 107.312 13

-2 10 5 957.492 92.6689 13

2 -10 -5 944.740 84.8010 21

2 10 -4 8702.85 398.022 24

2 -10 -4 7699.16 401.484 18

-2 10 4 8153.57 422.567 13

2 -10 -4 7955.78 402.860 21

2 10 -3 2929.42 150.891 24

-2 -10 3 2468.97 150.191 21

2 -10 -3 2529.17 155.653 21

-2 10 3 3197.64 183.509 13

2 -10 -3 2958.22 157.416 18

2 -10 -2 6710.27 329.778 21

-2 -10 2 6555.02 327.661 21

2 -10 -2 6535.96 328.549 18

2 10 -2 6737.53 366.949 13

-2 10 2 6659.02 356.241 13

2 -10 -1 298.695 49.3288 18

-2 -10 1 337.026 45.2935 18

-2 10 1 185.347 58.7736 13

2 10 -1 149.478 72.6970 13

-2 -10 1 410.709 55.9563 21

2 -10 -1 340.308 49.7574 21

2 -10 0 296.068 44.1592 18

2 10 0 390.854 76.5969 13

-2 10 0 201.867 64.9135 13

2 -10 0 377.814 49.7093 21

-2 -10 0 344.755 57.0356 21

2 -10 1 450.087 48.3394 18

-2 10 -1 419.094 70.4139 13

2 10 1 497.541 80.8535 13

-2 -10 -1 378.675 60.8689 21

2 -10 1 317.361 44.7695 21

2 10 2 25252.5 1186.90 13

-2 10 -2 25348.6 1183.12 13

-2 -10 -2 23851.1 1150.90 21

-2 10 -3 307.297 41.8049 24

-2 -10 -3 308.153 58.6746 15

-2 -10 -3 217.418 60.3610 21

-2 -10 -3 211.948 48.3278 26

2 10 3 338.126 71.8457 13

-2 10 -3 427.653 73.8033 13

-2 10 -4 4572.12 233.692 24

-2 -10 -4 4248.69 242.992 15

-2 10 -4 4419.96 234.687 16

-2 -10 -4 4443.49 243.225 21

-2 -10 -4 5053.71 239.003 26

2 10 4 4530.24 269.349 13

-2 10 -5 570.956 60.0462 24

-2 -10 -5 697.667 77.1783 15

-2 10 -5 712.079 66.7226 16

-2 -10 -5 663.000 76.5658 21

-2 -10 -5 633.600 67.6154 26

2 10 5 587.851 82.3754 13

-2 -10 -6 3008.93 170.623 26

-2 10 -6 2948.30 165.192 24

-2 10 -6 3170.62 173.002 16

-2 -10 -6 3218.59 178.512 15

-2 10 -6 2558.78 178.559 17

-2 -10 -6 2658.21 174.227 21

2 10 6 3205.10 195.162 13

-2 10 -7 738.518 93.8493 17

-2 10 -7 883.158 90.4102 16

-2 -10 -7 785.940 87.8274 15

-2 10 -7 889.666 74.8895 24

-2 -10 -7 908.427 82.8332 26

-2 10 -8-34.2897 71.6967 17

-2 -10 -8 72.5431 65.0183 15

-2 10 -8 64.6136 70.5449 16

-2 10 -8 76.1353 47.4216 24

-2 -10 -8 123.536 62.2643 26

-2 10 -9 3504.65 185.930 24

-2 -10 -9 3110.00 192.892 15

-2 10 -9 2922.08 200.511 17

-2 -10 -9 3172.63 194.672 21

-2 -10 -9 3296.92 190.247 26

-2 -10 -10 1121.26 104.762 18

-2 10 -10 1381.64 99.2276 24

-2 -10 -10 1207.74 112.005 15

-2 10 -10 1203.22 119.908 17

-2 -10 -10 1266.26 106.337 26

-2 -10 -10 1232.16 114.743 21

-2 10 -11 825.219 82.4597 24

-2 -10 -11 1004.48 99.6376 21

-2 -10 -11 916.021 97.9334 26

-2 -10 -11 869.277 90.6654 18

-2 10 -11 728.464 104.469 17

-2 10 -12 271.430 60.8471 24

-2 10 -12 316.076 87.0488 17

-2 -10 -12 359.342 71.8685 18

-2 -10 -12 346.704 79.5526 26

-2 -10 -12 244.584 77.9126 21

-2 10 -13 1061.03 90.3099 24

-2 -10 -13 1154.49 110.921 21

-2 10 -13 1084.78 116.119 17

-2 -10 -13 1007.36 107.082 26

-2 -10 -13 957.462 92.8445 18

-2 -10 -14 1528.65 110.576 18

-2 10 -14 1479.69 129.594 17

-2 -10 -14 1554.09 116.799 28

-2 10 -14 1464.16 109.472 24

-2 -10 -15 25.3765 58.1171 28

-2 10 -15-24.4705 72.3989 17

-2 10 -16 130.397 47.4710 24

-2 -10 -16 98.6145 59.2648 28

-2 10 -17 427.350 55.0479 24

-2 10 -18 215.102 40.1796 22

2 12 -17 260.521 42.7656 22

2 12 -16 856.769 67.4475 22

2 -12 -15 206.908 61.4460 28

2 12 -15 93.7282 42.4661 22

2 -12 -14 334.825 79.3742 26

2 -12 -13 1410.36 110.443 18

2 -12 -13 1502.93 123.632 26

2 12 -13 1569.22 107.286 24

2 -12 -12 3757.48 214.080 18

2 -12 -12 4002.71 222.816 26

2 12 -12 3984.27 209.618 24

2 -12 -12 3439.04 209.414 20

2 -12 -11 870.524 97.8257 26

2 12 -11 730.257 75.7048 24

2 -12 -11 718.101 78.3443 20

2 -12 -10 1468.91 122.979 21

2 -12 -10 1223.45 112.573 25

2 -12 -10 1848.91 127.817 26

2 12 -10 1624.87 108.425 24

2 -12 -9 102.810 66.2774 18

2 -12 -9 167.938 72.4984 21

2 -12 -9 131.473 67.7342 25

2 12 -9 157.787 51.8297 24

2 -12 -9 219.536 72.8847 26

2 -12 -8 3888.04 219.020 21

2 -12 -8 3657.96 214.663 18

2 12 -8 3840.53 207.722 24

2 -12 -8 4229.29 221.555 26

2 12 -7 2122.94 131.885 24

2 -12 -7 2138.60 141.401 18

2 -12 -7 1976.30 140.790 21

2 12 -7 2126.98 143.552 16

2 -12 -7 2299.85 146.283 26

2 -12 -7 2085.39 155.889 25

2 -12 -6 1851.95 124.927 26

2 -12 -6 1531.49 139.573 25

2 12 -6 1699.48 110.460 24

2 12 -6 1607.26 153.967 13

2 -12 -6 1585.98 120.584 21

2 12 -6 1792.40 121.022 16

2 -12 -6 1460.69 116.252 18

2 -12 -5 14.9403 59.5869 26

2 12 -5 54.2196 46.7236 24

2 12 -5 37.2174 78.0977 13

2 -12 -5 23.6869 52.9018 18

2 -12 -5 34.5553 60.5063 21

2 12 -5 52.8310 50.2183 16

2 12 -4 2762.03 156.663 24

-2 12 4 2551.23 180.545 13

2 -12 -4 2715.08 162.898 21

2 -12 -4 2695.50 161.536 18

2 12 -3 1750.56 149.294 13

-2 12 3 1559.03 128.945 13

2 -12 -3 1426.87 107.364 18

2 -12 -3 1296.56 104.762 21

2 -12 -2 110.840 52.6874 18

-2 12 2 11.4850 57.1004 13

2 12 -2-14.1160 75.4046 13

2 -12 -2 4.91470 48.1127 21

-2 -12 2 21.0175 44.9895 21

2 -12 -1 1416.98 103.531 21

-2 -12 1 1386.91 106.543 21

2 -12 -1 1587.07 107.404 18

2 12 -1 1985.20 156.099 13

-2 12 1 1933.80 144.723 13

2 -12 0 208.477 52.9127 18

-2 -12 0 198.236 51.4094 18

-2 12 0 394.646 77.2921 13

2 12 0 279.242 81.4683 13

2 -12 0 218.153 47.9695 21

-2 -12 0 228.367 58.0734 21

-2 -12 -1 228.432 59.5845 21

-2 -12 -1 199.706 53.2148 18

2 12 1 228.852 78.7482 13

-2 12 -1 285.372 77.1414 13

2 12 2 2681.11 197.138 13

-2 12 -2 2660.41 192.810 13

-2 -12 -2 2645.04 164.144 21

-2 -12 -2 2753.83 161.087 18

2 12 3 820.004 102.239 13

-2 12 -3 796.391 102.993 13

-2 -12 -3 505.830 73.1315 18

-2 12 -3 681.827 71.4133 16

-2 -12 -3 553.186 78.0621 21

-2 -12 -4 1249.20 91.7937 26

-2 12 -4 1137.86 87.5770 24

2 12 4 1126.21 118.103 13

-2 -12 -4 1055.05 102.959 21

-2 12 -4 1281.92 99.4730 16

-2 -12 -4 1096.82 97.7245 18

-2 -12 -5 75.1398 54.7505 26

-2 12 -5 66.2337 43.8868 24

2 12 5 64.9208 62.8312 13

-2 12 -5 85.1381 59.7494 16

-2 -12 -5 62.9624 59.5201 18

-2 -12 -5 22.8752 64.5352 21

-2 -12 -6 1874.80 116.995 26

-2 -12 -6 1621.54 125.886 21

-2 12 -6 1774.35 126.679 16

-2 -12 -6 1514.51 119.582 18

-2 12 -6 1522.98 107.150 24

-2 12 -6 1329.22 113.834 17

-2 -12 -7 134.901 60.5595 26

-2 12 -7 191.508 53.1813 24

-2 12 -7 268.165 80.1147 16

-2 -12 -7 137.964 73.2020 21

-2 12 -7 94.5431 64.5343 17

-2 -12 -7 118.970 66.8372 18

-2 -12 -8 2581.28 163.672 18

-2 12 -8 2453.19 151.322 24

-2 -12 -8 2618.95 157.654 26

-2 -12 -8 2300.19 164.042 21

-2 -12 -9 1017.83 104.584 18

-2 12 -9 1136.38 92.9768 24

-2 -12 -9 1276.06 105.099 26

-2 12 -10 929.650 86.0786 24

-2 -12 -10 1100.42 98.8147 26

-2 -12 -10 878.161 102.153 21

-2 12 -11-19.6393 51.0790 24

-2 -12 -11 96.5001 69.1444 26

-2 -12 -11 148.150 76.1301 21

-2 -12 -11 3.15700 62.3023 18

-2 -12 -12 558.305 87.0130 21

-2 -12 -12 556.873 77.2301 18

-2 12 -12 592.528 69.0863 24

-2 -12 -13 865.186 86.3804 18

-2 12 -13 1098.55 86.4613 24

-2 -12 -14 159.323 55.8498 18

-2 12 -14 134.272 50.3410 24

-2 12 -15 72.5900 46.7873 24

-2 12 -16 179.918 41.1050 22

2 14 -14 52.2749 34.6813 22

2 14 -13 178.767 40.3976 22

2 -14 -12 1398.00 107.662 26

2 14 -12 1327.75 86.3760 22

2 -14 -11 1330.86 101.341 26

2 14 -11 1154.11 78.3373 22

2 14 -10 106.654 37.3640 22

2 -14 -10 197.047 64.2301 18

-2 14 10 119.806 41.8153 16

2 -14 -10 59.3321 63.4755 26

2 -14 -10 109.465 56.7877 20

2 -14 -9 208.648 59.7954 20

2 -14 -9 235.152 65.8257 18

2 -14 -9 196.434 66.6713 26

2 14 -9 140.099 39.7323 22

2 -14 -8 2938.98 183.792 18

2 -14 -8 3304.62 184.280 26

2 -14 -8 3037.20 182.295 21

2 -14 -7 3847.90 211.713 18

2 -14 -7 3494.34 207.615 21

2 -14 -7 3682.22 207.869 26

2 -14 -6 190.383 64.1142 21

2 -14 -6 108.356 61.5615 26

2 -14 -6 57.6069 62.9524 18

2 -14 -5 634.192 78.6150 21

2 -14 -5 488.008 89.9328 25

2 14 -5 635.780 86.9183 16

2 -14 -5 579.511 76.8341 18

2 -14 -5 836.130 80.3166 26

2 14 -4 2573.91 157.665 16

2 -14 -4 2249.92 150.311 18

2 -14 -4 2312.17 143.946 26

2 -14 -4 2246.61 145.051 21

2 14 -3 168.567 65.4993 16

2 -14 -3 75.9442 57.0597 18

2 -14 -3 82.1216 53.5493 21

2 -14 -2 6.25992 56.1606 18

2 -14 -2 7.52392 48.4301 21

-2 -14 1 85.5870 51.2099 21

2 -14 -1 42.2548 45.6021 21

-2 -14 0 159.016 56.6605 21

-2 14 0 97.3189 60.0833 16

-2 14 -1 39.0323 58.5513 16

-2 -14 -1 48.1117 56.7868 18

-2 -14 -1-31.3223 58.2724 21

-2 14 -2 397.710 74.9036 16

-2 -14 -2 289.720 63.3067 18

-2 -14 -2 327.792 70.0571 21

-2 14 -3 646.331 84.0826 16

-2 -14 -3 496.005 73.5058 18

-2 -14 -3 416.750 74.0126 21

-2 14 -4 194.907 72.6206 16

-2 -14 -4 93.9564 66.0144 21

-2 -14 -4 185.134 65.7907 18

-2 -14 -5 2152.54 149.283 18

-2 -14 -5 1997.49 149.908 21

-2 -14 -6 42.1712 51.3146 26

-2 -14 -6 26.1476 62.2853 18

-2 -14 -6 86.0739 67.6853 21

-2 14 -7 81.8707 50.6823 24

-2 -14 -7 64.2027 69.5552 21

-2 -14 -7 126.224 54.4105 26

-2 -14 -7 100.270 64.5391 18

-2 -14 -8 1505.95 123.821 21

-2 14 -8 1758.35 116.002 24

-2 -14 -8 1676.83 115.657 26

-2 -14 -8 1632.54 121.967 18

-2 14 -9 106.321 52.4275 24

-2 -14 -9 118.549 59.0919 26

-2 -14 -9 209.153 69.5384 21

-2 -14 -9 72.6974 61.7753 18

-2 14 -10 209.921 53.7385 24

-2 -14 -10 100.061 58.8881 18

-2 -14 -11 7.20192 50.1889 20

-2 14 -13 202.777 40.0408 22

2 -16 -8 142.375 55.4796 20

-2 16 8 119.004 45.8958 16

2 -16 -7 686.127 76.8855 20

-2 16 7 1036.61 78.7510 16

2 -16 -6 618.734 72.7063 20

-2 16 6 681.482 71.1502 16

2 -16 -5 151.573 57.6332 20

-2 16 5 180.493 57.5918 16

2 -16 -4 704.354 76.3919 20

-2 16 4 885.919 85.0601 16

2 -16 -3 375.707 62.0909 20

-2 16 3 348.406 69.1930 16

2 -16 -2 648.258 74.1726 20

2 16 -2 992.581 99.1731 16

2 -16 -1 158.637 53.1542 20

2 16 -1 312.544 76.9807 16

-2 16 0 1496.29 116.118 16

2 16 0 1625.07 117.837 16

2 16 1 32.3211 61.2078 16

-2 16 -1-24.9971 70.3561 16

-2 -16 -2 77.6747 51.6607 21

-2 16 -2 34.3001 72.8938 16

2 16 2 151.889 68.5212 16

-2 -16 -3 44.0795 52.0685 21

-2 16 -3 52.7059 78.2108 16

-2 -16 -4 212.085 59.4834 21

-2 -16 -5 95.5781 56.4816 21

-2 -16 -6 112.913 58.3699 21

-2 -16 -7 78.9988 56.3766 21

-2 -16 -7 39.9827 51.1893 20

2 16 7 40.9501 47.5696 16

2 16 8 71.9633 46.5857 16

3 1 -22 65.2967 41.0179 8

3 1 -21 5.96964 43.0313 8

3 1 -20-11.2220 46.3165 8

3 -1 -20 37.2683 127.102 28

3 -1 -19 124.383 118.788 23

3 1 -19 50.5173 46.9335 8

3 1 -19 7.71060 149.918 28

3 -1 -19-95.3619 133.507 28

3 1 -18 22.6894 135.461 23

3 -1 -18 295.053 133.092 23

3 1 -18 271.253 54.3146 8

3 1 -18 219.027 153.758 28

3 -1 -18 99.1025 135.593 28

3 -1 -17 299.864 71.7844 7

3 1 -17 291.705 149.729 28

3 -1 -17 239.210 135.147 28

3 -1 -17 353.682 140.678 2

3 1 -17 322.185 149.308 23

3 -1 -17 245.805 126.448 23

3 1 -17 348.230 57.0488 8

3 1 -17 314.231 82.5224 5

3 -1 -17 328.619 83.7982 5

3 1 -16 38.6763 43.4597 8

3 1 -16-6.78617 62.7910 5

3 -1 -16 97.0704 62.9487 7

3 -1 -16 29.1960 68.8279 9

3 1 -16-23.2080 141.421 28

3 -1 -16 107.278 123.689 28

3 -1 -16-116.510 122.001 23

3 1 -16-7.86875 135.725 23

3 1 -16 35.5211 97.8622 17

3 -1 -16 175.719 135.271 2

3 1 -16 13.1253 37.6230 22

3 1 -15 1918.85 156.046 17

3 1 -15 2036.54 197.173 23

3 -1 -15 2069.85 181.478 23

3 -1 -15 1519.12 129.027 9

3 -1 -15 1999.76 222.935 2

3 -1 -15 1781.76 130.423 7

3 1 -15 1541.31 184.910 28

3 -1 -15 1833.56 181.496 28

3 1 -15 2172.66 120.876 8

3 1 -15 2087.48 160.746 5

3 1 -14 513.105 94.5588 17

3 -1 -14 406.947 65.9389 7

3 1 -14 502.752 134.897 23

3 -1 -14 591.976 123.566 23

3 -1 -14 455.705 72.1494 9

3 -1 -14 365.182 132.060 2

3 1 -14 755.877 128.719 28

3 1 -14 508.614 85.9671 5

3 1 -14 529.562 56.2314 8

3 1 -13 194.975 39.8815 8

3 -1 -13 100.987 62.8398 27

3 1 -13 85.6242 65.2632 27

3 1 -13 112.783 105.167 28

3 1 -13 124.697 78.8999 17

3 -1 -13 236.547 88.2506 17

3 -1 -13 89.7909 47.6550 7

3 1 -13 116.214 100.345 23

3 -1 -13 159.196 97.0876 23

3 -1 -13 130.325 51.8894 9

3 1 -13 195.956 63.4612 5

3 -1 -13 134.337 69.6836 26

3 -1 -13 220.580 128.596 2

3 -1 -13 204.318 82.0218 4

3 -1 -12 15454.8 773.989 7

3 -1 -12 16118.0 789.923 17

3 1 -12 16905.5 768.716 8

3 -1 -12 16747.8 786.353 27

3 1 -12 16656.8 786.947 27

3 1 -12 17371.4 817.845 23

3 -1 -12 17293.7 810.101 23

3 -1 -12 16302.5 845.260 2

3 -1 -12 14499.5 810.305 4

3 1 -12 17530.3 803.796 5

3 -1 -12 15657.1 776.366 9

3 1 -11 3942.35 225.274 5

3 -1 -11 3458.44 232.832 4

3 1 -11 3955.18 199.335 6

3 -1 -11 4009.64 218.464 17

3 -1 -11 3990.13 263.987 2

3 1 -10 89933.7 3985.83 5

3 -1 -10 83322.7 3995.75 4

3 1 -10 83994.9 3961.60 6

3 -1 -10 87196.6 3975.04 17

3 -1 -10 91123.6 4019.37 2

3 1 -9 746.397 70.2876 5

3 -1 -9 825.115 85.4065 4

3 1 -9 748.689 52.7507 6

3 -1 -9 602.008 51.4003 6

3 -1 -9 770.231 111.545 2

3 -1 -8 77857.7 3723.41 2

3 1 -8 83861.8 3702.22 5

3 -1 -8 77386.3 3709.98 4

3 -1 -7 28939.9 1410.23 2

3 1 -7 30959.9 1392.57 5

3 -1 -7 29187.7 1400.27 4

3 -1 -6 124606. 5163.51 2

3 1 -6 125538. 5145.03 5

3 -1 -6 124139. 5153.21 4

3 -1 -5 70341.9 3027.10 2

3 1 -5 72403.9 3013.35 5

3 -1 -5 69618.3 3019.81 4

3 1 -5 62780.1 3002.62 13

3 -1 -4 220794. 10057.6 2

3 -1 -4 233465. 10051.8 11

3 1 -4 206763. 10040.3 13

3 1 -4 227570. 10046.9 5

3 -1 -4 225070. 10052.7 4

3 1 -3 23182.2 1195.47 3

3 -1 -3 25300.6 1201.98 2

3 -1 -3 27165.6 1199.74 4

3 1 -3 23843.2 1192.36 5

3 1 -2 133841. 6318.74 3

3 1 -2 134824. 6316.50 5

3 -1 -2 145202. 6318.62 11

3 1 -1 37009.5 1641.64 12

3 1 -1 33875.3 1644.36 3

3 -1 0 9787.52 460.334 11

3 -1 1 31617.7 1461.10 11

3 -1 2 7103.83 343.026 11

-3 -1 -2 7040.22 347.577 1

3 -1 3 6792.16 300.738 11

-3 -1 -3 5072.14 294.799 1

3 -1 4 17571.8 846.431 11

-3 -1 -4 17220.0 832.953 1

-3 -1 -4 17769.4 830.839 2

-3 -1 -5 13561.2 704.827 1

-3 -1 -5 13498.6 701.122 2

3 -1 6 14177.7 677.854 11

-3 -1 -6 14713.8 703.309 1

-3 -1 -6 14313.2 697.606 2

-3 -1 -7 5452.60 305.027 1

-3 1 -7 5603.21 278.656 5

-3 -1 -7 5212.33 303.190 2

-3 -1 -8 22049.1 1052.22 1

-3 1 -8 22403.3 1022.65 5

-3 -1 -8 20890.5 1045.23 2

-3 1 -8 21148.2 1018.07 17

-3 -1 -9 19096.2 959.723 1

-3 1 -9 19595.5 923.674 17

-3 -1 -9 19840.0 923.965 17

-3 -1 -9 18455.2 959.133 2

-3 1 -9 19613.6 928.903 5

-3 -1 -10 62333.6 2868.28 17

-3 1 -10 60538.4 2868.74 17

-3 1 -10 63371.9 2877.59 5

-3 -1 -10 61170.1 2911.94 2

-3 -1 -11 8668.45 513.846 2

-3 -1 -11 9209.23 449.339 10

-3 1 -11 9458.60 450.254 10

-3 1 -11 9743.19 477.149 23

-3 -1 -11 9695.91 473.771 28

-3 -1 -11 9162.27 471.695 17

-3 1 -11 9200.55 472.184 17

-3 1 -11 8664.62 475.988 5

-3 -1 -11 9090.54 456.050 7

-3 1 -11 9802.23 449.782 8

-3 -1 -12 546.464 124.721 2

-3 1 -12 508.573 52.5448 10

-3 1 -12 389.736 88.0964 23

-3 -1 -12 286.370 78.1968 23

-3 -1 -12 379.476 83.6923 28

-3 -1 -12 353.539 92.4985 17

-3 1 -12 358.234 89.0256 17

-3 1 -12 494.839 73.9505 5

-3 1 -12 530.044 50.8120 8

-3 -1 -13 9379.44 572.856 2

-3 1 -13 10683.5 489.697 10

-3 -1 -13 9318.65 509.542 23

-3 1 -13 9507.38 515.941 23

-3 -1 -13 10668.6 523.015 17

-3 1 -13 10034.7 519.468 17

-3 -1 -13 9600.29 518.211 28

-3 1 -13 10443.2 488.046 8

-3 1 -13 9936.77 523.373 5

-3 1 -14 12779.3 600.665 17

-3 -1 -14 12499.0 598.663 17

-3 1 -14 12681.3 561.772 10

-3 1 -14 10431.0 587.960 23

-3 1 -14 10077.7 550.310 24

-3 -1 -14 10741.5 581.295 23

-3 -1 -14 12340.2 659.424 2

-3 -1 -14 11053.9 590.466 28

-3 1 -14 12873.2 561.241 8

-3 1 -14 12323.4 604.980 5

-3 -1 -15 1388.21 156.840 17

-3 1 -15 1277.44 155.481 17

-3 1 -15 1444.52 98.0229 10

-3 -1 -15 1124.38 123.173 23

-3 -1 -15 1245.68 192.321 2

-3 -1 -15 1262.50 150.075 28

-3 1 -15 1442.91 97.0467 8

-3 1 -15 1223.67 125.829 5

-3 1 -16 178.927 122.284 17

-3 -1 -16 254.925 118.230 17

-3 1 -16 128.170 51.4466 10

-3 -1 -16 126.671 87.7514 23

-3 -1 -16 25.9033 136.054 2

-3 1 -16 266.021 78.7078 5

-3 -1 -16 145.071 115.307 28

-3 1 -16 158.802 51.2206 8

-3 1 -17 535.036 128.708 17

-3 1 -17 492.668 62.2781 8

-3 -1 -17 341.026 121.824 28

-3 1 -18 2595.84 145.671 8

-3 -1 -18 1890.42 194.785 28

-3 1 -19 523.197 61.9080 8

-3 1 -20 124.648 45.9392 8

-3 1 -21 104.804 39.8538 8

-3 1 -22 29.4947 29.3696 8

3 3 -22 73.7431 36.7585 8

3 3 -21 164.872 43.0187 8

3 -3 -21 161.060 43.6501 8

3 -3 -20 9.12632 94.5713 23

3 3 -20 104.754 45.0009 8

3 -3 -20 67.1393 44.5652 8

3 -3 -19 163.785 106.499 23

3 -3 -19 148.726 48.4215 8

3 3 -19 143.964 46.9084 8

3 -3 -18 1127.46 88.1907 8

3 3 -18 1160.66 88.9992 8

3 -3 -17 181.541 72.1111 5

3 3 -17 328.414 81.8516 5

3 3 -17 243.950 51.7751 22

3 3 -17 268.804 52.5959 8

3 -3 -17 319.124 53.9693 8

3 -3 -17 240.588 116.989 23

3 3 -16 937.996 72.9408 24

3 -3 -16 1080.14 143.108 23

3 3 -16 920.561 94.7735 7

3 -3 -16 1013.40 96.4020 7

3 3 -16 1107.01 79.9215 8

3 -3 -16 1084.02 78.7558 8

3 3 -16 615.557 188.940 28

3 -3 -16 837.069 108.535 5

3 3 -16 849.104 111.382 5

3 3 -16 990.641 179.005 2

3 -3 -16 967.396 181.335 2

3 3 -15 5622.43 312.882 5

3 -3 -15 5244.18 310.091 5

3 3 -15 4428.33 350.582 28

3 3 -15 5026.21 300.162 17

3 3 -15 5188.04 278.396 7

3 -3 -15 5113.11 366.757 2

3 3 -15 5064.38 361.376 2

3 3 -15 5011.63 264.185 24

3 -3 -15 5516.86 321.686 23

3 3 -15 5033.16 352.141 23

3 3 -15 4879.71 280.767 9

3 3 -15 5544.85 268.410 8

3 -3 -15 5739.14 269.538 8

3 -3 -15 5444.89 279.802 7

3 -3 -15 5191.74 283.424 9

3 -3 -15 4834.13 291.115 26

3 3 -14 448.279 89.4749 17

3 -3 -14 358.646 65.5188 7

3 3 -14 300.764 63.7611 7

3 3 -14 486.386 151.172 28

3 3 -14 328.239 48.5070 8

3 -3 -14 272.532 45.3845 8

3 -3 -14 638.403 87.1016 26

3 -3 -14 395.771 66.2113 9

3 3 -14 337.521 69.6747 9

3 3 -14 155.501 67.3745 5

3 -3 -14 257.053 70.9556 5

3 3 -14 388.218 48.5058 24

3 -3 -14 612.719 117.282 23

3 3 -14 392.162 141.184 2

3 -3 -14 358.654 136.429 2

3 3 -13 3061.42 204.739 17

3 3 -13 3521.89 191.531 7

3 -3 -13 3686.19 191.731 7

3 -3 -13 3133.95 198.679 27

3 3 -13 2587.48 242.776 28

3 3 -13 4010.69 274.091 2

3 -3 -13 3693.23 270.083 2

3 -3 -13 3276.47 190.452 9

3 3 -13 3278.23 191.929 9

3 3 -13 4227.06 223.735 5

3 -3 -13 3745.51 218.412 5

3 -3 -13 3000.91 195.130 26

3 3 -13 3780.84 180.823 8

3 -3 -13 3664.93 180.474 8

3 -3 -13 3224.39 220.788 23

3 3 -13 2973.95 176.188 24

3 3 -12 435.470 45.9553 8

3 -3 -12 336.365 42.5851 8

3 3 -12 402.711 123.728 2

3 -3 -12 440.763 133.030 2

3 3 -12 416.707 48.4163 24

3 3 -12 388.425 61.5181 9

3 -3 -12 440.259 60.4218 9

3 3 -12 528.452 80.9373 17

3 3 -12 337.391 57.0270 7

3 -3 -12 416.138 58.7319 7

3 3 -12 380.157 69.0907 5

3 -3 -12 297.928 64.0853 5

3 -3 -12 534.259 68.3952 26

3 3 -12 330.355 87.7525 4

3 -3 -12 399.261 88.1248 4

3 -3 -12 572.844 73.0463 27

3 3 -12 563.155 118.674 28

3 3 -11 16494.7 798.183 17

3 3 -11 17156.6 835.759 23

3 -3 -11 16455.9 781.947 18

3 -3 -11 16831.3 784.817 15

3 -3 -11 15860.9 823.170 4

3 3 -11 17827.5 813.231 5

3 3 -11 15258.9 822.058 4

3 -3 -11 17220.2 811.850 5

3 -3 -11 17376.1 855.035 2

3 3 -11 17010.9 852.371 2

3 3 -10 2678.70 164.874 5

3 -3 -10 2337.31 160.646 5

3 3 -10 2509.77 178.570 4

3 -3 -10 2733.12 179.683 4

3 3 -10 2303.94 142.141 6

3 -3 -10 2321.67 142.005 6

3 -3 -10 3025.82 203.828 2

3 3 -10 2877.27 209.465 2

3 -3 -10 3332.14 146.585 18

3 -3 -10 3265.21 149.622 15

3 3 -10 3190.16 166.614 17

3 3 -9 5082.94 264.659 17

3 -3 -9 5071.08 257.623 21

3 -3 -9 5125.91 304.623 2

3 3 -9 5452.98 306.004 2

3 -3 -9 5416.00 287.142 4

3 3 -9 5541.71 274.434 5

3 -3 -9 5460.08 274.201 5

3 3 -9 4866.18 283.332 4

3 -3 -9 5250.00 253.622 6

3 3 -9 5019.66 252.673 6

3 -3 -8 1483.14 130.401 2

3 3 -8 1267.23 128.240 2

3 -3 -8 1392.43 90.3965 11

3 -3 -8 1566.89 115.055 4

3 3 -8 1532.59 100.798 5

3 -3 -8 1596.31 99.5344 5

3 3 -8 1498.55 112.187 4

3 3 -7 71404.9 3269.99 2

3 -3 -7 72969.5 3270.16 2

3 -3 -7 69059.7 3239.54 11

3 3 -7 66063.6 3242.10 13

3 3 -7 70951.8 3258.27 4

3 3 -7 74586.3 3249.80 5

3 -3 -7 70918.1 3248.01 5

3 -3 -7 72373.7 3258.58 4

3 -3 -6 2145.60 139.646 2

3 3 -6 2192.25 138.535 2

-3 3 6 1945.88 99.1194 13

3 3 -6 1945.80 112.088 13

3 -3 -6 2058.73 115.444 5

3 3 -6 2287.02 127.731 4

3 -3 -6 2149.73 126.036 4

3 3 -6 2257.64 116.671 5

3 -3 -5 40135.4 1859.93 2

3 3 -5 39543.9 1860.71 2

3 3 -5 37194.8 1845.34 13

-3 3 5 40119.4 1836.47 13

3 3 -5 41408.4 1846.36 5

3 -3 -5 40954.8 1845.71 5

3 -3 -5 41251.5 1853.10 4

3 3 -5 38907.2 1851.99 4

3 3 -4 134273. 6348.35 2

3 -3 -4 136326. 6348.32 2

3 3 -4 137230. 6342.40 4

3 -3 -4 136884. 6336.85 5

3 3 -4 141825. 6337.71 5

3 -3 -4 140497. 6342.69 4

3 -3 -4 137609. 6348.62 12

-3 3 4 141385. 6332.98 13

3 3 -4 132394. 6340.76 13

3 -3 -3 36031.7 1722.72 5

3 3 -3 35773.3 1722.96 5

3 -3 -3 38202.6 1727.03 4

3 3 -3 37008.6 1726.63 4

3 -3 -3 37363.7 1739.02 12

-3 3 3 39836.4 1722.13 13

3 -3 -2 51321.1 2432.87 3

3 3 -2 53241.8 2435.44 4

3 3 -2 52620.3 2433.47 3

3 -3 -2 51871.5 2455.96 12

-3 3 2 55008.8 2433.90 13

3 3 -1 1956.64 105.993 3

3 -3 -1 1805.77 104.221 3

-3 3 0 123471. 5665.82 13

-3 3 -1 475.272 42.4165 13

-3 3 -2 1911.39 105.542 13

-3 -3 -2 1748.43 111.488 1

-3 3 -2 1956.03 112.198 1

-3 3 -3 3213.94 173.139 2

-3 -3 -3 3269.05 173.718 2

-3 3 -3 3235.47 177.876 1

-3 -3 -3 3257.89 176.575 1

-3 -3 -4 49957.0 2272.41 2

-3 3 -4 47237.8 2271.33 2

-3 3 -4 48123.7 2274.34 1

-3 -3 -4 50974.3 2275.67 1

3 -3 5 75510.5 3377.29 11

-3 -3 -5 73240.3 3394.18 1

-3 3 -5 71677.0 3393.99 1

-3 3 -5 71143.0 3390.97 2

-3 -3 -5 73490.6 3391.92 2

-3 3 -6 696.306 56.6003 5

3 -3 6 608.294 57.1667 11

-3 3 -6 697.280 89.3447 1

-3 -3 -6 575.937 82.6532 2

-3 3 -6 515.583 75.8271 2

-3 -3 -6 576.741 83.1174 1

-3 -3 -7 20222.4 1006.68 2

-3 3 -7 19063.4 1005.38 2

-3 3 -7 22802.9 989.976 17

-3 3 -7 19708.2 1009.53 1

-3 -3 -7 20638.2 1010.60 1

3 -3 7 23099.6 1002.53 11

-3 3 -7 20987.3 986.652 5

-3 -3 -7 20281.7 985.811 5

-3 3 -8 5666.38 267.903 17

-3 3 -8 4694.97 256.777 5

-3 -3 -8 4534.96 256.079 5

-3 3 -8 4794.12 288.655 2

-3 -3 -8 5290.54 292.139 2

-3 -3 -8 5133.53 292.614 1

-3 3 -8 4847.85 291.553 1

-3 3 -9 36591.0 1755.82 17

-3 -3 -9 40767.4 1758.10 17

-3 3 -9 37264.2 1758.91 5

-3 -3 -9 37816.3 1759.62 5

-3 3 -9 36228.0 1786.83 1

-3 3 -9 36166.4 1786.89 2

-3 -3 -9 36374.5 1787.78 2

-3 -3 -10 5866.64 335.540 2

-3 3 -10 5937.26 335.980 2

-3 -3 -10 5812.34 295.966 17

-3 3 -10 5207.93 293.107 17

-3 3 -10 6193.26 297.030 5

-3 -3 -10 6120.34 295.895 5

-3 3 -11 4343.99 285.127 28

-3 3 -11 4006.73 300.060 2

-3 -3 -11 4471.73 302.461 2

-3 3 -11 4475.39 263.093 5

-3 -3 -11 4668.51 263.445 5

-3 -3 -11 4199.11 249.616 26

-3 -3 -11 4787.74 236.184 8

-3 3 -11 4950.25 237.232 8

-3 3 -11 4637.25 267.169 17

-3 -3 -11 5037.39 271.133 17

-3 -3 -11 4772.13 237.787 10

-3 3 -11 4899.10 238.614 10

-3 -3 -12 2251.23 143.030 15

-3 3 -12 1886.74 200.618 2

-3 -3 -12 2097.28 206.975 2

-3 3 -12 2528.63 140.356 10

-3 -3 -12 2551.68 140.979 10

-3 -3 -12 2456.84 159.758 23

-3 3 -12 2614.56 139.329 8

-3 -3 -12 2632.40 139.131 8

-3 -3 -12 2405.19 166.609 5

-3 3 -12 2318.53 166.122 5

-3 3 -12 2881.23 205.415 28

-3 -3 -12 3010.35 192.164 17

-3 3 -12 2559.54 179.797 17

-3 3 -13 8189.26 443.042 17

-3 3 -13 8167.81 489.691 2

-3 -3 -13 8856.43 498.096 2

-3 3 -13 8728.82 409.020 10

-3 -3 -13 8381.19 408.101 10

-3 -3 -13 6780.30 419.661 23

-3 -3 -13 7621.04 409.831 15

-3 -3 -13 8829.20 447.836 5

-3 3 -13 8935.74 447.503 5

-3 -3 -13 9212.62 409.680 8

-3 3 -13 8959.34 408.697 8

-3 -3 -13 7414.17 430.104 28

-3 3 -14 77.9694 107.400 17

-3 3 -14-54.4114 121.824 2

-3 -3 -14 43.7613 123.465 2

-3 3 -14 37.4179 40.5569 10

-3 -3 -14 16.3926 39.4254 10

-3 3 -14 18.8690 41.5226 24

-3 -3 -14 3.96595 68.4123 23

-3 -3 -14-2.53630 45.5925 15

-3 3 -14-36.3410 59.9093 5

-3 -3 -14-18.2772 58.9926 5

-3 3 -14 60.3587 40.2519 8

-3 -3 -14 69.6806 41.7865 8

-3 -3 -14 36.7662 90.9069 28

-3 3 -15 4615.96 266.694 17

-3 -3 -15 4202.46 321.590 2

-3 3 -15 3592.01 311.529 2

-3 3 -15 4293.56 215.236 10

-3 -3 -15 3984.33 213.006 10

-3 -3 -15 4055.51 254.675 5

-3 3 -15 4246.79 258.501 5

-3 -3 -15 3491.05 245.824 28

-3 3 -15 4498.08 215.445 8

-3 -3 -15 4365.30 214.478 8

-3 -3 -15 3461.27 210.054 15

-3 3 -15 3615.71 210.286 24

-3 -3 -15 3633.38 234.395 23

-3 -3 -16 239.378 106.453 28

-3 3 -16 190.500 49.8514 8

-3 -3 -16 174.442 46.9366 8

-3 -3 -16 212.521 75.8796 23

-3 3 -16 225.986 45.8469 24

-3 3 -16 159.622 115.833 17

-3 3 -16 198.669 67.2362 5

-3 -3 -16 103.319 67.2373 5

-3 -3 -16 75.8078 132.196 2

-3 3 -16 251.000 140.751 2

-3 3 -17 33.8660 112.085 17

-3 3 -17 26.4600 45.4466 8

-3 -3 -17 26.5628 40.8845 8

-3 -3 -17 42.0633 68.4099 23

-3 -3 -17 71.3215 100.115 28

-3 3 -18 74.9845 103.183 17

-3 -3 -18-43.7643 104.389 28

-3 3 -18 77.8549 46.4312 8

-3 -3 -18 71.1885 43.8908 8

-3 -3 -19 443.913 105.896 28

-3 -3 -19 550.112 59.8845 8

-3 3 -19 519.114 57.8412 8

-3 3 -20 100.146 40.6507 8

-3 -3 -20 96.0616 39.3487 8

-3 3 -21 9.72645 32.4123 8

3 -5 -21 443.307 100.884 28

3 -5 -20 150.084 77.9329 23

3 -5 -20 26.6546 100.749 28

3 5 -20 83.4110 40.0574 8

3 -5 -19 176.880 90.2333 23

3 -5 -19 201.658 115.497 28

3 5 -19 280.752 47.7038 8

3 5 -18 1692.22 111.213 22

3 -5 -18 1528.93 170.111 28

3 -5 -18 1616.03 153.443 23

3 5 -18 1832.70 109.717 8

3 -5 -18 1655.52 107.610 8

3 5 -17 677.254 64.9195 8

3 -5 -17 644.691 63.5765 8

3 5 -17 621.292 108.692 17

3 5 -17 681.700 65.0441 24

3 -5 -17 701.895 121.160 23

3 -5 -16 410.286 110.953 23

3 5 -16 268.068 51.1712 24

3 5 -16 223.193 74.1806 5

3 -5 -16 299.287 74.9969 5

3 5 -16 297.863 50.2576 8

3 -5 -16 319.121 50.5690 8

3 5 -16 474.312 99.8865 17

3 -5 -16 279.991 93.2318 26

3 -5 -15 117.995 80.5084 26

3 5 -15 182.989 66.3315 7

3 -5 -15 126.229 60.0567 7

3 5 -15 30.2813 40.0218 8

3 -5 -15 29.9363 36.6330 8

3 5 -15-18.5836 69.0974 5

3 -5 -15-6.12384 63.6780 5

3 5 -15 84.7109 87.4062 17

3 5 -15 48.4912 45.6422 24

3 -5 -15 27.2664 91.4715 23

3 5 -14 1137.08 165.997 2

3 -5 -14 1591.73 191.544 2

3 -5 -14 1155.79 116.797 26

3 -5 -14 1383.12 144.724 23

3 5 -14 1137.13 93.5883 24

3 5 -14 1743.43 93.8823 8

3 -5 -14 1354.33 104.801 7

3 5 -14 1536.53 108.719 7

3 -5 -14 1543.76 92.8982 8

3 -5 -14 1559.53 128.514 5

3 5 -14 1737.57 132.402 5

3 -5 -14 1068.34 92.9814 18

3 5 -14 1309.59 125.642 17

3 5 -13 4382.60 245.293 17

3 -5 -13 4113.38 220.185 18

3 -5 -13 4365.20 225.391 7

3 5 -13 4068.63 225.641 7

3 5 -13 3743.65 292.135 2

3 -5 -13 4078.94 301.243 2

3 -5 -13 4437.67 237.656 26

3 5 -13 4125.71 229.154 9

3 -5 -13 4428.82 230.391 9

3 -5 -13 4145.92 213.505 8

3 5 -13 4294.11 214.303 8

3 -5 -13 4009.24 247.766 5

3 5 -13 3861.68 246.188 5

3 5 -13 4004.72 218.495 24

3 -5 -12 1818.43 116.614 18

3 -5 -12 1673.45 114.865 20

3 -5 -12 1742.61 135.833 5

3 5 -12 2378.42 146.427 5

3 -5 -12 2093.02 111.295 8

3 5 -12 2166.43 111.548 8

3 5 -12 2008.18 142.168 17

3 -5 -12 1911.48 121.749 7

3 5 -12 2026.25 123.219 7

3 -5 -12 1780.87 132.097 27

3 -5 -12 1853.33 130.655 26

3 5 -12 1736.45 121.839 9

3 -5 -12 1971.46 125.145 9

3 5 -12 1897.95 115.452 24

3 -5 -12 1869.64 185.604 2

3 5 -12 1875.20 183.552 2

3 5 -11 242.500 44.2855 24

3 -5 -11 467.958 59.8168 9

3 5 -11 270.056 54.5549 9

3 -5 -11 317.443 36.5513 8

3 5 -11 239.158 37.7293 8

3 -5 -11 300.490 61.1736 21

3 -5 -11 334.774 46.3662 20

3 -5 -11 326.201 63.7767 27

3 5 -11 283.871 69.1029 17

3 -5 -11 287.108 51.6628 18

3 -5 -11 364.629 53.9260 15

3 -5 -11 232.400 53.3693 5

3 5 -11 308.307 60.7654 5

3 -5 -11 326.027 62.2867 26

3 -5 -11 164.175 100.433 2

3 5 -11 166.668 105.589 2

3 -5 -11 587.886 54.7925 7

3 5 -11 468.890 54.0857 7

3 -5 -10 3531.12 200.761 15

3 5 -10 3974.43 199.059 24

3 -5 -10 3664.94 206.549 26

3 -5 -10 3518.64 218.908 25

3 -5 -10 3806.29 200.495 18

3 -5 -10 3358.41 203.227 21

3 5 -10 3720.74 211.931 17

3 5 -10 4695.89 224.972 5

3 5 -10 3752.13 232.029 4

3 -5 -10 4220.55 236.304 4

3 -5 -10 4470.04 222.251 5

3 -5 -10 3934.90 255.148 2

3 5 -10 4639.88 262.929 2

3 -5 -9 2412.27 144.299 11

3 -5 -9 2780.63 156.736 5

3 5 -9 2821.31 173.988 4

3 5 -9 3093.34 161.214 5

3 -5 -9 2815.81 172.255 4

3 -5 -9 2536.66 143.464 18

3 -5 -9 2488.24 145.366 21

3 5 -9 2436.96 153.870 17

3 -5 -9 2767.06 193.056 2

3 5 -9 2762.96 193.712 2

3 -5 -9 2548.80 145.690 15

3 5 -8 36038.2 1819.45 13

3 5 -8 44038.4 1822.69 5

3 -5 -8 40758.3 1819.24 5

3 5 -8 38691.6 1829.95 4

3 -5 -8 39193.6 1829.86 4

3 -5 -8 38659.5 1807.04 18

3 -5 -8 37920.7 1807.83 21

3 5 -8 38218.0 1812.41 17

3 -5 -8 38791.6 1808.80 15

3 -5 -8 40563.4 1846.13 2

3 5 -8 39544.7 1846.20 2

3 -5 -7 65337.1 3053.72 5

3 5 -7 65841.6 3063.70 4

3 -5 -7 67064.0 3063.82 4

3 5 -7 71199.5 3056.81 5

3 -5 -7 67395.4 3046.31 18

3 -5 -7 67308.4 3076.72 2

3 5 -7 64309.9 3075.77 2

3 -5 -7 65054.9 3046.72 21

3 5 -7 59245.3 3055.93 13

3 -5 -7 67973.3 3047.61 15

3 -5 -6 3871.26 202.238 5

3 5 -6 3972.40 204.060 5

3 5 -6 3896.92 211.115 4

3 -5 -6 4121.24 212.145 4

3 5 -6 3528.70 208.306 13

-3 5 6 4246.84 198.893 13

3 5 -6 3815.79 224.055 2

3 -5 -6 3836.19 223.764 2

3 5 -5 740.929 67.3126 4

3 -5 -5 932.077 68.4945 4

3 5 -5 921.157 62.8033 5

3 -5 -5 866.373 61.9609 5

3 5 -5 898.228 71.9975 13

-3 5 5 932.904 61.6949 13

3 5 -4 88905.6 4186.29 4

3 -5 -4 94199.6 4186.91 4

3 -5 -4 89477.3 4197.52 12

3 5 -4 83849.0 4194.66 13

-3 5 4 96592.6 4188.45 13

3 5 -3 44684.4 2144.26 4

3 -5 -3 46730.7 2144.41 4

-3 5 3 49261.7 2151.96 13

3 5 -3 46832.9 2160.79 13

3 -5 -3 46117.1 2165.19 12

3 5 -2 1710.68 91.0984 4

3 -5 -2 1780.95 91.6977 4

-3 5 2 1808.45 103.819 13

3 -5 -2 1727.94 132.146 12

3 5 -1 30219.5 1453.15 4

3 -5 -1 32438.5 1454.20 4

-3 5 1 33969.1 1469.91 13

-3 5 0 77146.5 3699.91 1

-3 -5 0 76867.3 3698.39 1

-3 5 0 91646.2 3716.40 13

3 5 0 88589.4 3699.48 3

3 -5 0 77796.1 3696.94 3

-3 5 -1 21394.1 1028.27 1

-3 -5 -1 22343.8 1027.22 1

-3 5 -1 21407.1 1026.79 2

-3 -5 -1 22288.7 1026.78 2

-3 -5 -2 33174.8 1572.38 1

-3 5 -2 33126.3 1574.11 1

-3 5 -2 38455.4 1585.55 13

-3 -5 -2 34643.6 1572.41 2

-3 5 -2 32677.3 1571.89 2

-3 5 -3 26137.3 1219.36 1

-3 -5 -3 26386.0 1217.95 1

-3 -5 -3 25417.7 1216.23 2

-3 5 -3 26642.4 1217.77 2

-3 -5 -4 10065.6 468.797 1

-3 5 -4 9352.88 469.106 1

-3 -5 -4 9577.11 454.237 5

-3 5 -4 9437.96 454.173 5

-3 -5 -4 10002.5 467.738 2

-3 5 -4 9308.33 466.117 2

-3 -5 -5 6957.40 341.464 1

-3 5 -5 6299.89 340.085 1

-3 5 -5 6869.15 324.036 5

-3 -5 -5 6676.04 322.813 5

-3 -5 -5 6267.04 337.945 2

-3 5 -5 6584.27 340.024 2

-3 -5 -6 36270.0 1514.38 21

-3 5 -6 30504.8 1533.53 2

-3 -5 -6 33678.1 1536.56 2

-3 -5 -6 32060.7 1535.06 1

-3 5 -6 30025.2 1534.20 1

-3 -5 -6 31069.9 1515.30 5

-3 5 -6 32571.3 1516.53 5

-3 -5 -7 53687.4 2406.98 21

-3 5 -7 54594.4 2421.40 17

-3 -5 -7 49570.5 2434.39 2

-3 5 -7 50291.4 2435.94 2

3 -5 7 48905.3 2413.17 11

-3 5 -7 53920.7 2415.44 5

-3 -5 -7 52165.0 2414.02 5

3 -5 8 321.333 53.2400 11

-3 5 -8 403.398 74.9236 17

-3 -5 -8 317.720 50.0009 21

-3 -5 -8 224.746 78.4512 2

-3 5 -8 247.026 78.8549 2

-3 -5 -8 224.566 45.2871 5

-3 5 -8 296.865 47.3386 5

-3 -5 -9 363.866 56.3388 26

-3 -5 -9 354.752 53.4295 15

-3 5 -9 596.698 95.4741 2

-3 -5 -9 522.336 94.9972 2

-3 -5 -9 389.738 54.7181 21

-3 5 -9 633.774 63.3181 5

-3 -5 -9 589.452 61.5900 5

-3 5 -10 6371.37 348.165 5

-3 -5 -10 6254.70 346.201 5

-3 -5 -10 6185.69 333.040 15

-3 -5 -10 6424.33 332.198 21

-3 5 -10 5654.88 376.550 2

-3 -5 -10 6428.56 383.262 2

-3 5 -10 6667.29 323.230 8

-3 -5 -10 6898.26 323.118 8

-3 -5 -10 7071.27 324.162 10

-3 5 -10 7071.99 324.606 10

-3 -5 -11 3350.74 197.202 15

-3 -5 -11 3504.88 250.485 2

-3 5 -11 3231.05 251.753 2

-3 -5 -11 3755.81 187.411 8

-3 5 -11 3721.58 186.691 8

-3 5 -11 3589.90 213.397 5

-3 -5 -11 3505.92 213.781 5

-3 -5 -11 3279.66 195.271 21

-3 -5 -11 3824.62 188.403 10

-3 5 -11 3802.74 188.453 10

-3 -5 -12 3476.91 200.888 15

-3 -5 -12 3245.44 261.775 2

-3 5 -12 2705.78 248.062 2

-3 5 -12 3600.70 219.952 5

-3 -5 -12 3259.65 215.418 5

-3 -5 -12 3769.27 188.360 8

-3 5 -12 3772.93 188.962 8

-3 -5 -12 3308.85 198.566 21

-3 -5 -12 3733.24 188.293 10

-3 5 -12 3843.30 189.419 10

-3 -5 -13 59.0715 53.0336 15

-3 5 -13 167.765 112.548 2

-3 -5 -13 108.758 108.711 2

-3 5 -13 116.513 48.0972 24

-3 5 -13 260.692 63.9777 5

-3 -5 -13 222.814 62.5913 5

-3 -5 -13 93.1151 46.0044 18

-3 -5 -13 83.3481 61.8343 21

-3 5 -13 208.595 43.4912 10

-3 -5 -13 197.017 42.7772 10

-3 5 -13 267.883 42.2760 8

-3 -5 -13 226.172 39.8767 8

-3 -5 -14 1191.19 120.686 28

-3 -5 -14 1212.09 79.5562 8

-3 5 -14 1218.27 79.8356 8

-3 5 -14 1047.83 89.4984 24

-3 -5 -14 1152.38 93.5338 15

-3 -5 -14 975.505 105.840 5

-3 5 -14 1107.56 111.570 5

-3 -5 -14 877.538 81.1921 18

-3 5 -14 1011.36 160.344 2

-3 -5 -14 830.928 159.293 2

-3 5 -15 338.162 134.519 2

-3 -5 -15 514.925 141.060 2

-3 -5 -15 351.925 63.2472 15

-3 -5 -15 340.607 98.3584 28

-3 5 -15 453.167 55.2339 8

-3 -5 -15 480.867 54.3357 8

-3 5 -15 400.877 79.9967 5

-3 -5 -15 413.153 79.7006 5

-3 5 -15 384.103 61.0818 24

-3 5 -16 721.287 131.531 17

-3 -5 -16 567.108 105.061 28

-3 -5 -16 697.464 63.8820 8

-3 5 -16 702.342 63.6774 8

-3 5 -16 575.010 67.7101 24

-3 5 -17 675.954 126.768 17

-3 -5 -17 819.696 110.648 28

-3 -5 -17 578.305 59.5403 8

-3 5 -17 579.807 60.2266 8

-3 5 -17 635.024 63.9694 24

-3 5 -18 91.2504 98.8810 17

-3 -5 -18-40.3605 85.8514 28

-3 5 -18 35.8839 38.5205 8

-3 5 -19 29.7287 95.3110 17

-3 5 -19 53.5956 37.5159 8

-3 -5 -19 89.3055 82.2612 28

-3 5 -20 73.8290 31.9831 8

3 -7 -21 231.691 67.3999 28

3 -7 -20 20.0028 82.2923 28

3 -7 -19 1292.74 119.167 23

3 -7 -19 1533.69 140.640 28

3 7 -19 1724.84 104.484 22

3 -7 -18 120.709 80.6126 23

3 -7 -18 123.214 100.548 28

3 7 -18 45.1218 45.4331 22

3 7 -17 31.3931 78.7435 17

3 -7 -17 151.923 91.7366 23

3 7 -17 161.942 49.9078 24

3 7 -16 377.980 88.8871 17

3 -7 -16 315.747 90.7040 26

3 7 -16 349.894 58.1456 24

3 -7 -16 302.482 94.3926 23

3 7 -15 98.9444 82.3042 17

3 -7 -15 71.7833 51.7156 18

3 -7 -15 72.7154 81.5718 26

3 -7 -15 20.1480 87.9320 23

3 7 -15-5.22426 45.2460 24

3 -7 -14 33.4028 34.3897 8

3 -7 -14 169.336 78.9847 26

3 7 -14 52.5266 48.0784 24

3 7 -14 88.6142 77.8217 17

3 -7 -14 51.1936 51.8158 18

3 7 -13 2603.84 151.840 24

3 -7 -13 2695.39 156.759 18

3 7 -13 2449.69 166.486 17

3 -7 -13 2677.26 171.100 26

3 -7 -13 2380.47 152.860 20

3 -7 -12 2259.19 147.963 21

3 7 -12 2015.16 129.838 24

3 7 -12 1991.30 145.802 17

3 -7 -12 2228.60 148.004 26

3 -7 -12 2193.62 132.302 20

3 -7 -12 2113.53 134.665 18

3 -7 -11 70.1234 71.7298 25

3 -7 -11 185.951 54.4468 5

3 -7 -11 125.022 61.3843 21

3 -7 -11 174.601 55.7821 18

3 7 -11 174.756 68.6435 17

3 -7 -10 263.765 76.3539 25

3 -7 -10 170.338 52.9443 18

3 7 -10 170.439 64.0250 17

3 -7 -10 335.304 55.3832 5

3 -7 -10 252.978 64.4007 21

3 7 -9 17286.1 905.956 13

3 -7 -9 19413.3 888.825 21

3 -7 -9 20735.5 902.719 5

3 -7 -9 18590.8 885.183 18

3 7 -9 17781.7 889.121 17

3 -7 -9 18375.3 904.410 25

3 7 -9 19021.0 883.205 24

3 7 -8 801.295 67.4118 24

3 -7 -8 989.486 99.1856 25

3 7 -8 950.133 92.2019 13

3 -7 -8 946.647 75.6751 18

3 7 -8 922.461 80.7564 17

3 7 -7 40911.6 1905.17 17

3 -7 -7 41901.0 1905.10 21

3 7 -7 39933.1 1924.12 13

3 -7 -6 4862.20 240.777 21

-3 7 6 4879.62 244.742 13

3 7 -6 4258.60 255.690 13

3 -7 -5 16796.1 790.595 18

3 -7 -5 16958.9 791.322 21

-3 7 5 17657.6 801.581 13

3 7 -5 15578.6 810.190 13

3 -7 -4 5091.22 251.683 18

3 -7 -4 4881.24 253.215 21

3 7 -4 4964.68 275.540 13

3 -7 -4 5042.06 265.478 12

-3 7 4 5490.30 265.807 13

3 -7 -3 65923.2 3051.21 18

3 -7 -3 64629.5 3051.57 21

3 -7 -3 66340.9 3072.71 12

-3 7 3 70325.6 3067.61 13

3 -7 -2 4599.91 266.414 12

-3 7 2 4923.20 251.142 13

3 -7 -2 4683.48 233.287 18

3 -7 -1 39683.0 2063.24 12

-3 7 1 44154.0 2039.13 13

3 7 -1 46801.1 2053.27 13

3 7 0 1351.50 111.914 13

-3 7 0 1325.86 96.6098 13

3 7 1 130607. 5852.81 13

-3 7 -1 124099. 5840.62 13

-3 -7 -2 6642.66 328.558 15

-3 7 -2 7022.72 347.196 13

-3 7 -3 5267.29 280.736 13

-3 -7 -3 4910.27 260.693 15

-3 -7 -3 5705.20 266.610 21

-3 7 -4 3921.80 194.917 16

-3 -7 -4 3815.28 200.575 15

-3 -7 -5 6581.17 322.266 5

-3 -7 -5 7304.45 332.313 15

-3 7 -5 6933.68 326.871 16

-3 -7 -6 8062.56 378.035 15

-3 7 -6 7684.53 375.212 16

-3 -7 -6 8737.27 380.837 21

-3 -7 -6 7189.85 369.842 5

-3 7 -6 6708.83 384.465 2

-3 7 -7 942.306 97.2308 2

-3 -7 -7 947.227 81.1953 15

-3 7 -7 1081.40 89.6857 16

-3 -7 -7 1239.26 85.1098 26

-3 7 -7 1006.66 101.792 17

-3 -7 -7 1165.21 76.1973 5

-3 -7 -7 1107.16 85.8015 21

-3 7 -8 3593.08 237.724 2

-3 -7 -8 4446.81 217.520 26

-3 7 -8 4041.29 235.573 17

3 -7 8 3861.10 223.332 11

-3 -7 -8 4104.03 217.096 21

-3 -7 -8 3911.82 214.627 5

-3 7 -9 47.1803 66.8797 2

-3 -7 -9 68.7066 49.5549 26

-3 -7 -9 56.5109 56.5917 21

-3 -7 -9 68.3084 35.9292 5

-3 7 -9 51.8928 72.3556 17

-3 7 -10 1495.91 137.206 17

-3 7 -10 1339.32 141.114 2

-3 -7 -10 1454.12 110.430 15

-3 -7 -10 1424.59 110.872 26

-3 -7 -10 1488.63 110.513 21

-3 -7 -10 1598.59 111.383 5

-3 7 -11 5115.71 293.551 17

-3 7 -11 4596.18 314.505 2

-3 -7 -11 5211.03 270.515 26

-3 -7 -11 4379.89 271.245 5

-3 -7 -11 5469.10 271.667 15

-3 -7 -11 4989.26 268.912 21

-3 7 -12 1977.69 163.155 17

-3 -7 -12 1915.43 137.165 21

-3 -7 -12 1906.27 135.097 15

-3 -7 -12 2054.06 141.314 26

-3 -7 -13 2368.06 166.561 26

-3 -7 -13 2508.88 156.612 15

-3 -7 -13 2448.51 159.710 21

-3 -7 -13 2429.49 150.596 18

-3 7 -13 2489.23 150.517 24

-3 7 -13 2529.06 188.815 17

-3 -7 -13 2417.15 134.838 8

-3 -7 -14 1307.99 117.506 28

-3 -7 -14 1034.99 89.8994 18

-3 7 -14 1119.93 95.1684 24

-3 7 -14 1227.67 138.323 17

-3 -7 -14 1083.44 98.1369 15

-3 -7 -15 468.457 91.7903 28

-3 7 -15 395.870 64.2307 24

-3 7 -15 416.752 107.627 17

-3 -7 -15 564.842 74.2528 15

-3 7 -16 226.127 51.4427 24

-3 -7 -16 236.657 80.1506 28

-3 7 -16 385.747 110.136 17

-3 7 -17 834.257 73.2535 24

-3 7 -17 1003.75 122.059 17

-3 -7 -17 903.396 99.6589 28

-3 7 -18 890.168 110.651 17

-3 -7 -18 709.343 91.8596 28

3 -9 -19 167.846 70.8939 28

3 9 -19 104.751 42.3111 22

3 -9 -18 1099.41 115.951 28

3 9 -18 1159.55 83.4882 22

3 -9 -17 145.871 90.0490 28

3 9 -17 93.7594 46.7124 22

3 -9 -17 56.1130 64.3099 23

3 -9 -16 753.083 103.282 26

3 -9 -16 620.845 93.6335 23

3 9 -16 716.341 69.5462 24

3 -9 -15 135.293 86.1244 26

3 -9 -15 133.616 84.8084 23

3 9 -15 122.467 54.2166 24

3 9 -14 4044.23 240.512 17

3 -9 -14 4534.95 237.812 18

3 -9 -13 115.326 58.0407 20

3 9 -13 160.489 68.8134 17

3 -9 -13 161.505 61.2219 18

3 -9 -12 334.432 80.3371 21

3 -9 -12 461.542 67.9041 20

3 -9 -12 354.635 67.2635 18

3 9 -12 528.485 77.1678 17

3 9 -12 394.682 62.7059 24

3 -9 -11 1576.63 125.219 21

3 -9 -11 1544.39 116.327 18

3 9 -11 1353.20 114.141 17

3 -9 -11 1539.07 133.899 25

3 -9 -11 1756.74 127.908 26

3 9 -11 1512.59 107.590 24

3 9 -10 20.8530 57.3236 17

3 -9 -10 84.0876 54.8727 18

3 -9 -10 69.9862 66.9529 26

3 -9 -10 239.695 85.8257 25

3 9 -10 70.8840 48.0771 24

3 -9 -9 401.575 73.4693 26

3 9 -9 312.922 51.1502 24

3 -9 -9 346.835 86.8292 25

3 9 -9 382.846 78.3778 13

3 9 -9 404.152 64.8750 17

3 -9 -8 13168.2 619.098 25

3 -9 -8 12678.7 597.023 21

3 9 -8 12669.9 591.074 24

3 9 -8 11211.9 590.300 17

3 9 -8 12222.7 627.226 13

3 -9 -7 9354.10 464.338 21

3 9 -7 9862.41 458.742 24

3 9 -7 9416.41 494.461 13

3 -9 -7 9073.10 463.345 18

3 -9 -6 1276.44 91.5131 21

3 9 -6 1192.82 81.8501 24

-3 9 6 1067.26 91.1797 13

3 9 -6 1101.12 109.087 13

3 -9 -6 1162.27 89.2820 18

3 -9 -5 702.932 71.4478 21

3 9 -5 751.909 60.3874 24

3 9 -5 793.071 93.4282 13

-3 9 5 703.521 73.7310 13

3 -9 -5 731.750 68.5496 18

3 -9 -4 72918.2 3398.87 21

3 9 -4 74043.3 3394.59 24

3 -9 -4 74356.3 3399.51 18

-3 9 4 73831.2 3417.25 13

3 9 -4 73088.1 3434.52 13

3 -9 -3 10015.4 491.602 18

3 -9 -3 9932.43 491.597 21

-3 9 3 11313.2 516.134 13

3 -9 -2 8664.33 428.422 18

3 -9 -2 8837.37 428.952 21

3 9 -2 9255.05 468.594 13

-3 9 2 9693.36 455.390 13

3 -9 -2 8578.89 447.246 12

3 -9 -1 454.518 48.9598 18

3 -9 -1 205.344 69.4013 12

3 9 -1 360.216 77.1076 13

-3 9 1 339.580 62.9740 13

3 -9 -1 543.036 51.1013 21

3 -9 0 35139.8 1664.53 18

3 -9 0 35175.3 1663.74 21

-3 -9 0 36406.6 1671.27 21

-3 9 0 38993.8 1697.88 13

3 9 0 38329.1 1708.52 13

3 -9 0 32578.9 1715.11 12

-3 -9 0 36554.3 1668.77 15

-3 -9 -1 13579.8 616.371 15

3 -9 1 11696.1 607.247 18

-3 -9 -1 13447.2 618.744 21

3 9 1 13926.5 654.250 13

-3 9 -1 13636.2 641.465 13

-3 -9 -2 553.725 57.5414 15

-3 -9 -2 496.027 63.7176 21

3 9 2 557.144 82.2610 13

-3 9 -2 569.718 77.4734 13

-3 -9 -3 664.230 65.9666 15

-3 9 -3 768.872 87.3205 13

3 9 3 834.428 93.5758 13

-3 -9 -3 590.055 69.0696 21

-3 -9 -4 6654.58 366.314 21

-3 -9 -4 7883.01 363.497 26

-3 -9 -4 6869.95 366.598 15

-3 9 -4 7397.75 361.247 16

3 9 4 8097.36 402.450 13

-3 9 -4 7593.70 360.604 24

-3 -9 -5 99.9369 51.3705 21

-3 -9 -5 110.200 52.5636 15

-3 9 -5 85.0405 40.2620 16

-3 -9 -5 132.481 47.7321 26

3 9 5 36.3813 54.6790 13

-3 9 -5 84.5912 37.2235 24

-3 -9 -6 4887.14 252.974 15

-3 9 -6 4514.26 245.659 16

-3 9 -6 4573.80 241.304 24

-3 -9 -6 4837.77 245.729 26

3 9 6 4689.77 273.970 13

3 9 7 547.659 78.3765 13

-3 9 -7 503.471 89.4664 17

-3 -9 -7 646.524 79.3070 15

-3 9 -7 591.918 75.9622 16

-3 9 -7 790.614 67.5330 24

-3 -9 -7 663.815 71.3152 26

-3 9 -8 689.753 97.2058 17

-3 -9 -8 568.830 77.8257 15

-3 9 -8 609.123 66.9924 24

-3 -9 -8 706.358 86.1053 21

-3 -9 -8 737.052 77.2489 26

-3 9 -9 2969.02 199.021 17

-3 -9 -9 2884.50 182.535 21

-3 -9 -9 2831.32 182.478 15

-3 -9 -9 3273.91 180.919 26

-3 9 -9 3027.23 172.465 24

-3 -9 -10 267.100 74.2361 21

-3 9 -10 247.758 56.5644 24

-3 9 -10 281.885 90.6238 17

-3 -9 -10 280.637 72.9231 26

-3 -9 -11 176.957 73.5927 21

-3 9 -11 64.1023 49.5701 24

-3 -9 -11 82.4334 61.0354 18

-3 9 -11 65.3129 88.0709 17

-3 -9 -11 105.597 69.9960 26

-3 9 -12 300.210 100.824 17

-3 -9 -12 280.084 66.9718 18

-3 -9 -12 402.178 77.3566 15

-3 -9 -12 461.841 81.6700 21

-3 9 -12 365.760 65.3509 24

-3 -9 -12 328.250 80.7973 26

-3 -9 -13 3498.08 205.584 18

-3 -9 -13 3610.60 215.218 21

-3 9 -13 3572.62 230.026 17

-3 -9 -13 3774.28 214.506 15

-3 -9 -14 251.083 71.4624 28

-3 -9 -14 251.616 61.6448 18

-3 9 -14 231.674 95.9719 17

-3 9 -15 727.826 75.3370 24

-3 -9 -15 765.645 90.5248 28

-3 9 -15 944.673 115.649 17

-3 9 -16 815.625 71.9457 24

-3 -9 -16 642.724 81.4332 28

-3 9 -16 725.649 102.924 17

-3 9 -17 789.308 67.9853 24

-3 9 -17 822.430 96.9967 17

-3 -9 -17 708.185 78.3440 28

-3 9 -18 59.5097 37.5409 22

-3 9 -18 79.7702 38.1752 24

3 -11 -17 473.776 74.7533 28

3 11 -17 551.109 55.5238 22

3 -11 -16 930.450 99.8892 28

3 11 -16 994.797 75.9385 22

3 11 -15 1607.39 103.669 22

3 -11 -14 92.4274 70.5577 23

3 11 -14 89.6355 53.8154 24

3 -11 -14 77.5024 80.1650 26

3 -11 -13 3848.00 218.260 18

3 11 -13 3967.06 214.741 24

3 -11 -13 4266.71 234.608 26

3 -11 -13 3680.48 218.791 20

3 -11 -12 104.771 58.3604 18

3 11 -12 129.435 54.9554 24

3 -11 -12 147.118 78.2002 26

3 -11 -12 164.834 62.5328 20

3 -11 -11 1127.92 98.2148 18

3 11 -11 1046.82 90.8465 24

3 -11 -11 1166.49 111.160 26

3 -11 -11 1042.31 93.4279 20

3 -11 -10 1819.02 136.001 21

3 11 -10 1807.52 123.465 24

3 -11 -10 1933.70 140.912 26

3 -11 -10 1767.34 140.911 25

3 -11 -9 3698.81 212.327 21

3 -11 -9 3559.26 222.949 25

3 -11 -9 3762.58 216.037 26

3 11 -9 3587.92 200.178 24

3 -11 -9 3533.17 209.463 18

3 11 -8 14.5843 75.4518 13

3 -11 -8 25.4232 63.1378 21

3 -11 -8 68.2085 70.5791 26

3 -11 -8 36.9620 57.6190 18

3 -11 -8 70.1889 80.7355 25

3 11 -8 63.3293 47.6927 24

3 11 -7 652.734 71.4644 16

3 -11 -7 627.854 85.6984 26

3 11 -7 513.786 96.3647 13

3 -11 -7 518.991 79.0036 21

3 -11 -7 599.559 78.4495 18

3 -11 -7 689.335 105.252 25

3 11 -7 642.256 67.9925 24

3 -11 -6 645.681 82.3135 21

3 11 -6 605.061 102.144 13

3 -11 -6 607.074 76.2846 18

3 11 -6 690.668 68.0878 24

3 -11 -5 3017.67 171.553 18

-3 11 5 2748.74 182.440 13

3 11 -5 2619.17 204.193 13

3 11 -5 2969.97 162.950 24

3 -11 -5 2900.90 169.565 21

3 -11 -4 9445.18 475.805 18

-3 11 4 9833.23 501.014 13

3 11 -4 10248.4 472.803 24

3 -11 -4 9304.34 474.930 21

3 -11 -3 2123.74 133.687 18

3 -11 -3 2250.84 132.994 21

-3 -11 3 2126.67 129.348 21

-3 11 3 2148.37 155.497 13

-3 11 2 1454.20 119.588 13

3 -11 -2 1453.65 97.6813 18

-3 -11 2 1181.15 95.6193 21

3 -11 -2 1267.06 94.4976 21

3 -11 -1 22.8651 43.5215 21

-3 -11 1 32.6000 51.0765 21

3 -11 -1 47.7043 43.1551 18

-3 -11 1 30.1586 40.1676 18

3 11 -1 13.3855 68.9007 13

-3 11 1-21.1817 61.1861 13

-3 -11 0 3503.04 194.949 21

3 -11 0 3238.67 183.191 21

3 -11 0 3571.33 188.063 18

3 11 0 3829.83 242.139 13

-3 11 0 3590.89 221.216 13

-3 -11 -1 5320.24 288.466 21

-3 11 -1 5761.12 319.808 13

3 11 1 5964.79 334.037 13

-3 -11 -2 1101.67 100.161 21

-3 11 -2 1476.00 127.100 13

3 11 2 1371.08 130.664 13

3 11 3 1950.58 156.189 13

-3 11 -3 1845.78 120.661 16

-3 -11 -3 1892.01 130.647 21

-3 -11 -4 1566.87 126.455 21

-3 -11 -4 1905.23 116.438 26

3 11 4 1883.06 150.525 13

-3 11 -4 1852.73 120.359 16

-3 11 -4 1722.27 111.268 24

-3 -11 -5 2489.89 158.332 15

-3 11 -5 2403.18 147.305 16

-3 -11 -5 2218.40 153.751 21

-3 -11 -5 2450.21 143.135 26

3 11 5 2468.85 177.115 13

-3 11 -5 2331.86 137.854 24

-3 11 -5 1960.47 153.711 17

-3 11 -6 379.619 69.8178 16

-3 -11 -6 241.121 72.5728 15

-3 -11 -6 169.873 68.4753 21

-3 -11 -6 337.795 61.8212 26

-3 11 -6 268.529 75.3672 17

-3 11 -6 269.069 51.1452 24

3 11 6 309.813 73.4133 13

-3 -11 -7 2740.32 174.517 15

-3 11 -7 2435.61 173.657 16

3 -11 7 2312.32 181.000 11

-3 11 -7 2459.70 175.192 17

-3 11 -7 2859.66 158.769 24

-3 -11 -7 3077.15 166.303 26

-3 -11 -8 1232.95 98.2581 26

-3 -11 -8 918.300 100.058 18

-3 11 -8 935.504 109.265 17

-3 11 -8 1062.25 88.9733 24

-3 -11 -8 1187.96 108.319 15

-3 11 -9 142.530 78.7059 17

-3 -11 -9 170.535 76.4823 21

-3 -11 -9 182.798 69.6305 26

-3 11 -9 305.595 60.7081 24

-3 -11 -10 1799.95 138.855 21

-3 11 -10 1655.07 139.555 17

-3 -11 -10 1818.72 132.489 26

-3 11 -10 1869.56 123.392 24

-3 -11 -11 3485.18 200.978 21

-3 11 -11 2995.64 201.899 17

-3 -11 -11 3149.77 192.591 18

-3 -11 -11 3342.71 195.450 26

-3 11 -11 3300.59 188.000 24

-3 -11 -12 731.235 87.6452 18

-3 11 -12 752.878 96.8416 17

-3 11 -12 799.484 80.4879 24

-3 -11 -12 865.946 98.6220 21

-3 -11 -13 94.9657 74.4435 21

-3 11 -13 94.0360 53.4321 24

-3 -11 -13 55.7820 55.3611 18

-3 11 -13 3.58898 71.0497 17

-3 11 -14 532.036 66.5621 24

-3 -11 -14 544.180 68.8577 18

-3 11 -15 1185.29 88.6796 24

3 13 -15 127.986 38.4460 22

3 13 -14 1938.63 112.903 22

3 13 -13 873.686 67.6011 22

3 -13 -13 845.213 93.3479 26

3 -13 -12 388.163 69.2263 20

3 13 -12 354.626 49.2004 22

3 -13 -12 418.422 79.4066 26

3 -13 -11 940.636 99.0107 26

3 -13 -11 760.531 84.2741 20

3 -13 -10 2953.04 179.445 18

3 -13 -10 3345.97 186.669 26

3 -13 -10 2714.10 173.237 20

3 -13 -9 64.8399 67.5901 21

3 -13 -9 141.628 74.1423 26

3 -13 -9 86.1520 64.8695 18

3 -13 -9 81.5451 69.7933 25

3 -13 -8 346.272 74.1429 21

3 -13 -8 406.533 77.2915 26

3 -13 -8 279.566 70.8519 18

3 -13 -8 245.426 78.3262 25

3 -13 -7 96.0044 63.4713 21

3 -13 -7 72.8910 67.0625 26

3 -13 -7 26.0451 80.8685 25

3 -13 -7 96.1886 62.7713 18

3 -13 -6 117.523 64.6601 18

3 -13 -6 75.0183 63.6118 21

3 -13 -6 143.047 67.3013 26

3 -13 -6 171.508 86.0509 25

3 13 -6 102.863 63.8499 16

3 -13 -5 47.1035 61.5029 26

3 -13 -5 8.69391 58.1184 21

3 13 -5 132.163 82.2066 13

3 13 -5 161.479 59.8157 16

3 -13 -5 100.159 60.1520 18

3 -13 -4 186.743 62.7295 26

3 -13 -4 184.881 60.0997 21

3 -13 -4 252.071 62.2384 18

3 -13 -3 254.295 59.6251 21

3 -13 -3 394.785 66.8520 18

3 13 -3 368.838 93.1221 13

3 -13 -2 59.1811 48.3871 21

3 -13 -2 68.0863 53.7293 18

-3 -13 2 95.3243 52.4476 21

3 13 -2 95.3051 84.9082 13

3 -13 -1 750.613 76.2069 18

-3 -13 1 538.459 69.9754 21

3 -13 -1 540.372 65.5486 21

3 13 -1 833.767 116.625 13

-3 -13 0 144.798 54.0268 18

-3 -13 0 165.165 60.7951 21

3 13 0 107.838 78.0264 13

-3 -13 -1 308.885 68.1687 21

3 13 1 224.340 81.1782 13

-3 -13 -1 320.176 62.9351 18

-3 13 -1 377.510 65.2727 16

-3 -13 -2 2808.86 170.779 18

-3 13 -2 3145.53 174.112 16

-3 -13 -2 2571.99 170.995 21

3 13 2 2752.68 209.133 13

-3 -13 -3 209.179 70.3203 21

3 13 3 98.2843 73.8874 13

-3 -13 -3 99.2421 60.5818 18

-3 13 -3 131.729 63.6195 16

-3 -13 -4 1395.61 111.005 18

3 13 4 1226.85 129.277 13

-3 13 -4 1380.34 115.173 16

-3 -13 -4 1386.00 113.616 21

-3 13 -5 24.1660 45.4476 24

-3 -13 -5 10.1208 60.3515 18

-3 -13 -5 95.9958 73.4177 21

-3 13 -5-3.88865 65.6891 16

-3 -13 -6 4115.61 225.747 18

-3 13 -6 3737.04 212.891 24

-3 -13 -6 3943.79 226.469 21

-3 -13 -6 3773.64 212.389 26

-3 13 -6 4201.50 239.608 16

-3 -13 -7 88.2472 63.9116 18

-3 13 -7 101.990 51.5891 24

-3 -13 -7 98.5684 58.8300 26

-3 -13 -7-27.9585 72.9751 21

-3 -13 -8 673.838 84.8954 18

-3 13 -8 707.646 73.8111 24

-3 -13 -8 720.885 80.3154 26

-3 -13 -8 611.384 93.5688 21

-3 -13 -9 188.747 69.9816 18

-3 -13 -9 379.988 69.1747 26

-3 13 -9 264.387 59.2767 24

-3 13 -10 4604.90 228.600 24

-3 -13 -10 3904.08 229.329 18

-3 -13 -10 3899.47 234.401 21

-3 13 -11 163.549 54.3308 24

-3 -13 -11 148.882 69.9824 21

-3 13 -12 298.266 56.3360 24

-3 -13 -12 315.706 63.1988 18

-3 13 -13 210.943 52.7164 24

-3 -13 -13 190.309 56.7830 18

-3 13 -15 48.3263 33.9662 22

3 -15 -11 134.845 58.9964 26

3 -15 -11 140.247 57.3215 20

3 -15 -10 548.474 70.4328 26

3 -15 -10 469.335 70.9440 20

3 -15 -9 763.832 79.2218 26

-3 15 9 726.233 66.1081 16

3 -15 -8 881.143 80.7084 26

3 -15 -8 619.424 76.5594 20

-3 15 8 809.932 71.2112 16

3 -15 -7 125.759 58.8502 26

3 -15 -7-18.8449 54.3679 20

-3 15 7 16.2639 47.6261 16

3 -15 -6 698.390 77.0309 26

-3 15 6 846.276 74.9985 16

3 -15 -5 474.057 67.8835 20

3 -15 -5 488.244 68.0800 26

3 -15 -5 441.755 81.8561 25

-3 15 5 622.818 72.2086 16

3 -15 -4 1090.39 90.3960 20

3 -15 -4 884.796 106.599 25

-3 15 4 1193.69 93.0833 16

-3 15 3 285.770 63.1156 16

3 15 -3 332.752 75.2514 16

3 -15 -3 259.180 58.6318 20

-3 15 2 517.558 72.6426 16

3 15 -2 541.383 79.2935 16

3 -15 -2 402.141 62.8840 20

3 -15 -1 264.492 54.5420 20

-3 -15 0 164.531 55.7192 21

3 -15 0 159.852 50.9110 20

-3 15 -1 385.292 76.5575 16

-3 -15 -1 320.878 64.5951 21

3 -15 1 266.046 55.9325 20

-3 15 -2 121.095 68.8979 16

-3 -15 -2 102.088 59.6095 21

-3 -15 -2 33.7684 59.3162 18

-3 15 -3 532.501 89.0444 16

-3 -15 -3 388.483 72.2708 21

-3 -15 -3 401.625 73.6897 18

-3 15 -4 89.2685 78.5125 16

-3 -15 -4 74.0319 61.5993 21

-3 -15 -4 94.5701 59.0588 18

-3 -15 -5 1350.26 109.749 21

-3 -15 -5 1309.20 107.967 18

-3 -15 -6 103.397 65.1313 21

-3 -15 -6 160.058 64.5580 18

-3 -15 -7 1029.25 96.1096 21

-3 -15 -7 1046.26 94.2611 18

-3 -15 -8 102.622 58.7822 18

-3 -15 -8 90.2662 63.6926 21

-3 -15 -9 107.079 59.0729 18

-3 -15 -9 190.495 65.7960 21

-3 -15 -10 133.745 56.6593 18

-3 -15 -10 56.4239 49.1503 20

-3 17 5 199.715 59.3772 16

-3 17 4 300.925 65.3751 16

-3 17 3 754.075 85.2509 16

-3 17 1 88.9427 71.2341 16

-3 17 0 860.756 100.096 16

3 17 0 718.196 89.6710 16

-3 17 -1 394.315 88.2540 16

3 17 1 430.968 74.3466 16

3 17 2 431.121 70.5155 16

-3 -17 -2 104.156 54.0918 20

-3 -17 -3 46.6055 50.3345 20

3 17 4 304.026 64.8241 16

4 0 -22 206.383 45.0101 8

4 0 -21 63.2669 45.0832 8

4 0 -20 123.850 49.0415 8

4 0 -20 104.996 133.907 28

4 0 -19-2.45720 46.7303 8

4 0 -19 29.0597 133.812 28

4 0 -19-34.8946 134.579 23

4 0 -18 217.437 77.6604 7

4 0 -18 35.5780 47.0045 8

4 0 -18-38.0856 132.320 28

4 0 -18 99.5436 137.651 23

4 0 -17-39.2500 138.831 23

4 0 -17-45.5221 133.523 28

4 0 -17 14.1574 60.4185 7

4 0 -17 5.08047 46.0842 8

4 0 -17-58.3880 69.1088 5

4 0 -16 6898.40 445.808 23

4 0 -16 8569.62 503.464 2

4 0 -16 6961.16 437.896 28

4 0 -16 7257.76 391.637 7

4 0 -16 7274.39 394.638 9

4 0 -16 8446.17 385.526 8

4 0 -16 8474.49 436.758 5

4 0 -15 50.4014 127.967 23

4 0 -15 141.823 139.635 2

4 0 -15 106.937 123.298 28

4 0 -15 57.4815 64.4841 5

4 0 -15 86.0712 62.7626 9

4 0 -15-19.0922 53.6697 7

4 0 -15 11.4412 43.1752 8

4 0 -14 2240.60 145.010 7

4 0 -14 2595.16 176.024 5

4 0 -14 2213.33 148.774 9

4 0 -14 2797.34 242.363 2

4 0 -14 1886.39 197.922 23

4 0 -14 2616.58 136.675 8

4 0 -14 1942.50 167.083 17

4 0 -13 52.0674 74.5337 17

4 0 -13 20.5515 48.4039 7

4 0 -13 32.4327 29.5162 22

4 0 -13 31.4410 57.7256 5

4 0 -13 52.4534 53.9901 9

4 0 -13 68.2127 118.860 2

4 0 -13 71.2691 106.071 23

4 0 -13 45.1142 36.6378 8

4 0 -13 47.2206 78.8387 4

4 0 -12 3892.78 219.080 7

4 0 -12 4340.69 261.833 23

4 0 -12 4262.98 213.182 8

4 0 -12 4274.53 234.331 17

4 0 -12 4191.62 215.760 6

4 0 -12 4407.31 247.825 5

4 0 -12 4016.13 260.754 4

4 0 -12 4131.13 222.583 9

4 0 -12 4469.79 297.484 2

4 0 -11-15.0345 103.480 2

4 0 -11 10.0640 32.0782 6

4 0 -11 8.05868 46.5493 5

4 0 -11-15.8649 67.3067 4

4 0 -11-11.2175 60.6435 17

4 0 -10 755.794 117.312 2

4 0 -10 769.682 89.1502 4

4 0 -10 731.883 74.7148 5

4 0 -10 595.751 52.0082 6

4 0 -9 77.5067 83.2672 2

4 0 -9 26.9793 42.1602 5

4 0 -9 27.9211 27.7377 6

4 0 -9-4.01125 52.4913 4

4 0 -8 18683.9 885.555 5

4 0 -8 18343.5 894.743 4

4 0 -8 19169.0 913.863 2

4 0 -7 47.9165 65.2487 2

4 0 -7 17.2990 30.1886 5

4 0 -7 38.4294 48.2193 4

4 0 -6 8389.24 416.980 4

4 0 -6 8238.02 408.539 5

4 0 -6 9075.17 430.620 2

4 0 -5 104.647 52.8684 2

4 0 -5 8.76589 36.0499 4

4 0 -5 77.5866 25.4802 5

4 0 -4 1776.44 100.279 3

4 0 -4 1819.07 111.939 2

4 0 -3 111.711 71.5529 11

4 0 -3 46.3534 36.0259 3

4 0 -3 187.483 47.1939 12

4 0 -2 107537. 5344.42 3

4 0 -2 118049. 5350.31 12

4 0 -1 84.5744 44.4457 3

4 0 -1 16.6218 55.3444 12

4 0 0 6875.58 367.542 11

4 0 1 56.8571 86.8630 11

4 0 2 2935.99 188.906 11

-4 0 -3 59.0586 66.7807 1

4 0 3 122.270 63.7369 11

-4 0 -4 145797. 6784.41 1

-4 0 -4 148912. 6780.94 2

-4 0 -5 32.2328 55.2199 2

-4 0 -5 3.54918 63.4862 1

-4 0 -6 29274.3 1410.99 1

-4 0 -6 30125.4 1407.35 2

-4 0 -7-49.2973 75.1675 2

-4 0 -7-19.4347 86.0825 1

-4 0 -8 5915.68 316.099 5

-4 0 -8 5889.84 349.944 1

-4 0 -8 6861.73 317.481 17

-4 0 -8 5533.86 345.013 2

-4 0 -9 16.8774 45.0782 5

-4 0 -9 31.8504 96.8758 1

-4 0 -9 42.3866 96.6403 2

-4 0 -9 2.80359 59.5659 17

-4 0 -10 13183.9 641.022 5

-4 0 -10 12489.5 675.140 2

-4 0 -10 12861.8 632.604 17

-4 0 -10 13275.8 680.511 1

-4 0 -11 57.5304 71.4874 28

-4 0 -11 9.29993 53.3081 5

-4 0 -11 3.47779 62.6968 23

-4 0 -11 3.69176 33.2259 8

-4 0 -11-7.42504 74.1921 17

-4 0 -11-7.79674 37.0178 10

-4 0 -12 4685.65 291.899 28

-4 0 -12 5135.16 295.055 17

-4 0 -12 4942.38 297.137 5

-4 0 -12 5234.38 347.426 2

-4 0 -12 5588.35 267.788 10

-4 0 -12 5441.00 265.459 8

-4 0 -12 4856.70 284.855 23

-4 0 -13-9.83202 92.8386 28

-4 0 -13 62.9758 70.2846 23

-4 0 -13 22.2827 38.5846 8

-4 0 -13 115.421 127.097 2

-4 0 -13 52.5333 88.3685 17

-4 0 -13 34.9804 59.4447 5

-4 0 -13 9.44032 42.3646 10

-4 0 -14 43.4680 102.918 28

-4 0 -14 126.055 84.2977 23

-4 0 -14 197.258 48.3801 8

-4 0 -14 137.264 122.996 2

-4 0 -14 121.561 102.376 17

-4 0 -14 233.859 75.3755 5

-4 0 -14 194.378 48.7462 10

-4 0 -15 45.0893 47.9666 10

-4 0 -15 10.1501 47.0204 8

-4 0 -15-24.7698 143.623 2

-4 0 -15-102.202 111.276 17

-4 0 -15 6.81315 63.4031 5

-4 0 -15-23.4368 104.964 28

-4 0 -16 724.320 71.6285 10

-4 0 -16 590.731 133.550 17

-4 0 -16 516.145 127.668 28

-4 0 -16 758.672 72.2232 8

-4 0 -16 614.431 165.989 2

-4 0 -16 488.441 92.9509 5

-4 0 -17 35.7325 50.6250 8

-4 0 -17-41.6943 119.944 17

-4 0 -18 637.341 69.5873 8

-4 0 -19-13.3233 42.9417 8

-4 0 -20 107.400 42.8267 8

-4 0 -21 7.57641 35.0792 8

4 -2 -22 48.8238 39.0253 8

4 2 -22-1.62977 37.5983 8

4 -2 -21 124.994 118.254 28

4 -2 -21 226.570 46.8444 8

4 2 -21 171.036 47.4164 8

4 -2 -20 917.413 151.689 28

4 -2 -20 923.106 77.4106 8

4 2 -20 1047.16 79.2638 8

4 -2 -19-77.6592 121.699 28

4 -2 -19 119.461 114.955 23

4 -2 -19 23.9720 47.2172 8

4 2 -19 78.9971 48.1317 8

4 2 -18-42.6366 152.472 28

4 -2 -18 149.175 129.833 28

4 -2 -18 129.051 125.452 23

4 -2 -18 185.531 52.7025 8

4 -2 -18 643.047 94.0174 7

4 2 -18 616.201 88.5300 7

4 2 -18 150.900 52.8802 8

4 2 -17 302.601 162.157 23

4 -2 -17 382.656 133.926 23

4 2 -17 414.460 58.0474 22

4 2 -17 503.851 97.4918 5

4 -2 -17 503.294 95.4781 5

4 -2 -17 452.907 80.1171 7

4 2 -17 564.893 84.8781 7

4 2 -17 544.581 63.5036 8

4 -2 -17 536.182 63.5592 8

4 -2 -17 304.718 125.703 28

4 2 -17 390.374 159.842 28

4 2 -16 9719.62 581.557 28

4 2 -16 10912.4 593.075 23

4 -2 -16 10704.8 565.706 23

4 -2 -16 10187.8 617.857 2

4 2 -16 10129.1 619.132 2

4 2 -16 9836.03 523.494 9

4 -2 -16 10753.3 525.444 9

4 -2 -16 10260.7 520.048 7

4 2 -16 10141.3 520.251 7

4 2 -16 11230.0 561.242 5

4 -2 -16 10614.5 556.774 5

4 2 -16 11027.5 510.026 8

4 -2 -16 11063.4 510.051 8

4 2 -16 9963.02 505.579 22

4 2 -15 2026.06 137.030 7

4 -2 -15 1995.08 135.618 7

4 -2 -15 2292.28 164.569 5

4 2 -15 1795.26 155.392 5

4 2 -15 1946.46 225.553 2

4 -2 -15 1987.85 221.914 2

4 2 -15 1464.23 197.384 28

4 -2 -15 1899.18 184.538 23

4 2 -15 1818.54 138.626 9

4 -2 -15 1860.45 134.803 9

4 -2 -15 2093.90 121.482 8

4 2 -15 2167.57 122.763 8

4 -2 -14 668.719 75.8619 7

4 2 -14 641.606 74.0662 7

4 -2 -14 822.719 99.3432 5

4 2 -14 904.608 101.031 5

4 2 -14 809.706 151.981 2

4 -2 -14 778.774 150.645 2

4 2 -14 552.461 144.190 28

4 -2 -14 496.970 94.1110 27

4 2 -14 587.165 56.7614 24

4 -2 -14 606.663 123.480 23

4 -2 -14 670.627 80.1560 9

4 2 -14 702.208 80.2005 9

4 -2 -14 822.982 65.6042 8

4 2 -14 856.384 66.2209 8

4 -2 -14 539.630 88.9472 26

4 2 -13 6408.62 340.311 17

4 -2 -13 6276.89 326.304 7

4 2 -13 5965.85 325.559 7

4 -2 -13 6648.68 355.000 5

4 2 -13 5697.81 367.328 4

4 -2 -13 6093.03 368.924 4

4 2 -13 7357.93 360.839 5

4 2 -13 6955.16 412.839 2

4 -2 -13 6234.45 404.999 2

4 2 -13 5608.69 369.573 28

4 -2 -13 6671.28 340.026 27

4 2 -13 6702.43 341.504 27

4 -2 -13 6543.54 366.952 23

4 2 -13 6004.21 313.239 24

4 -2 -13 6515.28 329.891 9

4 2 -13 6163.39 328.382 9

4 -2 -13 6277.12 333.875 26

4 2 -13 7239.65 320.129 8

4 -2 -13 6813.83 318.503 8

4 2 -12 1371.63 108.084 17

4 -2 -12 1232.25 81.4103 8

4 2 -12 1359.35 82.5557 8

4 2 -12 1241.77 94.8067 9

4 -2 -12 1260.63 93.6559 9

4 -2 -12 1455.74 164.734 2

4 2 -12 1331.61 158.633 2

4 2 -12 1284.55 111.523 5

4 -2 -12 1315.66 113.761 5

4 2 -12 1240.20 128.297 4

4 -2 -12 1232.82 129.883 4

4 -2 -12 1429.70 106.841 27

4 2 -12 1499.97 108.413 27

4 2 -12 1329.70 139.068 28

4 -2 -12 1189.46 91.7133 7

4 2 -12 1106.27 90.3294 7

4 2 -11 10031.6 497.464 17

4 -2 -11 11539.8 563.433 2

4 2 -11 10359.5 555.374 2

4 -2 -11 9838.61 483.718 6

4 2 -11 10004.2 484.350 6

4 2 -11 11099.2 514.665 5

4 -2 -11 11259.7 514.271 5

4 2 -11 8810.93 520.486 4

4 -2 -11 9613.00 523.729 4

4 2 -10 1126.01 80.3908 17

4 2 -10 965.905 129.984 2

4 -2 -10 972.296 127.498 2

4 2 -10 659.531 56.4071 6

4 -2 -10 718.398 57.1649 6

4 2 -10 646.503 74.9142 5

4 -2 -10 886.608 79.4599 5

4 2 -10 834.579 98.3537 4

4 -2 -10 915.324 98.8486 4

4 -2 -9 1339.55 82.5292 6

4 2 -9 1343.01 82.8276 6

4 -2 -9 1268.36 139.553 2

4 2 -9 1418.43 142.509 2

4 -2 -9 1497.73 120.990 4

4 2 -9 1443.74 117.476 4

4 -2 -9 1531.49 103.518 5

4 2 -9 1306.45 100.645 5

4 2 -8 2934.88 187.142 2

4 -2 -8 2782.21 181.354 2

4 2 -8 3279.49 155.954 5

4 2 -8 2484.25 161.393 4

4 -2 -8 2656.09 160.741 4

4 -2 -8 3161.78 151.826 5

4 2 -7 3349.12 207.004 2

4 -2 -7 3667.82 211.538 2

4 2 -7 3573.12 181.192 5

4 -2 -7 3443.43 180.008 5

4 2 -7 3397.91 191.621 4

4 -2 -7 3466.46 192.314 4

4 2 -7 3214.43 168.137 13

4 -2 -7 3023.71 194.951 11

4 2 -6 5715.68 293.268 2

4 -2 -6 5421.28 291.880 2

4 2 -6 5128.49 262.484 13

4 -2 -6 6178.15 277.130 11

4 2 -6 5364.89 280.625 4

4 -2 -6 5670.31 282.239 4

4 2 -6 5388.44 271.071 5

4 -2 -6 5295.10 271.246 5

4 2 -5 283.235 65.9564 2

4 -2 -5 367.921 58.5710 2

4 2 -5 337.103 38.3037 5

4 -2 -5 271.275 34.7726 5

4 2 -5 371.796 32.9736 13

4 -2 -5 457.378 39.6703 11

4 2 -5 241.859 48.3905 4

4 -2 -5 316.775 49.1840 4

4 2 -4 67421.2 3280.68 2

4 2 -4 71330.7 3269.46 5

4 -2 -4 70389.7 3268.84 5

4 2 -4 69848.1 3275.72 4

4 -2 -4 72786.0 3276.94 4

4 -2 -4 70163.8 3273.95 3

4 2 -4 72255.4 3274.55 3

4 -2 -4 73201.4 3269.34 11

4 2 -3 16183.2 736.202 3

4 -2 -3 15339.7 735.022 3

4 -2 -3 15559.5 732.748 11

4 -2 -2 204681. 9495.65 3

4 2 -2 219482. 9497.32 3

4 -2 -2 199940. 9491.95 11

4 2 -1 5330.71 271.620 3

4 -2 -1 4816.72 268.324 3

4 2 -1 5785.18 259.603 12

4 -2 0 42055.8 1958.27 11

4 2 0 42759.1 1955.22 12

4 -2 1 112469. 5164.04 11

4 -2 2 253872. 11580.7 11

-4 2 -2 243730. 11588.9 1

-4 -2 -2 259967. 11590.1 1

4 -2 3 57562.2 2551.50 11

-4 2 -3 51358.0 2559.65 1

-4 -2 -3 56100.1 2562.39 1

-4 2 -4 76279.4 3649.48 2

-4 -2 -4 79428.7 3650.94 2

4 -2 4 84921.9 3646.24 11

-4 2 -4 74858.8 3652.66 1

-4 -2 -4 78578.8 3653.22 1

-4 -2 -5 825.277 85.7220 2

-4 2 -5 803.743 83.4344 2

4 -2 5 1013.99 91.2750 11

-4 -2 -5 644.021 87.9194 1

-4 2 -5 727.312 85.7805 1

-4 2 -6 14604.3 721.607 2

-4 -2 -6 15399.1 724.006 2

4 -2 6 13617.9 733.159 11

-4 2 -6 14578.7 726.836 1

-4 -2 -6 15408.3 729.055 1

-4 -2 -7 23726.6 1170.75 2

-4 2 -7 22565.8 1168.07 2

-4 -2 -7 23399.5 1174.52 1

-4 2 -7 22706.6 1172.57 1

-4 2 -8 3962.94 214.822 17

-4 2 -8 3795.05 209.770 5

-4 -2 -8 3823.88 211.199 5

-4 2 -8 3607.50 241.656 1

-4 -2 -8 4050.81 247.402 1

-4 -2 -8 3755.07 240.566 2

-4 2 -8 3606.36 237.493 2

-4 2 -9 1517.08 121.490 17

-4 -2 -9 1654.69 120.009 17

-4 2 -9 1784.33 117.261 5

-4 -2 -9 1553.29 113.057 5

-4 2 -9 1702.14 160.669 1

-4 -2 -9 1721.57 161.121 1

-4 2 -9 1498.58 150.543 2

-4 -2 -9 1521.18 153.792 2

-4 -2 -10 114.206 73.6324 17

-4 2 -10 125.325 76.4701 17

-4 2 -10 80.1269 46.8918 5

-4 -2 -10 4.03296 46.8498 5

-4 -2 -10 94.3639 91.5513 2

-4 -2 -10-7.43165 99.2692 1

-4 2 -10 37.1711 92.5536 1

-4 2 -10 65.1243 91.5567 2

-4 -2 -11 3068.01 250.591 2

-4 2 -11 3031.82 248.471 2

-4 2 -11 3607.05 183.235 10

-4 -2 -11 3581.76 183.694 10

-4 2 -11 3329.14 210.170 5

-4 -2 -11 3285.62 210.030 5

-4 2 -11 3608.92 182.195 8

-4 -2 -11 3626.97 182.385 8

-4 -2 -11 3597.25 213.341 17

-4 2 -11 3334.94 211.909 17

-4 -2 -11 2913.69 199.217 28

-4 -2 -12 387.035 127.765 2

-4 2 -12 423.057 124.388 2

-4 -2 -12 383.770 51.5931 10

-4 2 -12 359.184 50.3195 10

-4 -2 -12 277.295 68.1690 23

-4 2 -12 332.536 70.8031 5

-4 -2 -12 360.832 72.0154 5

-4 -2 -12 343.003 47.3270 8

-4 2 -12 312.591 48.2777 8

-4 -2 -12 350.843 82.2966 28

-4 2 -12 332.267 93.3434 17

-4 -2 -12 485.764 103.224 17

-4 -2 -13 1034.52 124.412 17

-4 2 -13 876.798 124.660 17

-4 2 -13 936.969 160.964 2

-4 -2 -13 857.582 163.693 2

-4 2 -13 1072.46 80.0800 10

-4 -2 -13 1098.80 80.0587 10

-4 -2 -13 1006.00 104.148 23

-4 -2 -13 870.464 78.5238 15

-4 2 -13 1130.40 79.0437 8

-4 -2 -13 1018.14 78.2605 8

-4 2 -13 1035.23 108.659 5

-4 -2 -13 937.606 107.806 5

-4 -2 -13 1068.52 116.961 28

-4 2 -14 106.626 108.981 17

-4 -2 -14 39.0845 106.057 17

-4 -2 -14-11.3760 121.537 2

-4 2 -14 124.517 117.096 2

-4 -2 -14 79.5505 44.3015 10

-4 2 -14 89.2444 45.2977 10

-4 -2 -14-4.03463 69.9776 23

-4 -2 -14 43.3992 42.4850 15

-4 -2 -14 33.3134 43.8144 8

-4 2 -14-2.36552 42.8976 8

-4 2 -14 62.3248 64.1888 5

-4 -2 -14 25.0829 63.0124 5

-4 -2 -14 133.718 91.6108 28

-4 -2 -15 1721.19 165.562 28

-4 -2 -15 1814.16 144.209 23

-4 2 -15 1580.35 114.027 24

-4 2 -15 2002.95 162.905 5

-4 -2 -15 1783.21 159.044 5

-4 2 -15 1737.53 217.024 2

-4 -2 -15 1929.94 220.411 2

-4 2 -15 2133.29 177.379 17

-4 2 -15 1995.66 123.564 8

-4 -2 -15 2016.65 124.311 8

-4 -2 -15 2026.92 124.331 10

-4 2 -15 2281.19 127.091 10

-4 -2 -16 76.0248 46.9055 10

-4 2 -16 170.031 50.6666 10

-4 2 -16 100.766 47.1200 8

-4 -2 -16 118.983 48.5756 8

-4 2 -16 51.4849 129.325 2

-4 -2 -16-51.7656 134.544 2

-4 -2 -16 100.313 104.229 28

-4 2 -16 151.625 115.415 17

-4 -2 -16 42.3419 65.0135 5

-4 2 -16-7.01945 71.3138 5

-4 2 -17 683.294 129.591 17

-4 -2 -17 427.777 119.949 28

-4 -2 -17 661.913 67.2848 8

-4 2 -17 615.628 68.4014 8

-4 2 -18 5.45159 111.069 17

-4 -2 -18 5.00882 102.183 28

-4 2 -18 127.219 49.7836 8

-4 -2 -18 66.2488 44.4404 8

-4 -2 -19 19.2037 40.8741 8

-4 2 -19 21.3189 43.5277 8

-4 -2 -20 12.6930 39.5296 8

-4 2 -20 43.6172 41.3639 8

-4 -2 -21 111.445 36.3384 8

-4 2 -21 200.868 38.6851 8

4 4 -21 842.424 67.6683 8

4 4 -20 48.0827 42.8574 8

4 -4 -20 77.8899 42.5811 8

4 -4 -20 84.5210 93.6953 23

4 -4 -19 1437.62 95.6662 8

4 4 -19 1419.03 95.5625 8

4 4 -18-2.47503 44.0631 22

4 4 -18 41.9339 45.0544 8

4 -4 -18 2.32098 43.0773 8

4 -4 -18 117.451 114.174 23

4 4 -17 94.5031 43.9862 8

4 -4 -17 588.459 84.5150 7

4 -4 -17 70.4509 44.3642 8

4 4 -17 642.820 88.9674 7

4 4 -17 62.0698 41.4441 24

4 -4 -17 104.984 114.413 23

4 4 -17 66.9744 90.9167 17

4 4 -17 69.4403 46.1335 22

4 -4 -16 37.2628 108.212 23

4 4 -16-6.80320 41.1379 24

4 4 -16-33.9043 66.3581 5

4 -4 -16 40.3706 62.9809 5

4 4 -16 16.2785 42.6405 8

4 -4 -16-11.5247 41.3573 8

4 -4 -16 26.8563 57.7033 7

4 4 -16 3.45342 60.7759 7

4 4 -16 41.7576 93.0755 17

4 -4 -15 5811.79 291.357 7

4 4 -15 5550.45 290.556 7

4 4 -15 5513.02 322.348 5

4 -4 -15 5659.12 322.796 5

4 4 -15 4990.49 275.444 24

4 -4 -15 5661.61 329.052 23

4 -4 -15 5607.21 294.639 9

4 4 -15 5294.44 293.020 9

4 -4 -15 5530.44 277.310 8

4 4 -15 5760.15 279.030 8

4 4 -15 5521.57 307.170 17

4 -4 -15 4857.69 298.680 26

4 -4 -14 63.8312 100.996 23

4 4 -14 34.9177 64.7035 5

4 -4 -14 22.9389 59.1272 5

4 4 -14 79.1699 147.011 28

4 -4 -14 127.007 75.4501 27

4 -4 -14 116.075 59.9526 7

4 4 -14 51.7485 58.3143 7

4 -4 -14 153.089 73.4753 26

4 -4 -14 77.6317 57.8616 9

4 4 -14 105.084 63.0686 9

4 4 -14 45.5716 39.0950 8

4 -4 -14 120.345 39.4797 8

4 4 -14 57.1083 79.0566 17

4 4 -14-106.402 138.829 2

4 -4 -14 105.577 124.777 2

4 4 -14 48.2471 40.5359 24

4 4 -14 206.524 153.043 23

4 4 -13 7195.35 379.014 5

4 -4 -13 6606.58 374.580 5

4 -4 -13 8254.26 369.410 27

4 4 -13 6555.74 349.655 7

4 -4 -13 6948.92 350.655 7

4 -4 -13 7554.19 362.251 26

4 4 -13 7034.16 353.913 9

4 -4 -13 7236.14 355.956 9

4 4 -13 7450.25 341.948 8

4 -4 -13 7129.29 340.890 8

4 4 -13 6879.81 364.261 17

4 -4 -13 6945.61 343.716 18

4 4 -13 6373.26 419.011 2

4 -4 -13 7063.04 430.530 2

4 4 -13 6386.35 417.168 23

4 4 -13 6835.91 342.864 24

4 4 -12 1317.66 106.546 5

4 4 -12 1041.72 119.244 4

4 -4 -12 1010.66 122.798 4

4 -4 -12 1188.56 105.368 5

4 -4 -12 1166.60 98.6369 27

4 4 -12 1076.07 101.793 17

4 -4 -12 1036.28 77.4417 18

4 -4 -12 1169.52 86.8550 7

4 4 -12 1149.24 85.9438 7

4 -4 -12 1103.40 93.9631 26

4 -4 -12 1222.31 88.9853 9

4 4 -12 962.576 86.1403 9

4 4 -12 1203.62 74.8150 8

4 -4 -12 1142.14 74.2960 8

4 -4 -12 1070.04 151.316 2

4 4 -12 1129.24 151.354 2

4 4 -12 1001.67 76.6562 24

4 4 -12 1018.65 156.315 23

4 -4 -11 22051.8 1051.66 20

4 4 -11 20722.2 1090.62 4

4 -4 -11 21837.7 1091.98 4

4 4 -11 23594.5 1079.50 5

4 -4 -11 21428.0 1075.20 5

4 -4 -11 24500.4 1064.99 27

4 -4 -11 21774.0 1050.49 18

4 4 -11 22623.9 1064.92 17

4 -4 -11 22564.2 1054.56 7

4 4 -11 21741.0 1054.39 7

4 -4 -11 22262.6 1116.88 2

4 4 -11 21865.4 1114.79 2

4 -4 -11 22738.0 1059.74 26

4 4 -11 21748.2 1055.80 9

4 -4 -11 22979.0 1057.06 9

4 4 -11 21168.5 1105.42 23

4 4 -11 23008.0 1051.20 24

4 4 -11 23609.6 1048.03 8

4 -4 -11 22598.5 1047.21 8

4 4 -10 9645.68 527.957 2

4 -4 -10 9895.24 530.568 2

4 4 -10 9244.97 505.353 4

4 -4 -10 9998.57 509.621 4

4 4 -10 9912.93 493.699 5

4 -4 -10 9196.95 490.598 5

4 -4 -10 9831.41 471.280 20

4 -4 -10 10311.1 472.780 18

4 4 -10 10073.1 484.518 17

4 4 -10 9809.72 470.379 6

4 -4 -10 9954.85 470.744 6

4 -4 -9 212.327 38.2357 18

4 4 -9 206.876 53.6076 17

4 -4 -9 226.780 43.0061 15

4 -4 -9 205.848 45.8310 11

4 4 -9 150.585 86.4284 2

4 -4 -9 249.277 88.1791 2

4 4 -9 285.840 52.2476 5

4 -4 -9 261.756 51.5520 5

4 4 -9 352.257 37.9887 6

4 -4 -9 410.994 37.4720 6

4 -4 -9 321.327 69.5482 4

4 4 -9 390.464 71.4730 4

4 -4 -9 171.309 45.7045 21

4 4 -8 8267.59 400.172 17

4 4 -8 7738.99 401.681 13

4 -4 -8 7736.15 398.857 11

4 -4 -8 7999.78 394.246 15

4 -4 -8 7968.95 395.380 21

4 -4 -8 8219.34 393.352 18

4 4 -8 8645.74 437.193 2

4 -4 -8 8421.82 434.861 2

4 4 -8 9362.68 411.397 5

4 -4 -8 8778.87 409.129 5

4 -4 -8 8264.49 420.255 4

4 4 -8 8248.20 419.590 4

4 -4 -7 12312.4 607.366 11

4 4 -7 11555.4 608.533 13

4 4 -7 12653.4 634.855 2

4 -4 -7 12899.6 635.202 2

4 -4 -7 13345.2 613.684 5

4 4 -7 12859.9 622.635 4

4 -4 -7 12913.0 622.153 4

4 4 -7 14315.3 615.203 5

4 4 -6 9153.48 469.074 13

-4 4 6 9659.98 455.277 13

4 4 -6 9645.38 488.672 2

4 -4 -6 9972.82 488.268 2

4 -4 -6 9987.63 467.853 5

4 4 -6 9699.69 477.103 4

4 -4 -6 9869.54 476.547 4

4 4 -6 9935.93 468.915 5

-4 4 5 3706.71 176.805 13

4 -4 -5 3535.69 199.712 12

4 4 -5 3535.92 188.617 13

4 -4 -5 3561.44 183.661 5

4 4 -5 3366.65 183.572 5

4 4 -5 3516.11 190.328 4

4 -4 -5 3647.79 190.898 4

4 4 -4 29004.4 1369.24 13

4 4 -4 28861.3 1365.08 4

4 -4 -4 28783.6 1365.06 4

-4 4 4 30867.0 1358.74 13

4 -4 -4 28064.3 1377.47 12

4 4 -3 31279.8 1432.47 3

4 4 -3 29391.8 1433.11 4

4 -4 -3 30633.9 1433.76 4

-4 4 3 32218.9 1431.02 13

4 -4 -3 30739.5 1456.12 12

4 4 -2 152858. 7162.24 4

4 -4 -2 152903. 7159.35 3

4 4 -2 158837. 7160.62 3

-4 4 2 164788. 7163.12 13

4 -4 -2 144588. 7192.12 12

4 -4 -1 9725.18 466.682 3

4 4 -1 10262.3 468.691 3

-4 4 1 9566.80 469.481 13

4 4 0 9724.60 463.809 3

4 -4 0 8503.39 459.357 3

-4 4 -1 117742. 5698.00 1

-4 -4 -1 125491. 5698.35 1

-4 4 -1 129232. 5699.19 13

-4 4 -2 77402.4 3649.64 1

-4 -4 -2 80988.9 3649.82 1

-4 -4 -3 1799.09 110.785 2

-4 4 -3 1648.16 107.499 2

-4 -4 -3 1795.31 115.108 1

-4 4 -3 1660.88 112.909 1

-4 4 -4 403.378 70.1922 1

-4 -4 -4 469.388 65.4749 2

-4 4 -4 465.261 63.4565 2

-4 -4 -4 495.665 73.9037 1

-4 4 -5 16928.7 842.194 2

-4 -4 -5 17805.2 843.510 2

-4 4 -5 17090.0 846.501 1

-4 -4 -5 18259.4 847.331 1

4 -4 5 17726.6 829.702 11

4 -4 6 30370.4 1403.29 11

-4 -4 -6 30194.5 1420.35 2

-4 4 -6 28711.1 1418.16 2

-4 4 -6 30728.4 1400.79 5

-4 4 -6 28888.4 1421.77 1

-4 -4 -6 30663.0 1423.43 1

4 -4 7 2554.85 157.037 11

-4 4 -7 2503.85 146.714 5

-4 -4 -7 2591.14 146.535 5

-4 4 -7 2650.01 181.968 1

-4 -4 -7 2303.78 177.379 1

-4 4 -7 2489.00 172.165 2

-4 -4 -7 2570.39 173.680 2

-4 4 -8 10514.8 537.530 2

-4 -4 -8 10748.6 538.948 2

-4 4 -8 10634.3 506.504 5

-4 -4 -8 10610.3 506.093 5

-4 4 -8 10279.3 512.318 17

-4 4 -8 10174.9 535.030 1

-4 -4 -8 10239.7 536.975 1

-4 -4 -8 9786.05 489.999 21

-4 4 -9 19129.6 957.403 2

-4 -4 -9 19084.3 959.857 2

-4 4 -9 19392.0 928.870 17

-4 -4 -9 18405.0 905.242 21

-4 4 -9 20395.3 928.710 5

-4 -4 -9 20735.0 928.297 5

-4 4 -9 18854.4 958.519 1

-4 -4 -10 315.158 104.546 2

-4 4 -10 526.796 113.630 2

-4 -4 -10 398.623 54.9367 21

-4 4 -10 514.087 46.2480 8

-4 -4 -10 485.435 47.4589 8

-4 4 -10 346.778 88.3285 17

-4 -4 -10 509.757 48.1187 10

-4 4 -10 517.040 48.4901 10

-4 4 -10 509.871 66.4385 5

-4 -4 -10 423.866 62.9531 5

-4 4 -11 160.082 55.3611 5

-4 -4 -11 165.803 55.5452 5

-4 4 -11 181.812 99.7005 2

-4 -4 -11 207.185 103.757 2

-4 -4 -11 78.2547 46.7828 21

-4 4 -11 216.229 37.1687 8

-4 -4 -11 189.135 36.2186 8

-4 -4 -11 85.8577 48.2400 15

-4 -4 -11 179.360 39.5837 10

-4 4 -11 198.352 39.0970 10

-4 4 -11 165.217 87.7810 17

-4 4 -12 2296.93 170.670 5

-4 -4 -12 2532.79 174.535 5

-4 4 -12 2526.62 189.483 17

-4 4 -12 2792.63 143.661 8

-4 -4 -12 2637.69 142.730 8

-4 -4 -12 2087.33 147.252 15

-4 -4 -12 2706.56 143.679 10

-4 4 -12 2695.07 143.905 10

-4 -4 -12 2199.69 219.613 2

-4 4 -12 2209.86 216.013 2

-4 4 -13 103.844 103.928 17

-4 4 -13 81.0551 113.910 2

-4 -4 -13-20.3578 107.298 2

-4 4 -13 46.7805 40.6851 10

-4 -4 -13 93.5340 40.9211 10

-4 -4 -13 79.8747 48.5971 15

-4 4 -13 11.1896 58.4756 5

-4 -4 -13 95.4163 61.3369 5

-4 4 -13 27.5770 40.6568 8

-4 -4 -13 68.3260 39.2414 8

-4 -4 -13 48.9862 78.2240 28

-4 4 -14 1898.81 164.697 17

-4 4 -14 1335.76 192.236 2

-4 -4 -14 1465.89 196.698 2

-4 -4 -14 1803.18 107.222 10

-4 4 -14 1838.78 107.731 10

-4 4 -14 1452.57 107.739 24

-4 -4 -14 1274.75 110.323 15

-4 4 -14 1645.57 141.432 5

-4 -4 -14 1605.51 140.363 5

-4 4 -14 1688.86 107.142 8

-4 -4 -14 1765.46 108.056 8

-4 -4 -14 1391.62 137.380 28

-4 4 -15 466.925 118.938 17

-4 4 -15 320.850 141.941 2

-4 -4 -15 477.988 154.935 2

-4 -4 -15 520.463 56.9505 10

-4 4 -15 477.520 57.1730 10

-4 4 -15 641.270 61.4649 8

-4 -4 -15 472.876 57.7978 8

-4 4 -15 326.282 58.0230 24

-4 4 -15 369.486 78.3001 5

-4 -4 -15 462.410 86.9922 5

-4 -4 -15 326.016 99.6582 28

-4 -4 -15 416.037 63.4020 15

-4 4 -16 720.533 66.1346 8

-4 -4 -16 652.844 65.0093 8

-4 -4 -16 510.745 106.004 28

-4 4 -16 484.531 62.7595 24

-4 4 -16 723.650 126.613 17

-4 4 -17 52.5543 40.9031 8

-4 -4 -17 15.9905 42.0460 8

-4 -4 -17 27.0817 92.9362 28

-4 4 -17 82.1818 113.614 17

-4 -4 -18 1303.12 85.7933 8

-4 4 -18 1250.23 86.7260 8

-4 4 -18 1486.11 148.553 17

-4 4 -19 497.387 53.3398 8

-4 -4 -19 363.335 49.1773 8

-4 4 -19 384.569 106.764 17

-4 4 -20 56.9237 36.0025 8

4 -6 -21-6.35070 81.9644 28

4 -6 -20 207.517 79.1150 23

4 -6 -20 178.617 97.8943 28

4 -6 -19 559.156 100.337 23

4 -6 -19 425.838 115.232 28

4 6 -19 592.032 56.8307 8

4 -6 -18 26.1714 109.564 28

4 6 -18 7.16459 44.7943 22

4 -6 -18 29.0830 92.1916 23

4 6 -18 48.0522 39.9229 8

4 6 -17 126.348 48.7800 22

4 6 -17 52.4124 41.4891 8

4 -6 -17 112.086 101.583 23

4 6 -17 91.2029 45.2376 24

4 6 -17 84.3598 86.2986 17

4 6 -16 2464.71 136.197 8

4 -6 -16 2313.89 134.734 8

4 6 -16 2230.09 168.065 17

4 6 -16 2243.89 139.405 24

4 -6 -16 2389.05 189.214 23

4 -6 -16 2312.00 168.494 26

4 6 -15 20.9146 81.4435 17

4 6 -15-2.54214 45.7061 24

4 -6 -15 230.674 67.3497 7

4 6 -15 297.123 71.7922 7

4 -6 -15 45.5690 107.724 23

4 -6 -15-44.7804 81.5736 26

4 -6 -15-19.0941 36.8062 8

4 6 -15-4.32575 36.8814 8

4 6 -14 872.866 108.202 17

4 -6 -14 793.921 77.8382 18

4 6 -14 790.674 73.9452 24

4 6 -14 793.814 100.704 5

4 -6 -14 589.178 89.9543 5

4 -6 -14 808.658 64.9239 8

4 6 -14 810.186 65.5398 8

4 -6 -14 873.263 84.3704 7

4 6 -14 888.918 83.5007 7

4 -6 -14 916.727 103.244 26

4 6 -13 1203.84 114.701 17

4 -6 -13 1108.88 93.9875 18

4 -6 -13 1335.12 114.144 26

4 6 -13 1220.04 100.196 9

4 -6 -13 1342.21 111.483 27

4 6 -13 1192.73 90.2803 24

4 -6 -13 1148.08 96.3031 20

4 -6 -13 1079.63 110.714 5

4 6 -13 1313.43 119.875 5

4 -6 -13 1280.46 82.5193 8

4 6 -13 1261.84 97.3801 7

4 6 -13 1411.54 84.2983 8

4 -6 -13 1333.88 97.7374 7

4 -6 -13 1247.26 99.5774 9

4 -6 -12 7478.02 368.662 20

4 6 -12 7379.04 358.759 8

4 -6 -12 7067.86 357.686 8

4 -6 -12 7499.82 369.000 18

4 6 -12 7073.81 379.337 17

4 6 -12 7226.97 369.386 7

4 6 -12 7474.32 366.963 24

4 6 -12 7455.44 372.357 9

4 -6 -12 7874.81 380.894 26

4 6 -12 7135.26 392.426 5

4 -6 -12 6695.03 388.900 5

4 -6 -12 8603.34 385.023 27

4 -6 -11 1156.53 87.3810 18

4 -6 -11 1177.36 83.1583 20

4 -6 -11 1142.81 73.5202 8

4 6 -11 1064.83 99.4683 17

4 6 -11 1131.01 84.7652 7

4 -6 -11 1151.64 97.0503 21

4 6 -11 1174.55 84.5219 24

4 6 -11 1066.45 87.3767 9

4 -6 -11 1231.35 99.3343 26

4 -6 -11 1123.09 117.625 25

4 6 -11 1338.05 105.515 5

4 -6 -11 1104.44 99.0724 5

4 -6 -11 1113.46 95.5871 27

4 -6 -10 10732.3 515.274 18

4 -6 -10 10707.6 521.629 26

4 -6 -10 11006.8 541.040 25

4 6 -10 9699.87 529.806 5

4 -6 -10 9707.78 529.261 5

4 -6 -10 11066.6 520.486 21

4 6 -10 10157.3 520.701 17

4 6 -10 10480.5 512.228 24

4 -6 -9 2352.51 154.695 25

4 6 -9 2184.78 133.499 17

4 -6 -9 2201.95 125.542 18

4 -6 -9 2384.33 130.926 21

4 6 -9 2246.20 139.591 5

4 -6 -9 1938.01 133.876 5

4 6 -9 1882.54 146.218 4

4 -6 -9 2021.13 147.228 4

4 -6 -9 2093.87 132.082 11

4 6 -9 1922.72 137.845 13

4 6 -8 7187.67 353.680 17

4 6 -8 6473.78 363.310 13

4 -6 -8 7488.84 362.768 5

4 6 -8 6837.50 369.567 4

4 -6 -8 7088.12 369.493 4

4 6 -8 7504.60 364.259 5

4 -6 -8 7221.75 350.762 18

4 6 -7 28895.4 1511.44 13

4 -6 -7 34216.8 1498.17 18

4 6 -7 31323.2 1512.53 4

4 -6 -7 33343.7 1513.34 4

4 6 -6 42036.3 2155.45 13

-4 6 6 48524.2 2143.66 13

4 -6 -6 48667.5 2141.27 21

4 6 -6 43614.1 2150.23 4

4 -6 -6 46421.4 2150.70 4

-4 6 5 32163.4 1442.97 13

4 -6 -5 28674.6 1453.74 12

4 6 -5 28951.9 1453.87 13

4 -6 -5 32455.7 1437.53 18

4 -6 -5 32598.1 1438.54 21

4 -6 -5 31284.6 1443.42 4

4 6 -5 28704.9 1442.34 4

-4 6 4 1434.57 86.9959 13

4 -6 -4 1210.55 101.676 12

4 6 -4 1268.41 97.9313 13

4 6 -4 1141.14 77.5291 4

4 -6 -4 1245.03 79.3599 4

4 -6 -4 1257.41 78.1343 18

4 -6 -3 70863.3 3269.32 12

-4 6 3 76202.5 3247.66 13

4 6 -3 71225.6 3259.62 13

4 -6 -3 70811.6 3233.99 4

4 6 -3 66632.4 3233.73 4

4 -6 -2 11011.5 570.889 12

-4 6 2 12194.6 539.295 13

4 6 -2 10701.1 519.239 4

4 -6 -2 11257.0 519.834 4

4 6 -1 16084.2 772.006 4

-4 6 1 16360.4 776.100 1

-4 6 0 17295.1 845.692 2

4 -6 0 19214.7 844.218 3

4 6 0 17957.5 844.917 4

-4 6 0 17434.2 847.888 1

-4 6 -1 88318.3 3552.00 13

-4 -6 -1 74852.8 3528.79 2

4 6 1 91027.0 3535.76 3

4 -6 1 77619.6 3529.49 3

-4 6 -1 70260.1 3528.20 2

-4 6 -1 71729.3 3530.36 1

-4 -6 -2 5534.11 276.721 2

-4 6 -2 5607.03 277.090 2

-4 6 -2 5871.39 280.805 1

-4 -6 -2 5300.20 276.688 1

-4 6 -3 22624.5 1137.96 2

-4 -6 -3 23781.9 1138.64 2

-4 -6 -3 23034.8 1138.59 1

-4 6 -3 23231.0 1140.54 1

-4 6 -3 27153.8 1155.01 13

-4 6 -3 25341.6 1129.87 5

-4 -6 -3 25465.6 1130.05 5

-4 6 -4 22145.3 1107.89 1

-4 -6 -4 23495.0 1108.10 1

-4 -6 -4 22962.2 1106.67 2

-4 6 -4 22354.8 1106.17 2

-4 6 -4 24276.8 1094.86 5

-4 -6 -4 24274.5 1094.86 5

-4 6 -4 23974.8 1094.92 16

-4 -6 -5 79.1163 49.2274 2

-4 -6 -5 67.8899 54.1599 1

-4 6 -5-6.74984 49.4170 1

-4 6 -5-16.0653 50.1889 2

-4 -6 -5 88.7680 44.8568 21

-4 6 -5 55.9882 25.9342 5

-4 -6 -5 41.7376 23.6599 5

-4 6 -5 77.1986 35.1780 16

4 -6 6 791.730 73.7868 11

-4 6 -6 774.358 71.4047 16

-4 -6 -6 845.536 71.5419 21

-4 6 -6 624.661 88.6564 1

-4 6 -6 729.098 86.1527 2

-4 6 -6 764.026 61.3863 5

-4 -6 -6 735.558 60.7409 5

-4 -6 -6 886.753 90.0734 2

-4 6 -7 976.738 103.342 2

-4 -6 -7 958.012 107.058 2

-4 -6 -7 998.484 79.6123 21

4 -6 7 1040.32 83.8110 11

-4 6 -7 1004.65 76.8463 5

-4 -6 -7 951.379 74.2536 5

-4 -6 -8 4527.10 235.540 26

-4 -6 -8 4461.76 270.953 2

-4 6 -8 4609.82 273.311 2

4 -6 8 4265.53 247.204 11

-4 6 -8 4554.37 256.513 17

-4 6 -8 5078.39 245.728 5

-4 -6 -8 5043.55 244.712 5

-4 -6 -8 3842.93 233.469 21

-4 6 -9 659.133 106.448 2

-4 -6 -9 865.116 117.760 2

-4 6 -9 807.160 78.6232 5

-4 -6 -9 915.002 80.9409 5

-4 -6 -9 995.045 82.7854 26

-4 6 -9 938.292 106.400 17

-4 -6 -9 926.989 79.2569 21

-4 -6 -9 911.835 79.9134 15

-4 -6 -10 430.624 62.7823 21

-4 -6 -10 488.555 69.7290 26

-4 6 -10 306.895 101.431 2

-4 -6 -10 334.107 102.052 2

-4 6 -10 224.428 54.2376 5

-4 -6 -10 314.741 56.1990 5

-4 6 -10 453.081 102.980 17

-4 -6 -10 442.205 61.9421 15

-4 -6 -10 324.735 37.4445 8

-4 -6 -10 399.886 40.3750 10

-4 -6 -11 317.855 42.8924 10

-4 6 -11 359.049 44.9102 10

-4 6 -11 281.771 57.9135 5

-4 -6 -11 384.747 63.7332 5

-4 -6 -11 394.819 69.3143 15

-4 -6 -11 430.686 71.8402 26

-4 6 -11 145.427 98.8226 2

-4 -6 -11 249.785 100.886 2

-4 -6 -11 380.287 42.6851 8

-4 6 -11 380.956 42.6129 8

-4 -6 -11 322.419 65.6234 21

-4 6 -12 243.173 42.7327 10

-4 -6 -12 362.234 44.4762 10

-4 -6 -12 301.330 67.8875 15

-4 6 -12 153.845 110.666 2

-4 -6 -12 289.082 117.407 2

-4 6 -12 171.458 61.0264 5

-4 -6 -12 199.228 62.5667 5

-4 -6 -12 424.609 70.1350 21

-4 6 -12 219.658 42.3989 8

-4 -6 -12 197.229 42.5481 8

-4 -6 -13 2587.54 169.148 28

-4 -6 -13 2459.51 157.290 15

-4 -6 -13 2213.06 219.342 2

-4 6 -13 2405.62 223.905 2

-4 6 -13 2164.34 147.301 24

-4 6 -13 2141.01 167.042 5

-4 -6 -13 2463.05 173.616 5

-4 -6 -13 2362.64 146.408 18

-4 6 -13 2606.62 137.834 8

-4 -6 -13 2606.29 137.500 8

-4 6 -13 2471.96 137.264 10

-4 -6 -13 2415.69 136.678 10

-4 -6 -13 2184.80 152.453 21

-4 6 -14 2613.14 164.770 24

-4 -6 -14 2759.56 190.433 5

-4 6 -14 2616.74 244.687 2

-4 -6 -14 2542.18 180.530 28

-4 6 -14 3167.63 156.948 8

-4 -6 -14 3055.09 155.688 8

-4 -6 -14 2858.83 173.294 15

-4 -6 -14 2452.87 160.244 18

-4 6 -15 1524.46 93.4901 8

-4 -6 -15 1436.70 91.3864 8

-4 6 -15 1192.76 101.432 24

-4 -6 -15 1323.83 106.504 15

-4 -6 -15 1223.84 123.581 28

-4 6 -16 261.298 44.8407 8

-4 6 -16 235.669 53.1036 24

-4 -6 -16 270.683 84.8843 28

-4 6 -17 1440.88 90.2860 8

-4 6 -17 1271.48 92.8990 24

-4 -6 -17 1342.84 122.882 28

-4 -6 -18 219.951 78.2330 28

-4 6 -18 414.782 47.1900 8

-4 6 -19 633.612 102.273 17

4 -8 -20 239.636 78.9117 28

4 -8 -19 63.6524 62.2506 23

4 8 -19 50.7885 44.7105 22

4 -8 -19 222.271 92.2519 28

4 -8 -18 21.2582 74.0202 23

4 8 -18 64.4849 47.6387 22

4 -8 -18 42.0556 96.7326 28

4 -8 -17 993.993 114.494 23

4 8 -17 826.232 75.6599 22

4 -8 -16 241.022 87.7670 26

4 -8 -16 253.258 94.1240 23

4 8 -16 152.601 52.8841 24

4 8 -16 185.714 72.0497 17

4 8 -15 544.002 66.1967 24

4 -8 -15 499.630 97.2856 26

4 -8 -15 526.224 110.136 23

4 8 -15 579.635 86.0403 17

4 -8 -14 162.206 78.8796 26

4 8 -14 159.693 52.1100 24

4 -8 -14 110.796 58.0563 18

4 8 -14 216.808 74.3743 17

4 8 -13 294.922 57.6094 24

4 8 -13 307.491 76.3999 17

4 -8 -13 354.337 64.5062 18

4 -8 -13 288.553 63.2254 20

4 -8 -13 294.162 84.8663 26

4 -8 -12 82.7987 57.7213 18

4 8 -12 33.6345 61.5346 17

4 -8 -12 146.089 53.6342 20

4 8 -11 2092.57 147.999 17

4 -8 -11 2506.88 153.505 21

4 -8 -11 2194.02 169.015 25

4 -8 -11 2372.85 145.639 18

4 8 -10 8408.10 430.445 17

4 8 -10 9017.83 426.563 24

4 -8 -10 8345.89 452.434 25

4 -8 -10 8663.51 429.740 18

4 -8 -9 2542.62 150.086 18

4 8 -9 2470.32 150.736 17

4 8 -9 2608.04 144.570 24

4 8 -9 2445.13 173.065 13

4 -8 -9 2352.61 161.846 11

4 -8 -9 2585.15 179.021 25

4 -8 -8 2854.01 160.072 21

4 8 -8 2663.62 155.261 17

4 8 -8 2549.49 177.168 13

4 -8 -8 2890.88 185.942 25

4 8 -8 2605.14 148.978 24

4 8 -7 36118.0 1683.16 24

4 8 -7 33323.1 1711.23 13

4 -8 -7 38108.6 1688.84 21

4 8 -6 11878.6 551.329 24

4 -8 -6 11441.4 555.763 21

4 8 -6 10062.4 577.856 13

-4 8 6 12164.5 565.056 13

4 -8 -6 11687.2 555.726 18

4 -8 -5 44131.1 1961.32 21

4 8 -5 37709.0 1985.86 13

-4 8 5 42728.8 1972.16 13

4 -8 -5 42446.7 1959.87 18

4 8 -4 78.8485 56.4433 13

4 -8 -4 72.7891 48.1196 12

-4 8 4 59.8424 40.4359 13

4 -8 -4 77.9559 41.9444 21

4 -8 -4 34.9044 37.8788 18

4 -8 -3 23970.4 1106.33 21

-4 8 3 26082.1 1127.49 13

4 -8 -3 22494.5 1132.76 12

4 -8 -3 22893.8 1105.11 18

4 -8 -2 41950.0 1910.26 21

-4 8 2 45305.3 1933.51 13

4 -8 -2 38765.9 1949.15 12

4 -8 -2 39779.1 1910.22 18

4 -8 -1 19893.3 979.512 21

4 -8 -1 21277.3 981.349 18

-4 8 1 23275.4 1007.03 13

4 8 -1 23251.5 1025.96 13

4 -8 -1 19261.1 1034.92 12

4 -8 0 10085.3 503.832 18

4 -8 0 10385.1 503.119 21

-4 8 0 11700.0 532.878 13

4 8 0 12088.7 553.032 13

-4 -8 0 10845.2 507.349 15

-4 8 -1 11105.1 504.679 13

-4 -8 -1 9985.93 478.472 15

4 8 1 11247.7 520.699 13

4 -8 1 9356.95 472.540 18

-4 -8 -2 4411.26 233.732 15

4 8 2 4813.99 269.140 13

-4 8 -2 4735.90 255.062 13

-4 -8 -3 25847.0 1183.73 21

-4 -8 -3 24748.3 1180.08 15

-4 8 -3 24727.4 1174.12 16

-4 8 -3 26620.0 1204.29 13

-4 -8 -4 813.917 79.2640 21

-4 -8 -4 834.569 73.5046 15

-4 8 -4 848.720 66.0134 16

-4 8 -5 770.632 65.9330 16

-4 -8 -5 689.195 69.2537 15

-4 -8 -6 60.5045 46.6846 26

-4 -8 -6 31.3936 52.5116 21

-4 8 -6 65.2140 48.6053 16

-4 -8 -6 118.811 48.8807 15

-4 -8 -7 59.7346 52.8935 15

-4 8 -7-34.6952 59.9264 16

4 8 7 25.3752 54.2415 13

-4 8 -7 43.3920 75.0412 17

4 -8 7 73.5053 51.0161 11

-4 -8 -7 30.2338 52.5220 21

-4 -8 -7 63.6269 49.9830 26

4 -8 8 3113.36 212.963 11

4 8 8 3779.67 226.562 13

-4 -8 -8 3520.40 207.009 15

-4 8 -8 3593.56 228.682 17

-4 -8 -8 3852.85 210.047 21

-4 -8 -8 4186.05 205.488 26

-4 8 -9 5526.41 312.167 17

-4 -8 -9 5578.39 289.175 26

-4 -8 -9 5411.08 292.817 21

-4 8 -10 6245.01 349.259 17

-4 8 -10 6277.59 320.495 24

-4 -8 -10 6196.03 324.533 26

-4 -8 -10 6180.47 326.759 21

-4 -8 -11 2579.25 165.794 15

-4 8 -11 2571.73 154.868 24

-4 -8 -11 2478.16 161.800 26

-4 -8 -11 2377.90 161.640 21

-4 8 -11 2729.33 194.876 17

-4 8 -12 782.582 126.550 17

-4 -8 -12 690.041 90.1587 15

-4 -8 -12 576.802 92.2935 26

-4 -8 -12 581.453 87.9188 21

-4 -8 -13 5130.79 286.898 28

-4 8 -13 5922.59 319.882 17

-4 -8 -13 5107.41 287.306 21

-4 -8 -13 5109.37 280.417 18

-4 -8 -13 5538.74 289.741 15

-4 -8 -14 2065.81 145.113 28

-4 8 -14 2153.19 174.684 17

-4 8 -14 1916.99 132.764 24

-4 -8 -14 1953.10 131.725 18

-4 -8 -14 2114.57 141.346 15

-4 -8 -15 3601.29 217.883 28

-4 8 -15 3722.58 208.568 24

-4 -8 -16 111.901 70.1116 28

-4 8 -16 253.636 97.2254 17

-4 8 -16 207.909 51.5465 24

-4 -8 -17 59.4231 63.3532 28

-4 8 -17 66.5896 85.2469 17

-4 8 -17 164.700 45.3884 24

-4 8 -18 558.655 89.4727 17

4 10 -19 331.985 43.8193 22

4 -10 -18 151.408 75.9915 28

4 -10 -17 269.166 84.9368 28

4 -10 -17 199.417 63.3542 23

4 -10 -16 36.5759 68.2349 23

4 10 -16 50.6442 44.1607 22

4 -10 -16 31.6394 78.0090 26

4 -10 -15 1447.29 127.061 23

4 -10 -13 163.679 64.7245 18

4 10 -13 90.7314 54.8764 24

4 -10 -13 72.4524 60.5773 20

4 -10 -12 816.796 89.3833 18

4 10 -12 942.760 84.8765 24

4 -10 -12 962.751 109.935 26

4 -10 -12 920.374 90.5679 20

4 -10 -11 1296.88 109.714 18

4 10 -11 1320.98 101.556 24

4 -10 -11 1378.60 123.273 26

4 -10 -11 1358.45 131.569 25

4 -10 -11 1454.40 106.911 20

4 -10 -10 14759.9 731.589 18

4 -10 -10 14518.7 751.963 25

4 -10 -10 15865.2 741.299 26

4 10 -10 15424.1 727.103 24

4 -10 -9 220.939 73.5166 21

4 -10 -9 119.689 87.6289 25

4 10 -9 197.367 53.0934 24

4 10 -9 239.056 78.7943 13

4 -10 -9 164.444 72.6508 26

4 -10 -8 1953.68 128.828 18

4 -10 -8 1940.93 132.728 21

4 10 -8 1842.51 120.388 24

4 10 -8 1588.12 154.597 13

4 -10 -8 2033.01 161.152 25

4 10 -7 469.447 60.2533 24

4 -10 -7 549.403 73.4381 18

4 10 -7 644.157 97.0161 13

4 -10 -7 522.700 72.8161 21

4 10 -6 14826.8 696.714 24

4 10 -6 14301.9 740.156 13

4 -10 -6 14421.0 700.364 18

4 -10 -6 14704.8 701.177 21

4 10 -5 1897.62 110.579 24

4 -10 -5 1642.02 117.523 21

4 10 -5 1807.05 153.348 13

-4 10 5 2012.81 132.607 13

4 -10 -5 1549.22 113.636 18

4 10 -4 180.443 41.4721 24

4 -10 -4 99.5212 46.5185 18

4 10 -4 169.067 73.7966 13

-4 10 4 171.098 54.7575 13

4 -10 -4 181.195 48.6595 21

4 10 -3 4533.63 225.260 24

4 -10 -3 4126.15 225.959 21

-4 10 3 4776.42 255.270 13

4 -10 -3 4209.58 228.657 18

4 -10 -2 28686.2 1391.43 21

4 10 -2 33348.2 1449.50 13

-4 10 2 33144.5 1427.18 13

4 -10 -2 28891.3 1393.06 18

4 -10 -1 5098.96 262.300 21

-4 -10 1 5107.20 270.576 21

-4 10 1 5715.93 298.742 13

4 10 -1 5209.77 312.460 13

4 -10 -1 5305.50 265.748 18

-4 -10 0 4480.83 230.711 21

4 -10 0 4038.78 217.150 21

-4 10 0 4871.48 258.482 13

4 10 0 4730.35 274.575 13

4 -10 0 4140.22 220.626 18

4 10 1 10551.0 516.507 13

-4 10 -1 11018.1 502.536 13

-4 -10 -1 9468.36 470.790 21

-4 -10 -1 9761.97 468.623 15

4 -10 1 9172.54 459.835 18

-4 -10 -2 4052.72 227.086 21

-4 -10 -2 4042.51 223.960 15

-4 10 -2 4187.74 249.272 13

4 10 2 4881.18 270.319 13

-4 10 -3 845.869 65.7322 24

4 10 3 761.241 104.068 13

-4 -10 -3 847.780 84.3667 21

-4 -10 -3 761.174 79.4323 15

-4 10 -3 771.748 69.7969 16

-4 10 -4 1367.30 94.0135 24

4 10 4 1537.36 138.813 13

-4 -10 -4 1544.90 100.241 26

-4 -10 -4 1345.03 110.062 21

-4 10 -4 1390.46 98.5423 16

-4 -10 -4 1447.23 107.870 15

-4 10 -5 9919.13 488.752 24

4 10 5 10327.1 532.054 13

-4 -10 -5 10789.4 491.215 26

-4 -10 -5 9907.87 499.489 15

-4 10 -5 9855.65 492.805 16

-4 -10 -5 9585.68 497.801 21

4 -10 6 316.338 72.4982 11

-4 10 -6 448.346 59.9958 24

4 10 6 443.831 84.2297 13

-4 10 -6 389.495 84.6009 17

-4 -10 -6 703.533 70.3088 26

-4 10 -6 466.446 72.0027 16

-4 -10 -6 482.061 78.2337 15

4 -10 7 523.536 83.5492 11

-4 10 -7 677.857 71.5864 24

-4 -10 -7 592.401 84.2928 15

-4 10 -7 505.410 88.2387 16

-4 10 -7 666.778 98.1770 17

-4 -10 -7 808.268 79.4582 26

4 10 7 497.099 84.9544 13

-4 -10 -8 1403.04 118.711 15

-4 10 -8 1394.20 129.612 17

-4 10 -8 1305.14 99.1535 24

-4 -10 -8 1384.11 109.114 26

-4 -10 -8 1322.31 115.239 21

-4 -10 -9 6080.72 316.140 15

-4 10 -9 5486.11 327.017 17

-4 10 -9 5969.98 305.571 24

-4 -10 -9 5987.67 309.096 26

-4 -10 -9 5593.31 313.312 21

-4 -10 -10 6408.86 322.986 15

-4 10 -10 5823.64 309.772 24

-4 10 -10 5393.84 330.749 17

-4 -10 -10 6103.43 314.377 26

-4 -10 -10 5806.36 319.354 21

-4 10 -11 748.147 81.2592 24

-4 -10 -11 699.053 91.2321 26

-4 10 -11 732.314 114.251 17

-4 -10 -11 714.072 84.2540 18

-4 -10 -11 744.476 95.0727 21

-4 -10 -12 9.74026 58.6493 18

-4 10 -12 4.61749 87.9551 17

-4 -10 -12 109.585 72.0833 21

-4 10 -12 35.8517 53.0385 24

-4 -10 -13 1192.26 109.763 21

-4 10 -13 1267.43 124.183 17

-4 10 -13 1071.30 93.7800 24

-4 -10 -13 1076.69 96.5292 18

-4 10 -14 3930.70 224.059 17

-4 10 -14 3413.55 197.229 24

-4 -10 -14 3483.34 198.090 18

-4 10 -15 69.6060 76.2076 17

-4 10 -16 234.241 75.9575 17

-4 10 -17 107.575 42.0644 24

4 12 -17 353.717 46.0308 22

4 12 -16 1661.54 94.8616 22

4 -12 -16 1255.20 103.195 28

4 12 -15 581.013 59.0139 22

4 -12 -15 551.288 86.4906 26

4 -12 -14 1759.97 132.503 26

4 -12 -14 1440.32 114.902 23

4 12 -14 1719.37 103.824 22

4 -12 -13 242.554 83.5855 26

4 -12 -13 141.466 63.4017 20

4 12 -13 115.828 44.4739 22

4 -12 -12 350.104 70.5712 18

4 -12 -12 365.959 69.1863 20

4 -12 -12 394.007 88.3385 26

4 -12 -11 542.318 76.2020 20

4 -12 -11 611.938 92.2309 26

4 12 -10 902.690 80.3496 24

4 -12 -10 832.368 105.998 25

4 -12 -10 763.049 83.7350 20

4 -12 -10 819.826 102.736 26

4 12 -9 316.405 61.0846 24

4 -12 -9 171.725 88.0184 25

4 -12 -9 292.891 81.6895 26

4 -12 -9 249.932 67.7239 18

4 -12 -9 184.042 70.5710 21

4 -12 -8 871.288 98.1641 26

4 12 -8 735.406 75.7973 24

4 -12 -8 849.653 116.110 25

4 -12 -8 686.917 88.0044 18

4 -12 -8 726.549 90.8308 21

4 -12 -7 3251.95 222.289 25

4 -12 -7 3315.86 197.873 26

4 -12 -7 3226.87 192.936 21

4 12 -7 3764.62 244.142 13

4 -12 -7 3021.22 216.047 11

4 -12 -7 3123.38 191.736 18

4 -12 -6 343.394 69.1679 21

4 12 -6 177.533 83.8597 13

4 -12 -6 219.214 65.5675 18

4 12 -6 248.451 59.3255 16

4 -12 -5 6447.08 342.263 21

4 -12 -5 7008.44 346.165 18

4 12 -5 6654.48 397.296 13

4 -12 -4 3102.61 178.735 18

4 -12 -4 3043.35 177.846 21

4 -12 -3 374.513 65.1126 18

4 -12 -3 273.391 58.6536 21

-4 -12 3 258.299 58.5077 21

-4 -12 2 3410.43 194.666 21

4 -12 -2 3174.47 186.370 21

4 -12 -2 3757.40 196.792 18

4 12 -2 3763.34 247.314 13

-4 -12 1 3448.96 191.749 21

4 12 -1 3826.87 244.973 13

4 -12 -1 3584.02 188.550 18

-4 -12 0 4168.91 238.846 21

4 12 0 4711.97 291.160 13

-4 -12 0 4440.51 234.962 18

4 12 1-45.9674 78.1466 13

-4 -12 -1 92.9247 65.4896 21

-4 -12 -1 99.6438 54.5918 18

-4 12 -1 65.2116 52.6118 16

-4 -12 -2 515.240 82.3376 21

4 12 2 597.464 104.732 13

-4 12 -2 574.620 72.9097 16

-4 -12 -3 2681.49 167.497 21

4 12 3 2383.21 197.914 13

-4 12 -3 2619.27 160.565 16

4 12 4 12572.1 618.921 13

-4 12 -4 12755.7 575.688 16

-4 -12 -4 11799.2 576.900 21

-4 -12 -5 4207.64 235.594 21

-4 12 -5 3927.29 217.038 24

4 -12 5 3716.07 252.495 11

4 12 5 4299.57 264.659 13

-4 12 -5 3284.18 229.883 17

-4 12 -5 4507.93 238.048 16

-4 -12 -5 4367.56 219.469 26

-4 12 -6 617.408 94.6258 16

-4 -12 -6 753.391 78.0802 26

4 -12 6 724.743 100.264 11

-4 12 -6 715.249 72.8531 24

-4 -12 -6 741.679 94.3347 21

-4 -12 -6 545.329 83.8565 18

-4 12 -6 581.936 91.6574 17

-4 -12 -7 153.147 60.9397 26

-4 -12 -7 72.5713 68.2086 18

-4 12 -7 122.363 75.0729 17

-4 12 -7 103.036 50.3710 24

-4 -12 -7-29.2728 74.9878 21

-4 12 -8 1089.83 116.457 17

-4 -12 -8 1473.49 106.687 26

-4 12 -8 1176.07 96.4885 24

-4 -12 -8 1220.93 110.717 18

-4 -12 -9 2846.29 182.083 18

-4 -12 -9 3243.44 180.760 26

-4 12 -9 2517.98 185.495 17

-4 -12 -9 3152.58 192.139 21

-4 12 -9 3099.08 176.551 24

-4 -12 -10 78.8218 74.5392 21

-4 12 -10 70.1448 51.1199 24

-4 -12 -11 323.331 71.7744 18

-4 -12 -11 267.327 77.4397 21

-4 12 -11 280.173 61.3720 24

-4 -12 -12 1046.64 99.5954 21

-4 -12 -12 971.567 90.7262 18

-4 12 -12 992.906 86.5772 24

-4 12 -13 912.853 79.2259 24

-4 -12 -13 805.806 82.7075 18

-4 12 -14 28.7076 46.5125 24

-4 -12 -14 93.3745 50.4344 18

-4 12 -15 375.787 54.0478 24

-4 12 -16 53.9024 33.0167 22

4 14 -13 24.1716 37.3081 22

4 -14 -13 97.8161 64.4411 26

4 14 -12 1174.76 74.8168 22

4 -14 -12 1195.46 101.408 26

4 14 -11 958.443 70.7386 22

4 -14 -11 1132.64 97.9501 26

4 -14 -11 948.385 89.7156 20

4 14 -10-3.52995 34.6819 22

4 -14 -10 74.9318 69.2524 26

-4 14 10-1.89508 39.4800 16

4 -14 -10-16.9470 56.0116 20

4 14 -9 854.400 66.6429 22

-4 14 9 824.450 71.1297 16

4 -14 -9 734.582 88.9555 25

-4 14 8 857.152 76.3275 16

4 -14 -8 829.718 97.3360 25

4 -14 -8 1114.74 99.0477 26

4 -14 -7 157.595 60.9469 20

4 -14 -7 221.320 81.8523 25

-4 14 7 268.581 54.4217 16

4 -14 -7 217.778 72.2221 26

4 -14 -6 11.4022 54.9913 20

4 -14 -6 39.8458 57.6911 21

4 -14 -6 9.38366 64.1020 26

4 -14 -6 108.153 86.0765 25

-4 14 6 38.4761 49.7589 16

4 -14 -5 801.615 86.1610 26

4 -14 -5 666.647 77.1942 21

4 -14 -5 573.033 105.592 25

-4 14 5 868.419 79.7603 16

4 -14 -4 595.087 78.3714 26

-4 14 4 678.790 73.5855 16

4 14 -4 561.431 79.6714 16

4 -14 -4 462.252 68.0327 21

4 -14 -3 307.278 65.2327 26

4 -14 -3 308.490 58.8113 21

-4 14 3 366.596 64.9021 16

-4 -14 2 61.1106 52.2959 21

4 -14 -2 24.1200 55.3877 26

-4 14 2-26.7998 55.6808 16

4 -14 -2 47.0632 47.5818 21

-4 -14 1 1621.29 116.653 21

-4 -14 0 3048.88 186.590 21

4 -14 0 3312.32 179.916 20

-4 -14 -1 2127.92 144.980 21

-4 -14 -1 1997.90 140.906 18

4 -14 1 2163.41 133.293 20

-4 -14 -2 54.5056 59.5326 18

-4 -14 -2 142.465 67.4028 21

-4 14 -2 153.773 71.4290 16

-4 -14 -3 719.148 89.9808 21

-4 14 -3 800.990 95.1323 16

-4 -14 -3 686.068 84.8848 18

-4 -14 -4 2695.76 184.360 21

-4 14 -4 3328.85 195.016 16

-4 -14 -4 3086.65 184.643 18

-4 -14 -5 178.990 67.8674 18

-4 14 -5 353.277 89.4674 16

-4 -14 -5 333.952 76.6215 21

-4 -14 -6 271.901 69.0961 18

-4 -14 -6 163.319 72.7795 21

-4 -14 -7 954.363 95.5374 21

-4 -14 -7 729.454 86.3507 18

-4 14 -7 822.353 77.0141 24

-4 -14 -8 1672.05 126.081 21

-4 -14 -8 1745.16 125.232 18

-4 14 -8 1705.39 115.287 24

-4 14 -9 207.232 51.9389 24

-4 -14 -9 110.902 61.5152 18

-4 14 -10-2.47021 47.5214 24

-4 -14 -10 75.2752 58.7730 18

-4 14 -11 64.5197 51.0947 24

-4 -14 -12 132.505 54.0329 18

-4 14 -13 47.4094 32.6371 22

4 -16 -9 52.3856 46.4458 26

4 -16 -8 60.9990 54.5686 20

4 -16 -8 86.4062 49.4452 26

-4 16 7 31.9469 48.2866 16

4 -16 -7 47.5636 55.4961 20

4 -16 -7 27.2092 48.6396 26

-4 16 6 1453.92 98.4513 16

4 -16 -6 1185.91 97.2291 20

-4 16 5 103.132 55.0652 16

4 -16 -5 129.563 56.2548 20

-4 16 4 344.411 63.2995 16

4 -16 -4 329.602 62.0419 20

4 -16 -3-18.7726 66.3964 25

-4 16 3 54.1726 61.4571 16

4 -16 -3 5.09300 50.8283 20

4 -16 -2 711.093 75.0701 20

-4 16 2 875.474 88.7489 16

4 16 -2 751.322 88.5768 16

4 -16 -1 211.208 56.8865 20

4 16 -1 334.457 73.1172 16

-4 16 0 492.067 81.7747 16

4 16 0 452.105 78.9866 16

-4 -16 -1 914.003 85.5842 21

-4 16 -1 1366.20 111.786 16

-4 16 -2 442.323 89.3792 16

-4 -16 -2 175.209 59.3750 21

-4 16 -3 125.270 83.0329 16

-4 -16 -3 97.2351 57.8416 21

-4 -16 -4-2.58354 57.0517 21

-4 -16 -4 102.764 60.6183 18

-4 -16 -5 212.549 61.3572 21

-4 -16 -5 233.086 65.5285 18

-4 -16 -6 40.0362 57.6531 21

-4 -16 -6-7.96407 55.3734 18

-4 -16 -7 412.114 67.6339 21

-4 -16 -7 307.559 62.0009 18

5 1 -22 525.192 56.4522 8

5 1 -21 14.4185 45.3641 8

5 -1 -20 167.162 128.242 28

5 1 -20 45.8577 46.8061 8

5 -1 -20-12.0991 117.477 23

5 -1 -19-13.7347 133.527 28

5 1 -19 104.679 141.004 28

5 1 -19 47.0172 46.9057 8

5 -1 -19-35.0806 128.272 23

5 1 -19 144.952 147.533 23

5 1 -18 492.249 155.152 28

5 -1 -18 504.020 149.188 28

5 -1 -18 683.966 150.816 23

5 1 -18 676.330 173.626 23

5 1 -18 597.841 68.2916 8

5 -1 -18 630.706 86.0419 7

5 1 -17 186.434 80.2994 5

5 -1 -17 81.9370 70.8538 5

5 -1 -17 304.977 79.5765 9

5 -1 -17 288.006 138.833 28

5 1 -17 32.0736 138.905 28

5 1 -17 199.242 150.010 23

5 -1 -17 245.611 140.884 23

5 1 -17 293.743 54.7764 8

5 -1 -17 233.656 73.8947 7

5 1 -16 3306.71 184.821 22

5 1 -16 3531.20 234.109 5

5 -1 -16 3379.05 205.626 9

5 1 -16 2825.79 256.389 28

5 -1 -16 3130.73 251.297 28

5 -1 -16 3483.77 263.927 23

5 1 -16 2858.11 263.779 23

5 1 -16 3754.64 189.944 8

5 -1 -16 3513.87 201.955 7

5 -1 -15 405.697 72.2805 7

5 -1 -15 431.222 158.833 2

5 -1 -15 589.969 141.355 23

5 -1 -15 661.902 101.464 27

5 1 -15 330.525 137.217 28

5 -1 -15 533.946 79.4954 9

5 1 -15 464.954 90.1452 5

5 1 -15 525.445 54.5416 22

5 1 -15 497.638 59.1050 8

5 -1 -14 10875.0 547.347 7

5 -1 -14 11148.4 638.565 2

5 -1 -14 11685.5 596.603 23

5 -1 -14 11160.6 563.437 27

5 1 -14 11634.2 564.608 27

5 1 -14 10555.0 591.441 28

5 -1 -14 11442.0 551.607 9

5 -1 -14 10363.4 595.063 4

5 1 -14 11680.9 583.825 5

5 1 -14 10585.6 534.706 22

5 1 -14 12136.9 540.947 8

5 -1 -13 97.9334 85.0902 4

5 -1 -13 107.538 51.8709 7

5 -1 -13 411.697 135.709 2

5 -1 -13 308.065 111.087 23

5 -1 -13 239.974 55.3089 9

5 -1 -13 245.831 66.5628 27

5 1 -13 277.522 69.7571 27

5 1 -13 262.536 70.6788 5

5 1 -13 277.616 43.1369 8

5 -1 -12 336.852 53.1242 7

5 -1 -12 375.995 66.9516 27

5 1 -12 504.758 69.3179 27

5 1 -12 327.643 42.5548 8

5 -1 -12 334.142 57.5948 9

5 -1 -12 459.049 91.6372 4

5 1 -12 350.529 47.3456 6

5 1 -12 388.793 68.5730 5

5 -1 -12 432.742 105.512 23

5 -1 -12 392.546 121.250 2

5 1 -11 2848.27 169.985 17

5 -1 -11 3031.33 232.583 2

5 1 -11 2849.30 155.717 6

5 -1 -11 2865.08 200.324 4

5 1 -11 3269.25 186.375 5

5 1 -10 8709.00 429.854 17

5 -1 -10 9814.65 489.065 2

5 1 -10 8482.06 418.710 6

5 1 -10 9131.89 444.785 5

5 -1 -10 8630.86 456.382 4

5 1 -9 208.848 33.6813 6

5 -1 -9 203.434 33.6835 6

5 -1 -9 284.448 66.9385 4

5 -1 -9 330.020 92.6438 2

5 1 -9 204.493 51.1265 5

5 -1 -9 298.402 60.1081 17

5 1 -9 290.817 54.1319 17

5 1 -8 5608.38 286.615 5

5 -1 -8 5338.47 297.869 4

5 -1 -8 5744.45 316.085 2

5 -1 -7 907.951 79.6861 4

5 -1 -7 893.933 100.202 2

5 -1 -6 266.025 55.0005 4

5 1 -6 301.813 39.9695 5

5 1 -6 276.803 23.6832 13

5 -1 -6 282.817 72.3470 2

5 1 -5 1862.86 113.758 3

5 1 -5 1937.81 105.648 5

5 -1 -5 1808.39 115.145 4

5 1 -4 70467.8 3190.11 3

5 -1 -4 71874.9 3194.65 4

5 1 -4 70173.2 3184.74 5

5 -1 -4 60968.0 3208.44 11

5 -1 -4 68916.0 3197.12 12

5 -1 -3 3593.83 196.681 11

5 -1 -3 3440.28 187.936 12

5 1 -3 3413.90 172.208 12

5 -1 -2 334023. 14994.0 11

5 -1 -2 313800. 14997.6 12

5 1 -2 337985. 14983.1 12

5 1 -2 318658. 14983.2 3

5 1 -1 50245.7 2327.19 12

5 -1 0 42148.0 1964.88 11

5 -1 1 15650.3 759.439 11

5 -1 2 104851. 4849.51 11

5 -1 3 104602. 5023.66 11

-5 -1 -3 110994. 5003.23 1

5 -1 4 589.983 107.571 11

-5 -1 -4 773.769 88.9362 1

5 -1 5 2150.16 147.972 11

-5 -1 -5 1662.25 144.102 1

-5 -1 -5 1980.55 139.144 2

5 -1 6 145661. 6271.87 11

-5 -1 -6 133204. 6286.51 1

-5 -1 -6 126464. 6282.78 2

-5 -1 -7 3388.24 228.837 1

-5 -1 -7 3577.58 229.565 2

-5 1 -8 8092.06 411.842 17

-5 -1 -8 8334.84 409.775 17

-5 -1 -8 8502.15 452.627 1

-5 -1 -8 8133.00 447.172 2

-5 1 -9 762.263 88.8329 17

-5 -1 -9 889.379 87.8601 17

-5 1 -9 655.312 74.8182 5

-5 -1 -9 921.220 121.380 2

-5 -1 -9 817.105 131.872 1

-5 -1 -10 8133.29 475.202 1

-5 1 -10 8964.95 408.728 10

-5 -1 -10 8927.45 408.500 10

-5 1 -10 8047.73 432.271 5

-5 -1 -10 8111.11 471.725 2

-5 1 -10 9057.99 407.844 8

-5 -1 -10 7315.31 419.781 28

-5 1 -10 7825.70 428.926 28

-5 1 -10 8028.41 428.187 17

-5 -1 -10 7932.05 425.780 17

-5 1 -11 1399.77 132.029 17

-5 -1 -11 1492.64 132.754 17

-5 -1 -11 1212.50 174.740 1

-5 1 -11 1632.92 98.0931 10

-5 1 -11 1409.41 119.923 5

-5 -1 -11 1502.19 175.365 2

-5 1 -11 1378.47 117.519 23

-5 -1 -11 1285.38 112.336 23

-5 1 -11 1589.82 96.5675 8

-5 -1 -11 1478.98 122.305 28

-5 1 -11 1544.61 134.591 28

-5 -1 -12 112.764 66.7384 23

-5 1 -12 110.385 43.3547 10

-5 1 -12 125.956 42.3591 8

-5 -1 -12 190.988 126.420 2

-5 -1 -12 170.226 90.2359 17

-5 1 -12 149.460 91.4574 17

-5 1 -12 22.2167 58.7527 5

-5 -1 -12 137.288 84.1385 28

-5 1 -12 96.9485 87.6736 28

-5 -1 -13 30.4716 66.7447 23

-5 1 -13 42.3666 45.3429 10

-5 1 -13 44.5401 43.7605 8

-5 -1 -13 132.721 132.581 2

-5 -1 -13 99.8518 94.5762 17

-5 1 -13 40.0725 102.278 17

-5 1 -13 85.5804 67.0676 5

-5 -1 -13 83.4607 92.0996 28

-5 1 -13 10.8611 98.0828 28

-5 -1 -14 93.0999 74.4904 23

-5 1 -14 91.9479 48.5581 10

-5 1 -14 142.351 48.4677 8

-5 -1 -14 71.8036 124.457 2

-5 -1 -14 164.096 106.882 17

-5 1 -14 142.139 107.675 17

-5 1 -14 65.7380 63.1343 5

-5 1 -14 59.2968 111.917 28

-5 -1 -14 111.332 92.3522 28

-5 1 -15 4.24234 37.4524 24

-5 1 -15-2.71886 49.7011 10

-5 1 -15-2.60119 48.2694 8

-5 -1 -15-75.9494 131.543 2

-5 1 -15-76.1891 69.2079 5

-5 -1 -15 209.736 109.509 28

-5 1 -15 28.8090 112.018 17

-5 -1 -15 28.9895 105.825 17

-5 1 -16 410.264 120.663 17

-5 -1 -16 518.683 123.266 17

-5 1 -16 462.131 63.8562 10

-5 1 -16 497.271 63.4220 8

-5 -1 -16 477.174 124.419 28

-5 -1 -16 503.978 95.0991 5

-5 1 -16 378.617 89.5423 5

-5 1 -17-17.1282 110.550 17

-5 1 -17 5.06682 47.0812 8

-5 1 -18 704.540 70.5848 8

-5 1 -19 53.0405 43.4291 8

-5 1 -20 17.8494 37.8860 8

-5 1 -21 18.7239 33.3791 8

5 -3 -21 378.916 118.679 28

5 -3 -21 380.037 52.4107 8

5 3 -21 455.778 54.4003 8

5 -3 -20 662.388 128.215 23

5 -3 -20 804.938 137.622 28

5 3 -20 780.028 71.2746 8

5 -3 -20 836.750 70.9857 8

5 -3 -19 1626.70 175.122 23

5 -3 -19 1671.36 178.251 28

5 3 -19 2024.00 122.455 8

5 -3 -19 1960.03 121.172 8

5 -3 -18 2045.52 189.228 23

5 -3 -18 1809.82 122.331 8

5 -3 -18 2248.25 147.397 7

5 3 -18 2107.63 146.835 7

5 3 -18 1897.86 123.047 8

5 -3 -18 2244.20 199.544 28

5 3 -17 69.9014 149.018 28

5 -3 -17 295.385 129.468 23

5 -3 -17 352.705 72.9121 7

5 3 -17 342.634 76.4071 7

5 3 -17 431.910 89.5809 5

5 -3 -17 328.026 84.5405 5

5 3 -17 349.478 56.1887 22

5 3 -17 409.620 58.7737 8

5 -3 -17 375.577 54.6953 8

5 3 -16 904.984 118.948 17

5 -3 -16 858.462 93.2496 7

5 3 -16 939.246 98.4496 7

5 -3 -16 997.542 98.3752 9

5 3 -16 1012.24 100.863 9

5 3 -16 710.452 172.026 28

5 -3 -16 857.562 150.948 23

5 3 -16 808.912 72.0749 24

5 3 -16 1066.24 121.284 5

5 -3 -16 981.827 117.397 5

5 -3 -16 1040.90 78.3826 8

5 3 -16 1102.46 80.2384 8

5 3 -16 997.985 77.6707 22

5 3 -15 685.175 103.527 17

5 3 -15 740.974 84.0608 7

5 -3 -15 561.203 79.5464 7

5 3 -15 409.119 150.201 28

5 -3 -15 708.011 102.015 27

5 -3 -15 517.746 94.1840 26

5 -3 -15 608.444 85.0257 9

5 3 -15 770.495 89.7122 9

5 -3 -15 864.554 69.0109 8

5 3 -15 715.939 67.0387 8

5 -3 -15 683.951 137.195 23

5 3 -15 595.213 164.654 23

5 3 -15 594.461 60.3612 24

5 3 -15 699.455 98.5911 5

5 -3 -15 834.397 102.138 5

5 3 -14 11566.0 593.137 17

5 -3 -14 12215.6 580.739 7

5 3 -14 11521.7 579.930 7

5 -3 -14 13553.9 598.529 27

5 -3 -14 11387.0 585.830 26

5 3 -14 11976.7 583.889 9

5 -3 -14 12281.2 584.050 9

5 -3 -14 12042.9 569.340 8

5 3 -14 12678.2 570.671 8

5 3 -14 12168.5 668.914 2

5 -3 -14 11839.9 668.454 2

5 -3 -14 13124.8 626.401 23

5 3 -14 11521.2 566.504 24

5 3 -14 10943.4 644.912 23

5 -3 -14 11610.5 610.041 5

5 3 -14 12339.3 613.877 5

5 3 -13 300.517 70.2420 5

5 -3 -13 147.715 63.2998 5

5 3 -13 226.737 119.915 2

5 -3 -13 223.925 126.331 2

5 3 -13 243.628 41.5437 8

5 -3 -13 266.013 42.2913 8

5 3 -13 251.855 78.0809 17

5 3 -13 180.204 55.8098 7

5 -3 -13 274.343 56.8551 7

5 3 -13-6.89106 114.614 28

5 -3 -13 286.415 70.1989 27

5 3 -13 152.857 73.2478 27

5 -3 -13 465.579 73.2462 26

5 3 -13 251.716 59.8213 9

5 -3 -13 260.730 57.6948 9

5 3 -13 134.566 127.063 23

5 3 -13 220.345 41.4010 24

5 -3 -13 233.486 86.7032 4

5 3 -13 280.102 93.4924 4

5 3 -12 646.790 56.0020 24

5 3 -12 461.181 132.172 23

5 -3 -12 693.185 71.7140 9

5 3 -12 658.012 69.0878 9

5 3 -12 777.040 58.6168 8

5 -3 -12 678.432 55.6207 8

5 -3 -12 671.566 82.0452 27

5 3 -12 339.391 111.550 28

5 3 -12 638.745 83.7592 27

5 3 -12 591.397 68.0004 7

5 -3 -12 595.237 66.5216 7

5 3 -12 807.129 88.2451 5

5 -3 -12 715.955 83.8046 5

5 3 -12 685.652 138.064 2

5 -3 -12 502.011 133.215 2

5 -3 -12 678.382 74.9101 26

5 3 -12 579.216 101.602 4

5 -3 -12 622.046 102.763 4

5 3 -12 774.456 62.9686 6

5 -3 -12 809.116 62.4695 6

5 -3 -12 622.832 59.0017 18

5 3 -12 736.129 86.5672 17

5 3 -11 16428.3 824.594 7

5 3 -11 18591.7 835.822 17

5 -3 -11 18233.9 821.586 18

5 3 -11 16717.2 821.286 6

5 -3 -11 16654.5 821.116 6

5 3 -11 17011.5 875.135 23

5 3 -11 17279.8 850.264 5

5 -3 -11 17627.8 848.569 5

5 3 -11 16121.5 862.492 4

5 -3 -11 16684.9 862.894 4

5 3 -11 17348.2 891.575 2

5 -3 -11 17350.1 887.105 2

5 -3 -10 13039.4 626.075 6

5 3 -10 13666.5 626.955 6

5 3 -10 13326.0 666.504 4

5 -3 -10 13095.5 665.014 4

5 3 -10 14591.8 653.986 5

5 -3 -10 13584.7 650.892 5

5 3 -10 13088.0 636.521 17

5 -3 -10 12685.6 625.226 18

5 -3 -10 12985.9 628.731 20

5 -3 -10 13771.9 686.790 2

5 3 -10 14099.1 690.492 2

5 -3 -9 8417.47 436.973 2

5 3 -9 8521.86 437.586 2

5 -3 -9 8329.25 381.217 18

5 3 -9 8348.14 390.327 17

5 -3 -9 7927.83 399.529 5

5 3 -9 7609.74 413.517 4

5 3 -9 8246.48 402.072 5

5 -3 -9 8095.10 415.029 4

5 -3 -9 7351.94 378.874 6

5 3 -9 7690.87 379.588 6

5 3 -8 4938.65 283.205 2

5 -3 -8 5098.68 282.999 2

5 3 -8 4430.60 237.881 13

5 -3 -8 4827.72 251.485 5

5 3 -8 4747.33 264.190 4

5 -3 -8 5060.91 265.794 4

5 3 -8 4953.83 252.163 5

5 -3 -8 4710.76 265.285 11

5 3 -7 34038.7 1538.17 2

5 -3 -7 33740.6 1536.90 2

5 3 -7 35636.6 1515.44 5

5 -3 -7 34576.3 1513.29 5

5 3 -7 32552.8 1523.41 4

5 -3 -7 32532.2 1523.10 4

5 3 -7 28960.8 1502.78 13

5 -3 -7 31258.6 1513.01 11

5 -3 -6 3864.44 195.513 5

5 3 -6 3556.61 204.611 4

5 -3 -6 3718.14 205.208 4

5 3 -6 4167.57 197.357 5

5 -3 -6 3823.42 193.795 11

5 3 -6 3265.50 188.180 13

5 3 -5 3542.32 198.357 13

5 -3 -5 3776.15 198.610 11

5 -3 -5 3726.32 217.974 12

5 3 -5 4384.37 202.303 5

5 -3 -5 4164.89 200.810 5

5 3 -5 3690.27 207.186 4

5 -3 -5 3810.76 208.184 4

5 -3 -4 7808.88 394.037 11

5 -3 -4 7893.40 415.168 12

5 -3 -4 8280.98 402.444 4

5 3 -4 7849.18 401.328 4

5 -3 -4 8365.00 398.964 3

5 3 -4 8894.52 401.143 3

5 -3 -3 85595.9 4021.32 3

5 3 -3 89351.3 4022.92 3

5 -3 -3 86491.2 4046.13 12

5 -3 -3 87776.7 4020.45 11

5 3 -2 86796.6 3881.04 3

5 -3 -2 84199.7 3880.12 3

5 -3 -2 79116.0 3906.51 12

5 3 -1 13795.1 637.546 3

5 -3 -1 12899.7 635.052 3

5 -3 1 285867. 12759.1 11

5 3 1 275206. 12746.5 12

-5 3 -2 139745. 6541.27 1

-5 -3 -2 147933. 6542.62 1

5 -3 2 140154. 6530.75 11

-5 3 -3 82552.6 3905.28 1

-5 -3 -3 86631.2 3906.25 1

5 -3 3 84462.0 3892.50 11

-5 3 -4 1980.40 139.977 1

-5 -3 -4 2125.77 142.445 1

-5 -3 -4 1895.31 134.350 2

-5 3 -4 1803.92 132.246 2

5 -3 4 2219.55 123.449 11

-5 -3 -5 14527.4 671.977 1

-5 3 -5 12638.8 666.413 1

-5 -3 -5 13826.0 666.823 2

-5 3 -5 13071.8 664.622 2

5 -3 5 13817.8 657.264 11

-5 3 -6 42956.0 2062.54 1

-5 -3 -6 46976.7 2066.67 1

-5 -3 -6 44541.9 2059.72 2

-5 3 -6 42162.4 2056.98 2

5 -3 6 43077.3 2056.89 11

-5 3 -7 19463.2 977.435 1

-5 -3 -7 20738.4 980.509 1

-5 3 -7 21036.3 952.535 17

-5 3 -7 19944.0 974.398 2

-5 -3 -7 19836.4 976.275 2

5 -3 7 18233.1 984.056 11

-5 3 -8 849.650 75.7856 5

-5 -3 -8 820.586 76.1642 5

-5 -3 -8 744.942 110.863 2

-5 3 -8 800.338 123.170 1

-5 3 -8 719.383 106.451 2

-5 -3 -8 785.804 120.069 1

-5 3 -8 882.065 94.1644 17

-5 3 -9 682.086 71.5909 5

-5 -3 -9 839.787 76.7339 5

-5 3 -9 548.798 90.1647 17

-5 -3 -9 539.536 85.2144 17

-5 3 -9 658.397 119.130 2

-5 3 -9 544.324 116.295 1

-5 -3 -9 794.036 125.939 1

-5 -3 -9 543.464 109.921 2

-5 3 -9 657.648 73.0739 14

-5 3 -10 1771.04 179.357 2

-5 -3 -10 2193.24 188.709 2

-5 -3 -10 2157.44 124.088 10

-5 3 -10 2336.77 124.736 10

-5 3 -10 2041.18 146.518 5

-5 -3 -10 2300.84 151.059 5

-5 -3 -10 2167.99 156.132 17

-5 3 -10 1972.83 156.859 17

-5 3 -10 2194.72 122.431 8

-5 -3 -10 2342.76 123.788 8

-5 -3 -10 1981.31 141.036 28

-5 3 -10 1830.30 190.800 1

-5 -3 -10 2324.36 199.188 1

-5 -3 -11 1504.07 174.152 2

-5 3 -11 1533.63 93.3800 10

-5 -3 -11 1475.46 92.8707 10

-5 3 -11 1478.36 119.384 5

-5 -3 -11 1374.46 117.778 5

-5 -3 -11 1236.12 129.119 17

-5 3 -11 1184.26 131.916 17

-5 3 -11 1412.48 89.6218 8

-5 -3 -11 1420.27 91.2769 8

-5 3 -11 1282.52 163.040 2

-5 -3 -11 1039.13 109.229 28

-5 3 -12 56.2648 41.3843 10

-5 -3 -12 65.4948 40.1274 10

-5 3 -12 5.46400 55.5603 5

-5 -3 -12 132.341 66.6688 5

-5 3 -12 116.831 102.080 17

-5 -3 -12 132.688 98.0348 17

-5 3 -12 90.9574 39.9590 8

-5 -3 -12 44.8221 41.2833 8

-5 3 -12 108.788 113.003 2

-5 -3 -12 9.94493 116.694 2

-5 -3 -13 2207.70 226.568 2

-5 3 -13 2238.07 222.067 2

-5 3 -13 2422.85 136.971 10

-5 -3 -13 2435.24 136.283 10

-5 -3 -13 1764.79 134.958 15

-5 3 -13 2427.71 135.455 8

-5 -3 -13 2484.55 137.014 8

-5 -3 -13 2587.21 189.638 17

-5 3 -13 2262.97 187.384 17

-5 3 -13 2221.74 167.979 5

-5 -3 -13 2168.48 169.848 5

-5 -3 -13 2042.88 157.435 28

-5 3 -14 39.2355 111.666 17

-5 3 -14-104.207 122.649 2

-5 -3 -14 186.374 128.867 2

-5 3 -14 113.862 45.1008 10

-5 -3 -14 88.7846 43.7406 10

-5 -3 -14 137.972 52.0424 15

-5 -3 -14 94.7872 47.0020 8

-5 3 -14 115.804 43.5706 8

-5 3 -14 19.1293 62.8793 5

-5 -3 -14 96.3913 69.6903 5

-5 -3 -14 90.8549 88.6605 28

-5 3 -15 586.610 127.847 17

-5 3 -15 320.615 143.791 2

-5 -3 -15 272.914 146.260 2

-5 3 -15 421.464 58.7695 10

-5 -3 -15 390.897 56.8888 10

-5 -3 -15 226.105 77.2398 5

-5 3 -15 251.804 77.0461 5

-5 -3 -15 272.629 56.9902 15

-5 3 -15 392.866 57.2853 8

-5 -3 -15 297.743 56.0479 8

-5 -3 -15 314.312 101.606 28

-5 3 -15 278.135 53.7650 24

-5 3 -16 168.262 46.5594 24

-5 -3 -16 182.416 100.547 28

-5 3 -16 347.968 125.477 17

-5 3 -16 205.572 52.0443 10

-5 -3 -16 192.794 50.3535 10

-5 3 -16 222.560 52.9863 8

-5 -3 -16 208.709 50.3149 8

-5 3 -17 536.273 62.8930 8

-5 -3 -17 494.713 60.0145 8

-5 3 -17 621.958 125.714 17

-5 -3 -17 373.394 102.579 28

-5 3 -18 68.4413 42.5265 8

-5 -3 -18 75.0564 44.2386 8

-5 3 -18 75.9623 106.660 17

-5 3 -19 58.0200 39.8617 8

-5 -3 -19 26.9603 39.0681 8

-5 3 -20 193.935 41.0282 8

-5 -3 -20 174.770 39.8043 8

5 -5 -20 92.8822 91.1048 23

5 5 -20 11.1581 39.1194 8

5 -5 -19 3294.06 222.833 23

5 5 -19 3217.88 174.860 8

5 -5 -18 1975.06 173.208 23

5 5 -18 1843.42 115.939 8

5 -5 -18 1910.40 116.081 8

5 5 -18 1882.27 120.938 22

5 5 -17 2066.02 126.209 24

5 -5 -17 2125.06 189.204 23

5 5 -17 2078.92 125.131 8

5 -5 -17 1926.33 123.770 8

5 5 -17 2156.95 129.195 22

5 -5 -16 2030.03 166.272 26

5 5 -16 2197.42 170.944 17

5 5 -16 2159.25 139.091 24

5 -5 -16 2162.43 196.863 23

5 -5 -16 2599.63 156.838 7

5 5 -16 2666.85 141.751 8

5 5 -16 2275.56 154.862 7

5 -5 -16 2580.15 140.833 8

5 5 -15 9388.28 474.870 7

5 -5 -15 9519.47 477.559 9

5 -5 -15 9411.30 484.724 26

5 5 -15 9161.41 477.233 9

5 5 -15 9616.88 510.946 5

5 -5 -15 9481.21 506.495 5

5 -5 -15 9832.40 474.396 7

5 5 -15 9775.72 462.116 8

5 -5 -15 9123.72 459.461 8

5 5 -15 9711.83 486.799 17

5 -5 -15 10156.3 517.430 23

5 5 -15 9315.14 463.776 24

5 5 -14 593.656 95.6108 17

5 5 -14 573.466 62.5855 24

5 -5 -14 687.038 82.0358 9

5 5 -14 734.707 80.5198 9

5 -5 -14 701.460 92.1498 26

5 -5 -14 668.004 76.2919 7

5 5 -14 672.301 77.0446 7

5 -5 -14 605.737 59.5817 8

5 5 -14 627.488 60.3196 8

5 -5 -14 837.494 92.6826 27

5 5 -14 557.667 89.2937 5

5 -5 -14 558.078 89.0805 5

5 5 -13 1878.30 137.647 17

5 -5 -13 2054.60 120.987 18

5 -5 -13 1808.64 122.482 7

5 5 -13 1773.37 123.784 7

5 5 -13 1728.11 114.258 24

5 5 -13 1772.39 126.636 9

5 -5 -13 2100.36 128.751 9

5 -5 -13 2086.26 136.186 26

5 5 -13 1802.81 108.856 8

5 -5 -13 1700.59 107.957 8

5 -5 -13 1615.65 143.073 5

5 5 -13 1555.59 139.293 5

5 -5 -13 2391.14 144.100 27

5 5 -12 13301.7 672.329 17

5 -5 -12 14072.4 661.009 18

5 5 -12 13800.6 663.777 7

5 -5 -12 14012.1 663.206 7

5 5 -12 13590.9 658.516 24

5 -5 -12 14424.9 667.008 9

5 5 -12 13161.7 664.596 9

5 -5 -12 13463.3 669.522 26

5 5 -12 14616.1 655.111 8

5 -5 -12 13638.8 653.698 8

5 -5 -12 14449.8 692.191 5

5 5 -12 15251.1 696.147 5

5 5 -12 12604.0 702.848 4

5 -5 -12 13259.1 704.016 4

5 -5 -12 13389.5 662.884 20

5 -5 -12 14053.6 672.774 27

5 5 -11 23607.5 1098.93 17

5 -5 -11 23603.3 1088.26 18

5 -5 -11 23699.7 1089.37 7

5 5 -11 21781.7 1087.98 7

5 -5 -11 27241.2 1103.38 27

5 -5 -11 24336.0 1097.57 26

5 -5 -11 23436.1 1116.90 25

5 -5 -11 23857.0 1091.10 9

5 5 -11 23302.0 1091.62 9

5 5 -11 23899.2 1086.94 24

5 5 -11 23479.8 1081.51 8

5 -5 -11 22568.0 1080.43 8

5 -5 -11 23516.3 1088.24 20

5 -5 -11 22738.8 1111.94 5

5 5 -11 23945.2 1114.76 5

5 5 -11 20739.3 1124.79 4

5 -5 -11 21586.6 1125.41 4

5 -5 -10 699.060 94.8160 4

5 5 -10 509.710 89.9085 4

5 -5 -10 1523.38 115.292 25

5 -5 -10 1285.55 82.0233 26

5 5 -10 706.445 130.691 23

5 5 -10 1030.32 65.1465 24

5 -5 -10 1146.24 70.0770 20

5 -5 -10 1035.22 68.9365 18

5 5 -10 1047.96 85.4836 17

5 5 -10 409.331 70.1735 5

5 -5 -10 438.316 69.6206 5

5 5 -10 456.337 54.1755 6

5 -5 -10 584.361 55.3243 6

5 5 -9 6763.61 322.163 6

5 -5 -9 6328.42 321.207 6

5 -5 -9 7075.80 332.316 21

5 -5 -9 7213.51 328.852 18

5 5 -9 6945.89 333.843 17

5 5 -9 5955.77 337.233 13

5 -5 -9 6491.05 341.245 11

5 -5 -9 6516.42 343.064 5

5 5 -9 6603.07 345.657 5

5 5 -9 6639.64 357.951 4

5 -5 -9 6427.46 356.316 4

5 5 -8 30136.3 1386.16 6

5 -5 -8 28770.7 1385.29 6

5 -5 -8 31995.8 1393.21 18

5 5 -8 29823.1 1418.14 4

5 -5 -8 30388.4 1406.54 5

5 5 -8 30610.1 1408.10 5

5 -5 -8 30894.7 1418.06 4

5 5 -8 30639.7 1394.81 17

5 5 -8 26860.9 1401.72 13

5 -5 -8 29207.3 1402.03 11

5 -5 -8 30668.9 1393.68 21

5 5 -7 3671.01 222.350 13

5 -5 -7 4506.83 224.232 11

5 5 -7 3998.79 222.936 5

5 -5 -7 3750.30 221.510 5

5 5 -7 4022.17 230.910 4

5 -5 -7 4096.65 231.605 4

5 -5 -6 114.564 36.6259 18

5 5 -6 136.491 45.0924 13

5 -5 -6 169.628 59.2317 12

5 5 -6 82.2557 44.9289 4

5 -5 -6 137.044 48.7183 4

5 -5 -6 123.188 38.4499 21

5 -5 -5 23320.3 1106.50 12

-5 5 5 24023.4 1080.28 13

5 5 -5 22768.0 1095.36 13

5 5 -5 22417.2 1089.10 4

5 -5 -5 22955.1 1089.18 4

5 -5 -4 9580.29 511.540 12

-5 5 4 10778.3 485.229 13

5 5 -4 10044.4 498.972 13

5 5 -4 9796.62 487.838 4

5 -5 -4 10036.6 488.026 4

5 5 -3 2537.93 145.378 4

5 -5 -3 2708.97 147.345 4

5 -5 -3 2392.54 184.897 12

-5 5 3 2789.94 146.883 13

5 5 -3 2844.95 146.429 3

-5 5 2 14407.6 619.066 13

5 -5 -2 12416.5 660.164 12

5 5 -2 12816.3 614.440 4

5 -5 -2 12381.1 610.091 3

5 5 -2 12868.0 612.061 3

-5 5 1 7876.14 364.948 13

5 -5 -1 6925.02 356.330 3

5 5 -1 7565.95 360.359 3

-5 5 0 1092.20 77.8338 13

5 5 0 1055.61 79.4749 3

5 -5 0 1080.20 74.8971 3

-5 5 -1 48191.3 2272.28 1

-5 -5 -1 49900.8 2271.76 1

-5 5 -2 6309.31 329.036 1

-5 -5 -2 6756.98 330.527 1

-5 5 -3 70306.6 3367.40 2

-5 -5 -3 74575.0 3368.63 2

-5 5 -3 71964.9 3371.48 1

-5 -5 -3 74939.7 3370.50 1

-5 -5 -4 20363.0 971.629 2

-5 5 -4 19784.2 970.877 2

5 -5 4 21853.0 966.579 11

-5 -5 -4 19994.1 974.100 1

-5 5 -4 19810.9 974.800 1

-5 -5 -5 14020.3 658.564 2

-5 5 -5 13131.8 656.410 2

5 -5 5 12900.4 644.391 11

-5 -5 -5 14040.0 661.610 1

-5 5 -5 13236.2 661.783 1

-5 5 -6 636.776 59.3229 5

-5 -5 -6 605.662 57.8628 5

-5 -5 -6 504.853 58.6568 21

-5 -5 -6 573.411 85.8390 2

-5 5 -6 604.424 82.5454 2

-5 5 -6 600.290 87.6292 1

-5 -5 -6 626.286 89.2344 1

5 -5 6 655.146 67.5132 11

-5 5 -7 7174.18 353.162 5

-5 -5 -7 7089.44 352.324 5

-5 -5 -7 7074.66 380.208 2

-5 5 -7 6541.19 377.485 2

-5 -5 -7 6834.37 344.744 21

-5 5 -7 6702.08 380.791 1

-5 -5 -7 7132.29 382.072 1

5 -5 7 7318.42 365.949 11

-5 -5 -8 7795.43 359.293 26

-5 -5 -8 7277.67 356.210 21

-5 -5 -8 7167.49 398.108 2

-5 5 -8 6653.39 395.329 2

5 -5 8 7808.47 395.626 11

-5 5 -8 6775.30 397.043 1

-5 -5 -8 7528.42 402.872 1

-5 5 -8 6801.11 364.954 5

-5 -5 -8 6766.15 364.980 5

-5 -5 -9 5136.24 311.086 2

-5 5 -9 4815.45 307.717 2

-5 -5 -9 4766.44 257.393 26

-5 -5 -9 4138.45 250.604 21

-5 5 -9 5630.34 275.440 14

-5 5 -9 5549.56 273.631 5

-5 -5 -9 5457.17 273.835 5

-5 5 -10 68.8156 47.2362 5

-5 -5 -10 77.9920 45.9497 5

-5 -5 -10 27.7774 54.5066 26

-5 -5 -10 43.3294 45.6667 21

-5 -5 -10 26.8662 31.8443 10

-5 5 -10 80.8576 33.4054 10

-5 5 -10 80.9679 32.8636 8

-5 -5 -10 81.5389 30.4569 8

-5 5 -10-7.75181 83.2181 2

-5 -5 -10 7.77258 91.5313 2

-5 5 -10-4.50326 92.6363 17

-5 5 -11 811.215 126.392 17

-5 5 -11 807.615 90.7534 5

-5 -5 -11 896.833 93.0307 5

-5 -5 -11 744.323 78.8955 21

-5 5 -11 948.819 70.0221 10

-5 -5 -11 912.242 68.9050 10

-5 5 -11 1087.70 69.5917 8

-5 -5 -11 924.879 68.0262 8

-5 -5 -11 633.728 132.807 2

-5 5 -11 901.542 142.913 2

-5 -5 -11 743.757 79.3641 15

-5 -5 -12 6963.99 371.665 15

-5 5 -12 6506.41 434.211 2

-5 -5 -12 7484.50 449.724 2

-5 5 -12 7769.87 361.926 10

-5 -5 -12 7768.28 361.844 10

-5 5 -12 7732.86 361.267 8

-5 -5 -12 7705.67 360.872 8

-5 -5 -12 7314.16 382.509 28

-5 5 -12 7776.83 408.545 17

-5 -5 -12 6178.69 364.724 21

-5 5 -12 7057.15 393.689 5

-5 -5 -12 6686.60 391.170 5

-5 5 -13 258.583 70.7107 5

-5 -5 -13 251.334 68.1455 5

-5 5 -13 344.428 50.1791 10

-5 -5 -13 302.406 50.3576 10

-5 5 -13 345.516 47.5748 8

-5 -5 -13 269.254 46.2276 8

-5 -5 -13 255.560 66.8184 15

-5 5 -13 361.516 117.757 17

-5 5 -13 134.873 121.097 2

-5 -5 -13 363.911 127.983 2

-5 -5 -13 360.752 83.4393 28

-5 -5 -14 212.106 79.5507 28

-5 5 -14 253.752 70.0017 5

-5 -5 -14 285.225 74.1977 5

-5 5 -14 252.765 59.6984 24

-5 5 -14 389.677 122.062 17

-5 -5 -14 342.432 67.2028 15

-5 5 -14 168.565 119.711 2

-5 -5 -14 349.056 123.656 2

-5 5 -14 272.657 48.3834 8

-5 -5 -14 308.688 50.2315 8

-5 5 -14 337.445 51.6942 10

-5 -5 -14 300.588 49.4937 10

-5 -5 -15 933.402 88.6484 15

-5 5 -15 1131.41 78.9572 8

-5 -5 -15 996.607 75.1079 8

-5 -5 -15 887.459 113.805 28

-5 5 -15 792.197 82.6544 24

-5 5 -15 1125.59 152.145 17

-5 5 -16 781.092 67.6700 8

-5 -5 -16 763.545 67.0182 8

-5 -5 -16 543.591 99.5692 28

-5 5 -16 691.281 71.1136 24

-5 5 -16 784.466 131.833 17

-5 5 -17 54.1623 39.1655 8

-5 -5 -17 117.471 41.0298 8

-5 -5 -17 78.3118 83.2894 28

-5 5 -17 226.947 111.737 17

-5 5 -18 85.3034 38.9826 8

-5 5 -18 181.661 98.7378 17

-5 5 -19 114.391 35.4306 8

-5 5 -19 152.838 92.4798 17

5 -7 -21 153.731 72.1993 28

5 -7 -20 277.870 95.7093 28

5 -7 -20 202.052 71.7843 23

5 7 -19 86.1356 46.5296 22

5 -7 -19-38.8448 93.0607 28

5 -7 -19 26.4465 81.6474 23

5 7 -18 207.329 52.8934 22

5 -7 -18 209.767 95.0743 23

5 -7 -18 103.422 109.601 28

5 -7 -17 1243.68 142.008 23

5 -7 -17 1295.86 126.533 26

5 7 -17 1391.57 97.4625 22

5 -7 -16 1121.81 119.803 26

5 7 -16 1079.19 110.909 17

5 7 -16 1135.35 89.2840 24

5 -7 -16 1348.91 148.072 23

5 -7 -15 2170.75 163.761 26

5 7 -15 2319.70 140.339 24

5 7 -15 2326.10 160.211 17

5 7 -14 1819.86 146.873 17

5 -7 -14 2191.74 138.881 18

5 7 -14 2169.00 141.216 7

5 -7 -14 2194.11 125.138 8

5 -7 -14 2035.37 147.254 20

5 -7 -14 2251.10 158.389 26

5 7 -14 2271.74 135.814 24

5 7 -13 12341.2 631.487 17

5 -7 -13 12525.5 623.843 18

5 -7 -13 14301.8 637.744 27

5 7 -13 12856.8 623.952 7

5 7 -13 12875.2 621.510 24

5 -7 -13 12866.6 627.497 20

5 -7 -13 12624.0 635.868 26

5 -7 -12 155.502 57.1172 20

5 7 -12 179.108 69.6250 17

5 -7 -12 194.289 58.7055 18

5 -7 -12 160.302 64.4248 27

5 -7 -12 288.560 74.6667 26

5 -7 -12 199.685 94.8630 25

5 7 -12 120.671 46.3467 24

5 -7 -11 20277.8 995.227 25

5 -7 -11 20731.1 978.030 26

5 7 -11 19404.8 964.139 24

5 -7 -11 20493.7 967.265 20

5 7 -11 20548.7 974.151 17

5 -7 -11 21662.9 970.761 18

5 -7 -10 1836.96 121.837 21

5 7 -10 1633.86 113.644 17

5 -7 -10 1592.25 112.051 18

5 -7 -10 1892.07 151.047 25

5 -7 -10 1879.50 120.787 26

5 -7 -9 15023.9 722.123 25

5 7 -9 15315.9 688.464 24

5 -7 -9 15375.2 692.498 18

5 7 -9 14055.1 691.116 17

5 -7 -9 13742.2 704.668 11

5 7 -9 12653.9 708.724 13

5 7 -8 132.345 55.9116 13

5 7 -8 140.739 48.2971 4

5 -7 -8 149.762 50.7101 11

5 -7 -8 121.916 46.9176 18

5 7 -8 134.692 46.5533 17

5 -7 -8 141.329 81.8087 25

5 7 -8 195.178 40.6614 24

5 -7 -8 109.546 52.5471 21

5 7 -7 20162.8 1060.89 13

5 7 -7 21383.9 1052.76 4

5 -7 -7 22819.5 1042.62 21

5 7 -7 23110.5 1037.37 24

5 -7 -6 285.030 48.3307 18

5 -7 -6 411.797 53.5910 21

5 7 -6 269.403 61.9226 13

-5 7 6 248.776 42.4130 13

5 7 -6 194.069 42.1041 4

5 -7 -5 22071.1 911.499 21

5 7 -5 16432.7 908.393 4

5 -7 -5 21111.6 910.446 18

5 -7 -5 17692.6 932.389 12

-5 7 5 20217.3 918.339 13

5 7 -5 17886.0 933.342 13

5 -7 -4 5704.65 268.804 21

5 -7 -4 5411.62 267.897 18

5 7 -4 4676.82 291.253 13

5 -7 -4 4894.00 298.095 12

-5 7 4 5749.75 281.054 13

5 -7 -3 31838.4 1409.65 21

5 -7 -3 29970.2 1409.01 18

5 -7 -3 27426.5 1448.66 12

-5 7 3 31175.5 1425.70 13

5 7 -3 28973.1 1441.00 13

5 -7 -2 15014.3 721.467 21

5 -7 -2 14660.9 778.915 12

-5 7 2 17262.6 743.275 13

5 -7 -2 15209.5 722.406 18

5 -7 -1 3188.24 170.443 18

5 -7 -1 3028.06 239.269 12

-5 7 0 1696.56 117.418 13

-5 7 -1 169.533 51.5813 13

-5 -7 -1 122.755 37.4281 15

-5 7 -2 3126.19 161.306 16

-5 7 -3 143.896 37.4438 1

-5 7 -3 144.806 33.6462 2

-5 7 -3 219.619 37.2879 16

-5 -7 -3 182.032 21.8661 5

-5 7 -4 2.74463 34.1692 2

-5 7 -4-5.78914 39.4278 1

-5 -7 -4 32.6950 18.3989 5

-5 7 -4 31.7025 36.3159 16

-5 -7 -5 2065.54 118.897 5

-5 7 -5 2019.24 125.970 16

-5 7 -5 2014.79 136.234 2

-5 7 -6 897.952 90.9940 2

-5 7 -6 825.682 80.3777 16

-5 -7 -6 875.231 65.9715 5

5 -7 6 891.247 82.6500 11

-5 -7 -6 797.194 77.0788 21

-5 -7 -7 4924.11 235.624 26

-5 7 -7 4080.38 256.678 2

-5 -7 -7 4291.02 237.869 15

-5 -7 -7 4516.74 234.516 5

5 -7 7 3962.96 244.242 11

-5 -7 -7 4589.92 238.766 21

-5 -7 -8 2329.19 126.102 26

-5 7 -8 1325.36 141.708 2

5 -7 8 1868.54 139.683 11

-5 -7 -8 1799.23 122.083 21

-5 7 -8 2005.16 149.862 17

-5 -7 -8 1629.00 117.121 5

-5 -7 -9 8004.96 375.225 26

-5 -7 -9 6839.34 376.490 5

-5 7 -9 6511.09 407.121 2

-5 7 -9 7827.84 402.022 17

-5 -7 -9 7532.73 375.767 21

-5 -7 -10 5545.47 289.150 15

-5 -7 -10 6096.37 289.986 26

-5 -7 -10 4532.77 285.160 5

-5 7 -10 6046.95 321.224 17

-5 7 -10 4559.83 321.132 2

-5 -7 -10 5476.17 285.578 21

-5 -7 -11 4186.24 230.855 21

-5 -7 -11 4021.73 233.606 15

-5 -7 -11 4036.05 233.447 26

-5 7 -11 4225.58 267.702 17

-5 -7 -12 410.110 48.0393 10

-5 -7 -12 448.819 80.0440 28

-5 -7 -12 500.101 48.9630 8

-5 -7 -12 452.256 80.8564 15

-5 -7 -12 437.026 77.2714 21

-5 -7 -13 176.530 66.8248 21

-5 -7 -13 136.336 35.2688 8

-5 -7 -13 130.000 56.2841 18

-5 -7 -13 150.375 66.2967 15

-5 -7 -13 160.935 72.7577 28

-5 7 -13 175.068 59.3112 24

-5 -7 -14 1517.70 125.834 28

-5 -7 -14 1557.91 110.860 18

-5 -7 -14 1690.70 122.831 15

-5 7 -14 1547.10 115.334 24

-5 -7 -15 853.127 88.0450 15

-5 -7 -15 735.748 94.6361 28

-5 7 -15 920.956 82.3826 24

-5 -7 -16 56.7807 68.4443 28

-5 7 -16 20.2271 44.6651 24

-5 -7 -17 1129.99 102.675 28

-5 7 -17 1237.80 87.6610 24

5 -9 -19 2.98341 78.7245 28

5 9 -19 103.572 44.5351 22

5 -9 -18 1739.65 143.566 28

5 9 -18 1858.67 110.009 22

5 -9 -18 1544.04 127.835 23

5 -9 -17 83.9790 78.7728 23

5 -9 -16 1490.03 136.424 23

5 -9 -16 1412.96 128.101 26

5 9 -15 15.9356 52.6316 24

5 -9 -15 15.0392 90.2497 23

5 -9 -15 118.418 82.7334 26

5 -9 -14 347.244 89.7632 26

5 9 -14 293.649 61.9615 24

5 -9 -13 3614.29 211.298 20

5 -9 -13 3713.03 209.504 18

5 -9 -12 7753.57 422.678 25

5 -9 -12 8399.26 401.970 20

5 9 -12 7074.76 392.963 17

5 -9 -12 8459.10 402.585 18

5 -9 -11 426.103 107.329 25

5 9 -11 401.722 60.4901 24

5 -9 -11 502.158 69.9485 20

5 -9 -11 533.284 75.4916 18

5 9 -11 314.767 64.1038 17

5 -9 -10 1127.73 98.4365 18

5 -9 -10 1162.59 109.161 26

5 -9 -10 1228.12 131.237 25

5 9 -10 1114.42 90.0926 24

5 -9 -9 586.597 82.1101 11

5 9 -9 580.987 92.6633 13

5 -9 -9 677.768 86.4283 21

5 -9 -9 645.727 108.722 25

5 9 -9 586.835 64.1121 24

5 -9 -8 56533.2 2558.91 21

5 9 -8 56370.2 2552.10 24

5 -9 -8 54114.7 2580.27 11

5 9 -8 50335.1 2587.07 13

5 -9 -8 55866.3 2587.65 25

5 9 -7 544.869 57.8808 24

5 -9 -7 516.152 66.8654 18

5 9 -7 358.413 76.6950 13

5 -9 -7 562.695 74.3035 21

5 9 -6 7036.75 347.980 24

5 9 -6 6903.71 387.957 13

5 -9 -6 7124.65 353.201 18

5 -9 -6 7062.60 354.476 21

5 9 -5 12867.8 609.776 24

5 9 -5 10923.5 643.535 13

-5 9 5 12534.0 627.313 13

5 -9 -5 13668.0 615.867 18

5 -9 -5 12998.8 614.940 21

5 9 -4 26994.4 1210.34 24

5 -9 -4 25912.5 1212.09 21

5 -9 -4 25712.0 1212.73 18

-5 9 4 26922.3 1233.64 13

5 -9 -4 23599.5 1234.02 12

5 9 -4 25463.2 1254.96 13

5 -9 -3 922.079 108.177 12

-5 9 3 975.373 96.8038 13

5 -9 -3 1432.32 89.3324 21

5 -9 -3 1299.47 87.3696 18

5 -9 -2-19.1018 81.8015 12

-5 9 2 4.04089 49.6221 13

5 -9 -2 28.6895 37.7514 21

5 -9 -2 7.71192 36.8754 18

5 -9 -1 2130.38 193.750 12

-5 9 1 2758.22 166.524 13

5 9 -1 2649.19 189.784 13

5 -9 -1 2416.15 136.760 21

5 -9 -1 2537.04 139.681 18

5 -9 0 10117.6 499.800 18

5 -9 0 10001.9 495.577 21

-5 -9 0 10633.6 503.735 15

5 9 0 12578.4 561.967 13

-5 9 0 11839.5 533.930 13

5 -9 0 9207.22 572.149 12

-5 -9 -1 2485.53 148.487 15

5 9 1 2636.19 193.937 13

-5 9 -1 2847.67 173.827 13

-5 -9 -2 3000.11 182.601 21

-5 9 -2 3240.91 172.655 16

-5 -9 -2 3410.68 181.738 15

5 9 2 3516.69 224.654 13

-5 -9 -3 3338.60 186.549 15

-5 9 -3 3263.98 177.654 16

5 9 3 3545.40 227.229 13

-5 -9 -3 3230.16 187.883 21

-5 9 -4 2397.06 145.314 16

-5 -9 -4 2582.44 152.659 15

5 9 4 2609.92 188.734 13

-5 -9 -4 2298.52 157.785 21

-5 -9 -4 2773.17 145.901 26

-5 -9 -5 2540.10 140.318 26

5 9 5 2303.31 178.273 13

-5 9 -5 2275.57 142.216 16

-5 -9 -5 2257.48 148.301 15

-5 -9 -6 1959.58 119.588 26

5 9 6 1735.11 150.830 13

-5 -9 -6 1764.58 127.565 15

-5 9 -6 1713.48 125.855 16

5 -9 6 1762.96 136.365 11

-5 9 -7 182.031 70.3244 16

-5 -9 -7 198.638 67.0608 15

5 9 7 196.688 71.5022 13

-5 9 -7 245.148 88.5198 17

-5 -9 -7 198.458 71.8413 21

-5 -9 -7 252.239 59.3308 26

5 -9 7 310.213 67.1252 11

-5 -9 -8 4588.38 258.468 15

-5 9 -8 4314.71 276.587 17

-5 -9 -8 4945.25 252.533 26

-5 -9 -8 4575.13 257.288 21

-5 -9 -9 3777.44 223.659 15

-5 9 -9 3550.24 208.786 24

-5 -9 -9 4217.91 218.389 26

-5 -9 -9 3849.47 222.733 21

-5 9 -9 3547.40 241.230 17

-5 9 -10 1188.19 100.090 24

-5 9 -10 1320.54 140.500 17

-5 -9 -10 1251.58 111.609 26

-5 -9 -10 1451.39 117.712 21

-5 9 -11 622.909 77.8894 24

-5 9 -11 577.863 114.861 17

-5 -9 -11 584.677 87.8126 26

-5 -9 -11 707.796 92.2094 21

-5 9 -12 5167.21 280.677 24

-5 9 -12 5659.22 316.195 17

-5 -9 -12 5043.85 286.793 21

-5 -9 -12 5192.83 282.936 18

-5 -9 -12 5546.45 292.709 15

-5 9 -13 1637.77 121.765 24

-5 9 -13 1865.25 162.202 17

-5 -9 -13 1731.40 126.330 18

-5 -9 -13 1947.98 136.406 15

-5 -9 -13 1818.54 132.361 21

-5 -9 -14-25.5378 53.6050 18

-5 9 -14 52.3902 94.0936 17

-5 -9 -14 120.863 64.8535 15

-5 9 -15 147.953 93.9343 17

-5 9 -16 1303.54 122.370 17

-5 9 -16 1145.48 87.3462 24

-5 9 -17 223.218 78.6210 17

-5 9 -17 149.709 44.4171 24

5 11 -18 34.7757 37.7206 22

5 -11 -17 726.389 91.5623 28

5 11 -17 883.734 69.4545 22

5 -11 -16 31.1780 74.8226 26

5 -11 -16 43.2578 64.4855 23

5 11 -16 37.9236 41.0412 22

5 -11 -15 178.222 75.0096 23

5 11 -15 144.729 45.7634 22

5 11 -14 264.708 52.0026 22

5 -11 -13 2887.97 192.245 26

5 -11 -13 2996.67 181.262 20

5 -11 -12 364.609 91.2997 26

5 11 -12 408.935 63.2505 24

5 -11 -12 332.033 71.3071 18

5 -11 -12 327.002 70.9255 20

5 -11 -11 1761.25 139.517 26

5 -11 -11 1475.68 143.038 25

5 11 -11 1833.32 118.181 24

5 -11 -11 1627.07 125.747 18

5 -11 -11 1603.27 121.577 20

5 -11 -10 471.371 90.1439 26

5 -11 -10 580.531 108.853 25

5 11 -10 563.596 70.7006 24

5 -11 -10 499.048 74.8166 20

5 -11 -9 191.745 75.6975 21

5 -11 -9 193.309 77.7201 26

5 -11 -9 252.038 68.7970 18

5 11 -9 252.737 76.9651 13

5 -11 -9 253.286 102.962 25

5 11 -9 294.612 56.8427 24

5 11 -8 5450.36 273.592 24

5 -11 -8 5092.80 279.149 21

5 -11 -8 5139.69 310.187 25

5 -11 -8 5517.50 288.542 26

5 -11 -8 4600.97 299.582 11

5 11 -8 5054.56 321.524 13

5 -11 -8 5213.96 278.508 18

5 11 -7 7588.22 364.493 24

5 11 -7 6889.99 411.161 13

5 -11 -7 6606.09 395.178 11

5 -11 -7 7167.58 368.673 18

5 -11 -7 7394.59 369.678 21

5 11 -6 1628.94 106.590 24

5 -11 -6 1476.51 113.177 21

5 -11 -6 1460.40 114.149 18

5 11 -6 1452.07 147.052 13

5 -11 -5 1503.11 111.799 21

5 -11 -5 1554.00 113.789 18

5 11 -5 1583.77 149.707 13

-5 -11 4 1764.63 124.319 21

5 -11 -4 2000.22 126.944 21

5 -11 -4 1956.72 126.517 18

5 11 -4 2168.12 178.695 13

5 -11 -3 10182.5 478.072 21

-5 -11 3 8856.15 479.311 21

5 -11 -3 10331.0 481.793 18

-5 -11 2 7420.00 389.913 21

5 -11 -2 7444.06 381.876 21

5 -11 -2 8172.45 388.129 18

5 11 -2 8737.84 450.578 13

5 -11 -1 4526.03 244.475 21

-5 -11 1 4956.72 260.098 21

5 -11 -1 4843.51 251.670 18

5 11 -1 5503.20 312.913 13

-5 -11 0 1931.94 132.089 21

5 11 0 1674.52 166.767 13

-5 11 -1 8102.74 400.802 16

-5 -11 -1 7822.02 409.013 21

5 11 1 8651.19 460.659 13

-5 11 -2 3393.03 184.462 16

-5 -11 -2 3066.88 190.731 15

-5 -11 -2 3092.25 193.060 21

5 11 2 3604.61 236.522 13

-5 11 -3 10523.2 510.493 16

-5 -11 -3 10144.7 517.251 15

5 11 3 10760.9 562.464 13

-5 -11 -3 10371.0 518.225 21

-5 11 -4 2132.95 146.467 16

-5 -11 -4 2605.95 159.921 15

-5 -11 -4 2318.31 155.837 21

5 11 4 2175.34 181.099 13

-5 11 -4 2360.78 138.228 24

-5 11 -5 4863.10 258.372 24

-5 -11 -5 4400.94 270.794 21

-5 11 -5 5142.03 270.498 16

-5 -11 -5 5338.26 276.634 15

-5 -11 -5 5079.75 259.199 26

5 11 5 4773.71 304.374 13

5 -11 5 4856.81 293.721 11

-5 11 -6 2864.37 164.575 24

-5 11 -6 2712.63 192.792 17

-5 11 -6 2698.34 179.896 16

-5 -11 -6 2859.57 179.490 15

-5 -11 -6 3164.40 168.220 26

5 -11 6 2447.42 187.826 11

5 11 6 2482.76 198.045 13

-5 -11 -7 10530.9 519.198 15

-5 -11 -7 10513.7 504.055 26

-5 11 -7 10331.6 503.520 24

-5 11 -7 9314.10 521.306 17

-5 -11 -8 767.619 101.347 21

-5 -11 -8 687.296 95.4095 15

-5 -11 -8 773.722 86.1157 26

-5 11 -8 688.926 79.0895 24

-5 11 -8 671.325 109.538 17

-5 -11 -9 93.8783 77.8152 21

-5 -11 -9-20.4719 69.8184 15

-5 -11 -9 148.219 68.9034 26

-5 11 -9 70.3519 90.6057 17

-5 11 -9 33.9201 52.5202 24

-5 11 -10 83.3436 57.9837 24

-5 -11 -10 105.256 73.6396 15

-5 -11 -10 37.1920 73.1715 21

-5 11 -10 91.8360 87.8902 17

-5 11 -11 3526.64 205.808 24

-5 -11 -11 3610.44 210.509 18

-5 11 -11 3561.51 228.760 17

-5 -11 -11 3875.43 217.388 21

-5 11 -12 637.751 78.6601 24

-5 11 -12 888.037 104.541 17

-5 -11 -12 684.070 83.3996 18

-5 -11 -12 770.561 90.6757 21

-5 11 -13 420.995 64.8947 24

-5 -11 -13 409.457 66.2201 18

-5 11 -13 245.212 86.6497 17

-5 11 -14 36.3890 51.3511 24

-5 -11 -14 90.9472 52.6175 18

-5 11 -14-6.74957 72.6130 17

-5 11 -15 446.624 57.4697 24

5 13 -15 195.989 40.4921 22

5 -13 -14 1936.82 131.263 26

5 13 -14 1746.99 106.800 22

5 -13 -13 72.7120 72.6412 26

5 -13 -13 61.1359 59.3687 23

5 13 -13 83.3218 40.9050 22

5 13 -12 668.446 61.6005 22

5 -13 -12 663.793 94.5136 26

5 -13 -12 666.397 81.6587 20

5 13 -11 48.6651 38.6202 22

5 -13 -11-3.04689 60.7770 20

5 -13 -11 132.747 73.3195 26

5 -13 -10 7.85115 73.1832 25

-5 13 10-2.04793 39.3568 16

5 13 -10 27.6753 37.9394 22

5 -13 -10 94.6772 63.7918 20

5 -13 -10 126.889 77.8769 26

-5 13 9 73.1854 43.4059 16

5 -13 -9 150.865 75.9568 26

5 -13 -9 145.634 86.2897 25

5 13 -9 120.576 39.0098 22

5 13 -8 134.356 39.2020 22

-5 13 8 118.229 48.2866 16

5 -13 -8 101.226 75.1621 26

5 -13 -8 68.1659 90.2529 25

5 -13 -8 125.041 63.0833 18

5 -13 -7 45.9740 64.0201 21

5 -13 -7 68.6619 60.3601 20

-5 13 7-7.41628 47.5662 16

5 -13 -7 14.4114 71.5567 26

5 -13 -7 47.7011 95.4116 25

5 -13 -7 69.2553 63.0019 18

-5 13 6 1473.10 100.016 16

5 -13 -6 1262.84 116.160 26

5 -13 -6 1309.27 145.506 25

5 -13 -6 1149.72 103.257 21

5 -13 -6 1422.98 110.411 18

5 -13 -5 206.557 77.8919 11

5 -13 -5 226.477 59.2935 21

5 13 -5 262.434 91.3358 13

5 -13 -5 119.890 60.3348 18

-5 13 4 718.455 74.5207 16

5 -13 -4 617.237 101.306 11

5 -13 -4 540.413 70.9429 21

5 13 -4 604.882 113.069 13

-5 13 3 85.9659 56.1545 16

5 -13 -3 43.0697 50.4869 21

-5 -13 3 101.045 54.6753 21

-5 -13 2 7554.44 423.961 21

5 -13 -2 8076.27 417.613 21

5 13 -2 9717.84 498.839 13

-5 13 2 9975.47 430.893 16

-5 -13 1 110.015 62.7948 21

5 13 -1 104.627 81.3056 13

-5 13 1 125.409 62.5282 16

-5 -13 0 6.54971 61.4950 21

5 -13 0 71.6290 48.9294 20

5 13 0 25.9301 79.0082 13

-5 -13 0-17.7924 51.3328 18

5 13 1 100.951 84.2674 13

-5 13 -1 238.463 66.8597 16

-5 -13 -1 209.066 69.1061 21

5 -13 1 230.926 78.6997 11

5 -13 1 223.765 51.5088 20

-5 -13 -1 301.713 63.6547 18

-5 -13 -2 7885.24 436.445 21

-5 -13 -2 8339.12 435.204 18

-5 13 -2 9841.90 442.551 16

5 -13 2 8065.00 470.707 11

5 13 2 9645.37 497.204 13

5 13 3 31.7136 72.9363 13

-5 -13 -3 131.130 74.3799 21

-5 -13 -3 37.3106 60.1198 18

-5 13 -3 3.53692 64.1944 16

5 -13 3 48.9283 67.3337 11

-5 -13 -4 385.332 78.5076 18

-5 13 -4 589.222 89.3650 16

-5 -13 -4 448.620 88.1028 21

-5 -13 -5 119.712 70.5384 18

-5 13 -5 122.089 78.4767 16

-5 -13 -5 160.942 76.7131 21

-5 13 -6 8320.47 422.603 24

-5 -13 -6 8889.69 439.261 21

-5 -13 -6 8456.12 434.500 18

-5 -13 -7 28.0530 70.1511 18

-5 13 -7 70.8414 50.7846 24

-5 -13 -7 123.102 78.7868 21

-5 13 -8 1751.95 115.922 24

-5 -13 -8 1593.24 126.610 18

-5 -13 -9 440.020 73.5047 18

-5 -13 -9 359.864 84.9336 21

-5 13 -9 483.048 64.8779 24

-5 13 -10 1316.39 102.937 24

-5 -13 -10 1623.42 120.750 21

-5 13 -11 149.784 53.5128 24

-5 -13 -11 201.650 69.4590 21

-5 13 -12 229.798 55.0460 24

-5 -13 -12 256.336 59.5677 18

-5 13 -13 56.0564 46.2481 24

-5 -13 -13-34.6630 49.8669 18

-5 13 -14 10.4999 42.3951 24

5 -15 -12 583.508 70.4099 26

5 -15 -11 42.6011 61.0611 26

5 -15 -11 54.0488 58.5365 20

5 -15 -10 119.395 64.8466 26

5 -15 -10 77.1645 58.6992 20

5 -15 -9 419.481 69.9850 26

-5 15 9 359.055 53.3888 16

5 -15 -8 324.711 64.0436 20

5 -15 -8 327.727 68.3375 26

-5 15 8 309.570 54.6058 16

5 -15 -8 272.825 68.2677 25

5 -15 -7 1087.52 94.3395 26

5 -15 -7 946.609 99.7230 25

5 -15 -7 1089.44 93.1825 20

-5 15 7 1138.33 87.0511 16

5 -15 -6 71.4290 54.3040 20

5 -15 -6 35.5415 60.6163 26

-5 15 6 54.6391 51.1676 16

5 -15 -6-18.6209 76.6944 25

5 -15 -5 628.806 75.4513 20

5 -15 -5 566.782 76.4753 26

-5 15 5 768.834 75.6382 16

5 -15 -5 533.672 100.839 25

5 -15 -4 79.1740 56.0502 20

5 -15 -4 88.7378 57.7682 26

-5 15 4 86.2980 57.9024 16

5 -15 -4 32.6009 93.1414 25

5 15 -3 1135.82 104.201 16

5 -15 -3 1071.35 93.3740 26

5 -15 -3 1085.72 93.2448 20

5 -15 -2 256.284 59.2022 20

-5 15 2 300.175 69.9218 16

-5 -15 1 503.018 71.2281 21

5 -15 -1 595.501 68.9805 20

-5 -15 0 2.85819 58.3734 21

5 -15 0 69.0009 50.9746 20

-5 15 -1 3677.40 189.670 16

-5 -15 -1 2420.91 170.053 21

-5 -15 -2 229.195 66.3769 18

-5 -15 -2 224.621 69.0951 21

-5 15 -2 328.763 79.9232 16

-5 -15 -3 1919.38 144.485 21

-5 -15 -3 2095.21 143.420 18

-5 -15 -4-12.4879 58.7069 18

-5 -15 -4 21.7352 64.6928 21

-5 15 -4 76.1130 79.2806 16

-5 -15 -5 731.365 87.3675 21

-5 -15 -5 733.375 84.1694 18

-5 -15 -6 235.868 67.0583 18

-5 -15 -6 239.372 69.1317 21

-5 -15 -7 434.020 71.8270 18

-5 -15 -7 469.379 76.2460 21

-5 -15 -8 84.5105 59.1682 18

-5 -15 -8 102.065 63.6334 21

-5 -15 -9 55.3515 54.0501 18

-5 17 4 389.792 68.1292 16

-5 17 3 152.053 64.5908 16

-5 17 2 82.2162 69.9227 16

-5 17 0 253.720 76.5441 16

5 17 0 166.267 63.2188 16

5 17 1 108.558 59.8529 16

5 -17 1 112.153 61.9888 25

5 17 2 23.4717 58.5760 16

5 17 3 68.9334 55.3761 16

6 0 -22 590.111 58.3175 8

6 0 -21 16.8174 44.7668 8

6 0 -20 144.481 130.739 23

6 0 -20-12.3501 124.656 28

6 0 -20-21.0360 50.3787 8

6 0 -19-104.719 147.368 23

6 0 -19 160.294 79.5063 7

6 0 -19 55.7689 133.504 28

6 0 -19-47.9315 50.3501 8

6 0 -18 702.977 94.0470 9

6 0 -18 642.461 165.070 23

6 0 -18 571.600 87.3704 7

6 0 -18 834.657 74.8326 8

6 0 -18 630.470 151.922 28

6 0 -17 67.4887 135.399 28

6 0 -17 48.1154 71.1467 9

6 0 -17 141.483 156.934 23

6 0 -17-3.70262 65.9123 7

6 0 -17-23.1012 69.9569 5

6 0 -17 20.8423 49.6929 8

6 0 -16 3678.35 284.114 28

6 0 -16 3759.12 300.545 23

6 0 -16 3927.18 240.958 9

6 0 -16 4726.79 227.300 8

6 0 -16 4058.07 238.131 7

6 0 -16 4406.96 275.699 5

6 0 -15 23.2292 57.6288 7

6 0 -15 183.176 122.689 28

6 0 -15 23.8163 43.9357 8

6 0 -15-88.0549 69.3876 5

6 0 -15 17.9087 62.7240 9

6 0 -15-34.5515 85.8701 4

6 0 -15 28.3752 41.8895 22

6 0 -14 12737.9 578.558 8

6 0 -14 12987.5 627.207 5

6 0 -14 10541.2 634.193 4

6 0 -14 13073.5 691.841 2

6 0 -14 12404.3 591.232 9

6 0 -14 11772.3 586.950 7

6 0 -14 11393.7 574.616 22

6 0 -13 25.8411 32.4834 22

6 0 -13-7.25623 79.5599 4

6 0 -13-50.6606 116.155 2

6 0 -13-11.0429 57.3782 5

6 0 -13-22.8992 45.7620 6

6 0 -13-35.0323 53.0399 9

6 0 -13 21.6124 47.3085 7

6 0 -13 1.97982 37.4922 8

6 0 -12 7904.93 380.077 6

6 0 -12 7824.97 462.852 2

6 0 -12 8468.99 415.044 5

6 0 -12 7714.43 429.826 4

6 0 -12 7136.22 384.154 9

6 0 -12 8632.57 378.255 8

6 0 -12 6831.12 380.859 7

6 0 -11 16.3068 32.7291 6

6 0 -11 86.7096 73.8101 4

6 0 -11-4.29003 47.8379 5

6 0 -11 63.3217 105.692 2

6 0 -10 27762.4 1325.23 17

6 0 -10 27590.9 1314.40 6

6 0 -10 31337.4 1344.08 5

6 0 -10 26939.8 1353.31 4

6 0 -10 30207.2 1381.59 2

6 0 -9 26.0387 40.7989 5

6 0 -9 35.5580 62.1838 4

6 0 -9 11.0249 51.5498 17

6 0 -9 6.00476 97.5536 2

6 0 -9 48.0097 29.6713 6

6 0 -8 64544.1 2976.48 2

6 0 -8 59289.2 2958.41 4

6 0 -8 66778.7 2950.10 5

6 0 -7 73.0746 54.8835 4

6 0 -7-9.04073 74.4290 2

6 0 -7 24.5798 35.4660 5

6 0 -6 105136. 4853.87 4

6 0 -6 105296. 4842.22 5

6 0 -5 13.0464 41.2290 3

6 0 -5 8.97763 45.0098 4

6 0 -5 17.8186 28.9667 5

6 0 -4 91374.1 4223.10 12

6 0 -4 91539.8 4218.05 3

6 0 -4 91687.4 4231.99 11

6 0 -3 72.0146 60.6562 12

6 0 -3 173.453 53.5831 3

6 0 -3 68.3516 78.3493 11

6 0 -2 46882.3 2239.11 12

6 0 -2 49062.1 2232.90 3

6 0 -1 21.1559 81.5519 12

6 0 0 143243. 6609.32 11

6 0 1 67.9213 93.1586 11

6 0 2 91571.8 4241.61 11

6 0 3 3.95036 60.3396 11

-6 0 -3-18.9924 68.6923 1

-6 0 -4 3887.23 232.253 1

-6 0 -5 21.1371 70.1482 2

-6 0 -5 89.4371 86.2080 1

-6 0 -6 20787.0 1012.80 2

-6 0 -6 20995.7 1020.08 1

-6 0 -7 115.771 107.117 1

-6 0 -7 50.2393 95.6870 2

-6 0 -8 5533.03 320.373 2

-6 0 -8 6056.45 333.934 1

-6 0 -8 4881.35 277.096 17

-6 0 -9 35.2076 65.2631 17

-6 0 -9-40.3093 46.7556 5

-6 0 -9-15.1699 106.088 1

-6 0 -9-63.4125 100.076 2

-6 0 -10 2057.55 196.479 1

-6 0 -10 2290.23 121.147 8

-6 0 -10 2153.40 148.218 5

-6 0 -10 1795.04 146.562 17

-6 0 -10 2137.19 188.419 2

-6 0 -10 2138.06 121.129 10

-6 0 -11 81.5232 81.6666 28

-6 0 -11 29.0786 111.978 1

-6 0 -11-28.2011 114.647 2

-6 0 -11 12.5206 82.1187 17

-6 0 -11 6.40450 39.7335 8

-6 0 -11 10.0682 63.0112 23

-6 0 -11-6.72162 40.7707 10

-6 0 -11-5.17435 55.0257 5

-6 0 -12 438.985 56.9540 10

-6 0 -12 396.480 104.896 17

-6 0 -12 374.048 77.6484 5

-6 0 -12 359.678 52.6292 8

-6 0 -12 401.056 92.3545 28

-6 0 -12 330.348 134.669 2

-6 0 -13-39.8144 47.2475 10

-6 0 -13-51.3876 111.487 17

-6 0 -13 58.4622 67.1852 5

-6 0 -13 40.0212 44.5963 8

-6 0 -13 20.0978 93.9249 28

-6 0 -13-11.6806 128.070 2

-6 0 -14 1110.24 86.9877 10

-6 0 -14 943.631 134.854 17

-6 0 -14 949.551 113.659 5

-6 0 -14 982.273 83.5438 8

-6 0 -14 780.875 128.978 28

-6 0 -14 1013.87 188.608 2

-6 0 -15 28.7126 116.364 17

-6 0 -15 7.16909 67.8908 5

-6 0 -15-35.0679 52.9491 8

-6 0 -15-51.9993 138.869 2

-6 0 -15-39.5171 51.0580 10

-6 0 -16 194.733 56.9504 10

-6 0 -16 115.117 50.9752 8

-6 0 -16 195.241 116.427 17

-6 0 -17 87.3410 52.7483 10

-6 0 -17-2.54905 49.0836 8

-6 0 -18 60.8636 44.7930 8

-6 0 -19 59.4364 43.6750 8

-6 0 -20 294.936 45.6598 8

-6 0 -21 8.28922 29.1822 8

6 2 -21 450.850 56.3751 8

6 -2 -21 540.379 58.6904 8

6 -2 -21 480.510 121.308 28

6 -2 -20 508.812 132.779 23

6 -2 -20 529.496 62.5148 8

6 2 -20 576.493 64.6373 8

6 -2 -20 497.595 132.426 28

6 -2 -19 68.3296 132.809 23

6 -2 -19 36.7741 45.2433 8

6 -2 -19 533.913 86.4387 7

6 2 -19 628.041 93.7536 7

6 2 -19 26.9764 45.9962 8

6 -2 -19 58.2407 130.127 28

6 -2 -18 33.2050 47.8434 8

6 2 -18 43.5238 49.2606 8

6 -2 -18 109.563 121.311 28

6 2 -18-46.8427 143.375 28

6 2 -18 88.2696 71.1351 7

6 -2 -18-43.4362 69.3391 7

6 -2 -18 87.8725 128.029 23

6 2 -17 833.204 97.0561 7

6 -2 -17 1047.40 101.597 7

6 -2 -17 975.637 102.479 9

6 2 -17 946.349 103.835 9

6 2 -17 923.150 80.0025 8

6 -2 -17 877.971 78.5911 8

6 2 -17 941.022 120.811 5

6 -2 -17 768.268 115.542 5

6 2 -17 556.607 163.757 28

6 -2 -17 1153.55 158.402 28

6 2 -17 752.345 74.8863 22

6 -2 -17 878.415 152.934 23

6 2 -16 1745.93 132.997 7

6 -2 -16 1850.32 134.746 7

6 2 -16 1727.23 196.352 28

6 2 -16 2054.24 141.266 9

6 -2 -16 1939.93 143.122 9

6 2 -16 1849.33 120.507 8

6 -2 -16 1851.35 119.574 8

6 2 -16 1743.72 160.523 5

6 -2 -16 1739.65 160.931 5

6 -2 -16 2133.41 204.506 23

6 2 -16 1929.44 118.532 22

6 -2 -15 796.994 130.345 4

6 2 -15 657.725 122.283 4

6 2 -15 657.948 84.2013 7

6 -2 -15 839.855 88.9872 7

6 2 -15 730.457 148.000 28

6 -2 -15 823.664 110.176 27

6 2 -15 754.248 66.1743 22

6 2 -15 688.495 105.828 5

6 -2 -15 756.642 107.819 5

6 -2 -15 643.486 94.7375 26

6 -2 -15 735.332 87.7620 9

6 2 -15 760.287 91.2983 9

6 2 -15 748.396 69.3853 8

6 -2 -15 813.918 70.6521 8

6 -2 -15 706.569 137.525 23

6 2 -15 614.474 166.426 23

6 2 -14 15245.6 653.721 8

6 -2 -14 14782.6 652.691 8

6 -2 -14 14771.3 699.016 5

6 2 -14 15122.2 702.159 5

6 2 -14 12201.4 709.887 4

6 -2 -14 13300.0 714.139 4

6 2 -14 13882.6 675.540 17

6 2 -14 13554.0 661.915 7

6 -2 -14 13867.9 661.449 7

6 -2 -14 14542.4 676.845 27

6 2 -14 14939.5 681.507 27

6 2 -14 9675.81 688.961 28

6 -2 -14 12418.4 663.884 26

6 2 -14 13587.2 664.296 9

6 -2 -14 14385.2 665.883 9

6 2 -14 13020.0 648.216 22

6 -2 -14 14670.2 709.682 23

6 2 -14 11969.4 714.555 23

6 2 -14 12795.4 645.128 24

6 2 -13 6556.43 303.408 8

6 -2 -13 6487.67 302.155 8

6 2 -13 6498.63 401.818 2

6 -2 -13 6548.64 401.460 2

6 -2 -13 6288.68 324.719 27

6 2 -13 6605.67 329.480 27

6 2 -13 4555.28 341.116 28

6 2 -13 5831.18 320.901 17

6 -2 -13 5791.71 310.147 7

6 2 -13 5528.40 308.222 7

6 -2 -13 5489.91 312.702 26

6 2 -13 6731.09 347.232 5

6 -2 -13 6886.21 345.894 5

6 2 -13 6005.93 359.990 4

6 -2 -13 5599.54 356.472 4

6 -2 -13 6230.34 313.591 9

6 2 -13 5988.23 313.260 9

6 -2 -13 6050.79 305.508 6

6 2 -13 6262.36 306.985 6

6 2 -13 5878.03 297.445 24

6 2 -13 5393.97 367.272 23

6 2 -12 233.765 66.3220 17

6 -2 -12 75.2132 44.4321 7

6 2 -12 166.657 48.8568 7

6 2 -12 54.4905 56.4545 5

6 -2 -12 63.3602 56.6341 5

6 -2 -12 66.0897 39.6373 6

6 2 -12 76.7702 40.5804 6

6 -2 -12 186.911 54.0166 9

6 2 -12 212.154 52.4651 9

6 2 -12 443.587 67.7709 27

6 -2 -12 301.249 68.3848 27

6 2 -12 292.615 95.7141 28

6 2 -12 99.8469 116.049 2

6 -2 -12 98.5384 116.304 2

6 2 -12 185.292 115.659 23

6 2 -12 141.006 37.6368 8

6 -2 -12 59.6973 34.8554 8

6 2 -12 86.2395 76.8792 4

6 -2 -12 216.727 85.3673 4

6 2 -11 15866.9 859.497 4

6 -2 -11 16969.9 860.731 4

6 2 -11 18589.1 848.313 5

6 -2 -11 18259.9 846.852 5

6 2 -11 16188.0 826.811 17

6 2 -11 18918.8 893.957 2

6 -2 -11 18243.0 890.946 2

6 -2 -11 17282.3 817.026 6

6 2 -11 17148.2 816.956 6

6 -2 -11 17982.1 830.160 27

6 2 -10 16461.8 765.166 2

6 -2 -10 15671.6 759.732 2

6 2 -10 14469.3 705.188 17

6 2 -10 16522.3 726.448 5

6 -2 -10 15086.0 722.724 5

6 2 -10 14562.5 735.626 4

6 -2 -10 14077.7 734.986 4

6 -2 -10 14160.0 695.627 6

6 2 -10 14676.9 696.471 6

6 2 -9 217.161 52.9852 5

6 -2 -9 143.879 47.9640 5

6 2 -9 149.963 64.8218 4

6 -2 -9 242.974 70.4742 4

6 2 -9 103.989 93.2390 2

6 -2 -9 246.601 91.6933 2

6 2 -9 240.464 49.6849 17

6 -2 -9 181.813 33.4431 6

6 2 -9 202.466 34.4961 6

6 2 -8 16676.7 706.958 5

6 -2 -8 16950.5 707.260 5

6 2 -8 14558.1 715.418 4

6 -2 -8 15225.4 717.721 4

6 2 -8 15079.8 732.261 2

6 -2 -8 15772.2 734.412 2

6 -2 -8 15516.5 709.435 11

6 -2 -7 45853.8 2109.40 11

6 2 -7 41620.2 2067.75 13

6 2 -7 48796.9 2086.16 5

6 -2 -7 47185.4 2084.83 5

6 2 -7 44673.8 2095.42 4

6 -2 -7 46218.9 2096.33 4

6 -2 -6 63792.5 3157.47 11

6 2 -6 64684.5 3126.58 13

6 2 -6 67241.2 3146.72 4

6 -2 -6 70023.7 3148.11 4

6 2 -6 73635.4 3138.33 5

6 -2 -6 69356.9 3136.31 5

6 2 -5 2743.36 148.518 3

6 -2 -5 2326.82 142.972 3

6 -2 -5 2173.27 149.711 11

6 -2 -5 2390.23 149.821 4

6 2 -5 2547.34 151.461 4

6 -2 -4 2760.19 167.451 11

6 -2 -4 3087.10 164.670 3

6 2 -4 2927.08 164.479 3

6 -2 -3 27217.0 1275.35 11

6 2 -3 27367.7 1276.69 3

6 -2 -3 27152.1 1275.60 3

6 2 -2 5186.74 253.337 12

6 -2 -2 5311.11 289.852 12

6 2 -2 4752.51 260.350 3

6 -2 -2 4741.21 258.225 3

6 2 -1 30178.3 1340.86 12

6 -2 -1 26714.7 1347.62 3

6 2 -1 28931.8 1350.03 3

6 2 0 85633.7 4112.15 12

6 -2 0 93171.0 4113.94 11

6 -2 1 19091.3 905.299 11

6 -2 2 719.202 79.6388 11

-6 2 -3 40241.9 1922.41 1

-6 -2 -3 41649.6 1923.61 1

6 -2 3 41193.7 1924.18 11

-6 2 -4 5179.02 284.389 1

-6 -2 -4 5040.05 280.532 1

6 -2 4 4536.11 288.674 11

-6 2 -5 3375.92 223.961 1

-6 -2 -5 3910.85 227.047 1

6 -2 5 3221.60 237.553 11

-6 2 -5 3713.00 220.957 2

-6 -2 -5 3475.05 220.307 2

-6 2 -6 56043.7 2817.40 1

-6 -2 -6 60414.2 2823.22 1

-6 2 -6 56203.3 2813.89 2

-6 -2 -6 60009.0 2817.59 2

-6 2 -7 3390.83 196.383 17

-6 2 -7 3022.92 229.908 1

-6 -2 -7 3407.95 237.720 1

-6 -2 -7 3239.60 227.967 2

-6 2 -7 3031.38 221.805 2

-6 2 -8 3638.40 208.679 14

-6 2 -8 3666.91 217.687 17

-6 -2 -8 3835.20 212.128 17

-6 -2 -8 4547.17 267.225 1

-6 2 -8 4189.04 266.174 1

-6 -2 -8 3855.58 257.619 2

-6 2 -8 3699.42 252.911 2

-6 2 -9 743.917 76.3458 14

-6 2 -9 999.567 89.4291 5

-6 -2 -9 900.321 86.2653 5

-6 2 -9 773.197 101.991 17

-6 -2 -9 935.560 94.2016 17

-6 2 -9 809.554 132.038 1

-6 -2 -9 947.460 137.250 1

-6 2 -9 983.786 133.523 2

-6 -2 -9 1073.55 131.076 2

-6 2 -10 2139.52 191.810 2

-6 -2 -10 1988.77 188.858 2

-6 -2 -10 2418.58 129.360 10

-6 2 -10 2326.52 128.008 10

-6 2 -10 2110.56 150.102 5

-6 -2 -10 1922.05 147.310 5

-6 2 -10 2024.73 157.179 17

-6 -2 -10 2119.51 155.070 17

-6 2 -10 2369.27 127.179 8

-6 -2 -10 2478.05 127.910 8

-6 2 -10 1814.93 157.834 28

-6 -2 -10 1765.46 140.733 28

-6 2 -10 1994.39 195.846 1

-6 -2 -10 2092.36 203.260 1

-6 2 -11 1340.96 172.109 2

-6 2 -11 1529.39 94.5198 10

-6 -2 -11 1547.62 94.1851 10

-6 2 -11 1087.60 114.279 5

-6 -2 -11 1271.17 117.915 5

-6 -2 -11 1349.21 129.836 17

-6 2 -11 1302.32 135.200 17

-6 -2 -11 1331.49 174.863 1

-6 2 -11 911.996 172.108 1

-6 -2 -11 1214.96 171.550 2

-6 2 -11 1280.99 90.4105 8

-6 -2 -11 1400.55 91.6220 8

-6 2 -11 1150.62 134.495 28

-6 -2 -11 1199.90 111.911 28

-6 -2 -12 273.912 52.3252 10

-6 2 -12 258.146 50.3477 10

-6 2 -12 188.473 71.3859 5

-6 -2 -12 154.389 70.1849 5

-6 2 -12 464.290 109.221 17

-6 -2 -12 335.550 97.3542 17

-6 2 -12 180.810 129.842 2

-6 -2 -12 203.121 132.277 2

-6 2 -12 307.848 49.7640 8

-6 -2 -12 236.237 51.3292 8

-6 -2 -12 393.555 86.9532 28

-6 -2 -13 7.84343 44.0828 10

-6 2 -13-18.2332 46.5173 10

-6 2 -13 19.1971 61.9541 5

-6 -2 -13 19.3025 61.6137 5

-6 -2 -13 116.072 135.620 2

-6 2 -13 11.5482 126.620 2

-6 -2 -13 135.162 103.359 17

-6 2 -13 5.34731 111.139 17

-6 2 -13 12.2398 42.2261 8

-6 -2 -13 32.1781 41.6607 8

-6 -2 -13-57.8304 84.8399 28

-6 -2 -14 250.616 52.8792 8

-6 2 -14 182.230 51.8332 8

-6 2 -14 110.734 131.743 2

-6 -2 -14 160.747 130.620 2

-6 2 -14 218.999 55.1971 10

-6 -2 -14 195.271 53.2248 10

-6 -2 -14 218.081 53.8867 15

-6 -2 -14 128.718 106.819 17

-6 2 -14 289.170 118.748 17

-6 2 -14 183.217 75.2679 5

-6 -2 -14 204.970 74.8294 5

-6 -2 -14 76.3226 86.3398 28

-6 2 -15 666.200 133.277 17

-6 -2 -15 602.124 70.0508 10

-6 2 -15 854.279 75.7086 10

-6 2 -15 624.772 96.9743 5

-6 -2 -15 649.696 99.5666 5

-6 2 -15 588.251 165.256 2

-6 -2 -15 770.922 170.514 2

-6 2 -15 714.666 71.5278 8

-6 -2 -15 673.801 71.3223 8

-6 2 -15 399.341 60.0486 24

-6 -2 -15 635.783 115.592 28

-6 2 -16 31.0963 48.5171 8

-6 -2 -16 13.0147 49.4370 8

-6 -2 -16 10.8259 44.7267 10

-6 2 -16 40.5492 47.5793 10

-6 -2 -16-10.0196 95.5455 28

-6 2 -16-115.637 107.611 17

-6 2 -17 535.110 63.9417 8

-6 -2 -17 507.136 64.1989 8

-6 2 -17 556.689 125.138 17

-6 2 -18 67.5743 43.2734 8

-6 -2 -18 72.1438 44.7236 8

-6 2 -18 132.865 105.069 17

-6 2 -19 72.2196 42.4517 8

-6 -2 -19 83.7691 41.1200 8

-6 2 -20 56.3846 36.1976 8

-6 -2 -20 23.3471 34.3582 8

-6 -2 -21 5.08677 25.3702 8

-6 2 -21 24.7894 26.8524 8

6 -4 -21 347.271 108.715 28

6 -4 -21 479.305 99.1050 23

6 -4 -20 249.983 115.959 28

6 -4 -20 224.690 47.1215 8

6 4 -20 180.459 46.9362 8

6 -4 -20 101.163 110.820 23

6 -4 -19 214.223 122.334 28

6 -4 -19 344.405 118.585 23

6 4 -19 348.358 54.0690 8

6 -4 -19 414.421 54.0494 8

6 -4 -18 2147.79 194.881 28

6 4 -18 2231.07 135.275 22

6 -4 -18 1867.68 194.288 23

6 -4 -18 1987.68 129.559 8

6 4 -18 2063.28 130.683 8

6 4 -18 2203.43 152.638 7

6 4 -17 1881.01 125.711 22

6 4 -17 1878.06 141.056 7

6 4 -17 1976.58 125.297 8

6 -4 -17 2061.28 125.877 8

6 -4 -17 2252.02 142.839 7

6 -4 -17 2317.21 200.528 23

6 -4 -16 898.255 151.760 23

6 4 -16 921.394 76.8189 24

6 -4 -16 851.890 100.263 9

6 4 -16 867.059 96.9240 9

6 -4 -16 722.367 107.645 26

6 4 -16 969.599 80.3236 22

6 4 -16 1044.26 79.7397 8

6 -4 -16 850.809 75.8821 8

6 -4 -16 959.855 96.9095 7

6 4 -16 1100.93 99.8868 7

6 4 -15-9.27817 40.4168 8

6 -4 -15-18.2334 41.8545 8

6 4 -15-27.2115 65.8615 5

6 -4 -15-80.2877 73.0476 5

6 4 -15 30.9478 58.6971 7

6 -4 -15-43.3219 57.9682 7

6 4 -15-122.331 142.339 28

6 -4 -15-13.4923 79.9262 27

6 -4 -15-11.9233 73.7531 26

6 4 -15 44.4367 64.8470 9

6 -4 -15 21.7051 66.7313 9

6 4 -15 107.413 80.5513 17

6 4 -15 98.0664 155.879 23

6 4 -15 9.31767 41.5978 24

6 -4 -15-6.71927 111.854 23

6 4 -14 5507.78 306.288 17

6 -4 -14 5446.56 299.313 26

6 -4 -14 5780.13 295.340 9

6 4 -14 5836.82 298.258 9

6 4 -14 5530.18 279.790 24

6 4 -14 5397.72 292.628 7

6 -4 -14 5791.97 294.089 7

6 -4 -14 6019.75 309.470 27

6 4 -14 5862.82 328.909 5

6 4 -14 4910.68 339.733 4

6 -4 -14 5373.81 343.014 4

6 -4 -14 5528.74 322.761 5

6 4 -14 6079.57 282.161 8

6 -4 -14 5694.18 279.484 8

6 4 -13 1661.14 122.929 17

6 4 -13 1330.08 102.684 7

6 -4 -13 1344.99 101.682 7

6 -4 -13 1459.80 112.553 26

6 -4 -13 1389.03 103.927 9

6 4 -13 1415.51 107.383 9

6 4 -13 839.009 172.557 23

6 4 -13 1424.26 92.0180 24

6 -4 -13 1751.71 118.276 27

6 4 -13 579.167 146.963 28

6 4 -13 1317.28 87.8750 8

6 -4 -13 1319.22 86.5714 8

6 4 -13 1321.33 122.559 5

6 -4 -13 1245.09 119.038 5

6 4 -13 1201.35 141.149 4

6 -4 -13 1117.46 139.511 4

6 4 -12 643.712 57.4408 8

6 -4 -12 592.192 55.0558 8

6 4 -12 509.659 84.1880 5

6 -4 -12 540.584 81.3761 5

6 4 -12 1257.32 97.5882 27

6 -4 -12 1164.97 91.5567 27

6 -4 -12 850.207 78.8155 20

6 4 -12 743.657 83.0769 17

6 -4 -12 751.335 65.9679 18

6 -4 -12 643.229 68.1277 7

6 4 -12 469.127 66.5667 7

6 -4 -12 818.821 82.2574 26

6 -4 -12 760.337 74.8316 9

6 4 -12 670.284 71.2060 9

6 4 -12 560.745 145.956 23

6 4 -12 721.849 61.7820 24

6 -4 -12 524.728 58.5726 6

6 4 -12 471.758 59.7220 6

6 4 -12 591.018 107.863 4

6 -4 -12 448.844 96.2893 4

6 -4 -11 5588.46 296.202 20

6 4 -11 7063.91 330.400 5

6 -4 -11 6611.65 326.015 5

6 4 -11 5655.06 336.701 4

6 -4 -11 6199.68 341.871 4

6 -4 -11 5752.02 305.384 27

6 4 -11 5834.21 308.123 27

6 4 -11 5445.27 302.737 17

6 -4 -11 5501.68 292.103 18

6 -4 -11 5822.14 296.660 7

6 4 -11 5867.21 296.372 7

6 -4 -11 5444.66 300.181 26

6 -4 -11 5362.72 322.094 25

6 -4 -11 5947.20 299.017 9

6 4 -11 5591.34 297.866 9

6 4 -11 5669.69 290.863 24

6 4 -11 6548.30 295.017 6

6 -4 -11 6461.80 294.213 6

6 4 -11 6365.22 290.221 8

6 -4 -11 6348.58 289.224 8

6 -4 -10 11812.5 567.358 6

6 4 -10 12837.4 569.807 6

6 -4 -10 11620.2 570.751 20

6 -4 -10 10076.6 587.702 19

6 -4 -10 11446.1 597.175 25

6 4 -10 12124.8 577.681 17

6 -4 -10 11738.3 569.262 18

6 4 -10 11568.7 609.937 4

6 4 -10 13901.2 601.022 5

6 -4 -10 12666.0 595.875 5

6 -4 -10 12205.8 611.229 4

6 4 -9 1259.46 78.8274 6

6 -4 -9 1007.06 75.8887 6

6 4 -9 1270.53 100.996 5

6 -4 -9 1299.95 100.197 5

6 4 -9 1181.18 114.444 4

6 -4 -9 1193.53 115.394 4

6 4 -9 1562.75 94.0016 17

6 -4 -9 1549.34 86.2607 18

6 -4 -9 1475.42 85.9875 20

6 4 -9 1198.88 86.6404 13

6 -4 -9 1187.50 114.728 11

6 4 -8 2173.27 125.824 5

6 -4 -8 1827.38 118.542 5

6 4 -8 1799.90 134.026 4

6 -4 -8 1676.04 131.231 4

6 -4 -8 1841.56 105.636 18

6 -4 -8 1906.27 104.476 20

6 -4 -8 1761.07 123.458 11

6 4 -8 1615.12 108.121 13

6 -4 -8 1713.54 98.1799 6

6 4 -8 1758.89 99.1758 6

6 4 -7 2094.07 139.562 4

6 -4 -7 2253.15 141.182 4

6 4 -7 1845.09 119.383 13

6 -4 -7 2132.86 126.629 11

6 -4 -7 1917.92 110.365 20

6 -4 -7 2038.55 112.797 18

6 -4 -6 5065.12 286.035 12

6 -4 -6 5172.58 265.602 11

6 4 -6 4928.90 265.349 13

6 4 -6 5115.90 274.660 4

6 -4 -6 5548.45 276.500 4

6 4 -5 16851.5 828.709 13

6 -4 -5 18181.3 833.642 4

6 4 -5 17044.0 832.145 4

6 -4 -5 17745.3 826.588 11

6 -4 -5 17449.7 848.901 12

6 4 -4 40524.6 1960.09 4

6 -4 -4 43122.0 1960.94 4

6 -4 -4 43100.1 1955.91 11

6 -4 -4 40381.4 1981.26 12

6 -4 -4 41323.7 1956.40 3

6 4 -4 43577.3 1958.45 3

6 -4 -3 33436.8 1619.56 12

6 -4 -3 33414.3 1584.19 11

6 -4 -3 34484.6 1584.14 3

6 4 -3 34495.9 1585.63 3

6 -4 -2 411.468 49.7379 3

6 4 -2 318.551 53.7812 3

6 4 -1 17135.6 791.860 3

6 -4 -1 16069.1 788.412 3

6 -4 0 23446.4 1100.05 11

6 -4 1 970.922 75.9420 11

6 -4 2 36780.1 1757.09 11

-6 4 -2 37895.2 1769.78 1

-6 -4 -2 39134.0 1770.31 1

6 -4 3 109140. 5022.33 11

-6 4 -3 104225. 5035.38 1

-6 -4 -3 114111. 5037.98 1

6 -4 4 3737.72 205.773 11

-6 -4 -4 3876.74 220.375 2

-6 4 -4 3823.65 220.134 2

-6 4 -4 3444.33 221.957 1

-6 -4 -4 3945.26 228.133 1

6 -4 5 3309.24 178.720 11

-6 4 -5 2904.78 194.833 1

-6 -4 -5 2911.34 193.354 1

-6 -4 -5 3018.78 189.902 2

-6 4 -5 2603.06 184.911 2

-6 4 -6 47689.6 2204.61 1

-6 -4 -6 49120.9 2205.88 1

6 -4 6 44483.6 2188.03 11

-6 -4 -6 48312.6 2200.67 2

-6 4 -6 45233.7 2197.03 2

-6 -4 -7 6134.57 316.356 21

-6 -4 -7 6965.06 365.988 2

-6 4 -7 6752.38 363.122 2

6 -4 7 5876.79 363.028 11

-6 4 -7 6699.29 367.794 1

-6 -4 -7 7134.08 373.232 1

-6 4 -8 1674.22 151.362 2

-6 -4 -8 1313.88 140.503 2

-6 4 -8 1980.93 126.904 14

-6 -4 -8 1506.94 99.0372 21

-6 4 -8 1671.00 114.843 5

-6 -4 -8 1439.23 112.674 5

-6 4 -8 1708.99 157.500 1

-6 -4 -8 1520.21 153.399 1

-6 4 -8 1610.84 130.661 17

-6 4 -9 388.863 90.8447 17

-6 4 -9 331.710 109.163 1

-6 4 -9 325.856 107.284 2

-6 -4 -9 341.835 102.087 2

-6 -4 -9 411.044 116.604 1

-6 -4 -9 368.004 54.0503 21

-6 -4 -9 342.432 60.9476 5

-6 4 -9 597.446 69.1393 5

-6 4 -9 546.291 48.1357 8

-6 -4 -9 447.461 47.0847 8

-6 4 -9 544.382 50.4666 10

-6 -4 -9 457.421 47.0873 10

-6 4 -9 616.523 80.6292 14

-6 4 -10 10776.2 588.183 17

-6 4 -10 10633.6 565.865 14

-6 4 -10 10953.0 627.046 2

-6 -4 -10 12006.7 637.624 2

-6 -4 -10 12536.1 559.494 10

-6 4 -10 13297.8 560.745 10

-6 4 -10 13078.0 559.727 8

-6 -4 -10 12751.2 558.735 8

-6 4 -10 11405.8 588.276 5

-6 -4 -10 11877.2 588.346 5

-6 -4 -10 9210.82 555.003 21

-6 4 -10 11662.2 633.208 1

-6 -4 -10 11738.0 632.844 1

-6 -4 -10 10916.4 570.247 28

-6 -4 -11 765.600 64.6036 10

-6 4 -11 720.331 65.6677 10

-6 4 -11 678.585 91.4041 5

-6 -4 -11 728.102 91.9472 5

-6 4 -11 774.223 64.7622 8

-6 -4 -11 864.979 66.5789 8

-6 4 -11 604.022 141.145 2

-6 -4 -11 606.880 140.802 2

-6 4 -11 632.700 117.399 17

-6 -4 -11 580.864 86.7133 28

-6 -4 -12 1021.20 81.0410 10

-6 4 -12 1080.37 81.7433 10

-6 4 -12 1017.99 108.524 5

-6 -4 -12 996.755 108.712 5

-6 4 -12 940.120 164.435 2

-6 -4 -12 591.670 152.361 2

-6 4 -12 1156.14 81.3093 8

-6 -4 -12 1028.86 79.9228 8

-6 4 -12 1107.44 142.165 17

-6 -4 -12 985.899 104.464 28

-6 4 -13 96.3990 112.458 17

-6 4 -13 46.6333 43.7084 10

-6 -4 -13 88.4008 45.7212 10

-6 -4 -13 3.15441 56.9709 15

-6 -4 -13 16.4077 39.9625 8

-6 4 -13 2.32617 41.2271 8

-6 4 -13-22.2658 121.033 2

-6 -4 -13-11.1842 117.866 2

-6 4 -13 24.5426 57.1121 5

-6 -4 -13 24.7412 57.5741 5

-6 -4 -13 28.7002 72.4040 28

-6 4 -14 2342.64 198.106 17

-6 -4 -14 2253.20 236.780 2

-6 4 -14 1891.37 219.627 2

-6 4 -14 2493.88 138.686 10

-6 -4 -14 2392.40 137.437 10

-6 4 -14 2197.94 172.990 5

-6 -4 -14 2030.32 171.458 5

-6 -4 -14 1990.46 142.992 15

-6 -4 -14 2044.83 161.142 28

-6 4 -14 2357.29 136.455 8

-6 -4 -14 2434.10 138.012 8

-6 -4 -15 245.892 92.0058 28

-6 4 -15 289.247 53.9605 8

-6 -4 -15 356.091 53.6820 8

-6 4 -15 370.114 124.259 17

-6 -4 -15 366.044 63.9178 15

-6 -4 -15 329.078 53.4097 10

-6 4 -15 383.415 56.3119 10

-6 4 -15 229.756 54.9548 24

-6 4 -16 284.225 119.309 17

-6 -4 -16 412.994 93.0455 28

-6 4 -16 190.442 52.1350 24

-6 4 -16 416.818 56.0590 8

-6 -4 -16 336.902 52.9712 8

-6 4 -17 345.262 114.610 17

-6 -4 -17 340.474 93.4622 28

-6 4 -17 405.488 53.6534 8

-6 -4 -17 342.254 53.2061 8

-6 4 -18 138.150 42.8857 8

-6 -4 -18 111.570 39.4461 8

-6 4 -19 348.947 45.5050 8

-6 4 -20 65.1057 28.8982 8

6 -6 -21 199.637 68.3866 23

6 -6 -20 638.630 101.930 23

6 6 -19 1293.47 93.9489 22

6 -6 -19 1227.70 140.210 23

6 6 -18 1377.31 97.2642 22

6 -6 -18 1394.31 153.323 23

6 6 -18 1291.29 91.1792 8

6 6 -17 4969.55 260.105 22

6 -6 -17 5166.89 310.768 23

6 6 -17 4975.06 255.414 8

6 -6 -17 4799.42 282.141 26

6 6 -16 2639.51 160.998 22

6 6 -16 2622.81 155.848 8

6 -6 -16 2682.60 155.795 8

6 -6 -16 3042.20 172.928 7

6 6 -16 2861.25 175.079 7

6 -6 -16 3030.23 223.064 23

6 6 -16 2945.32 162.509 24

6 -6 -16 2847.07 188.304 26

6 6 -15 920.908 82.4203 24

6 -6 -15 803.568 106.861 26

6 6 -15 1017.64 106.729 17

6 -6 -15 1056.36 77.7890 8

6 6 -15 1050.75 78.6936 8

6 -6 -15 1055.64 93.2693 7

6 6 -15 913.207 90.4768 7

6 6 -14 4376.39 270.427 17

6 6 -14 4793.66 256.077 24

6 -6 -14 4878.41 266.419 9

6 6 -14 4595.57 266.349 9

6 -6 -14 5231.55 280.806 27

6 6 -14 5259.62 267.829 7

6 -6 -14 5282.52 265.761 7

6 -6 -14 4568.86 274.204 26

6 6 -14 5081.73 252.275 8

6 -6 -14 4944.29 251.029 8

6 6 -13 7718.56 416.080 17

6 -6 -13 8205.14 407.880 18

6 6 -13 8194.41 402.832 24

6 -6 -13 8971.70 413.592 9

6 6 -13 8041.96 411.742 9

6 -6 -13 9213.54 424.853 27

6 6 -13 8024.96 408.925 7

6 -6 -13 8419.68 408.259 7

6 -6 -13 7461.51 394.667 8

6 6 -13 8240.82 397.379 8

6 -6 -13 8221.68 418.997 26

6 -6 -13 7728.40 411.434 20

6 6 -12 4996.37 274.895 17

6 -6 -12 5346.07 267.338 18

6 6 -12 4791.46 264.797 7

6 6 -12 4981.53 262.050 24

6 -6 -12 5150.18 267.798 9

6 6 -12 5150.06 269.357 9

6 -6 -12 5743.09 279.010 27

6 6 -12 5073.89 255.205 8

6 -6 -12 4627.96 252.633 8

6 -6 -12 5240.28 269.525 20

6 -6 -12 5492.69 278.905 26

6 -6 -12 4913.92 299.760 25

6 6 -11 22709.8 1087.48 17

6 -6 -11 23755.7 1083.38 18

6 6 -11 22853.1 1081.39 7

6 -6 -11 22395.1 1072.08 8

6 -6 -11 23325.6 1089.94 27

6 6 -11 22435.7 1082.68 9

6 6 -11 21827.2 1122.58 4

6 -6 -11 22607.1 1120.92 4

6 6 -11 24519.5 1080.93 24

6 -6 -11 23751.3 1082.97 20

6 -6 -11 22483.3 1111.61 25

6 -6 -11 22458.4 1089.44 26

6 6 -10 14555.6 712.714 17

6 -6 -10 15138.0 710.200 18

6 6 -10 12490.4 720.476 13

6 6 -10 16751.5 704.970 6

6 6 -10 14090.3 743.396 4

6 -6 -10 15027.6 744.135 4

6 -6 -10 15396.5 715.997 27

6 6 -10 15038.1 706.894 24

6 -6 -10 14634.1 707.556 20

6 -6 -10 13801.5 737.391 25

6 6 -9 33427.9 1806.59 13

6 -6 -9 35616.7 1810.98 11

6 -6 -9 40605.0 1787.36 6

6 6 -9 38391.3 1825.61 4

6 -6 -9 39398.1 1824.74 4

6 -6 -9 40264.8 1793.79 20

6 -6 -9 41587.6 1797.31 18

6 6 -9 37018.3 1794.47 17

6 -6 -9 38037.7 1822.75 25

6 -6 -8 1651.12 82.9847 6

6 6 -8 1338.23 114.376 4

6 -6 -8 1379.33 114.480 4

6 6 -8 1294.14 103.914 13

6 -6 -8 1146.71 99.6636 11

6 -6 -8 1289.46 91.6677 18

6 6 -8 1221.85 90.0595 17

6 -6 -7 6797.39 315.887 18

6 -6 -7 6336.17 329.486 11

6 6 -7 5411.03 328.578 13

6 -6 -7 6536.29 316.247 21

6 6 -7 6327.91 334.559 4

6 -6 -7 6299.50 332.802 4

6 6 -6 19279.7 1010.40 13

6 -6 -6 20342.7 1020.33 12

6 -6 -6 21760.4 1009.13 11

6 6 -6 20836.8 1006.35 4

6 -6 -6 21210.8 1006.04 4

6 -6 -6 22482.4 992.568 21

6 -6 -5 3403.40 185.249 18

6 6 -5 3379.95 209.555 13

6 -6 -5 3644.80 225.193 12

6 6 -5 3974.25 200.650 4

6 -6 -5 3811.32 198.787 4

6 -6 -5 3496.78 185.462 21

6 6 -4 11929.0 575.347 4

6 -6 -4 12699.3 576.365 4

6 -6 -4 11731.6 609.327 12

6 6 -4 11735.0 594.698 13

6 -6 -4 11815.9 565.150 21

6 -6 -4 11808.8 565.627 18

6 -6 -3 8380.19 388.233 18

6 6 -3 7729.64 394.506 4

6 -6 -3 8096.78 395.504 4

6 -6 -3 7933.01 443.845 12

6 6 -2 14109.4 688.377 4

6 -6 -2 15231.1 690.477 4

6 -6 -2 13411.5 683.583 3

6 6 -2 15362.4 688.095 3

6 -6 -2 15978.2 756.763 12

6 6 -1 879.589 68.1599 3

6 -6 -1 860.976 61.9417 3

-6 6 0 49951.5 2483.84 1

-6 -6 0 51534.1 2482.81 1

6 -6 0 53433.6 2481.08 3

-6 6 -1 22301.1 1044.84 1

-6 -6 -1 23098.4 1043.88 1

6 6 1 23999.9 1050.88 3

-6 -6 -2 275.671 53.9744 2

-6 6 -2 152.907 44.1584 2

-6 6 -2 293.057 55.9920 1

-6 -6 -2 219.982 56.9748 1

-6 6 -2 368.110 40.4207 16

-6 6 -3 35465.9 1687.99 2

-6 -6 -3 36114.4 1688.48 2

-6 6 -3 34226.4 1690.52 1

-6 -6 -3 37217.8 1691.74 1

-6 6 -3 36921.4 1672.68 16

-6 6 -4 10630.0 544.599 1

-6 -6 -4 11730.1 546.019 1

-6 6 -4 10531.6 539.549 2

-6 -6 -4 10966.6 541.607 2

-6 6 -4 10751.9 521.350 16

6 -6 4 11036.4 539.480 11

-6 6 -5 10721.8 547.574 1

-6 -6 -5 11534.9 549.069 1

6 -6 5 11614.7 538.825 11

-6 6 -5 10359.4 523.213 16

-6 6 -5 10497.7 542.170 2

-6 -6 -5 10731.4 543.245 2

6 -6 6 7366.96 386.438 11

-6 6 -6 7945.82 376.405 5

-6 -6 -6 7199.55 373.572 5

-6 -6 -6 7934.22 376.235 21

-6 6 -6 7192.00 397.429 2

-6 -6 -6 7761.98 400.723 2

-6 6 -6 6927.69 399.300 1

-6 -6 -6 7525.38 402.101 1

6 -6 7 18004.9 931.238 11

-6 -6 -7 19431.5 949.614 1

-6 6 -7 18430.2 947.056 1

-6 -6 -7 20269.3 911.526 26

-6 -6 -7 19469.4 912.182 21

-6 -6 -7 19626.5 948.630 2

-6 6 -7 18191.3 943.829 2

-6 6 -7 19508.9 918.643 5

-6 -6 -7 19394.9 918.327 5

-6 6 -8 1330.20 144.623 1

-6 -6 -8 1490.17 105.259 21

-6 -6 -8 1383.16 104.243 26

-6 6 -8 1691.86 114.116 5

-6 -6 -8 1733.76 113.529 5

-6 -6 -8 1617.03 148.472 2

-6 6 -8 1405.33 144.647 2

-6 6 -9 7180.48 446.458 2

-6 -6 -9 7659.72 449.480 2

-6 -6 -9 8419.75 387.866 10

-6 -6 -9 7476.70 398.892 21

-6 -6 -9 7765.54 400.883 26

-6 -6 -9 8224.41 386.268 8

-6 6 -9 8654.92 416.227 5

-6 -6 -9 7111.48 408.616 5

-6 6 -9 8811.07 419.974 14

-6 -6 -10 11836.9 625.162 2

-6 6 -10 10322.1 613.830 2

-6 6 -10 12301.5 550.619 10

-6 -6 -10 11584.5 549.409 10

-6 -6 -10 10280.4 558.530 21

-6 -6 -10 10645.5 562.271 26

-6 6 -10 12411.0 549.777 8

-6 -6 -10 11717.1 548.730 8

-6 -6 -10 10824.8 564.514 15

-6 6 -10 12705.5 574.826 14

-6 6 -10 11172.6 578.302 5

-6 -6 -10 10763.8 576.544 5

-6 -6 -11 15171.7 859.169 2

-6 6 -11 15686.6 863.416 2

-6 6 -11 17971.8 789.171 10

-6 -6 -11 16512.7 786.689 10

-6 6 -11 17907.7 787.487 8

-6 -6 -11 17805.6 787.814 8

-6 -6 -11 15996.3 802.602 15

-6 -6 -11 16510.9 803.844 28

-6 6 -11 16283.5 819.235 5

-6 -6 -11 15689.9 817.863 5

-6 -6 -11 14497.1 794.375 21

-6 -6 -12 1688.56 132.448 21

-6 6 -12 2055.94 201.055 2

-6 -6 -12 1797.48 198.842 2

-6 6 -12 2245.27 124.965 10

-6 -6 -12 2283.03 124.149 10

-6 -6 -12 2183.40 123.040 8

-6 6 -12 2347.85 124.480 8

-6 -6 -12 2017.17 143.944 15

-6 6 -12 1832.59 181.919 17

-6 -6 -12 1957.29 145.768 28

-6 6 -12 1805.05 149.732 5

-6 -6 -12 1943.78 151.743 5

-6 6 -13 167.440 115.747 2

-6 -6 -13 62.9463 37.2303 10

-6 6 -13 79.3160 37.1934 10

-6 6 -13 145.745 118.016 17

-6 -6 -13 106.795 60.9412 15

-6 -6 -13 57.5786 54.9052 5

-6 6 -13 18.9504 37.4128 8

-6 -6 -13 56.7225 37.1475 8

-6 -6 -13 83.0815 71.0650 28

-6 6 -14 3088.23 166.805 10

-6 -6 -14 2972.06 165.400 10

-6 6 -14 2832.69 176.556 24

-6 6 -14 3540.36 232.420 17

-6 6 -14 3390.25 168.500 8

-6 -6 -14 2975.76 165.002 8

-6 -6 -14 3053.15 184.763 15

-6 -6 -14 2527.97 184.937 28

-6 6 -15 860.650 83.6736 24

-6 6 -15 949.394 138.469 17

-6 6 -15 939.783 71.7853 8

-6 -6 -15 895.557 70.2446 8

-6 -6 -15 889.976 89.3647 15

-6 -6 -15 829.780 101.236 28

-6 6 -16 844.942 65.2016 8

-6 6 -16 722.841 71.3352 24

-6 6 -16 948.421 130.803 17

-6 6 -17 183.396 40.9039 8

-6 6 -17 145.755 45.8611 24

-6 6 -17 269.423 106.392 17

-6 6 -18 77.6230 33.0027 8

-6 6 -18 156.212 90.4053 17

6 -8 -20 75.7401 77.3137 28

6 -8 -19 1127.34 122.299 28

6 8 -19 1126.59 81.5290 22

6 -8 -19 923.107 103.800 23

6 8 -18 274.735 55.4360 22

6 -8 -18 188.781 90.0627 23

6 -8 -17 234.142 99.0095 23

6 8 -17 136.672 49.9986 22

6 -8 -17 190.159 85.5620 26

6 -8 -16 86.0773 102.429 23

6 -8 -16 119.951 81.2651 26

6 8 -15 6057.91 305.092 24

6 -8 -15 5703.94 323.785 26

6 -8 -14 6680.29 351.580 20

6 8 -14 6086.23 342.131 17

6 8 -14 6964.68 339.540 24

6 -8 -14 6898.73 358.929 26

6 -8 -13 1000.12 97.7702 20

6 8 -13 925.222 93.4227 17

6 -8 -13 1034.28 95.3517 18

6 8 -13 992.726 84.2490 24

6 -8 -13 873.022 105.217 26

6 -8 -12 1334.23 105.000 20

6 -8 -12 1227.14 136.115 25

6 -8 -12 1188.17 115.471 26

6 8 -12 1117.50 100.376 17

6 -8 -12 1255.74 102.907 18

6 -8 -11 27105.2 1238.43 20

6 8 -11 24149.3 1233.97 17

6 -8 -11 27019.6 1240.24 18

6 -8 -11 27115.9 1248.69 26

6 -8 -11 27000.4 1270.75 25

6 8 -10 5750.95 283.745 24

6 -8 -10 5844.01 291.248 18

6 -8 -10 5555.29 287.551 20

6 -8 -10 5798.92 324.188 25

6 8 -9 14658.5 684.005 24

6 -8 -9 13016.2 705.945 11

6 8 -9 13121.5 711.901 13

6 -8 -9 15095.1 690.757 18

6 -8 -9 14816.8 692.861 21

6 -8 -9 14381.2 722.287 25

6 8 -8 1505.92 101.372 24

6 -8 -8 1426.83 121.405 11

6 8 -8 1676.34 131.272 13

6 -8 -8 1807.58 116.361 21

6 -8 -8 1416.29 147.057 25

6 -8 -7 20981.7 1012.75 18

6 -8 -7 21391.1 1037.57 11

6 8 -7 19190.3 1039.51 13

6 8 -7 21899.6 1009.50 24

6 -8 -7 23051.5 1016.51 21

6 -8 -6 6350.29 334.503 18

6 8 -6 6580.49 366.021 13

6 8 -6 6898.80 329.762 24

6 -8 -6 6758.31 334.480 21

6 -8 -5 18237.3 848.129 18

6 8 -5 17934.5 883.775 13

6 -8 -5 17816.0 880.785 12

6 8 -5 18024.6 843.212 24

6 -8 -5 17672.3 847.164 21

6 -8 -4 4262.35 206.773 21

6 -8 -4 3884.71 204.713 18

6 -8 -4 3552.04 240.547 12

6 8 -4 3569.57 237.144 13

6 -8 -3 8549.53 383.155 21

6 -8 -3 7768.88 382.399 18

6 8 -3 7375.13 424.033 13

6 -8 -2 3382.75 175.192 21

6 -8 -2 3252.36 242.669 12

6 -8 -2 3331.60 176.016 18

6 8 -1 8766.59 430.538 13

6 -8 -1 7627.25 370.845 21

6 -8 -1 7526.10 373.472 18

6 -8 -1 7906.43 457.365 12

-6 -8 0 90.2393 39.6559 15

-6 -8 -1 15852.8 750.528 15

-6 -8 -2 11912.3 568.764 15

-6 8 -2 11773.6 563.105 16

-6 8 -3 4278.98 221.879 16

-6 -8 -3 4340.27 228.742 15

-6 -8 -4 104.021 49.8065 15

-6 8 -4 163.441 47.6256 16

6 -8 4 158.756 58.7819 11

6 -8 5 12533.9 653.632 11

-6 -8 -5 12797.6 641.428 21

-6 -8 -5 13473.1 640.903 15

-6 8 -5 12483.7 636.058 16

-6 -8 -5 14505.0 634.355 26

6 -8 6 11576.7 623.249 11

-6 -8 -6 12509.5 610.381 21

-6 -8 -6 13181.5 611.929 15

-6 8 -6 11551.7 609.743 16

-6 -8 -6 13524.5 605.522 26

6 -8 7 10021.0 545.097 11

-6 -8 -7 11456.1 525.541 26

-6 -8 -7 10690.3 530.013 21

-6 -8 -7 10487.7 529.798 15

-6 8 -8 335.821 106.253 17

-6 -8 -8 468.566 68.7360 26

-6 -8 -8 471.235 76.1846 21

-6 -8 -8 377.739 73.8576 15

-6 8 -9 8383.79 441.280 17

-6 -8 -9 8407.96 410.169 26

-6 -8 -9 7893.29 412.021 21

-6 -8 -9 7783.73 415.230 15

-6 8 -10 1908.87 182.764 17

-6 -8 -10 2146.32 144.350 26

-6 8 -10 2307.18 157.814 14

-6 -8 -10 2043.18 145.574 21

-6 8 -11 8663.73 465.860 17

-6 -8 -11 8294.45 430.923 21

-6 -8 -11 8585.94 436.074 15

-6 -8 -12 49.9155 68.8602 15

-6 8 -12 73.1122 105.945 17

-6 -8 -12 89.1778 67.6526 21

-6 8 -13 835.594 140.015 17

-6 -8 -13 1030.34 90.0521 18

-6 -8 -13 1073.03 101.958 15

-6 -8 -13 779.228 90.4121 28

-6 8 -14 1573.79 114.628 24

-6 8 -14 1916.58 162.857 17

-6 -8 -14 1506.15 111.135 18

-6 -8 -14 1554.71 120.515 15

-6 -8 -14 1436.20 116.738 28

-6 -8 -15 1532.21 111.820 15

-6 -8 -15 1294.80 108.317 28

-6 8 -15 1420.52 105.146 24

-6 8 -16 14.1988 46.6280 24

-6 8 -17 34.7825 41.6346 24

6 -10 -19 94.9067 59.3650 28

6 10 -19 165.453 38.4363 22

6 -10 -18 305.326 82.2340 28

6 -10 -18 238.342 60.2127 23

6 10 -17 15.8436 44.7957 22

6 -10 -17 25.5849 71.6059 23

6 -10 -16 1408.51 125.598 26

6 10 -16 1490.50 100.024 22

6 -10 -16 1566.48 132.541 23

6 -10 -15 7.48169 79.5730 26

6 10 -15 47.2982 47.0509 22

6 -10 -15-4.65731 87.9555 23

6 -10 -14 5589.00 333.135 26

6 10 -14 6605.13 315.705 22

6 10 -13 289.575 61.4615 24

6 -10 -13 300.061 73.0892 20

6 -10 -12 1923.51 134.616 20

6 -10 -12 1878.58 163.736 25

6 10 -12 1933.68 127.833 24

6 -10 -12 1814.35 134.885 18

6 -10 -11 2209.33 144.109 20

6 -10 -11 2101.23 159.162 26

6 -10 -11 2107.58 175.547 25

6 10 -11 2269.01 141.944 24

6 -10 -11 2243.85 150.581 18

6 -10 -10 14108.7 698.734 20

6 -10 -10 14464.7 711.021 26

6 -10 -10 14476.9 732.744 25

6 10 -10 14800.1 696.280 24

6 -10 -9 601.632 87.2327 21

6 -10 -9 694.680 81.4593 20

6 10 -9 642.346 101.003 13

6 -10 -9 490.257 117.123 25

6 10 -9 735.338 71.8262 24

6 -10 -8 7484.32 367.578 21

6 -10 -8 6951.68 402.007 25

6 10 -8 7017.09 403.024 13

6 -10 -8 6453.30 386.881 11

6 -10 -8 7026.25 364.321 18

6 10 -8 7349.19 359.583 24

6 -10 -7 8091.16 411.984 21

6 10 -7 7713.05 449.748 13

6 -10 -7 8113.02 441.113 11

6 -10 -7 8272.74 412.429 18

6 10 -7 8413.36 406.121 24

6 -10 -6 15862.5 756.251 21

6 10 -6 14109.9 796.224 13

6 -10 -6 15661.1 786.288 11

6 -10 -6 16134.9 757.077 18

6 10 -6 16105.8 752.118 24

6 -10 -5 5224.63 263.733 21

6 -10 -5 5040.24 263.800 18

6 10 -5 4450.22 306.349 13

6 -10 -4 6682.41 347.148 21

6 -10 -4 7045.95 349.285 18

6 10 -4 7392.88 404.404 13

6 -10 -3 8244.07 401.076 18

6 -10 -3 7958.73 397.264 21

6 -10 -2-41.7762 71.3263 12

6 -10 -2-7.03468 45.7137 18

6 -10 -2 52.3335 44.2817 21

6 -10 -1 4590.30 232.348 21

6 10 -1 5173.29 303.847 13

6 -10 0 535.291 131.469 12

6 10 0 582.829 107.343 13

-6 -10 -1 3054.05 174.955 15

6 10 1 3150.71 227.741 13

6 -10 1 2603.02 254.646 12

-6 -10 -1 3141.96 185.866 21

-6 10 -2 1350.86 95.2644 16

-6 -10 -2 1397.80 104.718 15

6 -10 2 1286.05 85.2836 20

6 10 2 1294.35 147.297 13

-6 -10 -2 1297.57 112.831 21

-6 -10 -3 1636.12 116.694 15

-6 10 -3 1396.82 104.373 16

-6 -10 -3 1667.61 120.814 21

6 10 3 1309.87 145.122 13

-6 10 -3 1483.69 98.1188 24

-6 -10 -4 1897.95 132.354 15

-6 10 -4 1815.84 123.501 16

-6 -10 -4 1754.17 133.118 21

-6 10 -4 1862.81 117.278 24

6 10 4 1962.07 176.797 13

6 -10 4 1870.50 146.336 11

-6 -10 -5 3028.79 180.781 15

-6 10 -5 2548.59 172.723 16

-6 -10 -5 3146.98 166.999 26

-6 10 -5 2951.57 165.019 24

6 -10 5 2478.09 188.261 11

6 10 5 2714.02 212.386 13

-6 10 -6 16526.3 887.730 16

-6 -10 -6 19118.8 893.158 15

-6 -10 -6 20677.5 882.035 26

6 10 6 19264.2 934.744 13

6 -10 6 17063.8 907.058 11

-6 10 -6 18069.0 878.825 24

6 -10 7 941.046 111.077 11

6 10 7 1006.42 122.157 13

-6 -10 -7 896.010 104.067 21

-6 -10 -7 1131.58 107.252 15

-6 -10 -7 1121.48 95.4675 26

-6 10 -7 1028.61 91.3826 24

-6 10 -7 957.050 130.164 17

-6 -10 -8 432.282 85.4689 21

-6 -10 -8 424.551 82.5182 15

-6 -10 -8 455.571 74.3856 26

-6 10 -8 439.873 67.4553 24

-6 10 -8 344.901 109.523 17

-6 -10 -9 623.473 93.4957 21

-6 -10 -9 713.086 98.2268 15

-6 -10 -9 563.118 83.9962 26

-6 10 -9 509.026 117.484 17

-6 10 -9 626.137 74.8713 24

-6 10 -10 3837.46 219.083 24

-6 -10 -10 3980.51 233.992 15

-6 -10 -10 3903.46 229.247 21

-6 10 -10 3704.52 250.520 17

-6 -10 -11 211.349 76.1074 21

-6 10 -11 215.613 60.2569 24

-6 10 -11 221.456 100.879 17

-6 -10 -12 83.5745 69.3803 21

-6 10 -12 78.9504 96.6172 17

-6 -10 -12 51.9927 60.9867 18

-6 10 -12 49.1046 55.2252 24

-6 -10 -13 83.4022 57.5086 18

-6 10 -13 192.937 88.9027 17

-6 -10 -13 179.503 69.3878 15

-6 10 -13 115.508 56.4531 24

-6 10 -14 981.211 87.2538 24

-6 10 -14 1132.72 118.125 17

-6 10 -15 154.636 81.4732 17

-6 10 -16 273.878 74.7142 17

-6 10 -17-14.3951 34.0817 24

6 12 -17 58.6243 34.5226 22

6 12 -16 31.0766 39.0237 22

6 -12 -15 517.792 74.7886 23

6 12 -15 642.196 59.5155 22

6 -12 -14-61.9181 77.6317 26

6 -12 -14 48.4284 76.5759 23

6 12 -14 17.3604 38.7459 22

6 -12 -13 145.218 68.8276 20

6 -12 -13 217.058 84.3187 26

6 12 -13 152.828 44.9627 22

6 -12 -12 86.2755 66.7112 20

6 -12 -12 232.787 85.8120 26

6 12 -12 65.2418 43.6521 22

6 -12 -11 2140.43 142.416 20

6 12 -11 1959.23 122.163 22

6 -12 -11 2186.65 156.618 26

6 -12 -11 1991.55 158.822 25

6 -12 -10 90.7263 91.9700 25

6 -12 -10 217.169 66.8377 20

6 12 -10 128.583 41.8866 22

6 -12 -10 105.079 82.2138 26

-6 12 10 169.795 45.5540 16

-6 12 9 3899.44 207.014 16

6 -12 -9 3898.69 220.993 20

6 -12 -9 3859.34 253.202 25

6 -12 -9 3887.05 233.001 26

6 12 -9 4022.08 205.187 22

6 -12 -9 3682.38 220.975 18

6 12 -8 1901.58 180.870 13

6 12 -8 2204.34 125.979 22

-6 12 8 2173.96 131.267 16

6 -12 -8 2145.25 154.812 26

6 -12 -8 1996.11 179.566 25

6 -12 -8 2218.12 148.147 18

6 12 -7 119.375 82.8736 13

6 -12 -7 199.048 71.8763 11

6 -12 -7 67.6305 112.969 25

6 -12 -7 194.386 65.0098 20

6 -12 -7 159.868 68.6178 21

6 -12 -7 102.577 68.0211 18

6 12 -7 165.491 40.4856 22

-6 12 7 173.838 51.1114 16

6 -12 -6 882.953 112.521 11

6 -12 -6 888.883 91.8301 18

6 12 -6 842.390 128.344 13

6 -12 -6 904.979 89.3321 21

6 -12 -5 9092.92 454.447 18

6 12 -5 9445.35 514.779 13

6 -12 -5 8832.62 490.672 11

6 -12 -5 8997.95 449.631 21

-6 12 4 10020.2 459.571 16

-6 -12 4 8406.26 459.483 21

6 -12 -4 8935.35 456.951 21

6 12 -4 9982.63 529.967 13

6 -12 -4 9494.10 504.136 11

6 -12 -4 9579.09 464.898 18

6 -12 -3 117.031 50.1068 21

-6 12 3 105.184 53.4878 16

-6 -12 3 76.6886 60.7772 21

6 -12 -2 191.715 50.7058 21

-6 12 2 281.689 58.7891 16

-6 -12 2 157.927 70.9837 21

6 12 -2 240.967 99.1743 13

-6 12 1 2974.53 168.529 16

-6 -12 1 2626.36 174.833 21

6 12 -1 2681.01 222.966 13

6 -12 0 11385.5 555.465 20

-6 -12 0 11132.9 569.975 21

6 -12 1 3113.85 176.392 20

-6 -12 -1 3396.29 199.274 21

6 12 1 3371.84 247.022 13

6 -12 1 3237.84 222.626 11

6 12 2-8.71185 80.1397 13

6 -12 2-174.972 72.5572 11

-6 -12 -2-14.5449 74.2567 21

-6 12 -2 21.6991 60.7087 16

6 12 3 42.3630 84.6665 13

6 -12 3-6.65682 67.5356 11

-6 12 -3 79.8380 64.5094 16

-6 -12 -3-14.7068 73.2970 21

-6 12 -4 4354.00 237.348 16

-6 -12 -4 3838.55 237.533 21

6 12 4 4390.25 282.496 13

6 -12 4 3804.30 258.265 11

-6 12 -5 2489.12 146.877 24

6 12 5 2405.50 193.510 13

6 -12 5 2298.72 180.918 11

-6 12 -5 2138.02 172.612 17

-6 -12 -5 2339.67 166.850 21

-6 -12 -5 2611.18 168.879 15

-6 12 -5 2433.40 167.652 16

-6 -12 -6 723.005 77.3401 26

-6 -12 -6 693.688 94.6922 21

6 -12 6 505.645 93.0461 11

-6 -12 -6 724.782 96.5747 15

-6 12 -6 565.472 107.013 17

-6 12 -6 666.123 71.8880 24

-6 -12 -7 98.6262 75.4849 15

-6 12 -7-13.7310 82.7891 17

-6 12 -7 30.0504 52.8265 24

-6 -12 -8 984.599 109.314 21

-6 12 -8 1113.40 89.1898 24

-6 12 -8 824.363 116.584 17

-6 -12 -8 999.706 102.667 18

-6 -12 -9 1052.46 108.126 21

-6 12 -9 993.520 89.0835 24

-6 -12 -9 1116.61 104.412 18

-6 12 -9 869.381 114.588 17

-6 12 -10 78.8957 52.1544 24

-6 -12 -10 10.4953 67.3219 21

-6 12 -10 57.8475 78.5079 17

-6 12 -11 27.8906 50.4320 24

-6 -12 -11 138.462 69.3749 21

-6 -12 -11 43.5930 58.6088 18

-6 12 -11 61.4711 73.4230 17

-6 12 -12 158.737 52.3710 24

-6 -12 -12 187.909 65.9579 21

-6 -12 -12 100.801 57.9660 18

-6 -12 -13 55.7798 51.4840 18

-6 12 -13 67.8893 49.4769 24

-6 12 -14-8.05668 45.8231 24

-6 12 -15 53.8085 41.8385 24

6 14 -13 218.400 40.5234 22

6 -14 -13 220.713 69.1457 26

6 14 -12 54.9608 36.2498 22

6 -14 -12-2.89172 61.2463 20

6 -14 -12 16.2513 65.7474 26

6 14 -11 107.772 38.3356 22

6 -14 -11 193.992 64.7988 20

6 -14 -11 85.1348 68.6349 26

6 14 -10 40.5815 37.6409 22

6 -14 -10 93.2825 69.5127 26

6 -14 -10 46.8280 60.7303 20

-6 14 9 233.784 48.7973 16

6 -14 -9 311.945 77.1992 26

6 -14 -9 272.776 82.6186 25

-6 14 8 889.077 76.7091 16

6 -14 -8 823.300 111.127 25

6 -14 -8 971.905 100.372 26

6 -14 -8 807.478 87.3540 20

-6 14 7 121.109 50.0599 16

6 -14 -7 80.5929 90.5030 25

6 -14 -7 64.3648 71.6760 26

6 -14 -7 65.0825 57.2692 20

-6 14 6 2.60009 51.2275 16

6 -14 -6-20.7745 96.8037 25

6 -14 -6 26.4601 57.0821 20

6 -14 -6 3.39815 70.2097 26

6 -14 -5 2327.04 151.959 20

6 -14 -5 2283.43 159.120 26

-6 14 5 2698.19 151.253 16

6 -14 -4 2218.14 144.443 20

6 -14 -4 2265.27 151.754 26

-6 14 4 2401.45 147.210 16

-6 -14 3 551.058 75.1344 21

6 -14 -3 556.006 72.4500 20

-6 14 2 173.721 62.9767 16

-6 -14 2 63.6375 59.3220 21

6 -14 -2 173.767 57.3940 20

-6 -14 1 1012.47 98.8910 21

6 -14 -1 1090.78 90.1150 20

-6 -14 0 4966.41 271.679 21

6 -14 0 5015.29 261.690 20

-6 14 -1 27.8326 66.9000 16

-6 -14 -1 95.2397 61.3138 18

-6 -14 -1 96.1122 67.5363 21

-6 14 -2 85.3138 70.8728 16

-6 -14 -2 49.0750 68.0921 21

-6 -14 -2 19.8965 59.7266 18

-6 -14 -3 1602.00 126.512 21

-6 -14 -3 1413.64 118.967 18

-6 14 -4 1638.78 130.536 16

-6 -14 -4 1514.39 122.194 21

-6 -14 -5 219.480 68.5339 18

-6 -14 -5 236.280 75.7208 21

-6 -14 -6 87.5415 66.1240 18

-6 -14 -6 99.7947 71.9842 21

-6 -14 -7-82.8478 69.0411 21

-6 -14 -7 78.5444 66.8080 18

-6 14 -7 27.1769 45.0992 24

-6 -14 -8 404.704 71.9212 18

-6 14 -8 327.960 56.0889 24

-6 -14 -9 144.059 60.6019 18

-6 14 -9 96.4846 48.0302 24

-6 -14 -10 65.1902 62.4647 21

-6 -14 -10-13.1207 55.0578 18

-6 14 -10-59.4841 47.1268 24

-6 14 -11-37.7197 48.8676 24

6 -16 -9 113.294 48.5524 26

6 -16 -8 51.7737 50.2810 26

6 -16 -8 91.4294 59.1535 20

-6 16 7 102.007 49.6824 16

6 -16 -7 224.592 55.2475 26

6 -16 -7 214.832 61.2534 20

6 -16 -6 342.543 58.3115 26

6 -16 -6 244.827 63.4707 20

6 -16 -6 290.987 64.8448 25

-6 16 6 261.012 58.5289 16

6 -16 -5 471.854 68.2730 20

-6 16 5 584.055 69.5964 16

6 -16 -4-15.6353 55.3046 20

6 -16 -4-19.9996 74.0991 25

-6 16 4 84.4173 58.7646 16

6 -16 -3 970.506 85.9707 20

-6 16 3 1085.49 92.9627 16

-6 16 2 133.475 63.8871 16

6 -16 -2 113.948 57.7800 20

6 16 -1 263.216 66.7032 16

-6 16 0 288.665 74.1504 16

-6 -16 0 144.258 57.6508 21

-6 16 -1 226.798 78.3740 16

-6 -16 -1 157.221 58.1466 21

-6 -16 -2 69.5470 58.8003 21

-6 -16 -3 308.469 64.5212 21

-6 -16 -4 108.241 58.4320 21

-6 -16 -4 39.9802 54.9502 18

-6 -16 -5 92.9331 59.4809 21

-6 -16 -5 44.3903 56.5174 18

-6 -16 -6 98.6466 58.5094 21

7 1 -21 135.381 48.5204 8

7 -1 -20 50.6913 132.033 23

7 1 -20 68.3851 48.7376 8

7 -1 -20 11.4590 118.267 28

7 -1 -19 1373.47 191.338 23

7 -1 -19 1479.59 174.416 28

7 1 -19 1568.79 178.189 28

7 -1 -19 1641.56 127.886 7

7 1 -19 1555.34 107.129 8

7 -1 -18 463.632 88.6253 9

7 -1 -18 335.344 134.312 28

7 1 -18 202.985 132.812 28

7 -1 -18 345.628 156.132 23

7 -1 -18 370.180 80.3017 7

7 1 -18 288.847 59.7044 8

7 -1 -17 416.300 87.1999 9

7 1 -17 321.443 144.069 28

7 -1 -17 482.528 143.121 28

7 1 -17 322.052 57.9794 22

7 -1 -17 497.506 157.616 23

7 -1 -17 403.502 79.3720 7

7 1 -17 572.635 65.7292 8

7 -1 -16 173.595 78.0399 9

7 1 -16 161.857 134.318 28

7 -1 -16 236.293 98.0697 27

7 1 -16 133.462 48.6466 22

7 1 -16 144.341 83.6792 5

7 -1 -16 158.858 133.913 23

7 1 -16 209.387 54.5081 8

7 -1 -16 211.744 74.6793 7

7 -1 -15 136.297 67.8438 7

7 -1 -15 106.261 68.7919 9

7 1 -15 34.7488 123.362 28

7 1 -15 48.0627 84.8008 27

7 -1 -15 234.326 93.0078 27

7 1 -15 45.5812 42.8572 22

7 1 -15 77.5075 73.6573 5

7 1 -15 33.4071 145.812 23

7 -1 -15-54.3113 98.8936 4

7 1 -15 99.9570 46.3113 8

7 -1 -14 17259.7 843.039 7

7 -1 -14 18230.8 847.445 9

7 1 -14 18997.5 862.418 27

7 -1 -14 18108.3 859.679 27

7 1 -14 13033.4 866.915 28

7 1 -14 16874.4 831.347 22

7 1 -14 19940.4 889.758 5

7 -1 -14 16261.3 897.057 4

7 1 -14 14671.4 886.601 23

7 1 -14 18745.9 840.347 6

7 1 -14 19403.3 836.311 8

7 -1 -13 1640.93 114.933 7

7 -1 -13 1535.66 116.672 9

7 -1 -13 1967.15 134.728 27

7 1 -13 1779.56 134.382 27

7 1 -13 1631.38 103.957 22

7 1 -13 1597.21 143.397 5

7 -1 -13 1624.35 158.606 4

7 1 -13 1278.97 172.458 23

7 1 -13 1895.41 113.491 6

7 1 -13 1919.45 108.412 8

7 -1 -12 1924.87 126.755 7

7 -1 -12 2108.48 131.219 9

7 1 -12 2982.38 151.994 27

7 -1 -12 2854.36 153.060 27

7 1 -12 1997.18 117.017 22

7 1 -12 1836.19 147.000 5

7 -1 -12 1870.89 170.323 4

7 1 -12 1772.64 120.465 6

7 1 -12 2051.75 118.550 8

7 1 -12 1920.09 181.631 23

7 1 -11 5576.91 294.750 5

7 -1 -11 5245.60 311.612 4

7 -1 -11 5765.86 279.441 27

7 -1 -11 4779.37 265.443 7

7 1 -11 4867.83 261.676 6

7 1 -11 5685.16 260.621 8

7 -1 -11 5298.82 259.218 8

7 1 -11 4836.70 275.434 17

7 -1 -11 5125.20 268.754 9

7 1 -10 50634.1 2398.81 17

7 1 -10 58131.0 2423.61 5

7 -1 -10 51240.1 2432.62 4

7 1 -10 50490.6 2389.63 6

7 1 -9 44843.0 2110.41 17

7 1 -9 44312.2 2102.00 6

7 1 -9 50838.1 2130.81 5

7 -1 -9 44897.8 2140.73 4

7 -1 -8 249.906 72.7662 4

7 1 -8 389.782 55.7450 5

7 -1 -8 283.326 37.3993 6

7 1 -8 291.419 37.3800 6

7 -1 -7 985.807 86.6162 11

7 1 -7 750.480 46.8839 13

7 1 -7 911.279 74.3858 5

7 -1 -7 907.843 94.2540 4

7 -1 -6 283282. 13746.6 4

7 1 -6 278014. 13742.2 3

7 1 -5 41338.7 2084.44 3

7 -1 -5 43168.7 2092.50 4

7 -1 -5 44259.4 2096.44 12

7 -1 -4 129602. 5729.55 11

7 1 -4 124126. 5700.72 3

7 -1 -4 117707. 5709.54 12

7 1 -4 125023. 5701.26 12

7 1 -3 6710.13 324.943 3

7 -1 -3 6172.83 337.086 12

7 1 -3 6449.62 322.674 12

7 1 -2 126042. 6034.55 3

7 -1 -2 140107. 6054.93 12

7 1 -2 130165. 6035.54 12

7 1 -1 53928.6 2510.71 12

7 -1 0 50844.1 2393.94 11

7 -1 1 44105.7 2088.82 11

7 -1 2 26765.4 1293.85 11

7 -1 3 3750.80 239.265 11

-7 -1 -3 3552.74 225.495 1

7 -1 4 36481.5 1561.75 11

-7 -1 -4 29553.7 1546.66 1

7 -1 5 11671.8 522.330 11

-7 -1 -5 8443.71 516.742 1

-7 -1 -6 18647.9 915.832 1

-7 -1 -6 18238.2 911.380 2

-7 1 -7 1792.57 121.708 14

-7 1 -7 1745.36 125.461 17

-7 -1 -7 1995.47 168.690 2

-7 -1 -7 1774.25 176.086 1

-7 1 -8 2836.88 183.182 17

-7 -1 -8 3002.48 180.149 17

-7 -1 -8 3348.84 234.001 2

-7 -1 -8 3348.04 239.451 1

-7 -1 -9 6500.73 392.523 1

-7 1 -9 6545.98 320.042 10

-7 -1 -9 6854.38 320.534 10

-7 1 -9 6229.75 340.696 17

-7 -1 -9 5895.79 334.721 17

-7 -1 -9 6120.79 384.859 2

-7 -1 -10 13631.9 736.622 2

-7 1 -10 15527.1 664.240 10

-7 1 -10 16148.5 664.201 8

-7 1 -10 13971.7 692.831 5

-7 1 -10 11479.1 677.546 28

-7 -1 -10 11630.6 672.369 28

-7 1 -10 13334.8 684.199 17

-7 -1 -10 13259.1 680.004 17

-7 -1 -10 14505.6 749.692 1

-7 -1 -11 4492.20 315.924 2

-7 1 -11 4566.02 224.471 10

-7 1 -11 4573.88 223.493 8

-7 1 -11 4231.11 254.352 5

-7 -1 -11 3468.37 238.752 28

-7 1 -11 3892.32 250.003 28

-7 1 -11 3875.78 254.216 17

-7 -1 -11 4044.38 248.751 17

-7 -1 -11 4321.17 322.388 1

-7 1 -12 1722.54 109.993 10

-7 1 -12 1728.16 109.478 8

-7 1 -12 1632.83 141.089 5

-7 1 -12 1268.28 142.882 28

-7 -1 -12 1232.66 133.466 28

-7 1 -12 1332.80 147.519 17

-7 -1 -12 1753.98 148.280 17

-7 -1 -12 1735.06 204.562 2

-7 1 -13 584.315 70.7427 10

-7 1 -13 732.490 73.0147 8

-7 1 -13 669.950 99.0302 5

-7 -1 -13 724.941 113.510 28

-7 -1 -13 332.650 151.205 2

-7 -1 -13 846.978 127.162 17

-7 1 -13 595.032 121.334 17

-7 -1 -14 8066.13 506.995 2

-7 1 -14 8878.56 388.352 10

-7 1 -14 7658.61 429.373 5

-7 -1 -14 8319.15 424.721 17

-7 1 -14 8388.96 428.439 17

-7 1 -14 8878.34 387.362 8

-7 -1 -14 6046.71 374.518 15

-7 -1 -14 6255.79 407.430 28

-7 1 -15 669.329 73.4127 8

-7 1 -15 614.197 101.079 5

-7 -1 -15 621.598 171.826 2

-7 1 -15 791.219 130.719 17

-7 -1 -15 683.835 126.497 17

-7 1 -15 755.074 76.8302 10

-7 1 -16 803.561 79.1513 8

-7 1 -16 892.282 140.453 17

-7 -1 -16 1187.85 138.554 17

-7 1 -16 856.436 79.6539 10

-7 1 -17-12.4439 48.4111 8

-7 1 -17 72.2614 103.947 17

-7 1 -17 74.9130 48.1736 10

-7 1 -18 486.905 60.3968 8

-7 1 -19 260.561 47.2026 8

-7 1 -20 20.8283 34.8435 8

7 -3 -21 373.451 109.765 28

7 -3 -21 488.217 111.379 23

7 -3 -20 1348.94 155.204 28

7 3 -20 1436.88 96.5482 8

7 -3 -20 1380.70 96.4524 8

7 -3 -20 1543.95 166.541 23

7 -3 -19 78.8319 116.371 28

7 -3 -19 19.5025 124.398 23

7 3 -19 4.80730 47.8555 8

7 -3 -19-14.3805 46.0973 8

7 3 -18 4709.06 256.064 22

7 -3 -18 4882.34 318.099 23

7 3 -18 4901.85 270.804 7

7 -3 -18 4795.58 268.128 7

7 3 -18 5098.30 255.709 8

7 -3 -18 4822.67 254.062 8

7 -3 -18 4723.04 312.100 28

7 -3 -17 7.34409 68.3916 7

7 3 -17 3.76860 68.7292 7

7 -3 -17 3.99367 71.9302 9

7 3 -17 61.1579 76.1498 9

7 3 -17 44.4234 133.500 28

7 3 -17 69.8505 48.1212 22

7 -3 -17 68.8864 49.3349 8

7 3 -17 105.569 52.2377 8

7 3 -17 187.753 171.888 23

7 -3 -17 51.3444 134.108 23

7 3 -16 499.725 81.9412 7

7 -3 -16 538.341 83.1652 7

7 -3 -16 447.430 105.836 27

7 3 -16 328.223 142.037 28

7 -3 -16 395.186 90.7158 26

7 -3 -16 645.811 87.0521 9

7 3 -16 554.676 85.5488 9

7 3 -16 504.090 63.2663 22

7 3 -16 247.269 167.743 23

7 -3 -16 506.174 140.013 23

7 -3 -16 620.902 62.6058 8

7 3 -16 562.906 63.2347 8

7 -3 -15 3748.85 202.891 7

7 3 -15 3649.58 202.185 7

7 3 -15 2364.97 246.459 28

7 -3 -15 3418.23 220.100 27

7 -3 -15 2549.24 200.358 26

7 3 -15 3413.60 204.087 9

7 -3 -15 3529.74 205.658 9

7 3 -15 3461.08 187.060 22

7 3 -15 3215.55 251.388 4

7 -3 -15 3429.54 253.958 4

7 -3 -15 3476.74 253.502 23

7 3 -15 2331.26 267.575 23

7 3 -15 3827.47 190.909 8

7 -3 -15 3832.54 189.098 8

7 3 -14 5268.19 291.082 17

7 3 -14 4939.02 276.427 7

7 -3 -14 5235.03 276.375 7

7 3 -14 5886.96 302.679 27

7 3 -14 3175.57 306.361 28

7 -3 -14 5741.93 296.442 27

7 -3 -14 4908.12 280.033 26

7 -3 -14 5497.89 282.191 9

7 3 -14 5103.09 279.411 9

7 3 -14 5070.09 263.943 22

7 3 -14 5558.49 316.682 5

7 -3 -14 5820.23 315.780 5

7 3 -14 4596.74 326.737 4

7 -3 -14 4778.48 325.713 4

7 3 -14 5207.38 262.832 24

7 3 -14 3615.80 335.428 23

7 -3 -14 5482.85 265.628 8

7 3 -14 5551.35 267.171 8

7 3 -13-10.8720 69.7433 17

7 -3 -13-2.86429 52.9040 7

7 3 -13 58.4648 52.9317 7

7 -3 -13 110.383 61.8662 26

7 3 -13 110.328 61.3424 9

7 -3 -13 58.9213 53.4629 9

7 3 -13-73.1637 132.079 23

7 3 -13 48.2942 35.4559 24

7 3 -13 29.6266 46.8963 6

7 -3 -13 46.3297 42.9960 6

7 3 -13 141.893 68.3533 5

7 -3 -13 92.4863 62.6247 5

7 3 -13-6.33759 103.379 28

7 3 -13 103.436 73.9316 27

7 -3 -13 103.461 71.0522 27

7 -3 -13 74.6101 37.6803 8

7 3 -13 55.7096 37.9842 8

7 3 -13 47.2152 84.8479 4

7 -3 -13 77.1147 84.9510 4

7 3 -12 462.622 47.1676 8

7 -3 -12 423.546 44.8850 8

7 3 -12 129.788 92.0104 28

7 3 -12 141.628 70.9236 27

7 -3 -12 337.999 51.1163 6

7 3 -12 446.617 77.7166 5

7 -3 -12 414.626 73.0637 5

7 3 -12 243.474 69.4650 17

7 3 -12 296.897 54.7041 7

7 -3 -12 291.143 57.1891 7

7 -3 -12 256.364 71.7324 27

7 -3 -12 157.285 61.8551 26

7 3 -12 280.709 43.6170 24

7 3 -12 95.2290 123.620 23

7 3 -12 212.778 55.7173 9

7 -3 -12 209.449 56.2583 9

7 3 -12 375.958 53.2480 6

7 3 -12 215.299 88.0303 4

7 -3 -12 302.644 93.9329 4

7 3 -11 21332.7 1012.77 17

7 -3 -11 21552.6 1006.77 7

7 3 -11 21201.4 1006.69 7

7 3 -11 23472.9 1000.99 8

7 -3 -11 22456.8 999.428 8

7 -3 -11 21988.3 1003.32 6

7 3 -11 21900.7 1003.78 6

7 -3 -11 21369.2 1052.83 4

7 -3 -11 22861.7 1036.29 5

7 3 -11 24008.5 1041.31 5

7 3 -11 20576.6 1049.91 4

7 -3 -11 22340.0 1009.45 9

7 3 -11 21372.9 1009.09 9

7 -3 -11 18537.5 1010.21 20

7 3 -11 22258.1 1018.14 27

7 -3 -11 22265.2 1018.19 27

7 3 -10 776.803 78.9807 17

7 3 -10 1243.88 98.1793 5

7 -3 -10 1121.76 92.9486 5

7 -3 -10 887.602 108.281 4

7 3 -10 842.306 105.664 4

7 -3 -10 792.823 96.1994 19

7 -3 -10 690.240 73.5830 20

7 3 -10 1053.91 69.0980 6

7 -3 -10 888.410 65.9142 6

7 3 -9 43819.1 1943.65 6

7 -3 -9 41610.6 1942.59 6

7 3 -9 42961.3 1950.30 17

7 -3 -9 42250.0 1944.69 18

7 -3 -9 36397.5 1960.58 19

7 -3 -9 42866.2 1948.60 20

7 3 -9 49241.1 1974.85 5

7 -3 -9 45326.5 1970.02 5

7 3 -9 42680.3 1983.95 4

7 -3 -9 44052.5 1985.41 4

7 3 -9 35754.7 1942.92 13

7 -3 -9 47994.2 1977.48 11

7 -3 -8 2166.84 110.493 6

7 3 -8 2136.61 111.042 6

7 -3 -8 1289.51 148.166 11

7 -3 -8 1686.77 111.710 18

7 3 -8 1809.33 111.865 13

7 -3 -8 2020.24 146.577 4

7 -3 -8 2542.34 137.073 5

7 3 -8 2677.85 141.601 5

7 3 -8 2028.11 147.706 4

7 -3 -8 1878.86 114.650 20

7 3 -7 52670.3 2527.79 13

7 3 -7 56488.2 2553.11 4

7 -3 -7 59290.9 2554.33 4

7 -3 -7 52688.1 2558.36 11

7 -3 -6 109562. 5090.74 12

7 3 -6 113825. 5083.02 4

7 -3 -6 117093. 5083.90 4

7 3 -6 103996. 5063.87 13

7 -3 -6 110664. 5079.84 11

7 -3 -5 84337.3 3870.77 11

7 -3 -5 79999.9 3887.23 12

7 -3 -5 87350.3 3878.23 4

7 3 -5 82682.9 3876.71 4

7 3 -5 85442.5 3872.60 3

7 -3 -5 82296.9 3870.45 3

7 -3 -4 10105.0 495.503 11

7 3 -4 10214.1 499.090 3

7 -3 -4 10061.9 497.014 3

7 -3 -3 33420.9 1569.53 11

7 3 -3 34570.1 1577.02 3

7 -3 -3 33052.3 1574.53 3

7 3 -2 39999.2 1802.28 3

7 -3 -2 37182.2 1798.59 3

7 3 -1 67892.5 3000.13 12

7 3 -1 65528.3 3019.27 3

7 -3 -1 59758.2 3015.26 3

7 3 0 46119.3 2237.15 12

7 -3 0 52164.3 2248.61 11

7 3 1 13554.0 654.913 12

7 -3 1 13967.0 661.021 11

7 -3 2 24580.5 1161.15 11

7 -3 3 454.725 71.9724 11

-7 3 -3 446.553 93.1697 1

-7 -3 -3 500.001 94.1564 1

7 -3 4 278.709 74.6817 11

-7 -3 -4 336.142 95.3900 1

-7 3 -4 294.247 93.2793 1

7 -3 5 1045.52 128.345 11

-7 3 -5 1069.43 119.637 2

-7 -3 -5 1439.40 128.201 2

-7 3 -5 1092.62 125.689 1

-7 -3 -5 1544.70 141.276 1

-7 3 -6 76.8732 85.9748 1

-7 -3 -6 41.1591 87.1221 1

-7 -3 -6 92.4134 87.2021 2

-7 3 -6 48.6216 79.1304 2

-7 3 -6 163.310 70.0003 14

7 -3 6 89.2972 101.036 11

-7 3 -7 2084.65 135.633 14

7 -3 7 1888.46 160.330 11

-7 -3 -7 1743.29 163.006 2

-7 3 -7 1719.85 159.124 2

-7 3 -7 1847.45 169.348 1

-7 -3 -7 1688.26 167.612 1

-7 3 -8 2868.29 209.198 17

-7 3 -8 4156.44 208.354 14

-7 -3 -8 3739.67 253.873 1

-7 3 -8 3747.70 256.871 1

-7 -3 -8 3454.24 246.022 2

-7 3 -8 2972.89 237.885 2

-7 3 -9 6752.43 436.165 2

-7 -3 -9 7590.86 444.465 2

-7 3 -9 7527.45 405.124 5

-7 -3 -9 7157.96 403.221 5

-7 3 -9 7441.16 408.287 17

-7 3 -9 8044.59 379.717 8

-7 -3 -9 8056.35 379.779 8

-7 3 -9 7409.13 391.677 14

-7 -3 -9 7903.31 380.481 10

-7 3 -9 8447.41 381.506 10

-7 3 -9 7083.10 448.739 1

-7 -3 -9 7651.60 452.928 1

-7 3 -10 174.091 103.141 2

-7 3 -10 229.978 122.309 1

-7 -3 -10 240.133 129.004 1

-7 -3 -10 242.206 116.535 2

-7 3 -10 380.189 50.2267 10

-7 -3 -10 315.029 49.8684 10

-7 3 -10 300.314 96.3701 17

-7 3 -10 377.164 47.6720 8

-7 -3 -10 308.788 46.8033 8

-7 3 -10 303.867 62.7315 14

-7 -3 -10 396.257 74.7843 28

-7 3 -10 334.328 65.0794 5

-7 -3 -10 235.450 64.1453 5

-7 -3 -11 6049.70 388.292 2

-7 3 -11 5600.81 379.952 2

-7 3 -11 6203.03 299.831 10

-7 -3 -11 6291.08 300.785 10

-7 3 -11 6023.21 297.694 8

-7 -3 -11 6129.74 298.568 8

-7 3 -11 5729.52 331.865 5

-7 -3 -11 5534.82 330.274 5

-7 3 -11 5950.29 391.150 1

-7 -3 -11 5687.15 387.782 1

-7 3 -11 5772.29 336.351 17

-7 -3 -11 4794.39 309.168 28

-7 3 -12 222.568 52.0604 10

-7 -3 -12 226.044 49.7621 10

-7 3 -12 250.745 52.3741 8

-7 -3 -12 199.700 49.4007 8

-7 3 -12 130.205 68.9244 5

-7 -3 -12 156.596 66.9845 5

-7 3 -12 212.811 149.855 2

-7 -3 -12 112.631 138.627 2

-7 3 -12 230.693 110.456 17

-7 -3 -12 232.486 83.4560 28

-7 -3 -13 1259.70 92.7874 10

-7 3 -13 1379.69 94.0724 10

-7 3 -13 1287.87 91.8704 8

-7 -3 -13 1327.14 93.7654 8

-7 3 -13 1079.64 119.979 5

-7 -3 -13 1022.51 119.692 5

-7 -3 -13 1309.79 193.295 2

-7 3 -13 1004.16 178.645 2

-7 3 -13 1045.83 149.909 17

-7 -3 -13 896.165 119.511 28

-7 3 -14 146.498 110.658 17

-7 -3 -14 38.3618 48.9401 10

-7 3 -14 24.6227 50.1727 10

-7 -3 -14 6.16554 52.2610 15

-7 3 -14-48.1662 70.4927 5

-7 -3 -14 69.4019 64.9693 5

-7 3 -14-7.78866 48.0437 8

-7 -3 -14 28.7461 45.2371 8

-7 -3 -14 81.1518 84.8954 28

-7 3 -14-12.4871 130.986 2

-7 -3 -14-37.6341 135.197 2

-7 3 -15 1507.12 104.068 8

-7 -3 -15 1463.90 101.621 8

-7 -3 -15 1500.06 103.199 10

-7 3 -15 1606.40 105.806 10

-7 3 -15 1751.16 171.823 17

-7 -3 -15 1061.20 100.249 15

-7 -3 -15 1216.44 132.843 28

-7 -3 -16 173.573 50.5621 10

-7 3 -16 194.797 52.8139 10

-7 3 -16 97.1670 51.9975 8

-7 -3 -16 155.203 49.6063 8

-7 3 -16 279.375 119.597 17

-7 3 -17 425.321 57.9421 8

-7 -3 -17 443.636 58.2112 8

-7 3 -17 494.799 118.346 17

-7 3 -18-24.5280 40.8236 8

-7 -3 -18 67.1756 42.0350 8

-7 3 -19 374.026 47.9808 8

-7 -3 -19 372.128 46.2071 8

-7 3 -20 45.1945 31.9083 8

7 -5 -21 174.929 94.9035 28

7 -5 -21 51.2168 80.4466 23

7 -5 -20 368.397 116.421 28

7 -5 -20 424.539 109.858 23

7 5 -19 2226.13 132.545 8

7 -5 -19 2516.74 193.477 28

7 -5 -19 2202.13 189.060 23

7 -5 -18 331.939 128.919 23

7 -5 -18 149.477 47.4238 8

7 5 -18 151.342 48.9661 8

7 5 -18 226.576 55.0450 22

7 -5 -17 135.819 82.0928 26

7 5 -17 241.690 67.9784 7

7 -5 -17 306.846 69.6904 7

7 -5 -17 183.738 121.839 23

7 5 -17 106.401 46.1286 8

7 -5 -17 145.277 46.2693 8

7 5 -17 177.870 54.8132 22

7 -5 -16 3348.30 212.314 26

7 5 -16 3385.61 206.856 9

7 -5 -16 3528.36 206.661 9

7 5 -16 3279.44 201.574 7

7 -5 -16 3607.55 202.961 7

7 -5 -16 3802.49 258.308 23

7 5 -16 3488.96 188.302 8

7 -5 -16 3354.49 187.024 8

7 5 -16 3307.96 190.093 22

7 -5 -15 7111.43 349.722 27

7 -5 -15 6466.96 340.518 26

7 -5 -15 6746.52 334.712 9

7 5 -15 6514.47 336.406 9

7 5 -15 6288.37 331.458 7

7 -5 -15 6172.53 329.082 7

7 5 -15 6300.32 318.531 24

7 5 -15 5989.97 318.642 22

7 5 -15 6165.06 315.550 8

7 -5 -15 5868.06 313.331 8

7 5 -14 537.697 90.2210 17

7 5 -14 457.168 72.8373 7

7 -5 -14 433.369 68.8899 7

7 -5 -14 658.672 96.8180 27

7 5 -14 469.603 60.6375 24

7 -5 -14 514.661 77.0801 9

7 5 -14 495.210 78.0105 9

7 -5 -14 611.143 87.6088 26

7 5 -14 391.171 52.5846 8

7 -5 -14 490.921 55.1482 8

7 5 -13 2084.62 146.565 17

7 -5 -13 2056.53 134.809 7

7 5 -13 1955.64 134.779 7

7 5 -13 2220.76 129.135 24

7 5 -13 2103.20 140.972 9

7 -5 -13 2248.34 139.646 9

7 -5 -13 2084.63 145.328 26

7 5 -13 1704.00 174.927 4

7 -5 -13 1964.05 178.987 4

7 -5 -13 1907.92 145.321 20

7 5 -13 2073.45 122.852 8

7 -5 -13 1976.90 120.788 8

7 -5 -12 5463.86 282.058 20

7 5 -12 4840.70 322.001 4

7 -5 -12 4977.36 318.518 4

7 -5 -12 6228.69 290.400 26

7 -5 -12 6073.90 316.828 25

7 -5 -12 5626.05 280.057 9

7 5 -12 5578.28 281.011 9

7 5 -12 5401.42 284.666 17

7 5 -12 5307.43 277.458 7

7 -5 -12 5227.52 274.967 7

7 5 -12 6745.98 302.025 27

7 -5 -12 6579.80 296.894 27

7 5 -12 6048.88 274.203 24

7 5 -12 5092.66 266.020 8

7 -5 -12 4968.90 264.411 8

7 -5 -12 4768.12 268.585 6

7 5 -12 5066.73 270.243 6

7 -5 -11 1281.03 144.180 19

7 -5 -11 1821.26 125.869 20

7 5 -11 2103.29 169.278 4

7 -5 -11 1963.87 166.941 4

7 5 -11 1790.19 130.627 17

7 -5 -11 1953.93 125.982 18

7 5 -11 1965.17 123.798 7

7 -5 -11 2327.05 128.344 7

7 -5 -11 1763.97 158.307 25

7 -5 -11 1644.51 129.235 26

7 -5 -11 2111.34 128.661 9

7 5 -11 2020.18 128.247 9

7 5 -11 2517.02 124.589 6

7 -5 -11 2742.98 124.594 6

7 -5 -11 2326.48 117.346 8

7 5 -11 2337.59 118.942 8

7 5 -11 2030.25 119.880 24

7 5 -11 1805.94 137.370 27

7 -5 -11 1702.56 132.683 27

7 -5 -10 63.1760 68.1650 4

7 5 -10 65.6545 74.0897 4

7 5 -10 26.4769 33.0369 13

7 -5 -10 35.3791 54.1416 27

7 -5 -10 112.528 34.8395 6

7 5 -10 119.452 37.2291 6

7 -5 -10 181.419 86.9245 25

7 5 -10-68.0699 108.676 23

7 5 -10 31.4454 37.7574 24

7 -5 -10 52.5404 43.8506 20

7 -5 -10 36.0422 43.1247 18

7 5 -10 101.238 53.6698 17

7 5 -9 2537.53 140.159 17

7 -5 -9 2387.54 137.572 18

7 5 -9 2021.35 141.458 13

7 -5 -9 2374.07 128.995 6

7 5 -9 2658.85 131.467 6

7 5 -9 2352.13 170.295 4

7 -5 -9 2427.92 168.241 4

7 -5 -9 2032.76 163.670 11

7 5 -9 2533.19 132.580 24

7 -5 -9 1918.63 164.190 25

7 -5 -9 2314.93 134.332 20

7 -5 -8 2996.39 176.282 6

7 5 -8 3435.25 178.663 6

7 5 -8 3614.77 217.739 4

7 -5 -8 3572.76 215.446 4

7 -5 -8 4040.63 187.567 20

7 5 -8 3086.79 194.757 13

7 -5 -8 3417.66 207.581 11

7 5 -8 3849.18 188.336 17

7 -5 -8 4133.95 190.120 18

7 5 -7 6196.79 323.153 4

7 -5 -7 6233.89 322.284 4

7 -5 -7 5948.53 295.973 18

7 -5 -7 6172.26 294.736 20

7 5 -7 5385.09 308.538 13

7 -5 -7 5891.29 312.664 11

7 5 -6 4220.75 252.917 4

7 -5 -6 4400.96 252.533 4

7 -5 -6 4736.00 233.018 18

7 -5 -6 4983.86 236.596 21

7 -5 -6 4928.98 231.953 20

7 -5 -6 4136.66 269.030 12

7 -5 -6 4440.19 247.766 11

7 5 -6 4247.00 249.991 13

7 5 -5 1028.85 84.7854 4

7 -5 -5 947.158 83.6202 4

7 5 -5 749.223 79.6240 13

7 -5 -5 876.589 79.2965 11

7 -5 -5 776.945 107.708 12

7 -5 -5 632.094 62.6435 21

7 -5 -4 1511.13 106.090 11

7 -5 -4 1471.88 150.468 12

7 5 -4 1588.50 107.040 3

7 -5 -4 1448.85 102.101 3

7 -5 -4 1435.78 88.1627 18

7 -5 -4 1570.81 110.277 4

7 5 -4 1346.54 106.424 4

7 -5 -3 96163.0 4510.98 3

7 5 -3 100707. 4513.66 3

7 5 -3 95016.4 4516.66 4

7 -5 -3 99812.8 4516.07 11

7 -5 -3 97858.9 4558.50 12

7 5 -2 3998.54 215.779 3

7 -5 -2 3777.90 210.617 3

7 -5 -2 4222.29 274.471 12

7 5 -1 21282.9 938.766 3

7 -5 -1 18317.3 930.187 3

7 -5 0 3788.27 226.154 3

7 5 0 4134.02 232.965 3

7 -5 0 4426.69 225.946 11

7 -5 1 21798.6 1033.21 11

-7 5 -2 2134.86 152.637 1

-7 -5 -2 2440.68 156.728 1

7 -5 2 1948.15 134.988 11

-7 5 -3 237.674 76.0521 1

-7 -5 -3 325.878 83.4076 1

-7 5 -3 509.309 55.3258 16

7 -5 3 376.631 60.9252 11

-7 -5 -4 29143.9 1279.76 2

-7 5 -4 26384.6 1274.84 2

-7 5 -4 25972.2 1279.97 1

-7 -5 -4 29945.0 1285.24 1

7 -5 4 27187.8 1264.98 11

-7 5 -4 24748.2 1250.05 16

-7 5 -5 18654.2 950.026 1

-7 -5 -5 20911.4 955.664 1

7 -5 5 19218.3 934.941 11

-7 -5 -5 20402.8 950.379 2

-7 5 -5 18351.2 945.031 2

-7 5 -6 140.116 75.2493 2

-7 -5 -6 188.851 81.3972 2

-7 5 -6 143.793 86.1863 1

-7 -5 -6 233.382 93.3336 1

7 -5 6 151.589 65.9747 11

-7 -5 -6 90.2552 48.3367 21

-7 -5 -7 2511.75 151.810 21

-7 5 -7 2789.83 193.365 2

-7 -5 -7 2805.62 195.045 2

7 -5 7 2248.27 188.848 11

-7 5 -7 2510.38 200.025 1

-7 -5 -7 3208.02 208.599 1

-7 -5 -8 3477.48 258.400 2

-7 5 -8 3629.52 258.400 2

-7 5 -8 3934.07 225.168 5

-7 -5 -8 3836.38 224.632 5

-7 -5 -8 3925.55 211.614 21

-7 5 -8 4006.72 244.309 17

-7 5 -8 4339.16 233.441 14

-7 5 -8 4103.30 271.858 1

-7 -5 -8 4501.15 276.070 1

-7 5 -9 629.196 88.8903 14

-7 5 -9 421.940 114.783 1

-7 -5 -9 390.929 119.189 1

-7 5 -9 525.084 115.160 2

-7 -5 -9 509.879 117.809 2

-7 5 -9 633.806 53.6352 10

-7 -5 -9 614.821 55.5473 10

-7 -5 -9 320.834 60.7656 21

-7 5 -9 410.333 106.075 17

-7 5 -9 587.844 53.8464 8

-7 -5 -9 687.844 54.4231 8

-7 5 -9 527.359 74.4203 5

-7 -5 -9 509.225 68.6610 5

-7 -5 -9 394.019 68.8050 28

-7 5 -10 116.252 67.9176 14

-7 5 -10 18.0168 107.659 2

-7 -5 -10 63.1377 105.854 2

-7 -5 -10 83.5540 37.8080 10

-7 5 -10 73.3915 40.5762 10

-7 -5 -10 76.2173 49.2866 21

-7 5 -10 163.799 102.891 17

-7 5 -10 98.7347 36.1521 8

-7 -5 -10 103.731 38.1983 8

-7 -5 -10 196.768 64.9062 28

-7 5 -10 9.94745 51.5959 5

-7 -5 -10 60.3112 55.0164 5

-7 5 -11 5193.81 367.662 2

-7 -5 -11 6298.08 384.380 2

-7 -5 -11 5907.17 291.037 10

-7 5 -11 6596.47 293.285 10

-7 5 -11 6391.64 290.933 8

-7 -5 -11 6263.27 290.760 8

-7 -5 -11 4501.67 289.051 21

-7 5 -11 6160.62 343.898 17

-7 5 -11 5430.45 322.945 5

-7 -5 -11 5050.79 318.505 5

-7 -5 -11 4970.12 302.121 28

-7 5 -12 871.097 162.213 2

-7 -5 -12 722.618 162.971 2

-7 -5 -12 1081.19 79.2196 10

-7 5 -12 1052.62 80.7111 10

-7 5 -12 1172.82 81.2983 8

-7 -5 -12 1161.41 80.2023 8

-7 -5 -12 806.448 96.6108 15

-7 5 -12 1002.66 144.907 17

-7 5 -12 1007.86 110.224 5

-7 -5 -12 1091.16 110.782 5

-7 -5 -12 632.626 98.9572 28

-7 5 -13 178.498 128.781 2

-7 -5 -13 178.387 122.139 2

-7 -5 -13 301.929 52.1178 10

-7 5 -13 404.475 55.2821 10

-7 -5 -13 317.214 51.0981 8

-7 5 -13 427.840 52.8634 8

-7 -5 -13 256.662 70.0005 15

-7 5 -13 529.196 130.847 17

-7 5 -13 345.655 77.0289 5

-7 -5 -13 253.551 71.6728 5

-7 -5 -13 263.767 77.4013 28

-7 5 -14 382.915 150.048 2

-7 5 -14 627.663 62.3529 10

-7 -5 -14 519.177 59.1326 10

-7 5 -14 499.503 127.569 17

-7 -5 -14 450.621 76.9383 15

-7 5 -14 591.636 61.2923 8

-7 -5 -14 566.499 61.9036 8

-7 5 -14 420.428 84.6973 5

-7 -5 -14 370.700 79.4374 5

-7 -5 -14 481.665 92.7897 28

-7 5 -15 76.1737 43.0515 10

-7 -5 -15 37.5613 41.4558 10

-7 5 -15 8.51244 50.5678 24

-7 -5 -15 19.7390 77.7157 28

-7 5 -15 74.8002 118.194 17

-7 5 -15 70.8678 42.8057 8

-7 -5 -15 75.2962 41.7830 8

-7 -5 -15 47.7377 56.1543 15

-7 5 -16 137.181 49.8704 24

-7 5 -16 198.153 44.2191 8

-7 -5 -16 91.5448 39.2087 8

-7 5 -16 197.395 111.589 17

-7 -5 -16 134.633 76.5167 28

-7 5 -17 37.8479 98.9063 17

-7 5 -17-11.6359 36.4645 8

-7 5 -18 212.743 91.0627 17

-7 5 -18 123.115 36.0930 8

-7 5 -19 47.9946 29.2164 8

7 -7 -20 487.009 92.4087 23

7 -7 -20 587.773 103.258 28

7 -7 -19 50.5149 91.8878 23

7 7 -19 168.585 51.5396 22

7 -7 -19 219.734 103.218 28

7 7 -18 2969.53 166.109 22

7 -7 -18 2833.88 207.080 23

7 7 -17 3462.53 194.751 22

7 -7 -17 3534.85 217.109 26

7 -7 -17 3837.57 247.114 23

7 -7 -16 79.8642 113.210 23

7 7 -16 70.1033 49.7807 22

7 -7 -16 93.9405 79.9112 26

7 -7 -15 3485.03 216.292 26

7 -7 -14 2621.97 168.174 26

7 -7 -14 1924.12 130.022 8

7 7 -14 2093.18 144.441 7

7 7 -14 2137.95 152.577 17

7 7 -14 2029.86 148.376 9

7 -7 -14 2579.08 167.358 20

7 7 -14 2427.14 142.646 24

7 -7 -13 6292.74 324.206 26

7 7 -13 5569.78 309.984 9

7 -7 -13 5827.33 316.001 20

7 7 -13 5707.91 312.081 17

7 7 -13 6051.22 304.869 24

7 7 -13 5782.58 319.101 29

7 -7 -13 6852.07 325.696 27

7 7 -13 5666.17 307.723 7

7 -7 -12 1008.49 96.9337 27

7 -7 -12 851.777 128.203 25

7 7 -12 838.316 77.3389 24

7 7 -12 876.981 91.5972 17

7 -7 -12 953.871 91.3932 18

7 -7 -12 943.230 99.0900 26

7 -7 -12 909.788 90.2292 20

7 -7 -12 548.547 115.734 19

7 -7 -11 264.451 71.2307 26

7 7 -11 315.145 83.2639 4

7 -7 -11 340.210 67.3198 27

7 -7 -11 327.406 58.7570 20

7 7 -11 270.823 64.3903 17

7 -7 -11 360.582 62.3081 18

7 -7 -11 367.357 106.413 25

7 7 -11 324.054 54.5727 24

7 -7 -10 2280.86 136.063 20

7 7 -10 2012.12 161.323 4

7 7 -10 2179.72 132.655 17

7 -7 -10 2269.30 137.971 18

7 -7 -10 2265.42 177.721 25

7 -7 -9 27054.8 1204.24 20

7 -7 -9 26652.5 1240.62 25

7 -7 -9 24529.1 1229.58 11

7 7 -9 21458.8 1220.19 13

7 -7 -9 29126.3 1209.23 18

7 7 -9 23076.2 1228.72 4

7 -7 -8 7333.31 352.793 20

7 -7 -8 7517.69 357.256 21

7 -7 -8 7077.93 387.990 25

7 -7 -8 6612.48 371.671 11

7 7 -8 5903.64 370.945 13

7 7 -8 6137.44 370.666 4

7 -7 -8 7499.84 356.365 18

7 7 -8 7524.33 349.811 24

7 7 -7 901.909 66.3189 24

7 7 -7 752.829 93.0795 13

7 -7 -7 879.290 88.6374 11

7 -7 -7 1015.38 81.0206 21

7 7 -6 802.780 79.0759 4

7 7 -6 841.362 97.2280 13

7 -7 -6 1045.77 93.8305 11

7 -7 -6 975.425 109.156 12

7 -7 -6 946.585 80.9772 21

7 -7 -6 1034.50 79.2413 18

7 7 -5 1437.02 95.8668 4

7 -7 -5 1103.49 126.467 12

7 7 -5 1262.22 117.611 13

7 -7 -5 1264.30 109.961 11

7 -7 -5 1276.50 90.4706 21

7 -7 -5 1331.25 90.3067 18

7 7 -4 4004.59 236.611 4

7 7 -4 4428.12 273.572 13

7 -7 -4 4171.28 283.796 12

7 -7 -4 5063.91 235.330 21

7 -7 -4 4916.81 237.142 18

7 7 -3 4802.08 270.274 4

7 -7 -3 5725.68 270.418 18

7 -7 -3 5108.66 331.016 12

7 -7 -3 5735.10 268.872 21

7 -7 -2 97.3133 39.7105 18

7 7 -2 114.801 30.1183 4

7 -7 -2 113.721 36.9410 21

7 -7 -2 218.013 113.036 12

-7 7 1 80.9385 31.7257 16

7 -7 -1 131.787 107.195 12

-7 7 1 36.9006 42.0906 1

-7 7 0 15552.5 686.581 16

-7 7 0 13166.7 695.583 1

7 -7 1 665.537 65.1199 3

-7 7 -1 750.313 66.7457 2

-7 7 -1 730.113 71.9088 1

-7 7 -2 87.1257 42.4640 1

-7 7 -2 135.790 41.2549 2

-7 7 -2 108.068 39.2848 16

7 -7 2 98.8897 53.0779 11

7 -7 2 87.9480 39.3928 3

7 -7 2 143.015 30.9915 20

-7 7 -3 26980.4 1350.17 1

-7 7 -3 26389.9 1344.50 2

-7 7 -3 29596.3 1334.70 16

-7 7 -4 7631.56 413.165 1

-7 7 -4 7355.24 406.528 2

7 -7 4 8887.38 417.860 11

-7 7 -4 8221.07 395.405 16

7 -7 5 6915.72 370.493 11

-7 -7 -5 8015.10 361.701 21

-7 7 -5 6618.38 353.755 16

-7 7 -5 6719.57 375.780 1

-7 7 -5 6741.60 370.480 2

-7 -7 -5 7106.88 350.414 5

7 -7 6 55.6576 56.0178 11

-7 -7 -6 40.4265 55.4131 21

-7 7 -6-20.7253 63.0062 2

-7 7 -6-10.7330 65.2555 1

-7 -7 -6 97.5667 50.6005 26

-7 -7 -6 53.7134 31.7085 5

-7 -7 -7 14608.8 697.410 5

-7 7 -7 13951.0 726.336 2

-7 7 -7 14151.2 730.007 1

7 -7 7 14703.7 725.352 11

-7 -7 -7 13872.5 693.041 21

-7 -7 -7 14862.9 691.240 26

-7 -7 -8 1289.64 98.8694 5

-7 -7 -8 1047.06 96.0187 26

-7 7 -8 941.115 131.341 2

-7 -7 -8 1211.03 100.324 21

-7 -7 -9 4148.38 243.690 5

-7 7 -9 3771.08 279.615 2

-7 -7 -9 4349.67 238.701 21

-7 7 -10 59.7746 72.9364 14

-7 -7 -10 6.40317 61.2425 21

-7 -7 -11 6183.59 353.262 21

-7 -7 -11 7038.95 339.706 10

-7 -7 -11 6566.18 363.231 15

-7 -7 -11 7356.59 339.602 8

-7 -7 -12 55.3063 34.2231 10

-7 -7 -12 20.2982 65.1679 28

-7 -7 -12 13.4200 34.4367 8

-7 -7 -12 31.5691 61.6440 21

-7 -7 -12 77.0221 67.1216 15

-7 -7 -13 2308.02 153.216 28

-7 7 -13 2252.42 148.110 24

-7 -7 -13 2224.19 153.919 15

-7 -7 -14 63.3772 65.9958 28

-7 -7 -14 46.2813 62.3638 15

-7 7 -14 9.09802 52.2421 24

-7 7 -15-120.246 105.936 17

-7 -7 -15-3.11858 63.4267 28

-7 -7 -15 32.0170 57.8108 15

-7 7 -15-16.6352 51.2218 24

-7 7 -16 51.1688 100.595 17

-7 7 -16 12.1074 44.0702 24

-7 7 -17 521.329 97.9747 17

-7 7 -17 309.409 48.8685 24

-7 7 -18 130.530 75.7831 17

7 -9 -19 149.623 81.4457 28

7 9 -19 135.062 44.0782 22

7 -9 -19 143.541 65.4689 23

7 9 -18 90.2528 46.7573 22

7 -9 -18 103.633 80.9124 23

7 -9 -17 1471.22 135.652 23

7 -9 -17 1320.01 121.075 26

7 -9 -16 1231.61 138.645 23

7 9 -16 1175.03 89.8860 22

7 -9 -16 990.470 112.511 26

7 9 -15 603.449 68.6188 22

7 -9 -15 748.490 102.423 26

7 9 -14 117.548 49.6129 22

7 -9 -14 44.8642 85.1518 26

7 -9 -14 33.0074 75.4026 20

7 -9 -13 8443.28 445.154 26

7 -9 -13 8711.33 439.301 20

7 -9 -12 8379.20 469.141 25

7 -9 -12 8862.93 451.549 26

7 -9 -12 8759.57 442.348 20

7 -9 -11 1869.90 132.659 18

7 9 -11 1845.50 126.295 24

7 -9 -11 1722.23 174.246 25

7 -9 -11 2066.09 135.982 20

7 -9 -10 2792.60 165.751 20

7 -9 -10 2657.38 168.087 18

7 -9 -10 2682.59 205.917 25

7 9 -10 2332.39 186.127 13

7 9 -10 2748.01 159.589 24

7 -9 -9 6117.82 294.484 20

7 9 -9 5586.40 288.211 24

7 -9 -9 5793.08 338.327 25

7 9 -9 4765.70 317.803 13

7 -9 -9 5278.47 319.158 11

7 -9 -8 10643.2 498.042 20

7 -9 -8 10416.6 501.298 21

7 9 -8 8644.81 524.482 13

7 -9 -8 9409.04 522.750 11

7 -9 -8 10341.4 538.244 25

7 9 -8 10312.4 492.792 24

7 -9 -7 209.657 73.7469 11

7 -9 -7 148.751 60.7113 18

7 9 -7 112.021 45.1563 24

7 9 -7 137.193 74.5967 13

7 -9 -7 149.031 61.0884 21

7 -9 -6-5.49387 62.7375 11

7 9 -6 4.54380 41.4677 24

7 9 -6-6.68578 68.4880 13

7 -9 -6-26.0055 54.6834 21

7 -9 -6 5.31806 53.1387 18

7 9 -5 8703.37 490.909 13

7 -9 -5 8021.52 478.872 12

7 -9 -5 9874.51 484.347 11

7 -9 -5 9093.27 450.311 18

7 -9 -5 9570.83 449.354 21

7 9 -4 4584.21 284.658 13

7 -9 -4 4205.58 276.265 12

7 -9 -4 4546.54 233.842 21

7 -9 -4 4431.50 235.706 18

7 -9 -3 159.562 90.5264 12

7 9 -3 310.290 87.0447 13

7 -9 -3 196.176 44.9719 21

7 -9 -3 165.024 47.8394 18

7 -9 -2 8027.92 394.869 21

7 -9 -2 8264.34 399.819 18

7 -9 -2 8133.64 472.119 12

7 -9 -1 4406.17 326.436 12

7 9 -1 5069.35 306.682 13

7 -9 -1 4578.91 238.780 21

-7 9 1 4982.12 243.250 16

7 -9 0 9426.86 541.671 12

-7 9 0 9211.90 438.411 16

7 9 0 9813.70 507.859 13

-7 -9 0 8545.50 440.442 15

7 9 1 137.772 79.7759 13

7 -9 1 238.448 42.2910 20

-7 -9 -1 211.595 52.7609 15

7 -9 2 5363.79 298.479 11

7 -9 2 5035.59 258.213 20

-7 -9 -2 5327.12 272.994 15

-7 9 -2 5227.40 265.719 16

-7 -9 -3 665.520 75.2078 15

-7 9 -3 648.655 68.9305 16

7 -9 3 821.719 96.0995 11

-7 9 -4 12334.3 604.049 16

-7 -9 -4 12412.2 609.023 15

7 -9 4 12712.6 631.621 11

-7 -9 -5 104.516 52.5318 26

-7 -9 -5 54.2073 63.1737 15

-7 9 -5 98.4166 55.9131 16

7 -9 5 68.6339 62.9459 11

-7 -9 -6 5676.94 276.802 26

-7 -9 -6 5214.45 288.017 21

7 -9 6 4945.51 300.458 11

-7 -9 -6 4955.82 284.252 15

-7 -9 -7 1439.53 112.689 26

-7 -9 -7 1432.70 122.426 21

-7 -9 -7 1592.53 125.531 15

7 -9 7 1372.35 136.634 11

7 9 8 17167.0 831.418 13

-7 -9 -8 15604.6 793.082 15

-7 -9 -8 16884.6 783.494 26

-7 9 -8 16116.5 815.361 17

-7 -9 -8 15839.9 789.083 21

-7 9 -9 5.60427 107.632 17

-7 -9 -9 110.336 71.7053 21

-7 9 -9 33.8861 81.4654 14

-7 -9 -9-48.6265 77.9359 15

-7 9 -10-42.5257 56.9998 24

-7 -9 -10 40.4780 68.9904 21

-7 -9 -10-8.20121 73.4606 15

-7 9 -10 15.3917 75.5135 14

-7 9 -10-57.0658 109.591 17

-7 9 -11 741.156 132.273 17

-7 -9 -11 518.473 86.1164 21

-7 9 -11 661.763 80.3340 24

-7 9 -12 1392.61 148.430 17

-7 -9 -12 1291.16 109.773 21

-7 9 -12 1360.60 110.986 24

-7 -9 -13 91.2570 57.1989 18

-7 9 -13 171.766 106.774 17

-7 -9 -13 144.162 70.8963 15

-7 9 -13 109.867 60.0512 24

-7 9 -14 290.217 101.870 17

-7 -9 -14 326.070 69.7866 15

-7 9 -15 208.883 92.4935 17

-7 9 -16 220.600 82.8067 17

-7 9 -16 216.308 50.0454 24

-7 9 -17-10.8833 36.7730 24

7 -11 -18 208.379 60.9374 28

7 11 -18 239.090 40.6998 22

7 -11 -17 151.637 60.2973 23

7 11 -17 49.2394 40.9093 22

7 -11 -16 101.197 72.0922 23

7 11 -16 85.7869 43.3706 22

7 -11 -16 11.9951 72.9721 26

7 -11 -15 982.153 116.625 23

7 -11 -15 975.896 109.750 26

7 11 -15 1274.98 85.5564 22

7 11 -14 1137.38 84.0061 22

7 -11 -13 969.101 101.003 20

7 11 -13 913.241 76.8203 22

7 -11 -12 2967.96 211.453 25

7 -11 -12 3266.87 203.070 26

7 -11 -12 2797.49 185.037 20

7 11 -12 3221.56 172.264 22

7 -11 -11 6190.00 363.913 25

7 -11 -11 6576.42 350.205 26

7 -11 -11 6366.69 337.799 20

7 11 -11 6515.30 323.270 22

-7 11 10 1310.67 92.9285 16

7 11 -10 1385.19 95.3115 22

7 -11 -10 1366.04 113.386 20

7 -11 -10 1576.96 153.630 25

7 -11 -10 1508.57 132.354 26

7 11 -9 141.584 78.7485 13

7 -11 -9 28.3520 83.4269 26

7 -11 -9 24.2342 107.607 25

-7 11 9 115.503 45.1794 16

7 11 -9 142.768 43.7204 22

7 -11 -9 81.3752 62.0101 20

7 -11 -9 89.2365 70.4883 18

7 -11 -8 1520.15 159.907 25

-7 11 8 1309.74 92.8148 16

7 -11 -8 1299.80 110.484 18

7 -11 -8 1257.56 129.886 11

7 11 -8 1014.60 134.826 13

7 11 -8 1337.16 88.9416 22

-7 11 7 5355.31 273.491 16

7 11 -7 5424.08 268.016 22

7 -11 -7 5165.20 285.771 18

7 11 -7 5174.06 330.528 13

7 -11 -7 5285.36 316.593 11

7 -11 -7 5315.25 282.977 20

-7 11 6 15226.7 702.373 16

7 -11 -6 14324.7 708.105 21

7 11 -6 15133.2 695.213 22

7 -11 -6 14109.4 710.506 18

7 11 -6 13459.0 759.070 13

7 -11 -6 14775.4 747.957 11

7 -11 -6 14687.5 708.157 20

7 11 -5 447.398 105.981 13

7 -11 -5 572.506 96.8686 11

7 -11 -5 422.924 69.0788 21

7 -11 -5 493.838 77.4270 18

-7 11 5 624.426 67.4423 16

7 -11 -4 132.223 74.4441 11

-7 11 4 58.9909 50.3765 16

7 11 -4 189.832 98.2541 13

7 -11 -4 98.6663 53.4511 21

-7 11 3 2253.03 139.156 16

7 -11 -3 2265.04 137.667 21

7 -11 -3 2198.72 174.911 11

-7 11 2 1523.64 101.945 16

7 -11 -2 1323.54 96.0257 21

-7 -11 1 8151.25 445.140 21

-7 11 1 9594.19 437.303 16

7 11 -1 9082.67 506.931 13

7 -11 -1 8516.35 431.242 20

-7 -11 0 2196.14 153.328 21

-7 11 0 2168.53 138.251 16

7 11 0 2310.47 200.562 13

7 -11 0 2354.98 173.172 11

7 -11 0 2031.91 128.209 20

-7 -11 -1 2926.44 178.565 21

-7 -11 -1 2534.69 160.764 15

7 -11 1 2822.40 194.598 11

7 11 1 2620.11 215.596 13

7 -11 1 2366.99 145.568 20

7 -11 2 676.519 97.8028 11

-7 11 -2 543.725 73.8194 16

-7 -11 -2 583.164 79.8141 15

7 11 2 552.833 111.917 13

7 -11 2 576.359 151.772 12

7 -11 2 491.380 58.3717 20

-7 -11 -2 597.622 92.1262 21

-7 -11 -3 9346.58 484.835 15

-7 11 -3 9951.66 480.410 16

7 11 3 10040.1 543.501 13

7 -11 3 9971.68 515.284 11

7 -11 3 9966.12 593.682 12

-7 -11 -3 9098.04 486.420 21

-7 11 -4 1427.93 117.292 16

-7 -11 -4 1392.97 119.215 15

-7 -11 -4 1163.07 122.401 21

7 11 4 1525.24 156.669 13

7 -11 4 1370.92 137.022 11

-7 11 -4 1401.81 99.5529 24

-7 11 -5 114.830 73.3624 16

-7 -11 -5 170.468 77.6771 15

7 -11 5-12.7391 66.8775 11

7 11 5-50.6497 94.9260 13

-7 11 -5 85.3310 47.9580 24

-7 11 -6 1034.16 89.8732 24

7 -11 6 872.304 112.984 11

-7 -11 -6 1133.21 112.991 15

-7 11 -6 907.118 130.386 17

7 11 6 919.529 128.246 13

-7 11 -7 1838.61 122.581 24

-7 -11 -7 1551.28 134.577 21

-7 11 -7 1486.81 154.445 17

-7 -11 -7 1712.95 139.173 15

-7 11 -8 2733.13 172.184 24

-7 -11 -8 2829.86 184.009 21

-7 11 -8 2801.28 204.980 17

-7 -11 -8 3038.92 190.892 15

-7 11 -9 969.354 91.9925 24

-7 -11 -9 1020.30 105.786 15

-7 -11 -9 967.839 107.923 21

-7 11 -9 813.105 126.225 17

-7 11 -10 107.320 58.1033 24

-7 -11 -10 130.485 73.0750 21

-7 11 -10 89.6895 93.2860 17

-7 -11 -10 115.183 74.6841 15

-7 11 -11 88.3028 58.0569 24

-7 -11 -11-34.0055 66.0823 21

-7 11 -11 182.922 94.1320 17

-7 -11 -11 16.4913 60.9065 18

-7 -11 -11 160.421 75.9157 15

-7 -11 -12 279.503 70.6260 21

-7 11 -12 258.342 60.0518 24

-7 11 -12 285.815 90.9061 17

-7 -11 -12 181.245 62.5172 18

-7 -11 -13 794.801 77.6817 18

-7 11 -13 826.215 103.965 17

-7 11 -13 728.623 75.4820 24

-7 11 -14 134.232 77.1203 17

-7 11 -14 74.5291 50.0181 24

-7 11 -15 56.0517 43.2044 24

7 13 -15 25.1111 32.8625 22

7 13 -14 132.265 41.0805 22

7 -13 -14 105.017 60.6490 23

7 -13 -14 80.4478 68.6770 26

7 13 -13-1.78311 38.7269 22

7 -13 -13 21.0404 70.1740 26

7 -13 -13-3.24735 67.6514 20

7 13 -12 7.58182 39.0781 22

7 -13 -12 130.896 76.5462 26

7 -13 -12-26.0615 65.6042 20

7 13 -11 119.636 41.9622 22

7 -13 -11 122.623 80.2448 26

7 -13 -11 172.739 68.3793 20

7 13 -10 2662.49 144.719 22

7 -13 -10 2455.80 161.544 20

7 -13 -10 2310.68 179.187 25

7 -13 -10 2682.41 172.510 26

7 -13 -9 502.690 108.156 25

-7 13 9 512.985 61.9541 16

7 13 -9 583.685 57.2704 22

7 -13 -9 524.668 94.6824 26

7 -13 -8 625.463 122.636 25

7 -13 -8 616.119 82.8983 20

7 -13 -8 702.998 99.2840 26

-7 13 8 652.503 68.3246 16

7 -13 -7 482.343 121.416 25

-7 13 7 442.496 61.6550 16

7 -13 -7 396.007 89.0421 26

7 -13 -7 497.336 76.1007 20

7 -13 -6 6115.20 327.808 20

-7 13 6 6453.08 322.317 16

7 -13 -5 144.942 62.2522 20

-7 13 5 254.315 60.0335 16

-7 -13 4 34.1699 58.3065 21

7 -13 -4 73.8015 57.0748 20

7 13 -4 85.0345 84.2730 13

-7 13 4 65.4114 56.6147 16

-7 -13 3 66.5517 64.4349 21

7 -13 -3 123.457 74.4714 11

-7 13 3 52.4817 57.0925 16

7 -13 -3 74.6979 57.7357 20

-7 -13 2 6199.98 363.363 21

7 -13 -2 6887.72 356.210 20

7 13 -2 7102.50 430.336 13

7 -13 -2 6706.69 399.138 11

-7 -13 1 10.8594 68.6513 21

7 -13 -1 51.7599 52.6833 20

7 13 -1 9.69135 84.7188 13

-7 13 1-15.5419 61.4072 16

7 -13 0 203.362 52.9021 20

-7 -13 0 166.512 73.6120 21

7 -13 0 80.0005 74.0059 11

7 13 0 58.0733 88.6592 13

-7 -13 0 72.5047 58.6176 18

7 -13 1 186.512 51.8942 20

-7 -13 -1 157.901 76.0558 21

7 -13 1 129.231 76.7905 11

7 13 1 188.808 95.6420 13

-7 -13 -1 217.147 64.6823 18

7 -13 2 3106.71 234.472 11

-7 13 -2 3887.55 213.082 16

-7 -13 -2 3552.58 214.714 21

-7 13 -3 748.010 92.4390 16

-7 -13 -3 610.065 92.2498 21

-7 13 -4 315.786 82.4231 16

-7 13 -4 342.741 93.3792 17

-7 -13 -4 445.562 86.7587 21

-7 -13 -5 168.163 73.3961 18

-7 13 -5 52.5169 80.5178 17

-7 -13 -5 299.668 78.8273 21

-7 -13 -6 86.9754 69.3309 18

-7 13 -6 21.3929 81.0296 17

-7 13 -6 208.901 51.4191 24

-7 -13 -6 303.572 80.1475 21

-7 13 -7-11.0477 45.3831 24

-7 -13 -7-3.54515 67.8946 18

-7 -13 -8 24.5405 75.6540 21

-7 13 -8 52.4125 48.2389 24

-7 -13 -8-27.3246 68.6135 18

-7 -13 -9 114.546 68.9548 21

-7 13 -9 98.8398 50.8460 24

-7 -13 -9 74.2379 61.2788 18

-7 -13 -10 18.1583 62.9154 21

-7 13 -10 38.0109 48.2801 24

-7 -13 -11-37.6087 60.9328 21

-7 13 -11 16.3803 46.9431 24

-7 13 -12-24.8919 46.9110 24

-7 -13 -12 38.5144 49.3113 18

-7 13 -13-6.96935 42.5004 24

7 -15 -12 119.968 56.7745 26

7 -15 -11 377.594 69.3259 26

7 -15 -10 331.039 67.9111 26

-7 15 9 271.592 49.9133 16

7 -15 -9 394.584 70.4296 26

7 -15 -8 76.5798 71.4783 25

-7 15 8 21.5048 46.7674 16

7 -15 -8 88.4975 68.0750 26

7 -15 -8 62.0299 62.3321 20

7 -15 -7 414.051 88.6919 25

-7 15 7 553.529 65.8446 16

7 -15 -7 490.192 77.4110 26

7 -15 -7 479.733 73.8686 20

7 -15 -6 699.445 106.999 25

-7 15 6 686.656 75.2191 16

7 -15 -6 759.800 85.9096 26

7 -15 -6 821.264 83.9755 20

7 -15 -5 360.065 99.3995 25

-7 15 5 381.674 63.5063 16

7 -15 -5 356.135 66.4463 20

7 -15 -5 337.020 72.6962 26

-7 15 4 42.5824 54.5181 16

7 -15 -4 58.7960 56.5223 20

7 -15 -4 93.4693 61.5168 26

7 -15 -3 1991.18 132.285 20

7 -15 -3 1755.23 130.722 26

7 -15 -2 251.479 60.7390 20

-7 15 2 201.267 68.2815 16

7 -15 -2 131.690 59.8018 26

-7 -15 2 191.774 58.4767 21

7 -15 -1 645.520 76.4002 20

-7 -15 1 549.255 76.7331 21

-7 -15 0 101.628 63.1891 21

-7 -15 -1 214.863 69.2746 21

-7 15 -1 354.951 77.0352 16

-7 -15 -2 99.0375 66.1992 21

-7 -15 -2 55.1125 59.3378 18

-7 15 -2 118.020 78.1892 16

-7 -15 -3 215.705 64.9170 18

-7 -15 -3 98.9596 67.2924 21

-7 -15 -4 12.0403 59.6208 18

-7 -15 -4-53.4380 64.4850 21

-7 -15 -5 17.8022 58.4566 18

-7 -15 -5-6.06106 65.7487 21

-7 -15 -6-39.9825 59.3577 18

-7 -15 -6 72.8998 63.2642 21

-7 -15 -7 13.5566 55.5891 18

-7 -15 -7 90.3037 61.2404 21

-7 -15 -8 102.016 55.9024 18

-7 -15 -9 232.157 59.2302 21

7 -17 -2 69.8517 55.0640 25

7 -17 -1 135.487 63.1315 25

7 -17 0-17.3492 64.2243 25

8 0 -21 21.1434 46.9605 8

8 0 -20 2589.79 155.975 8

8 0 -20 2916.64 177.580 7

8 0 -19 37.1068 119.265 28

8 0 -19 58.4038 72.8627 7

8 0 -19 5.19924 52.7528 8

8 0 -18 238.493 80.9927 7

8 0 -18 162.244 130.170 28

8 0 -18 246.199 86.1455 9

8 0 -18 379.031 60.6461 8

8 0 -17 31.1155 71.5331 7

8 0 -17-41.0790 51.4936 8

8 0 -17 118.966 127.534 28

8 0 -17-33.6274 80.5017 9

8 0 -16 4144.30 241.923 9

8 0 -16 3966.59 223.113 22

8 0 -16 3511.08 286.046 4

8 0 -16 4117.47 236.628 7

8 0 -16 3757.36 276.592 28

8 0 -15 7.92487 131.928 23

8 0 -15 2.54087 45.7485 8

8 0 -15 44.8884 47.5410 22

8 0 -15 9.50951 99.7565 4

8 0 -15 75.9673 72.0413 9

8 0 -15 3.54122 63.0446 7

8 0 -14 1066.16 96.5650 7

8 0 -14 1142.99 160.484 23

8 0 -14 1048.84 145.780 4

8 0 -14 1192.21 85.4436 22

8 0 -14 1129.90 122.390 5

8 0 -14 1088.13 92.0434 6

8 0 -14 1123.90 103.056 9

8 0 -14 1141.16 84.6497 8

8 0 -13 17.8139 49.9717 7

8 0 -13 80.0166 112.221 23

8 0 -13-113.892 86.1238 4

8 0 -13-35.1625 39.1891 22

8 0 -13-31.0656 70.6774 5

8 0 -13 6.41478 53.6916 9

8 0 -13 5.04880 48.6858 6

8 0 -13-21.4488 38.1870 8

8 0 -12 65724.7 3095.18 6

8 0 -12 64861.9 3099.76 7

8 0 -12 73329.9 3093.62 8

8 0 -12 74747.2 3139.80 5

8 0 -12 64836.9 3151.63 4

8 0 -12 68009.1 3102.99 9

8 0 -12 66300.3 3091.72 22

8 0 -11 24.1376 32.6228 8

8 0 -11 32.8255 78.9718 4

8 0 -11 6.05156 37.9441 6

8 0 -11 34.2224 55.6218 5

8 0 -11 41.2955 46.7605 9

8 0 -11 16.8640 41.9922 7

8 0 -11 41.7247 55.5590 27

8 0 -10 13229.3 645.628 6

8 0 -10 15020.9 679.413 5

8 0 -10 13292.6 692.821 4

8 0 -9-26.1914 35.4791 6

8 0 -9-5.30152 79.3712 4

8 0 -9 19.3710 47.4140 5

8 0 -8 28758.0 1355.18 6

8 0 -8 30934.0 1397.90 4

8 0 -7-12.3811 59.3509 3

8 0 -7 22.4606 69.2104 4

8 0 -6 14726.1 729.206 3

8 0 -6 15214.6 736.672 4

8 0 -5 88.8332 63.0521 12

8 0 -5 24.8313 53.5205 3

8 0 -4 71379.2 3513.89 12

8 0 -4 66477.4 3505.49 3

8 0 -3 32.9588 77.6710 11

8 0 -3 72.8532 72.0086 12

8 0 -3 11.0653 56.2365 3

8 0 -2 10500.1 558.495 12

8 0 -2 11581.7 554.522 3

8 0 -1 64.0183 84.5009 11

8 0 -1 153.934 84.1634 12

8 0 0 4808.60 282.724 11

8 0 1 54.6754 77.1128 11

8 0 2 28355.9 1355.06 11

8 0 3 42.1357 82.1436 11

-8 0 -4 9532.05 511.051 1

-8 0 -5-31.7802 106.592 1

-8 0 -6 8858.68 463.828 2

-8 0 -6 8937.23 473.702 1

-8 0 -6 7898.75 409.060 14

-8 0 -7 14.9475 109.855 1

-8 0 -7 137.372 90.9438 2

-8 0 -7 11.3928 59.5760 17

-8 0 -8 37629.7 1860.30 17

-8 0 -8 44021.3 1932.69 1

-8 0 -8 42270.5 1924.91 2

-8 0 -9 42.8984 69.6897 28

-8 0 -9 58.2113 74.0863 17

-8 0 -9 46.6572 121.353 1

-8 0 -9-25.7230 108.442 2

-8 0 -9-10.9057 41.8326 10

-8 0 -10 726.309 66.8510 10

-8 0 -10 634.050 64.6466 8

-8 0 -10 761.673 97.0103 28

-8 0 -10 600.044 102.975 17

-8 0 -10 641.854 151.251 1

-8 0 -10 484.120 137.717 2

-8 0 -10 541.376 85.0942 5

-8 0 -11 65.2709 46.3557 10

-8 0 -11 12.4942 45.0813 8

-8 0 -11-39.8369 84.5507 28

-8 0 -11 113.677 89.6019 17

-8 0 -11-11.2191 126.618 1

-8 0 -11 116.454 114.604 2

-8 0 -11 35.4670 64.8352 5

-8 0 -12 5792.77 398.407 2

-8 0 -12 5747.42 338.417 5

-8 0 -12 5764.43 403.597 1

-8 0 -12 6386.02 300.623 8

-8 0 -12 5960.06 332.413 17

-8 0 -12 6108.14 299.706 10

-8 0 -13-28.9597 70.8512 5

-8 0 -13-65.2252 138.098 2

-8 0 -13 14.0015 49.2831 8

-8 0 -13 31.7377 103.667 17

-8 0 -13-20.7396 52.8244 10

-8 0 -14 764.956 113.384 5

-8 0 -14 621.145 173.214 2

-8 0 -14 788.210 84.2114 10

-8 0 -14 1027.28 137.872 17

-8 0 -14 838.778 81.3493 8

-8 0 -15-30.0284 71.8848 5

-8 0 -15 10.8431 101.937 17

-8 0 -15 2.95773 55.0480 10

-8 0 -15 5.62824 52.2982 8

-8 0 -16 3338.25 213.401 17

-8 0 -16 2955.47 171.215 8

-8 0 -16 2748.94 168.398 10

-8 0 -17 2.45666 47.8860 8

-8 0 -17 104.159 48.3057 10

-8 0 -18 845.980 73.6945 8

-8 0 -19 14.2040 37.9794 8

-8 0 -20 84.6191 33.9567 8

8 -2 -21 996.172 135.610 23

8 -2 -20 53.1359 50.1254 8

8 2 -20 43.9135 48.3227 8

8 -2 -20 89.6679 109.652 28

8 -2 -20 60.8405 119.891 23

8 -2 -19 406.615 61.6690 8

8 2 -19 326.614 58.7800 8

8 -2 -19 445.691 77.3221 7

8 2 -19 324.432 80.4795 7

8 -2 -19 230.898 123.749 28

8 -2 -19 243.651 141.830 23

8 2 -18 1711.01 222.406 23

8 -2 -18 2400.51 228.145 23

8 -2 -18 2403.69 159.885 7

8 2 -18 2397.05 159.406 7

8 -2 -18 2459.35 166.835 9

8 2 -18 2161.10 161.687 9

8 -2 -18 2309.59 205.619 28

8 -2 -18 2380.51 144.788 8

8 2 -18 2527.49 146.361 8

8 2 -17 2483.42 154.037 22

8 2 -17 1908.94 228.353 23

8 -2 -17 2600.42 235.283 23

8 -2 -17 2561.94 169.893 7

8 2 -17 2749.09 172.677 7

8 -2 -17 2698.52 173.918 9

8 2 -17 2622.21 175.510 9

8 2 -17 2250.72 213.759 28

8 -2 -17 2559.85 153.495 8

8 2 -17 2819.64 156.532 8

8 2 -16 636.151 134.234 4

8 -2 -16 508.788 130.781 4

8 -2 -16 604.428 90.3982 9

8 2 -16 624.558 95.8600 9

8 2 -16 748.934 73.6541 22

8 -2 -16 691.177 153.365 23

8 2 -16 716.468 180.469 23

8 2 -16 704.530 90.7823 7

8 -2 -16 771.726 93.6431 7

8 -2 -16 890.294 123.900 27

8 2 -16 638.962 145.256 28

8 2 -16 851.575 75.8585 8

8 -2 -16 771.804 73.9963 8

8 -2 -15 6596.06 347.227 9

8 2 -15 6620.85 349.101 9

8 2 -15 5798.31 397.231 4

8 -2 -15 6318.22 404.108 4

8 2 -15 6353.02 344.067 7

8 -2 -15 6849.72 346.023 7

8 2 -15 6864.82 365.587 27

8 -2 -15 7112.90 364.583 27

8 2 -15 4725.24 371.425 28

8 2 -15 4570.16 394.515 23

8 2 -15 6631.95 332.038 22

8 2 -15 7346.47 336.052 8

8 -2 -15 7132.49 334.424 8

8 2 -14 896.375 72.3742 8

8 -2 -14 789.198 69.9196 8

8 2 -14 419.251 155.169 23

8 2 -14 510.027 114.526 4

8 -2 -14 643.879 131.526 4

8 2 -14 762.615 68.3762 22

8 2 -14 958.237 82.7968 6

8 -2 -14 951.954 81.4905 6

8 -2 -14 736.606 103.202 27

8 2 -14 425.733 124.585 28

8 2 -14 798.393 108.685 27

8 -2 -14 765.345 87.4676 9

8 2 -14 792.380 87.6040 9

8 -2 -14 821.504 86.1833 7

8 2 -14 714.411 84.0343 7

8 2 -13 2432.42 151.708 7

8 -2 -13 2214.13 150.359 7

8 2 -13 2514.71 139.421 22

8 2 -13 1593.97 197.543 23

8 -2 -13 2434.02 179.333 5

8 2 -13 2870.83 190.652 5

8 2 -13 2445.01 202.366 4

8 -2 -13 2354.12 202.314 4

8 -2 -13 2310.86 144.616 6

8 2 -13 2502.38 147.508 6

8 2 -13 2935.76 173.405 27

8 -2 -13 2718.33 170.300 27

8 2 -13 1594.66 180.021 28

8 2 -13 2357.74 154.846 9

8 -2 -13 2504.61 154.635 9

8 2 -13 2921.87 144.676 8

8 -2 -13 2629.31 141.530 8

8 2 -12 3088.96 185.293 7

8 -2 -12 3398.23 186.420 7

8 2 -12 3161.82 173.329 22

8 2 -12 2043.71 223.275 23

8 -2 -12 3810.17 219.145 5

8 2 -12 3565.08 216.330 5

8 -2 -12 3571.51 239.106 4

8 2 -12 2969.47 231.821 4

8 -2 -12 3469.73 180.086 6

8 2 -12 3034.36 177.688 6

8 2 -12 2658.79 181.894 29

8 2 -12 1756.85 195.842 28

8 2 -12 3803.89 201.270 27

8 -2 -12 3986.79 205.336 27

8 2 -12 3356.75 190.183 9

8 -2 -12 3640.91 191.678 9

8 2 -12 3708.45 177.056 8

8 -2 -12 3582.45 175.526 8

8 2 -11 13381.2 672.536 7

8 -2 -11 13911.2 673.466 7

8 2 -11 15344.1 686.410 27

8 -2 -11 15884.0 688.957 27

8 2 -11 13902.6 669.532 6

8 -2 -11 13841.0 668.845 6

8 2 -11 13880.3 675.408 9

8 -2 -11 14629.0 676.618 9

8 2 -11 14113.4 665.077 22

8 2 -11 16121.8 709.971 5

8 -2 -11 15772.2 707.756 5

8 2 -11 13622.4 718.523 4

8 -2 -11 13691.7 720.277 4

8 2 -11 15260.5 667.232 8

8 -2 -11 15016.2 666.431 8

8 2 -10 4450.94 234.288 5

8 -2 -10 4092.58 228.467 5

8 2 -10 3721.57 243.152 4

8 -2 -10 3890.55 247.585 4

8 2 -10 3673.60 199.580 6

8 -2 -10 3754.82 198.950 6

8 -2 -10 4348.51 212.246 19

8 -2 -10 3358.16 215.584 20

8 -2 -9 13897.7 700.642 4

8 2 -9 13761.2 701.441 4

8 -2 -9 12987.6 656.791 6

8 2 -9 14021.1 657.935 6

8 -2 -9 12433.1 667.405 20

8 2 -8 2511.64 172.992 4

8 -2 -8 2931.53 176.475 4

8 2 -8 2159.78 125.849 13

8 -2 -8 2444.05 130.635 6

8 2 -8 2395.81 130.851 6

8 -2 -8 3246.85 172.596 11

8 2 -7 75731.9 3760.43 13

8 2 -7 82634.9 3794.40 4

8 -2 -7 84969.8 3795.58 4

8 -2 -6 86107.1 4349.57 11

8 -2 -6 92822.6 4331.52 3

8 2 -6 96850.8 4334.56 3

8 -2 -6 98310.0 4340.73 4

8 2 -6 93037.8 4338.54 4

8 -2 -5 6038.04 336.071 12

8 -2 -5 5942.73 344.725 11

8 2 -5 5914.61 317.391 3

8 -2 -5 6132.12 317.871 3

8 -2 -4 42762.6 1957.03 12

8 -2 -4 40577.0 1952.53 11

8 2 -4 42127.9 1936.47 3

8 -2 -4 40356.7 1934.02 3

8 -2 -3 44052.7 1917.15 12

8 2 -3 40373.3 1882.21 12

8 -2 -3 40195.8 1900.90 11

8 -2 -3 37811.7 1886.71 3

8 2 -3 40841.5 1890.10 3

8 2 -2 80545.1 3790.73 12

8 2 -2 80449.6 3799.62 3

8 -2 -2 77716.2 3798.01 3

8 2 -1 8598.60 433.346 12

8 -2 -1 7656.40 443.351 11

8 2 -1 9015.18 443.945 3

8 -2 -1 9046.07 442.548 3

8 -2 0 27383.6 1439.74 11

8 2 0 33050.4 1435.07 12

8 -2 1 15513.3 771.855 11

8 -2 2 38735.6 1840.27 11

-8 2 -3 44913.9 2122.38 1

8 -2 3 39891.8 2126.66 11

-8 2 -4 11769.7 583.935 1

-8 -2 -4 12021.3 586.238 1

-8 2 -5 2135.72 189.832 1

-8 -2 -5 2150.41 186.044 1

8 -2 5 2368.83 183.467 11

-8 2 -6 271.608 74.5048 14

8 -2 6 213.239 85.3323 11

-8 2 -6 165.632 88.6291 2

-8 -2 -6 166.445 92.8906 2

-8 2 -6 183.921 101.872 1

-8 -2 -6 27.1190 122.135 1

-8 2 -7 20197.4 1116.29 17

-8 2 -7 26910.4 1120.84 14

8 -2 7 28253.6 1145.36 11

-8 2 -7 21863.9 1162.86 1

-8 -2 -7 23993.0 1169.39 1

-8 -2 -7 22289.3 1162.24 2

-8 2 -7 21429.2 1159.38 2

-8 2 -8 1606.59 114.583 14

-8 2 -8 1436.79 168.749 1

-8 -2 -8 1485.89 174.811 1

-8 -2 -8 1374.39 160.466 2

-8 2 -8 1328.23 154.631 2

-8 2 -8 1245.19 124.151 17

-8 -2 -9 7704.74 459.254 2

-8 2 -9 7444.45 455.771 2

-8 -2 -9 8381.02 388.169 10

-8 2 -9 8476.35 388.072 10

-8 2 -9 7613.04 411.769 17

-8 2 -9 7361.81 393.775 14

-8 -2 -9 7066.01 396.272 28

-8 2 -9 7937.56 469.005 1

-8 -2 -9 8388.88 475.375 1

-8 2 -10 80.5864 44.3202 10

-8 -2 -10 85.7671 46.5153 10

-8 2 -10 4.55578 90.3284 17

-8 -2 -10 101.659 80.9931 17

-8 2 -10 152.421 43.4147 8

-8 -2 -10 121.717 44.7334 8

-8 -2 -10 49.8376 69.1543 28

-8 2 -10 245.905 121.580 2

-8 -2 -10 76.0397 120.287 2

-8 2 -10-40.5693 138.173 1

-8 -2 -10-81.0422 129.811 1

-8 2 -10 90.8813 60.9599 5

-8 -2 -10 80.9510 65.5065 5

-8 -2 -11 5451.33 385.230 2

-8 2 -11 5158.05 381.355 2

-8 -2 -11 6087.06 295.036 10

-8 2 -11 5881.39 294.900 10

-8 -2 -11 5648.21 319.237 17

-8 2 -11 5102.72 323.103 17

-8 2 -11 6433.57 296.948 8

-8 -2 -11 6276.74 295.631 8

-8 -2 -11 4474.11 304.815 28

-8 2 -11 5873.77 392.545 1

-8 -2 -11 6368.21 400.886 1

-8 2 -11 5074.61 325.399 5

-8 -2 -11 5465.39 327.083 5

-8 -2 -12 2268.09 136.315 10

-8 2 -12 2209.62 135.657 10

-8 -2 -12 2297.54 170.551 17

-8 2 -12 1966.08 177.848 17

-8 -2 -12 1535.38 152.753 28

-8 2 -12 2274.58 134.632 8

-8 -2 -12 2223.89 133.184 8

-8 2 -12 2076.27 230.645 2

-8 -2 -12 2133.40 238.340 2

-8 2 -12 1914.39 167.254 5

-8 -2 -12 1990.24 167.596 5

-8 -2 -13 6656.69 332.858 10

-8 2 -13 6831.78 334.550 10

-8 -2 -13 7286.35 368.801 17

-8 2 -13 6592.53 372.719 17

-8 -2 -13 5383.59 348.243 28

-8 2 -13 6948.82 334.096 8

-8 -2 -13 6388.94 330.915 8

-8 2 -13 5866.81 373.374 5

-8 -2 -13 6144.63 374.212 5

-8 -2 -13 6584.37 448.584 2

-8 2 -13 5752.72 437.988 2

-8 2 -14 712.543 180.341 2

-8 -2 -14 423.705 158.310 2

-8 -2 -14 444.730 71.5431 15

-8 2 -14 740.981 107.834 5

-8 -2 -14 450.192 93.0086 5

-8 2 -14 680.246 75.6279 10

-8 -2 -14 716.728 76.2670 10

-8 -2 -14 487.360 104.236 28

-8 2 -14 613.450 73.9039 8

-8 -2 -14 650.327 72.2823 8

-8 -2 -14 833.396 125.638 17

-8 2 -14 578.979 134.335 17

-8 2 -15 192.585 108.188 17

-8 -2 -15 136.897 53.7752 8

-8 2 -15 84.5095 54.2483 8

-8 2 -15 183.476 57.9621 10

-8 -2 -15 106.120 54.7924 10

-8 2 -16 853.064 129.866 17

-8 2 -16 799.753 77.0072 8

-8 -2 -16 871.925 78.6165 8

-8 -2 -16 694.880 74.4454 10

-8 2 -16 874.452 79.0397 10

-8 2 -17 1144.03 129.309 17

-8 2 -17 1061.71 83.5997 8

-8 -2 -17 987.079 81.8632 8

-8 -2 -17 959.387 81.6552 10

-8 2 -17 1004.36 83.1068 10

-8 2 -18 122.744 87.1032 17

-8 2 -18-2.04826 42.3727 8

-8 -2 -18 2.04397 39.6878 8

-8 2 -19-5.00091 37.5537 8

-8 -2 -19-23.2073 37.8652 8

-8 2 -20 18.9947 28.9290 8

-8 -2 -20 54.0229 30.3816 8

8 -4 -21 816.010 117.144 23

8 -4 -21 820.673 115.425 28

8 -4 -20 580.242 122.147 23

8 -4 -20 525.727 117.943 28

8 -4 -19 224.953 128.139 23

8 -4 -19 291.958 121.747 28

8 -4 -19 117.438 48.0829 8

8 4 -19 162.377 49.3729 8

8 4 -18 2751.11 165.642 22

8 -4 -18 2538.21 225.710 23

8 4 -18 2993.36 163.617 8

8 -4 -18 2871.08 163.037 8

8 -4 -18 2936.23 178.283 7

8 4 -18 2779.41 178.875 7

8 4 -17 883.669 97.5322 9

8 -4 -17 925.398 101.532 9

8 4 -17 556.371 84.7494 7

8 -4 -17 828.415 92.0060 7

8 4 -17 911.085 80.1850 22

8 4 -17 700.313 70.8314 8

8 -4 -17 692.838 70.9951 8

8 -4 -17 982.669 160.427 23

8 4 -16 2948.62 185.607 7

8 -4 -16 3372.44 188.383 7

8 -4 -16 3528.14 210.239 27

8 -4 -16 2303.12 186.719 26

8 4 -16 3025.26 192.418 9

8 -4 -16 3024.12 190.664 9

8 4 -16 2882.27 171.268 22

8 -4 -16 2848.64 242.444 23

8 -4 -16 2941.43 168.865 8

8 4 -16 3413.14 173.441 8

8 -4 -15 2198.73 140.427 7

8 4 -15 2018.78 139.484 7

8 -4 -15 1922.59 159.160 27

8 -4 -15 1918.82 193.902 23

8 4 -15 1976.52 141.868 9

8 -4 -15 2117.83 145.538 9

8 -4 -15 1574.97 139.595 26

8 4 -15 1810.22 191.258 4

8 -4 -15 1800.70 181.551 4

8 4 -15 1930.16 125.308 22

8 -4 -15 2139.94 124.483 8

8 4 -15 2355.70 127.957 8

8 4 -14 167.388 47.8300 8

8 -4 -14 211.159 47.4680 8

8 -4 -14 193.867 98.6746 4

8 4 -14 164.217 102.955 4

8 -4 -14 233.201 65.1112 7

8 4 -14 197.266 66.9386 7

8 -4 -14 172.666 84.2274 27

8 4 -14 325.744 92.1384 27

8 -4 -14 249.453 74.8176 26

8 -4 -14 215.452 68.1736 9

8 4 -14 225.539 70.3694 9

8 4 -14 63.4781 143.856 23

8 4 -14 173.200 48.3136 22

8 -4 -13 239.428 82.4844 20

8 -4 -13 195.932 44.2445 8

8 4 -13 173.424 45.2418 8

8 4 -13 155.603 49.6111 6

8 -4 -13 231.755 52.5820 6

8 4 -13 134.891 87.9788 4

8 -4 -13 220.179 98.7797 4

8 4 -13 221.821 63.9907 7

8 -4 -13 244.736 60.5903 7

8 -4 -13 342.327 72.1740 26

8 -4 -13 327.255 67.0917 9

8 4 -13 269.368 67.7896 9

8 4 -13 264.231 139.482 23

8 4 -13 340.517 50.5299 24

8 4 -13 324.716 50.7388 22

8 -4 -13 439.866 82.2292 27

8 4 -13 325.716 65.5323 29

8 4 -13 367.861 86.3797 27

8 -4 -12 12630.4 683.852 20

8 4 -12 13608.4 730.585 4

8 -4 -12 14427.5 731.522 4

8 4 -12 14380.0 686.242 29

8 4 -12 14779.8 692.731 27

8 -4 -12 14989.8 691.977 27

8 4 -12 14157.2 683.846 17

8 -4 -12 14153.7 677.136 7

8 4 -12 13752.6 677.475 7

8 4 -12 14748.4 670.887 24

8 4 -12 14104.0 681.389 9

8 -4 -12 15023.9 681.684 9

8 4 -12 15604.7 670.198 8

8 -4 -12 14919.3 668.353 8

8 4 -12 15926.4 675.909 6

8 -4 -12 14929.4 673.777 6

8 -4 -12 13257.9 679.260 26

8 -4 -11 3579.20 192.777 19

8 -4 -11 3133.11 176.879 20

8 4 -11 2976.01 218.611 4

8 -4 -11 2802.56 212.629 4

8 4 -11 3177.15 181.867 27

8 -4 -11 3517.55 185.292 27

8 4 -11 3212.64 177.465 17

8 4 -11 2843.69 169.548 7

8 -4 -11 2765.40 166.823 7

8 4 -11 3065.12 160.309 8

8 -4 -11 2921.02 158.278 8

8 4 -11 3240.45 163.867 24

8 4 -11 3062.93 172.917 9

8 -4 -11 3157.35 173.496 9

8 -4 -11 2872.76 162.897 6

8 4 -11 2864.97 163.903 6

8 -4 -11 2979.26 173.557 26

8 4 -10 15450.4 681.670 6

8 -4 -10 14961.5 680.832 6

8 -4 -10 15517.1 697.879 27

8 4 -10 13841.3 687.003 17

8 4 -10 13874.3 727.046 4

8 -4 -10 14905.6 729.921 4

8 4 -10 14772.9 680.307 24

8 -4 -10 13645.5 686.015 20

8 4 -9 28270.0 1206.91 6

8 -4 -9 26463.5 1205.28 6

8 4 -9 26726.7 1252.04 4

8 -4 -9 28290.6 1253.51 4

8 -4 -9 21179.2 1246.29 11

8 -4 -9 27852.5 1213.02 20

8 4 -9 21167.8 1209.71 13

8 4 -9 26280.2 1211.41 17

8 4 -8 21265.0 934.619 6

8 -4 -8 20384.3 933.631 6

8 -4 -8 21120.2 975.329 4

8 4 -8 21117.8 977.521 4

8 -4 -8 17853.7 977.214 11

8 -4 -8 20023.6 938.551 20

8 4 -8 19404.2 936.244 17

8 4 -8 18133.3 944.892 13

8 4 -7 674.854 70.2621 13

8 -4 -7 805.095 101.794 12

8 4 -7 799.299 56.8582 6

8 -4 -7 841.459 58.0757 6

8 4 -7 715.674 95.7727 4

8 -4 -7 765.835 91.1601 11

8 -4 -7 765.261 63.8041 18

8 -4 -7 766.802 63.3314 20

8 -4 -7 743.481 90.4784 4

8 -4 -6 79893.4 3668.82 20

8 -4 -6 77693.8 3692.52 11

8 -4 -6 77305.8 3712.70 12

8 4 -6 78032.1 3698.65 4

8 -4 -6 85575.5 3701.99 4

8 -4 -5 35797.7 1734.65 12

8 -4 -5 36512.2 1705.45 11

8 4 -5 38154.9 1710.82 3

8 -4 -5 34730.6 1705.65 3

8 4 -5 35333.9 1713.26 4

8 -4 -5 37928.7 1715.09 4

8 -4 -4 502.159 111.942 12

8 -4 -4 387.457 63.2828 11

8 4 -4 355.719 65.1344 3

8 -4 -4 364.347 63.7257 3

8 4 -3 939.417 88.9812 3

8 -4 -3 905.438 86.6068 3

8 -4 -3 919.344 137.630 12

8 -4 -3 987.553 87.3917 11

8 4 -2 76260.0 3425.19 3

8 -4 -2 68824.6 3418.70 3

8 -4 -2 80726.4 3470.20 12

8 -4 -1 104048. 5100.37 3

8 4 -1 116535. 5106.38 3

8 -4 -1 111423. 5092.52 11

8 -4 0 11047.9 528.054 3

8 -4 0 9923.24 511.316 11

8 -4 1 9234.33 461.251 11

8 -4 2 70548.8 3263.70 11

-8 4 -2 65828.6 3279.29 1

-8 -4 -2 75202.8 3284.14 1

8 -4 3 5273.74 304.691 11

-8 4 -3 5588.84 325.893 1

-8 -4 -3 6194.66 331.733 1

8 -4 4 52184.5 2545.89 11

-8 4 -4 53780.9 2561.97 1

-8 -4 -4 58513.7 2566.43 1

-8 4 -5 4744.65 288.975 2

-8 -4 -5 5032.60 293.252 2

8 -4 5 4518.54 288.627 11

-8 4 -5 4779.00 297.711 1

-8 -4 -5 5121.44 300.566 1

-8 -4 -6 19228.8 896.626 2

-8 4 -6 18001.3 890.854 2

-8 4 -6 18646.6 900.433 1

-8 -4 -6 19035.7 901.387 1

-8 -4 -7 822.440 75.5732 21

-8 4 -7 967.560 131.149 2

-8 4 -7 910.431 137.921 1

-8 -4 -7 1165.84 149.668 1

-8 -4 -7 1041.72 134.840 2

-8 4 -7 1165.39 105.858 14

8 -4 7 781.365 128.939 11

-8 4 -8 20759.2 836.213 14

-8 4 -8 14568.6 865.218 2

-8 -4 -8 16258.9 874.535 2

-8 4 -8 15852.5 838.392 17

-8 -4 -8 14176.6 807.997 21

-8 -4 -8 18565.0 811.090 10

-8 4 -8 19415.1 812.286 10

-8 4 -8 16372.2 880.021 1

-8 -4 -8 17195.3 883.069 1

-8 4 -9 601.565 84.3296 14

-8 4 -9 497.546 128.774 2

-8 -4 -9 398.007 119.918 2

-8 4 -9 290.413 118.759 1

-8 -4 -9 359.692 125.495 1

-8 4 -9 553.712 55.0514 10

-8 -4 -9 468.797 53.9479 10

-8 -4 -9 483.726 57.4250 21

-8 4 -9 481.456 53.2475 8

-8 -4 -9 465.476 51.7478 8

-8 4 -9 286.312 99.2611 17

-8 4 -9 337.672 69.8119 5

-8 -4 -9 298.911 65.8288 5

-8 -4 -9 443.107 71.6797 28

-8 4 -10 382.901 136.213 2

-8 -4 -10 422.809 134.992 2

-8 4 -10 488.294 127.776 1

-8 -4 -10 532.025 146.588 1

-8 4 -10 500.290 57.4103 10

-8 -4 -10 460.107 56.5615 10

-8 4 -10 469.945 80.0507 5

-8 -4 -10 368.868 77.6740 5

-8 4 -10 470.707 113.567 17

-8 -4 -10 383.465 52.7493 8

-8 4 -10 517.545 56.8324 8

-8 -4 -10 512.106 76.5051 28

-8 4 -10 473.698 78.8649 14

-8 4 -11 5969.29 413.505 2

-8 -4 -11 6301.58 423.072 2

-8 -4 -11 6736.81 328.547 10

-8 4 -11 7108.72 329.029 10

-8 4 -11 6153.22 363.039 5

-8 -4 -11 6262.04 364.097 5

-8 4 -11 6084.62 369.565 17

-8 4 -11 7148.77 329.193 8

-8 -4 -11 6744.75 327.139 8

-8 -4 -11 5041.86 334.134 28

-8 4 -11 6167.45 416.517 1

-8 4 -12 5699.04 285.418 10

-8 -4 -12 5570.97 284.897 10

-8 4 -12 5286.73 321.210 5

-8 -4 -12 5341.08 322.080 5

-8 4 -12 5838.65 332.510 17

-8 4 -12 5983.29 284.667 8

-8 -4 -12 5602.94 283.184 8

-8 -4 -12 4323.14 295.141 28

-8 4 -12 5571.30 382.532 2

-8 -4 -12 5457.87 387.756 2

-8 4 -13 420.708 128.504 17

-8 4 -13 548.821 66.7044 10

-8 -4 -13 566.725 64.4461 10

-8 -4 -13 376.688 73.3718 15

-8 4 -13 518.789 61.9277 8

-8 -4 -13 561.469 61.1754 8

-8 4 -13 473.276 144.542 2

-8 -4 -13 425.428 145.291 2

-8 4 -13 256.074 78.3083 5

-8 -4 -13 595.026 92.9476 5

-8 -4 -13 338.938 89.7747 28

-8 4 -14 632.610 138.616 17

-8 4 -14 640.486 158.557 2

-8 -4 -14 616.026 149.568 2

-8 -4 -14 752.104 71.3652 10

-8 4 -14 833.350 74.2978 10

-8 4 -14 552.499 93.7566 5

-8 -4 -14 553.918 94.4791 5

-8 -4 -14 431.080 75.2991 15

-8 -4 -14 545.043 99.4757 28

-8 4 -14 773.148 71.1342 8

-8 -4 -14 599.415 67.0660 8

-8 -4 -15 140.739 82.6370 28

-8 4 -15 361.053 52.0694 8

-8 -4 -15 265.161 52.0533 8

-8 4 -15 284.948 121.725 17

-8 -4 -15 200.726 61.0738 15

-8 4 -15 139.209 53.7553 24

-8 -4 -15 216.017 52.4069 10

-8 4 -15 310.964 56.1810 10

-8 4 -16 121.779 45.6007 8

-8 -4 -16 50.3627 40.8544 8

-8 4 -16 57.4039 44.6726 10

-8 -4 -16 118.165 44.9103 10

-8 4 -16 58.6978 45.1357 24

-8 4 -16 146.756 112.037 17

-8 4 -17 229.724 102.483 17

-8 4 -17 233.928 45.6549 8

-8 -4 -17 187.131 44.6639 8

-8 4 -18 416.149 97.0167 17

-8 4 -18 412.398 49.5580 8

-8 -4 -18 400.946 47.9313 8

-8 4 -19 202.387 36.5178 8

8 -6 -21 1040.80 103.619 23

8 -6 -21 1041.39 110.893 28

8 -6 -20 511.463 104.212 23

8 -6 -20 345.107 104.011 28

8 -6 -19 273.084 112.006 23

8 6 -19 252.789 56.0442 22

8 -6 -18 580.503 124.305 23

8 6 -18 510.618 65.2856 22

8 -6 -17 1529.47 175.445 23

8 6 -17 1646.63 114.719 22

8 6 -17 1595.36 108.502 8

8 -6 -17 1966.44 127.174 7

8 -6 -16 3248.21 199.735 26

8 6 -16 3188.61 176.480 22

8 6 -16 2856.49 189.051 9

8 -6 -16 2856.22 169.127 8

8 -6 -16 3350.58 186.050 7

8 6 -16 2978.65 171.181 8

8 6 -16 2957.51 185.193 7

8 6 -15 268.511 55.5061 22

8 -6 -15 364.367 85.9960 26

8 6 -15 284.484 66.6487 7

8 -6 -15 334.248 67.3700 7

8 -6 -15 358.305 90.7010 27

8 -6 -15 239.003 51.1929 8

8 6 -15 252.499 48.0389 8

8 -6 -15 320.323 71.3260 9

8 6 -15 241.268 69.4745 9

8 6 -14 1503.55 111.084 22

8 6 -14 1807.78 124.208 7

8 -6 -14 1730.67 119.756 7

8 -6 -14 1440.93 130.917 26

8 6 -14 1845.54 130.008 9

8 -6 -14 1723.38 126.125 9

8 -6 -14 1350.96 131.782 27

8 6 -14 1791.56 108.845 8

8 -6 -14 1714.52 106.571 8

8 -6 -14 1292.19 134.171 20

8 6 -13 5393.31 293.761 17

8 -6 -13 5634.27 286.992 7

8 6 -13 5588.95 289.526 7

8 -6 -13 5433.26 297.382 26

8 -6 -13 5873.77 291.343 9

8 6 -13 5358.61 290.133 9

8 6 -13 5400.63 296.481 29

8 -6 -13 6243.85 306.735 27

8 6 -13 5579.78 283.297 24

8 6 -13 4786.70 333.360 4

8 -6 -13 5163.27 334.016 4

8 6 -13 5289.58 276.114 8

8 -6 -13 5070.93 274.015 8

8 -6 -13 5191.85 297.335 20

8 -6 -12 1187.62 120.258 19

8 -6 -12 1153.70 98.2593 20

8 -6 -12 1604.84 114.044 27

8 6 -12 1109.79 96.8775 17

8 -6 -12 853.663 82.8131 7

8 6 -12 725.125 81.9495 7

8 -6 -12 1463.58 145.528 25

8 -6 -12 1359.09 109.621 26

8 -6 -12 1008.39 89.1595 9

8 6 -12 1058.05 91.0490 9

8 6 -12 1209.29 83.9025 24

8 6 -12 776.698 71.7668 8

8 -6 -12 612.982 67.6838 8

8 -6 -12 716.293 116.324 4

8 6 -12 738.177 119.602 4

8 -6 -11 1951.28 178.885 19

8 -6 -11 2467.36 159.848 20

8 6 -11 2337.99 194.727 4

8 -6 -11 2513.88 194.199 4

8 -6 -11 2240.78 165.144 27

8 6 -11 2582.40 159.763 17

8 6 -11 2412.32 152.959 7

8 -6 -11 2646.48 145.439 8

8 6 -11 2870.08 148.231 8

8 6 -11 2694.92 152.007 24

8 6 -11 2390.38 155.597 9

8 6 -11 3217.90 154.269 6

8 -6 -11 3067.32 150.875 6

8 -6 -11 2524.46 195.721 25

8 -6 -10 4666.87 251.751 8

8 -6 -10 5525.98 266.850 20

8 -6 -10 5777.67 276.752 27

8 6 -10 5002.62 263.539 17

8 -6 -10 5372.80 268.039 18

8 6 -10 4879.58 260.286 7

8 6 -10 4159.90 269.762 13

8 -6 -10 4824.03 253.550 6

8 6 -10 5199.33 256.967 6

8 -6 -10 5503.43 306.434 25

8 6 -10 4967.36 263.752 9

8 6 -10 4449.48 299.934 4

8 -6 -10 4862.10 301.284 4

8 6 -10 5797.55 262.516 24

8 -6 -9 11425.8 537.757 27

8 6 -9 8583.73 537.061 13

8 -6 -9 10951.7 519.923 6

8 6 -9 12578.0 524.177 6

8 -6 -9 11349.9 529.962 20

8 -6 -9 10328.5 558.854 25

8 -6 -9 11093.9 530.807 18

8 6 -9 10305.5 563.232 4

8 -6 -9 10963.4 563.379 4

8 -6 -9 9372.37 558.463 11

8 -6 -8 4661.43 212.583 6

8 -6 -8 4027.13 219.689 20

8 -6 -8 3515.76 247.911 25

8 6 -8 3561.92 234.815 13

8 -6 -8 4173.58 248.434 11

8 -6 -8 4258.74 221.606 18

8 6 -8 4354.90 253.800 4

8 -6 -8 4331.81 251.142 4

8 -6 -7 9588.61 430.049 6

8 6 -7 9144.68 467.635 4

8 -6 -7 9461.34 466.768 4

8 -6 -7 8595.75 439.360 18

8 6 -7 8057.14 458.380 13

8 -6 -7 9165.80 463.089 11

8 -6 -7 7821.19 475.413 12

8 -6 -7 8919.78 437.940 20

-8 6 7 9200.52 429.503 16

-8 6 6 5631.38 260.170 16

8 6 -6 4925.56 295.236 13

8 -6 -6 5033.82 312.299 12

8 -6 -6 5584.33 290.138 11

8 -6 -6 4988.88 287.074 4

8 6 -6 4855.97 288.313 4

8 6 -5 50343.2 2448.72 4

8 -6 -5 54496.1 2450.84 4

8 6 -5 49985.7 2461.27 13

-8 6 5 53705.8 2422.94 16

8 -6 -5 53093.4 2429.68 18

8 -6 -5 49270.1 2479.64 12

8 -6 -5 54142.7 2452.24 11

8 -6 -5 52018.9 2427.86 20

8 6 -4 19278.5 991.825 4

8 -6 -4 21274.9 994.808 4

8 6 -4 22110.5 993.596 3

-8 6 4 21047.6 968.703 16

8 -6 -4 19541.6 973.164 18

8 -6 -4 21562.9 973.191 20

8 -6 -4 19825.5 1036.59 12

8 -6 -4 21598.5 998.869 11

8 -6 -3 49662.0 2258.10 4

8 -6 -3 47285.8 2249.60 3

8 6 -3 51842.6 2255.48 3

8 -6 -3 48841.7 2309.12 12

8 -6 -3 51419.7 2262.67 11

-8 6 3 47242.0 2232.09 16

8 6 -3 44930.4 2255.02 4

8 -6 -3 48740.9 2235.56 20

-8 6 2 5000.23 242.791 16

8 -6 -2 4441.89 242.454 20

8 6 -2 5728.82 269.042 3

8 -6 -2 4792.63 259.069 3

8 -6 -2 5477.68 335.050 12

-8 6 1 143806. 6637.51 16

8 -6 -1 145216. 6637.99 20

8 6 -1 150753. 6665.14 3

8 -6 -1 129456. 6654.98 3

8 -6 -1 151635. 6666.24 11

8 -6 -1 164517. 6732.06 12

-8 6 0 1006.16 70.5087 16

8 -6 0 1155.22 69.5794 20

8 -6 0 1043.84 95.2343 11

8 6 0 942.671 97.5672 3

-8 6 -1 10110.8 546.537 1

-8 -6 -1 10797.1 547.435 1

8 -6 1 11512.1 543.321 11

-8 6 -1 10411.0 517.272 16

8 -6 1 10588.9 542.654 3

-8 6 -2 12885.0 685.278 1

-8 -6 -2 14647.7 688.438 1

8 -6 2 14027.1 679.768 11

-8 6 -3 29027.5 1428.88 1

-8 -6 -3 31684.4 1431.69 1

8 -6 3 32266.6 1422.56 11

-8 6 -3 28842.1 1397.81 16

-8 -6 -3 31466.1 1426.58 2

-8 6 -3 28251.8 1421.72 2

-8 6 -4 1106.37 120.725 1

-8 -6 -4 1485.20 127.244 1

8 -6 4 1370.66 108.861 11

-8 6 -4 1111.65 112.938 2

-8 -6 -4 1110.33 116.613 2

-8 6 -4 1187.32 93.9105 16

-8 6 -5 720.794 102.400 2

-8 -6 -5 662.859 100.150 2

-8 6 -5 691.591 107.481 1

-8 -6 -5 728.518 110.837 1

8 -6 5 637.991 87.8687 11

-8 6 -6 10077.1 550.710 1

-8 -6 -6 11224.6 555.207 1

-8 -6 -6 10775.3 511.116 21

-8 -6 -6 10860.8 548.871 2

-8 6 -6 9107.88 539.826 2

8 -6 6 10207.6 541.391 11

-8 6 -7 8441.64 405.609 5

-8 -6 -7 7558.05 401.834 5

-8 -6 -7 7788.63 394.501 21

-8 6 -7 8142.56 445.396 1

-8 -6 -7 8047.74 442.893 1

8 -6 7 6802.84 432.506 11

-8 6 -7 7669.47 435.580 2

-8 -6 -7 8468.71 442.911 2

-8 6 -8 1227.74 118.575 14

-8 6 -8 1123.52 99.4494 5

-8 -6 -8 998.641 94.9194 5

-8 -6 -8 1297.68 79.9751 8

-8 6 -8 1070.13 148.104 1

-8 6 -8 810.111 136.068 2

-8 -6 -8 1077.32 144.785 2

-8 -6 -8 1347.53 153.758 1

-8 6 -8 1001.05 133.503 17

-8 -6 -8 1365.46 81.6634 10

-8 -6 -8 1099.36 97.0699 21

-8 6 -9 876.327 99.0423 14

-8 6 -9 497.557 77.2845 5

-8 -6 -9 531.760 79.9964 5

-8 6 -9 620.038 57.5827 8

-8 -6 -9 578.209 56.4721 8

-8 6 -9 464.321 120.653 2

-8 -6 -9 436.163 122.191 2

-8 6 -9 611.691 127.694 1

-8 6 -9 678.619 123.410 17

-8 6 -9 710.419 59.9558 10

-8 -6 -9 614.920 58.9109 10

-8 -6 -9 560.293 71.7394 21

-8 6 -10 489.977 85.5361 14

-8 6 -10 66.2185 119.173 2

-8 -6 -10 159.829 116.534 2

-8 6 -10 214.144 66.0212 5

-8 -6 -10 299.313 68.0350 5

-8 6 -10 285.080 44.9538 8

-8 -6 -10 317.148 45.7632 8

-8 6 -10 203.720 114.517 17

-8 -6 -10 281.860 64.3467 21

-8 -6 -10 300.716 47.0269 10

-8 6 -10 229.930 48.2083 10

-8 6 -11 40.4526 111.354 2

-8 -6 -11 10.0530 117.021 2

-8 6 -11 16.7781 54.4396 5

-8 -6 -11 61.6393 57.6809 5

-8 6 -11 5.92870 117.874 17

-8 -6 -11 2.89342 54.5602 21

-8 6 -11 2.28150 38.8780 10

-8 -6 -11-9.01066 39.9702 10

-8 -6 -11 3.78772 66.5500 15

-8 6 -11-3.14230 63.5931 14

-8 6 -11 12.8049 37.4362 8

-8 -6 -11 2.13690 36.5417 8

-8 6 -12 361.280 128.658 2

-8 6 -12 435.912 54.2831 10

-8 -6 -12 428.914 52.7037 10

-8 6 -12 452.017 130.757 17

-8 -6 -12 397.181 78.0010 15

-8 -6 -12 233.461 70.3522 5

-8 -6 -12 332.204 76.7102 28

-8 6 -12 383.545 49.2704 8

-8 -6 -12 474.828 53.7477 8

-8 -6 -13 34.0766 67.7590 15

-8 -6 -13 43.4716 68.4450 28

-8 6 -13 35.0479 38.8905 10

-8 -6 -13 43.4886 36.8177 10

-8 6 -13 90.4135 109.872 17

-8 6 -13 75.5551 41.4214 8

-8 -6 -13 50.7718 37.1531 8

-8 6 -14 80.9603 111.674 17

-8 -6 -14 3.56374 62.4187 15

-8 -6 -14 10.6911 68.7316 28

-8 -6 -14 33.0335 36.8145 10

-8 6 -14 36.1802 39.3532 10

-8 6 -14-36.9301 54.1736 24

-8 6 -14 17.2924 38.1225 8

-8 -6 -14 66.1403 38.3615 8

-8 6 -15 322.661 112.288 17

-8 6 -15 191.458 54.4993 24

-8 -6 -15 166.303 61.6162 15

-8 6 -15 212.382 43.3018 8

-8 6 -16 284.674 44.3742 8

-8 6 -16 272.431 110.825 17

-8 6 -16 312.743 55.2148 24

-8 6 -17 222.252 94.6990 17

-8 6 -17 173.745 36.2245 8

-8 6 -18-28.4068 77.2632 17

8 -8 -20 183.827 76.0469 28

8 -8 -20 122.732 62.2125 23

8 -8 -19 844.813 114.689 28

8 8 -19 998.000 75.5964 22

8 -8 -19 829.350 107.455 23

8 8 -18 1266.08 93.0276 22

8 -8 -18 1419.78 137.106 23

8 -8 -17 14.2463 75.7918 26

8 8 -17 58.1137 49.7347 22

8 -8 -17 122.278 101.979 23

8 -8 -16 1770.02 168.550 23

8 -8 -16 1684.07 138.253 26

8 -8 -15 4631.09 266.816 26

8 -8 -14 1043.45 116.169 26

8 -8 -14 931.264 111.130 20

8 8 -14 1211.43 91.1163 22

8 -8 -13 882.625 109.251 26

8 -8 -13 1017.73 107.609 20

8 8 -13 1146.91 89.3665 22

8 -8 -12 275.769 84.8106 26

8 -8 -12 242.057 75.1708 20

8 -8 -12 308.061 103.596 19

8 -8 -12 294.102 116.096 25

8 -8 -11 3569.58 224.850 26

8 -8 -11 4105.24 261.118 25

8 -8 -11 3854.71 218.633 20

8 8 -10 150.032 61.6216 13

8 -8 -10 130.361 61.0892 20

8 -8 -10 148.322 107.938 25

8 8 -10 85.9973 49.8184 24

8 -8 -10 137.511 64.6409 18

8 -8 -9 45.7199 54.4542 18

8 8 -9 22.9052 59.5305 13

8 -8 -9 87.3493 57.3133 20

8 -8 -9-44.0321 70.7204 11

8 -8 -9 193.414 104.508 25

8 8 -9 22.9568 45.6525 24

8 -8 -8 2583.96 187.100 11

-8 8 8 2802.54 149.382 16

8 8 -8 2787.07 158.266 24

8 -8 -8 3079.56 164.756 20

8 -8 -7 1048.57 88.6414 18

8 8 -7 860.894 108.202 13

8 -8 -7 819.743 103.720 11

8 8 -7 1123.06 81.8527 24

-8 8 7 1018.35 73.1464 16

8 -8 -6 15043.9 686.544 21

8 8 -6 12838.2 720.104 13

8 -8 -6 13827.7 711.869 11

8 -8 -6 13054.7 724.123 12

8 -8 -6 14830.7 684.884 20

8 -8 -6 14553.1 686.606 18

8 -8 -5 291.803 58.2135 18

8 -8 -5 222.930 92.4527 12

8 8 -5 264.906 81.1313 13

8 8 -5 242.144 34.6241 22

8 -8 -5 177.339 56.2626 21

8 -8 -5 214.938 69.1876 11

8 -8 -4 2009.73 131.823 18

8 8 -4 2313.47 178.516 13

8 -8 -4 2351.97 161.732 11

8 -8 -4 2160.85 185.965 12

8 -8 -4 2011.15 129.039 21

-8 8 4 2443.60 127.450 16

8 -8 -3 8026.39 512.105 12

8 -8 -3 9909.89 481.749 11

-8 8 3 9249.50 442.340 16

8 8 -3 9743.14 505.877 13

8 -8 -3 8996.31 443.971 21

8 -8 -2 8658.01 410.850 21

-8 8 2 8413.11 410.672 16

8 -8 -2 8559.34 412.448 20

8 -8 -2 7683.32 493.524 12

-8 8 1 13145.9 634.297 16

8 -8 -1 13116.5 633.662 20

8 -8 -1 14110.4 671.773 11

8 -8 -1 14889.6 741.316 12

8 -8 0 142.494 66.2188 11

-8 -8 0 147.427 48.1808 15

-8 8 0 155.874 44.5940 16

8 -8 0 396.782 44.1533 20

8 -8 0 154.657 121.796 12

8 -8 1 4730.12 244.221 20

8 -8 1 5401.43 285.311 11

8 -8 2 11929.9 588.914 20

8 -8 2 14196.8 632.876 11

8 -8 3 6046.46 328.567 11

-8 8 -3 5847.20 302.763 16

-8 8 -4 113.433 52.0993 16

8 -8 4 190.177 63.8368 11

8 -8 5 4609.21 275.000 11

-8 -8 -5 4895.87 261.304 21

-8 -8 -5 5202.58 248.825 26

8 -8 6 457.038 85.3346 11

-8 -8 -6 401.419 83.1082 21

-8 -8 -6 561.487 69.5126 26

-8 -8 -7 3736.02 217.521 21

-8 -8 -7 3891.18 219.564 15

8 -8 7 3307.22 241.669 11

-8 -8 -8 271.905 77.6889 15

-8 -8 -8 242.237 76.2818 21

-8 8 -8 230.861 121.914 17

-8 -8 -9 161.072 70.5740 21

-8 8 -9 163.156 81.7226 14

-8 -8 -9 59.7631 74.4906 15

-8 -8 -10 146.956 70.0000 21

-8 8 -10 435.605 84.0698 14

-8 -8 -11 219.104 67.5939 21

-8 8 -12 245.654 70.3437 24

-8 -8 -12 305.163 80.4866 15

-8 -8 -13 288.463 76.0326 15

-8 -8 -14 17.2487 59.8106 15

-8 8 -15 5.12975 50.8800 24

-8 -8 -15 59.8267 58.7168 15

-8 8 -16 17.0926 43.8864 24

-8 8 -17-4.85963 36.6340 24

8 -10 -19 92.1768 57.6008 28

8 -10 -18 1845.43 132.961 28

8 -10 -18 1534.86 117.313 23

8 -10 -17-37.2457 72.4210 26

8 -10 -17 59.6903 81.2894 23

8 10 -17 39.4123 43.2284 22

8 -10 -16 554.725 92.7679 26

8 -10 -16 696.866 110.446 23

8 10 -16 656.567 67.7931 22

8 10 -15 334.045 55.2675 22

8 -10 -15 469.905 90.2215 26

8 10 -14 1787.81 117.175 22

8 -10 -14 1828.19 143.735 26

8 -10 -13 191.469 75.5132 20

8 10 -13 183.345 52.4980 22

8 -10 -13 176.658 87.2921 26

8 -10 -12 2299.74 161.287 20

8 10 -12 2469.34 144.221 22

8 -10 -12 2469.05 193.006 25

8 -10 -11 2887.95 183.120 20

8 10 -11 2876.80 164.367 22

8 -10 -11 3102.09 222.987 25

8 -10 -10 3398.73 236.890 25

8 10 -10 3424.44 176.952 22

8 -10 -10 3329.94 193.711 20

8 -10 -9 1785.25 177.026 25

8 10 -9 1760.46 108.218 22

-8 10 9 1682.15 108.159 16

8 -10 -9 1920.49 129.766 20

8 -10 -8 33.4968 63.2786 18

8 -10 -8-26.8735 72.1077 11

-8 10 8 66.1825 43.4998 16

8 10 -8 29.8216 72.5920 13

8 10 -8 44.8381 39.7793 22

8 -10 -8 77.1080 113.469 25

-8 10 7 3352.12 182.963 16

8 10 -7 3460.56 178.267 22

8 10 -7 2755.13 229.665 13

8 -10 -7 3217.43 223.941 11

8 -10 -7 3297.47 192.748 20

8 -10 -7 3491.76 199.351 18

8 -10 -6 506.429 91.9233 11

-8 10 6 605.789 62.7383 16

8 10 -6 548.959 53.4848 22

8 10 -6 476.668 106.684 13

8 -10 -6 577.901 72.6832 20

8 -10 -6 337.762 73.7625 18

8 -10 -5 3850.36 216.514 20

-8 10 5 4092.21 211.250 16

8 10 -5 3856.50 271.529 13

8 -10 -5 3796.32 252.747 11

-8 10 4 133.707 46.2781 16

8 -10 -4 82.6232 65.0496 11

8 -10 -4 102.730 51.6771 20

8 -10 -4 197.421 83.7006 12

8 10 -4 174.538 87.3757 13

-8 10 3 535.707 60.6946 16

8 -10 -3 427.460 85.3996 11

8 -10 -3 490.075 64.4768 20

8 -10 -3 402.765 103.660 12

8 10 -3 459.758 112.582 13

8 -10 -2 2417.93 144.661 20

-8 10 2 2786.10 150.198 16

8 -10 -1 2507.34 148.601 20

-8 10 1 2800.72 151.695 16

8 10 -1 2412.77 215.448 13

8 -10 -1 2272.16 227.810 12

8 -10 -1 2522.21 189.785 11

8 -10 0 1501.98 98.9370 20

-8 10 0 1445.20 103.719 16

8 -10 0 1503.73 140.472 11

8 10 0 1563.82 166.824 13

8 -10 0 1389.67 195.135 12

-8 -10 -1 27.8931 53.6526 15

8 10 1-28.0935 93.5792 13

8 -10 1 86.1131 135.458 12

8 -10 1 30.7866 67.8082 11

8 -10 1 34.4044 40.1573 20

-8 -10 -2 6369.35 345.889 21

8 -10 2 7105.24 373.202 11

8 -10 2 7615.84 458.004 12

8 -10 2 6169.28 322.045 20

-8 10 -2 6361.53 333.223 16

-8 -10 -2 6421.86 337.789 15

8 10 2 7430.93 412.848 13

8 10 3 140.013 98.9519 13

-8 -10 -3 85.7814 61.2353 15

-8 10 -3 92.6531 60.0295 16

8 -10 3 183.134 69.9066 11

-8 -10 -3 96.0689 77.8471 21

-8 -10 -4 53.0523 66.0317 15

-8 10 -4 13.5757 66.6063 16

8 -10 4 24.5148 63.9926 11

8 10 4-64.6317 104.197 13

-8 -10 -5 87.4547 69.9010 15

-8 10 -5 67.8257 68.7298 16

8 -10 5-6.37402 68.7511 11

8 10 5 126.832 88.7016 13

-8 -10 -6 1291.63 119.721 21

8 10 6 1287.92 150.728 13

8 -10 6 1120.91 129.286 11

-8 -10 -6 1278.54 120.962 15

-8 10 -7 142.759 109.226 17

-8 -10 -7 69.8615 77.2911 21

8 10 7-41.8838 84.5749 13

-8 -10 -7 59.4058 76.8812 15

-8 10 -8 1039.17 96.9220 24

-8 -10 -8 975.246 109.905 21

-8 10 -8 1156.63 147.549 17

-8 -10 -8 1079.05 116.332 15

-8 10 -9 3.36498 55.0015 24

-8 -10 -9 47.4800 72.1358 15

-8 10 -9 85.0183 80.5089 14

-8 -10 -9 76.7486 70.5947 21

-8 10 -9-40.0008 115.657 17

-8 10 -10 116.666 65.0692 24

-8 -10 -10 212.087 75.6262 21

-8 10 -10 252.598 111.528 17

-8 -10 -10 262.710 78.5541 15

-8 10 -11 564.062 76.5689 24

-8 -10 -11 527.211 83.7726 21

-8 10 -11 694.174 120.628 17

-8 -10 -11 640.861 93.3742 15

-8 10 -12 15.2820 57.6671 24

-8 10 -12 59.9692 92.0693 17

-8 -10 -12 66.0909 58.0025 18

-8 10 -13 55.9687 54.1575 24

-8 10 -13 81.4136 92.0146 17

-8 -10 -13 2.77527 52.9003 18

-8 -10 -13 96.1505 67.5137 15

-8 10 -14 2.44536 50.5356 24

-8 10 -14 94.0549 87.7465 17

-8 10 -15 483.896 86.0222 17

8 12 -17 249.644 36.3085 22

8 12 -16 115.627 39.8657 22

8 -12 -16 39.7638 54.2768 23

8 -12 -16 120.018 66.2388 26

8 -12 -15 421.791 83.2299 23

8 12 -15 488.640 53.8196 22

8 12 -14 17.4417 39.9525 22

8 12 -13 932.855 72.8348 22

8 -12 -13 800.009 97.4558 20

8 12 -12 1796.17 113.563 22

8 -12 -12 1778.49 135.449 20

8 -12 -12 2017.08 146.613 26

8 12 -11 286.688 50.4662 22

8 -12 -11 301.694 73.5289 20

8 -12 -11 211.427 88.8621 26

8 -12 -11 312.331 106.928 25

8 12 -10 90.6237 44.4893 22

8 -12 -10 190.181 70.8186 20

8 -12 -10 117.868 86.9255 26

8 -12 -10 195.610 104.794 25

8 -12 -9 865.851 114.099 26

8 12 -9 906.605 73.1715 22

8 -12 -9 1059.04 143.030 25

-8 12 9 865.450 77.2012 16

-8 12 8 1375.84 97.8566 16

8 12 -8 1344.70 91.2870 22

8 -12 -8 1202.52 154.974 25

8 -12 -7 87.9770 71.0565 11

-8 12 7 91.2706 49.7748 16

8 12 -7 71.2913 38.7650 22

8 12 -7-8.74836 78.5553 13

8 -12 -7 78.8136 66.1275 20

8 -12 -7 141.370 122.766 25

-8 -12 6 1188.40 113.421 21

8 -12 -6 1459.74 145.874 11

8 12 -6 1608.40 164.318 13

8 -12 -6 1670.00 122.717 20

-8 12 6 1991.41 117.002 16

-8 -12 5 3715.29 239.866 21

8 12 -5 3989.22 295.532 13

8 -12 -5 4094.09 275.585 11

8 -12 -5 4637.93 241.804 20

-8 12 5 4820.36 236.988 16

-8 -12 4 4225.78 262.984 21

8 12 -4 4694.46 324.776 13

8 -12 -4 4833.42 300.202 11

8 -12 -4 4819.32 256.713 20

-8 12 4 5175.91 257.176 16

-8 -12 3 263.025 78.2753 21

8 -12 -3 407.572 69.0962 20

-8 12 3 264.911 65.2683 16

-8 -12 2 616.660 97.8098 21

8 -12 -2 718.019 76.2669 20

8 -12 -2 685.098 112.007 11

-8 12 2 764.649 81.0289 16

-8 -12 1 28.1076 80.1078 21

8 12 -1 163.050 100.836 13

8 -12 -1 73.2428 52.4165 20

-8 12 1 69.3806 62.8575 16

-8 -12 0 866.468 110.354 21

8 -12 0 1218.22 130.137 11

8 12 0 1175.42 150.615 13

8 -12 0 860.235 80.2748 20

-8 12 0 1030.03 95.6571 16

8 12 1 638.670 123.736 13

-8 -12 -1 544.423 96.5006 21

8 -12 1 642.920 101.813 11

8 -12 1 722.651 71.3313 20

-8 12 -2 1590.80 118.144 16

-8 -12 -2 1363.83 130.788 21

8 12 2 1381.95 165.337 13

8 -12 2 1178.99 136.003 11

8 12 3 86.2446 86.7906 13

-8 12 -3 164.651 73.8590 16

-8 -12 -3 149.780 71.4626 15

8 -12 3 248.449 87.4415 11

-8 -12 -3 110.244 82.9230 21

8 12 4 727.496 123.143 13

8 -12 4 639.622 106.272 11

-8 12 -4 582.712 87.9541 16

-8 -12 -4 669.264 92.0641 15

-8 -12 -4 555.838 98.5428 21

-8 12 -5 1755.66 118.711 24

-8 -12 -5 1846.03 141.820 15

-8 -12 -5 1819.94 146.725 21

-8 12 -5 1655.91 157.567 17

-8 -12 -6 191.779 75.5973 15

-8 12 -6 215.817 55.8001 24

-8 12 -6 290.991 102.068 17

-8 12 -7 42.6679 48.5790 24

-8 -12 -7-17.3581 72.7926 15

-8 -12 -7 54.8893 74.1649 21

-8 12 -7 126.676 98.3966 17

-8 12 -8 588.805 72.9594 24

-8 12 -8 417.823 104.790 17

-8 -12 -8 594.438 92.4449 15

-8 -12 -8 606.336 94.9657 21

-8 -12 -9 21.1495 64.9919 21

-8 12 -9 111.867 92.3087 17

-8 -12 -9 151.008 78.7298 15

-8 12 -9 132.108 57.3659 24

-8 -12 -10 41.9448 66.9250 21

-8 12 -10 28.6790 49.8346 24

-8 12 -10 64.2974 85.6805 17

-8 -12 -11 63.5825 61.3495 21

-8 12 -11 13.2528 49.8611 24

-8 12 -11 116.097 81.9498 17

-8 -12 -11-2.92058 59.1261 18

-8 12 -12 337.887 61.0457 24

-8 -12 -12 389.503 65.5789 18

-8 12 -12 457.643 85.2865 17

-8 12 -13-20.5839 46.8328 24

-8 -12 -13 49.3164 46.0571 18

-8 12 -14 91.1173 41.9452 24

8 -14 -14 45.6714 52.3914 26

8 -14 -13 18.1383 59.6824 26

8 -14 -12 696.453 85.9477 20

8 14 -12 698.454 55.3187 22

8 -14 -12 690.827 83.4502 26

8 -14 -11 39.0409 63.6648 20

8 -14 -11 5.77239 67.3907 26

8 -14 -10 300.103 82.9268 25

8 -14 -10 457.084 81.6241 26

8 -14 -9 341.070 81.4362 26

-8 14 9 366.777 55.2896 16

8 -14 -9 378.297 94.4547 25

8 -14 -8 1179.45 107.660 20

-8 14 8 1479.34 98.7415 16

8 -14 -8 1316.70 134.644 25

8 -14 -8 1384.95 118.291 26

8 -14 -7 179.960 67.2804 20

8 -14 -7 99.0343 76.0884 26

-8 14 7 163.203 53.0736 16

8 -14 -7 216.999 100.202 25

8 -14 -6 285.989 68.6472 20

8 -14 -6 442.085 85.1713 26

8 -14 -6 202.119 104.664 25

-8 14 6 419.368 65.4846 16

-8 14 5 777.644 81.3776 16

8 -14 -5 670.833 81.2712 20

-8 14 4 2387.16 145.382 16

-8 -14 4 1939.12 137.842 21

8 -14 -4 2263.71 144.072 20

-8 14 3-21.7151 57.4881 16

-8 -14 3 50.7007 59.7587 21

8 -14 -3 35.6035 55.6319 20

-8 14 2 140.709 62.3386 16

-8 -14 2 117.939 66.7009 21

8 -14 -2 158.063 58.0277 20

-8 14 1 935.706 93.9306 16

8 -14 -1 845.410 82.4851 20

-8 -14 0 918.861 101.390 21

8 -14 0 1052.50 90.7118 20

-8 -14 -1 131.804 72.0376 21

-8 -14 -1 44.7744 60.9333 18

-8 -14 -2 505.861 77.6293 18

-8 -14 -2 515.301 85.7831 21

-8 14 -2 480.134 88.7336 16

-8 -14 -3 10.2732 63.1795 18

-8 -14 -3 44.1199 68.5387 21

-8 -14 -4 48.1701 62.8986 18

-8 -14 -4-32.5940 70.9769 21

-8 -14 -5 20.4121 65.1652 18

-8 -14 -5 38.9452 74.4584 21

-8 -14 -6 108.784 66.3321 18

-8 -14 -6 118.842 72.8875 21

-8 -14 -7 78.4075 62.4588 18

-8 14 -8 108.916 44.3249 24

-8 -14 -8 44.0011 59.9730 18

-8 -14 -8 137.460 66.2268 21

-8 14 -9 193.160 48.5847 24

-8 -14 -9 148.434 61.2746 18

-8 -14 -9 193.140 63.7338 21

-8 14 -10-44.9100 44.1558 24

-8 -14 -10-55.7757 52.1488 18

-8 -14 -10 45.5510 54.0229 21

8 -16 -9 194.330 47.9500 26

8 -16 -8 105.052 49.4196 26

8 -16 -7 244.074 60.6561 25

8 -16 -7 248.604 54.9376 26

8 -16 -7 334.185 66.2729 20

8 -16 -6 53.5952 65.8532 25

8 -16 -6 23.6504 49.4293 26

8 -16 -6 123.624 63.7177 20

-8 16 6 33.7686 51.3194 16

8 -16 -5 211.606 74.3594 25

8 -16 -5 200.302 55.6067 26

8 -16 -5 183.405 63.1500 20

-8 16 5 221.185 60.5580 16

8 -16 -4-13.6102 74.6948 25

8 -16 -4 40.3152 49.0244 26

8 -16 -4 41.1194 60.4334 20

-8 16 4-34.8568 57.6683 16

8 -16 -3 48.4845 60.6659 20

-8 16 3 45.5590 60.4789 16

8 -16 -3 88.4613 78.6996 25

-8 16 2 8.96115 64.3915 16

8 -16 -2 35.5090 58.8190 20

-8 -16 1 185.738 54.3586 21

-8 -16 0-2.40255 54.8719 21

-8 -16 -1 47.5235 57.2229 21

-8 -16 -2 57.9535 59.2146 21

-8 -16 -3 67.2897 56.8361 21

-8 -16 -4 101.688 57.2403 21

-8 -16 -5 55.1785 54.8756 21

9 1 -20 62.1072 50.3466 8

9 -1 -20 250.046 77.0016 7

9 1 -19 1629.37 109.537 8

9 -1 -19 1428.15 122.823 7

9 -1 -19 1437.75 127.952 9

9 -1 -19 1531.28 160.218 28

9 1 -19 1116.31 175.195 23

9 1 -18 15576.9 678.468 8

9 -1 -18 14551.6 694.375 9

9 -1 -18 15062.5 691.849 7

9 -1 -18 11253.5 706.701 28

9 1 -18 11243.9 728.087 23

9 1 -17 4639.90 355.583 23

9 1 -17 6013.82 285.502 8

9 -1 -17 5560.06 301.609 9

9 -1 -17 5485.79 298.575 7

9 -1 -17 6058.72 324.660 27

9 1 -17 4594.93 324.368 28

9 -1 -17 5063.07 333.125 28

9 1 -17 5171.46 281.383 22

9 -1 -16 877.927 150.922 4

9 -1 -16 1062.43 109.590 9

9 1 -16 805.799 166.565 23

9 1 -16 1099.34 87.5312 8

9 1 -16 1143.43 129.827 27

9 1 -16 786.282 139.377 28

9 -1 -16 1110.97 128.061 27

9 1 -16 935.373 86.6969 22

9 -1 -16 1254.40 110.831 7

9 -1 -15 2922.28 182.714 7

9 -1 -15 2838.92 187.508 9

9 1 -15 2886.71 176.387 6

9 1 -15 3146.78 169.561 8

9 -1 -15 2730.55 241.801 4

9 -1 -15 3337.51 209.289 27

9 1 -15 3547.05 210.436 27

9 1 -15 2829.36 221.605 28

9 1 -15 2608.46 166.619 22

9 1 -15 2509.13 248.492 23

9 -1 -15 2980.11 240.717 23

9 -1 -14 22950.6 1065.06 7

9 -1 -14 22892.1 1069.38 9

9 1 -14 25342.2 1062.82 6

9 1 -14 25599.7 1057.44 8

9 -1 -14 22758.1 1131.44 4

9 -1 -14 22579.2 1079.31 27

9 1 -14 24100.8 1082.38 27

9 1 -14 21200.9 1051.81 22

9 -1 -14 20614.9 1052.91 22

9 1 -14 16460.7 1094.90 23

9 -1 -14 18866.6 1097.74 23

9 -1 -13 14974.1 727.850 7

9 -1 -13 15782.4 732.384 9

9 1 -13 15903.8 724.488 6

9 1 -13 16846.7 721.412 8

9 -1 -13 15129.3 789.784 4

9 1 -13 15808.7 743.069 27

9 -1 -13 16676.4 745.263 27

9 1 -13 14548.7 716.693 22

9 -1 -13 14047.7 719.152 22

9 -1 -13 13543.8 764.175 23

9 -1 -12 2375.21 146.214 7

9 1 -12 1712.70 138.382 29

9 1 -12 2679.69 166.269 27

9 -1 -12 2582.42 165.217 27

9 1 -12 1497.23 179.981 23

9 -1 -12 2359.32 150.420 9

9 -1 -12 2158.89 136.497 22

9 1 -12 2529.63 136.190 22

9 -1 -12 2469.49 198.891 4

9 1 -12 2750.46 143.486 6

9 1 -12 2892.76 138.978 8

9 -1 -11 53.5261 33.9013 8

9 1 -11 89.6751 34.8263 8

9 -1 -11 41.3045 44.7610 7

9 1 -11 89.7727 59.7173 27

9 -1 -11 41.8043 65.7458 27

9 -1 -11 61.0241 53.5588 9

9 1 -11 45.1404 93.7964 23

9 1 -11 58.9333 41.7741 6

9 -1 -11 14.1366 86.2486 4

9 1 -11 51.9597 31.9034 22

9 -1 -10 57254.4 2572.63 19

9 1 -10 54140.9 2569.98 6

9 -1 -10 55731.6 2622.24 4

9 1 -9 32190.6 1623.48 6

9 -1 -9 33655.4 1669.48 4

9 1 -8 23604.8 1227.52 6

9 -1 -8 24595.9 1270.60 4

9 -1 -7 6203.89 313.674 6

9 1 -7 6476.37 314.218 6

9 1 -7 6808.74 353.452 3

9 -1 -7 6826.73 360.447 4

9 -1 -6 2066.50 164.456 4

9 1 -6 2485.21 162.350 3

9 -1 -6 2090.94 168.663 12

9 1 -5 4885.22 291.954 3

9 -1 -5 4746.69 302.616 12

9 1 -4-21.6931 63.3946 3

9 1 -4 46.4260 65.9401 12

9 -1 -4 26.3403 76.7580 12

9 1 -3 30626.8 1473.50 3

9 -1 -3 32087.4 1489.29 12

9 1 -3 28852.6 1469.92 12

9 -1 -3 34317.8 1498.07 11

9 -1 -2 32975.9 1464.52 12

9 1 -2 28716.4 1442.42 12

9 1 -2 30425.9 1444.73 3

9 1 -1 3657.19 209.223 3

9 1 -1 3376.75 207.654 12

9 -1 -1 3182.82 215.777 12

9 1 0 933.577 108.270 12

9 -1 0 1078.82 128.275 11

9 -1 1 64923.6 3040.24 11

9 -1 2 185668. 8564.97 11

9 -1 3 14968.9 751.646 11

9 -1 4 55243.0 2329.15 11

-9 -1 -4 43356.7 2330.02 1

9 -1 5 30398.0 1373.50 11

-9 1 -5 29226.2 1343.33 14

-9 1 -6 41629.9 1850.06 14

-9 -1 -6 37613.4 1902.43 1

-9 -1 -6 36891.2 1891.71 2

-9 1 -7 12242.3 534.866 14

-9 -1 -7 11421.4 595.127 2

-9 1 -7 10332.3 534.096 17

-9 -1 -7 9712.76 527.188 17

-9 -1 -7 11247.6 600.773 1

-9 1 -8 4912.50 294.317 17

-9 -1 -8 4773.60 289.988 17

-9 -1 -8 5453.03 276.514 10

-9 1 -8 6057.60 278.743 10

-9 -1 -8 5716.92 356.113 2

-9 -1 -8 5592.26 367.555 1

-9 -1 -9 2295.90 232.144 1

-9 1 -9 2378.66 131.578 10

-9 1 -9 1829.91 155.939 17

-9 -1 -9 1997.58 150.756 17

-9 -1 -9 2359.26 218.928 2

-9 -1 -9 1745.13 142.988 28

-9 -1 -10 17046.8 873.614 1

-9 1 -10 17581.0 771.229 10

-9 1 -10 17771.6 771.340 8

-9 1 -10 14777.9 801.043 5

-9 1 -10 15592.8 791.599 17

-9 -1 -10 15885.8 786.933 17

-9 -1 -10 16020.8 856.287 2

-9 -1 -10 13450.4 778.647 28

-9 -1 -11 4994.11 363.789 1

-9 1 -11 5489.05 266.122 10

-9 1 -11 5368.95 265.309 8

-9 1 -11 4623.95 296.289 5

-9 1 -11 4890.29 295.391 17

-9 -1 -11 5315.38 291.789 17

-9 -1 -11 4799.32 361.876 2

-9 -1 -11 4006.42 278.900 28

-9 1 -12 2636.46 159.407 10

-9 1 -12 2699.84 157.454 8

-9 1 -12 2048.32 187.401 5

-9 1 -12 2633.22 195.276 17

-9 -1 -12 2969.62 190.624 17

-9 -1 -12 2424.77 259.305 1

-9 -1 -12 2318.11 258.534 2

-9 -1 -13 140.502 95.5331 17

-9 1 -13 238.592 109.653 17

-9 1 -13 195.110 60.4697 8

-9 1 -13 143.446 81.0864 5

-9 1 -13 197.385 62.2085 10

-9 -1 -13 190.873 155.311 2

-9 1 -14 814.686 129.492 17

-9 -1 -14 838.502 122.825 17

-9 1 -14 919.023 84.7649 8

-9 1 -14 751.847 111.988 5

-9 -1 -14 705.637 176.332 2

-9 1 -14 813.727 85.4624 10

-9 1 -15 816.779 81.1645 8

-9 1 -15 802.647 127.733 17

-9 -1 -15 1034.89 125.662 17

-9 1 -15 942.005 85.5028 10

-9 1 -16 86.4162 53.2411 8

-9 1 -16 196.392 100.771 17

-9 1 -16 145.213 53.4340 10

-9 1 -17 216.753 49.5074 8

-9 1 -17 226.628 91.2189 17

-9 1 -17 229.225 50.6564 10

-9 1 -18 35.5763 40.8679 8

-9 1 -19 104.921 38.6602 8

-9 1 -20 178.124 31.0660 8

9 -3 -21 401.013 109.045 23

9 -3 -20 334.085 108.848 28

9 -3 -20 464.351 129.616 23

9 -3 -19 493.339 126.452 28

9 -3 -19 661.624 84.7535 7

9 3 -19 376.596 79.9102 7

9 -3 -19 419.480 137.890 23

9 -3 -19 407.048 61.4291 8

9 3 -19 434.178 60.8482 8

9 3 -18 864.717 103.399 9

9 -3 -18 1040.29 105.064 9

9 -3 -18 833.549 77.4921 8

9 3 -18 883.731 80.1446 8

9 -3 -18 1020.14 163.405 23

9 -3 -18 987.282 144.887 28

9 -3 -18 794.157 95.7262 7

9 3 -18 787.746 98.8762 7

9 3 -18 853.698 80.9332 22

9 3 -17 2399.90 174.396 9

9 -3 -17 2791.05 181.251 9

9 -3 -17 2826.52 159.312 8

9 3 -17 2785.64 158.821 8

9 -3 -17 2402.46 224.514 23

9 -3 -17 2824.07 199.341 27

9 -3 -17 2829.73 175.469 7

9 3 -17 2820.71 177.506 7

9 3 -17 2481.73 157.055 22

9 -3 -16 22.7797 66.4901 7

9 3 -16 19.5068 66.6005 7

9 3 -16-71.3178 155.073 23

9 -3 -16 53.3745 75.5144 9

9 3 -16-12.6051 73.8275 9

9 3 -16 59.2434 53.1309 8

9 -3 -16-13.2333 49.1931 8

9 3 -16-51.3549 98.8989 4

9 -3 -16 59.9823 96.7888 4

9 -3 -16 88.3329 91.1462 27

9 3 -16 22.4152 50.9914 22

9 3 -15 4884.54 292.731 27

9 -3 -15 4887.98 290.891 27

9 3 -15 4752.99 275.992 9

9 -3 -15 5176.35 276.213 9

9 -3 -15 5106.63 259.990 8

9 3 -15 5382.73 261.377 8

9 3 -15 4163.54 322.897 4

9 -3 -15 5068.37 338.736 4

9 3 -15 4765.52 271.263 7

9 -3 -15 5207.23 274.668 7

9 3 -15 4948.30 259.471 22

9 3 -14 912.886 78.0129 22

9 -3 -14 916.122 95.1257 7

9 3 -14 823.889 92.3934 7

9 3 -14 893.581 138.186 4

9 -3 -14 769.445 135.083 4

9 -3 -14 873.935 85.0008 6

9 3 -14 905.331 87.8655 6

9 3 -14 772.424 163.760 23

9 3 -14 983.169 102.035 9

9 -3 -14 880.571 92.4713 9

9 -3 -14 828.043 94.3880 26

9 -3 -14 977.176 77.8663 8

9 3 -14 952.051 79.9821 8

9 -3 -14 1103.11 114.396 27

9 3 -14 1262.43 121.494 27

9 -3 -13 28421.9 1263.14 8

9 3 -13 29755.6 1265.21 8

9 3 -13 26010.7 1335.55 4

9 -3 -13 27679.5 1338.03 4

9 3 -13 28265.6 1288.82 27

9 3 -13 22838.3 1267.88 29

9 -3 -13 30532.4 1292.60 27

9 3 -13 29316.8 1270.41 6

9 -3 -13 28167.9 1268.34 6

9 -3 -13 23838.0 1268.98 26

9 -3 -13 28520.2 1278.07 9

9 3 -13 27450.5 1277.70 9

9 -3 -13 28191.5 1274.71 7

9 3 -13 26212.3 1273.05 7

9 3 -13 27750.3 1263.46 22

9 -3 -12 2601.80 134.936 8

9 3 -12 2467.58 134.642 8

9 3 -12 2064.61 191.874 4

9 -3 -12 2277.80 193.433 4

9 3 -12 2280.50 145.892 7

9 -3 -12 2342.23 143.451 7

9 3 -12 2184.88 148.843 29

9 3 -12 2164.19 157.547 27

9 -3 -12 2391.88 158.716 27

9 -3 -12 2372.94 139.783 6

9 3 -12 2348.91 139.769 6

9 -3 -12 2088.70 142.721 26

9 -3 -12 2412.28 148.443 9

9 3 -12 2371.45 147.551 9

9 3 -12 2437.30 134.723 22

9 3 -11 795.132 62.1839 22

9 -3 -11 806.038 61.9082 8

9 3 -11 810.549 63.0272 8

9 -3 -11 678.676 88.8292 20

9 -3 -11 772.870 78.9745 19

9 -3 -11 745.816 72.1632 7

9 3 -11 630.790 71.8232 7

9 -3 -11 799.583 114.195 4

9 3 -11 505.950 111.245 4

9 3 -11 624.875 64.8053 6

9 -3 -11 650.810 64.8622 6

9 3 -11 332.247 122.117 23

9 -3 -11 742.312 75.6231 26

9 -3 -11 809.605 113.695 25

9 3 -11 749.810 78.5528 9

9 -3 -11 807.575 79.5009 9

9 3 -11 819.133 87.3493 29

9 3 -11 767.487 88.0753 27

9 -3 -11 974.140 94.4773 27

9 -3 -10 1544.01 107.841 7

9 3 -10 1471.91 108.957 7

9 3 -10 1417.61 109.847 9

9 3 -10 1602.95 103.693 6

9 -3 -10 1506.42 102.493 6

9 -3 -10 1505.87 123.397 27

9 -3 -10 1489.79 137.074 25

9 3 -10 1489.73 97.9243 22

9 -3 -10 1310.37 117.449 20

9 3 -10 1479.64 152.852 4

9 -3 -10 1582.31 151.081 4

9 3 -10 1921.26 102.162 8

9 -3 -9 23836.3 1005.85 11

9 3 -9 20177.6 966.072 6

9 -3 -9 20328.8 965.778 6

9 3 -9 19277.7 1010.50 4

9 -3 -9 21414.7 1015.42 4

9 -3 -9 21157.7 987.993 19

9 -3 -9 20032.9 976.579 20

9 -3 -8 16060.3 895.261 19

9 -3 -8 20170.5 888.896 20

9 -3 -8 21083.8 932.668 11

9 3 -8 16742.4 883.142 13

9 3 -8 19437.4 879.832 6

9 -3 -8 18977.5 878.895 6

9 3 -8 19330.5 926.102 4

9 -3 -8 20845.7 929.018 4

9 3 -7 15536.3 774.646 13

9 -3 -7 14223.1 811.748 11

9 -3 -7 15864.1 761.734 6

9 3 -7 16391.7 762.831 6

9 -3 -7 17247.5 771.243 20

9 3 -7 16868.9 807.580 4

9 -3 -7 17598.0 808.520 4

9 -3 -6 2137.14 129.954 20

9 3 -6 2388.20 160.643 3

9 -3 -6 2482.03 161.801 3

9 -3 -6 1970.73 176.296 11

9 -3 -6 2547.34 169.367 4

9 3 -6 2134.66 163.138 4

9 3 -5 24255.4 1099.93 3

9 -3 -5 22857.7 1095.29 3

9 -3 -5 21404.4 1107.47 11

9 3 -4 146544. 6667.46 3

9 -3 -4 145317. 6666.42 3

9 -3 -4 141570. 6672.69 11

9 3 -3 1923.03 138.121 3

9 -3 -3 1715.58 130.532 3

9 3 -2 149.074 69.1134 3

9 -3 -2 128.358 68.8322 3

9 3 -2 215.339 54.0418 12

9 -3 -2 81.5542 107.635 12

9 -3 -1 30086.1 1508.30 11

9 3 -1 37077.9 1520.21 3

9 -3 -1 34381.5 1515.40 3

9 3 -1 29058.4 1490.98 12

9 -3 0 58979.9 2732.19 11

9 3 0 58201.0 2712.69 12

9 -3 1 5055.16 308.345 11

9 3 1 5843.38 294.330 12

9 -3 2 508.942 103.154 11

9 3 2 846.209 82.7861 12

-9 3 -3 3823.60 254.908 1

-9 -3 -3 4387.31 261.062 1

-9 3 -4 11987.5 598.216 1

-9 -3 -4 13315.6 606.568 1

-9 -3 -5 8112.30 419.251 2

-9 3 -5 7339.35 411.283 2

-9 3 -5 7456.37 423.033 1

-9 -3 -5 8021.80 429.817 1

-9 3 -5 7463.66 373.724 14

-9 3 -6 6164.94 388.142 2

-9 -3 -6 5916.78 387.344 2

9 -3 6 7207.75 394.323 11

-9 3 -6 7838.51 357.068 14

-9 -3 -6 6490.86 402.173 1

-9 3 -6 6052.26 399.661 1

-9 -3 -7 24210.6 1363.63 21

-9 3 -7 37165.8 1392.87 14

9 -3 7 36713.1 1426.53 11

-9 -3 -7 32175.8 1453.44 1

-9 3 -7 29060.5 1444.51 1

-9 3 -7 28691.1 1434.30 2

-9 -3 -7 31296.5 1443.25 2

-9 3 -8 986.724 99.8745 14

-9 -3 -8 909.972 75.5545 10

-9 3 -8 985.639 74.7718 10

-9 3 -8 709.310 112.797 17

-9 3 -8 943.121 147.025 2

-9 -3 -8 895.229 150.218 2

-9 3 -8 739.957 153.048 1

-9 -3 -8 1094.39 160.391 1

-9 -3 -9 924.411 76.7053 10

-9 3 -9 1016.11 76.5329 10

-9 3 -9 813.441 115.669 17

-9 3 -9 1006.59 97.1492 14

-9 -3 -9 586.897 86.2592 28

-9 3 -9 961.889 156.256 1

-9 -3 -9 844.222 154.181 1

-9 -3 -9 844.354 148.331 2

-9 3 -9 990.076 155.065 2

-9 3 -10 1615.69 196.973 2

-9 -3 -10 1519.04 196.002 2

-9 3 -10 1888.11 116.167 10

-9 -3 -10 1814.06 114.080 10

-9 3 -10 1586.98 158.906 17

-9 3 -10 1717.23 143.477 5

-9 -3 -10 1532.23 138.856 5

-9 -3 -10 1354.08 128.229 28

-9 3 -10 1812.39 214.318 1

-9 -3 -10 1770.08 211.623 1

-9 3 -10 1874.27 114.598 8

-9 -3 -10 1667.57 112.184 8

-9 3 -11 2964.50 267.010 2

-9 -3 -11 3277.52 276.808 2

-9 -3 -11 3365.89 180.655 10

-9 3 -11 3439.84 180.134 10

-9 3 -11 2835.74 207.402 5

-9 -3 -11 2660.99 204.830 5

-9 3 -11 2991.10 219.338 17

-9 3 -11 3207.90 177.008 8

-9 -3 -11 3320.70 178.001 8

-9 3 -11 2531.47 267.167 1

-9 -3 -11 2457.96 270.712 1

-9 -3 -11 2817.23 193.002 28

-9 3 -12 184.491 52.6355 10

-9 -3 -12 118.417 54.2449 10

-9 3 -12 83.3280 69.2904 5

-9 -3 -12 168.072 72.3340 5

-9 3 -12 130.111 111.322 17

-9 3 -12 133.322 50.4734 8

-9 -3 -12 203.024 53.3889 8

-9 3 -12-25.0935 137.698 2

-9 -3 -12 100.673 138.690 2

-9 -3 -12-29.5571 80.5248 28

-9 -3 -13 76.5462 52.2202 10

-9 3 -13 79.4690 51.9521 10

-9 3 -13 7.25160 72.1375 5

-9 -3 -13 51.0592 74.0394 5

-9 3 -13 121.650 112.350 17

-9 3 -13 113.630 52.1780 8

-9 -3 -13 75.3232 52.4459 8

-9 -3 -13-26.4247 83.3256 28

-9 3 -13-104.393 151.921 2

-9 -3 -13-183.069 160.591 2

-9 3 -14 401.529 63.6688 10

-9 -3 -14 438.302 63.7638 10

-9 3 -14 313.449 86.0022 5

-9 -3 -14 322.565 84.5509 5

-9 3 -14 531.936 66.0916 8

-9 -3 -14 354.046 61.2394 8

-9 3 -14 384.356 122.295 17

-9 -3 -14 283.898 66.7802 15

-9 3 -14 421.552 160.512 2

-9 -3 -14 197.784 146.833 2

-9 3 -15 890.258 83.3587 8

-9 -3 -15 1018.73 83.6892 8

-9 3 -15 1050.66 137.349 17

-9 3 -15 987.244 85.9050 10

-9 -3 -15 1010.36 84.6036 10

-9 3 -16 1034.68 84.0843 8

-9 -3 -16 1007.26 81.9732 8

-9 3 -16 1170.90 136.018 17

-9 -3 -16 975.554 81.9443 10

-9 3 -16 1111.22 85.5806 10

-9 3 -17 35.8354 40.7366 8

-9 -3 -17 12.5898 40.8259 8

-9 3 -17 147.004 91.2350 17

-9 3 -18 10.5647 37.1577 8

-9 -3 -18 20.9365 38.1831 8

-9 3 -18-3.32901 77.1396 17

-9 3 -19 55.2571 33.3585 8

-9 -3 -19 97.1850 32.2568 8

9 -5 -21 224.722 88.4978 23

9 -5 -21 130.212 80.9124 28

9 -5 -20 143.553 103.285 23

9 -5 -20 93.2008 95.6675 28

9 -5 -19 746.616 127.584 23

9 -5 -19 871.159 130.541 28

9 5 -19 829.627 76.7220 22

9 -5 -18 71.1629 65.9176 7

9 5 -18 82.7225 65.8761 7

9 5 -18 27.0611 50.7254 22

9 -5 -18 81.3864 124.015 23

9 -5 -17 575.814 141.142 23

9 5 -17 560.380 63.4748 8

9 -5 -17 504.422 60.3715 8

9 -5 -17 555.004 79.7912 7

9 5 -17 493.448 80.6018 7

9 5 -17 577.620 90.1240 9

9 -5 -17 367.151 82.5696 9

9 5 -17 513.469 69.3908 22

9 -5 -16 2372.51 160.561 26

9 -5 -16 2590.55 159.512 9

9 5 -16 2019.02 154.099 9

9 -5 -16 2534.13 216.538 23

9 -5 -16 2906.97 182.782 27

9 5 -16 2079.23 152.977 7

9 -5 -16 2265.11 151.825 7

9 -5 -16 2018.59 134.161 8

9 5 -16 2128.40 135.290 8

9 5 -16 2376.78 143.327 22

9 5 -15 1617.29 136.188 9

9 -5 -15 1938.04 139.143 9

9 -5 -15 1646.41 150.155 27

9 -5 -15 1486.65 136.871 26

9 5 -15 1851.06 137.395 7

9 -5 -15 1987.21 136.489 7

9 5 -15 2034.91 120.508 8

9 -5 -15 2047.54 120.012 8

9 5 -15 1870.01 122.077 22

9 -5 -14 544.013 60.3795 8

9 5 -14 474.456 58.0945 8

9 5 -14 436.805 117.878 4

9 -5 -14 459.886 118.613 4

9 -5 -14 383.681 85.6410 26

9 5 -14 427.158 77.0497 9

9 -5 -14 451.818 78.5977 9

9 -5 -14 554.468 77.6294 7

9 5 -14 434.476 77.4625 7

9 5 -14 550.613 66.9946 22

9 5 -14 366.750 70.5635 29

9 -5 -14 669.880 102.255 27

9 -5 -13 26.1256 77.9951 20

9 -5 -13 121.048 43.5593 8

9 5 -13 125.190 42.7543 8

9 5 -13 131.682 52.6540 6

9 -5 -13 110.393 49.8949 6

9 5 -13 133.072 63.5629 7

9 -5 -13 60.1220 58.2541 7

9 5 -13 170.446 101.420 4

9 -5 -13 128.021 96.1221 4

9 -5 -13 61.4989 69.3581 26

9 -5 -13 123.093 62.6126 9

9 5 -13 107.233 63.0989 9

9 5 -13 443.492 56.0879 22

9 -5 -13 73.4496 78.8559 27

9 5 -13 102.651 81.6658 27

9 5 -13 135.315 71.2674 29

9 5 -12 162.289 57.1395 7

9 -5 -12 84.5969 53.9864 7

9 -5 -12 87.5091 90.3346 4

9 5 -12 108.535 93.2728 4

9 -5 -12 141.088 39.0644 8

9 5 -12 199.290 42.7152 8

9 5 -12 194.317 51.3989 6

9 -5 -12 87.1939 46.6875 6

9 5 -12 122.330 76.4017 27

9 5 -12 209.713 72.4569 29

9 -5 -12 185.427 70.6185 19

9 -5 -12 76.1501 68.4736 20

9 5 -12 86.0254 60.4136 9

9 -5 -12 101.379 59.5280 9

9 -5 -12 44.9761 103.104 25

9 -5 -12 84.6339 72.5623 27

9 -5 -12 102.177 62.6452 26

9 5 -11 2642.94 181.027 27

9 -5 -11 2919.91 185.411 27

9 -5 -11 2683.15 177.455 20

9 -5 -11 2550.75 187.997 19

9 5 -11 2841.51 173.177 9

9 -5 -11 3000.04 174.449 9

9 5 -11 3135.08 225.561 4

9 -5 -11 2969.13 217.976 4

9 5 -11 3109.85 166.429 24

9 5 -11 2789.85 170.382 7

9 -5 -11 3065.93 171.418 7

9 -5 -11 3200.98 162.173 8

9 5 -11 2998.17 161.111 8

9 -5 -11 3456.54 168.253 6

9 5 -11 3537.41 171.057 6

9 -5 -10 1401.60 110.671 27

9 5 -10 1288.59 95.6511 7

9 -5 -10 1454.51 96.5475 7

9 -5 -10 1334.63 86.3961 8

9 5 -10 1395.00 87.5111 8

9 5 -10 1464.67 142.825 4

9 -5 -10 1570.12 142.940 4

9 -5 -10 1384.07 90.2866 6

9 5 -10 1437.86 90.5753 6

9 5 -10 1412.43 93.6502 24

9 -5 -10 1303.67 103.479 20

9 -5 -10 975.532 119.129 19

9 -5 -10 1394.21 99.0438 9

9 5 -10 1200.41 98.7363 9

9 5 -9 4480.52 280.791 13

9 -5 -9 3930.66 317.171 11

9 -5 -9 5837.75 290.748 27

9 -5 -9 5418.64 268.996 6

9 5 -9 5707.23 270.517 6

9 -5 -9 5699.40 280.351 20

9 5 -9 5751.74 273.520 24

9 5 -9 5171.55 316.081 4

9 -5 -9 5182.16 313.274 4

9 5 -8 193.525 51.5143 13

9 -5 -8 296.363 38.8793 6

9 5 -8 221.522 39.7642 6

9 -5 -8 212.568 73.5626 4

9 5 -8 290.577 81.4090 4

9 -5 -8 313.053 56.0572 20

9 5 -8 313.198 43.4069 24

9 -5 -8 211.919 83.1463 11

9 -5 -7 25298.3 1233.11 6

9 5 -7 27629.8 1235.46 6

9 5 -7 25985.2 1275.20 4

9 -5 -7 26812.5 1274.91 4

9 -5 -7 25702.8 1273.42 11

9 -5 -7 29571.0 1244.25 20

9 5 -7 23971.5 1259.72 13

9 -5 -7 25872.5 1289.41 12

9 -5 -6 3422.00 177.308 6

9 5 -6 3596.35 179.286 6

9 5 -6 3145.16 208.191 13

9 -5 -6 3802.61 220.447 4

9 5 -6 3625.97 219.381 4

9 -5 -6 3519.74 245.268 12

9 -5 -6 3330.46 213.355 11

9 -5 -5 30597.9 1435.03 11

9 -5 -5 28703.5 1465.35 12

9 -5 -5 29464.3 1433.23 3

9 5 -5 32647.9 1440.03 3

9 5 -5 28915.9 1439.43 4

9 -5 -5 30985.8 1441.54 4

9 -5 -4 17247.9 851.426 12

9 -5 -4 16785.1 806.021 11

-9 5 4 16252.6 774.395 16

9 5 -4 16663.5 804.902 3

9 -5 -4 17194.6 814.097 4

9 -5 -4 15397.6 799.699 3

9 5 -4 15460.5 809.881 4

9 -5 -4 17569.1 780.799 20

9 -5 -3 18341.8 856.080 11

-9 5 3 17529.0 826.395 16

9 5 -3 17234.3 856.777 3

9 -5 -3 15499.8 849.481 3

9 -5 -3 18351.9 830.560 20

9 -5 -2 1044.68 158.690 12

-9 5 2 1093.90 71.0786 16

9 5 -2 1286.00 114.771 3

9 -5 -2 986.465 99.9503 3

9 -5 -2 871.874 71.1807 20

9 -5 -1 84853.6 3809.84 11

-9 5 1 82548.8 3783.00 16

9 5 -1 89378.8 3820.76 3

9 -5 -1 77823.7 3810.26 3

9 -5 -1 80377.0 3782.39 20

9 -5 0 21922.6 1051.46 11

-9 5 0 20358.0 1024.59 16

9 -5 0 23215.2 1061.03 3

9 -5 1 61311.0 2682.76 11

-9 5 -1 55888.8 2655.67 16

-9 5 -2 68.8137 85.3309 1

-9 -5 -2 5.61825 86.9971 1

9 -5 2 106.243 55.2027 11

-9 5 -3 7883.10 459.095 1

-9 -5 -3 9141.17 467.087 1

9 -5 3 8453.39 444.351 11

9 -5 4 17202.5 882.347 11

-9 5 -4 17122.0 896.773 1

-9 -5 -4 19903.9 905.852 1

-9 -5 -4 19254.9 896.359 2

-9 5 -4 16922.8 887.887 2

9 -5 5 5092.15 323.891 11

-9 -5 -5 6158.58 341.001 1

-9 5 -5 5317.17 332.233 1

-9 -5 -5 5486.75 328.255 2

-9 5 -5 5529.99 328.495 2

-9 5 -6 998.628 139.040 1

-9 -5 -6 974.385 129.471 2

-9 -5 -6 1060.19 136.670 1

-9 5 -6 610.782 117.883 2

9 -5 6 642.362 119.663 11

-9 -5 -7 153.570 57.1191 21

9 -5 7 73.8779 106.923 11

-9 -5 -7 265.023 41.8782 10

-9 5 -7 258.931 42.3454 10

-9 5 -7 305.038 109.069 2

-9 -5 -7 403.668 113.190 2

-9 5 -7 202.040 107.300 1

-9 -5 -7 147.166 106.181 1

-9 5 -7 106.298 75.8048 14

-9 5 -8 2264.22 147.730 14

-9 5 -8 1531.52 174.062 2

-9 -5 -8 1812.04 185.248 2

-9 5 -8 1772.70 190.190 1

-9 -5 -8 1838.47 190.024 1

-9 -5 -8 1516.93 118.437 21

-9 5 -8 1723.86 135.996 5

-9 -5 -8 1640.78 134.317 5

-9 5 -8 1546.96 160.529 17

-9 5 -8 2124.87 116.105 10

-9 -5 -8 1883.39 113.927 10

-9 5 -8 2091.04 114.633 8

-9 5 -9 698.262 60.2393 8

-9 -5 -9 571.954 59.2397 8

-9 5 -9 485.453 76.3762 5

-9 -5 -9 470.010 77.7973 5

-9 5 -9 567.012 131.075 1

-9 -5 -9 613.826 141.531 1

-9 5 -9 515.979 131.651 2

-9 -5 -9 349.500 119.037 2

-9 -5 -9 424.541 66.8683 21

-9 5 -9 481.737 124.100 17

-9 -5 -9 665.134 61.1911 10

-9 5 -9 697.859 63.2890 10

-9 5 -9 782.237 93.5596 14

-9 5 -10 2643.55 157.246 14

-9 5 -10 1763.94 216.784 2

-9 -5 -10 1978.13 219.656 2

-9 5 -10 2060.01 222.652 1

-9 -5 -10 2105.15 219.572 1

-9 -5 -10 1550.96 131.526 21
[truncated: 493,059 more chars]
