# Supplementary material for: Kinetic trapping of 2,4,6-tris(4-pyridyl)benzene and ZnI2 into M12L8 poly-[n]-catenanes using solution and solid-state processes
Source: Sci Rep. 2023 Apr 5;13:5605. doi: 10.1038/s41598-023-32661-x (PMC10076325; doi:10.1038/s41598-023-32661-x)
Supplement: Supplementary file 5 — Supplementary Information 5. [file 41598_2023_32661_MOESM5_ESM.docx]

data_TPB_ZnI2_NB_RT

_audit_creation_method 'SHELXL-2017/1'

_shelx_SHELXL_version_number '2017/1'

_chemical_name_systematic ?

_chemical_name_common ?

_chemical_melting_point ?

_chemical_formula_moiety ?

_chemical_formula_sum

'C204 H150 I24 N30 O12 Zn12'

_chemical_formula_weight 7043.57

loop_

_atom_type_symbol

_atom_type_description

_atom_type_scat_dispersion_real

_atom_type_scat_dispersion_imag

_atom_type_scat_source

'C' 'C' 0.0181 0.0091

'International Tables Vol C Tables 4.2.6.8 and 6.1.1.4'

'H' 'H' 0.0000 0.0000

'International Tables Vol C Tables 4.2.6.8 and 6.1.1.4'

'I' 'I' -0.3257 6.8362

'International Tables Vol C Tables 4.2.6.8 and 6.1.1.4'

'N' 'N' 0.0311 0.0180

'International Tables Vol C Tables 4.2.6.8 and 6.1.1.4'

'Zn' 'Zn' -1.5491 0.6778

'International Tables Vol C Tables 4.2.6.8 and 6.1.1.4'

'O' 'O' 0.0492 0.0322

'International Tables Vol C Tables 4.2.6.8 and 6.1.1.4'

_space_group_crystal_system trigonal

_space_group_IT_number 148

_space_group_name_H-M_alt 'R -3 :H'

_space_group_name_Hall '-R 3'

_shelx_space_group_comment

;

The symmetry employed for this shelxl refinement is uniquely defined

by the following loop, which should always be used as a source of

symmetry information in preference to the above space-group names.

They are only intended as comments.

;

loop_

_space_group_symop_operation_xyz

'x, y, z'

'-y, x-y, z'

'-x+y, -x, z'

'x+2/3, y+1/3, z+1/3'

'-y+2/3, x-y+1/3, z+1/3'

'-x+y+2/3, -x+1/3, z+1/3'

'x+1/3, y+2/3, z+2/3'

'-y+1/3, x-y+2/3, z+2/3'

'-x+y+1/3, -x+2/3, z+2/3'

'-x, -y, -z'

'y, -x+y, -z'

'x-y, x, -z'

'-x+2/3, -y+1/3, -z+1/3'

'y+2/3, -x+y+1/3, -z+1/3'

'x-y+2/3, x+1/3, -z+1/3'

'-x+1/3, -y+2/3, -z+2/3'

'y+1/3, -x+y+2/3, -z+2/3'

'x-y+1/3, x+2/3, -z+2/3'

_cell_length_a 38.6805(7)

_cell_length_b 38.6805(7)

_cell_length_c 16.0202(3)

_cell_angle_alpha 90

_cell_angle_beta 90

_cell_angle_gamma 120

_cell_volume 20757.9(8)

_cell_formula_units_Z 3

_cell_measurement_temperature 303(2)

_cell_measurement_reflns_used 15912

_cell_measurement_theta_min 3.06000

_cell_measurement_theta_max 69.476

_exptl_crystal_description "prism"

_exptl_crystal_colour "colorless"

_exptl_crystal_density_meas ?

_exptl_crystal_density_method ?

_exptl_crystal_density_diffrn 1.690

_exptl_crystal_F_000 9936

_exptl_transmission_factor_min ?

_exptl_transmission_factor_max ?

_exptl_crystal_size_max 0.100

_exptl_crystal_size_mid 0.080

_exptl_crystal_size_min 0.050

_exptl_absorpt_coefficient_mu 22.539

_shelx_estimated_absorpt_T_min 0.211

_shelx_estimated_absorpt_T_max 0.399

_exptl_absorpt_correction_type "cylinder"

_exptl_absorpt_correction_T_min 0.0323

_exptl_absorpt_correction_T_max 0.1295

_exptl_absorpt_process_details ?

_exptl_absorpt_special_details ?

_diffrn_ambient_temperature 303(2)

_diffrn_radiation_wavelength 1.54184

_diffrn_radiation_type CuK\a

_diffrn_source "micro-focus sealed X-ray tube"

_diffrn_measurement_device_type "PhotonJet (Cu) X-ray source Rigaku"

_diffrn_measurement_method ?

_diffrn_detector_area_resol_mean ?

_diffrn_reflns_number 42589

_diffrn_reflns_av_unetI/netI 0.0488

_diffrn_reflns_av_R_equivalents 0.0780

_diffrn_reflns_limit_h_min -42

_diffrn_reflns_limit_h_max 36

_diffrn_reflns_limit_k_min -46

_diffrn_reflns_limit_k_max 48

_diffrn_reflns_limit_l_min -19

_diffrn_reflns_limit_l_max 16

_diffrn_reflns_theta_min 3.058

_diffrn_reflns_theta_max 76.440

_diffrn_reflns_theta_full 67.684

_diffrn_measured_fraction_theta_max 0.934

_diffrn_measured_fraction_theta_full 0.996

_diffrn_reflns_Laue_measured_fraction_max 0.934

_diffrn_reflns_Laue_measured_fraction_full 0.996

_diffrn_reflns_point_group_measured_fraction_max 0.934

_diffrn_reflns_point_group_measured_fraction_full 0.996

_reflns_number_total 9032

_reflns_number_gt 6532

_reflns_threshold_expression 'I > 2\s(I)'

_reflns_Friedel_coverage 0.000

_reflns_Friedel_fraction_max .

_reflns_Friedel_fraction_full .

_reflns_special_details

;

Reflections were merged by SHELXL according to the crystal

class for the calculation of statistics and refinement.

_reflns_Friedel_fraction is defined as the number of unique

Friedel pairs measured divided by the number that would be

possible theoretically, ignoring centric projections and

systematic absences.

;

_computing_data_collection ?

_computing_cell_refinement ?

_computing_data_reduction ?

_computing_structure_solution ?

_computing_structure_refinement 'SHELXL-2017/1 (Sheldrick, 2017)'

_computing_molecular_graphics ?

_computing_publication_material ?

_refine_special_details ?

_refine_ls_structure_factor_coef Fsqd

_refine_ls_matrix_type full

_refine_ls_weighting_scheme calc

_refine_ls_weighting_details

'w=1/[\s^2^(Fo^2^)+(0.1811P)^2^] where P=(Fo^2^+2Fc^2^)/3'

_atom_sites_solution_primary ?

_atom_sites_solution_secondary ?

_atom_sites_solution_hydrogens geom

_refine_ls_hydrogen_treatment constr

_refine_ls_extinction_method none

_refine_ls_extinction_coef .

_refine_ls_number_reflns 9032

_refine_ls_number_parameters 386

_refine_ls_number_restraints 92

_refine_ls_R_factor_all 0.0887

_refine_ls_R_factor_gt 0.0753

_refine_ls_wR_factor_ref 0.2561

_refine_ls_wR_factor_gt 0.2362

_refine_ls_goodness_of_fit_ref 1.052

_refine_ls_restrained_S_all 1.300

_refine_ls_shift/su_max 0.001

_refine_ls_shift/su_mean 0.000

loop_

_atom_site_label

_atom_site_type_symbol

_atom_site_fract_x

_atom_site_fract_y

_atom_site_fract_z

_atom_site_U_iso_or_equiv

_atom_site_adp_type

_atom_site_occupancy

_atom_site_site_symmetry_order

_atom_site_calc_flag

_atom_site_refinement_flags_posn

_atom_site_refinement_flags_adp

_atom_site_refinement_flags_occupancy

_atom_site_disorder_assembly

_atom_site_disorder_group

I1 I -0.03609(2) 0.25864(2) 0.18543(4) 0.1051(3) Uani 1 1 d . . . . .

I2 I 0.11558(3) 0.68216(2) 0.73621(5) 0.1219(3) Uani 1 1 d . . . . .

I3 I 0.03778(4) 0.27114(3) 0.41019(4) 0.1376(4) Uani 1 1 d . . . . .

I5 I 0.07383(3) 0.57503(3) 0.89442(5) 0.1267(3) Uani 1 1 d . . . . .

Zn5 Zn 0.02504(3) 0.29967(3) 0.27829(6) 0.0807(3) Uani 1 1 d . . . . .

Zn6 Zn 0.11419(4) 0.61720(4) 0.77252(8) 0.0980(4) Uani 1 1 d . . . . .

N19 N 0.0268(2) 0.35265(19) 0.3021(4) 0.0814(14) Uani 1 1 d . . . . .

N25 N -0.0127(2) 0.57874(17) 0.1279(4) 0.0799(14) Uani 1 1 d . . . . .

N13 N 0.0985(2) 0.5838(2) 0.6647(5) 0.0944(19) Uani 1 1 d . . . . .

C22 C 0.0334(2) 0.4268(2) 0.3265(4) 0.0786(17) Uani 1 1 d . . . . .

C11 C 0.0371(3) 0.4665(2) 0.3419(5) 0.0862(19) Uani 1 1 d . . . . .

C23 C 0.0025(3) 0.3977(2) 0.2824(5) 0.0829(18) Uani 1 1 d . . . . .

H23 H -0.016909 0.402660 0.260273 0.100 Uiso 1 1 calc R U . . .

C28 C 0.0161(3) 0.5416(2) 0.2369(5) 0.0866(19) Uani 1 1 d . . . . .

C10 C 0.0265(3) 0.4849(2) 0.2823(5) 0.0799(17) Uani 1 1 d . . . . .

H10 H 0.016164 0.472173 0.231551 0.096 Uiso 1 1 calc R U . . .

C9 C 0.0309(3) 0.5228(2) 0.2969(5) 0.0840(18) Uani 1 1 d . . . . .

C12 C 0.0527(3) 0.4854(3) 0.4181(5) 0.090(2) Uani 1 1 d . . . . .

H12 H 0.059458 0.472830 0.459062 0.108 Uiso 1 1 calc R U . . .

C24 C -0.0005(3) 0.3609(2) 0.2699(5) 0.0843(18) Uani 1 1 d . . . . .

H24 H -0.021700 0.341481 0.238925 0.101 Uiso 1 1 calc R U . . .

C16 C 0.0736(3) 0.5442(3) 0.5164(6) 0.093(2) Uani 1 1 d . . . . .

C7 C 0.0580(3) 0.5231(3) 0.4323(5) 0.088(2) Uani 1 1 d . . . . .

N31 N 0.1724(3) 0.6307(3) 0.7810(6) 0.109(2) Uani 1 1 d . U . . .

C21 C 0.0613(3) 0.4181(3) 0.3585(6) 0.093(2) Uani 1 1 d . . . . .

H21 H 0.083362 0.437377 0.387726 0.112 Uiso 1 1 calc R U . . .

C20 C 0.0561(3) 0.3803(3) 0.3464(6) 0.093(2) Uani 1 1 d . . . . .

H20 H 0.074261 0.374247 0.370910 0.111 Uiso 1 1 calc R U . . .

C8 C 0.0470(3) 0.5413(2) 0.3717(5) 0.091(2) Uani 1 1 d . . . . .

H8 H 0.050634 0.566553 0.381756 0.109 Uiso 1 1 calc R U . . .

C27 C 0.0122(3) 0.5342(3) 0.1531(5) 0.097(2) Uani 1 1 d . . . . .

H27 H 0.019272 0.516474 0.130336 0.116 Uiso 1 1 calc R U . . .

C15 C 0.0610(3) 0.5248(3) 0.5855(7) 0.106(3) Uani 1 1 d . . . . .

H15 H 0.043337 0.497460 0.584525 0.127 Uiso 1 1 calc R U . . .

C26 C -0.0023(3) 0.5530(3) 0.1014(5) 0.099(2) Uani 1 1 d . . . . .

H26 H -0.004724 0.547108 0.044664 0.119 Uiso 1 1 calc R U . . .

C30 C -0.0100(4) 0.5857(3) 0.2133(5) 0.106(3) Uani 1 1 d . . . . .

H30 H -0.018498 0.602501 0.235015 0.127 Uiso 1 1 calc R U . . .

C14 C 0.0737(3) 0.5446(3) 0.6616(6) 0.100(2) Uani 1 1 d . . . . .

H14 H 0.064672 0.530201 0.711040 0.120 Uiso 1 1 calc R U . . .

C29 C 0.0049(4) 0.5688(3) 0.2664(6) 0.106(3) Uani 1 1 d . . . . .

H29 H 0.007720 0.575292 0.322763 0.127 Uiso 1 1 calc R U . . .

C17 C 0.1000(4) 0.5844(3) 0.5163(6) 0.108(3) Uani 1 1 d . . . . .

H17 H 0.109966 0.598595 0.466931 0.129 Uiso 1 1 calc R U . . .

C18 C 0.1111(4) 0.6028(3) 0.5930(6) 0.117(3) Uani 1 1 d . . . . .

H18 H 0.128520 0.630219 0.594320 0.141 Uiso 1 1 calc R U . . .

C32 C 0.2510(3) 0.6481(3) 0.7850(7) 0.109(3) Uani 1 1 d . U . . .

C33 C 0.3036(3) 0.6277(3) 0.7876(8) 0.113(3) Uani 1 1 d . . . . .

H33 H 0.283204 0.601186 0.788125 0.135 Uiso 1 1 calc R U . . .

C34 C 0.2940(3) 0.6580(3) 0.7872(8) 0.113(3) Uani 1 1 d . . . . .

C35 C 0.2412(5) 0.6735(6) 0.7470(18) 0.187(7) Uani 1 1 d . U . . .

H35 H 0.258788 0.694660 0.713559 0.225 Uiso 1 1 calc R U . . .

C3 C 0.2012(5) 0.6645(5) 0.7633(16) 0.192(7) Uani 1 1 d D U . . .

H3 H 0.196056 0.685555 0.760683 0.230 Uiso 1 1 calc R U . . .

C5 C 0.2226(6) 0.6174(7) 0.8250(17) 0.129(6) Uani 0.63(2) 1 d . U P A 1

H5 H 0.228805 0.600762 0.855384 0.155 Uiso 0.63(2) 1 calc R U P A 1

C4 C 0.1837(5) 0.6095(6) 0.8227(16) 0.122(6) Uani 0.63(2) 1 d D U P A 1

H4 H 0.164613 0.587787 0.852847 0.147 Uiso 0.63(2) 1 calc R U P A 1

C1 C 0.1421(6) 0.5627(9) 0.2563(17) 0.258(13) Uiso 1 1 d DG U . . .

C6 C 0.1458(8) 0.5995(9) 0.2751(17) 0.326(19) Uiso 1 1 d DG U . . .

H6 H 0.155607 0.611221 0.326823 0.391 Uiso 1 1 calc R U . . .

C2 C 0.1348(10) 0.6188(9) 0.217(3) 0.37(2) Uiso 1 1 d DG U . . .

H2 H 0.137296 0.643381 0.229075 0.447 Uiso 1 1 calc R U . . .

N9 N 0.1506(12) 0.5363(12) 0.315(2) 0.39(2) Uiso 1 1 d D . . . .

O3 O 0.1497(16) 0.4999(14) 0.281(3) 0.58(4) Uiso 1 1 d D . . . .

C13 C 0.1201(10) 0.6012(13) 0.139(2) 0.47(3) Uiso 1 1 d DG U . . .

H13 H 0.112777 0.614126 0.100004 0.559 Uiso 1 1 calc R U . . .

C19 C 0.1164(11) 0.5644(13) 0.1204(16) 0.42(2) Uiso 1 1 d DG U . . .

H19 H 0.106568 0.552710 0.068679 0.508 Uiso 1 1 calc R U . . .

C25 C 0.1274(9) 0.5452(9) 0.179(2) 0.337(19) Uiso 1 1 d DG U . . .

H25 H 0.124878 0.520550 0.166427 0.405 Uiso 1 1 calc R U . . .

O2 O 0.1705(14) 0.5612(14) 0.392(2) 0.49(3) Uiso 1 1 d D . . . .

C4S C 0.1826(10) 0.6033(13) 0.748(3) 0.137(10) Uani 0.37(2) 1 d . U P A 2

H4S H 0.162673 0.578054 0.731844 0.164 Uiso 0.37(2) 1 calc R U P A 2

C5S C 0.2218(11) 0.6136(13) 0.741(3) 0.135(10) Uani 0.37(2) 1 d . U P A 2

H5S H 0.229257 0.598498 0.708671 0.162 Uiso 0.37(2) 1 calc R U P A 2

loop_

_atom_site_aniso_label

_atom_site_aniso_U_11

_atom_site_aniso_U_22

_atom_site_aniso_U_33

_atom_site_aniso_U_23

_atom_site_aniso_U_13

_atom_site_aniso_U_12

I1 0.1125(4) 0.0849(3) 0.0906(4) -0.0053(2) 0.0135(3) 0.0288(3)

I2 0.1389(6) 0.1095(5) 0.1304(6) -0.0323(4) -0.0128(4) 0.0719(4)

I3 0.2152(10) 0.1438(6) 0.0809(4) 0.0296(4) 0.0174(4) 0.1101(7)

I5 0.1323(6) 0.1370(6) 0.1075(5) -0.0114(4) 0.0088(4) 0.0648(5)

Zn5 0.1053(7) 0.0763(5) 0.0670(5) 0.0057(4) 0.0184(4) 0.0502(5)

Zn6 0.0966(7) 0.1100(8) 0.0909(7) -0.0313(6) -0.0156(5) 0.0543(6)

N19 0.097(4) 0.083(3) 0.071(3) -0.004(3) 0.004(3) 0.050(3)

N25 0.094(4) 0.069(3) 0.069(3) 0.001(2) -0.009(3) 0.035(3)

N13 0.108(5) 0.099(5) 0.088(4) -0.029(4) -0.023(3) 0.061(4)

C22 0.104(5) 0.078(4) 0.067(3) -0.013(3) -0.008(3) 0.056(4)

C11 0.113(6) 0.086(4) 0.074(4) -0.016(3) -0.022(4) 0.061(4)

C23 0.096(5) 0.086(4) 0.079(4) -0.006(3) -0.009(3) 0.055(4)

C28 0.111(6) 0.077(4) 0.075(4) -0.011(3) -0.019(4) 0.050(4)

C10 0.100(5) 0.086(4) 0.070(4) -0.009(3) -0.009(3) 0.058(4)

C9 0.104(5) 0.080(4) 0.072(4) -0.009(3) -0.015(3) 0.049(4)

C12 0.125(6) 0.092(5) 0.077(4) -0.018(3) -0.022(4) 0.072(5)

C24 0.094(5) 0.076(4) 0.087(4) -0.012(3) -0.007(4) 0.046(4)

C16 0.118(6) 0.092(5) 0.098(6) -0.020(4) -0.032(5) 0.074(5)

C7 0.115(6) 0.092(5) 0.074(4) -0.022(3) -0.024(4) 0.065(5)

N31 0.095(5) 0.114(6) 0.109(5) -0.008(4) -0.004(4) 0.047(5)

C21 0.115(6) 0.096(5) 0.092(5) -0.024(4) -0.027(4) 0.070(5)

C20 0.101(5) 0.089(5) 0.105(6) -0.023(4) -0.026(4) 0.061(4)

C8 0.119(6) 0.078(4) 0.091(5) -0.016(4) -0.022(4) 0.060(4)

C27 0.129(7) 0.102(5) 0.074(4) -0.013(4) -0.012(4) 0.069(5)

C15 0.115(7) 0.104(6) 0.100(6) -0.038(5) -0.023(5) 0.056(5)

C26 0.124(7) 0.107(6) 0.066(4) -0.001(4) -0.008(4) 0.056(5)

C30 0.158(9) 0.113(6) 0.076(4) -0.014(4) -0.033(5) 0.089(7)

C14 0.131(7) 0.106(6) 0.074(4) -0.014(4) -0.012(4) 0.067(6)

C29 0.159(9) 0.115(6) 0.077(5) -0.021(4) -0.027(5) 0.093(7)

C17 0.146(8) 0.102(6) 0.081(5) -0.017(4) -0.026(5) 0.066(6)

C18 0.168(10) 0.088(5) 0.085(5) -0.013(4) -0.014(6) 0.057(6)

C32 0.094(6) 0.110(6) 0.121(7) -0.001(5) -0.004(5) 0.050(5)

C33 0.092(6) 0.096(6) 0.146(9) -0.001(6) 0.007(6) 0.044(5)

C34 0.096(6) 0.098(6) 0.147(9) -0.003(6) 0.000(6) 0.051(5)

C35 0.122(10) 0.153(12) 0.285(19) 0.019(13) -0.026(11) 0.067(9)

C3 0.119(10) 0.157(12) 0.31(2) -0.013(14) -0.020(12) 0.075(10)

C5 0.091(9) 0.126(12) 0.168(16) 0.010(11) -0.002(10) 0.052(9)

C4 0.096(9) 0.116(11) 0.154(15) 0.024(10) 0.002(10) 0.053(8)

C4S 0.097(14) 0.137(18) 0.16(2) 0.019(17) -0.010(16) 0.048(13)

C5S 0.106(15) 0.138(17) 0.16(2) -0.001(17) 0.014(15) 0.057(13)

_geom_special_details

;

All esds (except the esd in the dihedral angle between two l.s. planes)

are estimated using the full covariance matrix. The cell esds are taken

into account individually in the estimation of esds in distances, angles

and torsion angles; correlations between esds in cell parameters are only

used when they are defined by crystal symmetry. An approximate (isotropic)

treatment of cell esds is used for estimating esds involving l.s. planes.

;

loop_

_geom_bond_atom_site_label_1

_geom_bond_atom_site_label_2

_geom_bond_distance

_geom_bond_site_symmetry_2

_geom_bond_publ_flag

I1 Zn5 2.5631(13) . ?

I2 Zn6 2.5531(16) . ?

I3 Zn5 2.5436(12) . ?

I5 Zn6 2.5229(16) . ?

Zn5 N19 2.052(6) . ?

Zn5 N25 2.052(6) 15 ?

Zn6 N31 2.046(9) . ?

Zn6 N13 2.058(7) . ?

N19 C20 1.311(11) . ?

N19 C24 1.350(11) . ?

N25 C26 1.317(12) . ?

N25 C30 1.388(11) . ?

N13 C18 1.318(14) . ?

N13 C14 1.330(13) . ?

C22 C23 1.361(11) . ?

C22 C21 1.381(11) . ?

C22 C11 1.488(10) . ?

C11 C10 1.372(11) . ?

C11 C12 1.396(10) . ?

C23 C24 1.387(11) . ?

C28 C27 1.366(11) . ?

C28 C29 1.403(12) . ?

C28 C9 1.482(11) . ?

C10 C9 1.407(11) . ?

C9 C8 1.374(11) . ?

C12 C7 1.383(11) . ?

C16 C15 1.289(15) . ?

C16 C17 1.367(15) . ?

C16 C7 1.535(11) . ?

C7 C8 1.386(11) . ?

N31 C3 1.255(19) . ?

N31 C4 1.29(2) . ?

N31 C4S 1.40(5) . ?

C21 C20 1.387(12) . ?

C27 C26 1.392(13) . ?

C15 C14 1.392(12) . ?

C30 C29 1.365(13) . ?

C17 C18 1.376(13) . ?

C32 C5 1.31(2) . ?

C32 C35 1.36(2) . ?

C32 C5S 1.43(4) . ?

C32 C34 1.510(15) . ?

C33 C34 1.353(16) 2_665 ?

C33 C34 1.394(15) . ?

C35 C3 1.43(3) . ?

C5 C4 1.38(3) . ?

C1 C6 1.3900 . ?

C1 C25 1.3900 . ?

C1 N9 1.542(18) . ?

C6 C2 1.3900 . ?

C2 C13 1.3900 . ?

N9 O3 1.496(18) . ?

N9 O2 1.512(18) . ?

C13 C19 1.3900 . ?

C19 C25 1.3900 . ?

C4S C5S 1.37(5) . ?

loop_

_geom_angle_atom_site_label_1

_geom_angle_atom_site_label_2

_geom_angle_atom_site_label_3

_geom_angle

_geom_angle_site_symmetry_1

_geom_angle_site_symmetry_3

_geom_angle_publ_flag

N19 Zn5 N25 99.1(3) . 15 ?

N19 Zn5 I3 111.20(18) . . ?

N25 Zn5 I3 107.08(17) 15 . ?

N19 Zn5 I1 106.6(2) . . ?

N25 Zn5 I1 108.17(19) 15 . ?

I3 Zn5 I1 122.16(5) . . ?

N31 Zn6 N13 99.1(3) . . ?

N31 Zn6 I5 111.1(3) . . ?

N13 Zn6 I5 110.6(3) . . ?

N31 Zn6 I2 106.6(3) . . ?

N13 Zn6 I2 106.3(2) . . ?

I5 Zn6 I2 120.96(5) . . ?

C20 N19 C24 118.4(7) . . ?

C20 N19 Zn5 119.9(5) . . ?

C24 N19 Zn5 121.6(5) . . ?

C26 N25 C30 116.3(7) . . ?

C26 N25 Zn5 125.9(5) . 14_455 ?

C30 N25 Zn5 117.8(5) . 14_455 ?

C18 N13 C14 117.3(7) . . ?

C18 N13 Zn6 118.1(6) . . ?

C14 N13 Zn6 124.4(7) . . ?

C23 C22 C21 117.7(7) . . ?

C23 C22 C11 122.0(7) . . ?

C21 C22 C11 120.3(7) . . ?

C10 C11 C12 119.9(7) . . ?

C10 C11 C22 121.4(6) . . ?

C12 C11 C22 118.7(7) . . ?

C22 C23 C24 120.7(7) . . ?

C27 C28 C29 116.4(8) . . ?

C27 C28 C9 124.4(8) . . ?

C29 C28 C9 119.2(7) . . ?

C11 C10 C9 120.8(7) . . ?

C8 C9 C10 118.3(7) . . ?

C8 C9 C28 119.7(7) . . ?

C10 C9 C28 121.8(6) . . ?

C7 C12 C11 119.7(7) . . ?

N19 C24 C23 121.0(7) . . ?

C15 C16 C17 120.8(8) . . ?

C15 C16 C7 120.6(9) . . ?

C17 C16 C7 118.5(9) . . ?

C12 C7 C8 119.8(7) . . ?

C12 C7 C16 121.2(7) . . ?

C8 C7 C16 118.8(7) . . ?

C3 N31 C4 111.8(13) . . ?

C3 N31 C4S 105(2) . . ?

C3 N31 Zn6 122.7(10) . . ?

C4 N31 Zn6 123.5(10) . . ?

C4S N31 Zn6 118.2(16) . . ?

C22 C21 C20 119.1(8) . . ?

N19 C20 C21 123.1(7) . . ?

C9 C8 C7 121.4(7) . . ?

C28 C27 C26 120.4(8) . . ?

C16 C15 C14 120.4(10) . . ?

N25 C26 C27 124.0(8) . . ?

C29 C30 N25 122.0(8) . . ?

N13 C14 C15 120.9(10) . . ?

C30 C29 C28 120.9(8) . . ?

C16 C17 C18 116.7(10) . . ?

N13 C18 C17 123.8(10) . . ?

C5 C32 C35 117.3(15) . . ?

C35 C32 C5S 94(2) . . ?

C5 C32 C34 122.5(13) . . ?

C35 C32 C34 119.9(13) . . ?

C5S C32 C34 120.4(18) . . ?

C34 C33 C34 121.3(11) 2_665 . ?

C33 C34 C33 118.7(11) 3_565 . ?

C33 C34 C32 120.7(10) 3_565 . ?

C33 C34 C32 120.6(10) . . ?

C32 C35 C3 113.7(19) . . ?

N31 C3 C35 125.6(17) . . ?

C32 C5 C4 121.1(19) . . ?

N31 C4 C5 123.5(18) . . ?

C6 C1 C25 120.0 . . ?

C6 C1 N9 127(3) . . ?

C25 C1 N9 113(3) . . ?

C1 C6 C2 120.0 . . ?

C6 C2 C13 120.0 . . ?

O3 N9 O2 131(2) . . ?

O3 N9 C1 119(3) . . ?

O2 N9 C1 107(2) . . ?

C19 C13 C2 120.0 . . ?

C13 C19 C25 120.0 . . ?

C19 C25 C1 120.0 . . ?

C5S C4S N31 120(4) . . ?

C4S C5S C32 118(4) . . ?

_refine_diff_density_max 2.457

_refine_diff_density_min -0.990

_refine_diff_density_rms 0.181

_shelx_res_file

;

TITL jm045_auto_a.res in R-3

shelx.res

created by SHELXL-2017/1 at 12:12:00 on 22-Jul-2022

CELL 1.54184 38.6805 38.6805 16.0202 90.000 90.000 120.000

ZERR 3.00 0.0007 0.0007 0.0003 0.000 0.000 0.000

LATT 3

SYMM - Y, X - Y, Z

SYMM - X + Y, - X, Z

SFAC C H I N ZN O

UNIT 612 450 72 90 36 36

MERG 2

DFIX 1.430 0.020 C1 C2

DFIX 1.430 0.020 C2 C6

DFIX 1.430 0.020 C6 C13

DFIX 1.430 0.020 C13 C19

DFIX 1.430 0.020 C19 C25

DFIX 1.430 0.020 C25 C1

DFIX 1.550 0.020 C1 N9

DFIX 1.500 0.020 O2 N9

DFIX 1.500 0.020 N9 O3

DANG 2.660 0.040 O2 O3

DFIX 2.100 0.020 C4 C3

DFIX 2.850 0.020 C6 O2

DFIX 2.850 0.020 O3 C25

DANG 2.500 0.040 C6 N9

SIMU 0.040 0.080 1.700 C1 C6 C2 C13 C19 C25

SIMU 0.040 0.080 1.700 N31 C32 C35 C3 C5 C4 C4S C5S

FLAT 0.100 C1 C6 C2 N9 O3 C13 C19 C25 O2

FMAP 2

PLAN 20

SIZE 0.050 0.080 0.100

ACTA

L.S. 30

TEMP 30.00

WGHT 0.181100

FVAR 0.30066 0.62927

I1 3 -0.036085 0.258636 0.185435 11.00000 0.11252 0.08488 =

0.09057 -0.00526 0.01351 0.02880

I2 3 0.115581 0.682157 0.736208 11.00000 0.13894 0.10954 =

0.13040 -0.03231 -0.01280 0.07189

I3 3 0.037779 0.271143 0.410193 11.00000 0.21521 0.14377 =

0.08087 0.02960 0.01738 0.11014

I5 3 0.073832 0.575029 0.894415 11.00000 0.13226 0.13701 =

0.10750 -0.01139 0.00880 0.06480

ZN5 5 0.025038 0.299672 0.278290 11.00000 0.10530 0.07633 =

0.06698 0.00574 0.01839 0.05024

ZN6 5 0.114187 0.617202 0.772517 11.00000 0.09656 0.10996 =

0.09091 -0.03133 -0.01564 0.05427

N19 4 0.026814 0.352654 0.302147 11.00000 0.09722 0.08281 =

0.07141 -0.00371 0.00366 0.05041

N25 4 -0.012719 0.578736 0.127895 11.00000 0.09431 0.06925 =

0.06875 0.00058 -0.00941 0.03534

N13 4 0.098477 0.583837 0.664672 11.00000 0.10764 0.09949 =

0.08786 -0.02875 -0.02318 0.06060

C22 1 0.033403 0.426804 0.326507 11.00000 0.10430 0.07847 =

0.06667 -0.01323 -0.00826 0.05589

C11 1 0.037148 0.466489 0.341852 11.00000 0.11278 0.08634 =

0.07402 -0.01582 -0.02157 0.06063

C23 1 0.002453 0.397739 0.282356 11.00000 0.09611 0.08625 =

0.07865 -0.00564 -0.00862 0.05471

AFIX 43

H23 2 -0.016909 0.402660 0.260273 11.00000 -1.20000

AFIX 0

C28 1 0.016114 0.541594 0.236887 11.00000 0.11138 0.07727 =

0.07454 -0.01137 -0.01873 0.04965

C10 1 0.026459 0.484892 0.282270 11.00000 0.09974 0.08573 =

0.06982 -0.00948 -0.00941 0.05812

AFIX 43

H10 2 0.016164 0.472173 0.231551 11.00000 -1.20000

AFIX 0

C9 1 0.030877 0.522773 0.296945 11.00000 0.10443 0.07972 =

0.07212 -0.00908 -0.01533 0.04917

C12 1 0.052703 0.485441 0.418071 11.00000 0.12456 0.09187 =

0.07700 -0.01756 -0.02203 0.07224

AFIX 43

H12 2 0.059458 0.472830 0.459062 11.00000 -1.20000

AFIX 0

C24 1 -0.000535 0.360853 0.269925 11.00000 0.09395 0.07643 =

0.08691 -0.01246 -0.00670 0.04581

AFIX 43

H24 2 -0.021700 0.341481 0.238925 11.00000 -1.20000

AFIX 0

C16 1 0.073642 0.544249 0.516393 11.00000 0.11771 0.09156 =

0.09807 -0.01954 -0.03246 0.07366

C7 1 0.058016 0.523051 0.432305 11.00000 0.11480 0.09177 =

0.07431 -0.02207 -0.02413 0.06455

N31 4 0.172389 0.630660 0.780964 11.00000 0.09524 0.11442 =

0.10863 -0.00848 -0.00390 0.04690

C21 1 0.061345 0.418125 0.358497 11.00000 0.11547 0.09562 =

0.09186 -0.02445 -0.02655 0.07029

AFIX 43

H21 2 0.083362 0.437377 0.387726 11.00000 -1.20000

AFIX 0

C20 1 0.056121 0.380295 0.346434 11.00000 0.10120 0.08947 =

0.10477 -0.02255 -0.02638 0.06062

AFIX 43

H20 2 0.074261 0.374247 0.370910 11.00000 -1.20000

AFIX 0

C8 1 0.047014 0.541276 0.371657 11.00000 0.11946 0.07774 =

0.09068 -0.01556 -0.02162 0.06041

AFIX 43

H8 2 0.050634 0.566553 0.381756 11.00000 -1.20000

AFIX 0

C27 1 0.012208 0.534205 0.153052 11.00000 0.12888 0.10163 =

0.07430 -0.01326 -0.01171 0.06867

AFIX 43

H27 2 0.019272 0.516474 0.130336 11.00000 -1.20000

AFIX 0

C15 1 0.061011 0.524835 0.585518 11.00000 0.11519 0.10448 =

0.10005 -0.03836 -0.02286 0.05649

AFIX 43

H15 2 0.043337 0.497460 0.584525 11.00000 -1.20000

AFIX 0

C26 1 -0.002277 0.553039 0.101362 11.00000 0.12392 0.10693 =

0.06576 -0.00085 -0.00845 0.05637

AFIX 43

H26 2 -0.004724 0.547108 0.044664 11.00000 -1.20000

AFIX 0

C30 1 -0.009951 0.585741 0.213299 11.00000 0.15837 0.11280 =

0.07643 -0.01449 -0.03338 0.08946

AFIX 43

H30 2 -0.018498 0.602501 0.235015 11.00000 -1.20000

AFIX 0

C14 1 0.073677 0.544615 0.661615 11.00000 0.13115 0.10597 =

0.07370 -0.01426 -0.01239 0.06681

AFIX 43

H14 2 0.064672 0.530201 0.711040 11.00000 -1.20000

AFIX 0

C29 1 0.004944 0.568761 0.266352 11.00000 0.15869 0.11497 =

0.07675 -0.02070 -0.02718 0.09299

AFIX 43

H29 2 0.007720 0.575292 0.322763 11.00000 -1.20000

AFIX 0

C17 1 0.100010 0.584405 0.516346 11.00000 0.14610 0.10161 =

0.08070 -0.01737 -0.02577 0.06613

AFIX 43

H17 2 0.109966 0.598595 0.466931 11.00000 -1.20000

AFIX 0

C18 1 0.111096 0.602796 0.593008 11.00000 0.16814 0.08824 =

0.08535 -0.01272 -0.01375 0.05692

AFIX 43

H18 2 0.128520 0.630219 0.594320 11.00000 -1.20000

AFIX 0

C32 1 0.251025 0.648072 0.784960 11.00000 0.09426 0.11007 =

0.12090 -0.00100 -0.00398 0.05020

C33 1 0.303621 0.627685 0.787567 11.00000 0.09206 0.09600 =

0.14617 -0.00086 0.00686 0.04389

AFIX 43

H33 2 0.283204 0.601186 0.788125 11.00000 -1.20000

AFIX 0

C34 1 0.294031 0.657955 0.787164 11.00000 0.09556 0.09830 =

0.14665 -0.00313 -0.00002 0.05058

C35 1 0.241160 0.673467 0.746996 11.00000 0.12236 0.15312 =

0.28486 0.01918 -0.02606 0.06744

AFIX 43

H35 2 0.258788 0.694660 0.713559 11.00000 -1.20000

AFIX 0

C3 1 0.201173 0.664508 0.763339 11.00000 0.11869 0.15666 =

0.30817 -0.01254 -0.02003 0.07519

AFIX 43

H3 2 0.196056 0.685555 0.760683 11.00000 -1.20000

AFIX 0

PART 1

C5 1 0.222595 0.617421 0.824982 21.00000 0.09142 0.12555 =

0.16810 0.00955 -0.00243 0.05204

AFIX 43

H5 2 0.228805 0.600762 0.855384 21.00000 -1.20000

AFIX 0

C4 1 0.183726 0.609519 0.822703 21.00000 0.09612 0.11644 =

0.15403 0.02386 0.00210 0.05270

AFIX 43

H4 2 0.164613 0.587787 0.852847 21.00000 -1.20000

PART 0

AFIX 66

C1 1 0.142058 0.562697 0.256317 11.00000 0.25794

C6 1 0.145778 0.599493 0.275083 11.00000 0.32623

AFIX 43

H6 2 0.155607 0.611221 0.326823 11.00000 -1.20000

AFIX 65

C2 1 0.134807 0.618762 0.216519 11.00000 0.37219

AFIX 43

H2 2 0.137296 0.643381 0.229075 11.00000 -1.20000

AFIX 0

N9 4 0.150557 0.536311 0.315276 11.00000 0.38731

O3 6 0.149718 0.499892 0.281266 11.00000 0.57979

AFIX 65

C13 1 0.120117 0.601234 0.139187 11.00000 0.46590

AFIX 43

H13 2 0.112777 0.614126 0.100004 11.00000 -1.20000

AFIX 65

C19 1 0.116397 0.564437 0.120420 11.00000 0.42297

AFIX 43

H19 2 0.106568 0.552710 0.068679 11.00000 -1.20000

AFIX 65

C25 1 0.127367 0.545169 0.178983 11.00000 0.33741

AFIX 43

H25 2 0.124878 0.520550 0.166427 11.00000 -1.20000

AFIX 0

O2 6 0.170539 0.561159 0.391907 11.00000 0.49178

PART 2

C4S 1 0.182610 0.603269 0.748483 -21.00000 0.09711 0.13734 =

0.16150 0.01903 -0.01019 0.04778

AFIX 43

H4S 2 0.162673 0.578054 0.731844 -21.00000 -1.20000

AFIX 0

C5S 1 0.221788 0.613578 0.741264 -21.00000 0.10598 0.13798 =

0.15584 -0.00084 0.01401 0.05671

AFIX 43

H5S 2 0.229257 0.598498 0.708671 -21.00000 -1.20000

AFIX 0

HKLF 4

REM jm045_auto_a.res in R-3

REM R1 = 0.0753 for 6532 Fo > 4sig(Fo) and 0.0887 for all 9032 data

REM 386 parameters refined using 92 restraints

END

WGHT 0.1830 0.7042

REM Highest difference peak 2.457, deepest hole -0.990, 1-sigma level 0.181

Q1 1 -0.0258 0.2487 0.2181 11.00000 0.05 2.46

Q2 1 -0.0241 0.2629 0.1648 11.00000 0.05 1.54

Q3 1 0.0283 0.2573 0.3750 11.00000 0.05 1.53

Q4 1 0.0490 0.2712 0.3807 11.00000 0.05 1.41

Q5 1 0.0848 0.5666 0.9300 11.00000 0.05 1.32

Q6 1 0.1452 0.6856 0.7526 11.00000 0.05 1.08

Q7 1 0.1172 0.6662 0.7888 11.00000 0.05 0.95

Q8 1 0.1919 0.6254 0.4111 11.00000 0.05 0.93

Q9 1 0.0939 0.5845 0.8471 11.00000 0.05 0.75

Q10 1 0.1235 0.5018 0.8357 11.00000 0.05 0.70

Q11 1 0.1030 0.6734 0.8021 11.00000 0.05 0.69

Q12 1 0.1359 0.6864 0.6837 11.00000 0.05 0.63

Q13 1 0.2415 0.6714 0.8258 11.00000 0.05 0.61

Q14 1 0.1065 0.6653 0.6855 11.00000 0.05 0.61

Q15 1 0.0375 0.4430 0.3447 11.00000 0.05 0.61

Q16 1 0.1214 0.6007 0.8087 11.00000 0.05 0.57

Q17 1 0.1663 0.5874 0.3691 11.00000 0.05 0.56

Q18 1 0.1326 0.6993 0.7986 11.00000 0.05 0.53

Q19 1 0.0803 0.5409 0.5475 11.00000 0.05 0.48

Q20 1 0.0583 0.5673 0.9506 11.00000 0.05 0.48

;

_shelx_res_checksum 14443

_shelx_hkl_file

;

0 0 -327545.70 2254.25 7

0 0 -6162348.012905.00 7

0 0 -6160153.012825.20 9

0 0 -9 1881.31 278.362 7

0 0 -9 2552.87 218.338 8

0 0 -12 4972.35 421.154 8

0 0 -12 4912.74 419.773 14

0 0 15 299.033 107.202 10

0 0 -15 528.187 74.049 8

0 0 -18 169.14 33.2778 2

-1 1 -17 164.807 42.4782 2

0 -1 -17 245.19 44.7663 8

-1 1 -14-42.0608 56.0203 14

1 0 -14 -36.401 48.8879 8

-1 1 -14 3.68653 49.9502 8

0 -1 -14-11.1589 49.6571 8

0 1 14 13.6031 75.873 10

-1 0 14-67.5701 87.4051 10

-1 1 -11 203.613 47.5397 14

1 0 -11 245.048 49.6422 14

0 1 11 133.49 88.8662 10

0 -1 -11 162.971 49.0771 8

1 0 -11 238.813 51.2969 8

-1 1 -11 174.361 50.1094 8

0 -1 -8 152.331 42.7485 6

0 -1 -8 144.345 105.505 7

1 0 -8 129.306 117.941 7

-1 1 -8 361.669 136.993 7

1 0 -8 110.016 46.5044 8

-1 1 -8 234.99 49.4301 8

0 -1 -8 192.908 49.9921 8

1 0 -5 360.991 134.442 7

0 -1 -5 394.233 111.901 7

-1 1 -5 566.494 147.733 7

-1 1 -5 406.661 73.2514 9

0 -1 -2 2376.22 212.353 7

0 -1 120406.30 1649.98 10

-1 0 -425529.60 2036.79 7

1 -1 -421839.00 2037.90 7

0 1 -423642.30 2067.28 7

0 -1 427954.50 2046.70 10

0 1 -7 224.029 64.9971 9

-1 0 -7 316.233 121.848 7

0 1 -7 421.773 147.12 7

1 -1 -7 160.478 99.783 7

-1 1 10 1163.36 180.282 10

0 1 -10 1056.88 110.36 8

1 -1 -10 1075.64 112.306 8

1 -1 -10 847.642 157.013 7

0 1 -10 901.752 175.989 7

0 1 -13 52.212 46.9209 14

1 -1 -13-7.23236 50.1584 14

0 1 -13 7.37472 49.551 8

1 -1 -13 48.5022 50.4316 8

-1 1 13 150.539 89.6556 10

1 -1 -16 -2.9941 39.7017 8

0 1 -16 2.96595 40.7418 8

-1 0 -19-6.15021 18.0168 2

0 1 -19-4.99678 18.6594 2

1 -1 -19-4.87793 18.0841 2

-1 2 -18 334.095 43.9853 2

-1 -1 -18 258.092 41.6246 2

-2 1 15 617.331 132.614 10

1 1 15 453.776 120.272 10

2 -1 -15 758.097 88.9344 8

-1 2 -15 854.377 94.2057 8

-1 2 -1210619.70 860.299 14

2 -1 -1210582.50 862.505 14

-2 1 12 9112.64 917.112 10

1 1 12 8838.19 921.123 10

2 -1 -1210865.50 863.82 8

-1 2 -1210891.00 863.485 8

2 -1 -935587.70 2758.65 8

-1 2 -934345.00 2758.54 8

2 -1 -930601.30 2823.36 7

-1 2 -929428.00 2849.12 7

1 -2 935785.10 2826.14 10

-2 1 935779.90 2823.22 10

-1 2 -6 7877.40 706.002 7

-1 -1 -6 7149.87 633.239 7

2 -1 -6 7484.54 653.169 7

1 -2 6 8912.89 642.497 10

-1 2 -316952.70 1393.46 7

-1 -1 -316109.50 1346.17 7

-1 2 -316938.50 1343.80 9

-1 2 -316285.00 1343.53 4

-1 -1 329129.40 2228.64 10

1 -2 -323484.90 2194.68 7

-2 1 -327083.30 2206.34 7

-1 2 328512.30 2178.25 4

1 1 -6 3843.26 339.784 9

1 -2 -6 3395.38 367.106 7

-2 1 -6 3766.47 403.544 7

1 1 -6 3501.74 409.546 7

-1 2 9 3762.50 361.509 10

2 -1 9 3514.42 360.364 10

1 -2 -9 3157.09 344.576 7

1 1 -9 2916.77 367.274 7

1 -2 -9 2993.51 281.917 6

1 -2 -9 3438.90 287.258 8

1 1 -9 3423.98 286.504 8

1 1 -12 3929.79 330.422 14

-1 2 12 3435.70 389.061 10

1 -2 -12 3901.64 333.44 8

1 1 -12 3760.11 331.617 8

1 1 -15 43.5691 46.1948 8

1 -2 -15 10.4138 47.709 8

-1 -1 15 25.6933 76.429 10

-1 2 15 -12.618 70.3751 10

-2 1 -18 274.86 41.5654 2

1 -2 -18 333.671 43.1749 2

0 -2 -19 4.77798 17.2303 2

-2 2 -19 3.76221 18.2286 2

2 0 -16 153.815 47.8555 8

-2 0 13 27.0597 76.7344 10

0 2 13 -27.799 80.1335 10

2 0 -13 40.0548 48.4355 8

-2 2 -13-3.42573 44.0233 14

2 0 -13-3.54147 49.61 14

0 2 10 2325.68 255.975 10

2 -2 10 1843.15 247.203 10

-2 0 10 2059.28 247.643 10

2 0 -10 1680.11 242.12 7

2 0 -10 2009.69 185.008 8

2 0 -7 7451.28 616.09 9

2 0 -7 6207.44 671.182 7

-2 2 -7 7933.31 722.527 7

0 -2 -7 6687.49 650.818 7

2 -2 7 7646.30 655.917 10

-2 2 -429835.40 2616.23 9

0 2 435701.80 2615.85 4

2 0 -431575.40 2696.46 7

0 -2 -431413.00 2645.92 7

-2 2 -434568.90 2692.36 7

-2 2 -1 1194.79 126.371 7

0 2 1 1459.92 123.455 9

0 2 -2 3003.64 268.79 9

-2 2 2 2458.83 260.372 4

-2 2 595786.40 6886.29 10

0 -2 5106614.0 6905.11 10

0 2 -587927.10 6954.07 7

-2 0 -582563.20 6879.62 7

2 -2 -580388.60 6882.88 7

0 2 -573520.10 6856.24 9

0 -2 811875.60 1040.75 10

-2 2 812055.70 1038.09 10

2 -2 -811856.40 976.578 8

0 2 -811811.80 976.337 8

2 -2 -811925.70 974.86 6

2 -2 -813247.00 989.001 9

2 -2 -811101.90 1038.92 7

0 2 -810937.10 1080.27 7

0 2 -11 154.597 45.3594 14

2 -2 -11 188.35 48.8208 14

-2 2 11 260.104 98.9437 10

2 -2 -11 155.32 50.8321 8

0 2 -11 151.10 50.182 8

0 2 -11 176.16 91.8043 7

0 2 -14-27.2096 52.2557 14

2 -2 -14 47.6112 48.2884 8

0 2 -14 61.0455 48.2145 8

2 0 14 84.243 95.3429 10

-2 2 14-52.7979 89.6334 10

-2 0 -17 67.0445 36.7731 2

2 -2 -17 21.2871 31.0478 8

-2 -1 -19 53.1857 19.1141 2

-1 3 -19 57.7392 20.4045 2

3 -2 -16 1005.11 105.069 8

-3 2 13 1467.67 227.975 10

2 1 13 1962.28 253.291 10

-1 3 -13 1785.56 178.99 14

3 -2 -13 2222.40 187.908 8

-1 3 -13 1999.84 184.702 8

1 -3 10 6346.86 626.364 10

-3 2 10 6681.56 631.055 10

3 -2 -10 6689.12 567.531 8

-1 3 -10 6749.82 567.807 8

3 -2 -10 5397.27 611.083 7

-1 3 -10 6016.74 656.457 7

3 -2 -10 7217.13 569.203 14

-1 3 -10 7467.40 567.719 14

1 -3 7 3103.45 289.317 10

-3 2 7 3129.09 283.776 10

-1 3 -7 2669.71 351.074 7

3 -2 -7 2401.11 273.922 7

-3 2 4243933.016621.20 10

1 -3 4241938.016621.40 10

-1 3 -4204332.016661.40 7

-1 3 -4197343.016584.30 9

-1 3 -4184861.016569.50 4

-3 2 4210441.016558.30 4

1 -3 1 9960.34 773.317 10

-3 2 1 9161.33 767.741 10

-1 3 -1 8969.16 786.504 7

-3 2 1 8391.02 790.025 4

2 1 1 9837.48 764.526 9

-2 -1 243992.60 3348.69 10

-1 3 240619.40 3330.33 4

-3 2 -240554.40 3346.33 4

2 1 -5 2083.81 289.543 7

1 -3 -5 2153.65 232.213 7

-3 2 -5 2591.06 287.714 7

1 -3 -843342.30 3520.51 6

-3 2 -844747.70 3639.82 7

2 1 -842736.10 3613.25 7

1 -3 -840923.20 3572.16 7

2 1 -842535.50 3520.88 8

1 -3 -842582.50 3522.62 8

3 -2 846817.40 3590.57 10

-1 3 845931.40 3586.38 10

2 1 -852533.70 3553.73 9

2 1 -11 7337.02 597.748 14

3 -2 11 6976.34 665.221 10

-1 3 11 6909.97 663.206 10

2 1 -11 7443.50 599.555 8

2 1 -11 5808.49 656.487 7

1 -3 -11 7389.55 600.731 8

-1 3 14 3572.45 440.027 10

-2 -1 14 3633.39 442.101 10

2 1 -14 4798.60 393.017 8

1 -3 -14 4661.05 393.707 8

2 1 -14 4935.23 391.572 14

-3 2 -17 485.536 62.8505 2

1 -3 -17 547.53 65.6575 8

-2 3 -17 951.367 93.5005 2

3 -1 -17 795.672 88.2728 8

-2 3 -14 3680.16 305.625 14

3 -1 -14 3619.60 306.662 8

-2 3 -14 3629.62 307.004 8

-3 1 14 2676.28 349.894 10

1 2 14 2934.77 360.279 10

-2 3 -11 550.605 79.692 8

3 -1 -11 489.983 77.5313 8

3 -1 -11 550.707 114.991 7

1 2 11 582.468 135.468 10

-3 1 11 531.197 129.194 10

2 -3 11 653.448 141.475 10

-2 3 -11 835.466 82.4747 14

3 -1 -11 726.138 82.0853 14

-1 -2 -11 617.248 80.60 14

-3 1 846083.60 3477.18 10

2 -3 844558.90 3474.88 10

3 -1 -839761.70 3477.04 7

-2 3 -845152.60 3549.72 7

-1 -2 -840524.30 3405.02 6

3 -1 -849940.40 3433.24 9

-2 3 -841142.30 3408.19 8

3 -1 -841956.50 3407.07 8

3 -1 -5 5270.37 490.365 7

-2 3 -5 5257.70 538.162 7

-1 -2 -5 4541.81 449.353 7

-2 3 -5 4649.12 436.736 9

-2 3 -2119014.0 8607.24 9

-3 1 295300.40 8615.80 4

-2 3 -2102078.0 8640.92 4

-1 -2 125522.00 2020.27 10

-2 3 124399.10 2031.83 4

1 2 -4142620.011265.70 9

-3 1 -4131729.011265.20 7

1 2 -4140060.011333.20 7

2 -3 -4113729.011252.20 7

-2 3 4154359.011235.20 4

-1 -2 4162068.011298.40 10

2 -3 -738440.00 3351.99 7

1 2 -741033.60 3412.86 7

1 2 -744065.70 3337.57 9

-2 3 742052.70 3359.10 10

-3 1 -738764.70 3385.53 7

2 -3 -1011240.50 980.759 14

1 2 -1012737.30 982.353 8

1 2 -1010881.90 1064.08 7

3 -1 1011707.40 1052.59 10

-2 3 1011702.20 1043.21 10

2 -3 -1012625.70 983.788 8

-2 3 13 363.208 110.552 10

1 2 -13 352.871 65.194 8

2 -3 -13 429.004 69.3818 8

1 2 -13 284.622 61.47 14

2 -3 -16 107.114 44.755 8

1 2 -16 125.891 45.7775 8

2 -3 -19 81.4092 21.1566 2

-3 1 -19 83.5685 21.2612 2

-3 3 -18 16.81 25.3233 2

0 -3 -18 15.9943 23.8151 2

3 0 -15 1347.31 125.415 8

-3 0 15 1085.75 171.445 10

0 3 15 1152.41 176.711 10

-3 3 -15 1098.16 122.596 14

3 0 -12 340.426 61.0998 8

3 -3 12 265.811 118.907 10

-3 0 12 320.325 111.173 10

0 3 12 361.782 117.418 10

3 0 -12 341.66 63.2745 14

-3 3 -912963.70 1265.73 7

3 0 -912788.70 1226.25 7

3 0 -914860.90 1156.54 8

0 -3 -914196.80 1154.65 6

3 -3 914272.70 1220.85 10

0 3 914653.40 1227.80 10

-3 0 913717.20 1217.64 10

3 0 -6 3103.57 336.171 7

0 -3 -6 2801.58 289.263 7

-3 3 -6 3056.43 371.583 7

-3 3 -3 2497.92 260.903 4

-3 3 -3 2759.48 280.056 7

-3 3 042196.00 3972.74 4

0 -3 050344.60 3938.30 10

0 3 050779.30 3944.05 9

0 -3 311653.10 988.70 10

0 3 -310891.60 974.152 9

-3 3 312024.50 960.716 4

0 3 -6 1518.68 274.592 7

-3 3 6 1871.29 197.759 10

0 -3 6 1539.88 197.407 10

3 -3 -6 1341.73 175.676 7

0 -3 917834.30 1487.15 10

-3 3 918557.50 1486.96 10

3 -3 -918642.50 1421.11 8

-3 0 -916903.30 1419.50 8

3 -3 -917699.20 1417.75 6

0 3 -918026.50 1420.10 8

3 -3 -914033.50 1460.28 7

0 3 -916848.30 1528.79 7

0 3 -12 131.439 52.4217 8

3 -3 -12 157.991 49.9318 8

-3 3 12 145.605 98.7814 10

3 -3 -12 155.869 53.1089 14

-3 0 -12 175.322 49.8422 14

0 3 -12 215.462 49.9473 14

-3 3 15 231.838 91.4723 10

3 0 15 293.626 103.902 10

0 3 -15 409.824 62.6917 8

3 -3 -15 414.023 64.3781 8

-3 0 -18 25.5862 25.3996 2

-3 -1 -17 245.908 45.3029 2

4 -3 -17 212.011 42.9785 8

-3 -1 -17 242.487 45.8115 8

3 1 14 1699.84 221.613 10

-4 3 14 1010.98 185.643 10

-3 -1 -14 1505.73 150.218 14

-1 4 -14 1449.64 145.029 14

-1 4 -14 1483.63 149.248 8

-3 -1 -14 1655.76 152.187 8

4 -3 -14 1537.64 150.736 8

-1 4 -11 1355.07 134.839 8

1 -4 11 1085.60 182.183 10

-4 3 11 1218.41 186.645 10

-1 4 -11 1178.78 211.343 7

-3 -1 -11 1261.67 135.352 8

4 -3 -11 1299.76 133.409 8

-3 -1 -11 1221.50 132.253 14

4 -3 -11 1526.49 138.313 14

-1 4 -11 1473.79 132.989 14

1 -4 8 306.061 127.774 10

4 -3 -8 593.797 68.9665 8

-3 -1 -8 335.807 64.0173 8

-1 4 -8 309.944 61.7518 8

-1 4 -8 378.171 174.381 7

4 -3 -8 376.329 101.309 7

-1 4 -548471.30 4275.08 9

-1 4 -547040.30 4236.02 4

1 -4 560979.30 4297.88 10

-4 3 565855.90 4296.55 10

-1 4 -556781.40 4387.77 7

-1 4 -217208.50 1590.35 7

3 1 219821.20 1528.58 9

-1 4 -218537.10 1548.87 9

-1 4 -214448.60 1576.41 4

-4 3 218578.60 1566.08 4

1 -4 222043.00 1563.43 10

-4 3 219008.60 1559.74 10

-1 4 1 190.564 48.6006 9

-1 4 1 267.59 30.6704 7

1 -4 -4 217.994 47.2746 7

-4 3 -4 150.739 95.6895 7

-4 3 -4 243.331 69.218 4

-1 4 4 273.776 66.3983 4

-4 3 -7 3465.65 451.576 7

3 1 -7 4257.86 442.545 7

3 1 -7 4376.90 382.904 9

-1 4 10 5122.18 494.926 10

4 -3 10 4668.34 492.463 10

1 -4 -10 5384.34 429.955 6

3 1 -10 5200.98 428.655 14

3 1 -10 5088.73 428.811 8

3 1 -10 3925.74 487.725 7

-3 -1 13 27.2028 86.9224 10

4 -3 13 72.2669 92.3543 10

-1 4 13 40.765 78.456 10

3 1 -13 91.844 48.1628 8

3 1 -13 31.1696 46.1253 14

-4 3 -16 308.25 53.3371 2

3 1 -16 280.917 52.8916 8

3 1 -19 82.1524 21.9746 2

1 -4 -19 73.1279 20.0668 2

-4 3 -19 67.1705 20.8018 2

-2 4 -18 98.8211 29.8662 2

-2 -2 -18 91.1838 29.0158 2

-2 4 -15 498.326 70.7804 8

4 -2 -15 476.391 69.5547 8

-2 4 -15 480.287 69.0212 14

-4 2 15 535.453 115.55 10

2 2 15 208.051 90.4075 10

2 -4 12 752.742 157.14 10

2 2 12 804.383 173.562 10

-4 2 12 818.067 159.419 10

4 -2 -12 1024.69 110.122 8

-2 -2 -12 927.274 106.111 14

-2 4 -12 1064.38 105.354 14

4 -2 -12 1053.72 109.033 14

-2 4 -12 859.922 107.36 8

-2 -2 -9 123.888 43.5925 6

-2 -2 -9 65.6785 46.4369 8

4 -2 -9 107.544 46.2934 8

-2 4 -9 188.22 50.9276 8

-4 2 9 254.933 107.853 10

2 -4 9 198.244 104.696 10

4 -2 -9 74.9575 76.076 7

-2 4 -9 179.812 138.041 7

-2 4 -6 238.85 81.9812 9

-2 4 -6 349.609 194.931 7

4 -2 -6 298.016 97.8392 7

-4 2 6 451.338 123.884 10

2 -4 6 274.026 101.003 10

-4 2 3 221.106 89.6898 4

-4 2 3 195.908 104.04 10

2 -4 3 209.294 106.645 10

-2 4 -3 135.166 60.887 9

-2 4 -3 149.157 130.312 4

2 2 0 5362.91 444.27 9

-2 -2 0 4955.89 435.895 10

2 -4 0 5449.56 422.64 10

-4 2 0 4100.87 462.637 4

-2 4 0 5103.50 432.256 7

-2 4 0 5340.26 468.031 4

-4 2 -3 3601.57 279.364 4

-2 -2 3 3874.07 307.334 10

-2 4 3 3299.83 265.898 4

2 -4 -6 2869.72 304.967 7

2 2 -6 3442.62 381.997 7

2 2 -6 3587.53 312.173 9

-4 2 -6 2684.12 349.69 7

2 2 -923188.00 2045.67 7

2 2 -924889.90 1955.15 8

-4 2 -922673.10 1955.40 8

2 -4 -925450.80 1957.88 8

2 -4 -924173.50 1954.09 6

4 -2 925091.70 2025.13 10

-2 4 924374.40 2018.01 10

4 -2 12 2478.75 313.133 10

-2 4 12 2501.65 311.277 10

2 -4 -12 3133.41 258.567 8

2 2 -12 2171.89 305.133 7

2 2 -12 3073.19 256.239 8

2 2 -12 2822.60 252.423 14

-2 -2 15 266.071 93.3424 10

-2 4 15 242.641 92.4257 10

2 2 -15 373.55 64.8331 8

2 -4 -18-17.4853 25.0214 2

-4 2 -18 23.9032 25.543 2

-1 -3 -19 23.8308 16.9043 2

-3 4 -19 3.74039 18.2026 2

-3 4 -16 1423.82 130.389 2

4 -1 -16 1207.65 126.476 8

1 3 13 354.631 118.059 10

3 -4 13 337.112 108.505 10

-4 1 13 214.749 102.042 10

-3 4 -13 407.473 62.5763 14

4 -1 -13 206.183 58.3751 8

-1 -3 -10 1446.14 163.49 6

4 -1 -10 1251.72 208.727 7

4 -1 -10 1793.79 168.464 8

-4 1 10 1787.21 231.221 10

1 3 10 1610.30 233.037 10

3 -4 10 1933.62 235.925 10

-1 -3 -10 1790.42 170.42 14

-3 4 -10 2343.46 174.684 14

4 -1 -10 2081.00 172.793 14

-3 4 -7 4800.31 511.051 7

4 -1 -7 3741.94 409.804 7

-4 1 7 4082.07 411.467 10

3 -4 7 4206.24 412.577 10

-3 4 -436243.80 2975.38 4

-3 4 -434748.90 2957.80 9

-3 4 -440731.60 3043.31 7

-4 1 440516.60 3014.27 10

-3 4 -1 259.808 59.4039 7

3 -4 1 267.249 55.7883 10

1 3 1 368.332 52.1383 9

-4 1 -274001.90 6313.72 4

1 3 -279319.90 6322.62 9

-1 -3 288338.50 6314.65 10

-3 4 273759.10 6311.66 4

-3 4 5 4439.06 284.25 4

3 -4 -5 3384.63 308.024 7

1 3 -5 3225.81 395.99 7

-1 -3 5 3525.81 344.504 10

1 3 -5 2158.84 273.909 4

1 3 -5 2537.99 306.143 9

-1 -3 8 37.6269 97.3024 10

-3 4 8 45.5111 86.7052 10

3 -4 -8 34.0008 42.0057 8

1 3 -8 87.5989 43.0271 8

-4 1 -8 70.4332 45.3439 8

1 3 -8 39.4916 122.767 7

3 -4 -8 30.1386 63.4562 7

1 3 -8 148.736 80.6634 9

3 -4 -8 53.9729 39.8627 6

-3 4 11 4498.80 459.946 10

4 -1 11 4161.43 460.965 10

-4 1 -11 4551.82 396.872 14

3 -4 -11 4637.48 399.917 14

1 3 -11 4647.43 395.402 14

1 3 -11 4563.13 397.777 8

-4 1 -11 4573.52 398.654 8

1 3 -11 3724.73 466.877 7

-4 1 -11 5125.48 394.796 11

3 -4 -11 5011.54 401.065 8

1 3 -14 596.924 91.148 8

-4 1 -14 765.839 93.3646 8

3 -4 -14 739.952 94.2762 8

1 3 -14 838.508 93.3638 14

-4 1 -14 843.531 96.3717 14

-3 4 14 474.999 124.059 10

3 -4 -17 297.40 47.4607 8

-4 1 -17 298.255 48.8086 2

-4 4 -17 857.31 89.5686 2

4 0 -17 834.76 87.8822 2

-4 0 14-13.1641 77.1084 10

0 4 14 13.3633 78.2751 10

4 -4 14 14.0322 82.1933 10

4 0 -14 -20.685 50.3605 8

4 0 -11 656.829 129.442 7

4 0 -11 664.847 82.4314 14

0 -4 -11 602.087 84.6163 14

-4 4 -11 681.932 77.6321 11

-4 0 11 629.989 135.648 10

0 4 11 880.057 150.904 10

4 -4 11 760.832 144.60 10

4 0 -11 588.952 80.7744 8

-4 0 835537.40 2801.25 10

4 -4 834455.00 2793.32 10

4 0 -832504.80 2726.98 8

0 -4 -833411.50 2727.77 6

4 0 -838987.40 2762.56 9

4 0 -833538.00 2806.40 7

-4 4 -834393.90 2875.74 7

-4 4 -5 996.053 200.355 7

-4 4 -5 963.161 123.782 4

0 4 5 1312.55 122.616 4

-4 4 -2 1014.94 194.82 4

-4 4 1 3817.66 402.20 4

-4 4 1 4321.53 349.672 10

0 -4 1 4069.90 359.095 10

-4 4 457387.80 4001.19 4

0 4 -446285.20 4102.84 7

0 -4 453221.50 4037.28 10

-4 4 459597.40 4032.37 10

0 4 -447427.30 4027.51 9

0 4 -7 995.096 242.821 7

4 -4 -7 866.739 141.459 7

-4 4 7 1335.02 177.145 10

0 -4 7 1442.18 192.67 10

0 4 -7 766.315 158.126 9

-4 0 -1012847.30 1056.20 8

4 -4 -1012996.60 1056.14 8

4 -4 -1012627.10 1057.19 14

0 4 -1014306.40 1054.94 14

-4 0 -1012842.60 1054.27 14

0 4 -1011368.30 1149.48 7

0 4 -1012675.60 1054.33 8

0 -4 1012168.70 1116.51 10

-4 4 1012381.00 1115.13 10

4 -4 -13 3235.61 279.074 8

-4 0 -13 3099.63 276.25 14

0 4 -13 3429.71 276.221 14

-4 4 13 2711.97 328.999 10

0 4 -13 2990.40 275.725 8

-4 0 -13 2970.71 277.748 8

-4 0 -16 45.7813 40.8605 8

4 -4 -16 40.2171 41.4638 8

-4 0 -19 4.55846 16.8575 2

-4 -1 -18 88.7854 27.9477 2

4 1 15-13.3991 70.7546 10

1 -5 15 38.6776 75.5258 10

-5 4 15-35.2244 76.9011 10

-1 5 -15 12.2856 45.7732 14

5 -4 -15 58.6407 46.6366 8

-4 -1 -15 10.592 43.5817 8

-1 5 -12 760.888 95.1127 14

-4 -1 -12 818.426 101.243 14

1 -5 12 679.964 145.156 10

-5 4 12 831.915 150.214 10

5 -4 -12 947.729 102.503 8

-4 -1 -12 1025.79 106.561 8

-1 5 -12 941.728 101.531 8

1 -5 9 9963.27 861.867 10

-5 4 9 9469.10 849.976 10

-1 5 -9 9706.81 789.953 8

-1 5 -9 9873.15 927.87 7

5 -4 -9 8334.19 827.691 7

-4 -1 -9 9586.78 785.264 6

-4 -1 -9 9340.16 790.036 8

5 -4 -910288.30 791.056 8

-1 5 -6 1561.58 173.543 4

5 -4 -6 2142.92 216.761 7

-1 5 -6 2401.16 364.71 7

1 -5 6 2507.08 255.305 10

-5 4 6 2901.84 262.235 10

-1 5 -6 1678.78 245.672 9

-1 5 -3 7019.64 667.613 9

4 1 3 8248.35 626.348 9

-5 4 3 7919.93 670.32 4

-5 4 3 7013.76 673.849 10

1 -5 3 8213.19 677.086 10

-1 5 058269.50 4429.83 9

1 -5 057500.80 4414.73 10

-5 4 048010.30 4469.65 4

-1 5 051635.60 4427.58 7

-1 5 3114138.0 8808.16 4

-5 4 -3103470.0 8833.86 4

-1 5 6 9168.27 762.193 4

-5 4 -6 8444.12 857.936 7

4 1 -610248.30 843.863 7

4 1 -915956.80 1319.53 8

5 -4 916997.70 1390.48 10

-1 5 915750.70 1386.64 10

4 1 -919118.20 1352.82 9

4 1 -916190.80 1402.03 7

-5 4 -915625.70 1322.99 8

1 -5 -916120.80 1320.82 6

4 1 -12 620.251 76.4834 14

-5 4 -12 441.384 64.3344 11

4 1 -12 601.918 76.8353 8

-1 5 12 655.588 139.876 10

5 -4 12 556.411 133.843 10

-4 -1 12 448.472 120.98 10

4 1 -12 463.551 113.691 7

-4 -1 15 950.278 168.132 10

-1 5 15 1075.69 169.729 10

5 -4 15 1239.74 183.908 10

4 1 -15 1421.07 129.053 8

-5 4 -18 54.9433 27.506 2

4 1 -18 64.2678 27.2425 2

1 -5 -18 98.7803 26.9675 2

-3 -2 -19 41.5659 17.8501 2

-2 5 -19 29.8426 18.4766 2

-3 -2 -16 162.87 50.7822 8

5 -3 -16 145.57 45.7143 8

-2 5 -13 8855.53 724.655 14

-3 -2 -13 8110.28 727.357 14

3 2 13 8251.73 797.346 10

2 -5 13 7976.61 788.782 10

-5 3 13 7972.78 786.157 10

5 -3 -13 9805.11 729.957 8

-3 -2 -13 9382.20 732.341 8

-2 5 -13 8698.40 727.461 8

-3 -2 -10 168.644 51.0739 8

5 -3 -10 183.675 48.831 8

-2 5 -10 109.268 47.6659 8

5 -3 -10 195.763 53.2747 14

-2 5 -10 185.412 48.6873 14

-3 -2 -10 209.227 48.6831 14

-3 -2 -10 117.413 42.323 6

-5 3 10 137.398 86.6419 10

2 -5 10 90.2718 94.2532 10

5 -3 -10 89.435 64.5499 7

-2 5 -10 21.8582 104.422 7

2 -5 749004.70 3209.42 10

-5 3 750026.40 3208.84 10

-2 5 -750277.70 3353.47 7

5 -3 -741942.80 3184.01 7

-5 3 4 2964.32 256.145 4

-2 5 -4 2283.42 351.085 7

-2 5 -4 2711.67 269.171 9

2 -5 4 2079.87 270.557 10

-5 3 4 2892.91 291.241 10

-2 5 -4 2033.65 278.02 4

-2 5 -1129141.010843.50 7

2 -5 1138105.010824.20 10

-5 3 1136419.010820.10 10

-5 3 1132034.010868.50 4

3 2 1137050.010827.00 9

-5 3 -2 1908.74 260.615 4

-2 5 2 2543.48 241.749 4

-3 -2 2 2509.68 238.04 10

-2 5 5 444.294 81.01 4

2 -5 -5 315.502 60.0905 7

2 -5 -8 9.66758 43.7037 8

5 -3 8-37.2525 124.682 10

-2 5 8 81.6141 97.3608 10

-5 3 -8 20.3873 42.8735 8

3 2 -8 34.0558 37.2527 8

3 2 -8 33.4072 102.351 7

2 -5 -8 14.593 40.8447 6

3 2 -8 133.901 61.1872 9

3 2 -11 8114.81 649.20 14

-5 3 -11 7721.04 649.452 14

-5 3 -11 8360.63 648.54 11

-2 5 11 7329.18 712.972 10

5 -3 11 7475.92 720.592 10

2 -5 -11 7942.46 653.544 8

-5 3 -11 7719.07 651.825 8

3 2 -11 7845.41 650.066 8

3 2 -11 6355.03 714.932 7

-5 3 -14 3274.37 288.652 8

3 2 -14 3522.15 289.401 8

2 -5 -14 3701.22 293.361 8

-2 5 14 2842.90 338.28 10

5 -3 14 2411.91 337.677 10

-3 -2 14 2741.82 339.873 10

-5 3 -17 100.301 37.3188 2

3 2 -17 213.133 39.7187 2

2 -5 -17 163.625 40.741 8

-3 5 -17 46.6838 33.8203 2

-2 -3 -17 80.112 37.7725 8

5 -2 -17 61.7549 31.8584 8

2 3 14 543.038 138.548 10

3 -5 14 620.168 132.829 10

-5 2 14 439.606 122.438 10

5 -2 -14 702.879 91.0538 8

-2 -3 -14 618.818 91.2827 8

-3 5 -14 730.888 88.9209 8

-3 5 -14 854.519 88.7051 14

-5 2 11 1488.60 205.955 10

3 -5 11 1307.20 206.459 10

2 3 11 1543.29 217.069 10

5 -2 -11 1668.25 150.823 14

-3 5 -11 1541.21 146.041 14

-2 -3 -11 1285.74 145.808 14

-2 -3 -11 1697.01 152.846 8

5 -2 -11 1573.60 148.851 8

-3 5 -11 1396.29 146.686 8

-5 2 844105.80 3163.62 10

3 -5 841576.10 3159.25 10

-3 5 -840028.60 3262.06 7

5 -2 -835962.10 3144.95 7

-3 5 -837954.90 3088.60 8

-2 -3 -836314.00 3087.02 8

5 -2 -838965.30 3086.10 8

-2 -3 -839292.40 3085.68 6

3 -5 514719.00 1063.72 10

-5 2 515145.60 1075.12 10

-3 5 -513046.40 1137.87 7

-3 5 -511612.70 1013.48 4

-3 5 -510001.10 1018.03 9

-3 5 -219997.00 1676.28 9

2 3 222107.00 1666.09 9

-5 2 222706.80 1684.46 10

3 -5 220842.80 1687.28 10

-3 5 -219834.60 1698.10 7

-3 5 -215215.40 1721.86 4

-5 2 219639.70 1697.13 4

-2 -3 149393.50 3966.69 10

-3 5 149488.50 3996.24 4

-2 -3 4124068.0 8179.34 10

-3 5 4118791.0 8131.90 4

2 3 -4108497.0 8154.99 9

3 -5 -7 4041.14 429.528 7

2 3 -7 5528.35 530.22 7

2 3 -7 5353.86 450.272 9

-2 -3 7 5238.92 487.119 10

-3 5 7 4807.65 460.425 10

2 3 -10 39.1001 38.4671 14

-5 2 -10 52.4239 41.9866 14

3 -5 -10 118.223 44.5445 6

2 3 -10-17.4594 105.53 7

5 -2 10-27.9826 106.574 10

-3 5 10 50.2716 97.5097 10

2 3 -10 125.562 44.6309 8

-5 2 -10 78.0809 46.3161 8

3 -5 -10 42.8729 43.2713 8

-5 2 -13 34.1539 47.6476 8

3 -5 -13-15.2015 52.2102 8

2 3 -13 28.0236 43.8333 8

-3 5 13-13.1263 83.7869 10

5 -2 13-29.7713 95.0158 10

-5 2 -13 20.6031 32.0097 11

2 3 -13 16.6878 43.7205 14

-5 2 -13-14.0777 45.522 14

-5 2 -16-14.9366 38.5215 8

3 -5 -16 2.91085 38.6099 8

2 3 -16 25.9849 39.7846 2

-5 2 -16 35.711 36.6016 2

3 -5 -19 85.3022 20.4806 2

2 3 -19 63.533 20.9277 2

-5 2 -19 76.2264 20.6122 2

-1 -4 -18 157.206 32.5531 2

5 -1 -18 182.613 33.3267 2

-4 5 -18 175.559 34.2765 2

-4 5 -15 386.883 65.5884 14

-4 5 -15 603.708 69.0429 2

5 -1 -15 370.712 66.097 8

1 4 15 286.755 97.2709 10

-5 1 15 387.405 99.6427 10

4 -5 15 397.953 110.723 10

5 -1 -12 4874.89 387.029 8

-4 5 -12 4671.48 381.018 11

-5 1 12 4248.36 448.466 10

1 4 12 4461.45 459.089 10

4 -5 12 3552.03 439.857 10

5 -1 -12 4827.43 388.215 14

-4 5 -12 4799.77 385.52 14

-1 -4 -12 3812.51 385.253 14

4 -5 9 8992.04 799.933 10

-5 1 9 9551.45 801.903 10

-1 -4 -9 8931.82 732.361 8

5 -1 -9 8561.87 728.32 8

5 -1 -910778.10 763.463 9

-1 -4 -9 8556.97 728.775 6

-4 5 -9 8009.60 864.291 7

5 -1 -9 8178.17 791.322 7

-4 5 -636005.40 3203.61 4

-4 5 -643424.00 3367.90 7

1 4 642079.70 3207.30 4

-5 1 646066.10 3292.63 10

4 -5 645067.70 3273.24 10

-5 1 3249584.018310.10 10

-4 5 -3203187.018328.70 4

-1 -4 0 1012.31 106.028 10

-4 5 0 697.165 173.732 4

1 4 0 933.804 109.834 9

-5 1 0 1076.92 159.792 4

-4 5 310455.60 972.267 4

5 -1 311605.90 928.601 9

1 4 -311258.10 992.638 9

-1 -4 312839.00 989.099 10

-4 5 621282.00 1526.00 10

-1 -4 622234.20 1554.17 10

1 4 -623194.90 1638.97 7

4 -5 -617053.00 1495.08 7

-4 5 620381.80 1466.88 4

1 4 -617285.70 1517.61 9

1 4 -614749.40 1463.82 4

-1 -4 9 4498.57 462.738 10

-4 5 9 4706.49 454.493 10

4 -5 -9 3903.82 420.515 7

1 4 -9 4298.97 494.702 7

1 4 -9 4714.81 394.88 8

-5 1 -9 4487.04 396.074 8

4 -5 -9 5363.77 400.541 8

4 -5 -12 1539.56 144.01 8

1 4 -12 1314.90 139.488 8

-5 1 -12 1302.53 141.422 8

1 4 -12 1155.70 201.833 7

-5 1 -12 1496.85 134.561 11

5 -1 12 1354.07 206.558 10

-4 5 12 1067.71 188.829 10

1 4 -12 1571.82 139.11 14

-5 1 -12 1315.86 139.66 14

4 -5 -15 3367.69 273.867 8

-5 1 -15 3183.25 273.76 8

1 4 -15 3080.17 270.506 8

4 -5 -18 62.4088 24.4198 8

-5 1 -18 82.9971 27.4086 2

-5 5 -19-13.2679 17.6303 2

5 0 -19-18.1766 18.386 2

0 -5 -19 5.35652 15.9344 2

5 0 -16 66.9741 41.813 8

-5 5 -16 150.913 44.9841 2

-5 5 -13 124.174 37.5769 11

-5 0 13 26.936 81.6666 10

5 -5 13 -14.207 82.2542 10

0 5 13 41.3658 83.594 10

5 0 -13 130.94 47.1332 8

5 0 -10 46.9364 40.9688 8

0 -5 -10 18.3792 44.2835 14

5 0 -10 29.126 39.8979 14

0 -5 -10 93.7266 47.1809 6

5 0 -10 138.246 82.3507 7

-5 0 10 184.987 100.637 10

5 -5 10 52.7493 92.7168 10

0 5 10 95.029 102.43 10

5 0 -728519.70 2264.04 7

-5 5 -725171.70 2327.06 7

5 -5 726906.80 2247.04 10

-5 0 727478.80 2265.31 10

-5 5 -4 712.244 139.108 4

0 5 4 801.939 107.193 4

-5 5 -4 866.214 169.321 7

0 5 129283.10 2366.60 9

-5 5 2176617.014990.40 4

0 5 -2186643.014978.30 9

-5 5 2187597.014968.30 10

0 -5 2195006.014968.30 10

0 -5 540356.80 3282.51 10

-5 5 546084.30 3275.80 10

0 5 -543204.80 3391.31 7

-5 5 547599.30 3242.44 4

0 5 -531480.20 3271.43 9

0 5 -532874.90 3248.15 4

0 -5 825403.70 1887.95 10

-5 5 824953.70 1873.05 10

0 5 -819549.40 1858.75 9

0 5 -824251.40 1958.18 7

0 5 -822943.20 1806.51 8

5 -5 -819913.30 1840.11 7

-5 0 -821764.60 1807.00 8

5 -5 -822898.80 1807.15 8

-5 0 -11-3.42682 41.8465 14

0 5 -11 40.1656 39.843 14

0 5 -11 37.97 98.2941 7

-5 5 11 25.3604 79.8706 10

0 -5 11 67.1455 89.0415 10

5 0 11 45.9053 101.456 10

5 -5 -11 21.5493 41.2571 8

-5 0 -11 47.9125 45.647 8

0 5 -11-6.88103 39.8432 8

-5 0 -11-14.6776 35.98 11

-5 0 -14 169.018 59.4726 14

0 5 -14 239.779 56.3872 14

-5 0 -14 297.519 59.0166 8

0 5 -14 236.977 55.7821 8

5 -5 -14 286.157 60.3959 8

0 -5 14 270.046 102.762 10

-5 5 14 301.734 98.5717 10

-5 0 -17-26.0329 30.2216 2

5 -5 -17 11.1455 31.2331 8

6 -5 -16 264.20 50.5448 8

-5 -1 -16 211.411 52.6118 8

6 -5 -13 18.0485 49.4767 8

-5 -1 -13 42.5502 50.5366 8

-1 6 -13 55.9414 49.9339 8

-1 6 -13 34.7119 43.6306 14

-5 -1 -13-30.2561 56.3704 14

1 -6 13 13.8181 83.4468 10

-6 5 13 38.6179 77.7398 10

-1 6 -10 431.586 64.0585 8

-5 -1 -10 247.325 58.7656 8

6 -5 -10 336.957 60.2441 8

-1 6 -10 526.38 64.0213 14

-5 -1 -10 400.513 61.5022 14

-6 5 10 160.163 96.3565 10

1 -6 10 185.779 115.251 10

-1 6 -10 379.463 145.331 7

1 -6 7 210.351 122.749 10

-6 5 7 68.2921 84.5044 10

-1 6 -7-17.2156 102.68 9

-1 6 -7 87.424 183.696 7

6 -5 -7 140.454 65.3524 7

-1 6 -416655.90 1408.45 9

5 1 416099.20 1329.90 9

-6 5 419620.40 1383.55 4

-6 5 418974.10 1403.35 10

1 -6 416773.70 1394.40 10

-6 5 1 7104.54 596.734 4

-1 6 -1 6076.96 569.317 7

-6 5 1 6047.00 501.431 10

1 -6 1 6103.61 518.192 10

-6 5 -217063.40 1471.06 4

-1 6 5 6846.31 517.372 4

-6 5 -5 5158.52 518.675 4

-6 5 -5 5355.21 570.064 7

5 1 -835871.60 2518.23 9

-6 5 -829662.00 2479.46 8

5 1 -834764.30 2566.81 7

1 -6 -830133.80 2475.64 6

6 -5 831645.70 2541.83 10

-5 -1 830643.90 2552.26 10

5 1 -830025.70 2473.50 8

5 1 -11 1625.53 155.933 8

5 1 -11 1542.15 218.89 7

-6 5 -11 1471.67 158.663 8

5 1 -11 1659.95 156.731 14

-6 5 -11 1828.51 156.582 11

1 -6 -11 1662.97 157.07 6

-1 6 11 1289.00 209.696 10

-5 -1 11 1477.53 212.67 10

6 -5 11 1667.89 222.393 10

-6 5 -14 2749.38 224.484 11

-6 5 -14 2424.98 233.145 8

5 1 -14 2788.51 234.217 8

-5 -1 14 1951.92 278.305 10

-1 6 14 2263.34 281.916 10

6 -5 14 2705.69 303.182 10

-6 5 -17 643.985 71.8688 2

5 1 -17 599.902 69.2897 2

6 -4 -17 380.43 49.364 8

-4 -2 -17 281.477 49.5422 8

-4 -2 -17 355.592 50.248 2

6 -4 -14 397.08 66.3721 8

-4 -2 -14 407.494 69.0578 8

-2 6 -14 283.374 57.619 8

-6 4 14 226.986 91.7232 10

4 2 14 391.117 112.779 10

2 -6 14 109.006 86.8184 10

-2 6 -14 258.949 57.4195 14

-4 -2 -11 89.7102 36.2232 11

-2 6 -11 120.17 42.7763 14

-4 -2 -11 35.2479 43.6657 14

-2 6 -11 83.4051 110.217 7

-6 4 11 102.207 84.8727 10

2 -6 11 93.4977 83.1525 10

-2 6 -11 49.2791 41.7389 8

6 -4 -11 51.802 45.1227 8

-4 -2 -11 26.0016 47.0944 8

6 -4 -853923.70 4780.17 7

-2 6 -861658.60 4739.10 8

-2 6 -863572.30 4928.83 7

6 -4 -858577.30 4735.52 6

6 -4 -860989.70 4737.01 8

-4 -2 -855362.70 4736.79 8

2 -6 862031.00 4809.10 10

-6 4 864902.00 4809.00 10

-2 6 -516752.30 1520.80 7

-6 4 517885.20 1400.57 10

2 -6 516444.10 1394.96 10

-2 6 -515037.80 1403.46 9

-6 4 518237.00 1358.23 4

-2 6 -513829.60 1378.02 4

-2 6 -2185823.014508.10 7

-6 4 2189902.014457.90 10

2 -6 2184940.014461.00 10

4 2 2181162.014443.30 9

-2 6 -2177855.014466.70 9

-2 6 -2135539.014513.40 4

-6 4 2194325.014498.70 4

-2 6 154723.40 4413.18 7

-4 -2 156421.70 4433.89 10

-2 6 155157.10 4459.29 4

-2 6 4 1511.35 170.631 4

-6 4 -4 1498.15 184.243 4

4 2 -752800.60 4312.32 7

4 2 -752789.70 4261.95 9

-6 4 -1012768.50 975.204 14

2 -6 -10 9468.65 971.132 14

4 2 -1014348.20 974.966 14

2 -6 -1011365.90 971.755 6

-2 6 1012491.20 1043.65 10

6 -4 1011051.80 1038.87 10

4 2 -1012334.70 1058.20 7

4 2 -1012529.40 972.675 8

-6 4 -1011402.60 974.614 8

-6 4 -13 383.511 54.7467 11

-6 4 -13 314.986 61.6254 8

4 2 -13 332.387 62.0856 8

-2 6 13 158.294 94.0232 10

6 -4 13 322.245 109.10 10

-4 -2 13 272.03 113.706 10

4 2 -13 278.917 60.4765 14

-6 4 -16 531.869 66.5735 2

2 -6 -16 396.877 65.423 8

2 -6 -19 281.126 32.5129 2

4 2 -19 261.066 33.1546 2

-6 4 -19 264.662 33.0146 2

-3 -3 -18 101.273 27.9862 2

-3 6 -18 69.7866 27.2945 2

6 -3 -18 88.172 27.1205 2

-3 6 -15 153.917 50.3581 14

-3 -3 -15 237.571 54.4216 8

6 -3 -15 253.691 54.7124 8

3 3 15 168.828 86.2822 10

3 -6 15 38.9034 74.9705 10

-6 3 15 164.617 74.4857 10

-3 6 -12 1067.56 118.171 8

3 3 12 1273.26 195.546 10

3 -6 12 901.248 169.502 10

-6 3 12 881.471 167.276 10

-3 -3 -12 1191.05 122.123 8

6 -3 -12 1158.91 120.623 8

-3 -3 -12 1004.16 120.951 14

-3 6 -12 1281.46 118.648 14

-3 -3 -9 8188.78 643.689 6

-3 6 -9 7341.42 787.793 7

6 -3 -9 7418.75 695.429 7

-3 6 -9 7568.37 645.735 8

6 -3 -9 7614.33 643.483 8

-3 -3 -9 8056.40 647.453 8

3 -6 9 7496.08 706.85 10

-6 3 9 7985.40 711.968 10

-3 6 -6 9081.44 824.076 7

-6 3 6 8679.99 679.092 10

3 -6 6 9110.38 680.655 10

-3 6 -6 6897.29 609.052 4

3 3 318830.00 1440.71 9

-3 6 -315100.50 1460.21 9

-6 3 318169.40 1477.86 4

-3 6 -316293.70 1535.75 4

3 -6 317575.80 1476.66 10

-6 3 319615.90 1484.72 10

-6 3 0 1318.41 215.885 4

-3 6 0 1497.38 151.598 7

3 -6 0 1501.01 135.806 10

-3 -3 0 1693.35 162.563 10

-3 6 0 1733.35 220.01 4

3 3 0 1546.81 167.176 9

-6 3 -3 5827.27 540.619 4

-3 -3 3 5865.97 534.892 10

3 3 -3 5466.42 538.031 9

-3 6 3 6347.06 528.516 4

3 3 -6 7955.34 646.97 7

-3 6 6 7367.91 543.562 4

3 3 -6 7039.31 575.162 9

3 3 -9 154.604 115.526 7

3 3 -9 42.3125 39.9517 8

3 -6 -9 140.188 48.4763 8

-6 3 -9 141.276 48.7844 8

-3 6 9 36.0931 86.6456 10

6 -3 9 107.972 112.059 10

3 -6 -9 124.394 45.9256 6

3 3 -9 53.9981 56.4329 9

-6 3 -12 756.302 101.269 8

3 3 -12 855.089 99.3122 8

3 3 -12 955.75 99.4598 14

-6 3 -12 770.999 97.5764 14

6 -3 12 730.851 158.214 10

-3 6 12 733.637 144.743 10

3 -6 -12 964.381 105.469 8

3 3 -12 816.811 158.523 7

-6 3 -12 977.995 95.389 11

-6 3 -15 382.774 72.6467 14

3 3 -15 526.614 70.0873 8

-6 3 -15 526.427 71.3316 8

3 -6 -15 586.359 74.5195 8

6 -3 15 350.809 113.491 10

-3 -3 15 274.581 98.5643 10

-3 6 15 380.489 103.096 10

-6 3 -18 90.3869 28.6779 2

3 3 -18 100.233 29.0194 2

3 -6 -18 80.566 27.0624 8

6 -2 -19 58.7822 19.1959 2

-4 6 -19 50.2385 20.2126 2

-4 6 -16 1848.08 161.826 2

-2 -4 -16 1780.58 162.438 8

6 -2 -16 1835.03 159.745 8

-4 6 -16 1420.64 158.139 14

2 4 13 43.5776 86.4871 10

-6 2 13 39.8797 81.5208 10

4 -6 13-41.6796 86.3986 10

-4 6 -13 87.5509 47.0426 14

6 -2 -13 55.4948 45.448 8

-2 -4 -13 39.3154 49.3386 8

6 -2 -10 6797.73 698.138 7

4 -6 10 7094.00 707.299 10

-6 2 10 6768.81 703.704 10

2 4 10 7893.22 728.156 10

-2 -4 -10 6723.87 647.952 14

-4 6 -10 8540.30 652.078 14

6 -2 -10 8617.40 653.848 14

-4 6 -10 7286.65 775.315 7

-2 -4 -10 8287.93 649.571 6

-2 -4 -10 8432.88 653.453 8

6 -2 -10 7824.68 649.143 8

4 -6 734081.80 2863.09 10

-6 2 736845.50 2877.37 10

-4 6 -735182.10 2995.85 7

6 -2 -734050.30 2857.19 7

4 -6 4 2241.34 237.668 10

-6 2 4 1479.26 230.425 10

-4 6 -4 2009.70 255.812 4

4 -6 199119.50 8039.60 10

-6 2 192327.50 8089.86 4

-4 6 -1101829.0 8060.53 7

2 4 1106879.0 8052.06 9

-6 2 1100155.0 8047.00 10

2 4 -2 231.207 102.389 9

-2 -4 2 359.773 93.312 10

-6 2 -2 531.681 144.493 4

-4 6 2 211.509 120.812 4

2 4 -560652.40 3941.53 7

-2 -4 556339.40 3877.29 10

2 4 -549067.90 3848.58 9

-4 6 552282.20 3819.11 4

4 -6 -8 39.4471 40.9899 7

2 4 -8 58.6844 118.153 7

-4 6 8-56.2619 109.97 10

-2 -4 8 150.622 105.204 10

2 4 -8 95.072 62.3543 9

4 -6 -8 29.1197 45.5743 8

-6 2 -8 59.9944 43.3733 8

2 4 -8 29.2009 37.1909 8

2 4 -11 3727.22 333.585 14

-6 2 -11 3809.25 336.366 14

-4 6 11 3561.78 394.242 10

6 -2 11 3877.13 412.017 10

4 -6 -11 4061.33 340.676 8

-6 2 -11 4189.97 336.069 11

-6 2 -11 3900.14 339.384 8

2 4 -11 4053.30 338.129 8

2 4 -11 3335.17 410.552 7

2 4 -14 1275.53 137.456 14

-6 2 -14 1412.61 140.968 14

2 4 -14 1241.53 138.077 8

-6 2 -14 1515.32 142.848 8

4 -6 -14 1480.08 142.10 8

6 -2 14 1600.34 213.605 10

-4 6 14 1322.32 186.752 10

4 -6 -17 555.534 73.4441 8

-6 2 -17 775.108 77.2534 2

2 4 -17 775.698 76.9429 2

-1 -5 -17 33.1333 33.3981 8

-5 6 -17 48.1779 33.3416 2

5 -6 14 578.683 127.658 10

1 5 14 442.462 114.079 10

-6 1 14 336.13 106.39 10

-1 -5 -14 612.574 81.2978 8

6 -1 -14 520.084 73.2163 8

-5 6 -14 493.001 72.3577 14

-1 -5 -11 2369.46 228.548 8

6 -1 -11 2469.55 224.275 8

6 -1 -11 2342.01 278.978 7

-1 -5 -11 2229.50 225.376 14

6 -1 -11 2529.99 226.708 14

-5 6 -11 2650.00 226.591 14

-1 -5 -11 2468.67 224.698 6

-6 1 11 2416.49 286.947 10

1 5 11 2397.49 295.994 10

5 -6 11 2397.23 288.772 10

-5 6 -11 2839.86 225.509 11

-1 -5 -8 1089.29 108.863 6

5 -6 8 824.539 167.503 10

-6 1 8 768.672 172.122 10

-1 -5 -8 1000.35 110.265 8

6 -1 -8 1034.73 107.649 8

6 -1 -8 1345.26 180.41 7

-5 6 -8 780.461 242.025 7

-5 6 -545809.80 3798.93 7

-5 6 -538689.70 3709.32 4

5 -6 549293.60 3737.84 10

-6 1 547288.20 3756.72 10

1 5 549156.70 3695.18 4

-5 6 -247285.90 4451.99 4

-6 1 246222.10 4391.69 4

-5 6 -254794.40 4385.54 7

-6 1 257049.80 4377.31 10

1 5 259127.10 4368.04 9

-5 6 1130545.010729.50 4

-5 6 1136611.010658.20 10

-1 -5 1128345.010675.20 10

-5 6 4109180.0 8793.31 4

-1 -5 4112075.0 8817.46 10

-5 6 4114910.0 8801.55 10

1 5 -4101285.0 8820.62 9

1 5 -7 4233.64 459.412 7

5 -6 -7 2924.41 322.94 7

1 5 -7 3579.49 370.967 9

-5 6 7 4027.04 367.505 10

-1 -5 7 4068.20 388.104 10

-6 1 -10 1294.37 130.095 8

1 5 -10 1260.74 126.964 8

-6 1 -10 1155.20 125.574 14

1 5 -10 1322.94 124.852 14

1 5 -10 1243.97 221.441 7

5 -6 -10 1119.91 128.271 8

-5 6 10 1305.84 182.255 10

6 -1 10 1340.88 209.898 10

-1 -5 10 1257.58 190.801 10

-6 1 -13 2013.40 170.271 11

5 -6 -13 2000.36 181.728 8

1 5 -13 1988.18 174.974 14

-6 1 -13 1723.23 175.686 14

-6 1 -13 1760.89 177.403 8

1 5 -13 1833.00 176.362 8

-1 -5 13 1456.45 225.695 10

6 -1 13 2023.76 253.403 10

-5 6 13 1596.50 223.946 10

5 -6 -16 156.293 44.4627 8

-6 1 -16 121.912 46.2944 8

-6 1 -16 140.32 43.0748 2

1 5 -16 119.032 42.8593 2

1 5 -19 37.9092 19.6667 2

6 0 -18 13.2021 23.0617 2

0 -6 -18 1.6218 22.4769 2

-6 6 -18 10.8201 23.5546 2

6 0 -15 1422.22 128.888 8

6 -6 15 826.318 168.95 10

-6 0 15 993.828 167.961 10

0 6 15 1050.52 168.739 10

-6 6 -15 1447.42 130.145 2

6 0 -12 5233.27 430.845 8

6 0 -12 5523.17 433.691 14

-6 6 -12 5319.87 428.722 11

0 6 12 4356.90 489.566 10

-6 0 12 3834.77 478.943 10

6 -6 12 4466.60 493.059 10

0 -6 -9 634.173 79.7653 8

0 -6 -9 704.161 77.8647 6

-6 6 -9 522.068 77.5933 8

6 0 -9 420.459 67.6904 8

6 0 -9 534.964 128.244 7

-6 0 9 470.928 136.317 10

6 -6 9 518.397 127.075 10

6 0 -9 613.318 99.6236 9

-6 6 -6 1120.34 142.679 4

-6 6 -6 1598.21 260.862 7

0 6 6 1162.56 138.551 4

-6 0 6 1174.96 209.205 10

6 -6 6 1453.07 186.624 10

-6 6 -375124.80 6520.72 4

-6 0 383947.80 6480.88 10

0 -6 040149.00 3124.49 10

0 6 039595.20 3135.26 9

-6 6 032645.90 3196.04 4

0 6 -317516.60 1614.87 7

-6 6 319721.10 1531.24 4

0 6 -317962.30 1536.24 9

6 0 318067.40 1471.92 9

0 -6 318385.70 1514.93 10

-6 6 318179.50 1516.92 10

0 -6 618374.00 1288.35 10

-6 6 618317.70 1264.01 10

6 -6 -612167.70 1210.26 7

0 6 -621050.30 1429.98 7

0 6 -611750.70 1262.15 9

-6 6 616922.30 1214.53 4

0 6 -612390.50 1218.74 4

0 -6 9 108.47 106.079 10

-6 6 9 281.81 104.909 10

-6 0 -9 146.595 42.4701 6

0 6 -9 321.519 156.62 7

6 -6 -9 311.183 57.4468 8

-6 0 -9 149.425 51.5548 8

0 6 -9 161.966 48.6207 8

0 6 -12 31.3147 42.9397 8

6 -6 -12 25.7168 44.8401 8

-6 0 -12 7.68976 45.3683 8

0 6 -12 143.946 45.8578 14

-6 0 -12 50.7405 46.4147 14

-6 0 -12 57.6789 33.5908 11

0 6 -12 54.813 98.3719 7

0 -6 12 13.824 84.7627 10

-6 6 12 25.5239 72.4712 10

6 0 12-31.8612 108.229 10

6 -6 -15 4746.15 390.673 8

-6 0 -15 4547.81 391.472 8

0 6 -15 5007.70 391.757 2

0 6 -15 4581.59 387.967 14

0 6 -18 15.8939 25.2902 2

-6 0 -18 9.87429 23.1448 2

6 -6 -18 51.5064 23.3999 8

-6 -1 -17 353.536 46.0929 2

7 -6 -17 222.131 42.9888 8

7 -6 -14 3014.19 259.066 8

-6 -1 -14 2652.42 260.017 8

-1 7 -14 2682.23 253.424 14

1 -7 14 2370.06 312.138 10

-7 6 14 2510.21 306.861 10

7 -6 -11 1196.48 121.997 8

-1 7 -11 1402.83 125.504 8

-6 -1 -11 924.315 119.825 8

-1 7 -11 1278.64 120.162 14

-6 -1 -11 1013.03 119.68 14

-1 7 -11 914.778 201.145 7

-7 6 11 1080.50 172.016 10

1 -7 11 1193.61 182.916 10

-6 -1 -11 1204.46 116.438 11

7 -6 -8 523.479 75.533 8

7 -6 -8 510.955 73.836 6

-7 6 8 641.176 140.981 10

1 -7 8 932.119 161.017 10

-1 7 -8 644.552 77.3191 8

-6 -1 -8 605.004 78.4859 8

-1 7 -8 412.029 120.443 9

-1 7 -8 591.178 216.761 7

7 -6 -8 407.127 92.8182 7

-1 7 -5 3394.74 414.413 9

-7 6 5 4416.82 393.818 10

1 -7 5 3773.95 390.711 10

-1 7 -5 2939.68 386.366 4

-7 6 5 4207.55 366.952 4

-1 7 -218808.90 1619.09 7

-1 7 -218399.20 1553.77 9

1 -7 220088.80 1535.73 10

-7 6 217711.40 1519.47 10

6 1 219329.00 1522.49 9

-7 6 217667.00 1577.43 4

-1 7 140971.20 3495.27 7

-1 7 148852.20 3520.82 9

-1 7 4218576.016575.20 4

-7 6 -4190441.016603.60 4

-6 -1 729770.40 2579.28 10

7 -6 729980.20 2554.63 10

6 1 -733746.80 2576.98 7

6 1 -1026213.00 1877.17 14

1 -7 -1023110.40 1876.22 6

-7 6 -1021511.20 1877.48 8

6 1 -1023024.50 1873.56 8

6 1 -1022898.90 1950.21 7

-1 7 1022073.50 1942.29 10

7 -6 1022752.60 1942.10 10

-6 -1 1021913.60 1944.53 10

-7 6 -13 167.028 42.8702 11

7 -6 13-14.2844 88.0839 10

-1 7 13 132.789 83.1132 10

-6 -1 13 54.2859 81.3544 10

6 1 -13 86.4758 48.2893 8

-7 6 -13 200.365 52.8066 8

-7 6 -16 40.4367 34.6401 2

6 1 -19 99.1046 21.4804 2

1 -7 -19 126.745 20.6325 2

-5 -2 -18 115.98 27.3389 2

-2 7 -15 251.549 56.7404 14

-5 -2 -15 340.608 62.3017 8

7 -5 -15 334.559 57.1395 8

2 -7 15 177.622 92.1343 10

5 2 15 321.892 103.787 10

-5 -2 -12 22.6551 53.3307 14

-2 7 -12 180.024 47.7883 14

-5 -2 -12 238.92 42.1825 11

-7 5 12 232.802 88.1055 10

2 -7 12 219.334 95.3683 10

-5 -2 -12 250.741 58.3545 8

-2 7 -12 222.70 51.9173 8

7 -5 -12 230.309 57.0864 8

-2 7 -9 1583.73 135.986 8

7 -5 -9 1032.53 156.546 7

2 -7 9 1402.61 196.688 10

-7 5 9 1319.77 190.683 10

-2 7 -9 1338.23 280.848 7

-5 -2 -9 1148.88 131.472 8

7 -5 -9 1571.76 135.50 8

-5 -2 -9 1168.84 126.455 6

-7 5 641932.20 3214.34 10

2 -7 640663.10 3216.21 10

-2 7 -633815.50 3181.78 4

-7 5 645004.00 3170.49 4

-2 7 -628886.50 3229.49 9

-2 7 -645626.00 3403.83 7

-2 7 -324157.40 2000.54 7

-7 5 324281.70 1922.56 4

-7 5 325563.90 1913.65 10

2 -7 323046.60 1903.66 10

5 2 323515.90 1868.61 9

-2 7 -323181.40 1916.96 9

2 -7 012939.20 1026.36 10

-2 7 013216.70 1050.31 9

-2 7 012865.10 1040.07 7

-7 5 -3 7260.91 693.186 4

-2 7 3 7659.49 656.175 4

-2 7 6 7845.93 655.661 4

5 2 -6 7345.97 669.532 9

7 -5 9 301.063 110.001 10

-2 7 9 238.893 106.586 10

-7 5 -9 242.167 59.8521 8

5 2 -9 242.233 49.5796 8

5 2 -9 208.369 66.5608 9

5 2 -9 123.643 99.0919 7

2 -7 -9 243.901 56.6465 6

5 2 -12 98.6401 44.9793 14

-7 5 -12 172.125 52.142 14

5 2 -12 86.6017 72.1142 7

5 2 -12 73.3272 43.4239 8

-7 5 -12 172.908 51.7214 8

-5 -2 12-27.6527 82.6249 10

-2 7 12 53.2647 73.3914 10

7 -5 12 101.428 95.2211 10

-7 5 -12 88.9622 40.4631 11

5 2 -15 17.7478 38.7455 8

-5 -2 15-12.2838 68.4966 10

7 -5 15-13.2075 78.5613 10

-7 5 -15 13.2746 35.7149 2

5 2 -18 71.081 26.8904 2

-7 5 -18 70.3014 27.633 2

7 -4 -19 40.6826 17.9254 2

-3 7 -19 35.0493 19.3007 2

7 -4 -16 1050.73 109.441 8

-4 -3 -16 1163.73 114.055 8

-3 7 -16 1044.82 110.428 14

-7 4 13 2455.27 324.035 10

4 3 13 3058.43 353.769 10

3 -7 13 2555.35 327.494 10

-4 -3 -13 3164.77 279.032 14

-3 7 -13 3184.71 274.093 14

-3 7 -13 2782.20 273.862 8

-4 -3 -13 3505.20 282.566 8

7 -4 -13 3558.99 279.78 8

-3 7 -1018910.20 1518.47 8

7 -4 -1015786.00 1553.40 7

-3 7 -1018509.80 1662.00 7

-7 4 1018699.50 1580.15 10

3 -7 1017843.20 1580.18 10

-4 -3 -1019803.20 1515.75 6

7 -4 -1018896.70 1516.21 8

-4 -3 -1019609.90 1520.07 8

-4 -3 -1016677.20 1514.90 14

-3 7 -1020447.70 1518.85 14

-3 7 -710786.70 1010.00 7

7 -4 -7 9288.03 811.688 7

-7 4 7 9948.80 839.711 10

3 -7 7 8546.76 829.181 10

-3 7 -417068.70 1567.41 9

-3 7 -418016.10 1622.42 4

-7 4 422858.80 1566.77 4

3 -7 417749.40 1563.67 10

-7 4 417679.30 1572.21 10

-7 4 123198.10 1956.53 4

-7 4 122682.00 1883.55 10

4 3 124813.50 1900.18 9

-3 7 -122503.80 1910.47 7

3 -7 123703.90 1886.11 10

-3 7 2 1446.37 205.375 4

-7 4 -2 1061.47 229.482 4

-3 7 5 1001.69 143.89 4

-7 4 -5 1121.84 157.676 4

4 3 -5 1491.67 172.082 9

-7 4 -8 1621.29 189.083 8

4 3 -8 2159.28 187.401 8

4 3 -8 1938.54 273.201 7

-3 7 8 1928.56 243.657 10

4 3 -8 2138.43 224.442 9

3 -7 -8 2461.25 194.558 8

4 3 -11 190.933 49.7083 8

3 -7 -11 364.191 62.5614 8

-7 4 -11 77.5392 50.0072 8

-7 4 -11 197.225 53.7169 14

4 3 -11 167.067 47.7973 14

-7 4 -11 131.129 45.6279 11

3 -7 -11 321.744 58.4028 6

4 3 -11 160.936 92.5151 7

-3 7 11 260.153 99.2105 10

7 -4 11 160.522 112.06 10

-7 4 -14 25.3064 42.4745 8

4 3 -14 64.8642 45.667 8

3 -7 -14 7.33652 46.0086 8

-7 4 -14 65.5612 33.70 11

7 -4 14-28.8043 91.9834 10

-3 7 14 48.9843 72.9125 10

-4 -3 14 13.1921 69.7255 10

-7 4 -14 60.7191 48.3801 14

-7 4 -17 510.417 64.9945 2

4 3 -17 647.258 66.174 2

3 -7 -17 516.10 64.7556 8

-4 7 -17 9.54884 32.3812 2

-3 -4 -17 28.4179 31.7472 8

7 -3 -17 33.4578 29.7514 8

-3 -4 -14 57.3723 47.5716 8

7 -3 -14 80.2316 49.4849 8

3 4 14 28.1365 86.2037 10

-7 3 14 25.3297 66.9003 10

4 -7 14 68.1559 81.1074 10

-4 7 -14-31.0313 46.6104 14

-4 7 -11 7426.85 613.831 8

-4 7 -11 7872.35 610.31 11

3 4 11 7014.71 688.258 10

-7 3 11 6649.95 670.477 10

4 -7 11 6610.03 673.635 10

-3 -4 -11 7720.95 615.733 8

7 -3 -11 7526.95 611.79 8

-4 7 -11 7815.55 612.217 14

-3 -4 -11 6466.88 610.723 14

-3 -4 -11 7292.28 609.978 6

-7 3 853244.60 3980.51 10

4 -7 850424.30 3969.49 10

-3 -4 -848083.30 3897.64 6

-4 7 -849699.00 3902.53 8

7 -3 -850236.60 3955.62 7

-4 7 -855985.20 4136.77 7

7 -3 -848507.60 3897.47 8

-3 -4 -846330.40 3899.84 8

-4 7 -516761.60 1650.69 4

-7 3 521023.80 1675.73 10

4 -7 521319.50 1661.96 10

-4 7 -521771.80 1777.28 7

-4 7 -2 7001.57 857.902 4

-7 3 2 8269.21 791.428 4

-4 7 -2 9029.39 781.393 7

3 4 210269.80 748.12 9

-7 3 2 7963.32 748.566 10

4 -7 2 9346.32 754.12 10

3 4 -1 370.403 106.595 9

-3 -4 1 570.835 91.2036 10

-4 7 1 757.313 169.724 4

7 -3 4 240.306 27.3677 9

-7 3 -4 213.238 88.0809 4

-4 7 4 135.246 84.2204 4

3 4 -4 113.673 92.3331 9

-4 7 7 4100.32 436.182 10

-3 -4 7 4892.87 473.842 10

3 4 -7 5163.83 507.317 7

3 4 -7 4650.20 434.90 9

-7 3 -10 617.50 72.6371 14

3 4 -10 679.326 70.3286 14

7 -3 10 436.844 126.118 10

-4 7 10 322.309 109.56 10

3 4 -10 490.573 135.066 7

4 -7 -10 284.475 65.9631 6

-7 3 -10 376.875 69.2254 8

4 -7 -10 287.295 67.3236 8

3 4 -10 366.296 64.4307 8

-7 3 -13 830.184 93.5823 14

3 4 -13 904.198 93.1801 14

4 -7 -13 695.549 92.0888 8

-7 3 -13 770.986 83.9273 11

3 4 -13 632.699 87.8079 8

-7 3 -13 588.231 88.565 8

-4 7 13 445.242 123.545 10

7 -3 13 925.985 165.094 10

4 -7 -16 510.283 67.7479 8

-7 3 -16 552.084 67.4402 2

3 4 -16 450.712 66.0346 2

4 -7 -19 488.972 48.9258 2

3 4 -19 515.521 50.5795 2

-5 7 -18 28.9271 25.9542 2

-2 -5 -18 24.0536 22.5566 2

7 -2 -18 25.7479 22.8796 2

-5 7 -15 439.136 70.2034 14

7 -2 -15 486.53 70.1826 8

-2 -5 -15 615.107 79.0176 8

5 -7 15 387.175 109.506 10

-5 7 -15 563.562 71.7277 2

7 -2 -12 2664.21 240.092 8

-5 7 -12 3021.26 241.383 14

-5 7 -12 2826.15 236.599 11

-2 -5 -12 2720.60 244.474 8

5 -7 12 1851.77 283.828 10

-7 2 12 1858.88 284.707 10

2 5 12 2846.01 319.735 10

-7 2 9 3166.67 341.013 10

5 -7 9 3139.86 338.677 10

7 -2 -9 2709.27 320.536 7

-5 7 -9 3285.14 438.811 7

7 -2 -9 2913.94 268.503 8

-2 -5 -9 3257.89 275.272 8

-2 -5 -9 3275.10 272.628 6

-5 7 -643382.30 4169.60 4

-5 7 -655237.80 4340.53 7

5 -7 657329.80 4215.96 10

-7 2 657158.10 4238.15 10

2 5 654326.20 4160.76 4

-5 7 -342930.10 3809.97 7

-5 7 -344106.90 3877.15 4

-7 2 350371.90 3808.44 4

-7 2 350866.70 3798.17 10

5 -7 342803.80 3787.75 10

2 5 348048.10 3766.60 9

2 5 076036.90 6019.98 9

-2 -5 077470.00 6013.96 10

-7 2 069623.40 6062.84 4

-5 7 072378.40 6085.52 4

-7 2 -3 6362.20 622.096 4

2 5 -3 6745.98 626.164 9

-2 -5 3 6971.06 607.003 10

-5 7 3 6950.59 613.733 4

-5 7 637306.50 2505.16 4

-5 7 632960.90 2526.64 10

-2 -5 634823.90 2564.50 10

2 5 -628653.60 2501.23 4

5 -7 -625387.30 2479.58 7

2 5 -638346.50 2630.63 7

2 5 -631473.80 2536.34 9

-2 -5 9 393.48 132.969 10

-5 7 9 621.70 125.097 10

-7 2 -9 634.069 83.8784 8

2 5 -9 520.26 74.233 8

5 -7 -9 682.316 81.6989 8

2 5 -9 685.902 170.26 7

-7 2 -12 70.2191 38.58 11

2 5 -12 136.239 108.834 7

-7 2 -12 81.4788 48.3469 8

2 5 -12 58.1014 44.9514 8

5 -7 -12 68.1861 51.5774 8

-7 2 -12 49.4155 45.9693 14

2 5 -12 66.6446 42.8097 14

-5 7 12 101.95 88.6829 10

7 -2 12 47.0136 93.371 10

-7 2 -15 16.9173 45.5912 8

5 -7 -15 119.136 50.1638 8

-7 2 -15-39.5623 55.3996 14

7 -2 15 13.6013 85.6899 10

2 5 -18-6.81931 24.5246 2

-7 2 -18 -11.668 24.1023 2

5 -7 -18 9.43699 22.5673 8

7 -1 -19-1.05759 15.758 2

-6 7 -19 9.32481 16.8129 2

-1 -6 -16 335.27 58.9311 8

7 -1 -16 367.642 54.5944 8

-6 7 -16 270.105 58.4622 14

-6 7 -13 85.2344 47.0229 14

6 -7 13-55.7229 105.116 10

1 6 13 126.152 91.9114 10

-7 1 13 80.3259 77.3227 10

-6 7 -13 65.6847 36.8116 11

7 -1 -13 30.055 43.7161 8

-1 -6 -13 91.4355 50.632 8

-7 1 10 2821.04 336.266 10

6 -7 10 3058.66 337.708 10

-1 -6 -10 2727.22 275.234 14

7 -1 -10 3373.24 278.357 14

-1 -6 -10 3548.82 280.58 8

7 -1 -10 3003.84 271.474 8

-1 -6 -10 3356.84 276.803 6

7 -1 -10 2698.39 326.546 7

-6 7 -718909.10 1604.42 7

-7 1 721697.60 1514.53 10

6 -7 720873.70 1483.18 10

6 -7 432336.30 2555.27 10

-7 1 433787.50 2573.35 10

-6 7 -426856.20 2578.15 4

-7 1 149414.40 4328.46 4

6 -7 152376.20 4262.86 10

1 6 155665.10 4283.10 9

-7 1 154236.60 4279.23 10

-6 7 290118.90 7449.51 4

1 6 -294731.40 7435.06 9

-1 -6 298382.10 7412.31 10

-6 7 287652.50 7401.23 10

1 6 -5 336.719 189.244 7

-1 -6 5 553.891 136.42 10

-6 7 5 621.676 114.988 10

1 6 -5 348.51 120.92 9

-6 7 5 399.309 106.056 4

1 6 -5 221.229 112.708 4

6 -7 -8 4368.31 345.177 8

-7 1 -8 4119.56 348.381 8

1 6 -8 4286.67 343.458 8

1 6 -8 4392.57 485.876 7

6 -7 -8 2818.51 351.391 7

-1 -6 8 4276.37 426.039 10

-6 7 8 4231.26 404.896 10

6 -7 -8 4065.99 342.847 6

1 6 -8 3860.25 406.571 9

-7 1 -11 3.86103 43.5861 8

1 6 -11 51.0018 39.2216 8

6 -7 -11 18.4528 46.0061 8

-7 1 -11 79.8384 46.7342 14

1 6 -11 74.1315 42.8315 14

1 6 -11 19.3802 106.248 7

-7 1 -11 104.588 41.1554 11

-6 7 11-74.5911 79.4371 10

-1 -6 11 41.9134 93.8245 10

7 -1 11 31.9398 97.8972 10

1 6 -14 1583.41 150.005 8

-7 1 -14 1508.87 153.733 8

7 -1 14 1569.43 219.697 10

-6 7 14 1328.46 195.632 10

-7 1 -14 1558.87 154.301 14

1 6 -14 1598.89 150.435 14

-7 1 -14 1484.06 140.934 11

6 -7 -14 1719.85 154.112 8

-1 -6 14 1172.64 197.865 10

1 6 -17 171.116 39.5524 2

-7 1 -17 182.681 38.0719 2

6 -7 -17 98.7303 34.391 8

-7 7 -17 164.037 38.4842 2

7 0 -17 185.43 38.7764 2

0 -7 -17 147.354 38.8961 8

-7 7 -14 125.769 47.43 2

-7 7 -14 97.1786 34.1659 11

7 -7 14 13.6874 81.6261 10

0 7 14 38.2373 77.1775 10

-7 0 14 12.9529 67.3166 10

7 0 -14 50.9331 42.1056 8

0 -7 -11 8439.13 652.141 8

7 0 -11 8122.68 646.357 14

0 7 11 7515.16 715.671 10

7 -7 11 7504.63 712.834 10

-7 0 11 6773.83 706.633 10

7 0 -11 6878.08 702.974 7

-7 7 -11 7075.28 645.413 8

7 0 -11 8164.68 644.213 8

0 -7 -11 8001.41 645.561 6

-7 7 -11 7704.24 643.24 11

0 -7 -8 152.43 51.8987 8

-7 7 -8 85.6778 51.7663 8

7 0 -8 141.237 42.0843 8

7 0 -8 157.354 94.5673 7

0 -7 -8 125.406 47.483 6

7 0 -8 62.826 70.2188 9

-7 0 8 121.796 118.365 10

7 -7 8 98.1749 93.8784 10

-7 7 -5 1243.05 177.121 4

-7 0 5 1074.98 198.027 10

-7 7 -5 1228.10 224.957 7

0 7 5 1249.80 148.17 4

0 7 2175936.013421.30 9

-7 0 2169628.013424.50 10

-7 7 -2141177.013505.80 4

0 -7 147231.30 3687.77 10

-7 7 141738.90 3759.10 4

-7 7 460612.20 4809.73 4

-7 7 461233.80 4813.24 10

0 -7 463852.30 4820.67 10

0 7 -459218.60 4848.52 9

7 0 458257.50 4752.13 9

7 -7 -717875.00 1771.66 7

0 7 -727174.00 1967.21 7

0 7 -719374.60 1773.56 4

7 -7 -724478.30 1754.69 6

-7 7 726135.60 1826.19 10

0 -7 723858.60 1843.48 10

0 7 -716762.30 1830.80 9

-7 0 -10 1356.91 134.858 8

7 -7 -10 1290.93 130.93 8

0 7 -10 1279.16 129.296 8

-7 0 -10 1306.55 129.643 14

0 7 -10 1304.76 127.907 14

0 -7 10 1119.94 189.57 10

-7 7 10 1113.69 179.28 10

7 -7 -10 1226.17 128.719 6

0 7 -10 1161.08 226.85 7

-7 0 -13 4191.49 341.211 11

-7 7 13 3217.42 393.727 10

0 -7 13 3233.73 400.476 10

7 0 13 3895.73 427.125 10

7 -7 -13 4296.59 350.333 8

0 7 -13 4231.90 346.671 8

-7 0 -13 3748.98 348.943 8

0 7 -13 4132.58 344.356 14

-7 0 -13 4016.82 348.173 14

-7 0 -16 181.347 45.8992 2

0 7 -16 259.171 51.385 2

-7 0 -16 268.538 49.8815 8

7 -7 -16 245.393 49.8644 8

0 7 -19-4.57899 17.2622 2

8 -7 -18-1.45852 20.2712 8

-1 8 -18 22.2709 24.651 2

-1 8 -15 1496.30 132.466 14

8 -7 -15 1266.03 130.059 8

-7 -1 -15 1333.62 135.09 8

-1 8 -15 1184.41 130.506 2

-1 8 -12 440.607 141.755 7

8 -7 -12 592.706 76.4878 8

-7 -1 -12 396.608 72.504 8

-1 8 -12 508.93 72.4769 8

-7 -1 -12 480.213 76.0139 14

-1 8 -12 554.195 70.5488 14

-8 7 12 391.434 116.486 10

1 -8 12 293.493 114.547 10

-7 -1 -12 584.718 68.3587 11

-7 -1 -913090.50 1078.73 8

8 -7 -913997.60 1077.46 8

-1 8 -913326.30 1076.55 8

-1 8 -914368.70 1250.78 7

-8 7 915079.80 1146.40 10

1 -8 913863.20 1154.22 10

8 -7 -913841.40 1075.85 6

-7 -1 -911763.90 1069.13 6

-8 7 674906.20 5236.79 4

-1 8 -675180.10 5456.13 7

-1 8 -656435.70 5251.12 4

1 -8 665356.30 5272.54 10

-8 7 673122.70 5267.36 10

-8 7 3 1400.82 207.341 4

-1 8 -3 1827.54 318.064 7

7 1 3 1374.77 140.492 9

-1 8 -3 1415.85 208.535 9

-8 7 3 1384.55 174.747 10

1 -8 3 1632.64 181.919 10

-1 8 022122.40 1838.09 9

1 -8 023707.30 1821.74 10

-8 7 022391.20 1930.67 4

-1 8 020700.80 1841.51 7

-8 7 -3161358.012979.60 4

-1 8 643889.00 3316.52 4

-8 7 -636688.70 3319.43 4

-7 -1 643575.60 3385.01 10

1 -8 -9 2191.01 207.767 8

8 -7 9 2037.50 264.59 10

-7 -1 9 2437.64 287.249 10

1 -8 -9 2008.39 202.238 6

7 1 -9 2705.30 246.327 9

7 1 -9 2790.04 285.038 7

7 1 -9 2428.14 202.092 8

-8 7 -9 1978.20 207.384 8

7 1 -12 356.62 59.8716 8

-8 7 -12 314.481 62.5021 8

7 1 -12 229.963 58.7125 14

8 -7 12 225.232 112.781 10

-1 8 12 394.00 117.766 10

-7 -1 12 308.089 111.284 10

1 -8 -12 465.481 65.0548 6

7 1 -12 175.759 81.761 7

-8 7 -12 292.253 54.6799 11

7 1 -15 852.206 92.2081 8

-8 7 -15 909.234 96.1339 2

-8 7 -15 774.979 81.996 11

7 1 -18 310.817 40.0127 2

-8 7 -18 278.738 40.6542 2

8 -6 -19-6.07702 15.9809 2

-6 -2 -16 542.181 65.2281 8

8 -6 -16 435.103 60.0983 8

8 -6 -13 333.122 65.9537 8

-6 -2 -13 328.794 68.8486 8

-2 8 -13 580.568 68.6807 14

-6 -2 -13 348.255 71.8992 14

2 -8 13 384.805 115.671 10

-8 6 13 318.117 108.585 10

-6 -2 -13 436.504 58.0034 11

-6 -2 -1010910.80 901.36 6

-6 -2 -10 9929.00 902.399 14

-2 8 -1011612.00 905.748 14

-6 -2 -1011638.20 909.262 8

8 -6 -1011641.70 906.363 8

-2 8 -1011948.60 1064.34 7

-2 8 -1010747.70 905.314 8

2 -8 1010837.90 973.784 10

-8 6 1011676.80 971.808 10

-2 8 -7 1828.45 218.041 4

-2 8 -7 3025.83 448.732 7

2 -8 7 2281.43 272.251 10

-8 6 7 2587.20 271.338 10

-2 8 -7 1337.48 281.892 9

8 -6 -7 2105.64 196.408 6

-2 8 -7 2371.87 199.272 8

8 -6 -7 2441.33 233.426 7

6 2 4 633.046 74.9014 9

-2 8 -4 647.216 152.501 9

-8 6 4 482.003 122.102 4

-2 8 -4 659.19 208.254 4

2 -8 4 695.68 127.885 10

-8 6 4 527.371 132.974 10

-8 6 1 1602.42 272.224 4

6 2 1 1939.25 193.323 9

-8 6 1 1594.34 155.665 10

2 -8 1 1708.54 169.926 10

-2 8 -1 2072.56 225.926 7

-2 8 222009.70 1744.47 7

-2 8 222233.20 1804.35 4

-8 6 -218459.60 1844.52 4

-2 8 5 6134.22 505.045 4

-8 6 -5 4943.32 509.154 4

6 2 -835793.20 2633.37 9

8 -6 830235.30 2646.35 10

2 -8 -831998.90 2587.65 6

-8 6 -830460.20 2592.50 8

6 2 -834156.80 2673.50 7

6 2 -832708.30 2584.57 8

6 2 -11 73.8204 48.2904 8

-8 6 -11 100.476 52.4605 8

6 2 -11 121.337 49.4272 14

-8 6 -11 153.795 52.8878 14

2 -8 -11 123.831 52.3314 6

-2 8 11 92.8992 97.5161 10

-6 -2 11 42.8163 86.5614 10

8 -6 11 141.783 106.184 10

-8 6 -11 89.8443 45.8026 11

6 2 -11 59.5033 80.4585 7

-8 6 -14 157.89 51.6167 14

-8 6 -14 127.856 47.4514 2

-8 6 -14 58.0769 33.6936 11

6 2 -14 75.2414 42.2733 8

-2 8 14 85.0185 77.8338 10

8 -6 14 27.8882 81.9699 10

-6 -2 14 78.5092 79.3058 10

-8 6 -17 34.7174 31.43 2

6 2 -17 70.4508 32.0737 2

8 -5 -17 250.25 44.5382 8

-5 -3 -17 317.259 48.2938 8

-5 -3 -17 337.942 46.8347 2

-8 5 14 491.896 122.343 10

3 -8 14 548.629 127.977 10

5 3 14 587.52 140.661 10

-5 -3 -14 716.128 92.5277 8

8 -5 -14 724.254 88.1534 8

-3 8 -14 836.806 87.031 14

8 -5 -11 5290.97 421.812 8

-5 -3 -11 4808.75 422.358 8

-5 -3 -11 4217.45 418.597 14

-3 8 -11 5161.68 419.341 14

-3 8 -11 4782.03 420.339 8

-3 8 -11 4466.59 537.363 7

-8 5 11 4607.89 479.921 10

3 -8 11 4331.85 479.995 10

-3 8 -11 5546.24 419.223 11

-5 -3 -11 5176.44 416.205 11

-5 -3 -8 9287.87 767.041 6

-8 5 810402.80 847.40 10

3 -8 8 9917.27 848.242 10

-5 -3 -8 8827.58 770.303 8

8 -5 -8 9072.02 768.254 8

-3 8 -810019.90 773.039 8

-3 8 -810287.90 1005.50 7

8 -5 -8 9500.31 812.847 7

-3 8 -5 118.26 115.003 4

-3 8 -5 105.474 109.521 9

-8 5 5 161.182 103.47 10

3 -8 5 36.2112 92.2099 10

-8 5 5 118.863 86.0787 4

-8 5 2364589.026297.20 4

5 3 2327344.026228.30 9

-3 8 -2311614.026254.80 9

-8 5 2320902.026229.40 10

3 -8 2349734.026237.70 10

-3 8 -2337224.026287.60 7

-3 8 -2275931.026344.90 4

-3 8 1322709.025478.30 7

-3 8 1328367.025538.90 4

-5 -3 1326041.025501.10 10

5 3 -1297308.025514.50 9

-3 8 4 509.172 109.087 4

-8 5 -4 379.517 116.815 4

5 3 -713560.30 1157.07 7

5 3 -712898.70 1110.61 9

-8 5 -10 3132.46 261.059 14

5 3 -10 3145.68 255.471 14

3 -8 -10 2871.49 257.376 6

-8 5 -10 2815.49 256.223 11

8 -5 10 2637.10 321.275 10

-3 8 10 2500.83 309.054 10

-8 5 -10 2530.24 258.745 8

5 3 -10 2994.83 334.862 7

5 3 -10 2993.72 255.479 8

5 3 -13 2786.52 230.525 14

-8 5 -13 2743.66 229.717 14

-8 5 -13 2692.86 224.454 11

-5 -3 13 1984.43 279.794 10

-3 8 13 2030.10 277.712 10

8 -5 13 2133.48 290.917 10

3 -8 -13 2486.85 231.855 8

5 3 -13 2497.55 226.226 8

-8 5 -13 2377.81 230.065 8

-8 5 -16 54.2832 47.1977 14

3 -8 -16 206.553 48.7213 8

5 3 -16 165.266 44.1296 2

-8 5 -16 143.703 43.9706 2

5 3 -19 8.61655 17.1563 2

3 -8 -19 25.4663 15.3139 2

8 -4 -18 40.6262 24.2956 2

-4 8 -18 8.90543 24.4767 2

-4 8 -15 692.92 84.8662 14

-4 -4 -15 733.336 88.3372 8

8 -4 -15 672.812 84.7227 8

-4 -4 -12 101.813 53.1799 14

-4 8 -12 213.827 51.2769 14

-8 4 12 39.7301 77.4203 10

4 -8 12 136.475 95.6593 10

4 4 12 283.862 119.567 10

-4 8 -12 215.568 52.2022 8

8 -4 -12 78.3424 46.5561 8

-4 -4 -12 220.157 56.2501 8

-4 8 -12 185.309 40.7872 11

-4 -4 -916955.40 1367.53 6

-4 8 -917478.20 1371.38 8

-4 8 -919569.80 1587.23 7

8 -4 -915541.20 1413.11 7

-4 -4 -916247.10 1369.77 8

8 -4 -917293.60 1367.64 8

4 -8 916211.30 1434.14 10

-8 4 916961.80 1436.45 10

4 -8 6 5139.23 507.902 10

-8 4 6 6278.50 533.43 10

-4 8 -6 4522.65 484.837 4

-4 8 -6 6326.24 697.225 7

-4 8 -3 5950.77 552.839 7

4 -8 3 5714.77 490.06 10

-8 4 3 5225.32 489.148 10

-4 8 -3 4153.79 594.807 4

-8 4 3 4497.13 500.507 4

4 4 3 6008.27 465.218 9

-4 8 -3 5044.27 498.507 9

-8 4 013762.60 1484.18 4

-4 8 016733.10 1427.41 7

-4 -4 017898.50 1439.43 10

4 -8 018088.80 1410.35 10

-4 8 016707.60 1504.37 4

4 4 018927.60 1450.32 9

-4 -4 364956.90 4789.40 10

4 4 -355202.90 4788.64 9

-4 8 360111.60 4785.43 4

-8 4 -356096.20 4801.90 4

-4 -4 626565.00 2258.13 10

4 4 -625236.50 2219.06 9

-4 8 627357.40 2208.56 4

4 4 -630057.20 2275.48 7

4 4 -916354.50 1262.15 9

4 -8 -915451.80 1223.75 8

-4 8 914555.70 1278.39 10

4 4 -914855.00 1218.66 8

-8 4 -914317.30 1225.24 8

4 4 -915370.80 1321.04 7

-8 4 -12 1747.74 183.684 14

4 4 -12 2203.92 185.478 14

4 -8 -12 2138.06 190.129 8

-8 4 -12 1963.33 181.884 11

-8 4 -12 1961.46 188.204 8

4 4 -12 1962.93 185.152 8

4 4 -12 1826.72 254.835 7

8 -4 12 1723.41 249.813 10

-4 8 12 2044.91 244.848 10

-8 4 -15 1029.76 101.811 11

-8 4 -15 1181.89 117.037 14

4 -8 -15 1031.60 113.42 8

-8 4 -15 1140.43 114.829 2

4 -8 -18 403.718 48.2618 8

-8 4 -18 433.36 50.4029 2

4 4 -18 467.028 50.4973 2

-5 8 -19 35.2538 18.5801 2

8 -3 -19 20.5809 16.8854 2

8 -3 -16 663.228 74.8437 8

-3 -5 -16 649.794 76.9075 8

-5 8 -16 721.505 78.2147 2

-5 8 -16 462.988 74.6837 14

-3 -5 -13 3402.24 301.121 8

8 -3 -13 3507.76 295.393 8

-5 8 -13 3391.71 288.841 11

-5 8 -13 3784.22 299.55 2

-5 8 -13 3275.60 292.568 14

-8 3 13 2501.64 341.346 10

5 -8 13 3051.54 355.931 10

3 5 13 3289.25 367.073 10

-5 8 -10 409.699 63.5062 14

-3 -5 -10 149.304 53.1668 14

-5 8 -10 335.446 175.085 7

8 -3 -10 326.641 88.5433 7

-3 -5 -10 210.884 53.1018 6

-3 -5 -10 249.891 58.5926 8

8 -3 -10 391.419 56.1611 8

-8 3 10 256.298 103.148 10

5 -8 10 420.302 114.333 10

-5 8 -713622.50 1195.66 7

-3 -5 -711648.60 926.591 1

5 -8 713029.90 1005.66 10

-8 3 713482.50 1026.66 10

-5 8 -7 9747.21 935.852 4

-8 3 413680.90 1034.63 10

5 -8 410290.70 1001.66 10

-5 8 -4 9733.37 1065.37 4

-8 3 413250.60 1016.46 4

-5 8 -151114.00 4185.31 7

5 -8 152616.50 4155.72 10

3 5 156535.90 4179.48 9

-8 3 144961.30 4223.24 4

-8 3 150456.90 4168.99 10

-3 -5 231196.80 2462.87 10

-5 8 228557.90 2489.43 4

-8 3 -230593.30 2510.86 4

3 5 -526545.40 2394.02 9

-3 -5 532324.10 2422.81 10

-5 8 529210.70 2381.54 4

3 5 -8 3544.06 334.381 9

3 5 -8 3792.42 401.318 7

-8 3 -8 2910.78 287.737 8

3 5 -8 3211.97 280.858 8

5 -8 -8 3405.72 286.003 8

-5 8 8 3371.10 339.757 10

-3 -5 8 3232.00 365.754 10

-8 3 -11 4456.23 420.274 8

3 5 -11 4163.54 495.293 7

3 5 -11 4639.46 417.087 8

8 -3 11 4833.20 496.365 10

-5 8 11 4644.07 473.492 10

-8 3 -11 4970.63 417.32 11

3 5 -11 5603.68 418.404 14

-8 3 -11 4938.37 418.925 14

5 -8 -11 5312.36 423.538 8

5 -8 -14 571.502 80.5736 8

8 -3 14 527.868 130.191 10

-5 8 14 342.193 105.932 10

-8 3 -14 634.381 68.1222 11

-8 3 -14 621.528 79.945 14

3 5 -14 557.106 77.4033 14

3 5 -14 577.265 75.2734 8

-8 3 -14 449.949 76.7028 8

5 -8 -17 8.59655 29.2864 8

3 5 -17 6.63967 29.8964 2

-8 3 -17 22.7273 29.6906 2

-2 -6 -17 348.275 49.9375 8

-6 8 -17 312.655 49.9375 2

-6 8 -14-9.35465 30.4552 11

-2 -6 -14 7.60151 46.907 8

8 -2 -14 -19.276 40.618 8

6 -8 14 13.5129 76.6425 10

2 6 14 53.7065 79.8689 10

-8 2 14 12.676 62.9543 10

-6 8 -14 88.4628 46.2114 2

-6 8 -14 40.1719 42.2183 14

8 -2 -1112413.50 1228.73 7

-6 8 -1114088.50 1183.26 14

-2 -6 -1115005.30 1183.61 6

-6 8 -1115261.80 1182.95 11

6 -8 1113696.90 1246.61 10

2 6 1113845.30 1258.37 10

-8 2 1112195.20 1238.61 10

-2 -6 -1115081.60 1188.61 8

8 -2 -1115214.30 1182.35 8

-8 2 8 7861.43 688.533 10

6 -8 8 7334.66 663.364 10

-6 8 -8 7458.94 813.33 7

8 -2 -8 6791.60 649.801 7

-2 -6 -8 7676.75 596.712 6

-2 -6 -8 7109.32 597.634 8

8 -2 -8 6823.88 591.702 8

6 -8 541354.30 3342.72 10

-8 2 547685.20 3385.06 10

-6 8 -540262.40 3417.84 7

-6 8 -536165.10 3350.53 4

-6 8 -2 408.27 258.076 4

-8 2 2 438.955 159.30 4

-6 8 -2 427.379 108.026 7

6 -8 2 518.669 88.392 10

-8 2 2 370.939 89.5168 10

2 6 2 625.586 93.8799 9

-6 8 1137215.010984.20 4

-2 -6 1136273.010916.00 10

2 6 -4 5876.67 606.462 9

-6 8 4 5901.44 579.558 4

8 -2 4 6287.04 520.339 9

6 -8 -7 5651.46 436.13 8

2 6 -7 6479.89 588.596 7

6 -8 -7 3740.27 433.848 7

6 -8 -7 6490.59 438.935 6

-2 -6 7 4717.05 515.261 10

-6 8 7 6043.04 499.443 10

2 6 -7 5040.58 496.962 9

2 6 -7 4660.74 452.564 4

-2 -6 1013254.40 1250.37 10

-6 8 1014070.60 1238.65 10

-8 2 -1013754.50 1181.24 14

2 6 -1014983.80 1180.24 14

2 6 -1014317.00 1288.60 7

2 6 -1014476.70 1181.63 8

-8 2 -1013227.20 1184.62 8

6 -8 -1015792.40 1186.87 8

-8 2 -1015597.00 1183.18 11

2 6 -13 969.287 103.041 8

-8 2 -13 864.377 105.042 8

-8 2 -13 1050.44 99.2424 11

-8 2 -13 901.794 104.602 14

2 6 -13 875.517 99.861 14

6 -8 -13 880.588 105.947 8

8 -2 13 929.451 180.385 10

-2 -6 13 937.017 161.561 10

-6 8 13 616.928 139.047 10

2 6 -13 660.627 150.565 7

-8 2 -16 1278.65 120.281 2

2 6 -16 1237.27 119.054 2

6 -8 -16 1104.00 117.892 8

2 6 -19 21.0774 17.1581 2

-7 8 -18 139.428 30.5638 2

-1 -7 -18 85.0279 26.7956 8

-1 -7 -18 136.56 27.646 2

8 -1 -18 123.848 28.2816 2

-1 -7 -15 308.031 60.9157 8

8 -1 -15 279.199 52.7458 8

-7 8 -15 268.112 54.5202 2

-7 8 -12 5414.22 439.248 11

-8 1 12 4236.78 495.208 10

1 7 12 5451.79 520.327 10

7 -8 12 4791.86 505.45 10

8 -1 -12 5471.02 440.422 8

-1 -7 -12 4794.12 439.624 6

-1 -7 -12 5632.37 448.067 8

8 -1 -9 3088.23 285.918 9

7 -8 9 2528.27 300.077 10

-8 1 9 2803.48 320.313 10

-7 8 -9 2527.96 244.391 8

8 -1 -9 2858.29 307.165 7

-1 -7 -9 2838.72 247.222 8

8 -1 -9 2682.24 237.706 8

-1 -7 -9 2684.57 241.327 6

1 7 623049.10 1868.70 4

-7 8 -619650.50 1881.26 4

-7 8 -623831.40 2015.26 7

-8 1 625293.20 1952.33 10

7 -8 625660.30 1913.16 10

-7 8 -342598.20 4044.03 4

1 7 350146.40 3940.94 9

-7 8 -346685.70 3970.82 7

7 -8 348264.90 3954.77 10

-8 1 352751.40 3965.52 10

-1 -7 0240109.019024.50 10

-7 8 0232272.019129.50 4

1 7 0238323.019035.30 9

-7 8 3 3586.02 391.32 4

1 7 -3 3716.59 403.07 9

-1 -7 3 3685.13 362.55 10

-7 8 3 4014.85 366.129 10

-7 8 6117298.0 7745.62 4

-7 8 6122587.0 7767.85 10

-1 -7 6115946.0 7803.23 10

1 7 -6141552.0 7930.27 7

7 -8 -674877.90 7704.15 7

1 7 -694224.20 7751.85 4

1 7 -685966.90 7777.82 9

-1 -7 910447.00 950.625 10

-7 8 910571.10 927.962 10

1 7 -910328.70 869.452 8

1 7 -911339.60 1010.27 7

-8 1 -910314.00 874.865 8

7 -8 -911048.70 871.592 6

7 -8 -910795.60 872.748 8

-8 1 -12 6.19545 37.4402 11

-8 1 -12 -10.771 47.924 14

1 7 -12 59.2453 40.7437 14

7 -8 -12 74.1198 48.8672 8

-8 1 -12 15.9583 43.7473 8

1 7 -12 51.018 45.5388 8

-7 8 12 62.1464 79.3505 10

-1 -7 12 112.558 88.63 10

8 -1 12-115.177 107.762 10

1 7 -12-93.2262 100.329 7

1 7 -15 1072.84 118.994 14

7 -8 -15 1118.70 119.739 8

-8 1 -15 1215.94 124.469 8

1 7 -15 1363.21 122.999 2

7 -8 -18 11.8864 21.3961 8

1 7 -18 13.4088 23.69 2

8 0 -19-16.7862 15.7301 2

0 -8 -16-42.5288 39.93 8

-8 8 -16 53.5136 37.1185 2

-8 8 -13 903.072 100.183 2

8 0 -13 841.906 94.2898 8

-8 0 13 636.479 136.461 10

0 8 13 749.138 148.669 10

8 -8 13 580.89 140.239 10

-8 8 -13 681.293 84.4799 11

0 -8 -13 932.62 100.203 8

-8 0 10-14.8008 88.1017 10

8 -8 10 66.1618 84.5279 10

8 0 -10 99.221 52.8841 9

8 0 -10 54.5637 81.2079 7

0 -8 -10 75.6362 52.0852 8

8 0 -10 101.83 41.7436 8

-8 8 -10-4.09527 49.5666 8

-8 8 -10-3.56377 41.2299 11

8 0 -10-3.33106 50.0965 14

0 -8 -10 79.9682 48.8182 6

0 -8 -7 3512.03 293.068 8

-8 8 -7 3153.68 316.666 4

-8 0 7 3547.27 389.777 10

8 -8 7 3596.95 356.863 10

0 -8 -7 3376.22 290.18 1

0 -8 -7 3197.75 288.922 6

0 8 7 3077.78 311.507 4

-8 8 -7 3495.58 296.977 8

-8 0 453366.50 3706.37 10

-8 8 -437197.50 3706.24 4

-8 0 126615.30 2081.95 10

0 8 124401.90 2078.99 9

0 8 -2 217.242 186.308 7

0 8 -2 219.302 114.088 9

-8 8 2 138.331 137.089 4

-8 8 2 193.297 47.9263 10

0 -8 2 345.238 86.5399 10

0 8 -5 6604.93 674.705 9

-8 8 5 6798.14 614.135 4

0 -8 5 7615.49 644.289 10

-8 8 5 7477.43 628.019 10

0 8 -5 5458.82 641.728 4

0 8 -8 345.065 111.475 9

8 -8 -8 388.203 61.2662 6

-8 0 -8 189.808 61.1851 8

0 8 -8 366.751 58.0089 8

8 -8 -8 250.299 57.9255 7

0 8 -8 504.786 188.065 7

8 -8 -8 243.167 57.178 8

-8 8 8 284.135 104.618 10

0 -8 8 451.789 136.203 10

0 -8 11 3821.18 410.305 10

-8 8 11 3718.50 399.582 10

8 0 11 4570.83 445.731 10

-8 0 -11 3516.65 342.158 14

0 8 -11 4305.02 343.637 14

0 8 -11 3836.64 452.557 7

-8 0 -11 3953.45 348.446 8

0 8 -11 4053.34 344.817 8

-8 0 -11 4236.55 343.517 11

8 -8 -11 3999.62 346.912 8

0 8 -14 464.586 64.4912 2

8 0 14 287.832 115.845 10

-8 8 14 244.453 90.4447 10

0 -8 14 304.068 102.748 10

8 -8 -14 340.811 61.8406 8

-8 0 -14 307.38 66.0453 8

-8 0 -14 305.311 50.2825 11

-8 0 -14 214.599 63.9277 14

0 8 -14 307.923 55.4877 14

-8 0 -17 438.465 54.7622 2

0 8 -17 459.93 56.8624 2

8 -8 -17 416.149 54.2122 8

9 -8 -19 45.1915 15.8245 2

-1 9 -19 32.7094 17.7007 2

9 -8 -16 42.8605 33.6147 8

-8 -1 -16-5.64515 38.4011 8

-8 -1 -16 65.0853 35.1498 2

-1 9 -16 16.1375 36.3858 2

-8 -1 -13 2148.96 193.775 8

-1 9 -13 1717.13 185.512 2

-8 -1 -13 2190.71 184.938 11

-9 8 13 1764.36 237.642 10

1 -9 13 1458.88 233.856 10

-8 -1 -13 2018.88 193.358 14

-1 9 -13 2272.75 188.821 14

9 -8 -13 2131.30 192.36 8

-8 -1 -10 2659.15 254.914 14

-1 9 -10 3567.63 260.169 14

9 -8 -10 2744.48 256.94 8

-8 -1 -10 2934.53 262.138 8

-1 9 -10 2964.40 257.347 8

-1 9 -10 2739.24 384.854 7

1 -9 10 2883.10 329.637 10

-9 8 10 2722.71 313.03 10

-8 -1 -10 2427.84 248.715 6

-8 -1 -10 3460.07 259.386 11

-1 9 -7 6022.61 726.189 9

9 -8 -7 7538.17 610.776 8

-9 8 7 8389.81 648.924 4

-9 8 7 8971.53 689.536 10

1 -9 7 8566.57 708.566 10

-8 -1 -7 6216.90 608.598 1

9 -8 -7 7384.68 610.734 6

-1 9 -7 6626.27 658.338 4

-1 9 -7 8139.19 612.719 8

9 -8 -7 7120.84 633.722 7

-1 9 -488154.20 6813.81 7

-1 9 -461078.80 6705.03 4

1 -9 489642.10 6648.73 10

-9 8 484267.20 6651.72 10

-9 8 489505.50 6659.49 4

-1 9 -489334.50 6709.68 9

8 1 480257.60 6591.34 9

-1 9 -152434.90 4046.40 7

-9 8 148627.00 4065.23 4

1 -9 148617.80 3976.07 10

-9 8 -289676.00 7242.02 4

-1 9 292858.60 7130.00 9

-1 9 287450.50 7089.13 7

-1 9 514506.00 1185.43 4

-8 -1 516656.10 1250.88 10

1 -9 -8 75.8057 51.0675 8

8 1 -8 137.83 100.96 7

1 -9 -8 16.4812 46.3981 6

9 -8 8 24.9028 93.9092 10

-8 -1 8 33.9686 126.852 10

-9 8 -8 72.957 52.1019 8

8 1 -8 10.8408 35.4066 8

8 1 -8 38.8546 69.0208 9

1 -9 -11 1708.18 173.988 8

-9 8 -11 1708.89 171.36 8

8 1 -11 1441.99 218.736 7

8 1 -11 1451.41 161.949 8

9 -8 11 2072.98 242.234 10

-1 9 11 1589.82 227.038 10

1 -9 -11 1520.47 166.251 6

-9 8 -11 2383.43 177.271 14

8 1 -11 2145.74 172.963 14

-8 -1 11 1352.11 225.769 10

-9 8 -11 1845.99 168.308 11

8 1 -14 45.062 38.7672 8

9 -8 14 40.233 77.335 10

-8 -1 14-26.1842 67.8028 10

-1 9 14 59.8786 73.3809 10

-9 8 -14 44.2802 40.2934 2

-9 8 -14-2.56379 33.2039 11

8 1 -17 431.318 51.2864 2

-9 8 -17 293.241 49.4706 2

1 -9 -17 397.699 52.0466 8

-7 -2 -17 776.596 77.5807 2

9 -7 -17 698.786 75.4927 8

-7 -2 -14 905.108 102.883 8

9 -7 -14 793.483 94.8074 8

-2 9 -14 832.713 94.4701 14

2 -9 14 501.029 131.414 10

-9 7 14 601.763 131.324 10

-2 9 -14 1078.25 100.58 2

-7 -2 -11 435.326 72.8193 8

-2 9 -11 457.644 68.8945 8

9 -7 -11 462.977 70.3963 8

-2 9 -11 330.00 158.449 7

-7 -2 -11 358.068 68.2575 14

-2 9 -11 518.277 69.8447 14

-7 -2 -11 478.199 64.6313 11

2 -9 11 522.596 127.478 10

-9 7 11 390.935 113.46 10

-2 9 -824357.60 1742.41 8

9 -7 -818843.30 1763.77 7

-2 9 -828416.60 2012.05 7

-7 -2 -820564.20 1734.25 6

9 -7 -821541.00 1738.11 6

2 -9 823255.80 1826.98 10

-9 7 823577.90 1811.76 10

-7 -2 -820879.00 1741.68 8

9 -7 -822425.90 1738.89 8

-2 9 -816124.50 1805.43 9

-2 9 -5 44.1278 148.554 9

-2 9 -5 41.0221 126.231 4

2 -9 5 197.777 106.241 10

-9 7 5 172.668 116.518 10

-9 7 5 216.731 98.0623 4

-2 9 -2158029.011638.90 7

-2 9 -2120820.011668.70 4

-2 9 -2147072.011581.80 9

7 2 2152867.011546.30 9

-9 7 2160252.011617.60 4

2 -9 2156703.011552.70 10

-9 7 2130606.011528.20 10

-2 9 113006.70 1107.32 7

-2 9 114708.70 1141.96 9

-2 9 415300.60 1206.21 4

-9 7 -412839.60 1232.94 4

2 -9 -713158.40 1127.72 8

-9 7 -712588.20 1132.38 8

2 -9 -715886.40 1130.81 1

7 2 -714878.50 1178.89 9

2 -9 -713477.50 1126.98 6

-2 9 714268.30 1162.42 4

7 2 -10 6102.66 480.144 14

-9 7 -10 5407.45 484.014 14

2 -9 -10 5690.77 482.258 6

7 2 -10 5679.84 559.548 7

7 2 -10 6117.51 479.916 8

-9 7 -10 5286.37 485.622 8

-9 7 -10 6025.83 482.832 11

-7 -2 10 5297.82 555.003 10

9 -7 10 5046.86 539.265 10

-2 9 10 5208.38 539.85 10

-9 7 -13 512.502 80.9822 8

7 2 -13 584.936 77.4371 8

-9 7 -13 706.395 86.9578 2

-9 7 -13 712.648 76.8172 11

-2 9 13 506.394 119.934 10

9 -7 13 439.784 127.144 10

-7 -2 13 357.642 117.115 10

-9 7 -13 732.155 85.188 14

7 2 -16 56.0342 34.4002 2

-9 7 -16 8.45879 35.6512 2

-9 7 -16-41.5292 45.9357 14

7 2 -19 50.5149 17.4164 2

-6 -3 -15 711.468 89.6226 8

9 -6 -15 714.657 84.7134 8

-3 9 -15 693.749 82.1891 14

-6 -3 -12 3243.59 248.753 11

3 -9 12 2003.44 299.082 10

-9 6 12 1915.66 294.48 10

-6 -3 -12 2916.52 256.511 8

9 -6 -12 2799.05 251.253 8

-6 -3 -12 2696.00 253.341 14

-3 9 -12 2975.37 250.971 14

3 -9 9 414.953 127.536 10

-9 6 9 472.237 118.109 10

-6 -3 -9 294.668 55.715 6

-3 9 -9 383.461 186.05 7

9 -6 -9 276.212 70.8348 7

-3 9 -9 285.132 58.932 8

9 -6 -9 349.831 58.7888 8

-6 -3 -9 372.012 63.3979 8

-9 6 611991.10 880.245 4

-3 9 -6 9094.01 902.792 4

-3 9 -611481.70 1127.02 7

-9 6 611611.70 920.527 10

3 -9 6 9733.26 901.724 10

-3 9 -3 5037.57 576.726 9

6 3 3 6757.71 536.974 9

-3 9 -3 4484.26 694.606 4

-3 9 -3 7124.30 669.255 7

3 -9 3 6983.68 566.998 10

-9 6 3 6085.41 558.034 10

-9 6 3 6682.41 599.74 4

-6 -3 0 409.364 98.1738 10

3 -9 0 248.975 59.6402 10

-3 9 0 464.916 84.5769 7

-9 6 0 314.726 200.972 4

-3 9 0 270.73 171.925 4

6 3 0 342.416 95.0379 9

-3 9 0 353.178 95.8906 9

-3 9 3 1377.77 183.125 4

-9 6 -3 1273.73 230.703 4

-3 9 619785.20 1605.25 4

-9 6 -619708.50 1614.48 4

6 3 -618594.10 1610.00 9

3 -9 -9 2480.10 200.739 6

6 3 -9 1421.01 249.09 7

6 3 -9 2305.10 231.285 9

-3 9 9 2058.86 250.472 10

9 -6 9 1938.02 254.568 10

-9 6 -9 1932.85 200.629 8

6 3 -9 2115.44 191.327 8

6 3 -12 2560.01 233.528 14

-9 6 -12 2480.80 233.584 14

6 3 -12 2073.20 290.213 7

-9 6 -12 2369.63 236.914 8

6 3 -12 2876.63 235.158 8

3 -9 -12 3138.41 240.116 6

-9 6 -12 2728.99 232.754 11

-3 9 12 2483.44 292.429 10

-6 -3 12 2100.53 288.543 10

9 -6 12 2249.26 299.192 10

-9 6 -15 219.557 56.1459 14

-9 6 -15 294.152 54.8384 2

-9 6 -15 249.40 41.2628 11

6 3 -18 15.4536 22.8791 2

-9 6 -18 11.4377 22.6605 2

9 -5 -19 24.6116 16.161 2

-4 9 -19 13.7845 17.6312 2

-4 9 -16 361.271 59.6138 14

9 -5 -16 430.356 57.2364 8

-5 -4 -16 339.824 57.4271 8

-4 9 -13 9405.20 743.648 14

9 -5 -13 9045.04 745.242 8

-5 -4 -13 9891.05 751.525 8

4 -9 13 7873.94 804.623 10

-9 5 13 7149.40 793.271 10

5 4 13 8638.79 824.656 10

-4 9 -10 57.3864 44.4082 8

-5 -4 -10 119.172 49.9359 8

9 -5 -10 98.8271 42.4076 8

-5 -4 -10 53.441 40.2494 14

-4 9 -10 115.859 48.3867 14

-5 -4 -10 132.058 41.0769 6

4 -9 10 135.35 87.8347 10

-9 5 10 79.1646 90.9241 10

-4 9 -10 28.8243 146.401 7

9 -5 -10 86.6086 49.3747 7

-5 -4 -711614.00 892.895 1

-4 9 -7 8890.32 917.07 4

-4 9 -710640.70 892.58 8

-4 9 -713798.20 1219.04 7

4 -9 710924.30 965.867 10

-9 5 713293.80 990.036 10

5 4 454700.90 4467.59 9

-4 9 -450186.30 4534.95 9

-4 9 -448599.70 4604.97 4

-9 5 459880.30 4519.17 4

-4 9 -460622.30 4632.98 7

4 -9 456072.90 4508.28 10

-9 5 460740.70 4530.23 10

-9 5 163977.30 5276.78 4

5 4 167369.20 5214.94 9

-9 5 161422.60 5196.09 10

-4 9 -165467.60 5223.65 7

4 -9 166592.40 5193.17 10

-5 -4 2 1086.84 141.983 10

-4 9 2 928.769 174.58 4

-9 5 -2 655.427 197.507 4

-4 9 5 2136.97 233.597 4

-5 -4 5 1767.90 264.246 10

5 4 -5 1913.47 238.389 9

-9 5 -5 2115.96 243.713 4

5 4 -8 5855.29 491.074 8

-9 5 -8 5917.15 503.138 8

5 4 -8 6516.94 595.841 7

-4 9 8 5823.98 550.092 10

5 4 -8 6445.67 540.864 9

4 -9 -8 5681.15 495.664 8

5 4 -11 8137.22 798.559 7

-9 5 -11 8440.04 722.233 8

5 4 -11 8376.38 715.234 8

5 4 -11 9199.94 716.822 14

-9 5 -11 8790.01 718.463 14

-9 5 -11 9401.11 718.531 11

4 -9 -11 8583.20 718.259 6

4 -9 -11 8300.98 720.051 8

-4 9 11 8499.22 777.795 10

9 -5 11 8137.07 790.279 10

4 -9 -14 443.912 71.3896 8

5 4 -14 414.076 66.7432 8

-9 5 -14 509.005 73.1695 8

-9 5 -14 495.805 72.0264 14

-9 5 -14 516.039 72.9516 2

-9 5 -14 572.791 64.9377 11

9 -5 14 497.74 120.327 10

-5 -4 14 359.01 107.309 10

-4 9 14 394.621 101.386 10

5 4 -17 33.8875 30.3362 2

-9 5 -17 11.2417 27.7722 2

4 -9 -17 6.37111 28.7848 8

-5 9 -17 640.45 72.8954 2

-4 -5 -17 626.644 71.5247 8

9 -4 -17 667.818 69.6721 8

-4 -5 -14 175.127 60.3034 8

9 -4 -14 225.21 53.5529 8

-9 4 14 247.131 84.4043 10

5 -9 14 93.0584 81.7992 10

4 5 14 113.161 83.0591 10

-5 9 -14 247.544 53.6394 14

-4 -5 -11 571.096 90.1831 14

-5 9 -11 732.681 93.5064 14

-4 -5 -11 914.573 101.735 8

9 -4 -11 762.418 89.3584 8

-5 9 -11 751.288 94.4636 8

-5 9 -11 879.332 91.3696 11

-4 -5 -11 953.608 87.5392 11

-4 -5 -11 833.434 91.3462 6

4 5 11 711.172 159.44 10

5 -9 11 513.635 135.461 10

-9 4 11 579.055 138.614 10

-5 9 -8 6314.81 728.412 7

9 -4 -8 6085.26 528.563 7

-5 9 -8 5530.00 478.12 8

-4 -5 -8 6068.48 480.861 1

5 -9 8 5750.23 546.181 10

-9 4 8 5820.40 559.65 10

9 -4 -8 5664.95 473.023 8

-4 -5 -8 5524.45 477.297 8

-4 -5 -8 5504.02 474.165 6

5 -9 5 2125.76 231.221 10

-5 9 -5 1494.34 258.90 4

-9 4 5 1402.82 230.222 10

-9 4 218967.60 1349.42 4

-5 9 -214846.10 1300.82 7

4 5 215690.00 1266.63 9

-5 9 -214513.60 1442.43 4

-9 4 215168.70 1267.59 10

5 -9 214794.00 1261.63 10

4 5 -1 9301.23 836.189 9

-5 9 1 9368.65 872.003 4

-4 -5 1 9965.49 817.89 10

-5 9 4 3767.84 383.305 4

4 5 -4 4207.15 401.99 9

-9 4 -4 3497.20 392.622 4

-4 -5 4 3977.20 394.018 10

4 5 -712436.60 987.73 9

5 -9 -711364.50 930.61 8

-5 9 712363.20 963.568 4

-4 -5 711005.10 1019.52 10

-5 9 710506.70 977.882 10

4 5 -7 9906.99 948.787 4

4 5 -713180.50 1046.37 7

5 -9 -711708.30 931.299 6

-9 4 -1013669.10 1228.89 8

4 5 -1015465.70 1324.24 7

4 5 -1015093.70 1223.59 8

9 -4 1013849.70 1296.61 10

-5 9 1014261.70 1280.53 10

5 -9 -1014930.00 1227.94 8

4 5 -1016319.10 1223.72 14

-9 4 -1014596.70 1225.88 14

-9 4 -1016004.00 1226.24 11

-9 4 -13 1010.69 115.49 8

4 5 -13 929.054 110.01 8

4 5 -13 760.372 160.267 7

-5 9 13 892.474 155.397 10

9 -4 13 1066.49 178.123 10

-9 4 -13 1077.77 115.46 14

4 5 -13 1102.40 112.457 14

5 -9 -13 1110.49 119.968 8

-9 4 -13 1143.56 108.814 11

4 5 -16 745.316 79.4271 2

-9 4 -16 578.084 76.43 2

5 -9 -16 681.621 79.1457 8

5 -9 -19 75.2151 17.5942 2

4 5 -19 71.8162 20.2488 2

9 -3 -18 75.0757 25.076 2

-6 9 -18 66.2445 25.7655 2

-6 9 -15 70.912 42.7406 2

-3 -6 -15 217.38 55.5473 8

9 -3 -15 150.732 43.7386 8

-6 9 -15 127.185 45.1482 14

-3 -6 -12 807.584 96.3438 8

9 -3 -12 746.659 87.1974 8

-6 9 -12 571.384 83.8821 14

-6 9 -12 659.121 80.8747 11

6 -9 12 408.899 132.174 10

-9 3 12 676.966 138.717 10

3 6 12 795.985 164.46 10

-6 9 -12 744.429 91.3094 2

-3 -6 -9 5055.24 419.318 6

6 -9 9 5056.82 487.059 10

-9 3 9 4912.02 493.702 10

-3 -6 -9 5100.84 422.546 8

9 -3 -9 4966.84 415.723 8

9 -3 -9 4328.40 464.168 7

-6 9 -9 4357.05 608.287 7

-6 9 -6 2734.15 463.457 7

6 -9 6 3208.55 349.869 10

-6 9 -6 3062.14 343.232 4

-6 9 -340239.00 3423.25 7

-6 9 -342540.80 3556.88 4

-9 3 350299.10 3438.94 4

3 6 341550.10 3372.13 9

6 -9 337094.90 3381.14 10

-9 3 344520.80 3398.30 10

-3 -6 038766.00 3072.04 10

3 6 037754.90 3079.07 9

-9 3 033500.40 3130.92 4

-6 9 042557.20 3188.45 4

-9 3 -313479.10 1033.75 4

-6 9 312226.10 1008.66 4

3 6 -3 9393.66 996.439 9

-3 -6 311545.70 983.363 10

-6 9 6 394.932 104.741 4

3 6 -6 342.497 115.128 9

3 6 -6 228.287 144.351 7

-3 -6 6 388.959 139.09 10

-6 9 6 261.843 93.4203 10

6 -9 -915700.80 1269.11 8

3 6 -916550.10 1386.30 7

3 6 -915617.50 1265.81 8

-9 3 -914906.60 1273.49 8

6 -9 -915790.90 1268.38 6

-3 -6 916040.90 1356.17 10

-6 9 914838.00 1321.04 10

3 6 -916532.80 1320.03 9

-9 3 -12 16.0514 38.3375 11

3 6 -12-17.5391 85.1817 7

3 6 -12-3.15054 39.2846 14

-9 3 -12 35.2898 50.2366 14

-3 -6 12 14.2242 84.6081 10

9 -3 12 63.7257 90.3527 10

-6 9 12 12.3807 72.5187 10

-9 3 -12-8.18219 48.8546 8

3 6 -12-23.3177 40.9588 8

6 -9 -12 26.6815 51.461 8

-9 3 -15 2453.44 251.993 14

-9 3 -15 2776.57 244.624 11

6 -9 -15 2848.56 253.066 8

3 6 -15 3423.31 256.791 2

-9 3 -15 3074.77 256.257 2

6 -9 -18 41.5528 24.2868 8

3 6 -18 33.8239 24.1813 2

9 -2 -19-7.68964 15.9359 2

-7 9 -16 372.407 64.9186 14

9 -2 -16 492.873 60.556 8

-2 -7 -16 502.189 66.5038 8

-7 9 -16 498.81 65.1837 2

-2 -7 -13 1194.81 127.044 8

9 -2 -13 1186.83 120.663 8

-7 9 -13 1193.44 119.458 14

7 -9 13 748.515 165.26 10

-9 2 13 1013.42 173.147 10

2 7 13 1140.30 184.847 10

-7 9 -13 1029.97 112.873 11

-7 9 -13 1363.72 126.411 2

7 -9 10 52.5389 89.4255 10

-9 2 10 28.8899 92.4361 10

9 -2 -10 52.9374 55.96 9

-7 9 -10 22.9728 46.3651 14

-7 9 -10 49.9948 41.3721 11

9 -2 -10 48.8571 68.4259 7

9 -2 -10 182.714 43.9199 8

-2 -7 -10 89.8016 46.9154 8

-2 -7 -10 125.258 46.1787 6

-9 2 7 66.8337 138.773 10

7 -9 7 185.649 110.704 10

-7 9 -7 46.2214 63.1168 4

-2 -7 -7 175.036 51.353 8

-2 -7 -7 127.247 46.1058 6

-7 9 -7 71.2348 208.867 7

-2 -7 -7 164.366 50.4082 1

-7 9 -7 116.568 49.4336 8

2 7 7 126.273 57.2831 4

-7 9 -415900.80 1585.91 4

7 -9 417648.70 1509.00 10

-9 2 420012.90 1534.94 10

2 7 140474.30 3083.89 9

-9 2 136695.30 3144.30 4

-9 2 138466.40 3078.21 10

7 -9 137358.30 3047.41 10

-7 9 2 7759.14 700.452 4

-9 2 -2 7295.95 706.889 4

-7 9 2 7585.46 624.378 10

-2 -7 2 6802.41 635.675 10

-2 -7 531994.30 2472.01 10

-7 9 530751.20 2438.03 10

-7 9 530424.30 2446.77 4

2 7 -525437.10 2465.11 9

2 7 -8 9474.76 824.982 7

2 7 -8 8765.03 676.926 8

-9 2 -8 7330.38 682.671 8

7 -9 -8 8220.55 677.351 6

-2 -7 8 8200.93 765.409 10

-7 9 8 7858.64 728.349 10

7 -9 -8 8015.57 677.788 8

2 7 -8 9497.41 756.341 9

-9 2 -1111599.00 883.144 11

7 -9 -1110657.70 884.337 8

-9 2 -1111013.40 882.863 14

2 7 -1111750.60 881.36 14

2 7 -11 9726.35 980.222 7

-9 2 -11 9858.79 885.835 8

2 7 -1110428.20 880.88 8

-7 9 1110264.60 939.103 10

9 -2 1110673.80 969.532 10

-2 -7 11 9018.86 942.142 10

7 -9 -14 41.0604 47.9154 8

-9 2 -14 14.7956 48.692 8

2 7 -14 157.90 49.8348 2

-9 2 -14 10.4517 53.3642 14

2 7 -14 98.3089 42.7209 14

-7 9 14 57.3907 71.4767 10

-2 -7 14 26.5893 79.2289 10

9 -2 14 59.739 91.6165 10

-9 2 -14 58.5934 34.1329 11

-9 2 -17 57.8258 29.6564 2

2 7 -17 122.649 32.4224 2

7 -9 -17 61.3259 29.7543 8

9 -1 -17 705.645 73.1883 2

-8 9 -17 680.935 73.721 2

-1 -8 -17 657.239 73.1825 8

9 -1 -14 338.269 57.6925 8

-1 -8 -14 387.345 66.9748 8

-8 9 -14 286.256 48.7589 11

-8 9 -14 398.177 63.54 2

8 -9 14 304.124 105.722 10

-9 1 14 244.873 91.3092 10

1 8 14 303.706 98.0171 10

1 8 11 4901.02 538.194 10

8 -9 11 5021.65 535.038 10

-9 1 11 5274.72 541.682 10

-1 -8 -11 6152.07 477.423 6

-8 9 -11 6110.04 477.072 11

-1 -8 -11 6180.78 482.334 8

-8 9 -11 5122.44 476.791 8

9 -1 -11 4970.21 525.484 7

9 -1 -11 5522.25 472.285 8

-9 1 8 8781.29 783.453 10

8 -9 8 9104.42 755.112 10

-8 9 -8 7738.57 689.719 8

9 -1 -8 8018.61 745.854 7

-1 -8 -8 8196.41 685.175 6

-1 -8 -8 8160.62 687.939 8

9 -1 -8 8215.97 680.896 8

-1 -8 -8 9103.09 689.375 1

-9 1 5 6085.06 563.574 10

8 -9 5 5874.66 528.705 10

-8 9 -5 4843.97 546.279 4

8 -9 286636.60 7008.62 10

-9 1 288445.50 7021.88 10

-8 9 -277706.90 7160.52 4

1 8 290196.90 7018.35 9

-1 -8 1 7176.76 606.38 10

-8 9 1 6453.40 697.607 4

-8 9 4 2462.28 294.679 4

-8 9 4 2489.72 277.863 10

-1 -8 4 2889.02 296.622 10

1 8 -4 2571.15 380.024 7

1 8 -4 2249.95 320.425 9

9 -1 4 2654.44 228.396 9

1 8 -7 147.154 43.0336 8

8 -9 -7 65.9154 42.5699 6

-1 -8 7 204.714 141.319 10

-8 9 7 32.2615 78.9363 10

1 8 -7 16.851 115.874 9

8 -9 -7 66.069 44.6603 8

1 8 -7 57.347 56.2653 4

-9 1 -7 91.7336 46.2257 1

1 8 -7 126.018 160.969 7

8 -9 -7 47.2606 33.6636 7

-8 9 7-15.0021 82.0217 4

1 8 -10 1380.88 244.652 7

1 8 -10 1614.48 137.676 14

-9 1 -10 1202.42 134.779 14

8 -9 -10 1572.83 142.175 8

-8 9 10 1358.00 183.792 10

-9 1 -10 1082.41 138.342 8

1 8 -10 1512.38 138.60 8

8 -9 -10 1538.01 140.894 6

-1 -8 10 1182.68 200.164 10

-9 1 -10 1224.96 135.603 11

-9 1 -13 1325.11 143.137 8

-1 -8 13 979.506 180.908 10

-8 9 13 976.755 174.993 10

9 -1 13 1297.02 209.859 10

-9 1 -13 1616.32 136.585 11

1 8 -13 1171.11 204.26 7

8 -9 -13 1435.42 141.097 8

-9 1 -13 1332.62 140.318 14

1 8 -13 1465.45 135.475 14

8 -9 -16 2.55221 33.9379 8

-9 1 -16 63.4459 36.1059 2

1 8 -16 34.4417 34.7924 2

1 8 -19 27.6593 17.2414 2

0 -9 -18 127.704 28.2123 8

9 0 -18 117.954 27.1992 2

-9 9 -18 148.905 30.3778 2

-9 9 -15 2542.38 225.526 2

9 0 -15 2497.58 221.475 8

0 -9 -15 2769.68 228.545 8

-9 9 -15 2392.53 215.911 11

-9 9 -12 376.09 57.0088 11

0 -9 -12 344.369 61.7369 6

0 -9 -12 331.745 66.3618 8

9 0 -12 203.795 53.9035 8

-9 9 -12 207.603 63.9148 2

0 9 12 315.92 108.076 10

9 -9 12 274.777 107.16 10

-9 0 12 413.717 111.077 10

0 -9 -9 46.5248 46.9365 8

9 0 -9-13.4384 81.304 7

9 -9 9 103.175 94.5711 10

-9 0 9 65.2873 108.34 10

0 -9 -9 51.5091 44.5755 6

9 0 -9 194.727 75.6232 9

-9 9 -9 107.825 54.3977 8

9 0 -9 53.6163 39.1153 8

0 9 682845.60 6646.12 4

-9 9 -671640.30 6661.26 4

9 -9 687831.20 6674.93 10

-9 0 696631.90 6733.77 10

-9 0 376884.80 4667.60 10

-9 9 -366679.30 4782.34 4

0 -9 017461.70 1438.16 10

0 9 017656.10 1456.04 9

-9 9 016974.60 1572.71 4

-9 9 3 840.368 185.479 4

0 9 -3 766.116 247.656 7

0 9 -3 773.593 166.913 9

9 0 3 1018.47 109.562 9

-9 9 3 586.912 113.067 10

0 -9 3 1003.77 139.813 10

-9 9 623233.20 1778.69 10

0 -9 622047.70 1799.23 10

-9 9 624622.30 1769.48 4

0 9 -619327.70 1785.00 4

0 9 -9 3347.86 392.49 7

0 9 -9 2424.37 227.147 8

0 -9 9 2763.70 314.986 10

-9 9 9 2443.08 281.777 10

-9 0 -9 2215.91 218.958 6

9 -9 -9 2757.05 230.616 6

9 -9 -9 2788.00 232.027 8

-9 0 -9 2751.65 236.936 8

0 -9 12 114.252 91.1957 10

9 0 12 137.406 106.281 10

-9 9 12 183.972 94.523 10

0 9 -12 261.789 52.323 14

-9 0 -12 141.648 55.1379 14

-9 0 -12 190.849 60.6368 8

9 -9 -12 235.405 58.5826 8

-9 0 -12 189.534 45.9206 11

0 9 -12 40.5954 114.172 7

0 9 -15 536.557 69.6547 14

0 9 -15 488.942 70.104 2

-9 0 -15 482.644 71.5129 8

9 -9 -15 512.963 70.9378 8

0 9 -18 107.832 27.8243 2

9 -9 -18 66.0702 23.4438 8

10 -9 -17 85.1473 28.3875 8

-1 10 -17 51.394 29.7646 2

-1 10 -17 69.6804 27.5931 3

-1 10 -14 846.451 98.2173 2

10 -9 -14 808.72 95.1565 8

-10 9 14 634.562 127.849 10

1 -10 14 801.154 146.112 10

-1 10 -14 1027.92 96.1236 14

-9 -1 -14 787.021 100.04 8

-9 -1 -11 741.745 80.307 11

10 -9 -11 720.866 83.4593 8

-9 -1 -11 613.363 85.4116 8

-1 10 -11 517.413 160.915 7

-9 -1 -11 365.042 75.6848 14

-1 10 -11 464.737 73.2553 14

-10 9 11 417.63 124.247 10

1 -10 11 456.028 130.467 10

-1 10 -11 873.73 86.1753 2

-9 -1 -819140.70 1397.97 1

10 -9 -817778.60 1393.10 6

-10 9 818718.40 1459.96 10

1 -10 817963.60 1487.28 10

-9 -1 -817203.90 1400.60 8

10 -9 -817678.00 1393.06 8

-1 10 -814073.20 1482.88 9

-1 10 -823795.00 1653.17 7

10 -9 -813071.00 1402.06 7

-1 10 -5 637.816 199.328 4

1 -10 5 1033.90 163.037 10

-10 9 5 490.073 143.177 10

-1 10 -5 756.245 228.171 9

9 1 5 785.313 84.4315 9

-10 9 5 681.768 147.001 4

-10 9 245132.80 3356.87 4

1 -10 247431.00 3282.15 10

-1 10 -251547.30 3416.03 7

-1 10 -248728.60 3334.07 9

9 1 245925.00 3279.12 9

-1 10 163595.70 5237.85 7

-9 -1 166213.50 5258.82 10

-1 10 166812.10 5259.60 9

-10 9 -4 969.815 226.313 4

-1 10 4 1252.95 177.572 4

1 -10 -7 242.178 51.9797 6

-10 9 -7 174.05 57.104 8

10 -9 7 96.8001 99.4995 10

-9 -1 7 112.387 144.962 10

-10 9 -7 219.029 62.1205 2

1 -10 -7 251.967 57.6718 8

-10 9 -7 88.8823 81.875 4

-1 10 7 302.352 86.2197 4

1 -10 -7 333.926 55.953 1

-10 9 -10 1332.17 131.539 11

9 1 -10 1095.85 123.589 14

-10 9 -10 1202.15 134.281 14

10 -9 10 1056.91 180.358 10

-9 -1 10 1245.55 209.443 10

1 -10 -10 1170.53 131.426 8

-10 9 -10 1278.93 141.363 2

1 -10 -10 1238.23 128.614 6

-10 9 -10 1195.93 133.411 8

9 1 -10 1339.92 124.856 8

9 1 -10 1400.88 198.162 7

9 1 -10 1418.37 165.737 9

-10 9 -13 583.39 83.7639 2

-10 9 -13 672.646 76.1123 11

9 1 -13 667.86 78.9738 8

1 -10 -13 677.354 87.5911 8

-1 10 13 429.858 118.622 10

10 -9 13 422.92 124.05 10

-9 -1 13 586.322 131.349 10

1 -10 -16 318.178 53.6257 8

-10 9 -16 277.092 38.9167 11

-10 9 -16 322.665 52.212 2

9 1 -16 387.93 52.5692 2

-2 10 -18 4.9237 23.9856 2

10 -8 -18 13.2887 20.0023 8

10 -8 -15 142.556 44.9352 8

-8 -2 -15 124.344 48.7007 8

-2 10 -15 144.369 46.6824 2

-2 10 -15 86.0926 42.3662 14

10 -8 -12 6793.43 562.359 8

-8 -2 -12 6821.31 567.934 8

-2 10 -12 6108.84 671.132 7

-8 -2 -12 6255.72 562.617 14

-2 10 -12 7248.41 561.347 14

-8 -2 -12 7589.71 561.542 11

2 -10 12 5569.44 620.281 10

-10 8 12 5547.60 612.282 10

-2 10 -12 6521.02 562.257 2

2 -10 911142.80 968.464 10

-10 8 911407.10 951.604 10

-2 10 -911030.60 885.564 8

-2 10 -912642.50 1101.58 7

10 -8 -910831.10 884.044 6

-8 -2 -910210.70 879.41 6

-8 -2 -910771.20 889.621 8

10 -8 -910987.80 884.637 8

-10 8 610266.20 838.35 10

2 -10 6 8876.67 830.586 10

-2 10 -6 8546.87 840.464 4

-10 8 611063.90 813.566 4

-10 8 3 3036.94 294.847 10

2 -10 3 3752.32 318.517 10

-10 8 3 3353.30 359.927 4

8 2 3 3154.72 282.902 9

-2 10 -3 2847.35 348.592 9

-2 10 -3 2686.51 417.71 7

2 -10 0 6813.09 541.057 10

-2 10 0 6077.41 555.341 7

-2 10 0 6338.65 565.46 9

-10 8 -313147.50 1267.35 4

-2 10 314645.20 1204.41 4

-8 -2 6 212.926 143.084 10

-10 8 -6 282.057 102.164 4

-2 10 6 101.544 105.32 4

2 -10 -9 2795.91 250.451 8

2 -10 -9 2631.82 247.742 6

-10 8 -9 2503.16 253.049 8

8 2 -9 3545.00 337.748 7

8 2 -9 2833.71 242.624 8

-8 -2 9 2207.89 324.553 10

10 -8 9 2708.39 309.052 10

8 2 -9 3372.06 295.861 9

-2 10 12 234.192 97.7444 10

10 -8 12 421.431 112.786 10

-8 -2 12 116.516 96.4531 10

8 2 -12 323.884 59.6258 8

-10 8 -12 338.139 63.1387 14

-10 8 -12 333.523 68.1523 2

2 -10 -12 459.02 67.7071 6

-10 8 -12 263.161 55.9975 11

8 2 -12 407.345 107.001 7

-10 8 -15 1207.06 114.115 2

-10 8 -15 1035.41 102.085 11

-10 8 -15 1010.67 110.276 14

8 2 -18 231.464 34.1193 2

10 -7 -19-11.1183 15.6421 2

-3 10 -16 195.006 48.8074 14

-7 -3 -16 316.548 53.4793 8

10 -7 -16 308.887 49.7839 8

3 -10 13-27.3393 87.3686 10

-10 7 13 62.247 75.3005 10

10 -7 -13 47.5877 48.2143 8

-7 -3 -13 15.8352 50.905 8

-3 10 -13 110.087 45.6711 14

-3 10 -13 75.0872 52.2925 2

-7 -3 -10 1649.44 161.191 8

10 -7 -10 1863.79 159.739 8

-3 10 -10 1677.78 157.344 8

-7 -3 -10 1191.98 150.652 14

-3 10 -10 1343.64 154.291 14

3 -10 10 1576.07 224.89 10

-10 7 10 1750.15 218.928 10

-7 -3 -10 1899.18 156.519 6

-3 10 -10 1499.06 295.075 7

-7 -3 -10 1929.17 157.816 11

10 -7 -7 2258.77 194.591 8

-3 10 -7 2983.01 470.876 7

3 -10 7 2228.46 278.766 10

-10 7 7 2406.46 275.369 10

-3 10 -7 2137.30 255.165 4

-3 10 -7 1164.64 282.008 9

10 -7 -7 2573.46 198.067 6

-3 10 -7 1789.86 192.645 8

-3 10 -472610.10 6041.25 9

7 3 471526.10 5946.86 9

-3 10 -461374.30 6110.53 4

-10 7 479688.30 6008.19 4

-3 10 -486039.20 6181.66 7

-10 7 478579.40 6005.93 10

3 -10 476422.50 5990.99 10

-3 10 -119948.00 1741.41 7

7 3 120803.00 1725.24 9

3 -10 121515.50 1704.78 10

-10 7 121220.50 1800.29 4

-10 7 -224776.80 2127.86 4

-3 10 224909.30 2068.03 4

-3 10 5 209.488 100.731 4

7 3 -5 294.192 95.1421 9

7 3 -824498.20 1884.04 9

-10 7 -821995.00 1843.59 8

7 3 -824132.70 1922.85 7

7 3 -822511.50 1831.13 8

7 3 -11 1105.28 120.734 8

-10 7 -11 1266.41 132.577 8

7 3 -11 1153.57 122.115 14

-10 7 -11 1256.71 127.989 14

7 3 -11 1110.26 186.051 7

-7 -3 11 1019.56 187.452 10

-3 10 11 1029.35 172.66 10

10 -7 11 1215.49 191.597 10

-10 7 -11 1264.00 124.411 11

3 -10 -11 1192.61 127.915 6

-10 7 -14 114.021 45.6728 2

7 3 -14 88.2724 41.1947 8

-7 -3 14 52.7516 71.1387 10

-3 10 14 11.3872 63.7009 10

10 -7 14 27.2885 78.8261 10

-10 7 -14 78.6857 49.3673 14

-10 7 -14 39.4159 33.9332 11

-10 7 -17 174.389 37.3059 2

7 3 -17 204.66 38.1121 2

-6 -4 -17 141.50 35.9393 8

10 -6 -17 116.195 32.0716 8

-4 10 -14 1449.02 145.058 14

-10 6 14 1052.63 182.006 10

10 -6 -14 1650.85 149.456 8

-6 -4 -14 1781.32 155.122 8

6 4 14 1481.30 211.925 10

4 -10 14 1321.41 196.949 10

-6 -4 -11 281.064 54.883 6

-6 -4 -11 305.427 49.7529 11

-6 -4 -11 282.296 61.2136 8

10 -6 -11 298.689 56.5254 8

-6 -4 -11 178.604 55.6715 14

-4 10 -11 369.493 61.1024 14

-4 10 -11 208.516 146.933 7

-10 6 11 197.286 94.8605 10

4 -10 11 181.699 103.586 10

-4 10 -11 253.746 51.2303 11

-4 10 -819524.30 1794.22 7

10 -6 -817032.30 1549.16 7

-4 10 -818366.20 1515.87 8

-10 6 820376.00 1600.31 10

4 -10 819724.20 1593.85 10

-6 -4 -819682.90 1514.01 6

-6 -4 -818110.60 1516.75 8

10 -6 -818749.10 1512.73 8

-4 10 -5 154.875 166.836 9

-4 10 -5 23.4794 159.478 4

-10 6 5 105.94 118.389 10

4 -10 5 47.4184 95.1776 10

-10 6 5 10.5185 94.4195 4

-10 6 2 2157.46 283.876 4

-4 10 -2 2004.00 255.184 7

4 -10 2 2155.47 201.007 10

6 4 2 2175.25 202.839 9

-4 10 -2 1585.23 232.975 9

-10 6 2 1868.18 191.465 10

-6 -4 1189392.014561.50 10

6 4 -1165846.014568.60 9

-4 10 1186738.014612.10 4

-4 10 1184564.014533.90 7

-4 10 4 6566.43 579.806 4

-10 6 -4 5540.33 592.632 4

6 4 -4 6629.39 583.78 9

6 4 -781689.50 6189.52 7

-4 10 775716.40 6136.53 4

6 4 -773727.60 6142.50 9

-10 6 -10 1222.74 141.38 8

6 4 -10 1029.38 126.072 8

10 -6 10 1375.01 204.971 10

-4 10 10 968.576 175.771 10

6 4 -10 1552.08 130.738 14

-10 6 -10 1811.43 145.061 14

6 4 -10 1010.67 196.267 7

-10 6 -10 1212.40 130.838 11

4 -10 -13 6099.98 504.097 8

6 4 -13 5984.22 498.465 8

-10 6 -13 5485.47 501.559 8

-10 6 -13 6292.72 507.191 2

6 4 -13 6981.39 504.147 14

-10 6 -13 6128.80 500.283 14

-10 6 -13 6355.71 498.585 11

-6 -4 13 4321.73 548.228 10

10 -6 13 4539.03 556.196 10

-4 10 13 4722.04 543.006 10

6 4 -16 478.497 56.8474 2

-10 6 -16 389.389 55.6577 2

-10 6 -16 239.289 54.2214 14

-10 6 -16 351.129 43.78 11

4 -10 -16 361.21 57.2462 8

-5 10 -18 10.2359 23.3248 2

10 -5 -18 15.9369 21.5572 2

10 -5 -15 57.6414 40.6188 8

-5 -5 -15 30.8502 45.1601 8

-5 10 -15 35.6491 41.8657 14

10 -5 -12 188.944 54.7572 8

-5 -5 -12 255.193 61.9855 8

-5 10 -12 216.58 48.0695 11

5 5 12 179.947 124.716 10

-10 5 12 158.847 89.7224 10

5 -10 12 124.119 94.4029 10

-5 10 -12 383.044 59.6142 14

10 -5 -9 91.9754 41.7844 8

-5 -5 -9 178.865 53.9717 8

-5 10 -9 162.11 49.086 8

-10 5 9 84.6151 99.7492 10

5 -10 9 299.562 119.393 10

-5 -5 -9 168.402 47.7391 6

-5 10 -9 260.257 207.653 7

10 -5 -9 169.466 70.2555 7

-5 10 -6 4533.69 635.284 7

-10 5 6 4867.04 467.179 10

5 -10 6 4297.29 431.814 10

-5 10 -6 3708.44 444.023 4

5 5 316727.90 1325.23 9

-5 10 -317291.90 1410.98 7

-10 5 316297.40 1382.96 4

-10 5 315440.50 1341.42 10

5 -10 316115.00 1338.42 10

-5 10 -313222.90 1524.72 4

-5 10 0 4514.33 445.842 7

-10 5 0 4444.55 533.531 4

5 5 0 5181.06 473.11 9

-5 10 0 4638.90 554.792 4

-10 5 -328231.40 2402.57 4

-5 10 326755.90 2374.59 4

5 5 -328734.30 2378.86 9

-5 -5 331169.70 2371.42 10

-5 10 611629.10 1057.95 4

5 5 -612850.10 1068.71 9

-5 -5 613149.60 1112.39 10

5 5 -9 1116.60 202.239 7

5 5 -9 901.599 98.5533 8

-10 5 -9 666.985 108.131 8

-5 10 9 714.537 147.056 10

5 5 -9 893.557 133.018 9

5 -10 -9 1116.30 108.153 8

5 5 -12-12.9795 44.4734 8

-10 5 -12 87.9198 53.7838 8

5 -10 -12 109.301 56.6188 8

5 5 -12 52.1567 42.8521 14

-10 5 -12 183.377 57.1209 14

5 -10 -12 119.033 51.8324 6

-5 10 12 36.9598 68.9587 10

10 -5 12 61.2895 95.3817 10

-10 5 -12 122.147 43.2518 11

5 5 -12-16.4564 88.7445 7

-10 5 -15 6.75323 38.9882 2

5 5 -15 119.798 44.2036 2

5 -10 -15 71.4052 45.1666 8

-10 5 -15 14.6441 27.5639 11

-10 5 -15-45.1619 50.6955 14

5 5 -18 44.1999 24.501 2

5 -10 -18 101.899 25.725 8

-6 10 -16 96.7859 41.8635 2

10 -4 -16 117.694 39.0566 8

-4 -6 -16 137.673 40.736 8

-6 10 -16 66.7251 43.4989 14

-4 -6 -13 635.985 84.0529 8

10 -4 -13 518.12 73.5135 8

-10 4 13 417.397 116.322 10

6 -10 13 449.095 119.992 10

4 6 13 436.828 122.94 10

-6 10 -13 405.409 61.7212 11

-6 10 -13 495.462 76.5909 2

-6 10 -13 520.253 72.358 14

-4 -6 -10 1617.98 179.975 14

-6 10 -10 2448.85 190.566 14

-4 -6 -10 1869.76 184.879 8

10 -4 -10 2024.64 178.927 8

-6 10 -10 2101.57 184.63 11

-10 4 10 1863.06 247.459 10

6 -10 10 1935.80 247.495 10

10 -4 -10 1479.16 213.849 7

-4 -6 -10 1947.18 180.511 6

-4 -6 -7 89.7772 44.5616 1

6 -10 7 13.0117 101.974 10

-10 4 7 115.538 124.629 10

-6 10 -7 263.871 265.621 7

-6 10 -7 39.1123 60.334 4

4 6 7 146.007 56.2406 4

-6 10 -4 1306.69 316.623 4

-6 10 -4 1356.11 240.209 7

4 6 4 1257.30 137.336 9

-10 4 4 1382.68 195.091 10

6 -10 4 694.858 147.641 10

-6 10 -194800.70 7417.41 7

4 6 195539.60 7411.14 9

-10 4 187749.80 7475.08 4

6 -10 191606.40 7380.02 10

-10 4 191472.90 7402.28 10

-6 10 2212848.016845.20 4

-10 4 -2200273.016860.00 4

-4 -6 2213747.016801.80 10

-4 -6 527841.60 2193.87 10

-6 10 526191.60 2168.57 4

4 6 -524873.90 2174.62 9

4 6 -8 5791.80 470.437 8

4 6 -8 6020.44 584.545 7

-10 4 -8 5822.71 485.919 8

-6 10 8 5238.77 521.46 10

-4 -6 8 5678.80 564.471 10

6 -10 -8 5673.13 473.343 6

6 -10 -8 5268.75 472.198 8

4 6 -8 5819.01 525.166 9

-6 10 11 2988.91 348.532 10

10 -4 11 3336.12 380.142 10

-4 -6 11 2610.76 357.882 10

-10 4 -11 3634.56 308.808 8

4 6 -11 3538.38 298.688 8

4 6 -11 3706.70 395.061 7

-10 4 -11 3389.88 299.679 14

4 6 -11 3740.73 297.731 14

-10 4 -11 3189.18 296.428 11

6 -10 -11 3419.74 302.435 8

6 -10 -14 935.577 107.25 8

-10 4 -14 948.049 106.463 8

-10 4 -14 912.212 107.486 2

4 6 -14 1049.04 106.829 14

-10 4 -14 984.805 108.427 14

10 -4 14 867.465 163.233 10

-6 10 14 569.598 128.981 10

-10 4 -14 1100.66 101.069 11

4 6 -17 101.172 30.4726 2

-10 4 -17 81.3205 31.612 2

6 -10 -17 60.756 30.8564 8

-7 10 -17 135.559 37.3995 2

-3 -7 -17 173.644 38.0004 8

-3 -7 -14 440.286 68.9311 8

10 -3 -14 420.964 59.8986 8

-10 3 14 214.27 89.9864 10

3 7 14 254.95 101.157 10

7 -10 14 222.822 97.1112 10

-7 10 -14 318.673 59.8583 2

-7 10 -14 311.41 49.2105 11

-7 10 -14 454.285 65.10 14

-7 10 -11 8553.08 705.943 14

10 -3 -11 6942.01 742.251 7

-3 -7 -11 9405.24 711.846 8

10 -3 -11 8480.56 702.26 8

-7 10 -11 8344.41 708.878 2

-3 -7 -11 9231.34 706.203 6

-10 3 11 7905.74 771.119 10

7 -10 11 7600.15 763.689 10

-7 10 -11 8458.47 704.209 11

-7 10 -8 2347.03 430.682 7

-3 -7 -8 1836.97 180.55 1

-3 -7 -8 1944.18 179.651 6

-3 -7 -8 1911.81 182.273 8

10 -3 -8 1823.82 173.483 8

-10 3 8 2375.54 280.851 10

7 -10 8 2601.16 262.595 10

7 -10 552727.30 4426.43 10

-7 10 -547005.40 4474.81 4

3 7 230367.10 2441.21 9

-7 10 -229613.40 2464.41 7

-10 3 230234.90 2443.07 10

-10 3 232836.30 2515.80 4

-7 10 -228619.90 2642.27 4

7 -10 229353.60 2425.09 10

-7 10 171080.20 5542.34 4

3 7 -165103.90 5474.39 9

-3 -7 168534.30 5453.42 10

3 7 -4 107.967 114.378 9

-3 -7 4 214.726 94.1992 10

-7 10 4 381.639 120.17 4

3 7 -7 103.044 90.4796 9

7 -10 -7 46.3232 43.6603 8

-7 10 7 8.6202 78.8565 4

3 7 -7 -8.9468 54.8453 4

3 7 -7 19.9387 125.597 7

7 -10 -7-3.12431 45.1072 6

3 7 -7 35.7414 36.7377 8

-3 -7 7 116.279 133.246 10

-7 10 7 51.1159 79.2413 10

7 -10 -10 72.1758 46.3119 6

7 -10 -10 105.001 47.4398 8

3 7 -10 104.463 46.1773 8

-10 3 -10 32.3001 56.8978 8

3 7 -10 98.2635 43.7955 14

-10 3 -10 175.032 50.6737 14

-7 10 10 11.6986 74.1164 10

-3 -7 10 30.9191 96.3783 10

-10 3 -10 175.629 48.9133 11

3 7 -10 97.7102 97.3721 7

-10 3 -13 3145.08 262.674 11

-10 3 -13 3103.18 270.454 8

3 7 -13 2432.45 332.105 7

-3 -7 13 2115.40 314.481 10

-7 10 13 2477.28 309.999 10

10 -3 13 3252.78 354.388 10

3 7 -13 3277.33 266.731 14

-10 3 -13 3079.28 267.716 14

7 -10 -13 2986.16 268.216 8

7 -10 -16 25.4386 36.6653 8

3 7 -16 12.8764 34.8436 2

-10 3 -16 47.111 34.0296 2

7 -10 -19 142.131 21.9595 2

-8 10 -18 46.6166 24.5539 2

-2 -8 -18 17.5238 21.7621 8

10 -2 -18 4.27265 21.982 2

-2 -8 -15 224.282 51.8875 8

10 -2 -15 217.51 47.1852 8

-8 10 -15 167.642 35.1013 11

-8 10 -15 137.844 43.7402 2

-8 10 -15 218.747 52.0031 14

-8 10 -15 156.865 42.3538 3

-10 2 12 616.685 149.11 10

8 -10 12 827.537 165.35 10

2 8 12 1130.15 180.436 10

-2 -8 -12 1299.14 123.187 8

10 -2 -12 937.477 107.424 8

-8 10 -12 929.367 114.104 2

-8 10 -12 1151.40 115.078 14

-8 10 -12 892.392 104.62 11

-2 -8 -12 1046.06 110.794 6

-2 -8 -9 383.714 68.684 8

10 -2 -9 463.648 60.1438 8

-2 -8 -9 424.226 67.4343 6

-8 10 -9 383.488 70.4166 8

8 -10 9 675.614 128.582 10

-10 2 9 303.001 127.068 10

-8 10 -9 430.21 74.9589 2

10 -2 -9 414.233 110.858 7

-8 10 -640121.60 3219.69 7

2 8 637708.80 3051.39 4

-10 2 640707.10 3132.74 10

8 -10 638722.10 3083.83 10

-8 10 -633567.20 3084.31 4

-8 10 -3124606.010716.80 4

-8 10 -3126051.010580.00 7

-10 2 3135060.010559.60 10

8 -10 3127142.010545.50 10

2 8 3138652.010543.90 9

-2 -8 024845.20 2099.06 10

2 8 025544.90 2112.22 9

-8 10 028789.70 2251.41 4

-10 2 026465.40 2193.38 4

-8 10 3 717.517 123.164 10

-2 -8 3 914.215 145.846 10

-8 10 3 906.054 182.789 4

2 8 -3 899.22 185.654 9

8 -10 -6 284.457 50.2767 7

2 8 -6 797.106 203.36 7

-8 10 6 521.419 116.633 10

-2 -8 6 618.267 167.433 10

2 8 -6 561.772 131.727 4

-8 10 6 442.168 117.623 4

2 8 -6 556.761 152.667 9

8 -10 -9 9579.14 760.117 8

2 8 -9 9120.41 756.204 8

2 8 -910044.00 893.295 7

-10 2 -9 8657.63 765.888 8

8 -10 -9 9709.16 759.528 6

-2 -8 9 9209.65 846.745 10

-8 10 9 8147.41 803.953 10

2 8 -9 8971.07 816.112 9

-10 2 -12 1887.21 189.146 8

-10 2 -12 2095.17 182.618 11

-2 -8 12 1744.31 240.463 10

10 -2 12 2065.80 269.182 10

-8 10 12 1486.26 225.635 10

2 8 -12 1652.53 259.384 7

8 -10 -12 1939.12 185.145 8

2 8 -12 2210.70 182.30 14

-10 2 -12 1827.09 181.963 14

2 8 -15 372.886 60.5894 14

8 -10 -15 401.375 62.9263 8

2 8 -15 357.799 60.1224 2

-10 2 -15 425.831 63.0938 2

8 -10 -18 15.3133 20.8884 8

2 8 -18-15.5832 22.647 2

-1 -9 -16 49.1668 37.5978 8

-9 10 -16 88.0647 37.8746 2

-9 10 -13 643.469 91.2715 2

10 -1 -13 690.349 86.0447 8

9 -10 13 590.107 139.201 10

1 9 13 835.588 148.545 10

-10 1 13 535.715 130.87 10

-9 10 -13 824.82 85.5321 11

-1 -9 -13 849.822 97.7194 8

9 -10 10 250.587 100.945 10

-10 1 10 576.052 144.484 10

10 -1 -10 518.98 99.383 9

10 -1 -10 415.56 104.836 7

-9 10 -10 356.114 72.3562 2

-9 10 -10 373.369 65.7805 11

-1 -9 -10 321.71 68.4694 8

10 -1 -10 381.653 59.1987 8

-1 -9 -10 340.296 62.6638 6

-1 -9 -713362.50 1000.75 8

-9 10 -711554.50 1001.88 2

-1 -9 -712602.90 995.922 6

-9 10 -710821.40 1036.30 4

9 -10 714058.30 1073.99 10

-10 1 714363.90 1123.15 10

1 9 713098.80 1031.28 4

-1 -9 -711137.00 993.261 1

-9 10 -4 3889.43 493.112 4

9 -10 4 3839.64 391.783 10

-10 1 4 4382.42 419.87 10

1 9 1 5246.99 465.978 9

-10 1 1 5647.18 469.719 10

9 -10 1 4902.92 418.666 10

-9 10 262521.70 4375.10 4

-1 -9 265218.40 4293.54 10

-9 10 549650.80 3678.74 10

-1 -9 549956.70 3698.31 10

-9 10 546407.40 3681.55 4

1 9 -541489.90 3730.13 9

10 -1 543942.10 3608.15 9

9 -10 -8 4347.66 377.432 6

1 9 -8 5776.29 549.883 7

1 9 -8 4497.20 375.888 8

-10 1 -8 4150.75 386.78 8

-9 10 8 4667.80 433.281 10

-1 -9 8 4788.66 473.337 10

9 -10 -8 4469.29 377.874 8

1 9 -8 4098.62 457.634 9

-10 1 -11 7167.41 535.756 11

-10 1 -11 6098.66 533.025 14

1 9 -11 7404.32 533.891 14

-1 -9 11 5018.63 593.516 10

-9 10 11 5549.89 581.492 10

-10 1 -11 6083.33 539.91 8

1 9 -11 6843.61 660.541 7

9 -10 -11 5740.98 533.434 8

1 9 -14 158.028 46.8115 14

-10 1 -14-3.50308 57.356 14

1 9 -14 185.847 49.8433 2

-10 1 -14 156.194 40.1565 11

-1 -9 14 26.5489 73.2751 10

-9 10 14 32.8042 71.2671 10

10 -1 14 105.356 89.9671 10

-10 1 -14 101.129 53.075 8

9 -10 -14 128.563 47.2744 8

9 -10 -17 147.867 31.942 8

1 9 -17 75.2952 32.1151 2

0 -10 -17 388.335 53.0853 8

-10 10 -17 367.635 53.7281 2

-10 10 -14 215.099 52.0198 2

10 -10 14 140.639 78.1905 10

-10 0 14 64.1462 72.6112 10

0 10 14 117.918 77.2593 10

-10 10 -14 187.479 54.1737 3

-10 10 -14 170.543 39.564 11

0 -10 -14 190.242 57.3007 8

10 0 -14 111.104 42.6263 8

-10 10 -11 166.846 53.9458 11

0 -10 -11 305.184 65.4454 8

10 0 -11 313.486 53.5479 8

10 -10 11 80.6623 96.7219 10

0 10 11 306.921 113.85 10

-10 0 11 260.759 116.442 10

10 0 -11 107.409 84.8882 7

0 -10 -11 391.298 62.3073 6

-10 10 -11 317.729 68.5149 2

0 -10 -820650.60 1611.69 1

-10 10 -821152.30 1622.90 2

0 -10 -818996.70 1609.78 6

0 -10 -819842.70 1614.18 8

10 0 -819687.40 1605.52 8

-10 0 818898.50 1712.31 10

10 -10 820059.30 1674.67 10

-10 10 -516868.40 1587.70 4

0 10 518459.20 1537.42 4

10 -10 518140.10 1548.59 10

-10 0 520802.10 1601.03 10

0 10 288205.70 7660.17 7

10 -10 2101165.0 7678.80 10

0 10 2102402.0 7697.85 9

-10 0 2101923.0 7700.57 10

-10 10 -288998.30 7840.79 4

-10 10 1 6709.23 723.128 4

0 -10 1 7097.90 623.221 10

0 10 -1 8015.75 720.602 7

-10 10 437616.40 3095.83 4

0 -10 443403.00 3086.05 10

-10 10 438285.60 3077.23 10

0 10 -440590.30 3218.44 7

0 10 -440293.10 3159.56 9

10 0 435085.30 3023.68 9

10 -10 -7 681.856 101.63 7

10 -10 -7 1188.16 105.85 6

-10 10 7 939.314 158.644 10

0 -10 7 765.049 177.923 10

0 10 -7 492.868 176.382 9

10 -10 -7 888.289 99.8419 8

0 10 -7 996.571 155.117 4

-10 0 -7 1079.42 103.791 1

0 10 -7 1209.29 281.41 7

-10 10 7 1018.95 149.581 4

10 -10 -10 5868.64 493.806 8

0 10 -10 6352.37 645.804 7

-10 0 -10 5956.56 502.277 8

-10 0 -10 6556.07 495.819 11

10 -10 -10 5538.12 491.785 6

-10 0 -10 5193.51 489.616 14

0 10 -10 6658.33 493.772 14

-10 10 10 4939.95 538.481 10

0 -10 10 5520.83 569.504 10

10 0 13 495.598 131.988 10

-10 10 13 337.217 100.866 10

0 -10 13 416.44 115.743 10

-10 0 -13 580.834 70.7844 11

0 10 -13 543.308 145.563 7

10 -10 -13 668.324 79.1918 8

0 10 -13 470.012 69.2075 14

-10 0 -13 319.304 73.9075 14

-10 0 -13 378.974 76.9043 8

0 10 -13 769.663 77.9508 2

10 -10 -16 43.3967 32.3324 8

0 10 -16 10.2673 33.7928 2

11 -10 -18 47.0159 20.4641 8

-1 11 -18 76.344 25.426 2

-10 -1 -15 35.891 39.9825 8

11 -10 -15 11.2055 36.9279 8

-1 11 -15 26.5469 39.0012 2

-10 -1 -15 9.72981 38.0021 2

-1 11 -15-23.1215 38.0021 14

-11 10 12 1220.75 187.908 10

11 -10 -12 1347.25 146.60 8

-10 -1 -12 1433.69 154.623 8

-1 11 -12 1648.56 144.515 14

-10 -1 -12 1312.38 147.282 14

-1 11 -12 1290.71 238.558 7

-1 11 -12 1449.76 146.697 2

10 1 12 1405.99 232.855 10

1 -11 12 1172.28 201.327 10

-10 -1 -12 1877.18 148.087 11

11 -10 -9 220.555 50.3804 6

-10 -1 -9 78.5957 36.3481 6

11 -10 -9 219.839 51.3205 8

-10 -1 -9 255.39 51.3776 11

-10 -1 -9 156.347 55.1758 8

-11 10 9 217.932 89.5238 10

1 -11 9 160.816 112.399 10

-1 11 -9 201.172 152.247 7

-1 11 -9 94.3928 45.9233 2

-11 10 6 4759.12 445.836 4

-1 11 -6 4772.51 382.332 2

1 -11 6 4585.60 468.884 10

-11 10 6 4829.55 463.981 10

-1 11 -6 4120.80 485.386 4

1 -11 350270.80 3986.17 10

-11 10 344175.00 3953.93 10

-11 10 353594.50 4044.57 4

10 1 349958.40 3963.11 9

-1 11 -356735.00 4068.31 9

-1 11 -359507.00 4165.41 7

-1 11 0 8834.06 780.318 7

-1 11 0 8518.39 774.196 9

1 -11 0 8924.36 751.691 10

-11 10 0 9057.12 893.19 4

-11 10 -314627.60 1183.79 4

-10 -1 316331.20 1058.96 10

1 -11 -630361.60 2459.43 6

11 -10 631503.80 2525.78 10

-10 -1 636510.70 2599.42 10

-11 10 -627011.10 2529.52 4

-1 11 631506.10 2512.85 4

10 1 -9 2115.50 226.576 9

-10 -1 9 1346.74 249.158 10

-11 10 -9 2126.60 182.725 14

1 -11 -9 1588.52 172.134 8

-11 10 -9 2060.01 176.218 11

1 -11 -9 1499.96 166.866 6

11 -10 9 1648.49 229.168 10

-11 10 -9 1947.36 186.989 2

10 1 -9 1885.17 241.473 7

10 1 -9 1669.25 163.668 8

1 -11 -12 77.6506 46.2502 6

10 1 -12 18.1206 40.1172 8

1 -11 -12 45.1496 53.7335 8

-11 10 -12 99.985 50.4435 14

10 1 -12 26.3439 61.1201 7

-11 10 -12 47.9993 39.8842 11

11 -10 12 81.4554 85.5951 10

-1 11 12 39.0731 83.2249 10

-10 -1 12 89.6115 92.8697 10

-11 10 -12 28.9301 50.3276 2

1 -11 -15 878.519 93.3719 8

-11 10 -15 824.262 90.9285 2

-11 10 -15 714.679 79.6054 11

10 1 -18 91.3548 23.5219 2

1 -11 -18 98.121 25.2524 8

-2 11 -16 143.292 41.5141 2

-9 -2 -16 155.563 40.2329 8

11 -9 -16 201.695 40.3183 8

-2 11 -16 56.7047 39.9393 14

-2 11 -13 3413.32 309.444 2

-9 -2 -13 3467.15 312.96 8

-2 11 -13 3966.79 307.131 14

-9 -2 -13 3215.13 309.937 14

11 -9 -13 3704.57 309.687 8

-11 9 13 2583.61 345.733 10

2 -11 13 2672.42 356.013 10

-9 -2 -13 4079.27 307.114 11

-2 11 -10 7355.14 599.295 2

11 -9 -10 7494.32 598.413 8

-9 -2 -10 7625.49 604.657 8

-2 11 -10 8827.04 795.975 7

-9 -2 -10 6094.60 593.229 14

-2 11 -10 7602.31 598.529 14

-9 -2 -10 6351.30 589.993 6

-9 -2 -10 8439.12 600.589 11

-2 11 -10 7262.47 594.979 11

-11 9 10 6890.78 655.891 10

2 -11 10 7174.72 675.591 10

-2 11 -7 2646.11 306.181 4

-2 11 -7 1799.97 350.476 9

11 -9 -7 2526.93 230.719 8

-11 9 7 3032.17 287.757 4

2 -11 7 2965.62 330.194 10

-11 9 7 3165.38 318.684 10

-9 -2 -7 2573.16 232.548 1

11 -9 -7 2515.54 231.151 6

-2 11 -7 2719.61 234.946 2

-2 11 -431335.70 2424.40 7

-2 11 -423159.80 2344.84 4

9 2 424763.40 2165.89 9

-2 11 -429071.10 2301.14 9

-11 9 427962.40 2237.05 4

2 -11 429306.30 2216.03 10

-11 9 428704.80 2219.28 10

-11 9 140841.30 3127.94 4

-2 11 -139316.30 3073.39 7

9 2 136988.80 3029.19 9

2 -11 135831.70 3009.90 10

-11 9 -238575.30 3672.91 4

-2 11 243936.50 3528.01 7

-2 11 247685.40 3608.46 4

-2 11 5 2162.23 244.746 4

-9 -2 5 2161.08 284.244 10

-11 9 -5 1940.47 274.299 4

2 -11 -8 6455.68 516.758 8

11 -9 8 5212.97 563.73 10

-9 -2 8 5583.08 613.201 10

9 2 -8 6872.02 597.081 7

-11 9 -8 5481.12 518.771 8

9 2 -8 6507.13 509.079 8

2 -11 -8 6191.36 513.433 6

-11 9 -8 5769.32 523.056 2

9 2 -8 6810.38 569.347 9

-2 11 11 26.1558 85.647 10

-9 -2 11 31.4899 89.2984 10

11 -9 11-27.4906 90.0173 10

-11 9 -11 15.7824 55.2902 2

2 -11 -11 29.0718 51.4314 8

9 2 -11 18.3545 39.4448 8

-11 9 -11 38.6733 51.132 14

2 -11 -11 25.7129 45.9656 6

9 2 -11 14.6881 74.5115 7

-11 9 -11-32.7346 43.1137 11

-11 9 -14 937.613 98.5616 2

-11 9 -14 785.314 88.2088 11

-11 9 -14 767.768 92.266 14

9 2 -14 882.477 90.528 8

-9 -2 14 635.302 132.325 10

11 -9 14 868.314 147.718 10

-2 11 14 657.44 127.623 10

-11 9 -17 921.664 90.3375 2

9 2 -17 947.642 89.1002 2

2 -11 -17 857.042 89.0827 8

11 -8 -17 40.2325 26.2965 8

-8 -3 -14 60.2675 49.8099 8

11 -8 -14 12.6354 41.7775 8

-3 11 -14-14.1808 42.5745 14

-11 8 14-11.4042 60.2239 10

3 -11 14 12.9193 80.272 10

-3 11 -14 33.557 44.1055 2

-11 8 11 89.2952 81.7815 10

3 -11 11 145.079 106.497 10

-3 11 -11 152.787 50.7424 2

-8 -3 -11 143.558 43.1163 11

-8 -3 -11 73.9118 47.5819 8

11 -8 -11 102.41 47.1176 8

-3 11 -11-52.1088 150.40 7

-3 11 -11 93.3376 41.2117 11

-3 11 -11 204.25 53.5056 14

-8 -3 -11 33.3938 45.1279 14

-8 -3 -11 44.6977 38.8033 6

-8 -3 -8 9889.65 817.834 6

11 -8 -810270.90 820.666 6

11 -8 -8 9055.84 846.67 7

-3 11 -812773.20 1116.80 7

-11 8 810605.20 899.602 10

3 -11 8 9577.44 899.902 10

-3 11 -810934.10 826.284 2

11 -8 -8 9831.83 819.571 8

-8 -3 -8 9606.06 825.813 8

-3 11 -513896.30 1495.69 4

8 3 516437.70 1362.17 9

-3 11 -516312.40 1516.43 9

-3 11 -519771.60 1680.85 7

-11 8 518009.50 1448.96 10

3 -11 518309.50 1427.17 10

-11 8 516561.50 1416.55 4

3 -11 215517.30 1218.79 10

-3 11 -215600.70 1266.80 9

8 3 215633.00 1221.91 9

-3 11 -212952.90 1415.09 4

-11 8 216395.20 1306.34 4

-11 8 213036.80 1196.95 10

-3 11 -217322.10 1310.85 7

-3 11 1 2089.25 201.349 7

-3 11 1 1959.13 286.246 4

-11 8 -4 195.726 126.826 4

-3 11 4 328.73 109.11 4

-11 8 -714746.50 1235.59 8

8 3 -716716.90 1287.36 9

-3 11 715754.00 1278.64 4

3 -11 -714859.90 1227.58 1

-3 11 10 458.727 117.09 10

-8 -3 10 394.168 130.923 10

11 -8 10 495.189 127.799 10

8 3 -10 404.607 91.7303 9

-11 8 -10 408.55 71.1624 11

-11 8 -10 503.086 86.1401 8

8 3 -10 608.159 72.3838 8

8 3 -10 344.207 114.333 7

-11 8 -10 476.778 79.6379 14

8 3 -10 496.448 69.3287 14

3 -11 -10 597.342 77.2685 6

-11 8 -13 321.088 55.8789 11

-11 8 -13 274.644 72.9569 3

8 3 -13 337.694 59.3022 8

11 -8 13 278.443 104.272 10

-3 11 13 322.861 100.97 10

-8 -3 13 267.901 102.445 10

-11 8 -13 359.458 66.0105 2

-11 8 -13 398.039 67.7837 14

-11 8 -16 899.62 91.9015 2

8 3 -16 991.19 90.0248 2

-11 8 -16 782.809 79.3775 11

-11 8 -16 758.281 90.2573 14

-7 -4 -15 322.177 60.0228 8

11 -7 -15 388.35 58.1126 8

-4 11 -15 313.509 57.8955 14

-4 11 -12 246.955 59.1862 2

11 -7 -12 370.775 59.8557 8

-7 -4 -12 286.155 63.805 8

-4 11 -12 322.286 59.001 14

-7 -4 -12 70.3269 56.7247 14

-4 11 -12 289.966 50.9142 11

-11 7 12 218.544 92.0551 10

4 -11 12 434.29 132.456 10

-7 -4 -12 429.485 57.901 11

-4 11 -911726.60 933.785 2

4 -11 911915.20 1010.37 10

-11 7 910920.30 999.389 10

-7 -4 -911196.10 927.18 6

-7 -4 -911427.90 933.069 8

11 -7 -911426.40 927.808 8

11 -7 -910512.70 960.401 7

-4 11 -913552.10 1194.93 7

-4 11 -6 6012.52 629.198 4

-11 7 6 7204.58 585.967 4

-11 7 6 7049.63 627.521 10

4 -11 6 6627.98 600.083 10

-4 11 -391847.10 7401.80 9

7 4 391779.70 7335.25 9

-4 11 -373802.40 7557.43 4

-11 7 399926.90 7407.96 4

-4 11 -3106080.0 7470.62 7

4 -11 391299.10 7350.35 10

-11 7 387421.40 7345.42 10

-4 11 052514.00 3859.78 4

7 4 044740.90 3755.83 9

-7 -4 046987.30 3755.14 10

-11 7 044207.70 3832.24 4

-4 11 046018.60 3737.98 7

7 4 -3 589.72 126.017 9

-11 7 -3 687.218 198.643 4

-4 11 3 339.814 123.08 4

7 4 -622903.00 1885.56 9

-4 11 624048.70 1888.47 4

-11 7 -621106.40 1889.72 4

11 -7 9 178.882 109.989 10

-4 11 9 243.115 99.0154 10

7 4 -9 412.651 91.8506 9

7 4 -9 414.373 59.4386 8

-11 7 -9 400.553 80.6236 8

-4 11 12 838.699 151.017 10

11 -7 12 873.975 165.695 10

-7 -4 12 579.627 149.738 10

-11 7 -12 1090.29 120.334 2

7 4 -12 1125.03 176.221 7

-11 7 -12 932.512 104.63 11

7 4 -12 813.771 103.299 8

-11 7 -12 985.17 113.778 8

4 -11 -12 1216.75 113.154 6

-11 7 -12 1034.81 108.914 14

7 4 -12 934.391 107.072 14

-11 7 -15-13.4223 36.4602 2

7 4 -15 5.61159 38.6698 2

-11 7 -15-51.4253 51.0804 14

-11 7 -15-10.6338 27.8809 11

7 4 -18 101.115 26.0395 2

-5 11 -16 1427.07 140.771 14

-5 11 -16 1439.66 139.433 3

-6 -5 -16 1565.41 143.72 8

11 -6 -16 1625.42 140.112 8

-11 6 13 3600.78 446.368 10

6 5 13 4803.26 486.289 10

5 -11 13 3699.59 451.621 10

-6 -5 -13 4990.05 408.283 8

11 -6 -13 5103.27 403.442 8

-5 11 -13 4540.81 394.977 11

-5 11 -13 5143.33 401.916 14

-5 11 -10 5388.55 401.47 14

-6 -5 -10 3336.39 390.166 14

11 -6 -10 4733.32 393.54 8

-6 -5 -10 4622.22 398.321 8

-6 -5 -10 5357.29 395.069 11

-5 11 -10 4951.99 397.181 11

-11 6 10 4681.87 464.293 10

5 -11 10 4600.08 464.971 10

-6 -5 -10 4555.34 393.492 6

-5 11 -10 4951.07 585.859 7

-6 -5 -7 185.411 52.2499 1

5 -11 7 415.579 128.034 10

-11 6 7 524.772 147.247 10

-5 11 -7 305.765 299.071 7

-5 11 -7 195.085 89.2302 4

-5 11 -483930.90 6106.32 7

6 5 473676.90 5929.27 9

-5 11 -466379.10 6008.88 9

-5 11 -467468.70 6133.29 4

5 -11 474232.60 5958.54 10

-11 6 479277.70 5981.07 10

-11 6 473287.20 5980.99 4

6 5 149918.30 3832.56 9

-5 11 -148466.00 3838.52 7

5 -11 147582.40 3801.04 10

-11 6 144519.60 3810.18 10

-11 6 149152.60 3908.51 4

-6 -5 2133912.010108.50 10

-11 6 -2112525.010164.90 4

-5 11 2125495.010141.00 4

-11 6 -516450.20 1551.17 4

-6 -5 522544.20 1593.54 10

-5 11 517447.30 1543.21 4

6 5 -518534.30 1547.31 9

-11 6 -8 4145.97 366.026 8

6 5 -8 4536.09 445.39 7

6 5 -8 4163.60 348.622 8

5 -11 -8 4206.89 353.705 6

-5 11 8 3966.30 404.193 10

5 -11 -8 3913.32 352.968 8

6 5 -8 3933.70 394.954 9

6 5 -11 3631.65 418.263 7

-11 6 -11 3636.87 344.819 8

6 5 -11 3902.19 336.717 8

11 -6 11 3557.42 409.901 10

-5 11 11 3878.81 395.166 10

-11 6 -11 4211.80 341.941 14

6 5 -11 3942.89 335.623 14

-11 6 -11 4224.80 339.639 11

5 -11 -11 3711.19 341.657 8

-6 -5 14 924.617 160.296 10

11 -6 14 775.405 160.82 10

-5 11 14 749.096 142.754 10

-11 6 -14 1028.45 116.929 2

-11 6 -14 1239.70 110.187 11

-11 6 -14 1124.47 115.963 14

5 -11 -14 1198.01 119.60 8

6 5 -17 84.3871 29.3702 2

5 -11 -17 59.3777 29.0386 8

-6 11 -17 224.217 39.1308 2

-5 -6 -17 149.618 35.2101 8

11 -5 -17 142.531 31.2481 8

-6 11 -14 181.784 53.1401 14

-6 11 -14 206.685 39.654 11

-11 5 14 158.445 74.2783 10

5 6 14 269.975 102.154 10

6 -11 14 167.557 84.8991 10

-5 -6 -14 198.953 57.5616 8

11 -5 -14 233.577 49.0772 8

-6 11 -11 32.2005 47.0268 14

-5 -6 -11 23.9102 45.5971 8

11 -5 -11 22.1471 38.9251 8

-5 -6 -11 3.33332 41.0071 6

6 -11 11 41.2697 96.6173 10

-11 5 11 41.3512 78.1839 10

-6 11 -11 3.37972 37.5535 11

-5 -6 -8 3408.29 328.953 8

11 -5 -8 3878.81 324.801 8

-5 -6 -8 4434.39 332.979 1

6 -11 8 3385.93 391.748 10

-11 5 8 3648.99 412.81 10

-6 11 -8 4115.02 622.451 7

-5 -6 -8 3761.41 327.066 6

6 -11 5 4247.45 458.815 10

-11 5 5 5080.74 505.976 10

-11 5 5 5615.95 483.085 4

-6 11 -5 4410.38 555.217 4

-6 11 -2 214.313 344.369 4

-11 5 2 182.433 156.811 4

6 -11 2 259.212 61.7957 10

-11 5 2 425.664 79.9028 10

5 6 2 146.945 68.3056 9

-6 11 -2 231.034 105.54 7

-5 -6 1 3351.50 325.554 10

-6 11 1 3793.64 407.988 4

5 6 -1 2931.53 338.719 9

-6 11 4 577.725 134.471 4

5 6 -4 452.628 128.248 9

-11 5 -4 700.295 162.883 4

-5 -6 4 479.526 129.773 10

6 -11 -7 9263.13 797.662 8

-6 11 710930.10 855.066 4

-5 -6 710175.00 908.527 10

-6 11 7 9927.79 849.884 10

6 -11 -7 9297.04 798.456 6

5 6 -710274.30 859.181 9

5 6 -712143.10 922.052 7

-11 5 -10 7862.86 657.549 11

6 -11 -10 7850.72 659.512 8

-11 5 -10 8120.53 659.88 14

5 6 -10 8649.75 654.301 14

5 6 -10 7910.82 655.074 8

5 6 -10 8110.38 758.48 7

-11 5 -10 7389.08 665.778 8

-6 11 10 7247.31 707.346 10

11 -5 10 6916.52 723.47 10

5 6 -13 3929.23 469.771 7

-11 5 -13 4341.97 405.85 8

-11 5 -13 5520.35 415.055 2

-11 5 -13 4376.46 401.853 14

5 6 -13 5249.83 405.409 14

-6 11 13 3922.42 447.91 10

11 -5 13 4302.11 472.814 10

6 -11 -13 4996.77 407.407 8

-11 5 -13 4919.35 401.974 11

6 -11 -16 1492.06 144.131 8

5 6 -16 1673.05 145.335 2

-11 5 -16 1547.42 146.362 2

11 -4 -18-20.5661 21.2568 2

-7 11 -18-18.2048 24.5845 2

-4 -7 -15 431.726 66.1933 8

11 -4 -15 379.599 59.1438 8

-7 11 -15 508.389 62.9331 3

-7 11 -15 395.824 63.7348 14

-7 11 -15 339.367 48.6138 11

-7 11 -15 563.835 66.9675 2

-7 11 -12 4175.92 349.62 2

-4 -7 -12 4678.24 352.551 8

11 -4 -12 4059.14 342.795 8

-7 11 -12 3515.28 341.378 14

-4 -7 -12 4377.28 344.688 6

7 -11 12 3314.20 400.464 10

-11 4 12 3069.78 393.578 10

4 7 12 4101.77 430.419 10

-7 11 -12 3892.02 340.933 11

-7 11 -9 60.2499 46.3282 2

-4 -7 -9 15.1945 41.8324 8

11 -4 -9 14.081 33.782 8

-7 11 -9 83.5791 51.1577 14

7 -11 9-26.9831 84.9842 10

-11 4 9-15.6081 98.3137 10

11 -4 -9-32.8978 61.1602 7

-4 -7 -9 22.8543 40.73 6

-7 11 -617309.90 1585.07 4

7 -11 618347.90 1558.08 10

-11 4 620043.50 1608.73 10

-7 11 -3 2470.21 315.707 7

7 -11 3 2555.95 269.578 10

-11 4 3 3000.85 290.069 10

4 7 3 2680.56 268.858 9

-11 4 3 3937.84 364.843 4

-7 11 -3 2920.64 514.56 4

-4 -7 016574.50 1322.04 10

4 7 015378.70 1326.97 9

-7 11 016111.40 1443.53 4

-11 4 014799.90 1396.36 4

-7 11 322501.60 1946.52 4

-4 -7 324023.10 1921.06 10

-11 4 -323570.90 1971.99 4

4 7 -322407.90 1943.95 9

-7 11 6 6644.59 625.787 4

4 7 -6 7573.59 680.917 7

4 7 -6 7074.33 637.999 9

-7 11 6 6261.19 604.606 10

-4 -7 6 7234.28 666.227 10

4 7 -9 754.468 129.16 9

7 -11 -9 786.037 92.5348 8

-7 11 9 528.999 124.225 10

-4 -7 9 904.89 175.716 10

4 7 -9 887.608 193.417 7

7 -11 -9 668.61 90.145 6

4 7 -9 821.663 88.1494 8

-11 4 -9 697.95 103.363 8

-11 4 -12 6994.67 559.789 11

4 7 -12 7702.65 561.495 14

-11 4 -12 6618.49 560.684 14

11 -4 12 5851.98 633.132 10

-7 11 12 6294.31 612.252 10

-4 -7 12 4919.72 611.679 10

7 -11 -12 6936.80 564.023 8

4 7 -12 5941.07 641.463 7

4 7 -12 7068.54 559.237 8

-11 4 -12 5980.10 564.728 8

-11 4 -15 328.174 45.3935 11

-11 4 -15 132.295 55.1497 14

7 -11 -15 282.71 54.0683 8

4 7 -15 351.685 54.911 2

-11 4 -15 296.494 56.3602 2

7 -11 -18 19.3115 20.111 8

4 7 -18 35.5221 21.1782 2

-8 11 -16 891.021 99.8831 14

-3 -8 -16 981.104 102.188 8

11 -3 -16 1041.14 97.9874 8

-8 11 -16 1001.07 100.591 2

11 -3 -13 7024.27 523.987 8

-8 11 -13 5767.61 518.007 11

3 8 13 5622.28 588.975 10

-11 3 13 5098.95 575.842 10

8 -11 13 5144.17 576.23 10

-8 11 -13 6336.53 522.729 14

-8 11 -13 6111.83 526.646 2

-3 -8 -13 7343.89 532.083 8

11 -3 -10 374.487 94.5703 7

-8 11 -10 516.838 76.8331 14

-8 11 -10 474.321 68.8622 11

-11 3 10 304.398 118.213 10

8 -11 10 254.893 112.661 10

11 -3 -10 644.675 111.457 9

-8 11 -10 530.897 80.5973 2

-3 -8 -10 477.382 73.7021 8

-3 -8 -10 428.752 69.7518 6

11 -3 -10 425.845 63.0212 8

8 -11 7 617.639 153.731 10

-11 3 7 466.757 182.364 10

-3 -8 -7 975.766 93.3097 6

-3 -8 -7 803.164 96.0014 8

-8 11 -7 609.342 126.534 4

-8 11 -7 374.34 286.369 7

3 8 7 845.386 117.409 4

-8 11 -7 468.197 91.6613 2

-3 -8 -7 1254.43 98.8105 1

8 -11 4 2146.46 251.197 10

-11 3 4 2624.55 282.057 10

-8 11 -4 2868.15 330.207 7

3 8 4 2248.29 234.882 9

-8 11 -4 1979.84 388.355 4

8 -11 1 1636.69 150.086 10

-11 3 1 1897.66 196.835 10

3 8 1 1541.96 192.565 9

-11 3 1 1978.98 292.936 4

-3 -8 248431.80 3888.27 10

-8 11 245868.60 3855.72 10

-11 3 -255278.70 3989.39 4

-8 11 251252.90 3961.68 4

3 8 -5 6030.72 601.381 9

11 -3 5 6034.51 502.265 9

-8 11 5 5926.92 582.171 4

-8 11 5 6704.46 559.341 10

-3 -8 5 6648.89 599.204 10

3 8 -8 104.032 86.5413 9

3 8 -8 41.2743 126.638 7

-3 -8 8 85.7676 129.521 10

-8 11 8 85.0851 80.5203 10

8 -11 -8 110.602 43.5733 8

-11 3 -8 107.541 59.1333 8

3 8 -8 80.2477 39.4003 8

8 -11 -8 94.7976 47.1054 6

3 8 -11 2464.54 222.358 8

-11 3 -11 2157.64 231.316 8

3 8 -11 2549.32 329.702 7

-3 -8 11 2129.20 290.358 10

11 -3 11 2487.12 307.551 10

-8 11 11 2182.22 273.925 10

8 -11 -11 2633.86 227.805 6

8 -11 -11 2380.38 226.762 8

-11 3 -11 2699.56 225.826 11

3 8 -11 2704.95 222.874 14

-11 3 -11 2423.89 225.527 14

-11 3 -14 4050.85 326.505 2

3 8 -14 4171.22 320.889 2

-11 3 -14 3250.81 317.263 14

3 8 -14 4031.09 318.954 14

8 -11 -14 3826.43 320.458 8

-11 3 -14 3457.81 321.316 8

-11 3 -14 3947.21 315.042 11

11 -3 14 3253.71 382.847 10

-3 -8 14 2702.97 364.429 10

-8 11 14 2815.88 352.38 10

8 -11 -17 70.9702 28.8557 8

3 8 -17 32.2113 27.2359 2

-9 11 -17 43.5109 30.0055 2

-2 -9 -17 14.2973 26.6241 8

11 -2 -17 48.2425 28.7279 2

-9 11 -17-44.4691 39.4107 14

-9 11 -14 176.594 38.039 11

-2 -9 -14 174.003 55.4528 8

11 -2 -14 207.867 44.3554 8

9 -11 14 12.7644 71.2126 10

2 9 14 125.349 81.1258 10

-11 2 14 37.7485 66.5117 10

-9 11 -14 140.579 50.2038 2

-9 11 -14 147.313 51.3381 3

-9 11 -11 2800.43 256.991 2

-2 -9 -11 3485.48 260.517 8

11 -2 -11 2734.66 245.288 8

11 -2 -11 2220.63 290.237 7

-9 11 -11 2748.08 248.266 11

-9 11 -11 2744.80 252.282 14

9 -11 11 2162.94 296.578 10

-11 2 11 2553.35 321.613 10

-2 -9 -11 2919.34 250.852 6

-2 -9 -817375.70 1428.36 6

-9 11 -817794.00 1438.94 2

-2 -9 -817840.70 1429.38 1

9 -11 818446.90 1499.18 10

-11 2 818253.70 1539.01 10

11 -2 -817136.10 1424.11 8

-2 -9 -818110.20 1433.21 8

9 -11 524375.10 2030.67 10

-9 11 -519908.50 2076.71 4

9 -11 220663.30 1759.72 10

-11 2 223538.20 1796.47 10

-9 11 -219699.10 1988.77 4

2 9 222672.00 1788.43 9

-2 -9 1 5643.97 504.568 10

2 9 -1 5699.05 536.706 9

-9 11 1 5396.88 609.813 4

2 9 -4 2886.73 353.599 7

-2 -9 4 2957.76 285.652 10

-9 11 4 2707.26 261.402 10

2 9 -4 2623.78 329.467 9

-9 11 4 1713.05 274.252 4

11 -2 4 2041.84 211.441 9

-2 -9 7 150.536 122.136 10

-9 11 7 125.315 80.8311 10

2 9 -7 98.2036 113.389 9

9 -11 -7 204.13 49.0854 8

2 9 -7 230.409 82.2519 4

-9 11 7 290.182 98.3176 4

9 -11 -7 204.117 52.2072 6

-11 2 -7 173.196 51.7182 1

2 9 -7 45.6051 136.44 7

9 -11 -10 73.7071 46.8988 8

-11 2 -10 65.4318 58.1822 8

2 9 -10 177.554 45.761 14

-11 2 -10 111.249 45.594 14

-11 2 -10 61.6018 44.4193 11

-2 -9 10 175.907 106.233 10

-9 11 10 45.6562 74.1068 10

9 -11 -10 28.8124 45.0896 6

2 9 -10-21.4599 111.301 7

-11 2 -13 169.913 48.4013 11

2 9 -13 163.459 47.1636 14

-11 2 -13 77.3091 51.4488 14

2 9 -13 35.4805 89.0606 7

9 -11 -13 212.08 58.4195 8

-11 2 -13 137.442 59.9432 8

2 9 -13 417.285 59.0412 2

-9 11 13 80.0564 76.9458 10

-2 -9 13 98.7227 89.7852 10

11 -2 13 80.3184 99.2675 10

9 -11 -16 296.994 51.7268 8

2 9 -16 425.352 55.2799 2

-1 -10 -18 606.084 62.2194 8

-10 11 -18 611.23 64.1378 2

11 -1 -18 695.56 63.2048 2

-10 11 -15 73.9739 41.5889 2

-10 11 -15 74.8867 29.8199 11

11 -1 -15 57.4798 34.9957 8

-1 -10 -15 86.1804 43.9701 8

-1 -10 -12 961.636 106.79 8

-10 11 -12 850.097 107.517 2

11 -1 -12 963.775 96.4646 8

-1 -10 -12 905.81 99.8905 6

-10 11 -12 837.38 97.2154 11

10 -11 12 778.605 149.74 10

1 10 12 653.983 147.905 10

-11 1 12 469.592 137.701 10

-1 -10 -917076.70 1394.86 8

11 -1 -916829.10 1385.17 8

-10 11 -917775.80 1396.48 11

11 -1 -920097.40 1455.49 9

10 -11 916143.90 1450.86 10

-11 1 917024.40 1487.68 10

11 -1 -918250.30 1460.73 7

-10 11 -916917.50 1401.73 2

-1 -10 -916528.60 1390.28 6

1 10 610476.90 941.866 4

10 -11 611510.30 968.553 10

-10 11 -6 9370.32 975.652 4

1 10 310976.50 1005.44 7

1 10 313984.90 1052.48 9

-11 1 314962.30 1075.34 10

10 -11 313732.40 1048.02 10

-10 11 -313021.80 1243.35 4

1 10 024618.60 2120.97 9

-1 -10 026854.10 2108.56 10

-10 11 025034.50 2251.26 4

1 10 -351892.20 4174.53 7

1 10 -356385.60 4148.61 9

-1 -10 353546.10 4074.02 10

-10 11 346875.60 4039.49 10

-10 11 349752.70 4118.38 4

-10 11 643815.80 3442.42 10

-1 -10 646455.90 3477.36 10

-10 11 644965.40 3450.06 4

1 10 -643318.50 3476.64 4

10 -11 -9 489.758 75.0994 8

10 -11 -9 546.246 76.9897 6

-10 11 9 743.766 131.457 10

-1 -10 9 614.867 146.876 10

1 10 -9 547.129 124.643 9

-11 1 -9 623.704 93.1728 8

-11 1 -9 557.785 76.6686 11

1 10 -9 840.674 204.004 7

-11 1 -12 304.791 73.7596 8

10 -11 -12 374.745 65.8238 8

1 10 -12 423.992 64.4906 2

-11 1 -12 297.431 67.8734 14

1 10 -12 517.296 65.8373 14

-11 1 -12 371.268 60.6819 11

-10 11 12 327.759 103.501 10

11 -1 12 242.029 115.613 10

-1 -10 12 264.981 112.78 10

1 10 -12 326.624 130.208 7

-11 1 -15 150.425 45.8897 2

1 10 -15 147.693 45.0482 2

10 -11 -15 150.316 45.6077 8

1 10 -15 75.7942 42.2173 14

1 10 -18 109.849 27.0024 2

10 -11 -18 104.584 23.5307 8

-11 11 -16 59.0504 23.7129 11

0 -11 -16 58.8861 37.0698 8

11 0 -16 123.654 35.624 2

-11 11 -16 99.5398 37.8894 2

-11 11 -13 1315.13 152.965 3

0 -11 -13 1600.56 144.789 8

-11 11 -13 1243.43 139.036 2

11 0 -13 1443.93 132.364 8

0 -11 -13 1322.44 133.874 6

-11 11 -13 1453.03 133.041 11

11 -11 13 1075.12 185.647 10

-11 0 13 944.15 180.874 10

0 11 13 1083.36 178.923 10

11 0 -10 2734.17 260.285 9

0 -11 -10 2282.99 209.169 8

-11 11 -10 2141.71 216.451 2

11 0 -10 1785.30 255.111 7

11 0 -10 2073.92 196.837 8

0 -11 -10 2308.64 204.922 6

-11 11 -10 2365.01 207.785 11

11 -11 10 1994.94 260.667 10

-11 0 10 1884.38 277.412 10

-11 11 -7 487.395 112.70 4

0 -11 -7 470.809 76.1721 8

0 11 7 264.765 99.1494 4

0 -11 -7 568.731 73.0303 6

-11 0 7 725.803 199.204 10

11 -11 7 495.406 131.665 10

-11 11 -7 635.319 80.3326 2

0 -11 -7 647.56 72.8631 1

-11 0 4 5867.59 504.145 10

11 -11 4 4592.86 465.679 10

-11 11 -4 4481.58 571.608 4

11 -11 1 1592.43 149.021 10

0 11 1 1790.25 204.537 9

0 11 1 1576.91 199.127 7

0 11 -220285.30 1673.07 7

0 -11 218234.60 1546.25 10

0 11 -218779.00 1599.23 9

-11 11 218671.10 1628.84 4

0 11 -523087.00 1773.49 7

-11 11 522417.10 1630.15 4

0 11 -518883.20 1676.26 4

0 -11 520627.90 1617.34 10

-11 11 518117.60 1609.38 10

11 0 518240.30 1540.45 9

0 11 -517597.60 1685.46 9

-11 0 -8 284.095 75.9458 8

-11 11 8 414.094 111.532 10

0 -11 8 352.091 132.14 10

0 11 -8 266.137 125.274 9

11 -11 -8 240.321 56.4302 8

-11 0 -8 318.676 59.1921 1

0 11 -8 355.917 57.2836 2

0 11 -8 559.657 197.993 7

11 -11 -8 272.15 55.0069 6

11 -11 -11 2213.10 205.708 8

0 11 -11 2160.69 317.932 7

-11 0 -11 2292.57 214.134 8

0 -11 11 1711.98 262.301 10

-11 11 11 1851.08 246.404 10

-11 0 -11 1711.64 198.81 14

0 11 -11 2277.59 201.639 14

0 11 -11 2989.25 211.216 2

-11 0 -11 2402.68 205.055 11

0 -11 14-13.0664 70.3224 10

11 0 14 -45.076 89.3889 10

-11 0 -14 7.60328 49.1961 8

11 -11 -14 44.3196 42.6482 8

0 11 -14 93.0457 45.7057 2

0 11 -14-2.75839 36.7295 14

11 -11 -17 44.4303 25.9829 8

0 11 -17 79.9562 29.4611 2

0 11 -17 80.8613 27.7036 3

12 -11 -16 42.639 31.5647 8

-1 12 -16-2.46819 32.9699 2

-11 -1 -13 599.422 97.0706 14

-1 12 -13 907.159 94.3858 14

-1 12 -13 993.304 102.453 2

-1 12 -13 533.632 158.318 7

1 -12 13 646.29 147.978 10

11 1 13 600.472 163.146 10

-12 11 13 575.012 125.248 10

12 -11 -13 989.828 100.668 8

-11 -1 -13 1085.29 97.3667 11

-11 -1 -13 862.342 106.268 8

-1 12 -10 1015.37 97.6294 14

-11 -1 -10 726.678 90.0381 14

1 -12 10 661.533 162.328 10

-12 11 10 363.334 121.452 10

-11 -1 -10 809.162 110.10 8

12 -11 -10 752.257 92.8638 8

-11 -1 -10 996.994 98.4998 11

-1 12 -10 697.773 86.0658 11

-1 12 -10 645.025 204.178 7

-1 12 -10 722.155 93.2245 2

-1 12 -7 2372.78 366.936 9

12 -11 -7 2477.62 225.263 8

-11 -1 -7 2555.07 226.502 1

12 -11 -7 2482.55 226.228 6

-12 11 7 2728.86 306.264 10

1 -12 7 2584.39 317.764 10

-1 12 -7 2544.72 227.016 2

-1 12 -7 2563.29 310.916 4

-12 11 7 3100.23 296.444 4

-1 12 -422867.10 1744.93 7

1 -12 420575.30 1542.60 10

-12 11 416643.80 1516.24 10

11 1 419312.60 1495.03 9

-1 12 -420147.70 1645.82 9

-1 12 -412495.30 1618.37 4

-12 11 416890.00 1559.57 4

1 -12 122257.90 1908.06 10

-1 12 -124526.80 1985.11 7

-12 11 125679.70 2042.64 4

-1 12 242858.00 3527.68 7

-12 11 -241596.60 3720.18 4

-11 -1 246100.90 3579.91 10

-1 12 245476.90 3573.33 9

-11 -1 5 8999.89 739.515 10

12 -11 5 7594.82 681.245 10

-12 11 -5 6219.71 720.539 4

-1 12 5 7546.17 682.246 4

-11 -1 8 1611.39 271.924 10

11 1 -8 1530.99 205.904 9

11 1 -8 1606.26 152.21 8

1 -12 -8 1681.18 162.606 8

1 -12 -8 1623.73 158.343 6

12 -11 8 1397.06 209.786 10

1 -12 -8 1835.88 159.45 1

-12 11 -8 1617.90 171.523 2

-12 11 -11 1833.69 156.636 11

1 -12 -11 1615.58 155.205 6

-1 12 11 1632.45 217.099 10

11 1 -11 1529.41 147.393 8

11 1 -11 1366.93 211.182 7

-12 11 -11 950.357 164.856 3

-11 -1 11 1224.34 217.95 10

12 -11 11 1136.78 201.318 10

1 -12 -11 1757.28 162.003 8

-12 11 -11 1819.12 161.233 14

-12 11 -11 1573.91 167.878 2

-12 11 -14 205.848 50.789 2

-11 -1 14 100.646 72.1936 10

12 -11 14-36.7795 70.3956 10

-1 12 14 32.5493 62.5096 10

-12 11 -14 225.53 56.2638 14

1 -12 -14 207.282 53.7304 8

11 1 -14 157.054 40.8805 8

-12 11 -14 124.453 39.7441 11

11 1 -17 178.187 32.2123 2

-12 11 -17 137.04 33.1932 2

1 -12 -17 145.548 31.6542 8

-2 12 -17 289.715 46.4133 2

12 -10 -17 363.765 44.3432 8

-2 12 -17 365.602 45.5885 3

12 -10 -14 3013.93 237.299 8

-10 -2 -14 2784.39 241.729 8

-2 12 -14 3038.49 239.516 2

-12 10 14 1713.36 262.277 10

2 -12 14 1905.77 279.857 10

-2 12 -14 2561.04 231.46 14

-2 12 -11 54.7763 36.239 11

-2 12 -11 92.4101 50.9048 2

-10 -2 -11 118.372 60.3529 8

12 -10 -11 66.5369 44.2948 8

-10 -2 -11 43.5276 48.2457 14

-2 12 -11 109.604 44.6339 14

2 -12 11 151.561 99.4655 10

-12 10 11 49.0883 78.6005 10

-2 12 -11 51.1354 132.527 7

-10 -2 -11 132.215 47.4137 11

-12 10 8 68.2701 89.8281 10

2 -12 8 48.505 107.54 10

-2 12 -8 22.136 112.615 9

12 -10 -8 59.8273 39.9269 8

-10 -2 -8 183.89 49.6102 1

-10 -2 -8 92.1874 55.7024 8

-10 -2 -8 118.386 36.2872 6

12 -10 -8 164.571 44.22 6

-2 12 -8 71.604 199.817 7

-2 12 -8 85.9846 41.9622 2

-2 12 -528511.20 2175.81 7

2 -12 525355.10 1918.12 10

-12 10 524302.80 1934.80 10

-2 12 -519507.80 2000.56 4

10 2 522021.70 1851.13 9

-2 12 -523418.60 2031.31 9

-12 10 522667.20 1915.53 4

-2 12 -2 2373.41 304.612 7

-12 10 2 1318.82 149.738 10

10 2 2 2120.94 198.499 9

2 -12 2 2106.21 196.052 10

-12 10 2 2205.81 291.797 4

-2 12 -2 1492.18 391.248 4

-2 12 -2 2014.36 253.628 9

-2 12 1 4986.36 450.451 9

-2 12 1 4482.39 412.326 7

-10 -2 443065.50 3080.34 10

-2 12 435604.10 3051.01 4

-12 10 -432943.60 3109.04 4

-10 -2 7 267.168 147.505 10

12 -10 7 98.3838 99.5654 10

10 2 -7-11.3482 91.1395 9

2 -12 -7 184.986 50.3272 8

-12 10 -7-24.3746 68.1196 4

-2 12 7 20.6691 75.0538 4

-12 10 -7 43.8328 45.4441 2

2 -12 -7 37.418 39.9306 1

10 2 -1026170.80 1795.56 9

2 -12 -1021257.80 1741.94 8

-12 10 -1020822.70 1753.48 2

10 2 -1022063.20 1734.13 8

10 2 -1021111.10 1813.23 7

-10 -2 1019130.70 1823.16 10

-2 12 1018765.10 1792.42 10

12 -10 1019388.30 1798.17 10

-12 10 -1023081.60 1745.14 14

2 -12 -1021070.70 1738.41 6

-12 10 -1021284.80 1740.03 11

-12 10 -13 307.648 65.2986 2

10 2 -13 252.125 50.2658 8

2 -12 -13 326.29 65.5969 8

12 -10 13 119.515 84.4303 10

-2 12 13 271.469 88.4865 10

-10 -2 13 253.08 98.1059 10

-12 10 -13 348.067 62.7493 14

-12 10 -13 301.319 74.692 3

2 -12 -13 282.656 56.065 6

-12 10 -13 300.721 53.5526 11

-12 10 -16-13.5501 33.0855 2

10 2 -16 15.5661 30.0241 2

2 -12 -16 37.2293 32.4077 8

-12 10 -16 3.05278 21.9606 11

-12 10 -16-18.2718 38.7892 14

-3 12 -18 30.8138 21.7196 2

-3 12 -15 185.298 49.2627 2

-3 12 -15 60.475 39.9349 14

12 -9 -15 189.46 44.5991 8

-9 -3 -15 195.49 49.8255 8

-9 -3 -12 8571.46 630.713 11

-3 12 -12 7235.60 624.368 11

12 -9 -12 8292.84 631.685 8

-9 -3 -12 7985.94 637.692 8

-9 -3 -12 6094.09 628.436 14

-3 12 -12 7944.74 628.514 14

-3 12 -12 8265.17 633.971 2

3 -12 12 6206.99 687.908 10

-12 9 12 6634.98 680.301 10

-3 12 -12 7496.31 763.05 7

-3 12 -9 7448.42 667.684 2

12 -9 -9 7692.29 665.309 8

-9 -3 -9 8161.13 675.246 8

-3 12 -910297.30 920.933 7

-9 -3 -9 8920.91 670.937 11

-3 12 -9 9326.79 673.835 14

-9 -3 -9 7386.43 662.083 6

-12 9 9 7972.95 735.60 10

3 -12 9 7856.61 747.244 10

-12 9 623405.40 1733.50 4

-9 -3 -621348.50 1660.92 1

-3 12 -620254.80 1662.96 2

-12 9 620084.70 1756.21 10

3 -12 621276.70 1743.77 10

-3 12 -619420.10 1782.38 4

-3 12 -357165.30 3872.25 7

9 3 347657.30 3683.85 9

-3 12 -348441.90 3768.71 9

-3 12 -333942.10 3887.01 4

-12 9 340273.60 3675.52 10

3 -12 347566.90 3699.90 10

-12 9 353413.00 3771.91 4

-12 9 030253.40 2478.90 4

-3 12 028673.90 2378.53 9

-3 12 028356.30 2356.82 7

9 3 027161.80 2368.01 9

-3 12 034993.40 2496.09 4

-3 12 3 2543.42 285.305 4

-12 9 -3 2030.27 340.423 4

9 3 -632729.30 2490.69 9

-12 9 -627074.90 2492.77 4

-3 12 630167.00 2483.21 4

3 -12 -630298.90 2424.02 8

12 -9 9 79.6864 94.0505 10

-9 -3 9 93.109 131.333 10

9 3 -9 285.706 83.4005 9

9 3 -9 185.099 104.212 7

9 3 -9 96.5199 45.2223 8

-12 9 -9 160.177 54.3029 11

-12 9 -9 103.47 65.7785 2

-12 9 -9 262.741 65.5695 14

3 -12 -12 32.4579 47.7193 6

-12 9 -12 3.45802 40.561 11

9 3 -12-6.03719 40.2007 8

-12 9 -12 67.5395 48.8726 14

-12 9 -12 56.9654 56.5313 2

-3 12 12 24.7572 75.8553 10

12 -9 12 41.6329 81.3111 10

-9 -3 12 30.3222 84.5627 10

9 3 -12 101.367 71.9379 7

-12 9 -12-7.38333 59.2529 3

-12 9 -15 1900.69 190.343 14

-12 9 -15 2019.22 185.254 11

9 3 -15 2600.09 193.952 2

-12 9 -15 2093.98 195.351 2

9 3 -18 110.49 24.8199 2

3 -12 -18 73.8125 23.491 8

-4 12 -16 90.0457 34.0118 3

-8 -4 -16 79.7883 38.5372 8

12 -8 -16 91.6312 33.7543 8

-4 12 -16 33.9366 38.1837 14

4 -12 13 1005.26 179.287 10

-12 8 13 1018.26 169.052 10

12 -8 -13 1393.73 132.932 8

-8 -4 -13 1398.69 140.281 8

-4 12 -13 1208.15 127.958 14

-4 12 -13 1216.68 123.501 11

-4 12 -13 1695.94 140.222 2

-8 -4 -10 855.015 92.4253 11

-4 12 -10 870.914 93.1261 11

12 -8 -10 1060.77 97.4803 8

-8 -4 -10 622.472 93.2001 8

-4 12 -10 872.333 98.9947 2

-4 12 -10 1168.57 259.319 7

-8 -4 -10 928.568 93.55 6

4 -12 10 658.201 156.902 10

-12 8 10 938.591 163.494 10

-4 12 -10 733.168 94.0872 14

-8 -4 -10 510.98 87.3889 14

-4 12 -7 6342.90 548.743 2

12 -8 -7 6962.93 547.552 6

12 -8 -7 6508.69 545.195 8

4 -12 7 6915.88 632.44 10

-12 8 7 7649.68 655.14 10

-4 12 -7 5381.31 618.582 4

-4 12 -452197.90 4923.36 4

-12 8 457870.40 4741.92 4

-12 8 456444.90 4720.54 10

4 -12 460950.40 4716.41 10

-4 12 -457379.30 4795.38 9

8 4 457877.40 4683.06 9

-4 12 -468538.60 4927.98 7

4 -12 115729.80 1276.19 10

-4 12 -114928.10 1311.50 7

-12 8 116688.70 1393.20 4

8 4 115389.30 1300.48 9

-12 8 -2 5198.49 595.39 4

-4 12 2 5608.20 545.603 4

-4 12 5 243.056 107.514 4

-12 8 -5 79.3698 108.515 4

8 4 -5 152.462 94.122 9

8 4 -8 578.585 141.161 7

-12 8 -8 591.684 92.169 8

8 4 -8 537.651 69.5399 8

8 4 -8 532.25 113.711 9

-12 8 -8 481.97 88.2278 2

-12 8 -11 626.093 101.767 2

8 4 -11 724.54 84.9222 8

-12 8 -11 695.352 92.9647 14

8 4 -11 854.91 90.8184 14

-12 8 -11 624.927 87.9225 11

8 4 -11 655.399 154.443 7

12 -8 11 698.241 152.041 10

-8 -4 11 451.173 134.384 10

-4 12 11 743.761 138.873 10

4 -12 -11 949.247 99.6564 6

-12 8 -14 324.347 53.0773 11

-12 8 -14 332.807 62.5565 2

-12 8 -14 440.147 65.8805 14

-8 -4 14 338.595 103.228 10

12 -8 14 249.384 102.199 10

-4 12 14 158.128 78.3336 10

8 4 -17 775.518 77.7956 2

12 -7 -17 65.3821 26.4571 8

-7 -5 -14 619.113 80.0614 8

12 -7 -14 703.173 77.0998 8

-5 12 -14 482.32 60.8052 11

-5 12 -14 539.836 71.3764 14

5 -12 14 445.043 112.926 10

7 5 14 499.599 131.686 10

-12 7 14 395.176 101.866 10

-12 7 11 1030.19 166.425 10

5 -12 11 790.80 163.484 10

-7 -5 -11 757.326 110.406 14

-5 12 -11 1357.69 119.043 14

-7 -5 -11 1171.04 120.522 8

12 -7 -11 978.777 109.566 8

-5 12 -11 950.414 107.529 11

-7 -5 -11 1360.09 112.97 11

-5 12 -11 1012.03 246.093 7

-7 -5 -11 917.08 107.999 6

12 -7 -8 122.414 42.119 8

-5 12 -8 143.26 50.1328 2

-7 -5 -8 139.735 47.7194 8

-12 7 8 126.59 110.39 10

5 -12 8 14.6857 95.5168 10

-5 12 -8 148.081 255.752 7

-7 -5 -8 138.739 42.9343 6

-5 12 -5123341.0 8076.95 7

-5 12 -580813.20 7894.27 4

-12 7 5110982.0 7838.64 10

5 -12 596368.60 7791.80 10

-12 7 5101681.0 7802.86 4

7 5 592113.60 7745.25 9

-12 7 2-34.6166 163.809 4

-5 12 -2 95.2999 345.621 4

7 5 2 120.363 58.3081 9

5 -12 2 222.787 64.254 10

-12 7 2 205.467 58.8049 10

-5 12 -2 189.65 105.795 7

-5 12 1148595.011255.00 4

7 5 -1123683.011185.90 9

-7 -5 1146791.011186.50 10

-5 12 1141679.011152.20 7

-5 12 4 2340.11 295.179 4

-12 7 -4 2065.86 308.548 4

7 5 -4 2626.31 296.156 9

-12 7 -7 4896.46 501.426 8

5 -12 -7 6640.86 499.686 6

7 5 -7 6255.97 552.839 9

-5 12 7 6681.02 562.645 4

-12 7 -10 389.587 80.3343 8

7 5 -10 317.562 61.1666 8

7 5 -10 306.902 84.7327 9

-5 12 10 469.398 113.191 10

12 -7 10 535.004 130.687 10

-12 7 -10 433.081 69.706 11

7 5 -10 272.848 112.694 7

-12 7 -10 645.561 83.3266 14

7 5 -10 529.921 64.1726 14

-12 7 -13 415.282 71.6436 2

-12 7 -13 481.423 68.954 14

12 -7 13 271.146 105.688 10

-5 12 13 365.061 96.3312 10

-7 -5 13 255.544 105.185 10

7 5 -13 352.214 109.505 7

-12 7 -13 308.394 60.3025 11

5 -12 -13 457.307 74.0758 8

-12 7 -16 443.466 58.9132 2

7 5 -16 438.382 55.9621 2

-12 7 -16 316.979 57.7995 14

5 -12 -16 410.755 58.2216 8

-12 7 -16 398.744 46.9271 11

12 -6 -18 39.3184 20.7307 2

-6 12 -18 30.5745 24.0819 2

-6 -6 -15 986.748 108.652 8

12 -6 -15 1063.88 105.178 8

-6 12 -15 961.044 103.384 14

-6 12 -12 249.752 59.5444 14

-6 -6 -12 368.379 71.3344 8

12 -6 -12 388.57 61.9269 8

-6 12 -12 259.091 52.4012 11

6 6 12 253.351 128.364 10

-12 6 12 199.473 94.4221 10

6 -12 12 236.373 103.584 10

-6 -6 -12 351.267 60.845 6

-6 -6 -9 2260.63 210.45 8

12 -6 -9 2279.35 203.489 8

12 -6 -9 1975.90 241.655 7

-6 12 -9 2142.42 441.495 7

-6 12 -9 2179.65 211.904 14

-6 -6 -9 2142.05 206.194 6

-12 6 9 2203.83 278.752 10

6 -12 9 2394.89 284.491 10

-6 -6 -9 2821.10 217.189 1

-6 12 -631262.70 2808.74 4

-6 -6 -632867.90 2683.92 1

6 -12 634795.90 2760.30 10

-12 6 638988.40 2815.24 10

-6 12 -3-54.2858 364.808 4

-6 12 -3 278.111 141.938 7

6 -12 3 237.351 76.6378 10

-12 6 3 222.435 76.159 10

6 6 3 248.434 68.5152 9

-12 6 3 370.949 136.279 4

6 6 033196.20 2851.10 9

-6 12 040388.60 2991.80 4

-12 6 033857.70 2929.51 4

-6 12 035344.40 2834.43 7

6 6 -3 2135.83 284.785 9

-6 -6 3 2675.91 287.705 10

-12 6 -3 2730.64 349.119 4

-6 12 3 3159.92 325.923 4

6 -12 -6 8409.49 703.732 8

6 -12 -6 8367.09 705.181 6

-6 12 6 9055.90 780.415 4

-6 -6 610662.10 835.699 10

6 6 -6 9013.50 772.407 9

6 6 -9 380.939 96.1456 9

6 -12 -9 639.338 79.1289 8

6 6 -9 441.517 139.521 7

6 -12 -9 618.251 78.6848 6

-6 12 9 547.696 120.769 10

-12 6 -9 532.458 75.6894 11

6 6 -9 500.959 68.3696 8

-12 6 -9 411.992 89.0196 8

6 -12 -12 7596.72 623.228 8

-12 6 -12 7018.00 626.322 8

6 6 -12 7458.28 617.017 8

6 6 -12 6532.29 695.712 7

6 6 -12 8545.76 621.303 14

-12 6 -12 7620.33 619.906 14

12 -6 12 7146.53 694.328 10

-6 12 12 6383.28 666.165 10

-12 6 -12 6892.04 629.726 2

-12 6 -12 7570.97 619.609 11

-12 6 -15-17.1384 49.4092 14

6 6 -15 35.8252 36.3282 2

-12 6 -15-17.1282 38.5398 2

6 -12 -15-11.8193 42.0704 8

-12 6 -15 43.1584 30.1555 11

6 -12 -18 639.579 67.3493 8

6 6 -18 795.563 69.8205 2

-5 -7 -16 905.554 88.8732 8

12 -5 -16 781.612 81.4171 8

-7 12 -16 847.922 87.6673 2

-7 12 -16 657.355 83.4823 14

-5 -7 -13 1060.79 112.715 8

12 -5 -13 1080.50 104.912 8

5 7 13 655.414 158.042 10

7 -12 13 762.00 149.059 10

-12 5 13 731.027 143.284 10

-7 12 -13 1030.49 108.544 2

-7 12 -13 900.713 98.3192 11

-7 12 -13 876.817 102.875 14

-7 12 -10 56.2105 40.6997 11

-7 12 -10 153.162 55.2587 14

7 -12 10 194.718 105.131 10

-12 5 10 74.335 87.4272 10

12 -5 -10 62.2769 37.4095 8

-5 -7 -10 103.163 48.0152 8

-5 -7 -10 191.711 48.0024 6

12 -5 -10 51.7506 55.1971 7

-7 12 -727816.00 2614.03 4

-5 -7 -732326.90 2548.44 6

-12 5 736236.20 2677.42 10

7 -12 732779.20 2625.59 10

-7 12 -737791.90 2944.22 7

-5 -7 -731863.80 2548.93 1

-5 -7 -730798.70 2552.12 8

5 7 486133.40 7247.66 9

-12 5 496933.20 7294.63 10

7 -12 484584.00 7263.82 10

-7 12 -494106.60 7367.72 7

-12 5 4105923.0 7321.20 4

-7 12 -484110.10 7468.91 4

-12 5 120076.70 1643.55 10

7 -12 119385.00 1612.24 10

-12 5 121173.70 1740.41 4

5 7 120489.90 1652.75 9

-7 12 -120806.70 1657.96 7

-5 -7 2 165.778 89.9646 10

-7 12 2 88.605 149.54 4

-12 5 -2 167.685 165.714 4

5 7 -532295.00 2863.54 9

-7 12 534733.30 2870.61 4

-5 -7 538179.20 2889.28 10

7 -12 -8 6.81944 41.8243 8

-7 12 8 10.6126 68.6546 10

-5 -7 8 36.3893 123.554 10

5 7 -8 14.5156 36.7501 8

-12 5 -8 5.59136 57.0566 8

7 -12 -8 59.2407 47.2283 6

5 7 -8 57.0132 71.8465 9

5 7 -8 127.275 104.841 7

-12 5 -11 9138.84 851.732 8

5 7 -1110401.60 843.135 8

5 7 -1110494.30 943.773 7

-5 -7 11 8903.40 914.814 10

-7 12 11 9426.42 893.761 10

12 -5 1110506.20 928.812 10

5 7 -1111600.30 844.13 14

-12 5 -11 9792.79 844.355 14

-12 5 -1110410.80 845.106 11

7 -12 -1110682.40 848.40 8

7 -12 -14 693.325 86.4646 8

5 7 -14 661.162 79.5805 2

-12 5 -14 667.071 87.805 2

-12 5 -14 686.41 76.6576 11

-7 12 14 425.779 107.398 10

12 -5 14 781.513 146.763 10

-12 5 -14 666.747 85.2331 14

5 7 -17 99.2282 30.9159 2

7 -12 -17 73.5139 28.5596 8

-8 12 -17 32.2967 28.7232 2

-4 -8 -17 24.0239 27.198 8

-8 12 -14 7.02996 41.1963 2

-8 12 -14 87.9045 45.2229 14

4 8 14 13.3766 67.8654 10

8 -12 14 12.5733 67.6594 10

-12 4 14 12.2053 66.8846 10

12 -4 -14 20.3463 36.5118 8

-4 -8 -14-22.0927 42.8151 8

-8 12 -14 7.16883 30.3305 11

8 -12 11 163.996 97.1506 10

-12 4 11 454.643 121.345 10

-4 -8 -11 282.21 60.9626 6

-8 12 -11 242.842 57.1968 11

-8 12 -11 335.234 66.4206 2

12 -4 -11 292.381 54.2273 8

-4 -8 -11 224.389 59.2845 8

-8 12 -11 178.561 61.1476 14

-8 12 -8 199.215 263.063 7

-4 -8 -8 212.818 53.7575 8

12 -4 -8 23.347 39.3311 8

-4 -8 -8 83.5453 44.6152 6

-4 -8 -8 135.606 48.3805 1

-8 12 -8 140.82 55.3822 2

8 -12 8 81.1866 103.25 10

-12 4 8 108.892 123.471 10

8 -12 5 5158.12 483.34 10

-12 4 5 5523.20 534.708 10

-8 12 -5 4310.70 579.576 4

-8 12 -2 6920.91 673.301 7

-12 4 2 7991.97 745.197 4

4 8 2 7335.74 652.327 9

-8 12 -2 7959.31 966.601 4

-12 4 2 8216.69 662.381 10

8 -12 2 7413.03 629.219 10

-8 12 114177.00 1374.13 4

-4 -8 116135.90 1288.81 10

4 8 -114919.20 1305.23 9

-4 -8 4 5093.31 477.222 10

-8 12 4 4992.64 442.247 10

4 8 -4 4545.15 489.303 9

-8 12 4 4510.55 489.985 4

4 8 -711878.70 974.656 4

4 8 -711803.70 993.333 9

8 -12 -710745.80 917.458 8

-8 12 712574.40 983.073 4

-8 12 711714.80 969.405 10

-4 -8 712189.50 1034.91 10

8 -12 -710959.90 919.711 6

4 8 -714671.70 1062.37 7

4 8 -710859.30 914.683 8

8 -12 -10 111.379 52.8837 6

8 -12 -10 93.921 45.3853 8

-12 4 -10 115.044 59.6036 8

4 8 -10 64.4215 41.7866 8

-12 4 -10 121.451 49.8815 14

4 8 -10 124.506 42.1124 14

-8 12 10 191.425 84.5359 10

-4 -8 10 249.411 113.239 10

-12 4 -10 92.4409 46.3081 11

4 8 -10 215.264 113.162 7

4 8 -13 1794.22 281.373 7

-12 4 -13 2262.57 222.442 8

-12 4 -13 2502.27 227.227 2

4 8 -13 2477.48 215.815 2

-12 4 -13 2468.18 218.45 14

4 8 -13 2923.53 220.363 14

-12 4 -13 2600.11 216.622 11

8 -12 -13 2329.73 218.398 8

-4 -8 13 1493.19 256.872 10

-8 12 13 1715.76 249.145 10

12 -4 13 2272.27 287.55 10

8 -12 -16 33.4166 30.5858 8

4 8 -16 59.7708 33.1341 2

12 -3 -18 159.058 28.1617 2

-9 12 -18 201.223 31.2535 2

12 -3 -15 91.0588 36.7008 8

-3 -9 -15 148.232 47.2778 8

-9 12 -15 63.2205 44.3707 14

-9 12 -15 122.506 42.0407 2

-9 12 -15 83.4816 29.1934 11

-9 12 -15 123.10 40.3825 3

9 -12 12 671.661 148.612 10

3 9 12 884.566 168.349 10

-12 3 12 818.133 159.833 10

-9 12 -12 1047.16 114.524 2

-3 -9 -12 1053.97 112.436 8

12 -3 -12 1111.90 102.753 8

-9 12 -12 865.17 105.141 14

-3 -9 -12 885.316 104.144 6

-9 12 -12 796.686 100.568 11

-9 12 -910974.50 963.761 2

12 -3 -911429.60 950.124 8

-3 -9 -912155.30 961.273 8

12 -3 -911309.10 1010.99 7

-3 -9 -911017.90 956.657 1

-9 12 -911873.90 961.364 11

-12 3 911223.30 1047.58 10

9 -12 910221.20 1014.99 10

-3 -9 -911589.20 955.731 6

-9 12 -914222.00 967.316 14

9 -12 6 198.191 115.235 10

-12 3 6 774.266 191.685 10

-9 12 -6 563.334 76.2075 2

-9 12 -6 299.583 152.124 4

-3 -9 -6 712.139 72.8171 1

3 9 3 155.581 71.6043 9

-9 12 -3 180.561 337.335 4

9 -12 3 172.596 61.154 10

-12 3 3 220.056 94.2604 10

3 9 020797.90 1825.37 9

-9 12 023292.30 1988.46 4

-3 -9 022550.60 1818.23 10

-12 3 022697.10 1929.29 4

-9 12 3 249.50 138.125 4

-3 -9 3 188.824 95.2948 10

-9 12 3 237.137 80.2241 10

3 9 -3 328.945 151.146 9

9 -12 -615777.50 1297.11 8

9 -12 -615485.00 1299.54 6

-9 12 616739.60 1373.59 4

-9 12 617877.20 1355.02 10

-3 -9 619199.30 1416.94 10

3 9 -615085.10 1389.00 9

9 -12 -9 57.2021 47.9061 8

-12 3 -9 29.2278 59.0877 8

3 9 -9 63.4475 127.875 7

-12 3 -9 163.135 51.0809 11

-3 -9 9 156.40 116.815 10

-9 12 9 64.9389 79.5426 10

9 -12 -9 107.476 49.5548 6

3 9 -9 50.6045 72.0794 9

9 -12 -12 141.024 51.9433 8

-12 3 -12-23.6108 60.096 8

12 -3 12 16.5627 98.6456 10

-9 12 12 11.3671 75.5326 10

-3 -9 12 60.2595 89.7491 10

3 9 -12 49.3102 42.9911 14

-12 3 -12 93.7441 53.4401 14

3 9 -12 88.3616 46.5591 2

3 9 -12 115.424 107.386 7

-12 3 -12 50.7988 45.5954 11

9 -12 -15 970.061 96.9267 8

-12 3 -15 915.635 90.7879 11

-12 3 -15 991.784 102.904 2

3 9 -15 1210.91 100.794 2

3 9 -18 11.8972 19.0186 3

3 9 -18 31.2146 21.1331 2

9 -12 -18 12.8235 18.9707 8

-2 -10 -16 194.425 44.2152 8

-10 12 -16 183.185 30.3758 11

-10 12 -16 183.21 43.4552 2

-10 12 -16 227.102 49.1084 14

2 10 13 1487.41 216.599 10

10 -12 13 1461.00 214.926 10

-12 2 13 1361.66 215.928 10

-10 12 -13 1803.75 168.412 14

12 -2 -13 1721.03 162.321 8

-2 -10 -13 1860.44 165.455 6

-2 -10 -13 2020.35 174.322 8

-10 12 -13 1684.06 170.111 2

-10 12 -13 1712.38 162.383 11

-10 12 -13 1729.20 179.773 3

12 -2 -10 21.4931 72.6417 9

-10 12 -10 139.234 52.1858 11

12 -2 -10 64.898 41.828 8

-2 -10 -10 157.806 55.7566 8

12 -2 -10 174.495 82.0898 7

-2 -10 -10 94.7571 48.5902 6

-10 12 -10 142.363 58.5671 2

-12 2 10 83.8968 108.852 10

10 -12 10 173.843 97.3189 10

-2 -10 -719584.90 1475.95 8

-10 12 -714677.80 1524.11 4

-2 -10 -717880.90 1469.30 6

10 -12 718144.90 1537.58 10

-12 2 719555.70 1604.35 10

-10 12 -717589.80 1477.26 2

-2 -10 -718913.70 1469.95 1

2 10 717583.20 1507.35 4

-10 12 -4 817.616 304.053 4

10 -12 4 835.213 134.374 10

-12 2 4 764.914 161.141 10

2 10 1 6496.19 546.735 9

10 -12 1 5673.59 482.635 10

-10 12 2 3903.64 475.201 4

-2 -10 2 4033.50 383.258 10

2 10 -5 6117.77 583.225 7

-10 12 5 4335.23 483.764 4

2 10 -5 5327.06 542.406 9

12 -2 5 4540.04 407.181 9

-10 12 5 5816.55 476.754 10

-2 -10 5 5588.04 501.314 10

10 -12 -8 5540.36 474.482 8

2 10 -8 7209.93 638.291 7

-12 2 -8 4913.77 490.836 8

-10 12 8 5331.07 520.923 10

-2 -10 8 5785.97 575.919 10

10 -12 -8 6213.61 477.623 6

2 10 -8 5618.13 555.494 9

-12 2 -11 218.384 52.3756 11

10 -12 -11 96.9251 51.3145 8

-12 2 -11 166.643 65.4864 8

10 -12 -11 149.23 55.1641 6

2 10 -11 169.672 51.1386 2

-12 2 -11 257.821 54.0265 14

2 10 -11 135.305 46.5535 14

-10 12 11 45.3572 79.7698 10

-2 -10 11 79.3016 99.3411 10

2 10 -11 213.058 127.358 7

-12 2 -14 347.723 63.6648 14

2 10 -14 475.292 62.9788 14

-12 2 -14 401.566 67.891 2

2 10 -14 231.789 58.0113 2

-12 2 -14 465.969 58.4028 11

10 -12 -14 255.785 58.27 8

-12 2 -14 353.919 68.1131 8

-2 -10 14 326.766 103.492 10

12 -2 14 482.537 122.605 10

2 10 -17 21.239 24.8548 2

10 -12 -17 16.1313 25.7591 8

-11 12 -17 755.012 79.3805 2

12 -1 -17 873.188 77.8609 2

-1 -11 -17 691.017 77.1346 8

-11 12 -14 32.6983 46.1622 3

-12 1 14-37.1226 70.1929 10

1 11 14 11.5696 65.6307 10

11 -12 14 12.2875 67.3392 10

-1 -11 -14 50.0903 46.5895 8

12 -1 -14 15.9758 35.9762 8

-11 12 -14 29.8091 43.3322 2

-11 12 -14 15.4577 31.1432 11

-12 1 11 5391.98 624.417 10

11 -12 11 5793.39 615.084 10

12 -1 -11 6836.71 554.747 8

12 -1 -11 6153.15 616.024 7

-1 -11 -11 7557.73 568.274 8

-11 12 -11 6427.92 559.282 11

-1 -11 -11 7149.73 561.103 6

-11 12 -11 6430.50 569.841 2

-1 -11 -8 38.1037 46.7211 8

-1 -11 -8 19.308 40.853 6

11 -12 8 64.4742 89.5862 10

-12 1 8 103.084 129.864 10

-1 -11 -8 37.9081 43.0644 1

-11 12 -8 33.3296 53.3609 2

12 -1 -8-2.56939 35.4458 8

11 -12 5 1814.71 219.19 10

-12 1 5 1948.01 263.672 10

-11 12 -5 1151.99 267.89 4

-12 1 2 4478.41 479.642 10

-11 12 -2 4902.34 688.826 4

11 -12 2 5016.09 440.193 10

1 11 2 6446.55 492.545 9

-1 -11 180778.40 6491.02 10

-11 12 180754.00 6617.57 4

-11 12 4 5577.13 547.419 4

1 11 -4 5320.49 600.097 7

1 11 -4 5607.37 591.487 9

-1 -11 4 5990.72 517.852 10

-11 12 4 4427.58 487.883 10

12 -1 4 5560.43 462.881 9

1 11 -7 2020.19 300.62 9

11 -12 -7 1968.13 194.759 8

-11 12 7 2156.58 253.834 10

-1 -11 7 2006.94 286.893 10

11 -12 -7 2307.42 199.038 6

-12 1 -7 2147.67 195.671 1

1 11 -7 2413.22 197.596 2

-11 12 7 2351.04 259.34 4

1 11 -7 1553.25 252.835 4

11 -12 -10 5082.04 414.439 8

-12 1 -10 4433.72 425.953 8

1 11 -10 5232.18 547.189 7

-12 1 -10 5352.93 416.737 11

11 -12 -10 5309.38 416.191 6

-12 1 -10 3934.74 407.66 14

1 11 -10 5270.29 412.018 14

-1 -11 10 4108.06 487.428 10

-11 12 10 4207.27 456.44 10

1 11 -10 5208.33 413.917 2

1 11 -13 80.9025 46.585 2

-12 1 -13-49.0049 51.5277 14

1 11 -13 60.9384 39.7682 14

-11 12 13 64.8728 68.1097 10

-1 -11 13-14.1618 82.9595 10

12 -1 13-48.8779 106.702 10

-12 1 -13 21.933 54.519 8

11 -12 -13-41.0988 49.5369 8

1 11 -13-37.6453 101.278 7

-12 1 -13 76.1363 42.6769 11

1 11 -16 722.745 74.3829 2

11 -12 -16 585.148 70.9571 8

12 0 -18 12.5998 18.3594 2

0 -12 -18 10.9032 18.9225 8

-12 12 -15 77.7718 41.1192 2

-12 12 -15 45.569 28.6421 11

0 -12 -15 81.3848 40.2756 8

0 12 12 209.341 98.257 10

12 -12 12 171.139 88.9638 10

-12 0 12 60.3839 90.4077 10

0 -12 -12 201.842 51.6596 6

-12 12 -12 22.4301 66.0762 3

12 0 -12 99.3789 68.8715 7

-12 12 -12 105.177 55.8834 2

0 -12 -12 148.02 51.9883 8

12 0 -12 109.195 43.6808 8

-12 12 -12 175.756 48.3363 11

-12 0 931370.20 2821.21 10

12 -12 930061.30 2776.18 10

0 -12 -934716.20 2720.77 6

12 0 -936518.60 2780.67 9

-12 12 -932465.70 2724.55 11

0 -12 -935224.90 2725.73 8

12 0 -934301.70 2794.14 7

12 0 -934053.60 2714.47 8

-12 12 -933864.00 2725.88 14

-12 12 -933278.20 2733.60 2

0 -12 -626745.10 2046.37 8

0 -12 -626117.50 2042.62 6

12 -12 625169.50 2106.09 10

-12 0 631950.10 2200.51 10

0 -12 -624711.20 2040.16 1

0 12 625323.50 2099.89 4

-12 12 -624490.60 2045.17 2

-12 12 -621041.40 2126.60 4

0 12 340282.80 3306.13 7

-12 12 -342875.80 3549.49 4

12 -12 342067.20 3346.15 10

-12 0 345511.30 3383.14 10

-12 12 0 308.802 250.20 4

0 12 0 142.468 99.5368 9

0 12 0 240.746 121.003 7

0 12 -334165.80 2111.60 7

12 0 328653.80 1898.52 9

0 -12 329074.10 1924.97 10

0 12 -335143.50 2056.67 9

-12 12 329256.00 1998.27 4

0 -12 6 9556.25 863.407 10

-12 12 6 9310.67 852.783 10

0 12 -6 9854.31 894.029 4

0 12 -6 7191.01 894.559 9

12 -12 -6 8618.19 771.443 8

0 12 -6 9520.48 774.122 2

-12 0 -610455.00 774.594 1

-12 12 611668.30 867.622 4

12 -12 -6 9307.87 774.421 6

-12 12 9-35.6185 78.7696 10

0 -12 9-86.1866 111.498 10

0 12 -9-15.5691 82.2504 9

12 -12 -9-17.3282 45.482 8

-12 0 -9 72.4346 63.8039 8

0 12 -9 113.566 46.6856 14

-12 0 -9 50.1827 42.3981 11

0 12 -9 132.955 140.422 7

0 12 -9 103.87 44.5821 2

12 -12 -9 13.6185 40.6046 6

12 -12 -12 1771.80 158.624 8

-12 0 -12 1808.62 170.567 8

-12 12 12 944.922 184.796 10

0 -12 12 1197.05 208.071 10

12 0 12 1651.07 240.565 10

-12 0 -12 1154.20 150.139 14

0 12 -12 1577.85 151.776 14

-12 0 -12 1831.71 156.338 11

0 12 -12 2193.11 162.625 2

0 12 -12 1305.16 247.224 7

0 12 -15 507.587 67.805 14

0 12 -15 580.979 71.5333 2

12 -12 -15 603.30 71.4039 8

12 -12 -18 173.642 27.6604 8

0 12 -18 241.47 31.5573 2

13 -12 -17 51.0796 24.5899 8

-1 13 -17 33.9489 27.3784 2

-1 13 -17 70.8499 26.5953 3

12 1 14 58.7865 86.0873 10

1 -13 14-12.7497 64.681 10

-1 13 -14 28.6866 36.2406 14

-1 13 -14 47.3046 42.2088 2

-12 -1 -14 53.4919 45.3002 2

-12 -1 -14-65.5873 49.9041 8

13 -12 -14 12.0593 39.2502 8

-1 13 -11 697.256 79.8861 11

-12 -1 -11 709.477 86.868 11

-1 13 -11 749.779 88.1774 2

13 -12 -11 576.201 84.5121 8

-12 -1 -11 732.266 105.059 8

-12 -1 -11 511.557 83.857 14

-1 13 -11 851.451 87.8324 14

1 -13 11 547.033 146.608 10

-13 12 11 653.753 125.527 10

-1 13 -11 698.511 195.607 7

-1 13 -814919.40 1437.12 9

13 -12 -816190.00 1323.12 8

-13 12 817139.40 1398.97 10

1 -13 817730.60 1427.68 10

-1 13 -820477.60 1564.15 7

-12 -1 -814045.00 1339.25 8

-12 -1 -816262.70 1323.97 1

13 -12 -816768.40 1323.71 6

-1 13 -816953.00 1324.66 2

1 -13 5 293.24 125.362 10

-13 12 5 147.657 100.192 10

12 1 5 249.408 55.1582 9

-1 13 -5 447.193 233.87 9

-1 13 -5 108.486 251.932 7

-1 13 -5 306.015 194.211 4

-13 12 5 280.009 125.489 4

-1 13 -213677.70 1176.10 9

12 1 212073.60 1105.29 9

1 -13 213351.30 1110.17 10

-13 12 213165.60 1204.79 4

-1 13 -216193.00 1251.86 7

-1 13 126133.50 1845.27 9

13 -12 120416.90 1764.18 10

-1 13 123776.30 1809.46 7

-12 -1 126441.20 1849.76 10

-13 12 -468899.70 6248.19 4

-12 -1 482736.30 6180.43 10

13 -12 473764.60 6138.82 10

-1 13 477897.30 6163.60 4

-1 13 7 5123.88 486.299 4

1 -13 -7 4928.16 428.561 1

1 -13 -7 5587.04 436.491 8

13 -12 7 5624.47 503.442 10

1 -13 -7 5458.45 432.445 6

-13 12 -7 4804.92 430.257 3

-13 12 -7 4185.22 493.236 4

-13 12 -7 4983.50 437.088 2

-13 12 -10 2032.65 254.249 3

12 1 -10 2951.39 295.888 9

1 -13 -10 2863.74 248.809 8

-13 12 -10 2605.93 260.888 2

12 1 -10 2760.14 312.28 7

12 1 -10 2792.30 237.642 8

-13 12 -10 2524.90 243.931 11

1 -13 -10 2763.15 244.38 6

13 -12 10 2501.32 300.504 10

-12 -1 10 2409.35 327.943 10

-13 12 -10 3178.14 253.247 14

-13 12 -13 -4.6606 49.4679 2

12 1 -13-16.1335 35.2441 8

1 -13 -13 11.4098 46.1138 8

-13 12 -13 60.1333 41.6035 11

1 -13 -13-3.23738 43.2102 6

-12 -1 13 13.8455 67.2563 10

13 -12 13 50.6422 70.6077 10

-1 13 13 35.0312 73.5581 10

-13 12 -13 13.4024 60.1058 3

-13 12 -13 83.7105 52.1989 14

1 -13 -16 4.70807 30.8364 8

12 1 -16-6.15849 26.3833 2

-13 12 -16-1.49334 21.2645 11

-13 12 -16 2.62232 33.0367 2

-13 12 -16-48.8393 39.0884 14

13 -11 -18 181.763 26.4304 8

-2 13 -18 197.324 30.9918 2

-11 -2 -15 465.208 73.5024 8

13 -11 -15 646.245 72.9588 8

-2 13 -15 723.731 75.7212 2

-2 13 -15 573.222 70.328 14

-11 -2 -12 3997.25 430.408 14

-2 13 -12 5612.84 431.831 14

13 -11 -12 5221.36 434.296 8

-2 13 -12 5567.16 436.909 2

-13 11 12 4245.98 476.92 10

2 -13 12 4185.90 495.251 10

-11 -2 -12 6052.80 436.412 11

-2 13 -12 4883.25 427.302 11

-11 -2 -12 5707.94 446.78 8

-2 13 -12 4934.32 555.059 7

-11 -2 -9 259.489 56.055 11

-2 13 -9 362.445 63.9268 14

-13 11 9 237.375 96.6037 10

2 -13 9 183.063 113.374 10

13 -11 -9 267.143 53.7208 6

-11 -2 -9 210.769 44.1058 6

13 -11 -9 278.096 54.8305 8

-2 13 -9 232.407 55.904 2

-2 13 -9 161.341 174.709 7

-11 -2 -9 253.804 70.9993 8

-11 -2 -9 172.572 58.9316 1

-13 11 6 1705.18 246.122 10

-2 13 -6 1609.62 324.272 9

13 -11 -6 1465.90 147.751 8

-11 -2 -6 1447.54 148.214 1

13 -11 -6 1612.78 151.695 6

-2 13 -6 1661.34 291.454 4

-13 11 6 1770.61 226.299 4

-2 13 -6 2431.23 468.74 7

2 -13 6 2633.79 262.551 10

-2 13 -6 1743.28 154.073 2

-13 11 3 3523.84 387.401 10

-13 11 3 6042.17 522.895 4

-2 13 -3 2890.12 585.25 4

-2 13 -3 6180.62 626.241 7

-2 13 -3 6043.90 538.065 9

11 2 3 5745.87 423.462 9

2 -13 3 6061.11 447.164 10

-2 13 0 6922.62 631.521 9

-13 11 0 7371.42 763.974 4

-2 13 0 6673.20 618.135 7

-2 13 3 3900.92 409.96 4

-11 -2 3 3852.86 395.286 10

-13 11 -3 3738.25 507.287 4

2 -13 -6 252.237 51.5669 8

-13 11 -6 99.4449 101.501 4

-2 13 6 11.7331 96.396 4

2 -13 -6 235.672 46.0896 1

-11 -2 6 296.316 179.125 10

13 -11 6 238.522 108.285 10

13 -11 9 2507.25 340.379 10

-11 -2 9 2878.78 389.884 10

-13 11 -9 2905.93 296.057 3

11 2 -9 4040.10 355.894 9

2 -13 -9 3492.84 296.368 8

11 2 -9 3880.07 377.933 7

11 2 -9 3405.60 285.895 8

2 -13 -9 3460.30 292.637 6

-13 11 -9 3175.48 295.742 11

-13 11 -9 3550.62 301.23 14

-13 11 -9 3182.57 307.027 2

2 -13 -12 181.98 55.2012 6

-13 11 -12 167.295 64.9918 2

11 2 -12 189.547 47.2777 8

2 -13 -12 267.151 60.6627 8

-13 11 -12 326.557 60.3712 14

-13 11 -12 193.173 78.6055 3

-2 13 12 98.0316 74.8492 10

13 -11 12 239.676 96.04 10

-11 -2 12 137.774 95.3062 10

11 2 -12 259.952 96.5436 7

-13 11 -12 245.571 52.8059 11

-13 11 -15 454.967 59.0471 11

-13 11 -15 513.615 70.0054 14

2 -13 -15 514.211 70.1253 8

-13 11 -15 434.248 68.5603 2

11 2 -15 651.253 65.7778 2

11 2 -18 84.0065 21.4702 2

2 -13 -18 84.4255 22.2638 8

13 -10 -16 470.953 56.8065 8

-3 13 -16 486.359 58.6144 3

-3 13 -16 510.798 61.1569 2

-3 13 -16 345.317 56.7378 14

3 -13 13 192.973 101.956 10

-13 10 13 103.457 66.7863 10

-3 13 -13 168.288 50.9467 2

-3 13 -13 190.041 47.9496 14

13 -10 -13 183.097 51.4926 8

-10 -3 -13 255.466 62.8667 8

-10 -3 -10 5147.16 377.563 11

-3 13 -10 4348.89 371.134 11

-10 -3 -10 4379.53 368.125 6

13 -10 -10 4578.39 373.344 8

-10 -3 -10 4738.50 385.793 8

-3 13 -10 5905.33 595.876 7

-3 13 -10 4493.75 374.248 14

-10 -3 -10 3188.66 366.51 14

-13 10 10 4459.14 435.996 10

3 -13 10 3355.34 437.499 10

-3 13 -10 4768.49 377.356 2

3 -13 7 475.732 137.568 10

-13 10 7 233.712 132.202 10

-3 13 -7 95.268 190.852 9

13 -10 -7 214.82 50.8339 8

-3 13 -7 509.051 139.739 4

-10 -3 -7 438.854 60.5874 1

13 -10 -7 355.132 59.6097 6

-13 10 7 346.873 107.79 4

-3 13 -7 433.04 60.9077 2

-3 13 -4 6298.33 700.412 7

-3 13 -4 5029.40 699.215 4

10 3 4 5164.53 441.499 9

-3 13 -4 4263.12 558.187 9

3 -13 4 6019.39 485.244 10

-13 10 4 4001.88 453.741 10

-13 10 4 6096.70 524.845 4

3 -13 1 4827.54 424.078 10

10 3 1 4409.92 438.286 9

-13 10 1 5675.25 566.268 4

-3 13 -1 4615.93 466.731 7

-3 13 213826.80 1130.98 7

-3 13 214691.60 1226.37 4

-13 10 -212709.40 1292.30 4

-13 10 -5 5277.18 545.545 4

-3 13 5 5818.02 514.489 4

-10 -3 5 5065.26 533.448 10

10 3 -5 4752.46 496.605 9

10 3 -8 491.351 110.951 9

-13 10 -8 235.792 68.221 2

10 3 -8 306.196 50.5842 8

13 -10 8 205.019 100.464 10

-10 -3 8 310.913 156.087 10

-13 10 -8 178.114 48.9758 3

3 -13 -8 262.941 58.4286 8

10 3 -11 91.9679 91.1994 7

13 -10 11 13.6597 91.6885 10

-3 13 11-12.4351 70.5577 10

-10 -3 11-16.6323 86.1363 10

3 -13 -11 22.2486 50.5492 6

3 -13 -11 53.4154 53.6925 8

10 3 -11 14.8847 40.3686 8

-13 10 -11 18.9887 44.0202 11

-13 10 -11 43.4244 50.2771 14

-13 10 -11 120.584 67.5625 2

-13 10 -11-7.18465 70.2163 3

-10 -3 14 50.7526 72.8687 10

-3 13 14 132.879 62.7722 10

13 -10 14 86.5575 77.6744 10

-13 10 -14 210.548 53.2585 14

-13 10 -14 166.015 42.3271 11

-13 10 -14 130.462 49.8411 2

10 3 -17 33.7635 25.8924 2

3 -13 -17 34.4615 26.2313 8

13 -9 -17 123.961 28.9181 8

-4 13 -17 114.719 35.0089 2

13 -9 -14 26.9979 39.3108 8

-9 -4 -14 11.198 48.9053 8

-4 13 -14 62.4528 43.8471 14

-13 9 14-10.8314 59.3596 10

4 -13 14-63.7785 71.1374 10

-4 13 -14 23.1027 43.6492 2

-13 9 11 103.081 79.4551 10

4 -13 11 119.523 96.7211 10

-9 -4 -11 184.781 46.2624 6

-4 13 -11 200.564 54.6673 2

-9 -4 -11 253.119 62.7422 8

13 -9 -11 173.518 51.2333 8

-4 13 -11 314.727 57.4998 14

-9 -4 -11 136.093 52.0832 14

-9 -4 -11 237.995 53.057 11

-4 13 -11 256.336 159.634 7

-4 13 -11 224.671 49.4327 11

13 -9 -8 605.343 76.9077 8

-4 13 -8 683.282 84.8887 2

-9 -4 -8 642.957 76.5411 6

-13 9 8 615.079 151.398 10

4 -13 8 611.003 152.25 10

-9 -4 -8 709.103 91.6841 8

-4 13 -8 512.789 294.16 7

-4 13 -578117.10 5488.25 7

-13 9 565843.50 5180.85 4

-13 9 569943.80 5204.26 10

4 -13 565946.80 5168.14 10

-4 13 -559157.60 5313.48 4

-4 13 -558731.90 5291.04 9

9 4 561081.70 5118.41 9

-13 9 2105400.0 7492.65 4

-13 9 278129.40 7372.81 10

4 -13 299214.80 7393.09 10

-4 13 -296782.70 7704.93 4

-4 13 -2105622.0 7476.96 7

-4 13 -297445.10 7456.18 9

9 4 298704.40 7402.20 9

9 4 -129437.10 2568.69 9

-4 13 134202.40 2643.55 4

-4 13 131495.40 2533.20 7

9 4 -4 1073.50 162.808 9

-4 13 4 851.408 165.961 4

-13 9 -4 551.947 188.54 4

-4 13 743463.70 3376.66 4

-13 9 -740953.50 3381.21 4

-13 9 -738743.10 3313.19 2

4 -13 -740599.10 3309.31 6

4 -13 -742763.00 3309.95 1

9 4 -745210.70 3374.11 9

9 4 -10 1041.30 156.558 9

9 4 -10 1267.59 202.667 7

9 4 -10 1297.70 118.91 14

-13 9 -10 1468.23 134.641 14

-13 9 -10 1335.31 128.374 11

-13 9 -10 1099.87 141.697 2

-13 9 -10 694.576 126.125 3

-9 -4 10 1431.03 223.654 10

-4 13 10 919.676 165.449 10

13 -9 10 1119.65 182.48 10

9 4 -10 1167.94 119.02 8

9 4 -13 54.9277 74.0034 7

-13 9 -13 97.1879 50.4948 14

-4 13 13-11.1458 65.4224 10

13 -9 13 13.5098 75.4502 10

-9 -4 13 169.682 87.95 10

-13 9 -13 53.2231 55.4992 2

-13 9 -13 41.4903 38.4894 11

4 -13 -13 60.7073 46.6537 6

-13 9 -16 196.937 32.244 11

9 4 -16 283.54 40.9582 2

-13 9 -16 206.125 42.4693 2

-13 9 -16 173.49 45.1282 14

13 -8 -15 423.457 59.6196 8

-8 -5 -15 459.782 63.4639 8

-5 13 -15 344.088 59.3221 14

13 -8 -12 4753.20 371.726 8

-8 -5 -12 4641.54 378.868 8

-5 13 -12 4378.20 369.49 14

-5 13 -12 4109.12 366.115 11

-13 8 12 3991.34 427.513 10

5 -13 12 3357.21 422.369 10

-5 13 -12 4328.00 374.561 2

13 -8 -9 7536.55 651.656 8

-8 -5 -9 7874.78 660.261 8

-5 13 -9 9615.47 953.651 7

13 -8 -9 7006.33 683.034 7

-8 -5 -9 8449.38 657.48 11

-8 -5 -9 7853.63 653.212 6

-13 8 9 7547.84 729.257 10

5 -13 9 7223.03 728.292 10

-5 13 -9 8510.98 661.257 14

-5 13 -9 8115.14 659.84 2

-5 13 -6 4425.72 544.916 4

-13 8 6 5337.32 481.087 4

-13 8 6 4984.27 522.768 10

5 -13 6 4513.94 475.198 10

-5 13 -6 4861.51 410.135 2

-13 8 327071.90 2514.14 10

8 5 332192.80 2519.53 9

-5 13 -330924.70 2596.11 9

-13 8 332936.60 2592.32 4

5 -13 332429.30 2526.83 10

-5 13 -336977.20 2646.76 7

-5 13 -330076.20 2906.49 4

-5 13 043072.00 3538.57 7

-5 13 053278.70 3713.73 4

-13 8 040456.50 3639.80 4

8 5 043255.50 3557.95 9

-13 8 -3 4979.12 596.206 4

-5 13 3 6394.46 572.58 4

-13 8 -6 75.6328 97.8191 4

5 -13 -6 22.205 41.2657 6

8 5 -6-11.7769 95.247 9

-5 13 6-52.2314 111.499 4

-13 8 -9 1388.20 148.165 14

-5 13 9 1399.54 190.898 10

8 5 -9 1458.56 184.367 9

-13 8 -9 1277.94 157.029 2

8 5 -9 1393.28 135.575 8

8 5 -9 1747.41 232.035 7

-13 8 -9 1387.24 156.35 8

-13 8 -9 1446.17 144.058 11

13 -8 9 1156.05 194.024 10

-5 13 12 233.276 93.4347 10

13 -8 12 157.851 101.741 10

-8 -5 12 46.079 90.8692 10

8 5 -12 325.391 54.818 8

8 5 -12 346.974 114.965 7

-13 8 -12 331.998 74.9019 2

-13 8 -12 238.293 55.3411 11

-13 8 -12 314.917 60.9184 14

8 5 -12 202.684 55.757 14

8 5 -15 626.391 68.8224 2

-13 8 -15 494.596 71.6644 2

-13 8 -15 531.419 61.4352 11

-13 8 -15 390.114 69.0313 14

8 5 -18 26.7248 20.5666 2

13 -7 -16 64.6867 32.5401 8

-7 -6 -16 83.0021 35.9074 8

-6 13 -16 61.3964 31.982 3

-6 13 -16 51.187 38.7719 14

13 -7 -13 649.665 71.8487 8

-7 -6 -13 624.192 81.2578 8

-6 13 -13 357.016 67.6756 14

-6 13 -13 441.231 60.7121 11

6 -13 13 348.591 106.71 10

-13 7 13 275.46 101.36 10

7 6 13 418.406 133.216 10

13 -7 -10 2570.47 243.324 8

-7 -6 -10 2809.87 252.527 8

-6 13 -10 2483.96 450.085 7

-6 13 -10 2891.63 253.27 14

-7 -6 -10 2744.39 246.557 6

-6 13 -10 3152.72 253.135 11

6 -13 10 2892.49 327.979 10

-13 7 10 2368.85 309.95 10

-6 13 -7 3567.23 324.519 2

-6 13 -7 3269.10 405.61 4

6 -13 7 3856.47 405.519 10

-13 7 7 4236.98 443.149 10

-7 -6 -7 3585.03 319.693 1

-7 -6 -7 3952.09 320.253 6

13 -7 -7 3724.39 317.815 8

-7 -6 -7 3918.06 327.336 8

-6 13 -4 6305.22 642.696 7

-6 13 -4 6135.89 819.24 4

7 6 4 5102.02 467.392 9

-13 7 4 4833.12 496.794 10

6 -13 4 5439.32 490.447 10

-13 7 4 6051.87 540.395 4

6 -13 127445.10 2286.43 10

-13 7 129775.30 2413.39 4

7 6 128840.60 2322.66 9

-6 13 -129460.30 2328.62 7

-7 -6 253077.50 4119.97 10

-6 13 250575.90 4165.36 4

-13 7 -246500.80 4190.16 4

-13 7 -568318.10 6041.46 4

-7 -6 584716.50 6060.43 10

7 6 -572706.20 6019.58 9

-6 13 574321.30 6030.92 4

6 -13 -8 6274.29 548.567 8

6 -13 -8 7356.78 552.276 6

-13 7 -8 6075.87 558.856 2

7 6 -8 6612.89 600.104 9

-6 13 8 5842.18 592.855 10

7 6 -8 7086.45 545.596 8

-13 7 -8 5829.54 558.988 8

7 6 -8 7490.72 652.988 7

7 6 -11 3280.70 402.194 7

7 6 -11 4056.43 324.154 8

-13 7 -11 3039.11 334.346 8

-13 7 -11 3155.67 339.734 2

7 6 -11 4796.07 328.978 14

-13 7 -11 4033.06 330.253 14

6 -13 -11 3516.50 329.346 8

-6 13 11 3730.69 381.526 10

13 -7 11 3219.77 392.33 10

-13 7 -11 3685.34 326.661 11

-13 7 -14 25.3195 44.5918 2

7 6 -14 38.2938 38.4969 2

-13 7 -14 57.2135 36.9282 11

6 -13 -14 19.9042 44.5538 8

-13 7 -14 6.04951 48.2453 14

-7 -6 14-38.7342 72.0136 10

-6 13 14 9.96775 56.5555 10

13 -7 14-13.2221 72.4637 10

7 6 -17 12.5446 24.8256 2

6 -13 -17 36.4677 27.4871 8

-6 -7 -17 152.258 32.642 8

-7 13 -17 98.3352 37.1535 14

-7 13 -17 159.547 36.6819 2

-7 13 -14 574.076 63.3643 11

-7 13 -14 577.217 73.2144 14

6 7 14 420.173 119.334 10

7 -13 14 223.824 93.7804 10

-13 6 14 501.148 108.328 10

-6 -7 -14 574.974 77.4751 8

13 -6 -14 608.979 70.6254 8

-7 13 -11 6334.05 509.133 14

7 -13 11 6005.64 578.51 10

-13 6 11 5087.91 564.677 10

-6 -7 -11 6012.40 504.935 6

-6 -7 -11 6348.17 512.212 8

13 -6 -11 5823.57 502.189 8

-7 13 -11 6048.83 506.333 11

-13 6 8 6602.31 675.025 10

7 -13 8 6128.35 645.791 10

-6 -7 -8 7924.10 580.547 1

-6 -7 -8 7156.93 576.609 6

-7 13 -8 6731.28 907.102 7

-6 -7 -8 6548.57 580.656 8

13 -6 -8 6620.54 572.338 8

-7 13 -520365.40 1691.59 7

7 -13 517300.30 1491.08 10

-13 6 518306.90 1535.08 10

6 7 517840.40 1461.22 9

-13 6 518993.50 1520.74 4

-7 13 -513995.90 1608.74 4

6 7 237189.70 2933.46 9

-7 13 -236131.40 2964.34 7

-13 6 233259.70 2925.99 10

-13 6 239111.40 3023.37 4

-7 13 -241603.30 3334.55 4

7 -13 236426.00 2912.03 10

-7 13 1 1890.85 334.80 4

-6 -7 1 2334.89 251.103 10

6 7 -1 2127.43 261.698 9

-7 13 4 680.756 161.526 4

6 7 -4 657.488 147.429 9

-13 6 -4 636.305 181.699 4

-6 -7 4 460.349 132.732 10

7 -13 -7 2502.44 232.801 8

-7 13 7 2757.47 302.402 4

7 -13 -7 2874.93 237.112 6

-6 -7 7 2950.32 354.546 10

-7 13 7 2656.18 279.20 10

6 7 -7 2584.93 293.346 9

-13 6 -7 2266.64 242.298 8

6 7 -7 2693.60 230.064 8

6 7 -7 2789.96 326.938 7

7 -13 -10 2435.31 235.063 8

6 7 -10 2616.81 327.83 7

-13 6 -10 2135.48 244.083 8

6 7 -10 2685.74 231.464 8

-13 6 -10 2648.38 236.92 11

7 -13 -10 2450.02 235.092 6

6 7 -10 3039.22 232.583 14

-13 6 -10 2716.58 238.378 14

-6 -7 10 2574.30 324.752 10

13 -6 10 2401.58 306.993 10

-7 13 10 2351.79 279.696 10

7 -13 -13 532.277 83.1849 8

13 -6 13 394.779 123.596 10

-7 13 13 400.068 107.077 10

6 7 -13 494.921 78.2322 14

-13 6 -13 667.029 83.2698 14

-13 6 -13 598.635 78.386 11

6 7 -13 968.664 87.1096 2

-13 6 -13 916.104 99.8244 2

6 7 -13 427.617 127.104 7

6 7 -16 9.11032 28.2481 2

7 -13 -16 35.1154 31.647 8

-8 13 -18 78.1311 23.8348 2

13 -5 -18 60.8468 21.0719 2

-8 13 -15 105.051 44.2886 2

-8 13 -15 109.141 38.5298 3

-5 -8 -15 86.3817 43.9976 8

13 -5 -15 58.1894 34.8948 8

-8 13 -15 77.1298 44.1671 14

-8 13 -15 57.3218 27.8232 11

8 -13 12 453.159 121.374 10

-13 5 12 320.613 106.745 10

5 8 12 258.711 125.548 10

-5 -8 -12 317.413 66.4046 6

-8 13 -12 574.187 71.4172 11

-5 -8 -12 461.186 75.0542 8

13 -5 -12 373.25 61.9852 8

-8 13 -12 710.301 78.6799 14

-8 13 -12 352.347 73.7701 2

-8 13 -9 6043.09 536.47 2

-5 -8 -9 6287.87 534.759 8

13 -5 -9 6038.51 524.938 8

13 -5 -9 6134.31 577.70 7

-5 -8 -9 6449.10 533.118 1

-8 13 -9 6380.42 535.559 11

-5 -8 -9 6380.42 530.195 6

-8 13 -9 7343.93 541.201 14

-13 5 9 6792.99 631.377 10

8 -13 9 5755.15 595.624 10

-5 -8 -6 5408.79 482.899 1

-8 13 -6 5011.88 605.781 4

-13 5 3 9112.05 749.12 4

-8 13 -3 7837.78 719.462 7

5 8 3 7398.53 658.409 9

-13 5 3 7462.55 671.127 10

-8 13 -3 7097.35 1008.62 4

8 -13 3 7436.96 650.755 10

-13 5 062645.30 5319.46 4

5 8 062444.40 5235.86 9

-8 13 076710.80 5418.76 4

-13 5 -313820.50 1190.24 4

-5 -8 313063.90 1115.01 10

5 8 -311218.80 1128.25 9

-8 13 314721.50 1172.35 4

8 -13 -626449.10 2230.62 6

-8 13 629197.70 2313.29 4

-5 -8 633636.60 2358.95 10

-8 13 627797.30 2274.74 10

5 8 -628111.50 2309.34 9

8 -13 -627959.70 2229.39 8

8 -13 -9 3084.80 289.086 8

-13 5 -9 3394.57 292.655 11

8 -13 -9 3635.60 293.584 6

-13 5 -9 3134.56 306.327 8

5 8 -9 3435.63 286.573 8

5 8 -9 3271.39 391.649 7

-8 13 9 3092.59 332.573 10

-5 -8 9 3138.49 380.141 10

5 8 -9 2994.19 335.323 9

8 -13 -12-50.9944 54.6812 6

-13 5 -12 -17.306 43.7798 11

-13 5 -12 17.5084 57.0341 2

5 8 -12 86.3169 45.6359 2

5 8 -12 9.45315 48.7524 14

-13 5 -12 152.342 57.2733 14

-13 5 -12-14.6566 59.8792 8

8 -13 -12 41.41 49.3353 8

-8 13 12 33.5499 67.5415 10

-5 -8 12 15.3397 85.5504 10

13 -5 12 31.3896 93.3697 10

5 8 -12-36.2184 87.9657 7

-13 5 -15 286.579 54.8181 2

5 8 -15 235.503 47.9339 2

-13 5 -15 310.799 43.8482 11

-13 5 -15 115.693 50.5801 14

8 -13 -15 242.93 51.7951 8

5 8 -18 51.5867 21.1353 3

8 -13 -18 72.9967 22.2448 8

5 8 -18 90.491 23.5634 2

-9 13 -16 379.894 56.2803 14

-9 13 -16 313.272 52.371 2

13 -4 -16 344.804 48.3764 8

-4 -9 -16 403.991 56.1164 8

-9 13 -13 5399.70 462.258 2

-4 -9 -13 6344.52 468.073 8

13 -4 -13 5682.39 456.639 8

-13 4 13 4314.03 507.857 10

9 -13 13 4050.57 504.56 10

4 9 13 4695.54 523.095 10

-9 13 -13 5283.88 454.204 11

-9 13 -13 5832.02 458.95 14

-9 13 -10 185.80 61.655 2

-4 -9 -10 65.4457 46.0875 6

-9 13 -10 265.217 60.5138 14

13 -4 -10 213.475 77.9866 7

13 -4 -10 138.553 41.3859 8

-4 -9 -10 147.851 52.7756 8

9 -13 10 55.1302 88.6901 10

-13 4 10 147.84 96.8732 10

-9 13 -10 115.211 49.6198 11

4 9 759466.10 5008.77 4

-4 -9 -763299.90 4979.67 8

-4 -9 -765195.20 4974.76 1

-13 4 769209.80 5121.48 10

9 -13 761789.50 5045.56 10

-9 13 -767917.30 5312.99 7

-9 13 -758939.70 4980.42 2

-9 13 -754087.80 5047.31 4

-4 -9 -762640.70 4973.77 6

9 -13 462311.60 5254.95 10

-13 4 471174.70 5294.97 10

-9 13 -466170.90 5335.35 7

-9 13 -459691.90 5470.24 4

4 9 465518.60 5250.93 9

-13 4 1 8302.82 750.471 4

9 -13 1 6972.69 586.287 10

4 9 1 7973.94 648.516 9

-4 -9 2 1153.36 166.713 10

-13 4 -2 1273.44 277.287 4

-9 13 2 1207.87 245.827 4

-9 13 513125.80 1098.87 4

-4 -9 513650.00 1097.60 10

-9 13 513161.40 1049.99 10

4 9 -511295.30 1090.34 9

13 -4 511931.70 1004.22 9

9 -13 -8 10.0624 41.193 8

4 9 -8 59.7067 114.773 7

-13 4 -8 42.4633 64.1195 8

9 -13 -8 90.6121 47.1373 6

-4 -9 8 37.2637 125.06 10

-9 13 8 30.6021 69.3195 10

4 9 -8-13.3049 79.1908 9

4 9 -11 1515.82 130.501 2

9 -13 -11 1085.94 129.109 8

-13 4 -11 1128.01 141.55 8

9 -13 -11 1268.56 133.125 6

-13 4 -11 1189.38 127.48 14

4 9 -11 1367.17 125.624 14

-13 4 -11 1219.04 128.405 11

-4 -9 11 1030.65 190.346 10

-9 13 11 922.94 161.61 10

13 -4 11 1130.89 198.737 10

4 9 -11 1179.68 213.551 7

4 9 -14 1496.52 123.245 2

-13 4 -14 1476.83 132.996 2

13 -4 14 868.158 169.066 10

-4 -9 14 817.416 162.731 10

-13 4 -14 963.346 118.85 14

4 9 -14 1129.58 117.943 14

-13 4 -14 1126.33 115.386 11

9 -13 -14 1178.77 121.636 8

9 -13 -17 142.089 32.8701 8

4 9 -17 173.643 33.7287 2

-10 13 -17-30.5446 35.0648 14

13 -3 -17 45.445 25.8154 2

-10 13 -17 37.0798 27.2275 2

-3 -10 -17 35.4629 27.0668 8

-10 13 -14 4.97075 31.0924 11

-3 -10 -14 129.363 49.4264 8

13 -3 -14 8.0912 35.4156 8

-10 13 -14 48.0195 44.5903 3

-10 13 -14 25.2869 41.2232 2

-10 13 -14 122.386 46.7137 14

3 10 14-61.9051 75.8851 10

10 -13 14 48.5567 72.2498 10

-13 3 14-12.0388 57.1801 10

-10 13 -11 2582.76 244.929 2

-3 -10 -11 2768.40 240.683 8

13 -3 -11 2576.24 229.421 8

13 -3 -11 2193.51 275.78 7

-10 13 -11 2875.64 241.871 14

-3 -10 -11 2671.23 235.468 6

-10 13 -11 2563.65 236.004 11

-13 3 11 2267.33 301.653 10

10 -13 11 2176.08 289.976 10

-3 -10 -8 2529.56 217.44 6

-3 -10 -8 2861.52 220.486 1

10 -13 8 2314.95 280.062 10

-10 13 -8 2323.08 225.665 2

13 -3 -8 2229.35 209.578 8

-3 -10 -8 2311.05 222.219 8

-13 3 8 1899.29 312.328 10

-10 13 -5 7747.24 935.86 4

10 -13 5 9311.87 846.172 10

-10 13 -2 51.374 353.198 4

3 10 2 153.925 80.5342 9

-13 3 2 318.659 94.6726 10

10 -13 2 184.111 49.052 10

-3 -10 1 236.616 88.0166 10

3 10 -1 414.754 134.38 9

-10 13 444308.90 3662.04 4

-3 -10 447309.60 3631.45 10

-10 13 442498.50 3604.63 10

3 10 -444611.70 3677.94 9

-10 13 735919.80 2836.56 10

-3 -10 737500.40 2897.97 10

10 -13 -736321.10 2787.63 6

3 10 -735752.30 2855.92 4

3 10 -733486.20 2872.58 9

10 -13 -732701.80 2783.54 8

-10 13 739554.80 2861.94 4

3 10 -735753.30 2784.13 2

-13 3 -732186.30 2783.06 1

3 10 -738585.50 2924.85 7

10 -13 -10 2234.53 206.188 8

-13 3 -10 1742.16 218.633 8

3 10 -10 2427.29 318.418 7

-13 3 -10 2253.72 206.245 11

10 -13 -10 2162.13 205.351 6

3 10 -10 2252.61 202.803 2

-3 -10 10 2145.70 285.621 10

-10 13 10 2158.07 249.255 10

-13 3 -10 2052.86 203.106 14

3 10 -10 2654.68 204.184 14

-13 3 -13 53.6599 51.7431 8

10 -13 -13 34.6132 46.2431 8

3 10 -13 144.925 46.9652 2

-13 3 -13 67.9544 55.3688 2

-3 -10 13 14.2223 79.4231 10

-10 13 13 63.3184 61.9662 10

13 -3 13 62.6135 91.7897 10

-13 3 -13 52.7948 42.5192 11

3 10 -13 89.1774 102.915 7

-13 3 -13 3.34716 48.3452 14

3 10 -13 36.0375 40.6589 14

10 -13 -16 180.49 40.7249 8

3 10 -16 213.469 41.5758 2

-11 13 -18 186.618 28.7408 2

13 -2 -18 140.958 25.5523 2

-2 -11 -18 149.202 26.1912 8

-11 13 -15 297.766 44.5171 11

13 -2 -15 342.079 47.8179 8

-2 -11 -15 292.603 55.1908 8

-11 13 -15 272.253 54.0139 2

-11 13 -12 1745.69 206.19 3

13 -2 -12 2109.44 181.95 8

-11 13 -12 2111.36 185.34 11

-2 -11 -12 1964.27 185.708 6

-2 -11 -12 2214.91 192.428 8

-13 2 12 1743.63 247.734 10

2 11 12 1759.91 245.652 10

11 -13 12 1582.85 234.277 10

-11 13 -12 1878.17 193.628 2

13 -2 -9 6588.29 618.558 7

13 -2 -9 6489.17 548.761 8

-2 -11 -9 7439.39 563.839 8

-11 13 -9 6371.86 566.99 2

-2 -11 -9 6935.62 557.619 1

-2 -11 -9 6887.11 556.219 6

-13 2 9 6921.25 666.307 10

11 -13 9 5345.75 609.475 10

-11 13 -9 6320.62 560.178 11

11 -13 6 60.9065 89.9549 10

-13 2 6 103.257 151.655 10

2 11 6 59.4941 83.6022 4

-2 -11 -6 94.3583 46.9663 8

-11 13 -6 166.617 44.2477 3

-2 -11 -6 128.049 41.5439 6

-11 13 -6 211.696 48.6474 2

-11 13 -6 162.75 146.411 4

-2 -11 -6 196.735 43.973 1

11 -13 314023.00 1227.62 10

-13 2 315554.80 1271.10 10

2 11 316022.20 1253.64 9

-11 13 -313937.40 1481.21 4

2 11 016000.60 1388.51 9

-11 13 017687.80 1571.68 4

2 11 -364832.10 3934.42 7

-2 -11 365644.30 3831.65 10

2 11 -363781.70 3923.44 9

-11 13 360227.40 3901.11 4

-2 -11 6 2037.67 239.808 10

-11 13 6 1522.53 188.303 10

2 11 -6 1451.87 222.562 4

2 11 -6 1472.99 240.186 9

11 -13 -6 1462.31 118.098 8

2 11 -6 1112.99 114.034 2

-11 13 6 1307.04 199.072 4

-13 2 -6 707.513 108.976 1

11 -13 -6 1296.09 119.248 6

-13 2 -9 267.084 57.4416 11

-2 -11 9 360.51 141.993 10

-11 13 9 184.659 78.2611 10

2 11 -9 343.678 106.294 9

2 11 -9 103.974 45.1953 2

11 -13 -9 158.842 54.8111 8

-13 2 -9 243.791 79.7295 8

11 -13 -9 181.209 55.7467 6

2 11 -9 426.767 57.31 14

2 11 -9 182.969 141.189 7

2 11 -12 4321.12 324.667 2

11 -13 -12 3585.69 323.081 8

-11 13 12 2999.02 359.671 10

-2 -11 12 3115.13 389.217 10

13 -2 12 3649.19 409.629 10

-13 2 -12 3807.44 321.319 11

-13 2 -12 3334.82 319.438 14

2 11 -12 4018.08 320.119 14

-13 2 -12 3750.70 335.799 8

2 11 -12 3638.70 430.671 7

2 11 -15 798.529 88.4023 14

11 -13 -15 824.563 91.214 8

2 11 -15 908.608 91.6044 2

11 -13 -18 186.385 27.0397 8

2 11 -18 160.646 27.2714 3

2 11 -18 199.385 30.4405 2

13 -1 -16 70.0507 29.554 2

-12 13 -16 75.3792 23.7324 11

-1 -12 -16 124.903 36.0266 8

-12 13 -16 43.9758 31.5815 2

-12 13 -13-27.9344 39.3611 11

-12 13 -13-13.2252 47.2165 2

1 12 13-49.7148 77.2142 10

-13 1 13 54.4012 78.4061 10

12 -13 13 37.8949 79.5576 10

-1 -12 -13 12.6981 41.9659 6

-12 13 -13 12.4906 53.7156 3

-1 -12 -13 11.5886 49.1499 8

13 -1 -13 2.71642 33.6348 8

-13 1 10 1301.04 228.387 10

13 -1 -10 1636.78 203.474 9

12 -13 10 1324.74 204.506 10

-12 13 -10 1364.66 151.888 11

-1 -12 -10 1593.60 156.053 8

-12 13 -10 1835.43 171.16 2

-1 -12 -10 1798.72 153.584 6

-12 13 -10 1222.57 166.882 3

13 -1 -10 1481.73 205.699 7

13 -1 -10 1404.89 141.529 8

1 12 7 3535.52 375.594 4

-1 -12 -7 4221.33 338.318 8

-12 13 -7 3759.07 338.927 2

-1 -12 -7 4268.16 331.433 1

-12 13 -7 3727.27 335.296 3

-13 1 7 4451.82 474.748 10

12 -13 7 3589.78 393.332 10

-12 13 -7 2863.66 391.485 4

-1 -12 -7 3829.32 330.847 6

-12 13 -4 1835.47 393.425 4

12 -13 4 2421.96 250.00 10

-13 1 4 2120.41 273.875 10

-13 1 1 9014.47 840.622 10

1 12 110126.70 842.766 9

-12 13 217920.50 1684.06 4

1 12 -221663.80 1711.56 7

-1 -12 218892.90 1595.36 10

13 -1 5 813.331 96.6001 9

1 12 -5 753.366 234.924 9

-12 13 5 1151.87 189.981 4

1 12 -5 999.419 255.524 7

-1 -12 5 1066.08 181.085 10

-12 13 5 613.446 152.024 10

1 12 -5 825.539 205.99 4

-13 1 -8 8601.58 653.982 1

12 -13 -8 7776.01 650.676 8

12 -13 -8 7854.25 650.888 6

1 12 -8 9096.16 821.336 7

-13 1 -8 6158.82 665.623 8

1 12 -8 7756.06 747.919 9

1 12 -8 8456.45 651.954 2

-1 -12 8 7013.84 741.079 10

-12 13 8 7984.32 708.052 10

1 12 -11 10.6734 40.9885 2

-1 -12 11 65.7566 90.1298 10

-12 13 11 22.2932 73.069 10

12 -13 -11 73.2501 49.6996 8

-13 1 -11-29.2845 72.3405 8

-13 1 -11 65.2313 44.5207 11

1 12 -11-45.6096 124.99 7

-13 1 -11 71.1714 47.7146 14

1 12 -11 61.126 39.5361 14

12 -13 -14 82.6218 43.4562 8

-13 1 -14 46.8917 46.8599 8

13 -1 14 72.2951 87.5899 10

-1 -12 14 51.0827 73.9707 10

-13 1 -14 138.26 42.432 11

1 12 -14 79.8513 42.1959 2

-13 1 -14 143.515 51.8389 2

1 12 -14 74.9524 41.5455 14

12 -13 -17 199.844 32.329 8

1 12 -17 130.171 32.8995 2

1 12 -17 160.489 32.6991 3

-13 13 -17 409.211 46.4564 11

13 0 -17 558.481 54.8532 2

-13 13 -17 566.175 57.8972 2

0 -13 -17 565.991 55.9186 8

-13 13 -14 1074.43 124.504 2

13 0 -14 1150.40 114.311 8

-13 13 -14 1214.99 116.693 11

0 -13 -14 1320.13 125.49 8

-13 0 11-50.0704 96.2434 10

13 -13 11-39.4539 81.7319 10

0 13 11 13.4623 83.6633 10

0 -13 -11 61.0126 46.1663 6

0 -13 -11 25.3815 52.6056 8

13 0 -11 33.6708 37.7186 8

-13 13 -11 54.6927 69.6266 3

13 0 -11-13.4183 69.5023 7

-13 13 -11 55.2722 46.6586 11

-13 13 -11 5.80717 61.0625 2

-13 13 -8 651.503 92.5489 11

-13 0 8 622.532 191.728 10

13 -13 8 668.792 140.951 10

13 0 -8 648.074 131.008 9

0 -13 -8 761.009 92.0016 8

13 0 -8 651.201 77.4989 8

-13 13 -8 765.586 92.616 14

-13 13 -8 491.306 92.5363 2

0 -13 -8 786.452 85.3957 1

0 -13 -8 729.614 85.4418 6

-13 13 -8 534.998 86.0925 3

-13 0 5 477.714 164.15 10

13 -13 5 368.322 105.756 10

0 13 5 315.205 115.64 4

0 -13 -5 321.043 53.1225 6

-13 13 -2 453.333 338.46 4

0 13 2 730.518 135.008 9

0 13 2 561.299 88.0809 7

13 -13 2 411.606 67.2677 10

-13 0 2 799.166 145.28 10

-13 13 118997.70 1813.81 4

0 -13 120044.10 1696.12 10

0 13 -123194.60 1792.30 7

0 13 -413777.70 1081.78 9

13 0 412639.00 898.50 9

0 13 -415150.10 1142.82 7

0 -13 413404.30 950.82 10

-13 13 4 8057.58 882.739 10

-13 13 411238.10 981.259 4

0 13 -4 8802.76 1018.89 4

13 -13 -7 658.716 76.4888 6

-13 13 7 425.923 125.615 10

0 -13 7 387.019 145.165 10

0 13 -7 428.884 181.632 9

13 -13 -7 445.19 70.7569 8

-13 13 7 525.896 130.159 4

0 13 -7 415.924 143.611 4

0 13 -7 564.487 72.5899 2

-13 0 -7 697.98 76.4209 1

13 -13 -10 7996.33 606.205 8

-13 0 -10 6695.93 622.697 8

0 13 -10 8041.20 760.961 7

-13 0 -10 8248.63 608.447 11

0 13 -10 6838.48 598.395 11

13 -13 -10 7960.74 605.652 6

-13 0 -10 5615.55 596.457 14

0 13 -10 8024.99 603.445 14

0 13 -10 7931.11 606.061 2

0 -13 10 6676.93 689.402 10

-13 13 10 6206.77 647.984 10

13 0 13 1513.91 241.123 10

0 -13 13 1240.05 216.883 10

0 13 -13 2005.75 170.93 14

-13 0 -13 1462.33 170.156 14

-13 0 -13 1719.45 185.856 2

0 13 -13 1937.86 173.845 2

-13 0 -13 2210.20 175.451 11

13 -13 -13 1948.56 173.925 8

-13 0 -13 1947.50 185.974 8

0 13 -13 1889.27 269.377 7

13 -13 -16 177.713 35.5376 8

0 13 -16 110.705 33.9061 2

14 -13 -18 98.0825 19.2616 8

-1 14 -18 102.245 23.6305 2

14 -13 -15 49.8217 33.703 8

-1 14 -15-11.2522 34.018 14

-13 -1 -12 2374.78 298.034 14

-1 14 -12 4048.58 302.874 14

14 -13 -12 3676.40 305.151 8

-1 14 -12 3615.52 306.026 2

1 -14 12 2776.15 366.54 10

-13 -1 -12 4059.63 307.067 11

-13 -1 -12 4040.03 324.913 8

-1 14 -12 3562.96 429.802 7

-13 -1 -9 88.0483 47.8916 11

-1 14 -9 160.191 45.0776 11

1 -14 9-17.8781 108.381 10

-14 13 9 64.9734 77.9088 10

14 -13 -9 33.3258 44.1928 6

14 -13 -9 92.9591 46.5511 8

-13 -1 -9 113.22 77.4648 8

-13 -1 -9 171.875 55.9435 1

-1 14 -9 262.60 181.533 7

-1 14 -9 307.039 53.3134 2

-1 14 -9 110.611 48.7334 14

1 -14 6 88.1383 111.798 10

-14 13 6-16.2239 92.042 10

-1 14 -6-30.4197 160.625 9

14 -13 -6 59.1175 38.2265 8

-1 14 -6 32.442 34.9022 2

-1 14 -6 35.3539 237.249 7

-1 14 -6-47.4324 175.385 4

-14 13 6-71.6932 115.981 4

14 -13 -6 51.4582 39.121 6

-13 -1 -6 13.2476 35.3226 1

1 -14 325584.60 1987.79 10

-1 14 -327999.90 2164.86 7

13 1 323511.20 1965.93 9

-1 14 -325375.60 2089.69 9

-14 13 326927.20 2067.71 4

-1 14 0 4076.06 433.688 9

-1 14 0 4047.28 425.101 7

-14 13 -323131.00 1989.66 4

14 -13 321987.40 1776.35 10

-13 -1 326420.70 1837.15 10

-1 14 320391.50 1737.47 7

-1 14 325007.10 1849.26 4

14 -13 6 5412.97 562.691 10

1 -14 -6 6544.09 508.181 1

-13 -1 6 7769.55 665.348 10

-1 14 6 5769.71 570.932 4

1 -14 -6 5913.63 510.95 8

-14 13 -6 6149.50 511.821 2

-14 13 -6 5214.08 608.55 4

-14 13 -6 6179.83 509.077 3

-14 13 -9 163.408 57.2295 11

-14 13 -9 74.0389 56.4083 14

-13 -1 9 168.081 146.105 10

14 -13 9 51.7263 86.0284 10

13 1 -9 11.9883 93.1605 9

1 -14 -9 64.8579 52.9005 8

-14 13 -9 154.965 68.9007 2

13 1 -9 73.4576 88.9829 7

13 1 -9 27.2186 44.8559 8

1 -14 -9 117.321 55.6694 6

-14 13 -9 170.227 60.8696 3

-14 13 -12-7.27748 40.7771 11

-1 14 12-12.1852 80.7999 10

-13 -1 12-30.7326 82.6862 10

14 -13 12-25.5547 74.8532 10

1 -14 -12 20.9101 44.3635 6

13 1 -12 18.8804 36.3324 8

1 -14 -12 48.3839 52.1625 8

13 1 -12 -12.92 72.0547 7

-14 13 -12 87.4064 50.4715 14

-14 13 -12 67.9298 57.4464 2

-14 13 -12 8.11026 65.0625 3

1 -14 -15-14.0409 36.6658 8

-14 13 -15 12.5752 27.7083 11

-14 13 -15 -52.635 42.5451 2

13 1 -15 32.2401 29.01 2

-14 13 -15 35.9737 40.7294 14

1 -14 -18 151.183 24.2847 8

-2 14 -16 25.6241 29.0548 3

-2 14 -16 25.8616 31.7496 2

-2 14 -16 1.9447 32.6023 14

14 -12 -16-10.2143 27.346 8

-12 -2 -13 1753.44 163.176 8

-2 14 -13 1372.14 145.245 14

14 -12 -13 1501.52 150.014 8

-2 14 -13 2026.24 158.138 2

2 -14 13 1173.23 196.968 10

12 2 13 1261.30 218.756 10

-2 14 -10 4999.34 360.439 2

14 -12 -10 4336.32 354.418 8

-2 14 -10 4792.90 534.99 7

-12 -2 -10 4576.13 376.00 8

-2 14 -10 4174.81 353.801 14

-12 -2 -10 2853.52 345.487 14

-2 14 -10 4391.94 352.364 11

-12 -2 -10 4649.87 358.503 11

-14 12 10 3770.01 407.222 10

2 -14 10 3572.73 430.816 10

-2 14 -7 8476.45 1095.18 9

14 -12 -711444.70 959.152 8

-12 -2 -712457.00 960.593 1

14 -12 -711053.80 958.691 6

-2 14 -712885.20 1084.41 4

-2 14 -712003.50 962.471 2

-14 12 713614.60 1039.82 4

2 -14 713270.80 1063.26 10

-14 12 711357.10 1051.56 10

2 -14 4 9264.51 668.56 10

-2 14 -4 9216.25 890.246 7

-14 12 4 8855.09 711.929 4

-2 14 -4 8466.92 797.827 9

12 2 4 7872.44 618.052 9

-2 14 -4 7007.79 841.059 4

-14 12 118289.50 1492.33 4

2 -14 115899.90 1342.76 10

12 2 115293.30 1355.31 9

-2 14 -117180.10 1409.51 7

-14 12 -213011.50 1281.86 4

-12 -2 214999.10 1138.48 10

-2 14 214488.10 1178.29 4

-2 14 212148.20 1065.78 7

14 -12 5 2196.86 251.047 10

-12 -2 5 2795.70 310.51 10

-2 14 5 2133.65 262.667 4

2 -14 -5 2162.32 190.647 8

-12 -2 8 176.80 141.333 10

14 -12 8 152.526 96.2139 10

12 2 -8 83.9665 88.262 9

2 -14 -8 152.767 54.722 8

-14 12 -8 33.3044 51.9438 2

-14 12 -8 111.536 55.1456 11

12 2 -8 129.024 41.2923 8

2 -14 -8 120.654 46.7415 1

-14 12 -8 98.2696 44.0183 3

12 2 -11 29.2172 67.6529 7

-2 14 11 12.5222 83.0271 10

14 -12 11 26.2813 75.762 10

-12 -2 11-34.0037 104.152 10

2 -14 -11 3.66392 48.5927 6

2 -14 -11 8.29205 51.8801 8

12 2 -11-11.3482 40.715 8

-14 12 -11-3.93894 48.0942 11

-14 12 -11 56.5597 51.9196 14

-14 12 -11 45.0355 66.8624 3

-14 12 -11 43.9085 63.974 2

-14 12 -14 357.504 59.2303 11

-14 12 -14 527.264 71.9441 14

-14 12 -14 546.393 74.3029 2

2 -14 -14 389.372 65.9591 8

-14 12 -17 130.897 20.8194 11

2 -14 -17 176.854 30.9575 8

12 2 -17 184.771 29.9162 2

14 -11 -17 909.595 86.376 8

-3 14 -17 929.82 88.2642 3

-3 14 -17 947.797 90.6245 2

-11 -3 -14 5848.68 445.24 8

-3 14 -14 5277.20 438.401 2

-3 14 -14 5331.80 433.484 14

14 -11 -14 5171.13 435.443 8

-3 14 -11 979.78 87.4485 14

-11 -3 -11 333.329 74.6279 14

-14 11 11 507.305 116.103 10

3 -14 11 564.026 146.73 10

-11 -3 -11 582.721 92.5638 8

14 -11 -11 575.138 79.7766 8

-11 -3 -11 682.192 81.3242 11

-3 14 -11 622.929 75.837 11

-3 14 -11 910.338 211.527 7

-3 14 -11 630.015 82.3958 2

14 -11 -8 3837.85 342.422 8

-11 -3 -8 3579.00 358.603 8

3 -14 8 3622.26 428.589 10

-14 11 8 4219.35 434.293 10

-3 14 -8 5203.88 627.40 7

-11 -3 -8 3836.04 338.947 6

14 -11 -8 4281.51 344.185 6

-3 14 -8 4000.75 346.387 2

-11 -3 -8 4474.83 347.622 1

11 3 516810.50 1410.58 9

-3 14 -517993.70 1616.04 9

-3 14 -517933.20 1397.09 2

-14 11 519969.70 1494.71 4

-3 14 -519141.40 1650.57 4

-11 -3 -515983.10 1392.15 1

-14 11 517495.50 1471.37 10

3 -14 518860.50 1468.43 10

-14 11 2 1222.89 244.11 4

-3 14 -2 799.165 370.222 4

3 -14 2 465.371 100.638 10

11 3 2 456.083 104.441 9

-3 14 -2 844.114 181.324 9

-3 14 -2 634.792 186.637 7

-3 14 112055.30 994.413 7

-3 14 114176.70 1125.17 4

11 3 -110716.60 1025.56 9

-11 -3 4 429.043 135.668 10

-14 11 -4 368.369 195.271 4

-3 14 4 520.445 152.066 4

14 -11 7 9959.85 898.277 10

-11 -3 713465.20 1002.85 10

11 3 -712037.80 912.898 9

-14 11 -710048.10 840.794 2

3 -14 -711028.80 840.259 8

3 -14 -710434.00 836.121 1

-14 11 -7 9280.72 832.713 3

-14 11 -7 8689.06 901.597 4

-3 14 711099.50 907.753 4

-14 11 -10 7991.22 835.124 3

11 3 -1010508.80 879.549 9

3 -14 -1011203.00 834.902 8

11 3 -1011234.90 824.769 8

11 3 -1010082.70 907.029 7

-14 11 -1010237.20 848.325 2

14 -11 10 9055.79 884.985 10

-11 -3 10 9068.79 920.423 10

-3 14 10 8278.72 874.625 10

3 -14 -1011179.20 832.042 6

-14 11 -10 9556.04 831.776 14

-14 11 -10 9854.41 830.578 11

3 -14 -13 1474.29 143.642 8

-14 11 -13 1516.75 138.393 11

3 -14 -13 1573.83 139.524 6

14 -11 13 1123.65 184.376 10

-11 -3 13 980.535 183.308 10

-3 14 13 1080.32 173.992 10

-14 11 -13 1391.13 150.739 2

-14 11 -13 1329.63 139.338 14

-14 11 -16 63.3979 34.3666 2

3 -14 -16 102.50 34.9492 8

-14 11 -16 79.7823 37.5565 14

11 3 -16 133.939 32.5758 2

-14 11 -16 95.7508 25.4131 11

-4 14 -18 109.09 26.2445 2

-4 14 -15 14.5167 37.5949 2

-10 -4 -15 31.171 41.3759 8

14 -10 -15 2.55114 34.8953 8

-4 14 -15-28.3961 40.0811 14

-4 14 -15 111.352 37.4552 3

-4 14 -12 479.013 67.9626 2

14 -10 -12 373.627 63.1403 8

-10 -4 -12 416.669 70.9668 8

-4 14 -12 272.96 53.5877 11

-4 14 -12 331.964 60.2798 14

4 -14 12 379.345 119.333 10

-14 10 12 244.368 88.4171 10

-4 14 -9 3066.28 480.146 7

-4 14 -9 2771.70 253.228 14

4 -14 9 2436.33 327.808 10

-14 10 9 2741.23 320.285 10

-10 -4 -9 2843.42 247.277 6

-4 14 -9 2996.96 254.727 2

-4 14 -9 2802.67 251.186 11

-10 -4 -9 2880.17 252.763 11

-10 -4 -9 2849.20 262.543 8

14 -10 -9 2699.76 248.795 8

-4 14 -6 1799.69 432.763 9

-4 14 -6 3221.19 263.656 2

-10 -4 -6 3143.64 259.901 1

-14 10 6 2756.47 359.532 10

4 -14 6 3502.81 343.547 10

-14 10 6 3400.97 338.248 4

-4 14 -6 2926.47 411.876 4

-4 14 -6 4026.44 632.15 7

14 -10 -6 2630.96 258.206 6

-4 14 -3 3991.22 729.541 4

-14 10 3 5540.00 465.128 4

-4 14 -3 5213.60 520.104 7

-14 10 3 2939.29 333.952 10

10 4 3 4218.92 358.172 9

-4 14 -3 4150.62 450.579 9

4 -14 3 4101.32 363.295 10

-4 14 0 112.892 207.935 4

-14 10 0 317.408 198.853 4

-4 14 0 238.296 75.252 7

10 4 0 437.822 105.215 9

-14 10 -3 883.925 250.211 4

-4 14 3 744.544 167.806 4

-4 14 6 9639.30 872.115 4

4 -14 -6 9673.04 800.268 8

4 -14 -610020.20 800.872 6

10 4 -610490.20 870.768 9

-14 10 -6 8737.95 884.091 4

-14 10 -9 324.581 65.0051 3

10 4 -9 546.413 113.555 9

-14 10 -9 627.562 79.8163 11

10 4 -9 486.837 64.3698 8

-14 10 -9 369.562 77.8973 14

-14 10 -9 526.744 90.8711 2

10 4 -9 450.627 135.867 7

14 -10 9 253.445 109.206 10

-10 -4 9 462.287 167.62 10

14 -10 12 1166.09 189.258 10

-10 -4 12 1148.35 194.859 10

-14 10 -12 1412.64 155.194 2

10 4 -12 1336.05 129.262 8

-14 10 -12 1369.63 136.838 14

-14 10 -12 1368.85 135.807 11

4 -14 -12 1489.05 140.54 6

-4 14 12 1248.67 181.312 10

10 4 -12 1070.09 182.76 7

-14 10 -15 175.782 49.63 14

-14 10 -15 171.715 36.3076 11

10 4 -15 258.347 43.432 2

-14 10 -15 124.238 45.2826 2

4 -14 -18 143.594 26.1428 8

10 4 -18 201.743 27.2703 2

-5 14 -16-39.6337 37.1615 14

-9 -5 -16 19.5039 30.3991 8

14 -9 -16 6.21059 27.2004 8

-5 14 -16 23.9131 29.9198 3

-5 14 -13 229.827 56.0913 2

-5 14 -13 275.045 45.5061 11

14 -9 -13 200.519 51.0783 8

-9 -5 -13 268.274 61.3869 8

-5 14 -13 343.326 54.2489 14

9 5 13 66.2938 98.9488 10

5 -14 13 67.6181 80.2852 10

-14 9 13 247.318 87.4852 10

-5 14 -10 1674.17 156.455 2

14 -9 -10 1641.03 149.04 8

-9 -5 -10 1699.35 161.859 8

-5 14 -10 1549.82 151.603 11

-9 -5 -10 1740.47 153.895 11

-9 -5 -10 1077.93 146.586 14

-5 14 -10 1654.87 155.48 14

-5 14 -10 1598.53 335.596 7

-14 9 10 1306.48 206.541 10

5 -14 10 1476.21 222.295 10

-9 -5 -10 1543.30 147.758 6

-5 14 -713420.00 1118.49 2

5 -14 715432.50 1208.53 10

-14 9 714929.60 1240.45 10

-5 14 -713101.50 1228.82 4

-9 -5 -713979.00 1113.33 6

14 -9 -713166.30 1112.31 8

-9 -5 -714190.50 1126.73 8

-14 9 4 5753.00 543.139 4

-5 14 -4 5992.98 854.609 4

-14 9 4 4491.66 480.831 10

-5 14 -4 6634.81 691.397 7

-5 14 -4 5828.07 610.755 9

9 5 4 5157.19 465.757 9

5 -14 4 5832.89 495.393 10

9 5 116504.60 1426.70 9

-5 14 -116428.40 1436.46 7

-14 9 118309.10 1528.50 4

5 -14 117488.70 1400.36 10

-14 9 -225427.00 2260.36 4

9 5 -225072.30 2164.46 9

-5 14 228405.00 2218.88 4

5 -14 -514222.90 1121.94 6

-14 9 -512683.20 1222.73 4

9 5 -512943.60 1185.55 9

-5 14 513831.50 1206.20 4

5 -14 -8 7491.29 635.277 6

9 5 -8 8294.73 693.002 9

-14 9 -8 6881.41 640.915 2

9 5 -8 8020.10 630.623 8

9 5 -8 8544.72 729.607 7

-14 9 -11 22.8519 43.5038 11

-14 9 -11 57.949 50.388 14

9 5 -11 45.9121 40.5956 14

-14 9 -11-19.5889 67.6898 2

9 5 -11 16.5663 80.5226 7

9 5 -11 48.0227 40.2953 8

14 -9 11 14.1086 80.081 10

-5 14 11 129.537 80.0246 10

-9 -5 11 118.441 110.852 10

-14 9 -14 159.886 40.7852 11

-14 9 -14 129.482 55.3861 2

9 5 -14 143.041 44.7665 2

-9 -5 14 63.5244 71.7659 10

14 -9 14 74.6824 74.9208 10

-14 9 -14 213.256 53.8599 14

9 5 -17 480.609 53.8871 2

14 -8 -17 457.606 50.0777 8

-6 14 -14 487.512 63.1781 14

-6 14 -14 345.225 50.6628 11

-8 -6 -14 495.869 71.6634 8

14 -8 -14 384.732 58.6017 8

-14 8 14 261.117 88.3886 10

6 -14 14 397.217 106.615 10

8 6 14 289.028 108.199 10

-6 14 -11 5239.52 432.10 11

-8 -6 -11 5324.02 431.18 6

-6 14 -11 5314.72 433.619 14

-8 -6 -11 5513.71 439.765 8

14 -8 -11 4968.01 428.888 8

6 -14 11 3611.40 484.021 10

-14 8 11 4166.95 486.377 10

-8 -6 -8 1336.08 129.269 6

-6 14 -8 1259.86 134.24 2

6 -14 8 1312.72 205.719 10

-14 8 8 1616.51 234.72 10

-8 -6 -8 1255.05 137.581 8

14 -8 -8 1237.42 124.579 8

-6 14 -516277.50 1467.18 2

8 6 519161.30 1492.78 9

-14 8 520271.40 1571.06 10

6 -14 521369.60 1538.32 10

-14 8 520125.50 1556.78 4

-6 14 -517084.20 1711.20 4

-14 8 271390.10 6556.40 10

6 -14 284855.50 6556.25 10

-14 8 292080.80 6665.95 4

8 6 283517.90 6574.54 9

-6 14 -286682.00 6619.15 7

-6 14 -2105151.0 7078.81 4

-8 -6 1 1289.16 183.384 10

8 6 -1 1203.79 184.962 9

-6 14 1 1113.01 254.554 4

-8 -6 429489.80 2244.63 10

-6 14 427591.00 2248.93 4

8 6 -425547.00 2226.29 9

-14 8 -424614.50 2267.41 4

-6 14 738503.30 3071.36 4

-14 8 -734019.50 2995.16 2

6 -14 -737851.60 2995.89 6

8 6 -738906.80 2990.92 8

8 6 -739840.80 3059.87 9

8 6 -10 8331.26 773.083 7

8 6 -10 8258.85 673.484 8

-14 8 -10 8272.00 679.567 11

-14 8 -10 8488.85 682.589 14

8 6 -10 8918.44 673.166 14

-6 14 10 7061.66 721.054 10

14 -8 10 7117.93 740.407 10

-14 8 -10 7033.54 693.101 2

8 6 -10 8201.20 723.871 9

8 6 -13 596.904 140.767 7

8 6 -13 977.81 92.3897 2

-14 8 -13 792.653 105.003 2

-8 -6 13 721.542 145.526 10

-6 14 13 431.807 110.195 10

14 -8 13 674.42 139.365 10

-14 8 -13 869.947 95.2453 14

-14 8 -13 716.711 88.5058 11

6 -14 -13 705.87 92.1374 8

-14 8 -16 137.484 28.4163 11

6 -14 -16 88.7929 34.3153 8

-14 8 -16 15.5125 38.8384 14

8 6 -16 119.816 33.5489 2

14 -7 -18-5.72723 17.6715 2

-7 14 -18 7.37176 20.8199 2

-7 14 -15 29.6134 39.3894 14

-7 14 -15 54.0334 24.7638 11

-7 -7 -15 50.125 38.468 8

14 -7 -15 72.4879 34.3908 8

-7 14 -15 46.2033 37.2881 3

14 -7 -12 1237.53 124.707 8

-7 -7 -12 1520.07 139.122 8

-14 7 12 965.147 174.487 10

7 -14 12 865.859 171.768 10

-7 14 -12 1425.73 130.831 14

-7 -7 -12 1154.21 124.818 6

-7 14 -12 1169.08 124.469 11

14 -7 -9 9436.22 847.043 8

-7 -7 -910697.60 859.351 8

14 -7 -9 9607.82 889.999 7

-7 14 -913004.20 1231.44 7

-7 -7 -910267.40 855.04 1

-7 14 -912410.40 863.064 14

-7 -7 -910208.10 851.584 6

-14 7 910020.00 941.362 10

7 -14 9 9135.78 919.65 10

-7 14 -911099.20 859.493 11

-7 14 -6 1839.20 347.041 4

-7 -7 -6 2957.04 226.133 1

-14 7 6 2484.98 350.166 10

7 -14 6 1519.83 270.603 10

7 -14 311129.40 936.933 10

-14 7 311847.00 1019.07 4

7 7 311602.70 943.218 9

-7 14 -310644.90 1398.06 4

-7 14 -312370.10 1026.07 7

-14 7 310080.80 943.24 10

7 7 0 9358.67 825.73 9

-7 14 010111.90 810.318 7

-7 14 011781.60 1007.88 4

-7 14 344867.40 3567.83 4

-14 7 -338348.80 3582.08 4

-7 -7 345114.90 3531.00 10

-7 -7 6 7849.40 673.782 10

7 -14 -6 6395.93 547.331 6

-7 14 6 7739.76 647.856 4

7 7 -6 7214.70 621.036 9

7 -14 -6 6400.89 545.427 8

-14 7 -9 9737.30 965.449 2

7 7 -912295.50 1010.09 9

7 -14 -911858.30 956.434 8

-14 7 -911490.00 957.761 11

7 -14 -911502.80 956.34 6

-14 7 -911176.10 971.202 8

7 7 -912303.30 951.908 8

7 7 -912879.20 1062.67 7

-14 7 -912675.60 963.797 14

-7 14 911458.40 1003.79 10

7 -14 -12 1133.47 122.726 8

-7 14 12 1157.85 165.655 10

-14 7 -12 1056.97 118.15 11

-14 7 -12 765.387 130.962 2

7 7 -12 867.245 176.967 7

14 -7 12 1081.91 182.18 10

-14 7 -12 1206.74 121.41 14

7 7 -12 1338.43 120.573 14

-14 7 -15 36.9706 28.6921 11

7 7 -15 33.4157 31.8554 2

-14 7 -15 3.54642 37.472 2

-14 7 -15 -31.854 46.0115 14

7 -14 -15 22.2439 36.5551 8

7 -14 -18 184.515 29.0652 8

7 7 -18 227.22 30.0854 2

-8 14 -16 1092.23 115.226 14

-8 14 -16 1214.93 117.15 2

-6 -8 -16 1268.93 117.948 8

14 -6 -16 1257.81 112.785 8

-8 14 -13 22.9279 49.205 2

6 8 13 244.545 103.90 10

-14 6 13 12.6922 65.9057 10

8 -14 13 131.181 87.1213 10

-6 -8 -13 78.2832 50.5508 8

14 -6 -13 47.2536 40.8857 8

-8 14 -13 109.549 48.6626 14

-8 14 -13 53.5372 36.4446 11

-14 6 10 289.654 113.146 10

8 -14 10 189.152 103.681 10

14 -6 -10 247.611 48.5092 8

-6 -8 -10 242.567 60.2102 8

14 -6 -10 99.8704 61.0811 7

-6 -8 -10 274.915 56.3402 6

-8 14 -10 243.442 62.2428 14

-8 14 -10 192.951 52.3083 11

-6 -8 -716284.60 1300.31 6

-6 -8 -716685.80 1302.35 1

14 -6 -714977.30 1296.64 8

-6 -8 -717457.60 1308.60 8

-8 14 -714601.10 1395.05 4

-14 6 718024.80 1448.56 10

8 -14 714619.30 1369.89 10

8 -14 4 3398.19 352.916 10

-14 6 4 3648.51 383.593 10

-14 6 4 5266.24 446.149 4

-8 14 -4 4361.03 681.59 4

-8 14 -4 3814.38 451.809 7

6 8 4 3574.73 347.71 9

8 -14 137618.60 3102.99 10

-14 6 141869.30 3250.82 4

6 8 140991.60 3152.99 9

-8 14 -138639.40 3152.30 7

-6 -8 2 9526.91 833.381 10

-8 14 2 9583.74 899.663 4

-14 6 -2 9193.57 920.022 4

-6 -8 5 7310.94 618.812 10

6 8 -5 6342.93 595.49 9

8 -14 -5 5932.33 507.226 8

-8 14 5 6796.09 620.998 4

8 -14 -8 6456.45 540.902 8

-14 6 -8 6350.61 557.094 8

6 8 -8 7365.09 651.82 7

8 -14 -8 6699.58 542.903 6

-8 14 8 6382.10 586.486 10

-6 -8 8 6167.27 646.077 10

-14 6 -8 6025.11 547.426 2

6 8 -8 7233.69 609.078 9

-14 6 -11 810.658 127.996 2

8 -14 -11 995.853 107.889 8

-14 6 -11 715.51 113.579 8

8 -14 -11 1003.32 107.87 6

-14 6 -11 836.281 103.309 14

6 8 -11 1045.20 100.87 14

-14 6 -11 956.237 102.704 11

14 -6 11 812.052 161.61 10

-8 14 11 693.054 133.359 10

-6 -8 11 486.692 157.70 10

6 8 -11 739.402 171.71 7

-14 6 -14 636.616 88.5748 2

6 8 -14 700.333 77.5126 2

14 -6 14 477.334 120.661 10

-14 6 -14 591.021 78.717 14

8 -14 -14 522.892 76.5545 8

-14 6 -14 614.925 73.2891 11

8 -14 -17-12.1074 24.782 8

6 8 -17-19.0642 25.0255 2

-5 -9 -17 21.4376 23.5467 8

-9 14 -17-32.4463 29.173 2

-9 14 -14 87.6182 46.7695 2

-9 14 -14 154.346 47.2749 14

-5 -9 -14 106.627 46.2652 8

14 -5 -14 8.13246 35.8921 8

-9 14 -14 101.522 45.3972 3

-14 5 14 184.722 78.5653 10

5 9 14 13.0809 69.263 10

9 -14 14 12.0318 64.9477 10

-9 14 -14 36.1121 29.6478 11

-9 14 -11 3103.83 284.452 2

-5 -9 -11 3512.32 285.894 8

14 -5 -11 3104.06 272.47 8

-9 14 -11 2970.65 278.208 11

-9 14 -11 3015.25 280.565 14

-5 -9 -11 3625.16 280.627 6

9 -14 11 2568.04 336.445 10

-14 5 11 2525.62 338.90 10

-5 -9 -8 579.53 79.7752 8

-9 14 -8 174.027 285.015 7

14 -5 -8 350.708 61.6581 8

-5 -9 -8 920.679 82.2014 1

-9 14 -8 633.338 85.0287 2

-5 -9 -8 598.31 74.899 6

9 -14 8 421.187 130.686 10

-14 5 8 436.325 165.285 10

9 -14 5 93.94 81.2322 10

-14 5 5 67.1619 130.322 10

-9 14 -5 201.42 186.217 7

-9 14 -5 35.2674 228.174 4

9 -14 262420.60 5199.54 10

-14 5 274957.30 5342.60 4

-9 14 -264939.70 5257.42 7

-9 14 -273945.30 5669.80 4

-14 5 263795.40 5236.05 10

5 9 267178.20 5235.66 9

-9 14 124652.20 2145.93 4

-5 -9 125358.30 2037.00 10

5 9 -123672.60 2053.36 9

-9 14 442654.10 3804.63 4

5 9 -443855.40 3798.53 9

-9 14 447230.10 3740.31 10

-5 -9 449760.50 3787.52 10

-9 14 7 256.753 84.4198 10

-5 -9 7 395.374 144.772 10

5 9 -7 219.724 104.647 9

9 -14 -7 269.604 51.1981 8

-9 14 7 119.373 104.878 4

9 -14 -7 268.812 58.5197 6

5 9 -7 585.731 148.288 7

-14 5 -7 234.407 64.6085 8

-14 5 -10 2017.55 277.972 2

9 -14 -10 2822.15 267.092 8

5 9 -10 3188.26 375.206 7

-14 5 -10 2418.95 281.01 8

-14 5 -10 3165.78 269.27 11

9 -14 -10 2895.86 267.805 6

-9 14 10 2993.63 313.56 10

-5 -9 10 2623.05 347.235 10

5 9 -10 3933.71 266.42 14

-14 5 -10 3273.49 269.75 14

5 9 -13 582.727 73.4073 14

-14 5 -13 514.524 73.6204 14

-9 14 13 378.799 96.496 10

14 -5 13 432.643 131.075 10

-5 -9 13 227.403 108.853 10

-14 5 -13 537.438 69.7375 11

9 -14 -13 456.17 75.4999 8

5 9 -13 582.423 72.7827 2

-14 5 -13 640.611 90.9676 2

5 9 -13 524.258 132.721 7

9 -14 -16 537.899 63.2829 8

5 9 -16 525.064 62.6248 2

14 -4 -18 205.183 28.7835 2

-10 14 -18 243.021 32.5244 2

14 -4 -15 34.4032 30.2753 8

-4 -10 -15 9.15521 38.4234 8

-10 14 -15 36.3928 27.0618 11

-10 14 -15 65.5997 36.538 3

-10 14 -15 6.14633 36.8141 2

-10 14 -15 82.9913 45.0474 14

-10 14 -12 242.554 53.5265 11

10 -14 12 282.682 96.7546 10

4 10 12 198.151 101.924 10

-14 4 12 86.196 82.7155 10

-4 -10 -12 277.773 57.1929 6

-10 14 -12 319.599 66.7488 14

-10 14 -12 231.795 63.9478 2

-10 14 -12 276.598 80.5214 3

-4 -10 -12 347.282 67.1234 8

14 -4 -12 255.857 52.5766 8

10 -14 9 138.803 105.359 10

-14 4 9 153.701 121.637 10

14 -4 -9 137.00 38.4399 8

-4 -10 -9 178.758 56.5385 8

-10 14 -9 159.903 58.1401 14

-10 14 -9 186.182 62.5345 2

-10 14 -9 173.568 56.041 11

-4 -10 -9 134.882 49.8982 1

-4 -10 -9 157.184 48.9422 6

-10 14 -6 60.1369 45.1357 3

-10 14 -6 80.4655 45.1578 2

-4 -10 -6 78.8757 39.8947 1

-14 4 6 84.3347 154.531 10

10 -14 6 377.622 115.819 10

-4 -10 -6 97.2496 40.121 6

-10 14 -6 94.0393 135.722 4

4 10 336441.30 2892.23 9

-14 4 335177.70 2906.21 10

-10 14 -340463.00 3292.13 4

10 -14 334775.90 2870.87 10

4 10 0 7632.64 742.193 9

-10 14 0 9836.30 956.859 4

-14 4 0 9291.54 869.484 4

-10 14 3 245.754 165.021 4

-10 14 3 173.266 58.6048 10

-4 -10 3 288.842 106.172 10

4 10 -3 180.949 128.064 9

4 10 -6 596.046 153.015 9

10 -14 -6 623.504 77.6328 8

4 10 -6 695.406 77.0481 2

-10 14 6 743.589 168.67 4

10 -14 -6 686.765 84.3763 6

-10 14 6 712.807 123.043 10

-4 -10 6 637.997 166.199 10

-14 4 -9 220.223 51.5064 14

4 10 -9 234.951 86.227 9

10 -14 -9 117.626 50.3941 8

-14 4 -9 71.8706 46.8692 11

-10 14 9 82.7094 64.3385 10

-4 -10 9-18.8228 132.671 10

-14 4 -9 215.359 79.0379 8

4 10 -9 110.701 46.2631 2

4 10 -9 61.9049 126.911 7

10 -14 -9 251.042 53.4169 6

4 10 -12 160.493 48.2409 2

-14 4 -12 116.936 63.3063 2

4 10 -12 19.3111 102.083 7

-10 14 12 31.765 65.9772 10

14 -4 12 16.1541 93.2677 10

-4 -10 12 217.257 100.475 10

-14 4 -12 67.6725 51.025 14

4 10 -12 48.8055 44.5095 14

10 -14 -12 95.575 54.0675 8

-14 4 -12-69.6186 67.8765 8

-14 4 -12 34.6081 44.1817 11

-14 4 -15 135.997 34.4189 11

10 -14 -15 78.0989 40.257 8

4 10 -15 44.7891 34.5644 2

4 10 -18 7.54808 19.3861 2

4 10 -18 9.78858 16.8327 3

10 -14 -18 17.3366 18.5623 8

-3 -11 -16 213.299 41.8892 8

-11 14 -16 181.871 44.9149 14

-11 14 -16 172.237 29.6468 11

-11 14 -16 228.35 42.9161 2

-14 3 13-26.4815 71.2796 10

11 -14 13-25.4284 69.7032 10

3 11 13-26.7417 71.9795 10

-3 -11 -13-21.7103 42.5513 6

-11 14 -13 58.7643 49.231 2

-11 14 -13 102.075 57.2823 3

-11 14 -13 97.4676 46.9183 14

-3 -11 -13 112.257 52.4688 8

14 -3 -13 2.74464 35.5778 8

-11 14 -13 57.3116 37.6873 11

-11 14 -10 58.5685 50.7784 11

-11 14 -10 43.0783 56.6661 14

14 -3 -10 35.3603 77.2382 9

-3 -11 -10 111.551 50.7388 6

14 -3 -10-12.0853 70.8426 7

-11 14 -10 63.2134 55.4272 2

14 -3 -10-5.42995 40.2491 8

-3 -11 -10 16.8687 47.7163 8

11 -14 10 54.4401 88.0506 10

-14 3 10 53.235 103.975 10

3 11 7 6400.16 601.357 4

-3 -11 -7 6974.23 566.48 8

-11 14 -7 7060.63 569.742 3

11 -14 7 6571.27 628.085 10

-14 3 7 8410.83 728.73 10

-11 14 -7 6030.81 645.325 4

-11 14 -7 6927.55 568.958 2

-3 -11 -7 6530.54 558.561 1

-3 -11 -7 6704.59 559.638 6

-14 3 4 7624.36 613.619 10

11 -14 4 5886.93 552.505 10

3 11 4 6054.15 557.49 9

-11 14 -4 5748.03 775.993 4

3 11 138976.40 3179.63 9

-3 -11 2 929.578 159.516 10

-11 14 2 846.519 249.599 4

3 11 -529201.50 2057.17 7

-11 14 523508.60 1992.11 4

-3 -11 525953.50 1985.97 10

-11 14 524768.10 1957.27 10

3 11 -524751.80 2028.00 9

11 -14 -522404.70 1892.11 8

14 -3 523659.20 1902.40 9

3 11 -8 524.782 134.662 9

11 -14 -8 377.29 63.6008 8

3 11 -8 326.71 146.17 7

11 -14 -8 408.927 65.805 6

-11 14 8 405.286 105.877 10

-3 -11 8 395.604 145.555 10

-14 3 -8 299.158 86.0599 8

3 11 -8 454.798 62.8759 2

3 11 -11 883.14 85.863 2

11 -14 -11 671.72 85.2814 8

-14 3 -11 464.857 101.279 8

11 -14 -11 870.775 90.4072 6

-11 14 11 362.888 102.226 10

-3 -11 11 368.382 132.657 10

-14 3 -11 507.688 80.6949 14

3 11 -11 506.228 75.4691 14

-14 3 -11 622.829 81.281 11

3 11 -11 357.587 150.548 7

-14 3 -14 756.36 88.8354 2

3 11 -14 628.721 72.114 2

-3 -11 14 304.471 101.938 10

14 -3 14 472.928 122.636 10

3 11 -14 507.59 67.9198 14

-14 3 -14 409.922 71.0774 14

-14 3 -14 581.016 68.2714 11

11 -14 -14 485.661 71.3911 8

11 -14 -17 74.0201 26.9499 8

3 11 -17 138.537 29.2542 2

-2 -12 -17 156.748 32.0589 8

-12 14 -17 171.88 33.1735 2

14 -2 -17 161.765 29.6552 2

-12 14 -14 2628.30 225.049 2

-12 14 -14 2172.20 226.042 3

-2 -12 -14 2800.82 224.79 8

14 -2 -14 2340.18 212.505 8

-12 14 -14 2368.03 213.303 11

14 -2 -11 7654.71 602.073 8

14 -2 -11 6453.61 656.325 7

-2 -12 -11 8348.40 616.799 8

-12 14 -11 7480.53 609.844 11

-2 -12 -11 7989.19 609.203 6

-12 14 -11 5638.99 626.536 3

12 -14 11 6210.68 661.015 10

-14 2 11 6355.57 681.859 10

-12 14 -11 6971.10 619.254 2

-2 -12 -8 4043.59 349.378 1

14 -2 -8 4155.09 344.989 8

-12 14 -8 3912.54 359.853 3

-2 -12 -8 4563.97 358.576 8

12 -14 8 4024.83 413.539 10

-14 2 8 3906.13 474.641 10

-2 -12 -8 4360.03 351.766 6

-12 14 -8 3949.43 360.587 11

-12 14 -8 3628.72 358.90 2

-2 -12 -5 5835.38 493.278 6

-12 14 -5 4787.61 648.386 4

-12 14 -5 5675.05 495.726 2

12 -14 5 4579.34 535.229 10

-14 2 5 6789.21 612.402 10

-2 -12 -5 6859.66 494.994 1

12 -14 2 973.34 108.278 10

-14 2 2 1452.30 189.514 10

-12 14 -2 1229.52 479.071 4

2 12 2 1210.91 164.586 9

-2 -12 1108894.0 8732.11 10

2 12 -1108813.0 8772.52 9

-12 14 1108266.0 8868.47 4

2 12 -410024.00 849.83 7

-12 14 4 9145.29 807.254 4

-2 -12 410673.30 773.982 10

14 -2 4 8526.67 709.625 9

2 12 -4 9881.83 856.441 9

2 12 -711492.00 985.297 4

2 12 -7 9931.36 997.832 9

12 -14 -710502.50 895.395 8

-2 -12 712357.80 1009.15 10

-12 14 711410.80 958.312 10

-14 2 -710593.50 895.938 1

12 -14 -711036.90 898.399 6

-12 14 712247.40 979.078 4

2 12 -711338.60 896.282 2

12 -14 -10 5517.98 471.18 8

-14 2 -10 4773.72 490.031 8

2 12 -10 5706.98 602.298 7

-14 2 -10 5025.49 466.653 14

2 12 -10 6583.60 471.015 14

-2 -12 10 5176.88 558.309 10

-12 14 10 5032.64 513.293 10

12 -14 -10 5668.45 471.294 6

2 12 -10 5611.25 469.963 2

-14 2 -10 5945.01 474.785 11

12 -14 -13 2124.37 191.771 8

-14 2 -13 2312.44 191.084 11

-14 2 -13 2074.06 204.575 8

2 12 -13 2060.58 282.476 7

-14 2 -13 2037.74 208.378 2

2 12 -13 2049.96 191.053 2

-2 -12 13 1553.85 241.57 10

14 -2 13 2115.20 268.568 10

2 12 -13 2204.45 188.796 14

-14 2 -13 1817.63 189.436 14

2 12 -16 276.851 43.0192 2

12 -14 -16 248.135 41.2912 8

-1 -13 -18 256.714 31.2421 8

-13 14 -15 930.706 90.7465 11

-1 -13 -15 927.271 97.7104 8

-13 14 -15 910.326 100.716 2

1 13 12 1296.38 189.163 10

-14 1 12 1084.49 191.386 10

13 -14 12 1205.21 188.354 10

-13 14 -12 912.173 156.824 3

-1 -13 -12 1637.90 146.245 8

-13 14 -12 1158.09 143.956 2

-13 14 -12 1512.83 138.883 11

-1 -13 -12 1356.68 134.985 6

14 -1 -12 1412.46 129.908 8

14 -1 -921019.10 1531.82 9

-13 14 -915621.30 1465.61 3

14 -1 -918533.30 1449.68 8

-1 -13 -919692.00 1463.11 8

-1 -13 -918240.80 1455.79 1

-13 14 -917482.30 1462.22 11

-13 14 -917899.30 1470.08 2

13 -14 915998.00 1512.05 10

-14 1 915885.10 1562.47 10

-1 -13 -917922.70 1455.11 6

-13 14 -6 271.559 49.9851 3

-13 14 -6 87.2779 137.352 4

-1 -13 -6 218.313 56.1192 8

1 13 6 216.077 96.7699 4

-1 -13 -6 272.805 50.1657 6

-13 14 -6 359.027 56.4103 2

-1 -13 -6 318.478 48.8709 1

-14 1 6 368.709 173.912 10

13 -14 6 190.976 99.801 10

13 -14 311785.40 1049.14 10

1 13 312580.90 1035.93 7

-14 1 314980.70 1120.22 10

-13 14 -313203.30 1335.74 4

1 13 313165.00 1087.82 9

1 13 043511.40 3352.14 7

1 13 038175.90 3316.15 9

-13 14 041177.60 3486.77 4

-13 14 3 61.7814 155.799 4

1 13 -3 25.4536 190.511 7

-1 -13 3 205.79 106.031 10

1 13 -3 115.56 150.032 9

14 -1 3 212.333 78.7933 9

13 -14 -612797.10 1036.21 6

-1 -13 613521.30 1128.24 10

-13 14 612166.30 1110.80 10

1 13 -612731.90 1150.10 4

1 13 -6 9846.37 1149.30 9

13 -14 -611647.20 1032.04 8

-13 14 613382.10 1126.96 4

1 13 -613957.60 1215.94 7

1 13 -613179.50 1035.28 2

-14 1 -613688.00 1034.85 1

-14 1 -9 434.917 66.4415 14

1 13 -9 519.434 70.5026 14

1 13 -9 440.342 126.692 9

13 -14 -9 343.759 65.6899 8

1 13 -9 197.956 146.805 7

-13 14 9 313.126 98.5366 10

-1 -13 9 276.929 143.42 10

1 13 -9 502.398 62.0758 11

-14 1 -9 496.129 72.5408 11

1 13 -9 518.188 69.6315 2

-14 1 -9 252.475 93.3273 8

13 -14 -9 439.879 71.3854 6

-14 1 -12 40.9003 42.2175 11

-14 1 -12 17.2255 72.3364 8

13 -14 -12 35.2833 46.0811 8

-14 1 -12-30.3758 48.6884 14

1 13 -12-11.6711 38.4701 14

-14 1 -12 102.965 71.7914 2

1 13 -12 34.6916 44.7356 2

1 13 -12-86.0476 104.481 7

-1 -13 12 46.5145 96.3319 10

14 -1 12 17.3286 103.078 10

13 -14 -15 68.4981 34.4848 8

1 13 -15 34.5096 35.4284 2

1 13 -15 47.8599 33.0285 14

1 13 -18-1.15066 16.6835 3

1 13 -18 25.6731 19.5174 2

13 -14 -18 61.9727 18.0478 8

-14 14 -16 79.0108 23.9423 11

14 0 -16 90.6005 28.235 2

-14 14 -16 57.7643 33.1608 2

0 -14 -16 41.6315 30.3587 8

0 14 13 1036.45 169.237 10

-14 0 13 957.727 175.236 10

14 -14 13 879.863 168.176 10

-14 14 -13 1145.82 140.571 2

0 -14 -13 1602.33 140.675 8

-14 14 -13 1156.65 154.271 3

-14 14 -13 1497.74 132.543 11

14 0 -13 1427.03 124.927 8

0 -14 -13 1322.44 130.135 6

0 -14 -10 184.363 59.2056 6

-14 14 -10 288.933 67.9995 14

-14 14 -10 183.419 60.8513 11

-14 0 10 38.3526 121.713 10

14 -14 10 78.2239 91.7996 10

14 0 -10 187.225 86.1399 9

0 -14 -10 240.433 60.6592 8

-14 14 -10 174.833 73.8359 2

14 0 -10 222.942 91.3503 7

-14 14 -10 87.8949 66.2775 3

14 0 -10 211.716 44.9428 8

0 -14 -7 42.8196 37.9097 1

0 -14 -7 77.5855 46.4902 8

-14 14 -7-101.226 122.286 4

-14 14 -7 6.72651 38.638 3

0 14 7-34.3281 90.2391 4

-14 0 7 23.8136 147.948 10

14 -14 7 37.5048 86.8415 10

-14 14 -7-23.3514 46.1364 2

0 -14 -7 20.4089 40.2918 6

14 -14 447279.40 4149.23 10

0 14 456457.30 4203.21 4

-14 14 -446308.60 4314.04 4

-14 0 457617.70 4215.33 10

0 14 153608.30 4391.02 7

0 14 155194.30 4412.64 9

-14 14 220658.00 1940.80 4

0 -14 223654.40 1857.50 10

14 0 220584.80 1843.42 9

0 14 -532383.70 2595.16 9

14 -14 -530206.50 2382.10 8

14 0 530722.30 2399.05 9

0 14 -530179.00 2384.87 2

-14 14 532794.80 2492.81 4

-14 0 -528918.40 2381.99 1

0 14 -539988.00 2653.55 7

0 -14 536069.60 2475.99 10

-14 14 521167.90 2413.72 10

0 14 -533165.00 2562.93 4

-14 14 8 13.6692 83.8714 10

0 -14 8 72.2582 116.909 10

0 14 -8 81.5083 117.573 9

0 14 -8 53.6636 40.1884 2

-14 0 -8-49.9645 78.0141 8

-14 0 -8 95.7114 49.7374 11

-14 0 -8 136.251 48.7253 1

14 -14 -8 82.5199 45.8512 6

14 -14 -8 26.3921 41.8335 8

0 14 -11 1535.26 157.266 2

-14 0 -11 1707.38 185.655 8

0 -14 11 1443.76 230.898 10

0 14 -11 2023.42 157.237 14

-14 0 -11 1309.19 152.181 14

-14 0 -11 1992.61 163.351 11

0 14 -11 1278.56 256.399 7

14 -14 -11 1404.34 155.059 8

0 14 -14 326.983 52.1816 14

0 14 -14 350.39 54.2113 2

14 -14 -14 217.038 51.0695 8

14 -14 -17 60.8754 22.5186 8

0 14 -17 72.1334 27.215 2

0 14 -17 109.137 27.0318 3

15 -14 -16 9.38693 25.6527 8

-1 15 -16 37.4605 27.4846 3

-1 15 -16-17.4414 27.9327 2

15 -14 -13 380.71 65.0642 8

-14 -1 -13 476.714 84.5445 8

1 -15 13 345.894 108.053 10

14 1 13 560.583 140.127 10

-1 15 -13 286.937 64.8395 2

-1 15 -13 507.407 148.209 7

-1 15 -13 627.555 66.5481 14

-1 15 -10 51.1887 38.3115 11

-15 14 10 159.099 80.3329 10

1 -15 10 -53.416 104.436 10

-1 15 -10 40.4729 46.6721 2

15 -14 -10 106.211 47.1238 8

-14 -1 -10 7.68091 84.3465 8

-1 15 -10 190.584 162.038 7

-14 -1 -10 82.223 47.597 11

-1 15 -10 102.98 47.2314 14

-14 -1 -10 58.9589 40.362 14

-15 14 7 679.555 164.004 10

1 -15 7 625.964 156.088 10

-1 15 -7 674.932 218.536 9

15 -14 -7 701.652 90.3311 8

-1 15 -7 844.064 92.2308 2

-14 -1 -7 936.822 91.2832 1

-1 15 -7 862.869 197.085 4

-15 14 7 1034.11 170.164 4

-1 15 -7 1218.44 269.435 7

15 -14 -7 704.662 88.4483 6

1 -15 4 3645.54 275.035 10

-1 15 -4 3008.44 442.344 4

-15 14 4 2827.72 330.685 4

-1 15 -4 4835.56 509.136 7

14 1 4 3485.99 225.802 9

-1 15 -4 4333.44 450.136 9

1 -15 1 3220.56 320.02 10

14 1 1 2936.10 321.065 9

-15 14 1 2692.59 438.358 4

-1 15 -1 3701.39 401.724 7

-14 -1 2 697.843 154.545 10

-1 15 2 560.404 137.708 9

15 -14 2 597.91 77.878 10

-15 14 -2 760.03 336.803 4

-1 15 2 709.864 92.8437 7

15 -14 526970.80 2292.85 10

1 -15 -531396.10 2240.59 8

1 -15 -525360.70 2234.15 1

-15 14 -524988.80 2390.28 4

-15 14 -527241.40 2238.02 2

-1 15 530718.10 2326.86 4

-15 14 -8 6126.20 482.322 11

1 -15 -8 5632.82 468.191 6

-15 14 -8 6971.83 479.024 14

15 -14 8 5429.81 529.312 10

14 1 -8 7286.87 554.312 9

-14 -1 8 6407.85 621.892 10

-15 14 -8 5264.76 476.918 2

1 -15 -8 6007.18 473.298 8

1 -15 -8 5068.77 464.093 1

-15 14 -8 4112.40 513.667 4

-15 14 -8 4714.28 467.351 3

-15 14 -11 1035.48 108.92 11

-15 14 -11 1213.20 116.431 14

15 -14 11 754.463 151.407 10

-14 -1 11 471.144 155.409 10

-1 15 11 678.516 144.774 10

-15 14 -11 531.214 122.157 3

-15 14 -11 845.576 121.607 2

14 1 -11 877.801 154.758 7

14 1 -11 971.951 96.278 8

1 -15 -11 1024.50 104.916 6

1 -15 -11 917.637 109.042 8

-15 14 -14 148.667 50.1554 14

1 -15 -14 6.41484 40.9188 8

-15 14 -14 12.4462 47.1517 2

-15 14 -14 21.2817 33.8668 11

1 -15 -17 154.21 30.0035 8

14 1 -17 195.258 28.972 2

-15 14 -17 177.694 23.197 11

15 -13 -17 123.977 26.3806 8

-2 15 -17 121.286 31.1369 2

-2 15 -17 167.445 30.0552 3

-13 -2 -14 1314.67 126.917 8

-2 15 -14 1241.70 119.454 2

-2 15 -14 966.189 110.29 14

15 -13 -14 1176.13 115.984 8

-15 13 11 47.2512 66.9961 10

2 -15 11 99.1628 93.7318 10

-13 -2 -11 18.3118 45.9806 11

-2 15 -11 40.8443 40.5059 14

-13 -2 -11-73.4443 49.2578 14

-2 15 -11-26.3783 133.806 7

-2 15 -11 26.0197 43.3776 2

-2 15 -11 40.2238 34.8065 11

-13 -2 -11-43.7736 69.4381 8

15 -13 -11 27.877 45.6427 8

-2 15 -8 4147.85 517.414 9

15 -13 -8 4584.38 393.585 8

-13 -2 -8 4767.80 394.495 1

-13 -2 -8 3505.26 413.069 8

-15 13 8 4695.24 478.689 10

2 -15 8 3985.20 475.458 10

15 -13 -8 4676.31 392.983 6

-13 -2 -8 5401.58 404.802 11

-2 15 -8 4825.09 395.956 2

-2 15 -546235.40 3537.03 2

-15 13 549904.90 3639.49 4

-2 15 -546424.70 3766.45 9

13 2 543865.20 3551.94 9

-2 15 -564609.00 3919.90 7

-13 -2 -542883.40 3532.62 1

-2 15 -550732.50 3780.31 4

-15 13 533746.80 3567.12 10

2 -15 550657.20 3614.41 10

-2 15 -212100.40 1049.01 7

-2 15 -210712.90 996.256 9

-2 15 -211132.80 1174.25 4

2 -15 211213.20 925.887 10

13 2 210212.40 922.577 9

-15 13 210292.60 1025.80 4

15 -13 120474.90 1748.02 10

-2 15 124601.30 1794.77 7

-2 15 124785.00 1841.77 9

-2 15 4 388.367 152.334 4

15 -13 4 305.161 99.0774 10

-13 -2 4 175.306 135.718 10

-15 13 -4 149.647 222.932 4

2 -15 -4 366.426 53.5588 6

15 -13 710579.80 858.167 10

-13 -2 711802.40 956.37 10

13 2 -710962.50 868.985 9

-15 13 -7 9670.34 791.888 2

2 -15 -710268.50 791.519 8

2 -15 -7 9215.94 784.389 1

-15 13 -7 9061.75 786.156 3

-15 13 -7 9072.43 872.939 4

-2 15 7 9053.46 849.884 4

-15 13 -10 1964.35 235.494 3

13 2 -10 3030.16 287.449 9

2 -15 -10 2608.74 232.836 8

13 2 -10 2763.40 221.008 8

13 2 -10 2370.43 297.573 7

-15 13 -10 2372.56 245.89 2

-13 -2 10 2272.40 324.04 10

15 -13 10 2062.48 274.81 10

2 -15 -10 2556.86 228.628 6

-15 13 -10 2719.61 234.837 14

-15 13 -10 2255.29 230.136 11

2 -15 -13 1912.84 184.131 8

-15 13 -13 2140.82 181.814 11

2 -15 -13 2049.23 180.928 6

15 -13 13 1444.28 220.401 10

-2 15 13 1529.27 217.778 10

-15 13 -13 2252.40 184.374 14

-15 13 -13 1815.75 192.956 2

-13 -2 13 1474.64 227.919 10

-15 13 -16-15.2616 31.7142 2

2 -15 -16 6.40935 27.1833 8

-15 13 -16 -17.583 35.5592 14

13 2 -16 7.3206 24.6349 2

-15 13 -16 7.36632 21.0271 11

-3 15 -18 26.7611 19.8191 2

-3 15 -15 53.2135 34.9765 3

-3 15 -15-16.6145 36.8834 2

-3 15 -15 35.4371 32.3306 14

-12 -3 -15 12.0628 36.7354 8

15 -12 -15 33.8325 31.4157 8

-3 15 -12 697.206 80.7429 14

15 -12 -12 590.853 80.1468 8

-3 15 -12 626.701 82.8078 2

-3 15 -12 563.14 180.954 7

3 -15 12 499.919 133.385 10

-3 15 -12 603.107 74.8331 11

-12 -3 -12 766.79 103.511 8

-12 -3 -9 685.46 115.612 1

-3 15 -9 1621.50 130.493 14

-15 12 9 1023.90 176.433 10

3 -15 9 1126.44 199.081 10

-12 -3 -9 1208.68 141.076 8

15 -12 -9 1059.56 115.753 8

-3 15 -9 1279.88 120.574 11

-12 -3 -9 1490.59 126.921 11

-3 15 -9 1502.63 317.92 7

-3 15 -9 810.094 113.76 2

-3 15 -6 7598.81 764.292 4

-3 15 -6 5322.56 759.687 9

15 -12 -6 6500.32 574.379 8

-3 15 -6 6982.45 578.597 2

-15 12 6 8513.14 672.774 4

15 -12 -6 6756.00 575.654 6

-12 -3 -6 7397.08 576.861 1

3 -15 6 7722.88 661.947 10

-15 12 6 6853.09 674.779 10

-3 15 -6 9680.60 952.754 7

-15 12 314295.90 1003.24 4

-3 15 -317444.30 1139.91 7

3 -15 315108.20 913.08 10

-3 15 -315860.00 1055.68 9

12 3 314513.80 897.449 9

-15 12 0 7520.08 810.373 4

-3 15 0 7359.63 667.618 7

-3 15 0 8076.14 828.262 4

-3 15 0 7378.14 695.139 9

12 3 0 7601.59 677.54 9

-12 -3 321255.40 1737.81 10

-3 15 320774.30 1752.32 4

-15 12 -318610.20 1834.59 4

-12 -3 6 145.10 154.462 10

15 -12 6 95.8988 86.3138 10

12 3 -6 77.018 95.5422 9

3 -15 -6 287.446 56.2269 8

-15 12 -6 346.729 55.0641 2

-3 15 6 313.63 121.574 4

-15 12 -6 358.505 146.415 4

15 -12 9 2640.78 277.685 10

-12 -3 9 2199.95 318.581 10

12 3 -9 2273.23 258.65 9

3 -15 -9 2335.97 210.89 8

12 3 -9 2309.26 201.787 8

12 3 -9 2369.04 290.144 7

-15 12 -9 2846.89 222.953 14

-15 12 -9 2056.26 220.065 2

-15 12 -9 1596.74 204.588 3

-15 12 -9 2515.61 216.922 11

3 -15 -12 911.53 99.1765 8

-15 12 -12 775.626 92.1602 11

-15 12 -12 671.315 91.7277 14

-3 15 12 830.532 135.41 10

-12 -3 12 483.05 135.882 10

15 -12 12 476.597 127.498 10

12 3 -12 718.255 139.146 7

3 -15 -12 987.786 98.8298 6

-15 12 -12 675.545 107.288 2

-15 12 -15 28.3384 41.243 14

12 3 -15 27.2652 30.1768 2

3 -15 -15 35.4003 37.6569 8

-15 12 -15 31.7715 27.0043 11

-15 12 -15-27.3504 38.977 2

3 -15 -18 9.50006 16.949 8

-4 15 -16 57.4982 28.6324 3

15 -11 -16 62.3311 28.4637 8

-4 15 -16 66.3731 33.0639 2

-4 15 -16 -29.248 34.034 14

15 -11 -13 211.403 46.2528 8

-11 -4 -13 165.239 57.2813 8

4 -15 13 248.052 93.113 10

-4 15 -13 105.239 49.6697 2

-4 15 -13 112.02 40.9176 14

-4 15 -13 101.715 36.2356 11

-4 15 -10 1651.56 148.971 2

15 -11 -10 1407.22 140.253 8

-11 -4 -10 1748.27 161.786 8

-4 15 -10 1761.64 340.735 7

-4 15 -10 1512.61 143.777 11

-11 -4 -10 1572.92 146.758 11

4 -15 10 1364.06 218.585 10

-15 11 10 1380.47 203.225 10

-4 15 -10 1626.32 146.032 14

-11 -4 -10 936.452 135.815 14

-11 -4 -10 1331.49 135.639 6

-15 11 7 522.232 155.579 10

4 -15 7 334.302 127.989 10

-15 11 7 479.587 123.085 4

15 -11 -7 471.305 62.9745 8

-4 15 -7 591.378 179.278 4

-11 -4 -7 445.987 60.7804 6

15 -11 -7 540.468 66.4064 6

-11 -4 -7 497.064 64.7696 1

-4 15 -7 374.504 64.0521 2

-11 -4 -7 300.656 81.7499 8

-4 15 -4 6939.69 698.181 9

11 4 4 6337.04 521.826 9

-4 15 -4 8125.85 797.351 7

-15 11 4 6438.24 597.765 4

4 -15 4 7224.21 555.885 10

-4 15 -4 7302.10 904.514 4

11 4 1 4504.13 453.791 9

-15 11 1 5502.88 572.769 4

-4 15 -1 4683.06 474.808 7

4 -15 1 5001.35 437.207 10

-4 15 216944.20 1321.72 4

-15 11 -215566.30 1405.28 4

-4 15 214079.40 1201.64 7

11 4 -215033.40 1261.80 9

4 -15 -5 8462.07 663.239 8

-15 11 -5 6393.08 764.295 4

4 -15 -5 7835.17 662.271 6

11 4 -5 8762.63 742.271 9

-4 15 5 8291.15 750.839 4

4 -15 -8 4192.00 361.357 8

-15 11 -8 4156.15 366.996 2

-15 11 -8 4427.61 369.73 11

11 4 -8 4653.67 355.57 8

-11 -4 8 5249.64 513.259 10

15 -11 8 3969.75 418.263 10

-15 11 -8 3559.47 353.687 3

11 4 -8 4501.48 422.675 9

-15 11 -11 91.0485 56.6521 14

-15 11 -11-26.8906 60.6874 2

11 4 -11-31.7184 89.9449 7

11 4 -11-2.85643 41.9376 8

-15 11 -11 35.6282 45.4651 11

4 -15 -11 56.37 52.575 8

-4 15 11-11.7204 67.5722 10

-11 -4 11-86.2562 101.048 10

15 -11 11 -13.411 87.8267 10

-15 11 -14 296.966 52.0731 11

11 4 -14 350.78 53.5959 2

11 4 -17 90.9563 24.9653 2

4 -15 -17 55.4489 25.5591 8

-5 15 -17 85.8648 30.7879 2

15 -10 -17 101.611 24.9582 8

-5 15 -14 140.073 50.6078 2

15 -10 -14 215.889 46.7401 8

-10 -5 -14 209.869 55.6529 8

-5 15 -14 190.606 37.0424 11

-5 15 -14 278.499 51.7147 14

-15 10 11 892.657 164.39 10

5 -15 11 998.698 178.813 10

-5 15 -11 1400.82 129.468 2

15 -10 -11 1157.96 117.629 8

-10 -5 -11 1297.30 132.501 8

-5 15 -11 1030.46 119.414 14

-5 15 -11 1142.18 117.575 11

-5 15 -11 1109.48 253.357 7

-10 -5 -11 1057.83 114.566 6

-15 10 8 4761.45 497.815 10

5 -15 8 4535.76 479.488 10

-5 15 -8 4417.62 399.619 2

-10 -5 -8 4929.44 395.542 6

-10 -5 -8 5236.06 414.113 8

15 -10 -8 4395.09 392.868 8

-5 15 -5113807.0 9527.15 2

-15 10 5113083.0 9598.40 10

5 -15 5127731.0 9591.06 10

-5 15 -5132829.0 9842.65 4

10 5 5119627.0 9548.35 9

-15 10 5132541.0 9621.35 4

-5 15 -221405.10 1803.20 7

10 5 220442.20 1747.30 9

5 -15 220856.40 1734.57 10

-5 15 -225712.00 2206.77 4

-15 10 222930.40 1845.90 4

-5 15 132365.10 2610.43 7

10 5 -130854.80 2645.66 9

-5 15 134454.70 2740.08 4

-5 15 410177.40 882.924 4

10 5 -410311.70 864.84 9

-15 10 -4 8039.28 904.842 4

5 -15 -7 906.079 96.5406 6

5 -15 -7 1006.63 97.6072 1

-5 15 7 960.48 166.228 4

10 5 -7 695.766 139.033 9

-15 10 -7 650.88 90.8347 2

-15 10 -10 7918.16 713.261 2

10 5 -10 8840.95 689.426 8

10 5 -10 8402.79 783.271 7

-15 10 -10 8299.34 698.627 14

10 5 -10 8565.41 687.291 14

10 5 -10 8476.99 744.237 9

-10 -5 10 7236.39 784.084 10

15 -10 10 6948.64 747.287 10

-5 15 10 7805.37 743.424 10

-15 10 -10 8787.57 698.344 11

10 5 -13 41.5758 73.5824 7

-15 10 -13 65.2161 41.5334 11

-10 -5 13 42.2409 78.897 10

15 -10 13 90.4118 87.02 10

-5 15 13 51.1208 63.1126 10

5 -15 -13 132.771 48.8637 6

10 5 -13 142.817 43.0686 2

-15 10 -13 49.1179 57.8769 2

-15 10 -13 180.85 54.8687 14

-15 10 -16 24.6881 21.6885 11

10 5 -16 3.95188 26.0546 2

-15 10 -16-10.3489 37.3761 14

15 -9 -18 135.696 23.3093 2

-6 15 -15 704.497 75.3293 14

-6 15 -15 493.824 72.6999 3

-9 -6 -15 606.443 77.0572 8

15 -9 -15 712.816 74.153 8

-6 15 -12 4397.65 382.382 2

15 -9 -12 4749.57 377.422 8

-9 -6 -12 4951.68 388.302 8

-6 15 -12 4493.27 377.754 14

6 -15 12 3677.61 436.274 10

-15 9 12 3243.08 420.064 10

-6 15 -12 4341.12 374.94 11

-6 15 -9 986.592 103.652 11

15 -9 -9 793.245 92.0725 8

-9 -6 -9 849.814 105.612 8

-6 15 -9 818.786 102.984 2

-9 -6 -9 899.843 97.8008 6

-15 9 9 952.575 178.781 10

6 -15 9 507.187 147.988 10

15 -9 -9 766.224 117.732 7

-6 15 -9 778.147 297.233 7

-6 15 -9 1005.45 107.097 14

-6 15 -6 2135.84 213.404 2

-6 15 -6 2427.40 380.099 4

-15 9 6 2640.64 294.618 4

-15 9 6 2792.11 343.467 10

6 -15 6 2907.58 292.405 10

6 -15 319569.80 1539.11 10

9 6 319562.40 1540.14 9

-15 9 321264.50 1625.55 4

-15 9 315652.30 1527.80 10

-6 15 -321160.60 1653.55 7

-6 15 -321820.00 2149.62 4

-15 9 034529.00 3210.02 4

-6 15 038358.30 3102.74 7

9 6 037246.90 3120.02 9

-15 9 -3 1386.78 301.615 4

-9 -6 3 2035.29 237.334 10

-6 15 3 1314.32 243.206 4

-15 9 -6 105.478 111.532 4

6 -15 -6 183.389 48.2088 6

9 6 -6 211.862 103.298 9

-6 15 6 90.5425 115.602 4

6 -15 -9 125.197 55.3643 6

9 6 -9 33.7832 71.0128 9

9 6 -9 34.2038 92.0802 7

9 6 -9 52.0658 37.4544 8

-6 15 9 33.5639 76.9229 10

15 -9 9-27.9274 89.1973 10

-15 9 -9 25.3199 45.5462 11

-15 9 -9 93.7904 55.7974 14

-15 9 -9 -28.34 59.0866 2

-15 9 -12 12.8348 61.2692 2

-15 9 -12 36.1397 43.1654 11

-9 -6 12 15.5966 88.4581 10

15 -9 12 -97.571 90.1363 10

-6 15 12 10.8198 69.0673 10

-15 9 -12 51.7348 54.0503 14

9 6 -12 16.0034 77.7279 7

-15 9 -15-74.8044 42.4251 14

-15 9 -15 28.6802 41.0024 2

9 6 -15 2.41578 30.9423 2

-15 9 -15 23.6495 29.7023 11

9 6 -18 15.2738 17.0632 2

-8 -7 -16 314.523 47.7323 8

15 -8 -16 310.006 42.1553 8

-7 15 -16 333.089 46.1379 3

-7 15 -16 208.343 47.2347 14

15 -8 -13 94.3606 42.3329 8

-8 -7 -13 86.7608 50.0381 8

-15 8 13 226.951 74.7856 10

7 -15 13 105.55 80.1965 10

8 7 13 96.0978 97.1435 10

-7 15 -13 123.923 49.2644 14

-7 15 -13 123.213 37.0421 11

-7 15 -10 884.84 312.428 7

15 -8 -10 757.138 89.872 8

-8 -7 -10 867.301 102.926 8

7 -15 10 855.003 169.811 10

-15 8 10 738.54 156.405 10

-7 15 -10 904.131 102.357 14

-8 -7 -10 941.023 98.5087 6

-7 15 -10 739.146 95.6259 11

-7 15 -724247.20 2059.49 2

-8 -7 -727754.00 2056.24 1

-8 -7 -726213.90 2053.63 6

15 -8 -723262.30 2050.12 8

-8 -7 -727384.10 2064.95 8

7 -15 726766.50 2138.54 10

-15 8 726427.80 2194.42 10

-7 15 -723169.30 2171.69 4

-15 8 413226.90 1224.42 10

-7 15 -416213.50 1367.45 7

8 7 414181.20 1205.89 9

-7 15 -417457.20 1687.77 4

7 -15 414413.30 1216.90 10

-15 8 416584.00 1285.34 4

-7 15 -114836.80 1313.69 7

7 -15 115608.20 1275.05 10

-15 8 116211.10 1412.10 4

8 7 116119.90 1316.21 9

-8 -7 2-24.9199 94.8086 10

8 7 -2-13.5225 113.752 9

-7 15 2 186.262 183.091 4

-15 8 -2 77.56 177.569 4

-8 -7 5 9862.38 758.436 10

-15 8 -5 7440.09 748.663 4

7 -15 -5 7423.07 635.034 6

-7 15 5 8045.66 738.491 4

8 7 -5 8592.19 721.157 9

7 -15 -5 7714.37 634.473 8

7 -15 -8 1599.31 140.986 8

8 7 -8 1652.36 230.436 7

-7 15 8 1250.13 173.676 10

7 -15 -8 1406.67 139.357 6

-15 8 -8 1140.05 141.015 2

-15 8 -8 1346.83 143.509 11

8 7 -8 1547.00 194.92 9

7 -15 -11 3629.58 313.019 8

8 7 -11 3280.17 395.641 7

-15 8 -11 3536.67 310.163 11

7 -15 -11 3548.12 311.779 6

-7 15 11 3188.68 352.646 10

15 -8 11 3080.09 368.975 10

8 7 -11 4198.48 308.055 14

-15 8 -11 3552.63 310.852 14

-15 8 -11 2714.53 324.755 2

-15 8 -14-2.76082 34.9979 11

7 -15 -14 31.498 42.3291 8

8 7 -14 49.9442 35.6376 2

-15 8 -14 83.3104 53.5095 2

-15 8 -14 159.193 48.792 14

7 -15 -17 99.4895 28.5519 8

8 7 -17 114.109 28.5886 2

-8 15 -17-27.4892 27.2129 2

-8 15 -14 914.609 96.0992 3

-7 -8 -14 778.678 93.5654 8

15 -7 -14 828.147 85.8188 8

-8 15 -14 657.166 79.7895 11

-8 15 -14 851.82 91.6405 14

-8 15 -11 3168.02 255.748 14

-7 -8 -11 2606.14 246.206 6

15 -7 -11 2806.22 244.196 8

-7 -8 -11 2907.11 255.137 8

8 -15 11 2218.80 305.22 10

-15 7 11 2178.28 305.872 10

-8 15 -11 2872.95 250.23 11

-8 15 -8 836.965 415.269 7

15 -7 -8 633.523 80.213 8

-7 -8 -8 833.377 96.1632 8

-7 -8 -8 705.858 87.4141 6

8 -15 8 490.525 140.306 10

-15 7 8 820.094 190.495 10

-7 -8 -8 1021.51 93.576 1

-8 15 -521847.80 2123.95 4

-15 7 524990.90 1965.40 4

-8 15 -523409.50 1869.48 3

7 8 521278.30 1893.68 9

-15 7 524805.20 1971.32 10

8 -15 522925.80 1918.79 10

-8 15 -527163.90 2134.44 7

-15 7 2 9921.34 832.533 4

7 8 2 8446.38 732.236 9

-8 15 -2 8295.67 756.403 7

8 -15 2 8567.44 701.198 10

-15 7 2 7396.35 720.816 10

-8 15 142604.70 3344.73 4

-7 -8 141844.00 3232.85 10

7 8 -135919.20 3229.71 9

-15 7 -440031.30 3327.26 4

7 8 -437003.40 3273.85 9

-8 15 441796.70 3311.59 4

-7 -8 441557.30 3283.15 10

8 -15 -725561.70 2111.10 8

-8 15 727123.50 2194.86 4

8 -15 -725775.60 2113.11 6

7 8 -727564.10 2183.62 9

-7 -8 729649.10 2247.72 10

-8 15 725305.30 2153.80 10

7 8 -729775.40 2225.28 7

-15 7 -1016020.50 1568.80 2

7 8 -1018736.90 1604.12 9

7 8 -1020289.60 1663.61 7

-15 7 -1019754.30 1557.50 11

8 -15 -1019673.40 1555.86 6

-8 15 1017594.20 1596.94 10

-7 -8 1017114.50 1642.26 10

15 -7 1017342.80 1625.22 10

-15 7 -1018641.40 1555.66 14

7 8 -1022576.90 1550.79 14

8 -15 -1018268.50 1554.25 8

8 -15 -13 17.4151 46.7492 8

-15 7 -13 160.407 51.7718 14

7 8 -13 95.6124 79.3802 7

15 -7 13 153.964 91.8747 10

-15 7 -13 90.2487 45.2141 11

7 8 -13 85.3631 42.9311 2

-15 7 -13 17.1821 54.3916 2

8 -15 -16 8.66683 29.7926 8

7 8 -16 22.7977 26.2282 2

-9 15 -18 129.361 26.089 2

15 -6 -18 129.238 22.697 2

-9 15 -15 28.1773 34.3246 3

-9 15 -15-2.99518 39.6209 2

-9 15 -15 25.4746 25.0159 11

-6 -9 -15 98.8793 41.0673 8

15 -6 -15 63.7506 33.0972 8

-9 15 -15 87.903 43.6318 14

-9 15 -12 530.401 84.6542 2

-6 -9 -12 592.841 83.4312 8

15 -6 -12 657.187 71.499 8

-6 -9 -12 609.427 76.2716 6

-9 15 -12 422.92 78.0281 14

-15 6 12 400.12 116.238 10

9 -15 12 260.252 110.153 10

-9 15 -12 429.94 68.1868 11

-9 15 -9 8.27334 52.1162 14

-9 15 -9 68.6944 44.9073 11

-6 -9 -9 17.8691 44.2607 1

-6 -9 -9 33.0063 39.7195 6

9 -15 9 14.7478 97.7823 10

-15 6 9-76.3733 121.873 10

-6 -9 -9-4.13932 49.0641 8

15 -6 -9-2.66195 30.7066 8

-6 -9 -6 328.19 49.4011 1

-9 15 -6 27.8641 173.518 4

-9 15 -6 132.712 255.308 7

-15 6 6 128.495 150.868 10

9 -15 6 146.397 90.9946 10

-6 -9 -6 64.0774 37.0657 6

-9 15 -312595.10 1163.86 7

-15 6 316365.10 1222.66 4

6 9 313263.30 1111.65 9

-9 15 -316720.10 1701.45 4

-15 6 311932.70 1117.51 10

9 -15 313328.40 1096.37 10

-15 6 042174.70 3703.88 4

6 9 042664.40 3614.28 9

-9 15 317318.50 1524.05 4

-6 -9 317647.90 1472.25 10

-15 6 -316820.40 1546.67 4

6 9 -6 5883.00 565.753 9

9 -15 -6 5839.41 486.224 8

9 -15 -6 5632.35 488.933 6

-9 15 6 6153.84 582.028 4

-6 -9 6 7820.01 624.09 10

-9 15 6 6167.50 532.974 10

6 9 -6 5774.99 484.673 2

-9 15 9 264.068 85.3204 10

-6 -9 9 313.928 137.53 10

6 9 -9 489.847 113.196 9

9 -15 -9 338.44 61.855 8

-15 6 -9 404.588 81.9988 8

9 -15 -9 268.901 61.8391 6

6 9 -9 420.595 146.706 7

-15 6 -9 532.13 75.2462 14

6 9 -9 242.163 56.1185 2

-15 6 -9 323.591 74.566 2

-15 6 -9 435.677 68.3132 11

9 -15 -12 264.678 56.9858 6

6 9 -12 290.381 119.699 7

-15 6 -12 184.297 58.1422 14

6 9 -12 151.623 49.814 14

-6 -9 12 46.9542 88.4238 10

15 -6 12 60.9515 90.3797 10

-9 15 12 82.4144 68.1169 10

-15 6 -12 160.862 51.9221 11

-15 6 -12 176.205 74.9604 2

6 9 -12 348.013 55.7306 2

9 -15 -12 118.336 54.664 8

-15 6 -15 95.5658 32.674 11

6 9 -15 112.615 35.9365 2

9 -15 -15 55.7813 36.9756 8

-15 6 -15 23.526 42.7861 14

6 9 -18 23.182 18.6861 2

6 9 -18 23.5043 18.3062 3

9 -15 -18 37.049 18.6589 8

-5 -10 -16 138.186 36.4191 8

-10 15 -16 117.971 39.5081 14

-10 15 -16 119.188 36.7052 2

-10 15 -16 113.372 24.8486 11

-10 15 -13-2.94621 36.0004 11

-5 -10 -13 30.3083 38.9762 6

-10 15 -13 64.0693 50.547 14

-5 -10 -13 46.116 50.5143 8

15 -5 -13 8.25944 34.5986 8

-10 15 -13 8.03906 46.3722 2

-10 15 -13 20.5704 48.8279 3

5 10 13-14.2828 79.6559 10

-15 5 13 25.4785 72.2653 10

10 -15 13 63.663 73.4207 10

-10 15 -10 351.652 75.5334 2

-10 15 -10 250.299 60.8578 11

-10 15 -10 323.035 70.6123 14

15 -5 -10 196.618 76.127 7

-5 -10 -10 394.662 64.9024 6

15 -5 -10 305.218 52.4221 8

-5 -10 -10 328.111 65.9148 8

10 -15 10 171.334 92.9366 10

-15 5 10 174.116 112.145 10

-5 -10 -7 7947.69 649.205 8

-5 -10 -7 7960.72 641.656 6

-5 -10 -7 8729.58 644.139 1

-15 5 7 8800.94 800.138 10

10 -15 7 6213.82 697.418 10

-10 15 -7 7341.86 649.048 2

-10 15 -7 5503.66 718.954 4

-10 15 -4 4121.71 454.436 7

5 10 4 3873.69 373.199 9

-10 15 -4 5345.70 761.788 4

-15 5 4 4032.09 409.655 10

10 -15 4 3782.40 367.44 10

-15 5 1 2475.90 416.119 4

5 10 1 3523.90 336.176 9

-10 15 2 5943.03 595.962 4

-15 5 -2 4361.40 575.15 4

-5 -10 2 5066.11 487.79 10

5 10 -5 676.765 161.261 9

15 -5 5 510.203 68.298 9

10 -15 -5 343.684 53.2721 8

10 -15 -5 218.884 53.5866 6

-10 15 5 515.909 162.134 4

-10 15 5 523.635 101.547 10

-5 -10 5 577.11 155.985 10

-5 -10 8 1518.69 248.663 10

5 10 -8 1804.54 224.915 9

10 -15 -8 1540.46 149.493 8

-15 5 -8 1159.72 159.857 8

-15 5 -8 1602.10 156.23 11

10 -15 -8 1744.67 155.965 6

-10 15 8 1641.77 192.557 10

5 10 -8 1498.59 244.525 7

5 10 -8 1812.67 149.705 2

-15 5 -8 1272.02 150.946 2

10 -15 -11 36.9319 51.6321 6

10 -15 -11 86.1832 51.7983 8

-15 5 -11 6.40664 72.8989 8

-15 5 -11 87.1964 70.8176 2

5 10 -11 60.5969 44.4721 2

5 10 -11 91.9387 44.6668 14

-15 5 -11 141.963 54.209 14

5 10 -11 78.6414 103.963 7

-10 15 11 51.7215 62.7229 10

15 -5 11 16.3192 104.324 10

-5 -10 11 119.609 117.501 10

-15 5 -11 76.098 45.8038 11

-15 5 -14 222.91 53.5688 14

-15 5 -14 181.431 59.8625 2

5 10 -14 166.68 47.8959 2

10 -15 -14 189.908 50.7693 8

-15 5 -14 282.905 47.1998 11

5 10 -17 28.1461 24.2935 2

10 -15 -17 11.3283 23.4507 8

-4 -11 -17 279.401 37.0416 8

-11 15 -17 165.98 39.5712 14

15 -4 -17 290.232 36.2841 2

-11 15 -17 223.664 37.8509 2

-4 -11 -14 202.48 53.1884 8

15 -4 -14 147.528 39.3843 8

-11 15 -14 108.922 49.4872 3

-11 15 -14 212.399 50.2416 2

-11 15 -14 319.786 57.5794 14

-11 15 -14 138.503 38.5808 11

-11 15 -11 1320.83 126.836 11

-15 4 11 748.685 174.718 10

11 -15 11 864.872 174.105 10

-11 15 -11 1187.98 136.485 2

-4 -11 -11 1225.86 131.299 8

15 -4 -11 1140.50 115.611 8

-11 15 -11 1080.35 149.957 3

-4 -11 -11 1113.98 122.399 6

-11 15 -11 1294.23 132.359 14

-4 -11 -8 3064.65 259.722 1

-11 15 -8 3044.51 266.858 14

-11 15 -8 2645.33 267.586 2

-4 -11 -8 3185.83 267.503 8

15 -4 -8 2766.93 252.255 8

11 -15 8 2661.10 320.422 10

-15 4 8 3052.74 387.833 10

-4 -11 -8 3126.05 260.09 6

-11 15 -8 3028.50 270.846 11

-11 15 -8 2923.23 274.525 3

-11 15 -520642.20 1689.28 3

-11 15 -521163.90 1690.37 2

-11 15 -518479.20 1885.81 4

-4 -11 -521553.60 1686.10 6

-15 4 524710.90 1815.10 10

11 -15 519605.70 1733.51 10

-4 -11 -520580.10 1685.98 1

-15 4 2 2648.11 274.142 10

4 11 2 2357.35 253.845 9

11 -15 2 2111.78 202.334 10

-11 15 124885.00 2111.69 4

-4 -11 123752.10 1956.01 10

4 11 -122779.90 1980.40 9

-11 15 430163.20 2351.31 4

-11 15 424015.50 2253.99 10

-4 -11 431276.00 2305.88 10

4 11 -431152.00 2358.00 9

-15 4 -7 1328.48 161.795 8

11 -15 -7 1764.05 161.117 6

-15 4 -7 1595.05 157.518 1

-11 15 7 1706.42 203.699 10

-4 -11 7 1663.04 251.799 10

4 11 -7 1505.36 233.931 9

11 -15 -7 1650.43 156.263 8

-11 15 7 1741.83 232.212 4

4 11 -7 1949.11 159.493 2

4 11 -7 1932.36 233.508 4

-15 4 -10 585.383 110.067 2

4 11 -10 715.761 89.8176 2

11 -15 -10 638.45 91.8331 8

-15 4 -10 546.373 118.732 8

11 -15 -10 800.424 96.3493 6

4 11 -10 941.208 90.0839 14

-15 4 -10 807.70 92.2439 14

4 11 -10 646.093 169.467 7

-11 15 10 574.524 119.073 10

-4 -11 10 697.521 168.808 10

-15 4 -10 933.822 99.0848 11

11 -15 -13 80.8773 48.6201 8

-15 4 -13 96.466 64.4582 2

4 11 -13 158.379 47.2839 2

-15 4 -13 73.0443 46.7145 14

4 11 -13 123.243 45.7575 14

4 11 -13 35.5714 86.7409 7

-4 -11 13 267.66 98.1698 10

15 -4 13 14.9315 80.7255 10

-15 4 -13 78.8892 42.0826 11

4 11 -16 17.0009 28.0475 2

11 -15 -16 20.887 27.2268 8

-12 15 -18 69.7933 20.6294 2

-12 15 -15 432.432 55.0411 11

-3 -12 -15 488.948 65.6467 8

-12 15 -15 473.33 65.8418 2

-12 15 -15 525.806 67.4658 14

-3 -12 -12 569.862 73.9287 6

-12 15 -12 387.048 75.8919 14

-15 3 12 336.937 120.483 10

12 -15 12 379.445 117.241 10

3 12 12 340.324 112.365 10

-12 15 -12 583.904 90.0582 2

-3 -12 -12 677.665 84.6003 8

-12 15 -12 540.397 74.6658 11

-12 15 -12 382.816 94.6535 3

15 -3 -12 558.426 66.8117 8

-12 15 -912032.20 1033.44 3

-12 15 -912085.70 1021.05 14

15 -3 -911789.60 1007.29 8

-3 -12 -913616.20 1022.76 8

-3 -12 -913904.90 1017.67 1

-12 15 -912032.70 1022.02 11

-12 15 -912532.70 1028.42 2

12 -15 911020.40 1075.64 10

-15 3 910774.70 1114.18 10

-3 -12 -912609.60 1014.68 6

-12 15 -6 325.562 57.7134 3

-3 -12 -6 162.835 54.0407 8

-12 15 -6 425.744 61.5793 2

-3 -12 -6 614.468 60.2109 1

-15 3 6 89.0025 171.199 10

12 -15 6 82.1292 94.3974 10

-3 -12 -6 264.356 50.7826 6

-12 15 -6 295.598 172.977 4

12 -15 351045.30 4352.13 10

-15 3 359241.90 4417.04 10

3 12 357372.20 4389.30 9

-12 15 -354159.20 4719.96 4

3 12 015832.50 1359.62 9

-12 15 016068.30 1552.79 4

-3 -12 314145.20 1164.53 10

3 12 -312982.00 1224.91 9

-12 15 313020.10 1228.88 4

-3 -12 630680.60 2134.72 10

-12 15 627423.90 2086.18 10

-15 3 -622054.90 2013.21 1

3 12 -624660.50 2135.82 9

12 -15 -626115.60 2016.03 8

-12 15 627932.00 2120.70 4

12 -15 -625152.00 2018.79 6

3 12 -625247.90 2015.80 2

3 12 -632195.30 2183.87 7

3 12 -9 338.554 56.8502 14

-15 3 -9 264.735 54.9852 14

-12 15 9 167.069 81.6981 10

-3 -12 9 267.393 142.917 10

3 12 -9 231.514 93.7263 9

12 -15 -9 202.294 58.737 8

3 12 -9 288.313 56.0548 2

-15 3 -9 370.039 63.2079 11

3 12 -9 154.178 142.278 7

12 -15 -9 272.866 60.8217 6

-15 3 -9 292.635 93.7066 8

-15 3 -12 7628.73 703.841 8

3 12 -12 8715.31 809.057 7

-15 3 -12 9159.96 691.401 11

-3 -12 12 6527.20 751.14 10

15 -3 12 8042.52 773.429 10

12 -15 -12 8032.73 689.34 8

-15 3 -12 7296.04 709.397 2

3 12 -12 9101.13 690.691 2

-15 3 -12 7361.31 684.543 14

3 12 -12 9839.73 688.617 14

12 -15 -15-5.09064 34.0701 8

3 12 -15 30.6621 32.738 2

12 -15 -18 169.983 26.8948 8

3 12 -18 238.681 30.0548 2

3 12 -18 236.853 28.9892 3

-13 15 -16-1.39124 20.0643 11

-13 15 -16-19.7026 30.707 2

15 -2 -16 33.1058 25.762 2

-2 -13 -16-17.6162 28.8405 8

-15 2 13 160.183 85.6693 10

13 -15 13 194.544 86.2325 10

2 13 13 171.213 86.7017 10

-2 -13 -13 281.452 54.5937 6

-13 15 -13 216.696 71.5665 3

-13 15 -13 256.635 62.59 2

-13 15 -13 295.107 52.5228 11

-2 -13 -13 314.982 60.105 8

15 -2 -13 322.443 48.9671 8

-13 15 -10 -52.494 51.0466 11

15 -2 -10-15.9078 37.5433 8

13 -15 10 13.3481 85.1999 10

-15 2 10-56.7019 119.042 10

15 -2 -10-12.0706 78.053 9

-2 -13 -10 17.0502 53.1482 8

-13 15 -10 7.2105 72.7709 3

15 -2 -10 -25.744 71.7751 7

-2 -13 -10 14.1215 45.0697 6

-13 15 -10 99.6096 69.706 2

-15 2 7 2503.35 343.233 10

2 13 7 2334.78 253.693 4

-2 -13 -7 2259.69 206.957 8

-13 15 -7 2305.20 210.166 2

-2 -13 -7 2293.64 198.728 1

13 -15 7 2176.07 262.403 10

-2 -13 -7 2211.26 199.626 6

-13 15 -7 2166.43 206.086 3

-13 15 -7 1457.42 270.01 4

-2 -13 -4 9944.33 783.725 6

-15 2 4 9593.66 877.888 10

13 -15 4 8703.57 814.782 10

-13 15 -4 9573.01 1051.06 4

2 13 1 3168.20 339.007 9

2 13 -2 592.057 182.492 7

-13 15 2 255.821 218.714 4

-2 -13 2 308.638 107.273 10

-2 -13 524852.50 1824.04 10

15 -2 521893.80 1739.72 9

2 13 -523888.90 1897.96 9

13 -15 -521966.90 1725.15 8

2 13 -521576.00 1726.07 2

-13 15 523196.60 1839.21 4

2 13 -527763.10 1916.88 7

-13 15 515771.10 1760.85 10

13 -15 -520992.40 1727.42 6

-13 15 8 865.007 152.262 10

-2 -13 8 1203.09 212.23 10

2 13 -8 1002.54 186.997 9

-15 2 -8 857.867 112.154 1

13 -15 -8 1100.25 114.446 6

-15 2 -8 1172.93 120.752 11

2 13 -8 1086.09 109.946 2

-15 2 -8 966.898 130.695 8

13 -15 -8 1068.01 112.27 8

-2 -13 11 1228.24 215.605 10

13 -15 -11 1338.67 140.352 6

13 -15 -11 1410.86 143.08 8

-15 2 -11 1302.68 168.914 8

2 13 -11 1495.39 140.655 2

-15 2 -11 880.222 154.944 2

2 13 -11 1639.28 139.406 14

-15 2 -11 1386.49 138.282 14

-15 2 -11 1567.50 145.824 11

2 13 -11 1538.93 254.446 7

2 13 -14 167.155 42.8997 2

2 13 -14 148.102 43.2173 14

-15 2 -14 197.944 46.143 11

13 -15 -14 184.335 46.3149 8

2 13 -17 1.64742 23.389 2

2 13 -17 1.69078 21.9364 3

13 -15 -17 1.48153 21.2563 8

-14 15 -17 45.6332 24.3107 2

-14 15 -17 26.9638 15.2324 11

-1 -14 -17 41.16 22.8694 8

15 -1 -17 55.9533 20.5386 2

-14 15 -14 11.7262 44.8931 2

-1 -14 -14 16.2814 40.3891 8

-14 15 -14 111.835 37.9265 11

-15 1 11 256.829 124.843 10

14 -15 11 349.05 112.634 10

-14 15 -11 405.934 97.982 3

-1 -14 -11 713.703 86.6708 8

-14 15 -11 594.517 100.211 2

15 -1 -11 628.781 124.606 7

15 -1 -11 456.967 65.287 8

-1 -14 -11 681.047 78.9223 6

-14 15 -11 512.584 80.1431 11

-1 -14 -8 187.719 51.219 6

-1 -14 -8 238.517 60.4682 8

-14 15 -8 123.734 51.4969 3

-14 15 -8 184.505 63.2291 2

-1 -14 -8 124.061 45.1296 1

-15 1 8 267.46 149.551 10

14 -15 8 154.908 91.6499 10

-14 15 -8 199.829 61.5923 11

-15 1 520888.10 1680.51 10

1 14 520147.50 1631.76 4

-1 -14 -520526.00 1554.21 8

-14 15 -518059.60 1552.54 2

-1 -14 -519491.40 1550.25 1

-14 15 -517625.30 1729.59 4

-1 -14 -520353.30 1552.90 6

14 -15 516792.60 1595.05 10

-14 15 -518489.50 1552.69 3

1 14 2 1540.58 183.063 7

1 14 2 1888.05 222.282 9

-14 15 -2 2176.07 495.829 4

-15 1 2 1706.79 235.22 10

1 14 -132254.00 2642.35 7

1 14 -130299.60 2605.10 9

-14 15 134153.70 2728.45 4

-1 -14 130510.10 2560.69 10

1 14 -443501.60 3511.50 7

15 -1 439953.30 3355.42 9

1 14 -444499.00 3522.21 9

-1 -14 442758.20 3400.25 10

-14 15 441873.50 3448.72 4

1 14 -7 977.309 189.688 4

14 -15 -7 1064.35 106.001 6

-1 -14 7 1081.35 193.396 10

-14 15 7 960.874 162.539 10

1 14 -7 936.833 216.037 9

14 -15 -7 1080.31 106.46 8

-14 15 7 1156.96 181.151 4

-15 1 -7 845.777 102.199 1

1 14 -7 939.529 103.146 2

1 14 -10 128.186 39.5371 11

-1 -14 10 145.661 118.343 10

14 -15 -10 115.413 50.6969 8

-15 1 -10 23.6695 83.3719 8

1 14 -10 146.379 51.9524 2

-15 1 -10 146.911 48.7276 14

1 14 -10 195.047 47.3758 14

14 -15 -10 161.483 51.6441 6

-15 1 -10 197.661 54.237 11

1 14 -13 -19.422 96.6354 7

1 14 -13 100.855 46.707 2

-15 1 -13 193.357 49.1351 11

-15 1 -13 -54.083 53.562 14

1 14 -13 123.281 42.7006 14

-1 -14 13 41.7655 75.1483 10

15 -1 13 62.0554 80.5465 10

14 -15 -13 125.206 47.8646 8

14 -15 -16 3.84271 25.7929 8

1 14 -16-14.8033 26.8249 2

1 14 -16 37.4134 28.468 3

-15 15 -15 118.471 45.5172 2

0 -15 -15 215.78 41.8021 8

-15 15 -15 166.342 34.197 11

15 0 -15 203.732 36.6479 2

-15 15 -12 3.67086 50.2127 14

-15 15 -12 99.5202 48.5028 11

0 15 12 180.676 81.9175 10

-15 0 12 76.8146 84.3037 10

15 -15 12 36.831 69.7843 10

0 -15 -12 70.9215 45.1622 6

15 0 -12 42.2179 32.5589 8

0 -15 -12 111.128 53.1542 8

15 0 -12 12.2261 62.1946 7

-15 15 -12 58.4553 56.9722 2

-15 15 -12 25.4074 76.0882 3

15 -15 9 1863.48 254.393 10

0 -15 -9 2363.77 207.482 6

-15 0 9 2284.66 327.849 10

15 0 -9 2825.34 279.544 9

0 -15 -9 2842.36 210.612 1

0 -15 -9 2617.17 214.635 8

-15 15 -9 2100.73 220.22 2

-15 15 -9 1841.02 213.734 3

-15 15 -9 1958.68 212.422 11

-15 15 -9 1891.56 209.327 14

0 15 610600.90 912.295 4

0 -15 -611309.10 844.177 8

-15 15 -610365.50 842.766 2

0 -15 -6 9264.31 835.733 1

-15 15 -6 8935.69 960.428 4

0 -15 -610262.10 839.768 6

15 -15 6 9652.47 895.238 10

-15 15 -610646.00 841.448 3

-15 15 -373169.00 5422.50 4

-15 0 377824.60 5196.96 10

15 -15 359201.20 5100.65 10

0 15 034392.90 3028.31 9

-15 15 041757.40 3222.39 4

0 15 037685.00 3039.20 7

-15 15 386289.60 6962.05 4

0 15 -396451.20 7040.10 7

15 0 379750.90 6863.58 9

0 -15 386306.20 6888.26 10

0 15 -394784.40 7010.62 9

0 15 -6 297.308 159.81 4

15 -15 -6 390.088 61.1107 6

0 -15 6 209.534 119.645 10

-15 15 6 177.787 85.2252 10

0 15 -6 227.564 174.245 9

15 -15 -6 324.872 55.3753 8

-15 15 6 477.176 140.901 4

0 15 -6 270.64 217.718 7

-15 0 -6 551.036 60.9754 1

0 15 -6 357.984 59.1877 2

-15 0 -9 192.459 55.1046 1

0 15 -9 195.465 46.5149 11

0 15 -9 188.008 105.617 9

15 -15 -9 129.163 48.7343 8

-15 0 -9 79.7908 82.9599 8

0 15 -9 246.341 56.7987 14

0 -15 9 130.431 119.741 10

15 -15 -9 128.937 47.8698 6

0 15 -9 188.269 50.8694 2

-15 0 -9 179.032 55.6097 11

0 15 -12 526.631 69.3734 14

-15 0 -12 305.452 69.9957 14

15 -15 -12 550.884 75.6414 8

-15 0 -12 685.458 78.3352 11

0 15 -12 717.124 79.0898 2

0 -15 12 402.423 120.764 10

0 15 -12 378.487 149.893 7

-15 0 -12 531.215 100.553 8

0 15 -15 38.2184 33.6307 2

15 -15 -15 37.2746 31.2655 8

0 15 -15 83.3948 33.2854 14

0 15 -18 42.0631 18.0417 2

-1 16 -17 17.2133 22.2259 2

-1 16 -17 14.1171 22.0406 3

16 -15 -17-1.22164 18.8581 8

-1 16 -14-20.6784 38.4683 2

16 -15 -14 34.8349 34.2304 8

-1 16 -14 27.7107 34.1744 14

-1 16 -11 762.08 83.1121 2

-1 16 -11 526.748 172.072 7

-1 16 -11 643.605 72.5208 11

-15 -1 -11 580.88 80.5158 11

-1 16 -11 549.864 72.6121 14

-15 -1 -11 351.969 69.5463 14

16 -15 -11 541.742 77.1167 8

-15 -1 -11 586.476 115.583 8

1 -16 11 574.619 147.842 10

-15 -1 -8 521.927 81.8346 11

-1 16 -8 410.208 75.2896 14

1 -16 8 443.912 165.232 10

-1 16 -8 406.022 148.771 9

16 -15 -8 562.098 76.2032 8

-1 16 -8 678.18 79.8609 2

-1 16 -8 450.997 127.493 4

-15 -1 -8 649.739 78.5219 1

16 -15 -8 605.645 75.4484 6

-1 16 -5 599.613 238.129 9

15 1 5 382.524 72.165 9

16 -15 -5 475.371 57.3602 8

16 -15 -5 345.067 55.3466 6

-1 16 -5 290.636 54.6107 2

-15 -1 -5 292.495 52.4663 1

1 -16 5 361.362 122.052 10

-1 16 -5 270.468 208.309 4

-1 16 -5 636.769 277.97 7

-16 15 5 349.756 153.013 4

-16 15 2 4034.79 520.068 4

-1 16 -2 4691.08 531.677 7

15 1 2 4188.10 408.995 9

1 -16 2 4775.85 422.005 10

-1 16 141621.60 3425.79 9

-1 16 141886.00 3390.69 7

-15 -1 422963.70 1736.35 10

-16 15 -420737.60 1860.62 4

16 -15 416846.40 1646.49 10

-1 16 422207.90 1724.55 4

1 -16 -420525.10 1614.88 8

1 -16 -7 989.092 100.626 1

-16 15 -7 970.779 107.047 14

16 -15 7 799.553 148.037 10

-15 -1 7 964.48 242.995 10

15 1 -7 1137.62 174.464 9

1 -16 -7 929.039 105.89 8

-16 15 -7 1053.42 110.769 2

-1 16 7 795.275 161.534 4

-16 15 -7 580.566 177.302 4

-16 15 -7 1010.38 104.523 3

-16 15 -10 18.1009 48.7594 11

16 -15 10 50.6675 77.6347 10

-15 -1 10-20.3132 113.294 10

15 1 -10-12.0222 77.7799 9

1 -16 -10 62.2414 50.1684 8

-16 15 -10 4.36885 51.8374 14

-16 15 -10 46.2548 73.0959 2

1 -16 -10 58.2728 47.3195 6

-16 15 -10 32.5582 64.3676 3

15 1 -10 43.5588 75.213 7

1 -16 -13 77.7508 47.1483 8

-1 16 13-10.3096 57.6033 10

-15 -1 13 13.6309 67.7876 10

16 -15 13-56.6644 65.4377 10

1 -16 -13 40.0022 41.9465 6

-16 15 -13 138.629 50.0632 14

-16 15 -13 31.5661 56.2047 2

-16 15 -13 80.4469 42.1739 11

-16 15 -16-61.1392 33.3368 14

15 1 -16-27.0491 24.2542 2

1 -16 -16 1.96593 26.2645 8

-16 15 -16 1.41719 21.006 11

-2 16 -18 59.6593 19.7889 2

16 -14 -15-20.3064 30.1905 8

-2 16 -15 45.1266 34.1087 3

-2 16 -15 18.2878 35.0621 2

-2 16 -15-24.7929 32.5142 14

-2 16 -12 68.5503 46.1228 2

16 -14 -12 75.8849 45.1554 8

-14 -2 -12 98.432 67.5615 8

2 -16 12 153.488 92.7774 10

-2 16 -12 49.3137 33.5026 11

-2 16 -12 67.988 39.1695 14

-2 16 -9 141.57 44.0396 11

-14 -2 -9 56.0568 51.8788 1

-2 16 -9 141.475 51.1019 14

-16 14 9 222.367 97.522 10

2 -16 9 235.007 121.66 10

-14 -2 -9 74.4717 82.2563 8

16 -14 -9 84.9982 44.1021 8

-14 -2 -9 174.084 54.5688 11

-2 16 -9 285.394 177.059 7

-2 16 -9 66.8337 49.357 2

-16 14 6 3455.59 543.722 10

2 -16 6 7594.28 603.33 10

-2 16 -6 6771.23 694.421 4

-2 16 -6 5319.20 699.787 9

16 -14 -6 6104.52 508.473 8

-2 16 -6 6547.87 513.031 2

-16 14 6 7368.58 616.25 4

-14 -2 -6 6474.77 510.046 1

-2 16 -6 9355.05 845.417 7

16 -14 -6 5959.04 509.466 6

14 2 3 2444.43 275.263 9

-2 16 -3 3262.24 414.01 9

-16 14 3 3244.76 391.966 4

-2 16 -3 2289.87 484.827 4

-2 16 -3 3401.70 476.79 7

2 -16 3 3140.68 306.009 10

-16 14 045602.50 3301.10 4

-2 16 037320.50 3095.86 7

-2 16 037669.60 3121.07 9

14 2 033901.20 3090.96 9

-2 16 047771.70 3305.95 4

-16 14 -3 9221.54 1072.65 4

16 -14 3 9583.82 871.221 10

-14 -2 312845.30 959.284 10

2 -16 -310274.70 840.264 6

-2 16 311234.80 961.963 4

16 -14 6 2530.24 293.302 10

2 -16 -6 2516.98 233.79 1

14 2 -6 3388.53 326.834 9

-14 -2 6 3141.74 385.924 10

-2 16 6 2562.37 313.561 4

-16 14 -6 2659.83 238.236 2

-16 14 -6 3009.36 372.376 4

-16 14 -6 2892.59 238.579 3

2 -16 -6 2765.31 239.455 8

-16 14 -9 381.097 80.0614 11

16 -14 9 521.107 121.894 10

-14 -2 9 316.472 150.715 10

14 2 -9 595.56 127.94 9

2 -16 -9 690.316 82.0146 8

-16 14 -9 407.377 75.7559 14

14 2 -9 521.587 140.599 7

2 -16 -9 586.948 78.5454 6

-16 14 -9 310.535 68.6988 3

-16 14 -9 435.242 87.2244 2

-16 14 -12 268.396 61.7107 14

2 -16 -12 407.997 64.8057 6

-16 14 -12 227.96 89.2933 3

-16 14 -12 354.767 80.015 2

2 -16 -12 390.581 67.4209 8

-2 16 12 267.842 88.5845 10

-14 -2 12 78.194 87.8421 10

16 -14 12 293.218 97.9708 10

-16 14 -12 379.209 64.9817 11

14 2 -12 373.154 99.8344 7

-16 14 -15 171.826 45.1824 14

-16 14 -15 90.2962 41.644 2

-16 14 -15 112.221 33.9783 11

14 2 -15 175.406 34.6954 2

2 -16 -15 90.1517 37.6746 8

16 -13 -16 14.3708 23.3136 8

-3 16 -16 8.75072 26.0323 3

-3 16 -16 11.0668 30.1499 2

-3 16 -16 -5.321 28.6349 14

-3 16 -13 65.004 45.9742 2

-3 16 -13 148.293 42.5129 14

3 -16 13 150.86 93.6178 10

13 3 13 179.054 107.032 10

-3 16 -13 104.386 35.5508 11

16 -13 -13 125.005 46.2201 8

-13 -3 -13 186.457 65.0748 8

-3 16 -10 6943.26 565.879 11

-13 -3 -10 7596.90 572.907 11

-3 16 -10 7363.21 570.487 2

16 -13 -10 6932.46 566.157 8

-13 -3 -10 7832.86 600.105 8

-3 16 -10 8893.33 796.057 7

-16 13 10 5696.57 621.939 10

3 -16 10 6549.18 655.187 10

-13 -3 -10 4945.21 559.70 14

-3 16 -10 7867.26 570.158 14

-3 16 -7 1153.15 302.69 9

16 -13 -7 1539.66 149.129 8

-13 -3 -7 1397.77 148.809 1

16 -13 -7 1763.36 152.721 6

3 -16 7 1510.17 236.719 10

-16 13 7 1568.73 247.182 10

-3 16 -7 2092.30 292.724 4

-16 13 7 1750.58 228.088 4

-3 16 -7 1612.85 154.307 2

-3 16 -450436.80 3198.59 9

13 3 447169.20 2958.23 9

-3 16 -443871.40 2920.59 2

3 -16 447629.70 2993.19 10

-16 13 445397.10 3052.14 4

-3 16 -445285.50 3311.94 4

13 3 1 1848.82 215.271 9

3 -16 1 1512.76 195.411 10

-3 16 -1 1957.39 250.313 7

-16 13 1 1893.55 333.973 4

13 3 -2 1386.31 196.91 9

-13 -3 2 867.691 191.463 10

-3 16 2 1392.68 140.559 7

-3 16 2 1226.26 250.115 4

-16 13 -2 1115.67 350.59 4

13 3 -5 122.786 102.329 9

3 -16 -5 166.949 42.7802 8

-3 16 5 120.564 124.238 4

-13 -3 5 75.9323 129.257 10

16 -13 5 56.40 85.5836 10

3 -16 -5 106.046 41.4059 6

-16 13 -5 24.5403 164.961 4

-16 13 -8 137.484 60.0739 11

3 -16 -8 105.36 48.7307 1

16 -13 8 315.035 103.443 10

-13 -3 8 118.05 147.173 10

13 3 -8 150.455 89.8131 9

-16 13 -8 157.124 83.2249 4

-16 13 -8 163.326 45.259 3

-16 13 -8 137.901 55.275 14

-16 13 -8 264.211 60.056 2

3 -16 -8 84.5057 53.2656 8

-16 13 -11 36.8186 48.7337 11

-3 16 11 116.593 76.8632 10

-13 -3 11 53.247 99.3626 10

16 -13 11 217.243 95.8251 10

-16 13 -11 55.4551 69.038 2

3 -16 -11 121.279 53.1897 8

-16 13 -11 108.984 64.8827 3

-16 13 -11 140.546 55.5549 14

13 3 -11 225.413 100.024 7

3 -16 -11 146.486 50.2258 6

13 3 -14 578.417 67.9959 2

-16 13 -14 517.639 68.7886 11

3 -16 -14 576.044 73.8272 8

-16 13 -14 772.131 80.6177 14

-16 13 -14 483.239 80.7337 2

3 -16 -17-1.49158 20.5124 8

-16 13 -17 35.2554 16.612 11

13 3 -17-5.16353 20.1618 2

16 -12 -17 231.61 32.3008 8

-4 16 -17 261.514 38.451 2

-4 16 -17 253.371 36.1088 3

-12 -4 -14 2136.93 178.555 8

16 -12 -14 1818.09 165.064 8

-4 16 -14 1862.87 169.60 2

-4 16 -14 1725.74 162.908 14

-4 16 -14 1614.21 166.083 3

-4 16 -11 270.55 54.376 11

4 -16 11 276.239 118.619 10

-16 12 11 277.995 96.8723 10

-12 -4 -11 210.224 45.8778 6

-4 16 -11 313.499 58.4208 14

-12 -4 -11 355.222 76.3664 8

16 -12 -11 331.295 56.9891 8

-4 16 -11 267.272 61.3517 2

-4 16 -11 234.283 156.17 7

-12 -4 -813036.70 912.228 11

16 -12 -810313.10 898.389 8

-12 -4 -810519.30 924.798 8

-4 16 -811005.40 903.934 2

-12 -4 -810559.00 897.052 6

-16 12 8 9540.99 991.911 10

4 -16 811224.90 995.901 10

-4 16 -811016.60 904.704 14

-12 -4 -811860.40 903.122 1

-4 16 -8 9913.64 990.547 4

-4 16 -522782.20 1755.09 7

-4 16 -516879.70 1340.09 2

-16 12 518500.10 1440.47 4

-4 16 -516421.80 1582.63 9

12 4 516123.90 1358.63 9

-16 12 512274.90 1372.24 10

-12 -4 -517139.50 1336.45 1

-4 16 -520163.40 1665.13 4

4 -16 517056.30 1402.16 10

12 4 225146.40 2181.55 9

-4 16 -226898.60 2261.70 9

4 -16 226226.30 2175.64 10

-4 16 -228482.10 2266.07 7

-4 16 -228707.10 2561.36 4

-16 12 228703.00 2285.60 4

12 4 -1 3697.65 375.578 9

-4 16 1 3508.12 332.337 7

-4 16 1 3485.00 458.322 4

4 -16 -4 579.703 70.2962 6

-4 16 4 650.748 161.109 4

-16 12 -4 297.403 197.368 4

12 4 -4 663.618 152.189 9

16 -12 7 4283.53 441.175 10

-16 12 -7 4039.57 463.339 4

-12 -4 7 4830.36 523.306 10

12 4 -7 4585.73 449.663 9

-4 16 7 4482.46 457.589 4

-16 12 -7 4468.65 383.418 2

4 -16 -7 4778.17 384.661 8

-16 12 -7 4122.52 377.494 3

4 -16 -7 4994.87 382.942 1

4 -16 -7 4460.15 383.092 6

16 -12 10 422.136 115.814 10

-12 -4 10 688.775 171.145 10

-4 16 10 447.282 115.565 10

12 4 -10 457.73 113.331 9

12 4 -10 547.094 137.698 7

-16 12 -10 507.874 77.5904 11

4 -16 -10 604.049 81.1474 8

-16 12 -10 449.67 94.7283 2

-16 12 -10 363.308 77.6198 14

-16 12 -13 1032.03 113.169 14

-4 16 13 749.991 136.289 10

-12 -4 13 785.941 159.903 10

16 -12 13 737.59 146.845 10

4 -16 -13 1058.85 113.511 6

-16 12 -13 1093.45 112.013 11

12 4 -13 1411.68 112.636 2

-16 12 -13 900.891 127.133 2

4 -16 -13 1114.36 116.332 8

12 4 -16 347.274 41.9116 2

4 -16 -16 257.517 42.4333 8

-16 12 -16 277.775 38.1307 11

-16 12 -16 230.143 44.916 14

-5 16 -18 282.412 35.4611 2

-5 16 -15-2.80533 31.0141 3

-11 -5 -15-11.6691 36.6233 8

16 -11 -15 13.8282 31.6587 8

-5 16 -15 11.15 36.2798 2

-5 16 -15-17.5991 34.3191 14

16 -11 -12 101.069 42.3122 8

-11 -5 -12 134.868 59.7074 8

5 -16 12 59.5344 81.9003 10

-5 16 -12 123.565 45.5331 14

-5 16 -12 140.619 48.7913 2

-5 16 -12 42.3032 36.4735 11

5 -16 9 1799.97 267.298 10

-16 11 9 1676.17 263.515 10

-5 16 -9 2984.17 482.676 7

16 -11 -9 2047.33 192.358 8

-11 -5 -9 2603.66 223.273 8

-5 16 -9 1930.62 198.774 2

-5 16 -9 2024.71 200.171 14

-11 -5 -9 2062.14 192.836 6

-5 16 -9 2113.41 198.987 11

-11 -5 -9 2575.62 204.934 11

16 -11 -6 271.111 50.5315 8

-16 11 6 335.152 132.892 4

-11 -5 -6 560.417 61.6464 1

-16 11 6 72.9524 112.172 10

5 -16 6 369.423 114.592 10

-5 16 -6 258.312 191.266 4

-5 16 -6 530.778 406.062 7

-5 16 -6 393.479 61.3842 2

-5 16 -316491.20 1168.31 7

5 -16 314013.10 1002.12 10

-16 11 315570.60 1106.15 4

-5 16 -312612.00 1513.09 4

11 5 313093.80 996.596 9

-5 16 -314009.10 1125.59 9

-5 16 0 1364.71 173.33 7

-16 11 0 1126.14 286.251 4

-5 16 0 1666.06 355.917 4

11 5 0 1316.27 187.012 9

-5 16 325309.20 2130.35 4

-16 11 -324928.80 2205.98 4

11 5 -6 2951.02 326.193 9

5 -16 -6 2741.65 257.737 8

-5 16 6 3099.93 347.321 4

5 -16 -6 3414.82 259.814 1

5 -16 -6 2960.90 259.217 6

-16 11 -6 2182.21 339.572 4

11 5 -913734.30 1131.76 7

-11 -5 911826.80 1146.87 10

-5 16 911781.50 1083.04 10

16 -11 911557.50 1092.71 10

-16 11 -913161.30 1041.81 14

11 5 -914318.30 1100.04 9

-16 11 -913187.10 1041.44 11

-16 11 -911680.80 1044.24 2

-16 11 -12 332.643 89.9615 2

11 5 -12 484.695 122.191 7

-16 11 -12 535.156 74.9654 14

-11 -5 12 252.43 116.953 10

-5 16 12 466.873 102.84 10

16 -11 12 574.132 126.513 10

-16 11 -12 526.044 71.1223 11

11 5 -15 78.4139 32.4837 2

-16 11 -15-7.13112 40.1412 2

-16 11 -15 49.0058 37.4985 14

-16 11 -15 70.0319 32.5504 11

5 -16 -18 44.0477 17.6509 8

-6 16 -16-7.71269 33.0332 14

16 -10 -16 14.4038 24.8633 8

-6 16 -16 54.2865 28.3528 3

6 -16 13 13.5061 75.4956 10

10 6 13 81.8052 95.9772 10

16 -10 -13 41.2242 37.8383 8

-10 -6 -13 103.53 50.1021 8

-6 16 -13 58.0638 33.6649 11

-6 16 -13 89.1683 45.2822 14

-6 16 -13 82.8591 46.7417 2

16 -10 -10 1296.29 131.521 8

-10 -6 -10 1551.39 153.434 8

-6 16 -10 1152.62 134.473 11

-16 10 10 1010.42 189.079 10

6 -16 10 1137.84 205.465 10

-6 16 -10 1512.56 346.539 7

-10 -6 -10 1616.47 136.204 6

-6 16 -10 1537.23 144.209 2

-6 16 -10 1159.52 137.797 14

6 -16 7 159.512 104.592 10

-16 10 7 245.558 147.359 10

-10 -6 -7 352.23 72.1327 8

16 -10 -7 130.971 43.7476 8

-6 16 -7 237.728 53.8176 2

-10 -6 -7 320.013 46.9472 6

-6 16 -7 169.482 140.807 4

-6 16 -4 1211.58 591.831 4

-6 16 -4 2136.31 366.203 7

-16 10 4 1370.22 186.998 10

6 -16 4 2165.28 210.776 10

-16 10 4 1428.23 245.528 4

-6 16 -4 1800.01 158.625 3

-6 16 -4 1585.85 157.048 2

10 6 4 1865.27 191.285 9

10 6 137409.10 3061.55 9

6 -16 137041.40 3031.81 10

-16 10 140763.80 3174.61 4

-6 16 -137231.30 3068.71 7

10 6 -2 411.046 128.77 9

-16 10 -2 610.779 245.87 4

-6 16 2 506.66 183.071 4

-10 -6 2 532.088 146.487 10

6 -16 -527316.60 2248.43 6

-6 16 530816.50 2358.19 4

10 6 -530719.90 2332.38 9

-16 10 -527050.80 2372.32 4

10 6 -8 2950.26 302.147 9

-16 10 -8 2795.35 249.294 14

-16 10 -8 2564.03 246.876 11

-16 10 -8 2342.94 241.569 2

6 -16 -8 2851.77 243.209 6

10 6 -8 3332.12 341.318 7

-16 10 -11 3017.65 282.543 14

-16 10 -11 3533.86 285.862 11

16 -10 11 2893.66 337.84 10

-6 16 11 3191.23 329.772 10

-10 -6 11 3152.73 373.775 10

10 6 -11 3368.85 371.628 7

-16 10 -11 2995.81 302.039 2

-16 10 -14 79.9525 38.5345 11

-16 10 -14 171.555 47.2899 14

10 6 -14 18.2735 32.0492 2

-16 10 -14 37.9326 51.2777 2

10 6 -17 112.834 26.1153 2

-7 16 -14 149.568 45.7318 3

-7 16 -14 54.632 29.6877 11

-7 16 -14 160.619 45.5386 14

-9 -7 -14 70.6639 43.5764 8

16 -9 -14 79.296 36.7122 8

-7 16 -11 1669.95 161.808 14

16 -9 -11 1579.64 153.185 8

-9 -7 -11 1771.36 170.724 8

-9 -7 -11 1690.70 156.60 6

-7 16 -11 1665.97 159.318 11

-16 9 11 1398.61 214.11 10

7 -16 11 1354.66 217.528 10

-7 16 -8 928.145 119.05 11

-9 -7 -8 1351.60 132.304 8

16 -9 -8 1095.80 110.935 8

-7 16 -8 1096.70 120.884 2

-9 -7 -8 1199.72 115.434 6

7 -16 8 770.462 169.875 10

-16 9 8 1066.75 219.621 10

-7 16 -8 1129.39 121.118 14

-7 16 -5 1743.30 166.372 2

-7 16 -5 1706.84 444.761 7

9 7 5 1708.87 190.113 9

7 -16 5 1720.88 217.533 10

-7 16 -5 1390.55 432.151 4

-16 9 5 2592.20 283.563 4

-16 9 5 1852.76 242.96 10

-7 16 -212402.80 1079.46 7

-16 9 214354.70 1151.29 4

9 7 211763.10 1040.65 9

7 -16 211887.70 1017.69 10

-7 16 1 429.743 225.79 4

9 7 -1 308.976 109.912 9

-9 -7 1 338.91 125.809 10

-16 9 -4 8657.69 945.753 4

-9 -7 410311.60 917.875 10

9 7 -410300.80 903.665 9

-7 16 411000.90 943.761 4

-16 9 -7 865.457 97.0846 2

7 -16 -7 881.377 101.899 6

9 7 -7 1232.10 167.298 9

-7 16 7 1230.13 192.662 4

9 7 -10 732.446 177.063 7

-16 9 -10 819.166 96.9465 11

-16 9 -10 866.731 101.402 14

7 -16 -10 837.055 97.7647 6

9 7 -10 777.739 134.949 9

16 -9 10 829.166 155.67 10

-7 16 10 625.406 128.358 10

-16 9 -10 514.966 103.869 2

7 -16 -13 17.305 48.0008 8

9 7 -13 43.6339 38.1686 2

-16 9 -13 71.7284 63.1592 2

-9 -7 13-14.1228 76.0188 10

16 -9 13 13.0656 76.5636 10

-16 9 -13-13.0333 42.6039 11

-16 9 -13 82.596 49.4299 14

9 7 -13 29.804 70.7464 7

-16 9 -16 48.148 24.4952 11

7 -16 -16-4.17319 30.2472 8

9 7 -16-1.92891 25.8874 2

16 -8 -18 36.9931 15.4899 2

-8 16 -18 30.0226 19.3085 2

-8 16 -15 579.055 68.6298 3

-8 -8 -15 616.221 71.2303 8

16 -8 -15 528.118 62.2887 8

-8 16 -15 513.866 67.3496 14

-8 16 -15 449.248 55.9329 11

-16 8 12 1618.03 255.682 10

8 -16 12 1901.01 267.672 10

-8 16 -12 2212.78 212.735 11

-8 16 -12 2466.92 217.695 14

-8 -8 -12 2555.00 215.274 6

16 -8 -12 2431.62 212.392 8

-8 -8 -12 2719.65 225.015 8

16 -8 -9 1214.83 132.936 8

-8 -8 -9 1636.91 152.078 8

-8 -8 -9 1475.57 143.249 1

-8 16 -9 1588.02 149.913 14

-8 16 -9 1438.24 444.014 7

-8 -8 -9 1313.40 138.521 6

8 -16 9 1328.34 205.685 10

-16 8 9 1426.52 234.335 10

-8 16 -9 1493.51 147.331 11

-8 16 -6 696.956 327.262 7

-8 -8 -6 558.72 69.8082 6

8 -16 6 694.327 136.215 10

-16 8 6 972.353 208.629 10

-8 -8 -6 617.573 74.2492 1

-8 16 -6 618.124 231.064 4

-16 8 3 8609.11 858.916 10

-16 8 313655.20 976.49 4

8 8 310209.20 864.34 9

-8 16 -313620.50 1616.80 4

-8 16 -310141.00 934.179 7

8 -16 310233.90 851.678 10

8 8 023920.60 2011.89 9

-16 8 021153.90 2095.12 4

-16 8 -3 201.914 177.751 4

-8 -8 3 177.876 112.356 10

-8 16 3 120.546 170.803 4

-8 16 6 547.703 165.865 4

8 -16 -6 757.083 83.3631 6

-8 -8 6 674.278 174.556 10

8 -16 -6 658.185 78.5987 8

8 8 -6 670.475 138.59 9

8 8 -911299.00 884.809 9

8 -16 -9 9785.71 818.786 8

8 8 -911688.60 935.19 7

-16 8 -910522.60 826.216 14

8 -16 -910414.10 820.129 6

-16 8 -9 8729.18 825.166 2

-16 8 -910213.70 823.576 11

-8 -8 9 9307.19 926.489 10

-8 16 9 8691.42 855.934 10

8 -16 -12 1719.38 163.533 8

-16 8 -12 1636.03 161.577 11

8 -16 -12 1635.26 162.379 6

-16 8 -12 1031.04 173.544 2

8 8 -12 1746.71 159.501 2

8 8 -12 1367.66 228.937 7

16 -8 12 1628.82 225.398 10

-8 16 12 1286.80 188.182 10

8 8 -12 1952.75 160.918 14

-16 8 -12 1930.92 164.491 14

-16 8 -15 126.688 33.1642 11

-16 8 -15 61.3674 41.7302 14

8 8 -15 91.8259 32.8349 2

8 -16 -15 101.264 36.424 8

8 8 -18 31.0831 17.4651 3

8 -16 -18 9.56318 16.5048 8

16 -7 -16-10.0829 22.9405 8

-7 -9 -16-24.4146 28.4699 8

-9 16 -16 -33.724 37.2902 14

-9 16 -16-21.7733 32.02 2

-7 -9 -13 2005.22 179.735 8

16 -7 -13 1869.40 169.037 8

-9 16 -13 2151.08 175.423 14

-16 7 13 1240.87 207.266 10

9 -16 13 1280.74 212.619 10

7 9 13 1877.97 246.457 10

-9 16 -13 1744.33 180.661 3

-9 16 -13 1931.84 169.849 11

16 -7 -10 374.304 58.0962 8

-7 -9 -10 374.367 71.9184 8

-7 -9 -10 395.227 63.6812 6

-9 16 -10 398.893 68.2686 11

-9 16 -10 370.17 74.8266 14

-16 7 10 357.317 129.514 10

9 -16 10 478.048 127.503 10

-9 16 -7 3605.56 468.138 4

9 -16 7 4115.91 428.781 10

-16 7 7 4908.23 518.847 10

-7 -9 -7 4066.66 355.461 1

-7 -9 -7 4350.85 355.309 6

16 -7 -7 3879.31 350.494 8

-7 -9 -7 4927.87 368.535 8

7 9 4 4079.70 392.90 9

-9 16 -4 3901.24 491.875 7

-9 16 -4 5870.57 866.283 4

-16 7 4 5350.11 489.935 4

-16 7 4 3747.94 415.17 10

9 -16 4 4324.91 393.922 10

-16 7 1 9049.30 900.241 4

7 9 1 9165.72 804.45 9

-7 -9 216824.70 1326.20 10

-9 16 214088.70 1386.63 4

-16 7 -214334.40 1404.72 4

-9 16 5 5626.98 563.95 10

-9 16 5 7201.57 661.33 4

9 -16 -5 7112.95 536.614 6

9 -16 -5 6199.75 532.855 8

7 9 -5 5560.01 607.948 9

-7 -9 5 7652.99 646.359 10

7 9 -8 395.055 112.295 9

9 -16 -8 237.073 54.232 8

-16 7 -8 172.993 55.094 11

9 -16 -8 197.894 54.3368 6

-7 -9 8 226.318 142.184 10

-9 16 8 287.68 89.6459 10

7 9 -8 307.587 133.405 7

-16 7 -8 142.112 45.0817 2

7 9 -8 183.299 44.7252 2

7 9 -11 2385.18 173.407 2

-16 7 -11 1342.48 188.048 2

9 -16 -11 1703.35 169.683 8

7 9 -11 1984.34 266.409 7

-9 16 11 1250.30 194.083 10

9 -16 -11 2046.38 173.375 6

16 -7 11 1555.08 233.212 10

-7 -9 11 1385.89 244.375 10

-16 7 -11 1433.14 165.912 14

7 9 -11 1818.39 164.674 14

-16 7 -11 1905.47 172.121 11

-16 7 -14 59.7136 37.8897 11

9 -16 -14 115.026 43.994 8

-16 7 -14 22.5004 44.0216 14

7 9 -14 84.7356 38.673 2

-16 7 -14 54.6625 53.2548 2

9 -16 -17 25.0273 22.5958 8

7 9 -17 41.3831 23.2022 2

-10 16 -17 215.917 35.1709 2

-10 16 -17 123.668 34.1847 14

16 -6 -17 237.539 31.8631 2

-6 -10 -14 304.294 55.7554 8

16 -6 -14 275.857 45.9254 8

-10 16 -14 367.363 59.1621 14

-10 16 -14 173.105 49.9358 2

-10 16 -14 251.934 44.7807 11

-10 16 -14 212.89 56.1942 3

-16 6 11 6583.26 727.154 10

10 -16 11 6881.61 722.676 10

-6 -10 -11 8423.31 663.72 6

-10 16 -11 7510.35 670.412 2

-6 -10 -11 9481.22 673.094 8

16 -6 -11 7881.61 656.609 8

-10 16 -11 6822.17 679.61 3

-10 16 -11 8787.92 669.422 14

-10 16 -11 7853.48 664.697 11

-10 16 -8 2999.78 253.693 11

-10 16 -8 2628.34 248.659 2

16 -6 -8 2427.23 231.286 8

-6 -10 -8 2897.51 247.543 8

-6 -10 -8 2642.34 237.207 6

-10 16 -8 3277.71 251.633 14

10 -16 8 3007.53 320.728 10

-16 6 8 2504.39 354.386 10

-6 -10 -8 2415.85 236.651 1

-10 16 -8 2823.42 259.638 3

6 10 524944.70 2111.50 9

-10 16 -526103.60 2078.28 2

-10 16 -525213.00 2348.02 4

10 -16 526092.90 2124.27 10

-10 16 -531209.00 2302.13 7

-10 16 -525751.00 2077.99 3

-16 6 530801.10 2197.91 10

-6 -10 -525051.20 2071.80 6

6 10 235690.80 2682.75 9

10 -16 231595.80 2630.84 10

-16 6 230227.60 2673.95 10

-16 6 237468.60 2790.61 4

-10 16 153244.10 4109.77 4

-6 -10 150329.30 3960.41 10

6 10 -144906.80 3968.99 9

6 10 -420678.40 1841.54 9

-10 16 421945.00 1781.74 10

-6 -10 422406.10 1830.21 10

-10 16 421285.30 1870.59 4

6 10 -715862.30 1364.01 9

-10 16 717265.50 1379.60 4

10 -16 -715574.60 1288.50 6

-10 16 716621.00 1330.99 10

-6 -10 718042.80 1415.60 10

6 10 -716344.60 1285.41 2

10 -16 -714942.90 1285.41 8

10 -16 -10 546.414 80.4137 8

10 -16 -10 630.091 79.2215 6

6 10 -10 745.457 76.3488 2

-16 6 -10 178.686 77.6582 2

-16 6 -10 414.824 73.7954 14

6 10 -10 522.882 68.0236 14

6 10 -10 356.372 148.747 7

-16 6 -10 466.882 75.29 11

-6 -10 10 394.779 145.725 10

-10 16 10 488.531 105.474 10

10 -16 -13 111.179 47.8148 8

6 10 -13 82.2776 41.656 2

-16 6 -13 37.5416 58.8605 2

-16 6 -13 92.1798 50.0272 14

6 10 -13 50.4574 40.3538 14

16 -6 13 28.3252 81.7876 10

-6 -10 13-14.0944 77.3703 10

6 10 -13 84.2556 89.0965 7

-16 6 -13 28.6146 41.9146 11

10 -16 -16 458.878 57.9655 8

6 10 -16 546.987 59.4744 2

-11 16 -18 18.5636 17.8173 2

-11 16 -15 68.8009 43.8501 14

-11 16 -15 62.5368 38.2248 2

16 -5 -15 26.0702 27.4454 8

-5 -11 -15 93.2519 40.6341 8

-11 16 -15 42.9602 25.5309 11

-11 16 -12 6.96484 42.1433 11

-5 -11 -12 13.1119 42.62 6

-5 -11 -12 48.4945 47.6075 8

16 -5 -12 91.7876 36.8722 8

-16 5 12 14.1372 74.692 10

11 -16 12-13.2849 76.6386 10

-11 16 -12 36.9276 56.8222 2

-11 16 -12 57.869 62.5176 3

-11 16 -12 3.68759 56.0561 14

-5 -11 -9 1455.36 140.263 6

-16 5 9 896.387 219.706 10

11 -16 9 974.951 186.751 10

-11 16 -9 1581.89 154.293 2

-5 -11 -9 1558.41 149.435 8

16 -5 -9 1302.71 130.476 8

-11 16 -9 1416.11 164.616 3

-11 16 -9 1289.51 145.334 11

-5 -11 -9 1594.36 143.243 1

-11 16 -9 1407.77 147.655 14

-11 16 -618414.70 1494.21 2

-5 -11 -619399.50 1494.58 8

-11 16 -618663.30 1494.23 3

-11 16 -615947.10 1640.30 4

-5 -11 -618154.70 1487.83 1

11 -16 617897.80 1547.68 10

-16 5 622786.00 1661.66 10

-5 -11 -617927.90 1486.25 6

11 -16 311592.90 977.293 10

-16 5 311007.80 1021.64 10

5 11 312692.40 1011.50 9

-11 16 -314240.10 1556.25 4

5 11 037479.10 3236.86 9

-16 5 040902.20 3362.96 4

-11 16 045208.80 3484.58 4

-5 -11 3100008.0 7994.44 10

5 11 -395964.10 8027.59 9

-11 16 3102128.0 8071.09 4

-11 16 611800.30 917.745 10

-5 -11 613593.00 993.572 10

11 -16 -610763.30 867.69 6

5 11 -611154.80 863.963 2

5 11 -610146.60 958.062 9

11 -16 -610531.10 863.795 8

-11 16 611888.40 975.787 4

5 11 -612020.50 991.214 7

-16 5 -6 9295.84 861.905 1

5 11 -9 1219.71 212.501 7

-5 -11 9 787.879 193.44 10

5 11 -9 1251.36 177.517 9

11 -16 -9 1197.87 116.955 8

-16 5 -9 927.779 113.682 14

-16 5 -9 747.599 127.912 8

-11 16 9 1055.71 148.707 10

11 -16 -9 1181.40 118.968 6

-16 5 -9 1213.07 122.185 11

-16 5 -9 789.777 118.008 2

5 11 -9 1256.58 114.897 2

11 -16 -12 1005.24 117.007 8

11 -16 -12 1198.88 118.006 6

5 11 -12 1602.09 120.041 2

-16 5 -12 514.818 123.105 2

5 11 -12 1108.87 111.863 14

-16 5 -12 931.963 112.701 14

5 11 -12 1057.88 200.886 7

16 -5 12 1134.80 186.438 10

-5 -11 12 815.457 170.062 10

-16 5 -12 1049.60 115.595 11

5 11 -15 126.926 35.5016 2

-16 5 -15 139.022 35.471 11

11 -16 -15 55.5023 35.4315 8

11 -16 -18 228.356 29.4281 8

5 11 -18 235.035 30.2152 2

5 11 -18 256.435 30.5341 3

-12 16 -16-17.5102 36.6024 14

-12 16 -16 13.2776 18.6625 11

-12 16 -16-4.80009 29.8295 2

-4 -12 -16 30.3143 28.5453 8

-4 -12 -13 131.636 44.2931 6

-12 16 -13 63.7624 46.3031 2

-12 16 -13 219.348 53.8401 14

-4 -12 -13 85.9124 48.0499 8

16 -4 -13 173.442 39.5765 8

4 12 13 289.423 96.8885 10

-16 4 13 115.946 80.7192 10

12 -16 13-12.1886 69.9149 10

-12 16 -13 148.95 43.5094 11

-12 16 -13 177.765 65.0738 3

12 -16 10 1713.88 241.691 10

-16 4 10 1431.74 253.091 10

16 -4 -10 2173.65 245.20 9

-12 16 -10 1843.83 196.141 2

-4 -12 -10 2158.79 190.827 8

16 -4 -10 1822.90 229.924 7

16 -4 -10 1743.09 173.089 8

-12 16 -10 1697.68 205.35 3

-12 16 -10 2450.42 196.617 14

-4 -12 -10 1905.00 181.797 6

-12 16 -10 2128.73 192.216 11

-4 -12 -7 36.3708 37.8899 1

-4 -12 -7 33.3457 46.6101 8

-12 16 -7 40.9179 43.9282 2

-4 -12 -7 41.5701 35.3891 6

-12 16 -7-8.25331 49.7369 3

-12 16 -7 20.3385 105.338 4

4 12 7 142.719 76.9035 4

-16 4 7-25.4377 153.611 10

12 -16 7 128.199 95.7675 10

4 12 4 429.579 103.188 9

-12 16 -4 468.579 62.8551 3

-12 16 -4 694.534 69.5621 2

12 -16 4 422.198 88.8087 10

-16 4 4 270.991 135.021 10

-12 16 -4 688.882 389.858 4

-4 -12 -4 407.77 60.242 6

4 12 1 60.3407 89.3663 9

-4 -12 2 3979.10 399.144 10

-12 16 2 3772.71 506.238 4

-4 -12 5 432.841 140.69 10

-12 16 5 381.674 101.549 10

4 12 -5 604.381 170.814 7

-12 16 5 428.173 179.191 4

12 -16 -5 623.204 72.0104 6

16 -4 5 375.829 72.4247 9

12 -16 -5 566.326 65.8118 8

4 12 -5 549.468 179.664 9

-4 -12 8 155.819 128.232 10

-12 16 8 152.017 70.0665 10

4 12 -8 108.213 96.9444 9

12 -16 -8 120.725 48.2967 8

4 12 -8 208.021 78.4529 4

4 12 -8 207.68 143.25 7

-16 4 -8 232.984 57.8591 11

-16 4 -8 134.425 56.4233 8

12 -16 -8 214.148 55.6289 6

4 12 -8 155.936 43.8899 2

12 -16 -11 456.145 72.4561 8

-4 -12 11 207.001 115.936 10

-16 4 -11 159.948 81.8651 2

4 12 -11 537.276 71.2205 2

4 12 -11 537.966 66.3833 14

-16 4 -11 446.322 70.6411 14

12 -16 -11 455.914 75.7595 6

4 12 -11 394.274 139.009 7

-16 4 -11 608.872 75.7368 11

4 12 -14 288.176 47.0174 14

-16 4 -14 149.407 48.8732 14

4 12 -14 181.249 45.7731 2

12 -16 -14 111.273 44.9589 8

-16 4 -14 241.712 46.5123 11

4 12 -17 15.5948 22.9935 2

4 12 -17 54.4626 23.7078 3

12 -16 -17 32.5756 22.4322 8

-13 16 -17-5.25212 23.2372 2

-3 -13 -17-8.86762 20.8959 8

-13 16 -17-72.1573 32.5177 14

16 -3 -17 10.2221 19.2002 2

-13 16 -14 765.833 89.9714 2

-3 -13 -14 772.382 86.0824 8

-13 16 -14 722.909 86.1481 14

16 -3 -14 689.912 73.8491 8

-13 16 -14 592.086 75.9395 11

-13 16 -14 716.311 94.5525 3

-13 16 -11 6846.49 563.033 11

-3 -13 -11 7588.26 561.975 6

-16 3 11 5511.23 630.863 10

16 -3 -11 8264.01 626.468 9

-13 16 -11 7214.73 576.888 2

-3 -13 -11 7616.25 568.284 8

16 -3 -11 5493.57 601.576 7

16 -3 -11 6885.93 552.668 8

-13 16 -11 5074.87 578.719 3

13 -16 11 5087.10 607.039 10

-13 16 -11 6245.59 563.311 14

-3 -13 -8 634.33 77.928 6

-16 3 8 621.501 200.925 10

13 -16 8 704.065 140.784 10

-13 16 -8 582.761 93.6628 2

-3 -13 -8 718.936 88.3323 8

-13 16 -8 576.018 90.6433 3

-13 16 -8 416.194 118.684 4

-3 -13 -8 607.909 78.2313 1

-13 16 -8 779.47 92.3626 14

-13 16 -8 705.992 95.5116 11

-13 16 -515593.70 1275.82 2

-3 -13 -516191.60 1275.62 8

-3 -13 -516217.30 1273.07 6

-13 16 -511926.30 1452.06 4

-13 16 -515649.40 1275.43 3

-16 3 516315.10 1393.93 10

-3 -13 -516281.20 1272.48 1

13 -16 513032.70 1308.90 10

-13 16 -2 4767.56 846.673 4

-16 3 2 4967.40 494.161 10

3 13 2 5039.50 478.868 9

-13 16 1 297.882 263.763 4

-3 -13 1 229.148 107.405 10

3 13 -1 152.649 135.918 9

-3 -13 411401.90 1041.36 10

16 -3 411014.30 992.942 9

3 13 -413773.50 1138.01 9

-13 16 412981.20 1106.48 4

3 13 -413647.00 1113.82 7

-3 -13 7 396.848 137.40 10

-13 16 7 511.671 110.517 10

3 13 -7 337.396 136.979 9

13 -16 -7 537.388 67.5189 8

-13 16 7 396.613 136.564 4

3 13 -7 401.142 124.553 4

13 -16 -7 716.024 76.4338 6

3 13 -7 462.945 63.7834 2

-16 3 -7 345.036 63.6773 1

-16 3 -10 1896.59 160.797 11

-16 3 -10 1386.63 149.479 14

3 13 -10 1968.54 153.377 14

13 -16 -10 1441.55 154.291 8

3 13 -10 1594.61 153.072 2

13 -16 -10 1516.87 153.463 6

-3 -13 10 1279.38 231.307 10

3 13 -10 1755.88 263.157 7

-16 3 -10 1063.49 172.711 8

-16 3 -13 2409.45 254.201 14

3 13 -13 3170.50 255.679 14

-3 -13 13 2026.25 304.321 10

16 -3 13 2658.28 323.88 10

-16 3 -13 3251.85 259.286 11

3 13 -13 3391.02 261.234 2

3 13 -13 2640.43 342.299 7

13 -16 -13 2976.66 258.159 8

13 -16 -16 456.579 55.4888 8

3 13 -16 519.688 57.9905 2

-14 16 -15-36.8297 40.2157 2

-2 -14 -15 23.7528 34.3713 8

-14 16 -15 50.9923 27.7223 11

-2 -14 -12 23.1948 45.8526 6

-14 16 -12 103.565 76.6969 3

-2 -14 -12 31.8342 47.8387 8

16 -2 -12 89.5165 35.3611 8

-16 2 12 59.9587 86.5931 10

14 -16 12 12.5468 72.5446 10

2 14 12 52.181 76.6103 10

-14 16 -12 107.946 59.9256 2

-14 16 -12 116.704 47.4891 11

-2 -14 -9 827.714 101.522 6

-16 2 9 816.911 206.134 10

14 -16 9 781.037 152.85 10

16 -2 -9 916.098 156.258 9

-2 -14 -9 936.831 109.469 8

-14 16 -9 804.687 117.831 3

-2 -14 -9 869.181 99.4639 1

-14 16 -9 943.747 119.032 2

-14 16 -9 989.86 112.801 11

-16 2 6 4294.80 477.646 10

2 14 6 4055.16 394.474 4

-2 -14 -6 4378.90 330.602 8

-14 16 -6 3404.75 325.471 2

-2 -14 -6 3778.44 321.861 1

-2 -14 -6 4096.96 324.086 6

-14 16 -6 3436.20 325.603 3

-14 16 -6 3487.23 466.976 4

14 -16 6 3591.20 378.173 10

2 14 317315.60 1329.47 9

-2 -14 -316282.70 1262.19 6

2 14 314282.80 1276.03 7

-14 16 -318043.50 1676.03 4

14 -16 314959.20 1274.43 10

-16 2 316050.50 1353.17 10

2 14 0 1054.50 187.625 9

-14 16 0 652.573 365.306 4

2 14 0 1137.28 220.147 7

-14 16 3 8248.16 877.327 4

-2 -14 3 9477.61 818.378 10

2 14 -3 9093.45 896.25 7

2 14 -3 9524.68 903.51 9

-16 2 -610150.80 886.623 1

14 -16 -611310.90 891.807 6

-2 -14 611997.80 989.268 10

2 14 -611844.80 1011.44 4

14 -16 -610820.90 887.895 8

-14 16 612486.80 1001.97 4

2 14 -610771.70 1026.83 9

2 14 -612458.30 1054.21 7

2 14 -610849.40 888.567 2

-2 -14 9 2428.11 342.637 10

14 -16 -9 2816.32 250.816 6

2 14 -9 2676.91 322.064 9

-16 2 -9 2248.94 267.242 8

2 14 -9 3660.54 397.499 7

2 14 -9 2682.84 244.31 11

-16 2 -9 3506.33 261.409 11

2 14 -9 3437.27 252.276 14

-16 2 -9 2288.20 243.66 14

14 -16 -9 2807.52 250.074 8

2 14 -9 2851.28 249.228 2

2 14 -12 4395.51 473.211 7

-16 2 -12 4697.59 355.94 11

-2 -14 12 3076.18 411.542 10

16 -2 12 4173.86 440.55 10

2 14 -12 4565.88 354.838 2

-16 2 -12 3283.72 346.166 14

2 14 -12 4564.39 350.288 14

14 -16 -12 3983.20 351.695 8

14 -16 -15 165.545 38.8047 8

2 14 -15 100.93 36.2922 2

-15 16 -16 176.771 30.444 11

-15 16 -16 205.303 38.8197 2

-1 -15 -16 224.393 37.1287 8

16 -1 -16 214.62 34.063 2

-1 -15 -13 159.317 44.4133 6

-15 16 -13 126.736 43.6286 11

-15 16 -13 63.1722 67.9445 3

-1 -15 -13 182.42 51.3693 8

1 15 13 55.3524 64.3968 10

-16 1 13 173.863 86.9058 10

15 -16 13 68.4009 67.3238 10

-15 16 -13 14.5938 53.0458 2

-1 -15 -10 992.593 110.565 6

-15 16 -10 930.58 128.519 2

-16 1 10 743.819 183.832 10

15 -16 10 1018.53 166.75 10

16 -1 -10 1250.18 170.473 9

-1 -15 -10 950.88 114.919 8

16 -1 -10 1146.86 171.712 7

-15 16 -10 1020.80 116.761 11

-15 16 -10 840.401 130.78 3

-1 -15 -7 6031.02 487.68 6

15 -16 7 5084.27 539.713 10

-16 1 7 6941.05 669.247 10

1 15 7 5200.73 544.941 4

-1 -15 -7 6515.78 495.781 8

-15 16 -7 4319.09 573.081 4

-15 16 -7 5941.59 495.217 2

-15 16 -7 6131.06 492.091 14

-15 16 -7 5644.49 491.866 3

-1 -15 -7 5635.04 484.899 1

-1 -15 -4 1098.95 105.153 6

-1 -15 -4 867.773 100.964 8

-15 16 -4 1145.98 107.05 3

-16 1 4 857.986 193.669 10

15 -16 4 692.984 125.595 10

-15 16 -4 1199.58 104.931 2

-15 16 -4 969.142 371.42 4

1 15 1 2431.37 276.839 9

1 15 1 2008.33 256.237 7

-1 -15 2 262.478 114.322 10

-15 16 2 558.075 227.271 4

1 15 -2 715.325 231.86 7

-1 -15 519055.30 1451.38 10

1 15 -517286.00 1551.20 9

15 -16 -516259.50 1360.00 8

16 -1 515444.30 1376.92 9

1 15 -517591.80 1363.71 2

-15 16 517863.70 1479.55 4

-16 1 -517333.00 1362.17 1

15 -16 -516256.60 1362.88 6

1 15 -521198.20 1570.19 7

1 15 -518218.50 1518.19 4

-1 -15 8 314.103 141.179 10

1 15 -8 290.328 141.923 9

15 -16 -8 389.631 67.869 8

-16 1 -8 440.752 75.469 11

1 15 -8 229.838 111.957 4

-16 1 -8 549.295 75.1081 1

1 15 -8 460.175 68.1098 2

15 -16 -8 550.373 72.5383 6

1 15 -11 801.893 100.531 2

-16 1 -11 757.581 133.206 8

-1 -15 11 737.504 171.632 10

-16 1 -11 1048.51 106.344 11

-16 1 -11 718.263 94.7257 14

1 15 -11 1185.10 101.993 14

15 -16 -11 762.575 99.1996 8

1 15 -11 1186.93 222.647 7

1 15 -14 171.72 40.42 14

15 -16 -14 145.309 42.2322 8

1 15 -14 235.865 47.9317 2

1 15 -17 30.2667 21.9546 2

15 -16 -17-16.7321 19.24 8

1 15 -17 35.9249 21.9552 3

-16 16 -17 189.721 23.0051 11

0 -16 -17 117.297 26.7733 8

16 0 -17 170.858 25.8305 2

-16 16 -17 120.594 31.2354 14

-16 16 -14 -8.2096 48.319 2

-16 16 -14 26.1168 35.4246 11

-16 16 -14 88.136 47.457 14

0 -16 -14 27.2892 38.7641 8

16 0 -14 17.9214 29.2319 2

-16 16 -11 1255.32 122.965 11

-16 16 -11 1292.52 126.831 14

0 -16 -11 1073.58 116.774 6

-16 0 11 767.715 179.198 10

16 -16 11 875.817 154.814 10

16 0 -11 1024.34 156.342 9

0 -16 -11 1111.33 121.627 8

-16 16 -11 983.023 136.115 2

16 0 -11 890.288 160.951 7

-16 16 -11 863.991 141.649 3

16 -16 8 3765.17 451.671 10

0 -16 -8 4891.89 406.978 6

-16 0 8 4532.54 557.809 10

16 0 -8 5507.37 487.195 9

0 -16 -8 5243.31 405.71 1

-16 16 -8 4548.49 411.619 3

0 -16 -8 5366.00 413.896 8

-16 16 -8 4866.73 418.862 2

-16 16 -8 4726.70 411.449 14

-16 16 -8 4699.96 419.875 11

-16 16 -8 3141.04 458.26 4

-16 0 5 2841.39 342.871 10

0 -16 -5 2442.79 201.585 8

-16 16 -5 2102.78 199.642 2

0 -16 -5 2118.21 196.031 1

16 -16 5 2104.69 242.867 10

0 16 5 2124.07 290.386 4

-16 16 -5 2399.96 202.101 3

-16 16 -5 2285.25 392.744 4

0 16 2 6956.24 682.675 9

0 -16 -2 8508.84 619.081 6

-16 16 -2 7968.79 964.976 4

-16 0 2 7428.88 709.684 10

0 16 2 5924.55 635.336 7

-16 16 119880.00 1846.98 4

0 16 -120742.20 1772.05 7

0 -16 120361.60 1699.03 10

0 -16 410634.70 884.14 10

16 -16 -410048.10 807.05 8

16 0 410058.70 841.013 9

0 16 -411922.70 1040.92 9

0 16 -4 9211.02 809.043 2

-16 16 411120.50 949.788 4

0 16 -4 9901.09 1003.37 4

0 16 -7 1521.29 147.279 2

0 16 -7 1615.67 281.494 9

16 -16 -7 1509.48 147.266 8

0 16 -7 2177.71 334.387 7

-16 16 7 1817.69 236.917 4

0 16 -7 1454.99 249.463 4

0 -16 7 2095.29 254.342 10

16 -16 -7 1464.47 146.633 6

-16 0 -7 1987.90 163.297 11

-16 0 -7 1277.82 143.992 1

0 -16 10 91.3711 111.987 10

16 -16 -10 3.54241 43.8236 8

-16 0 -10-109.338 100.483 8

0 16 -10 37.4648 33.7449 11

0 16 -10 44.1364 44.0347 2

0 16 -10 100.996 45.5108 14

0 16 -10 228.674 148.857 7

-16 0 -10 56.9835 47.9723 11

0 16 -13 996.232 109.284 2

0 16 -13 967.448 192.085 7

0 -16 13 822.365 161.056 10

16 0 13 893.831 168.699 10

0 16 -13 1156.98 105.886 14

16 -16 -13 1038.43 107.981 8

0 16 -16 7.92707 26.2686 2

16 -16 -16 38.9481 23.9904 8

0 16 -16 22.5351 26.394 3

-1 17 -15 335.903 50.8649 3

-1 17 -15 385.11 49.324 14

17 -16 -15 321.019 48.0375 8

-1 17 -15 395.978 53.8769 2

1 -17 12-61.5952 90.1956 10

-1 17 -12 68.0927 110.233 7

-1 17 -12 7.04166 44.6494 2

-16 -1 -12-28.5711 78.9662 8

17 -16 -12-19.3696 39.2676 8

-1 17 -12 10.7133 36.3713 14

-1 17 -12 28.9281 33.1276 11

17 -16 -9 789.15 100.67 8

-1 17 -9 884.475 102.427 2

-16 -1 -9 862.542 107.29 1

-1 17 -9 857.246 100.393 11

-16 -1 -9 1254.51 117.411 11

17 -16 -9 773.226 98.5404 6

-1 17 -9 1085.10 106.452 14

1 -17 9 738.652 173.144 10

1 -17 6 8145.03 682.596 10

-1 17 -6 7220.09 777.448 9

17 -16 -6 6938.98 585.831 8

-1 17 -6 7503.72 590.482 2

-17 16 6 8326.03 697.811 4

-16 -1 -6 6658.88 586.116 1

-1 17 -6 7745.98 756.889 4

17 -16 -6 6839.76 586.189 6

-1 17 -3 7071.60 715.244 7

-1 17 -3 6002.26 478.516 3

1 -17 3 6483.30 549.807 10

16 1 3 6141.77 523.918 9

-1 17 -3 7270.07 689.449 9

-16 -1 -3 5115.13 473.466 5

-1 17 -3 4609.50 696.052 4

-1 17 -3 5953.92 477.345 2

-17 16 3 6259.29 635.237 4

-1 17 014676.90 1357.66 9

-1 17 015248.00 1342.79 7

-1 17 019279.50 1547.06 4

-17 16 016347.60 1526.84 4

1 -17 -3 4126.36 366.771 6

-1 17 3 4775.47 494.953 4

-16 -1 3 4762.86 481.697 10

-17 16 -3 4540.51 368.276 3

-17 16 -3 4562.87 670.339 4

-17 16 -6 2041.29 179.202 2

1 -17 -6 1865.81 178.434 8

1 -17 -6 1852.66 173.118 1

17 -16 6 1841.22 223.129 10

16 1 -6 2431.83 266.18 9

-1 17 6 1419.05 238.171 4

-17 16 -6 2042.68 177.785 3

-17 16 -6 1448.51 295.313 4

17 -16 9 7634.25 812.924 10

-17 16 -9 8140.17 773.368 3

1 -17 -910470.90 773.808 8

-17 16 -9 9770.94 785.463 2

1 -17 -9 9419.01 767.803 6

-17 16 -9 8962.30 773.089 14

-17 16 -9 9450.48 778.762 11

-16 -1 9 9915.47 915.618 10

16 1 -910755.70 846.443 9

-17 16 -12 748.928 138.271 3

1 -17 -12 1340.37 118.157 8

-17 16 -12 1116.15 113.148 11

-17 16 -12 1045.83 112.243 14

1 -17 -12 1012.57 106.92 6

16 1 -12 899.411 154.857 7

-1 17 12 874.184 142.889 10

17 -16 12 654.283 139.133 10

-16 -1 12 709.49 154.711 10

-17 16 -12 864.792 128.041 2

1 -17 -15-19.1073 32.0718 8

-17 16 -15 31.8249 33.622 2

-17 16 -15-1.99494 26.5548 11

-17 16 -15 85.5875 38.68 14

16 1 -15 12.7098 23.5056 2

17 -15 -16 50.2209 25.271 8

-2 17 -16 104.424 29.5663 3

-2 17 -16 73.0138 30.1873 2

-15 -2 -13 569.501 92.1185 8

-2 17 -13 456.194 65.622 14

17 -15 -13 599.331 72.1947 8

-2 17 -13 632.014 79.046 2

2 -17 13 446.328 118.296 10

15 2 13 481.479 137.491 10

-2 17 -10 3685.60 330.154 11

-15 -2 -10 4591.77 341.191 11

-2 17 -10 4094.73 332.598 14

17 -15 -10 3377.57 329.398 8

-2 17 -10 3913.57 334.789 2

-2 17 -10 4318.89 501.245 7

-15 -2 -10 3469.64 367.878 8

2 -17 10 3376.07 416.444 10

2 -17 7 79.6806 102.891 10

-2 17 -7 150.022 179.14 9

17 -15 -7 146.71 47.8894 8

-2 17 -7 182.708 211.233 7

-2 17 -7 136.482 46.0968 2

-15 -2 -7 283.937 51.3271 1

-2 17 -7 236.628 135.09 4

-17 15 7 138.842 110.60 4

-15 -2 -7 315.029 60.5304 11

17 -15 -7 187.462 47.2613 6

-2 17 -439992.20 3284.11 2

-17 15 443960.90 3411.33 4

2 -17 445025.70 3352.39 10

-2 17 -441978.80 3562.13 4

-2 17 -440108.10 3285.26 3

-2 17 -448698.80 3546.65 9

15 2 443307.90 3317.95 9

17 -15 -439746.70 3278.97 8

-17 15 1 597.323 252.593 4

15 2 1 437.537 120.261 9

-2 17 -1 623.003 170.758 7

-17 15 -239406.90 3633.53 4

-15 -2 249016.00 3472.81 10

-2 17 239627.70 3379.46 7

-2 17 246669.80 3525.76 4

2 -17 -241916.90 3360.16 6

2 -17 -5 773.469 82.4038 8

15 2 -5 717.553 159.925 9

2 -17 -5 675.625 76.2266 1

-2 17 5 533.178 165.127 4

17 -15 5 614.756 121.47 10

-15 -2 5 811.702 197.572 10

-17 15 -5 516.024 229.385 4

-17 15 -5 830.804 82.9348 3

-17 15 -5 681.298 80.0286 2

-17 15 -8 3197.29 273.53 11

-17 15 -8 3541.06 267.257 14

15 2 -8 3608.75 339.567 9

-15 -2 8 3610.42 423.965 10

-17 15 -8 2633.92 262.772 2

2 -17 -8 3345.29 263.10 8

2 -17 -8 2644.20 252.509 1

-17 15 -8 2033.13 315.541 4

-17 15 -8 2484.75 256.343 3

17 -15 8 2849.06 315.371 10

17 -15 11 146.341 77.0178 10

-15 -2 11 217.692 118.792 10

-2 17 11 370.166 98.3675 10

2 -17 -11 334.623 58.9422 6

2 -17 -11 257.755 60.0258 8

15 2 -11 214.748 90.8959 7

-17 15 -11 153.70 60.1135 14

-17 15 -11 242.731 62.4511 11

-17 15 -11 213.004 79.3078 3

-17 15 -11 197.43 80.5053 2

-17 15 -14 840.77 93.2471 11

2 -17 -14 878.74 96.2692 8

15 2 -14 1044.25 90.9548 2

-17 15 -14 782.432 105.588 2

-17 15 -14 804.161 96.2975 14

-17 15 -17 24.0041 15.6052 11

-17 15 -17-49.1636 28.0286 14

2 -17 -17 -7.9692 19.8355 8

15 2 -17-5.26521 16.7162 2

-3 17 -17 163.38 28.5552 3

-3 17 -17 195.175 30.7587 2

17 -14 -17 96.3379 22.6476 8

-3 17 -14 1415.18 126.062 3

17 -14 -14 1312.21 122.779 8

-3 17 -14 1508.44 129.234 2

-3 17 -11 22.8645 44.312 2

-3 17 -11 37.4734 40.9226 14

17 -14 -11-13.3903 39.3919 8

-14 -3 -11 44.3702 78.473 8

3 -17 11-16.7086 88.2265 10

-3 17 -11 16.0288 34.5225 11

-3 17 -11 164.706 150.41 7

-14 -3 -8 101.764 48.7354 1

-14 -3 -8 29.9733 49.7529 11

17 -14 -8 44.861 40.8595 8

-3 17 -8 19.7042 89.0339 4

-3 17 -8-11.0584 45.9993 14

3 -17 8 17.1619 108.175 10

-3 17 -8 136.021 47.7346 2

-14 -3 -512920.30 1000.17 1

3 -17 513513.70 1073.03 10

17 -14 -511376.50 996.659 8

-17 14 513487.30 1111.93 4

-3 17 -513511.70 1004.97 2

14 3 512409.90 1024.33 9

-3 17 -511198.90 1227.05 9

-3 17 -514057.80 1273.44 4

17 -14 -511403.60 998.211 6

-17 14 2 3294.73 419.163 4

-3 17 -2 2694.84 580.032 4

-3 17 -2 3658.31 415.233 9

14 3 2 3316.49 312.044 9

-3 17 -2 3508.69 410.144 7

-14 -3 -2 2719.27 252.627 5

3 -17 2 3555.31 313.259 10

-3 17 151022.70 4288.55 7

14 3 -151667.40 4325.30 9

-14 -3 4 6244.81 636.858 10

-17 14 -4 6535.59 732.333 4

17 -14 4 5535.33 574.777 10

3 -17 -4 6738.66 534.349 6

-3 17 4 6770.13 640.921 4

17 -14 7 3650.94 398.717 10

14 3 -7 4951.02 428.462 9

-14 -3 7 4883.88 510.214 10

-3 17 7 3905.78 419.592 4

3 -17 -7 4108.04 347.89 8

-17 14 -7 3520.34 435.766 4

-17 14 -7 3579.08 341.998 3

-17 14 -7 4270.88 350.632 14

3 -17 -7 4503.70 345.17 1

-17 14 -7 3866.01 347.353 2

-17 14 -10 1640.14 157.66 14

17 -14 10 1268.97 194.421 10

-14 -3 10 1435.71 245.745 10

14 3 -10 1867.90 208.637 9

3 -17 -10 1613.98 155.621 8

-17 14 -10 1226.48 156.825 3

-17 14 -10 1545.84 155.977 11

14 3 -10 1455.08 218.74 7

-17 14 -10 1335.45 165.627 2

3 -17 -13 2235.58 198.735 8

-17 14 -13 1973.81 193.806 11

3 -17 -13 2235.56 194.923 6

17 -14 13 1512.36 227.605 10

-17 14 -13 1993.31 192.863 14

-14 -3 13 1440.01 237.075 10

-17 14 -13 1874.64 210.763 2

14 3 -13 2855.47 194.56 2

3 -17 -16 204.32 35.6924 8

14 3 -16 213.045 32.4238 2

-17 14 -16 183.312 29.9322 11

-4 17 -18-1.10392 17.0693 2

17 -13 -15 125.193 35.25 8

-4 17 -15 182.103 43.5424 2

-4 17 -15 199.37 41.6216 3

-4 17 -15 78.0915 34.7829 14

-4 17 -12-5.82605 37.6743 14

-13 -4 -12-22.9155 63.889 8

17 -13 -12 22.0226 39.0922 8

-4 17 -12 2.9142 34.5382 11

4 -17 12 30.2705 76.7752 10

-4 17 -12 7.40031 42.4921 2

-4 17 -9 107.925 46.374 11

-4 17 -9 164.054 56.714 14

-13 -4 -9 117.127 78.106 8

17 -13 -9 130.027 42.6602 8

-4 17 -9 46.6291 47.4948 2

-13 -4 -9 124.251 55.1003 1

4 -17 9 122.642 127.327 10

-17 13 9 66.6997 90.1965 10

-13 -4 -9 171.724 57.7954 11

4 -17 6 962.86 173.903 10

-17 13 6 542.883 141.203 10

-4 17 -6 331.863 277.412 9

17 -13 -6 846.776 103.327 8

-17 13 6 1091.69 189.196 4

-13 -4 -6 1631.77 113.964 1

-4 17 -6 1182.59 280.32 4

-4 17 -6 1567.92 409.778 7

17 -13 -6 918.78 104.63 6

-4 17 -6 1216.97 113.258 2

-4 17 -319168.80 1554.11 2

-17 13 321242.90 1690.86 4

13 4 318539.30 1593.15 9

-4 17 -320307.10 1732.89 9

-4 17 -319434.60 1554.05 3

4 -17 318464.10 1597.16 10

-4 17 -316084.00 1941.87 4

-4 17 -322815.50 1775.56 7

-17 13 0 1542.08 357.933 4

-4 17 0 1906.49 216.90 7

-4 17 0 2314.46 410.403 4

13 4 0 1574.95 219.467 9

4 -17 -3 7481.56 620.756 6

-13 -4 3 8211.26 728.552 10

-17 13 -3 7687.64 832.726 4

-4 17 3 7702.93 736.97 4

13 4 -6 3043.47 286.439 9

-13 -4 6 3447.34 365.386 10

-4 17 6 2566.96 285.51 4

4 -17 -6 2825.95 193.406 8

-17 13 -6 1856.16 183.33 2

4 -17 -6 2248.96 188.648 6

17 -13 6 2423.20 254.344 10

4 -17 -6 1635.45 181.089 1

-17 13 -6 1872.78 181.972 3

-17 13 -6 1907.28 296.94 4

-17 13 -9 7950.47 601.62 11

-17 13 -9 8543.21 602.282 14

17 -13 9 6472.73 643.779 10

-13 -4 9 7442.88 725.255 10

13 4 -9 8722.79 664.744 9

-17 13 -9 5468.17 586.582 3

13 4 -9 7998.79 690.011 7

-17 13 -9 6692.12 600.491 2

4 -17 -9 7175.03 592.549 8

-17 13 -12-75.0528 72.7729 2

4 -17 -12 64.3283 45.9189 8

-4 17 12 31.1619 57.9661 10

17 -13 12 48.9464 77.1047 10

-13 -4 12 47.5064 88.3282 10

-17 13 -12 97.0887 50.9544 14

-17 13 -12 78.6485 45.6138 11

13 4 -12-71.4634 83.7484 7

4 -17 -12 20.9036 48.9881 6

13 4 -15 30.4811 26.7044 2

-17 13 -15 98.2078 39.902 14

4 -17 -15 24.9048 31.518 8

-17 13 -15 27.1346 28.7511 11

-5 17 -16 17.7753 28.8547 2

17 -12 -16 23.3223 23.5585 8

-5 17 -16-26.5155 31.5156 14

-5 17 -16 47.396 27.7111 3

-5 17 -13 311.604 60.6456 2

-5 17 -13 294.939 46.839 11

17 -12 -13 269.175 50.2719 8

-12 -5 -13 279.962 65.4166 8

-5 17 -13 245.961 52.471 14

5 -17 13 296.514 100.202 10

-5 17 -10 2298.94 212.828 2

-12 -5 -10 2752.52 219.778 1

-5 17 -10 2284.03 211.059 14

17 -12 -10 2325.78 207.374 8

-5 17 -10 2174.59 208.388 11

-5 17 -10 2102.26 379.799 7

-12 -5 -10 2873.11 240.858 8

5 -17 10 1819.05 278.001 10

-17 12 10 1645.13 258.169 10

-12 -5 -10 2119.95 203.611 6

-12 -5 -710944.00 870.113 6

-5 17 -710460.60 1021.28 4

17 -12 -710102.80 869.836 8

-17 12 712080.20 965.41 4

-17 12 7 8148.72 966.079 10

5 -17 712718.20 972.315 10

-12 -5 -710521.50 871.725 1

-5 17 -710901.30 876.509 2

-17 12 4 87.67 134.684 4

-5 17 -4 49.4763 38.3802 3

12 5 4-7.41963 59.1035 9

-17 12 4 33.0308 43.3882 10

5 -17 4 9.75519 77.657 10

-5 17 -4 78.2069 41.0375 2

-5 17 -4-75.1248 242.911 7

-5 17 -4 70.0297 452.842 4

12 5 1 4423.71 436.265 9

5 -17 1 4715.87 414.47 10

-17 12 1 4935.80 551.138 4

-5 17 -1 4120.47 442.531 7

-17 12 -2-33.4336 224.351 4

-5 17 2 66.8061 161.404 4

12 5 -2-13.4285 104.976 9

12 5 -511068.60 909.267 9

-17 12 -5 9025.07 951.78 4

-5 17 510587.80 927.615 4

5 -17 -510158.60 822.715 8

5 -17 -5 9943.54 823.144 6

12 5 -8 1078.63 168.27 9

5 -17 -8 1020.48 110.323 8

-17 12 -8 812.35 112.622 14

5 -17 -8 1212.58 114.187 6

-12 -5 8 1146.24 248.081 10

17 -12 8 716.365 150.125 10

-17 12 -8 1245.74 117.25 2

-17 12 -8 836.641 115.42 11

5 -17 -11 2530.45 215.545 8

12 5 -11 2287.24 288.014 7

-5 17 11 1850.42 242.319 10

17 -12 11 1542.01 245.62 10

-17 12 -11 2200.87 211.169 14

-12 -5 11 2358.79 297.606 10

-17 12 -11 2484.17 237.946 2

-17 12 -11 2513.72 214.234 11

-17 12 -14 2315.77 208.828 14

-17 12 -14 1949.78 220.796 2

12 5 -14 2546.76 208.637 2

-17 12 -14 2504.27 210.917 11

5 -17 -17 135.533 27.7061 8

12 5 -17 131.209 25.1222 2

17 -11 -17 110.039 23.405 8

-6 17 -17 222.462 33.9778 2

-6 17 -14 710.081 83.5579 3

-11 -6 -14 764.06 89.318 8

17 -11 -14 723.285 77.3426 8

-6 17 -14 676.386 83.1086 2

-6 17 -14 573.575 70.5603 11

-6 17 -14 729.681 79.9083 14

-17 11 11-13.3359 76.9046 10

6 -17 11 111.215 95.942 10

-11 -6 -11 54.7748 39.1459 6

-6 17 -11-32.5771 161.78 7

-6 17 -11 38.5565 40.6202 11

-6 17 -11 10.5102 46.2089 14

-11 -6 -11 36.8286 57.166 8

17 -11 -11-6.18897 38.2272 8

-6 17 -11 20.334 50.0364 2

6 -17 8 1798.90 262.947 10

-17 11 8 1465.65 271.434 10

-6 17 -8 1967.43 191.801 2

17 -11 -8 1833.21 183.03 8

-11 -6 -8 2434.28 218.964 8

-11 -6 -8 2098.36 186.491 6

-6 17 -8 2368.72 195.081 14

-6 17 -8 1391.98 253.39 4

-6 17 -8 2205.94 195.916 11

-17 11 5 226.144 131.93 4

-6 17 -5 450.64 64.7979 3

11 6 5 257.417 73.3335 9

-6 17 -5 97.4192 286.88 7

-6 17 -5 519.00 63.7822 2

-17 11 5 271.782 97.1269 10

6 -17 5 228.684 94.3948 10

-6 17 -5 740.23 395.926 4

6 -17 2 4248.68 401.802 10

-17 11 2 4893.35 526.282 4

11 6 2 4152.01 417.823 9

-6 17 -2 4800.29 472.571 7

11 6 -112115.70 1062.89 9

-6 17 111796.50 1022.25 7

-6 17 114773.60 1205.65 4

-6 17 4 415.812 168.455 4

-17 11 -4 491.187 214.055 4

11 6 -4 385.28 134.315 9

6 -17 -4 485.026 62.7538 6

6 -17 -710176.80 786.943 6

11 6 -710423.90 858.877 9

-17 11 -7 8842.98 783.061 2

-6 17 710213.70 878.605 4

11 6 -10 1445.49 185.246 9

11 6 -10 1360.97 212.939 7

-17 11 -10 1332.56 154.487 2

6 -17 -10 1676.73 143.798 6

-17 11 -10 1192.77 138.779 14

17 -11 10 887.993 175.072 10

-6 17 10 1158.06 169.542 10

-11 -6 10 957.001 210.542 10

-17 11 -10 1311.96 138.315 11

-17 11 -13 155.293 49.9666 11

11 6 -13 252.183 47.1879 2

-17 11 -13 135.128 68.926 2

-17 11 -13 268.584 55.992 14

11 6 -13 234.895 88.5132 7

17 -11 13 231.033 93.9213 10

-11 -6 13 83.9628 80.9912 10

11 6 -16 411.633 51.3096 2

-17 11 -16 412.873 55.2562 14

-17 11 -16 515.698 51.2817 11

17 -10 -15 1569.96 142.886 8

-7 17 -15 1419.69 137.071 11

-7 17 -15 1712.66 145.642 14

-7 17 -15 1786.48 149.185 3

17 -10 -12 3738.75 318.795 8

-10 -7 -12 4544.33 337.418 8

-7 17 -12 3720.63 321.825 14

-7 17 -12 3705.29 327.078 2

-10 -7 -12 4071.92 320.801 6

-7 17 -12 3324.43 318.019 11

-17 10 9 131.637 119.438 10

7 -17 9 32.185 94.0438 10

-7 17 -9 237.765 60.6421 2

-10 -7 -9 220.429 67.8817 8

17 -10 -9 246.229 46.6968 8

-10 -7 -9 221.253 51.6355 6

-7 17 -9 102.829 56.9127 14

-7 17 -9 180.721 53.3082 11

-7 17 -9 214.492 288.915 7

17 -10 -6 366.187 54.174 8

-17 10 6 722.196 152.251 4

-7 17 -6 416.839 64.0264 2

-10 -7 -6 364.174 53.4753 6

-17 10 6 348.421 144.877 10

7 -17 6 260.732 104.812 10

-7 17 -6 252.271 237.912 4

-7 17 -6 424.334 345.10 7

-17 10 3 653.034 172.272 4

-7 17 -3 386.784 54.4533 3

-7 17 -3 311.557 54.1131 2

7 -17 3 316.652 83.477 10

-17 10 3 230.324 72.313 10

-7 17 -3 574.66 771.756 4

10 7 3 630.654 104.906 9

-7 17 -3 505.995 168.658 7

10 7 0 837.23 153.908 9

-17 10 0 420.103 228.946 4

-7 17 0 821.096 336.833 4

-7 17 0 787.821 128.48 7

-7 17 332730.30 2705.66 4

-10 -7 332306.50 2670.29 10

-17 10 -331176.00 2751.16 4

10 7 -6 3824.23 384.408 9

-17 10 -6 3878.18 432.36 4

7 -17 -6 3724.70 311.957 6

7 -17 -6 3421.51 310.167 1

-7 17 6 4152.89 420.632 4

-17 10 -9 8912.66 739.211 14

17 -10 9 8269.67 795.182 10

-7 17 9 7729.56 770.468 10

7 -17 -9 9715.56 735.992 6

-17 10 -9 8346.39 739.75 2

10 7 -9 9095.08 791.756 9

10 7 -9 9397.32 832.748 7

-17 10 -9 8985.92 738.979 11

10 7 -12 4916.15 512.612 7

-17 10 -12 3490.78 442.178 2

10 7 -12 5799.96 430.045 2

-17 10 -12 5246.60 430.231 14

17 -10 12 4182.72 483.237 10

-17 10 -12 6020.66 436.608 11

-10 -7 12 4188.27 497.08 10

10 7 -15 43.5897 27.6714 2

-17 10 -15-4.59806 36.0483 14

-17 10 -15 2.1339 28.0676 11

10 7 -18 31.877 16.4148 3

-8 17 -16 72.5683 30.8667 3

17 -9 -16 31.9377 24.1457 8

-8 17 -16 48.8828 34.8669 14

-9 -8 -13 1366.15 127.985 8

17 -9 -13 1243.40 115.79 8

-8 17 -13 1065.57 123.914 3

9 8 13 878.562 172.187 10

8 -17 13 667.212 155.095 10

-8 17 -13 1081.71 112.732 11

-8 17 -13 1269.42 120.292 14

-8 17 -10 688.392 92.1412 14

17 -9 -10 561.42 76.4103 8

-9 -8 -10 907.181 99.7916 8

-17 9 10 396.989 137.694 10

8 -17 10 670.035 149.497 10

-9 -8 -10 649.179 90.9327 1

-9 -8 -10 646.652 83.7946 6

-8 17 -10 798.579 92.3512 11

17 -9 -7 3689.00 372.238 8

-9 -8 -7 5793.05 401.49 8

-8 17 -7 5715.75 855.547 7

-8 17 -7 4153.98 513.435 4

8 -17 7 4746.37 457.55 10

-17 9 7 3844.40 504.635 10

-9 -8 -7 4750.25 380.64 1

-9 -8 -7 4742.41 378.327 6

-8 17 -7 4616.64 386.614 2

8 -17 419522.60 1570.08 10

-17 9 415988.60 1573.33 10

-8 17 -418941.50 1529.60 2

-8 17 -424449.20 1747.65 7

9 8 419326.50 1566.90 9

-17 9 419880.10 1638.61 4

-8 17 -420271.70 2151.43 4

-8 17 -122526.50 1955.56 7

9 8 123820.30 1958.02 9

-17 9 125859.00 2068.50 4

-9 -8 2 9510.97 790.839 10

-17 9 -2 8705.75 884.388 4

9 8 -2 7938.55 776.012 9

-8 17 2 8035.63 845.128 4

-9 -8 522207.40 1552.55 10

-17 9 -520130.10 1573.14 4

8 -17 -516349.50 1418.81 6

-8 17 521235.50 1559.45 4

8 -17 -517564.10 1419.15 8

9 8 -518770.30 1505.95 9

-9 -8 8 5025.66 560.33 10

-8 17 8 5133.64 479.827 10

8 -17 -8 5402.05 440.375 6

8 -17 -8 4881.81 437.494 8

9 8 -8 5932.37 546.959 7

-17 9 -8 5117.04 446.008 14

9 8 -8 5411.79 501.789 9

9 8 -8 6035.50 436.779 2

-17 9 -8 4389.12 434.854 2

-17 9 -8 5241.84 446.861 11

-17 9 -11 5335.55 431.317 11

8 -17 -11 4955.98 428.335 6

17 -9 11 4821.87 494.214 10

-8 17 11 4401.95 461.95 10

9 8 -11 4703.94 514.906 7

9 8 -11 5521.25 423.399 14

-17 9 -11 4836.75 427.623 14

9 8 -11 5886.46 427.299 2

-17 9 -11 3582.91 440.728 2

8 -17 -11 4935.46 428.415 8

-17 9 -14 157.003 48.7037 14

-17 9 -14 106.977 40.0483 11

8 -17 -14 80.2055 43.1032 8

9 8 -14 113.21 37.4088 2

9 8 -17 91.5353 24.7568 2

8 -17 -17 78.5237 24.3747 8

-9 17 -17-29.8991 29.9936 14

-9 17 -17 97.9865 27.7765 2

-9 17 -14 249.378 55.3581 3

-8 -9 -14 357.257 58.8777 8

17 -8 -14 247.704 45.8364 8

-9 17 -14 252.309 43.5484 11

-9 17 -14 348.863 57.3435 14

-17 8 11 192.923 103.232 10

9 -17 11 366.895 114.931 10

-9 17 -11 355.219 80.7943 3

-8 -9 -11 298.297 69.7587 8

17 -8 -11 361.586 54.4411 8

-9 17 -11 335.511 70.2403 14

-9 17 -11 356.755 61.8758 11

-8 -9 -11 372.597 65.1408 6

-9 17 -8 3393.04 293.456 11

-8 -9 -8 3375.64 281.36 6

-9 17 -8 3519.02 290.934 14

17 -8 -8 2760.09 274.217 8

-8 -9 -8 3981.87 297.821 8

-9 17 -8 2549.55 347.05 4

-8 -9 -8 3271.00 283.151 1

-9 17 -8 3271.06 302.059 3

-17 8 8 3074.08 406.654 10

9 -17 8 3107.50 356.398 10

-17 8 5 206.45 148.119 4

-8 -9 -5 107.688 36.0798 6

-9 17 -5-35.4617 207.813 7

8 9 5 31.1725 56.8678 9

-17 8 5 48.334 113.335 10

9 -17 5 48.8547 73.2529 10

9 -17 225793.90 2183.27 10

-9 17 -225002.80 2241.65 7

-17 8 231946.90 2336.54 4

8 9 228432.80 2225.13 9

-8 -9 1 1562.63 228.268 10

8 9 -1 1817.51 239.654 9

9 -17 -4 3166.47 268.566 6

-8 -9 4 3269.36 364.924 10

8 9 -4 2747.21 350.545 9

-9 17 4 2914.45 394.883 4

-17 8 -4 3668.41 445.406 4

-8 -9 7 2245.82 302.376 10

8 9 -7 2279.93 259.523 9

9 -17 -7 1976.74 184.411 8

8 9 -7 2189.55 182.656 2

-9 17 7 2035.39 272.427 4

9 -17 -7 1904.40 185.802 6

-9 17 7 1730.07 215.796 10

8 9 -10 71.8891 77.5173 9

9 -17 -10-3.73447 49.5843 8

8 9 -10-37.9356 107.602 7

-17 8 -10 27.6576 51.1609 14

-17 8 -10 100.365 51.6611 11

-8 -9 10 77.611 123.871 10

-9 17 10 19.6369 58.4566 10

17 -8 10 30.0013 98.2669 10

8 9 -10 45.1769 38.6172 2

-17 8 -10-6.01259 58.1247 2

9 -17 -10 37.0479 51.2425 6

8 9 -13 442.536 128.485 7

8 9 -13 878.406 83.4483 2

-17 8 -13 492.574 100.517 2

17 -8 13 672.725 133.013 10

-17 8 -13 662.29 82.5844 11

-17 8 -13 555.34 80.6892 14

9 -17 -13 605.279 83.778 8

8 9 -16 87.241 28.7408 2

9 -17 -16 113.034 32.5021 8

-10 17 -18-1.19211 17.3691 2

-10 17 -15 149.456 45.6345 2

17 -7 -15 185.269 34.7277 8

-7 -10 -15 150.719 41.2116 8

-10 17 -15 123.943 29.9459 11

-10 17 -15 187.497 45.0871 14

-10 17 -15 189.324 42.5042 3

-10 17 -12 3405.27 318.531 3

-10 17 -12 3670.69 310.538 2

-7 -10 -12 3793.96 308.939 8

-10 17 -12 3444.59 300.649 11

-7 -10 -12 3848.61 301.613 6

17 -7 -12 3674.96 295.961 8

-10 17 -12 3102.94 299.979 14

-17 7 12 2487.32 346.507 10

10 -17 12 2397.91 343.94 10

-17 7 9 1398.60 250.318 10

-7 -10 -9 1625.23 160.298 1

-10 17 -9 1597.03 165.834 14

17 -7 -9 1541.22 149.099 8

-7 -10 -9 1888.64 169.917 8

-10 17 -9 1688.26 183.598 3

-7 -10 -9 1755.51 159.68 6

10 -17 9 1568.68 226.042 10

-10 17 -9 1513.02 164.811 11

-7 -10 -6 3119.70 240.488 8

-7 -10 -6 2666.75 225.63 6

-7 -10 -6 2089.25 224.108 1

-10 17 -6 2604.33 234.762 3

-17 7 6 2907.03 372.541 10

-10 17 -6 2515.28 415.654 4

10 -17 6 2955.36 294.263 10

7 10 341425.10 3195.05 9

-10 17 -338978.80 3242.33 7

-17 7 334294.80 3194.12 10

10 -17 338919.10 3165.25 10

-17 7 345822.70 3293.65 4

7 10 0 8513.31 782.178 9

-17 7 0 7745.72 876.373 4

-7 -10 3 8531.86 750.928 10

-10 17 3 8786.65 818.285 4

-17 7 -3 8172.53 833.269 4

-10 17 3 7810.24 681.40 10

-7 -10 6 2872.70 338.59 10

10 -17 -6 2845.52 229.141 8

7 10 -6 2742.23 309.387 9

10 -17 -6 2691.32 231.265 6

-10 17 6 2957.67 341.28 4

7 10 -6 2671.53 225.513 2

-10 17 6 3034.65 272.403 10

-17 7 -6 2114.21 224.003 1

7 10 -9 529.029 123.859 9

10 -17 -9 455.166 74.7693 8

-17 7 -9 570.815 80.4271 11

-17 7 -9 540.67 80.3402 14

10 -17 -9 525.133 75.895 6

-7 -10 9 450.956 153.85 10

-10 17 9 462.081 96.5375 10

7 10 -9 576.039 160.34 7

7 10 -9 530.46 69.8165 2

-17 7 -9 421.783 72.3304 2

10 -17 -12 1401.87 149.058 8

10 -17 -12 1551.70 150.546 6

-17 7 -12 773.545 158.554 2

7 10 -12 1670.52 148.142 2

7 10 -12 1752.06 148.725 14

-17 7 -12 1667.94 149.509 14

7 10 -12 1393.04 220.892 7

17 -7 12 1078.03 198.579 10

-7 -10 12 1283.45 213.611 10

-17 7 -12 1792.40 153.084 11

7 10 -15 273.587 42.3889 2

-17 7 -15 258.241 41.4974 11

10 -17 -15 212.812 43.152 8

7 10 -18 72.9197 19.1214 3

10 -17 -18 80.8513 18.8671 8

-6 -11 -16 4.11808 26.5549 8

-11 17 -16 39.5347 28.7053 2

-11 17 -16-3.77837 34.8366 14

-11 17 -16 4.96066 18.1319 11

-17 6 13 12.1598 65.6814 10

11 -17 13 85.8142 68.6043 10

6 11 13 28.0614 75.7129 10

-6 -11 -13 43.803 38.092 6

-11 17 -13 104.936 48.9035 2

-11 17 -13 212.109 54.6699 14

-6 -11 -13 48.2749 44.7234 8

17 -6 -13 65.3843 32.3133 8

-11 17 -13 51.6145 51.8667 3

-11 17 -13 88.3773 40.5403 11

-11 17 -10 2559.85 275.248 3

-11 17 -10 3256.72 261.589 14

-11 17 -10 2648.07 259.758 2

17 -6 -10 2737.54 242.167 8

-6 -11 -10 3011.88 259.486 8

17 -6 -10 2325.47 285.607 7

-11 17 -10 3109.08 259.882 11

-6 -11 -10 2793.30 249.919 6

11 -17 10 2449.93 311.403 10

-17 6 10 2307.24 325.173 10

-6 -11 -10 2829.86 254.382 1

-6 -11 -7 48.5813 36.0317 6

-6 -11 -7 97.8388 52.4509 8

-11 17 -7 44.8384 48.2871 3

-17 6 7 52.2871 148.353 10

11 -17 7 39.0528 81.0294 10

-11 17 -7 22.6191 114.846 4

-6 -11 -7 63.8817 39.0623 1

-11 17 -7 94.1774 45.7437 2

-17 6 4 4400.19 471.215 10

-11 17 -4 4789.69 394.429 3

-6 -11 -4 4558.39 390.242 6

11 -17 4 4185.52 420.168 10

-11 17 -4 5008.89 395.716 2

-11 17 -4 5346.42 844.008 4

6 11 4 4724.24 437.579 9

-17 6 131323.30 2568.26 4

6 11 128922.10 2440.70 9

-11 17 2 4739.62 545.338 4

-17 6 -2 3767.40 537.626 4

-6 -11 2 4272.88 435.556 10

11 -17 -5 2654.37 233.278 6

-11 17 5 2908.86 365.268 4

-11 17 5 2966.98 278.801 10

-6 -11 5 2572.43 320.765 10

11 -17 -5 2566.52 230.89 8

6 11 -5 2780.03 331.453 9

6 11 -8 410.596 122.972 9

11 -17 -8 601.717 76.0446 8

6 11 -8 614.981 172.948 7

-17 6 -8 589.683 82.028 11

6 11 -8 567.772 113.594 4

-6 -11 8 613.169 175.809 10

-11 17 8 378.86 94.0525 10

6 11 -8 600.622 72.0815 2

11 -17 -8 436.953 72.9712 6

11 -17 -11 822.181 94.3501 8

6 11 -11 706.993 171.079 7

-6 -11 11 491.342 154.973 10

17 -6 11 695.287 156.10 10

6 11 -11 853.496 84.7066 14

-17 6 -11 692.44 89.9384 14

-17 6 -11 743.229 93.5715 11

11 -17 -11 704.578 92.5474 6

6 11 -11 722.916 86.3373 2

-17 6 -11 395.368 101.16 2

-17 6 -14 619.219 86.9704 14

-17 6 -14 899.181 89.8688 11

6 11 -14 886.738 89.1369 2

11 -17 -14 853.043 91.6913 8

6 11 -17-5.78276 22.0523 2

11 -17 -17 11.5926 20.3542 8

-12 17 -17 70.4767 30.1159 14

17 -5 -17 84.8665 22.7597 2

-12 17 -17 72.1489 25.9586 2

-12 17 -14 145.587 49.9742 2

17 -5 -14 123.604 34.8657 8

-5 -12 -14 89.9967 43.8863 8

-12 17 -14 209.555 52.3416 3

-12 17 -14 192.044 49.7695 14

-12 17 -14 148.062 36.6669 11

-5 -12 -11 1579.06 140.422 6

-12 17 -11 1134.38 138.462 11

17 -5 -11 1300.28 183.42 9

-17 5 11 977.235 191.114 10

12 -17 11 1220.81 193.912 10

-12 17 -11 1978.34 162.318 2

-5 -12 -11 1532.26 148.399 8

17 -5 -11 1420.20 130.868 8

-12 17 -11 1111.30 142.266 14

-12 17 -11 1186.28 164.167 3

-12 17 -8 5342.52 436.655 11

12 -17 8 4621.30 485.037 10

-12 17 -8 5603.78 431.425 14

-17 5 8 5599.66 589.572 10

-5 -12 -8 4648.10 420.071 1

-5 -12 -8 5865.40 433.017 8

-5 -12 -8 4869.68 420.307 6

-12 17 -8 4939.77 437.523 3

-12 17 -8 3680.63 480.923 4

-12 17 -8 4585.70 430.856 2

-12 17 -5 5410.37 469.589 2

-5 -12 -5 5901.49 465.783 6

12 -17 5 5466.52 508.531 10

-17 5 5 6113.25 590.29 10

-12 17 -5 5522.38 469.048 3

-12 17 -5 4624.59 703.401 4

5 12 221822.20 1864.70 9

-12 17 -229286.90 2499.81 4

-5 -12 1 5685.74 532.839 10

5 12 -1 5040.75 548.314 9

-12 17 1 6841.49 726.635 4

-5 -12 417928.30 1534.21 10

-12 17 418306.40 1592.08 4

5 12 -417877.80 1574.95 9

12 -17 -7 1025.92 111.941 6

-12 17 7 1239.55 154.736 10

-5 -12 7 1372.46 221.358 10

5 12 -7 1490.00 210.458 9

12 -17 -7 1106.72 109.252 8

-12 17 7 1196.68 197.132 4

5 12 -7 950.778 104.955 2

-17 5 -7 921.874 107.489 1

5 12 -7 1188.85 180.451 4

12 -17 -10 279.391 60.839 8

5 12 -10 290.762 52.8122 14

-17 5 -10 274.653 58.5234 14

5 12 -10 308.906 136.806 7

12 -17 -10 321.806 63.342 6

5 12 -10 331.62 58.9456 2

-17 5 -10 261.176 63.3305 11

-5 -12 10 133.286 123.688 10

-17 5 -13 184.348 57.1735 14

5 12 -13 419.491 61.5029 14

5 12 -13 490.915 135.487 7

5 12 -13 559.179 64.5536 2

12 -17 -13 553.088 68.8247 8

17 -5 13 169.081 96.9721 10

-5 -12 13 210.388 98.7322 10

-17 5 -13 373.184 62.2157 11

5 12 -16 317.087 44.2001 2

12 -17 -16 341.734 45.2765 8

-4 -13 -15 83.7216 36.2918 8

-13 17 -15 38.4094 36.5655 2

-13 17 -15 120.368 29.2409 11

-13 17 -15 126.548 45.1306 14

-17 4 12 202.968 93.0945 10

13 -17 12 254.296 97.1901 10

-4 -13 -12 446.971 64.3623 6

-13 17 -12 384.324 90.3589 3

17 -4 -12 377.809 52.6628 8

-4 -13 -12 518.03 73.9855 8

-13 17 -12 157.663 61.8987 14

-13 17 -12 472.144 82.8582 2

-13 17 -12 324.219 60.4958 11

-13 17 -9 486.696 86.4463 14

-4 -13 -9 608.229 78.6569 1

-13 17 -9 546.671 92.8824 2

-4 -13 -9 671.265 86.8906 8

-13 17 -9 526.071 97.654 3

-13 17 -9 455.875 83.5863 11

-4 -13 -9 683.748 79.0073 6

-17 4 9 593.629 185.845 10

13 -17 9 648.899 138.148 10

-4 -13 -6 534.44 69.6628 6

-4 -13 -6 301.647 65.0418 1

-13 17 -6 654.48 79.9768 2

-13 17 -6 763.87 81.0623 3

13 -17 6 597.092 121.534 10

-13 17 -6 695.922 210.658 4

-4 -13 -6 760.30 81.7908 8

-17 4 3 4155.12 444.023 10

-13 17 -3 4655.55 362.869 2

-13 17 -3 4661.70 814.782 4

13 -17 3 3441.18 369.64 10

-4 -13 -3 4193.45 360.919 6

-13 17 -3 4900.89 365.11 3

4 13 3 3968.01 412.536 9

4 13 0 1499.28 226.53 9

-13 17 0 1641.46 451.838 4

4 13 -317189.40 1548.33 9

-13 17 318105.70 1573.21 4

-4 -13 317468.80 1489.16 10

-4 -13 6 1397.99 205.61 10

4 13 -6 1337.89 220.28 9

13 -17 -6 951.473 99.5223 8

4 13 -6 1004.91 98.4125 2

-17 4 -6 833.913 96.2432 1

-13 17 6 1040.91 198.699 4

4 13 -6 1104.34 211.073 7

13 -17 -6 933.46 103.602 6

4 13 -9 102.542 82.2363 9

13 -17 -9 120.813 49.8687 8

4 13 -9 179.934 48.5038 2

-17 4 -9 155.155 53.8413 11

13 -17 -9 172.305 58.2475 6

-4 -13 9 39.5807 121.604 10

-17 4 -9 53.5994 41.8482 14

4 13 -9 42.8243 123.809 7

-17 4 -12 1719.63 154.479 11

4 13 -12 1950.32 150.975 14

-17 4 -12 1351.68 146.785 14

13 -17 -12 1370.68 151.825 8

-4 -13 12 1229.32 212.501 10

17 -4 12 1575.60 226.409 10

4 13 -12 1284.64 231.894 7

4 13 -12 1563.49 149.874 2

13 -17 -15 307.501 50.1899 8

4 13 -15 379.348 49.9339 2

-14 17 -16 521.295 56.6882 11

17 -3 -16 613.799 60.1609 2

-14 17 -16 678.041 67.2443 2

-3 -14 -16 566.592 62.6621 8

-14 17 -16 513.539 65.6129 14

-3 -14 -13 957.211 93.9657 6

-14 17 -13 928.515 100.265 14

3 14 13 678.957 131.455 10

-17 3 13 633.563 133.96 10

-14 17 -13 891.167 105.906 2

-3 -14 -13 937.736 99.0149 8

-14 17 -13 797.413 115.96 3

-14 17 -13 817.434 93.3699 11

17 -3 -13 927.503 86.8738 8

-14 17 -10 372.967 74.5153 11

-17 3 10 337.968 133.083 10

14 -17 10 254.454 103.734 10

17 -3 -10 382.478 111.524 9

-3 -14 -10 370.861 71.0469 8

-14 17 -10 277.892 89.2104 3

-14 17 -10 332.152 83.7729 2

-3 -14 -10 427.966 68.1362 6

-14 17 -10 279.509 75.0775 14

17 -3 -10 348.564 97.8133 7

-3 -14 -7 1082.92 106.828 6

14 -17 7 714.584 147.709 10

-17 3 7 1260.45 253.699 10

-14 17 -7 906.435 110.221 3

-14 17 -7 769.308 196.772 4

-3 -14 -7 1193.93 115.785 8

-14 17 -7 960.148 122.026 11

3 14 7 1109.25 163.936 4

-3 -14 -7 935.664 102.773 1

-14 17 -7 1096.84 115.683 2

-14 17 -423733.60 1876.31 3

-14 17 -423921.60 2222.68 4

-14 17 -422471.70 1874.42 2

-3 -14 -424196.40 1874.43 6

14 -17 421409.10 1896.73 10

-17 3 426290.60 1991.32 10

3 14 131652.40 2614.33 9

3 14 -226603.20 2266.25 7

-3 -14 226796.00 2210.87 10

-14 17 226446.60 2323.98 4

-3 -14 511637.90 954.505 10

14 -17 -510728.00 860.052 8

3 14 -511386.10 1022.57 9

-17 3 -510227.50 859.318 1

3 14 -514211.90 1031.15 7

-14 17 511446.90 991.692 4

14 -17 -510037.90 862.331 6

3 14 -510648.90 860.737 2

3 14 -815356.80 1436.86 9

14 -17 -816210.00 1347.92 8

-3 -14 818354.00 1469.69 10

14 -17 -817019.20 1350.50 6

3 14 -817254.40 1348.70 2

-17 3 -815367.10 1348.33 1

3 14 -816662.70 1429.37 4

-17 3 -817669.80 1358.58 11

3 14 -11 3655.99 295.533 2

3 14 -11 3126.71 399.804 7

3 14 -11 3876.25 291.761 14

-17 3 -11 2570.02 285.806 14

14 -17 -11 3563.50 295.986 8

14 -17 -11 3454.02 294.106 6

-17 3 -11 3797.45 299.414 11

-3 -14 11 2265.30 354.157 10

3 14 -14 11.9491 32.8428 14

3 14 -14-36.0628 36.1184 2

14 -17 -14-11.0582 33.8842 8

3 14 -17 35.1962 22.264 3

14 -17 -17 21.0176 18.5153 8

3 14 -17 41.6535 22.3957 2

17 -2 -17 470.973 43.4316 2

-15 17 -17 381.321 39.7505 11

-15 17 -14 238.488 56.4061 2

-2 -15 -14 328.833 54.0385 8

-15 17 -14 213.101 44.6384 11

-2 -15 -11 1628.73 147.149 6

-15 17 -11 1481.51 149.628 11

-17 2 11 962.344 204.128 10

15 -17 11 1107.00 187.039 10

17 -2 -11 1362.39 188.413 9

-2 -15 -11 1760.41 155.258 8

-15 17 -11 1451.58 166.112 2

17 -2 -11 1220.51 185.346 7

-15 17 -11 1245.24 176.612 3

15 -17 8 4748.06 554.473 10

-2 -15 -8 5935.59 506.168 6

-17 2 8 6608.55 673.992 10

-15 17 -8 6337.33 522.999 11

-2 -15 -8 7136.47 517.016 8

-15 17 -8 6330.57 519.611 2

-15 17 -8 4180.32 566.483 4

-15 17 -8 6054.52 516.661 3

-2 -15 -8 5986.27 504.568 1

-17 2 554673.30 3598.67 10

-15 17 -542018.80 3429.52 3

-2 -15 -541448.20 3425.45 1

-15 17 -542387.10 3429.49 2

-15 17 -538516.30 3635.68 4

-2 -15 -544721.10 3427.94 6

15 -17 536748.40 3459.28 10

-2 -15 -544896.20 3429.72 8

2 15 544549.70 3514.75 4

2 15 2 6636.83 589.226 9

-15 17 -2 6327.30 904.521 4

2 15 2 6471.22 561.576 7

-15 17 -2 6115.38 516.292 3

2 15 -1 7750.52 692.616 7

2 15 -1 6532.50 656.76 9

-2 -15 1 6341.47 608.60 10

-15 17 1 5880.54 749.079 4

-15 17 4 2466.87 340.572 4

-17 2 -4 2108.01 194.985 1

2 15 -4 2215.22 333.631 7

15 -17 -4 2211.51 195.402 8

2 15 -4 2427.09 364.532 9

2 15 -4 2582.74 199.553 2

17 -2 4 1789.09 221.425 9

-2 -15 4 2265.09 273.782 10

-2 -15 7 1558.92 250.732 10

2 15 -7 1485.86 254.988 9

15 -17 -7 1659.66 162.427 8

2 15 -7 1922.23 300.624 7

2 15 -7 1650.84 251.268 4

-15 17 7 1737.94 248.836 4

-17 2 -7 1665.87 163.126 1

2 15 -7 1754.25 162.883 2

15 -17 -7 1982.78 168.62 6

-2 -15 10 1122.91 202.06 10

15 -17 -10 1037.79 118.581 8

-17 2 -10 664.965 139.75 8

15 -17 -10 1345.31 123.082 6

2 15 -10 1538.90 244.19 7

-17 2 -10 831.148 112.097 14

2 15 -10 1224.60 117.567 14

2 15 -10 1441.29 123.882 2

-17 2 -10 1435.02 130.497 11

15 -17 -13 472.30 64.8608 8

-17 2 -13 567.454 69.6824 11

2 15 -13 525.043 64.4318 2

17 -2 13 555.043 128.578 10

-2 -15 13 221.233 101.007 10

-17 2 -13 135.042 52.9909 14

2 15 -13 504.813 60.5742 14

2 15 -13 378.00 139.144 7

2 15 -16 7.60381 26.8053 2

15 -17 -16 19.0415 25.0009 8

2 15 -16 24.252 26.5035 3

-1 -16 -15 132.363 37.7186 8

-16 17 -15 218.325 44.2305 2

-16 17 -15 157.155 34.2809 11

17 -1 -15 152.856 32.3995 2

-16 17 -12 1741.35 155.857 11

-1 -16 -12 1635.04 150.104 6

-16 17 -12 1155.39 178.049 3

-1 -16 -12 1831.80 156.861 8

-16 17 -12 1614.12 170.345 2

-17 1 12 1141.38 201.124 10

16 -17 12 1174.11 189.557 10

1 16 12 1229.86 191.141 10

-16 17 -9 6542.15 572.84 11

-1 -16 -9 7445.30 571.477 8

-1 -16 -9 6880.60 562.254 1

-16 17 -9 7144.99 582.972 2

-1 -16 -9 6659.37 563.049 6

-16 17 -9 6408.23 576.247 3

17 -1 -9 7383.02 637.468 9

-17 1 9 6163.95 688.931 10

16 -17 9 5339.69 608.738 10

-16 17 -6 73.7286 44.6419 2

-1 -16 -6 43.1998 34.9424 1

1 16 6 13.6927 93.1318 4

-1 -16 -6 88.7673 38.819 6

-16 17 -6 100.797 41.8696 3

-16 17 -6 107.991 150.638 4

-1 -16 -6 95.7249 42.5375 8

16 -17 6-10.2711 65.7883 10

-17 1 6-51.5001 160.068 10

-16 17 -3 42.3198 31.9286 2

-16 17 -3 66.8896 39.6012 3

-17 1 3 85.1408 127.213 10

-16 17 -3 54.5375 352.679 4

1 16 0 5270.34 462.444 7

-16 17 0 4076.53 343.163 3

-17 1 0 3849.58 340.077 5

1 16 0 4844.56 458.583 9

-16 17 0 4085.47 613.723 4

1 16 -318405.80 1414.73 9

1 16 -316560.40 1388.42 7

-16 17 315311.20 1352.13 4

1 16 -316094.00 1197.42 3

-1 -16 317466.70 1280.01 10

17 -1 314468.80 1238.41 9

-17 1 -313264.70 1191.39 5

1 16 -621542.90 1630.43 7

16 -17 -617536.80 1431.85 6

-1 -16 618713.50 1525.45 10

1 16 -616551.40 1585.12 9

16 -17 -617873.10 1429.99 8

1 16 -617292.30 1430.91 2

-17 1 -617330.50 1430.13 1

1 16 -619657.90 1576.84 4

-16 17 619139.70 1540.11 4

1 16 -9 4419.83 355.938 14

1 16 -9 2905.59 414.624 9

16 -17 -9 3905.99 354.069 8

-1 -16 9 3153.44 435.029 10

-17 1 -9 4177.72 361.005 1

16 -17 -9 4345.67 357.029 6

-17 1 -9 4501.65 363.721 11

1 16 -9 4166.74 356.834 2

1 16 -12 1002.62 97.9697 2

-17 1 -12 1069.80 101.104 11

-1 -16 12 593.72 146.83 10

1 16 -12 731.973 185.297 7

16 -17 -12 757.929 92.9114 8

1 16 -12 1016.02 91.8183 14

-17 1 -12 439.517 86.0024 14

1 16 -15 527.146 56.7748 2

16 -17 -15 306.085 50.0373 8

-17 17 -16-5.33412 19.7263 11

0 -17 -16 1.75478 23.9023 8

-17 17 -16 34.3313 31.0317 14

17 0 -16 7.87014 18.5777 2

0 -17 -13 40.9662 38.892 6

-17 17 -13 108.459 46.9715 14

0 -17 -13 26.9159 41.3053 8

-17 17 -13 3.19251 36.4591 11

-17 17 -13-5.26872 58.8163 2

-17 17 -10 238.31 67.4285 11

-17 17 -10 201.501 81.8261 2

-17 17 -10 205.117 63.8844 14

17 -17 10 134.172 82.4772 10

-17 0 10 150.497 120.971 10

17 0 -10 250.545 94.93 9

0 -17 -10 243.899 62.5193 8

-17 17 -10 147.396 71.1974 3

0 -17 -10 232.855 56.8736 6

17 0 -10 153.404 80.7342 7

17 -17 7 1945.78 275.34 10

17 0 -7 3099.58 323.505 9

-17 0 7 3555.55 429.436 10

0 17 7 2441.07 302.277 4

0 -17 -7 2921.90 243.781 8

-17 17 -7 2861.32 245.317 2

-17 17 -7 2569.93 236.908 13

0 -17 -7 2886.48 236.286 1

-17 17 -7 1953.96 323.461 4

-17 17 -7 2377.65 238.199 3

0 -17 -7 2881.45 238.226 6

-17 17 -7 2685.47 241.882 14

-17 17 -4 6077.57 525.243 3

-17 17 -4 6203.69 790.275 4

-17 0 4 7289.38 658.635 10

0 -17 -4 6655.80 524.475 8

-17 17 -4 6145.88 523.13 2

0 17 4 6852.56 636.399 4

0 -17 -111760.60 940.955 6

0 17 110674.40 1024.65 9

-17 17 -111295.40 941.051 3

0 17 112804.90 1002.80 7

0 -17 210842.00 859.758 10

17 0 2 9592.46 839.225 9

-17 0 -2 8993.47 779.541 5

0 -17 5 13.8838 95.3685 10

0 17 -5 128.356 175.82 9

17 -17 -5 19.3825 32.4499 8

-17 17 5 -19.313 124.891 4

0 17 -5-54.6305 172.045 4

17 -17 -5 27.0326 34.3102 6

-17 0 -5-10.4414 33.243 1

0 17 -5 52.0496 35.7068 2

0 17 -5-61.4505 206.182 7

0 17 -8 349.339 68.8943 14

0 -17 8 218.948 115.459 10

0 17 -8 318.85 147.206 9

17 -17 -8 331.347 61.3034 8

0 17 -8 435.41 64.9701 2

-17 0 -8 382.135 63.569 1

0 17 -8 676.365 202.139 7

-17 0 -8 356.351 72.2568 11

0 17 -8 318.502 116.766 4

17 -17 -8 361.533 62.0718 6

17 -17 -11 274.85 55.4894 8

-17 0 -11 312.015 64.175 11

0 17 -11 269.328 59.9843 2

0 17 -11 270.715 51.2908 11

0 17 -11 263.262 51.1461 14

0 17 -11 142.61 127.307 7

0 -17 11 224.358 112.223 10

0 17 -14 285.893 49.1554 2

0 17 -14 156.175 38.5006 14

17 -17 -14 202.454 43.6048 8

17 -17 -17 114.706 22.3801 8

0 17 -17 183.868 27.9427 2

0 17 -17 117.509 26.1991 3

18 -17 -16 42.8742 21.6999 8

-1 18 -16 52.2396 26.5135 2

-1 18 -16 67.8372 26.2226 3

-1 18 -13 459.334 60.0423 14

18 -17 -13 400.542 62.0554 8

-1 18 -13 496.88 67.4884 2

1 -18 10 18.3264 105.708 10

-1 18 -10 78.6891 44.8756 2

18 -17 -10 20.5937 40.5095 8

-1 18 -10 9.40091 43.0594 14

-17 -1 -10 118.005 53.9093 11

-1 18 -10 19.5587 39.0762 11

1 -18 7 1880.80 240.372 10

-1 18 -7 985.304 258.223 9

18 -17 -7 1390.69 144.408 8

-1 18 -7 1965.76 335.551 7

-17 -1 -7 1646.14 148.154 1

-1 18 -7 1770.67 282.157 4

-18 17 7 1169.97 220.313 4

18 -17 -7 1355.11 143.126 6

-1 18 -7 1771.23 152.045 2

-1 18 -4 3427.87 460.814 7

1 -18 4 2630.70 271.505 10

-1 18 -4 2455.77 389.949 9

17 1 4 2258.63 229.228 9

18 -17 -4 2391.04 191.236 8

-18 17 4 2823.14 339.605 4

-1 18 -4 1733.61 373.997 4

-1 18 -4 1949.07 192.609 2

-1 18 -4 1932.34 194.208 3

-1 18 -111753.10 1103.16 7

-18 17 114469.30 1241.01 4

-17 -1 -112650.00 985.518 5

17 1 1 9794.12 1036.31 9

1 -18 -233707.10 2580.78 6

-18 17 -233874.90 2935.71 4

-1 18 235413.30 2760.24 4

-1 18 227076.20 2597.26 7

-18 17 -232978.40 2580.46 3

17 1 -5 4436.07 475.993 9

-17 -1 5 5440.57 555.484 10

1 -18 -5 4331.72 394.05 8

-18 17 -5 4710.90 395.792 2

-18 17 -5 3514.84 559.427 4

-18 17 -5 4666.70 396.459 3

1 -18 -5 5188.05 393.789 1

-1 18 5 4861.72 496.837 4

18 -17 8 3648.03 413.747 10

1 -18 -8 4429.97 371.276 6

17 1 -8 4234.44 436.52 9

-17 -1 8 4930.12 552.237 10

-18 17 -8 4466.39 380.314 2

1 -18 -8 4604.44 376.061 8

-18 17 -8 2944.20 430.696 4

-18 17 -8 4412.55 374.959 14

1 -18 -8 4809.40 369.869 1

-18 17 -8 3933.52 372.957 3

-18 17 -11 2642.30 222.991 11

1 -18 -11 2513.72 216.749 6

-1 18 11 1656.71 248.528 10

17 1 -11 2412.36 266.958 9

1 -18 -11 2616.15 221.737 8

17 1 -11 2153.12 271.958 7

-17 -1 11 1867.11 287.879 10

-18 17 -11 1840.12 240.848 3

-18 17 -11 2445.21 221.512 14

-18 17 -11 2458.06 244.676 2

1 -18 -14 22.2102 36.0757 8

1 -18 -14 9.74357 31.0591 6

-18 17 -14 4.24876 41.7844 2

-18 17 -14 79.8312 45.2017 14

-18 17 -14 10.2782 34.339 11

17 1 -14 39.2384 26.8725 2

-18 17 -17 119.44 18.3793 11

1 -18 -17 113.471 22.7362 8

-18 17 -17 65.2558 25.5954 14

-2 18 -17 142.165 27.0441 3

18 -16 -17 91.1428 21.4162 8

18 -16 -14 19.2621 31.2978 8

-2 18 -14 110.894 40.1855 2

-2 18 -14 122.172 38.116 3

-2 18 -14 52.8403 31.1021 14

-2 18 -11-51.2562 130.002 7

18 -16 -11-13.3334 41.9963 8

-2 18 -11 15.5173 32.1443 11

-2 18 -11-3.74058 44.5058 2

2 -18 11-16.8985 102.029 10

-2 18 -11 49.9685 41.2914 14

-2 18 -8-7.38353 48.5214 14

2 -18 8-52.2277 101.979 10

18 -16 -8 18.4821 41.3621 6

18 -16 -8 29.3322 41.0757 8

-2 18 -8 -32.951 204.716 7

-2 18 -8 32.7319 43.6119 2

-16 -2 -8 36.9691 45.1245 1

-2 18 -8-20.6483 100.289 4

-16 -2 -8 22.2836 52.4288 11

2 -18 5 958.028 165.798 10

16 2 5 837.11 116.451 9

-2 18 -5 448.982 265.109 9

18 -16 -5 615.862 91.7618 8

-2 18 -5 825.426 289.128 7

-16 -2 -5 1684.46 109.081 1

18 -16 -5 703.066 93.8584 6

-2 18 -5 1374.10 108.274 2

-2 18 -5 1076.81 301.864 4

-2 18 -5 1260.86 109.452 3

-18 16 5 771.344 188.143 4

-2 18 -216560.00 1259.62 7

-2 18 -212973.90 1057.45 2

-18 16 214381.10 1241.34 4

-2 18 -214139.20 1220.58 9

16 2 211850.20 1108.00 9

-2 18 -213335.60 1058.98 3

2 -18 214640.30 1126.09 10

-16 -2 -212556.00 1056.68 5

-2 18 -211973.70 1355.47 4

-2 18 1 8327.22 748.392 7

2 -18 -1 8470.81 708.479 6

-18 16 -1 9153.46 709.511 3

-16 -2 1 8489.47 708.754 5

-2 18 1 9528.29 923.728 4

16 2 -1 8467.73 786.742 9

-18 16 -4 3568.67 313.949 2

2 -18 -4 4370.46 319.994 6

-18 16 -4 3312.07 523.167 4

-18 16 -4 3916.92 318.436 3

2 -18 -4 3248.56 311.191 1

-2 18 4 4182.01 437.467 4

2 -18 -7 808.266 84.1374 1

-18 16 -7 841.838 111.848 11

-18 16 -7 822.771 93.8618 14

18 -16 7 549.913 124.622 10

-16 -2 7 1038.41 232.394 10

16 2 -7 815.513 149.779 9

2 -18 -7 698.70 91.1154 8

-18 16 -7 764.106 91.0695 2

-2 18 7 912.089 161.48 4

-18 16 -7 619.454 174.754 4

-18 16 -7 656.299 83.8601 3

-18 16 -10 126.13 61.2422 11

18 -16 10 11.9878 69.2317 10

-16 -2 10 173.046 135.017 10

16 2 -10 196.744 89.1748 9

2 -18 -10 177.354 55.9654 8

16 2 -10 134.374 98.6446 7

-18 16 -10 221.55 69.0473 3

2 -18 -10 237.615 56.8943 6

-18 16 -10 138.355 59.4805 14

-18 16 -10 213.562 74.2908 2

2 -18 -13 3.31039 42.0053 8

2 -18 -13 20.8943 39.6064 6

-18 16 -13 79.1892 43.3992 14

-18 16 -13 58.096 41.7657 11

-18 16 -13-28.6513 60.2336 2

16 2 -13-4.63657 30.9615 2

-18 16 -16 -24.259 31.9966 14

2 -18 -16-5.19004 23.4982 8

-18 16 -16 12.2452 20.9964 11

16 2 -16 -13.063 18.846 2

18 -15 -15 19.7353 26.5692 8

-3 18 -15 14.985 30.492 3

-3 18 -15-2.47145 30.8182 2

-3 18 -15-9.44422 28.2782 14

-3 18 -12 455.578 167.834 7

18 -15 -12 547.481 71.4109 8

-15 -3 -12 602.627 106.143 8

-3 18 -12 544.525 67.715 14

-3 18 -12 474.163 65.4837 11

3 -18 12 393.86 118.172 10

-3 18 -12 494.051 75.358 2

18 -15 -9 4643.77 401.376 8

-3 18 -9 4560.56 404.61 2

-3 18 -9 5147.72 407.449 14

-3 18 -9 4666.96 403.915 11

-15 -3 -9 5828.10 417.179 11

-15 -3 -9 4097.25 401.431 1

3 -18 9 4577.87 495.756 10

3 -18 6 5299.89 493.914 10

-3 18 -6 4666.43 630.366 9

18 -15 -6 5012.96 413.585 8

-3 18 -6 5190.83 419.014 2

-18 15 6 6336.85 533.689 4

-15 -3 -6 4552.14 412.13 1

-3 18 -6 5939.24 634.807 4

18 -15 -6 4637.54 412.095 6

-3 18 -3 5180.26 420.103 2

-18 15 3 5385.10 562.064 4

-3 18 -3 5027.80 771.356 4

-3 18 -3 5336.54 603.611 9

15 3 3 4112.63 452.019 9

-3 18 -3 5175.57 420.401 3

3 -18 3 4633.80 470.612 10

-3 18 -3 5880.98 647.869 7

-3 18 0 67.2832 82.5338 7

3 -18 0 149.89 39.6211 6

-3 18 0-38.5718 293.606 4

-18 15 0 70.50 270.813 4

15 3 0 111.886 101.706 9

-15 -3 0 75.8151 37.9066 5

3 -18 -328625.90 2344.39 6

-15 -3 332881.90 2466.78 10

15 3 -330006.00 2435.75 9

-18 15 -328531.50 2600.54 4

-3 18 329823.80 2475.32 4

3 -18 -6 59.2784 39.8798 6

18 -15 6 67.1672 71.5824 10

-15 -3 6 48.1737 141.149 10

15 3 -6 28.3575 90.6028 9

3 -18 -6 58.2145 38.4829 8

-18 15 -6-23.8094 150.02 4

3 -18 -6 21.9664 31.7562 1

-18 15 -6 71.8264 37.4042 2

-3 18 6-15.8843 112.838 4

-18 15 -6 84.052 34.3496 3

-18 15 -9 7244.53 602.556 11

-18 15 -9 7879.69 601.897 14

18 -15 9 6274.09 641.318 10

-15 -3 9 7613.08 736.47 10

15 3 -9 7569.22 659.411 9

-18 15 -9 6184.59 594.106 3

15 3 -9 7657.73 681.678 7

-18 15 -9 6820.36 604.596 2

3 -18 -9 7556.84 596.324 8

15 3 -12 966.552 96.3459 2

3 -18 -12 945.393 106.871 8

-18 15 -12 1079.06 107.431 11

-18 15 -12 888.851 103.348 14

-3 18 12 817.865 133.743 10

18 -15 12 627.293 132.10 10

-15 -3 12 704.047 156.627 10

-18 15 -12 841.392 131.105 2

15 3 -12 846.252 151.396 7

3 -18 -12 1006.90 105.807 6

3 -18 -15 41.8959 31.435 8

-18 15 -15 83.1142 31.4908 11

-18 15 -15 147.426 38.8001 14

15 3 -15 60.686 25.8358 2

18 -14 -16 138.641 27.4079 8

-4 18 -16 140.929 32.4806 2

-4 18 -16 121.64 31.5005 3

-4 18 -16 73.4251 29.4525 14

-4 18 -13 346.709 56.9129 11

-4 18 -13 559.261 73.3834 2

18 -14 -13 501.685 64.0885 8

-14 -4 -13 450.298 82.908 8

4 -18 13 238.875 97.2838 10

14 4 13 484.55 128.598 10

-4 18 -13 563.984 63.5328 14

-4 18 -13 501.909 68.91 3

4 -18 10 628.198 172.867 10

-4 18 -10 1013.40 268.336 7

18 -14 -10 1146.51 121.926 8

-14 -4 -10 1374.50 169.849 8

-4 18 -10 1384.02 127.34 14

-14 -4 -10 1175.98 129.658 1

-4 18 -10 1270.27 124.538 11

-4 18 -10 1116.28 128.512 2

-4 18 -7 8205.31 845.024 7

-4 18 -7 5406.18 624.191 4

18 -14 -7 5150.50 472.14 8

-18 14 7 6146.24 566.081 4

4 -18 7 6376.29 565.751 10

-14 -4 -7 5476.20 473.527 1

-14 -4 -7 7437.05 491.546 11

-4 18 -7 5187.57 476.281 2

-4 18 -4 7917.44 632.867 3

-18 14 4 6927.94 732.215 4

-4 18 -4 8325.99 870.211 9

14 4 4 7158.40 660.32 9

4 -18 4 8153.16 688.515 10

-4 18 -4 9283.42 1050.39 4

-4 18 -4 7372.97 631.052 2

14 4 1 2185.88 263.977 9

-4 18 -1 2625.06 296.533 7

-18 14 1 2454.08 399.261 4

-4 18 259201.70 4560.49 4

-14 -4 256056.10 4397.91 5

4 -18 -254791.70 4395.97 6

-18 14 -252665.30 4643.59 4

-4 18 251446.80 4413.88 7

14 4 -254672.00 4475.39 9

-14 -4 261247.70 4508.80 10

-14 -4 515077.60 1152.12 10

14 4 -513646.00 1089.24 9

4 -18 -513155.80 999.951 8

4 -18 -513163.90 1000.83 6

-18 14 -511895.40 996.691 2

4 -18 -512321.00 996.037 1

18 -14 511511.80 1051.29 10

-18 14 -511209.40 996.279 3

-4 18 513310.20 1110.61 4

-18 14 -510393.10 1141.01 4

18 -14 8 602.177 125.788 10

-14 -4 8 1005.94 238.984 10

14 4 -8 687.912 133.641 9

-18 14 -8 872.647 96.70 14

-18 14 -8 586.941 78.5168 3

-18 14 -8 628.508 86.9572 2

4 -18 -8 688.905 86.2299 8

14 4 -11 122.374 89.8928 7

-18 14 -11 75.5914 52.3253 11

-18 14 -11 181.743 58.1668 14

-4 18 11 74.4975 67.4239 10

18 -14 11 85.0321 72.6199 10

-14 -4 11 128.577 112.753 10

-18 14 -11-7.23068 73.3353 2

4 -18 -11 93.3814 54.241 8

-18 14 -14 104.786 41.2989 11

14 4 -14 165.625 37.3064 2

-18 14 -14 270.508 50.7163 14

-18 14 -14 104.609 53.6779 2

4 -18 -14 120.75 42.8345 8

4 -18 -17 29.8396 20.1111 8

-5 18 -17-6.29966 22.1322 2

-5 18 -17 1.48298 20.557 3

18 -13 -17-15.6577 15.5175 8

18 -13 -14 996.773 98.8671 8

-5 18 -14 1231.49 110.004 2

-5 18 -14 1022.00 99.8697 14

-5 18 -14 840.68 92.591 11

-5 18 -14 1053.48 104.842 3

18 -13 -11 3580.63 310.64 8

-5 18 -11 3923.69 490.183 7

-5 18 -11 3277.99 315.303 2

-5 18 -11 3900.94 314.355 14

5 -18 11 2464.52 371.453 10

-5 18 -11 3408.20 311.73 11

18 -13 -8 103.043 43.4693 8

-5 18 -8 126.758 103.314 4

-5 18 -8 330.562 60.3878 14

-18 13 8 60.9114 97.0983 10

5 -18 8 98.6009 97.0145 10

-13 -5 -8 127.217 47.0258 1

-5 18 -8 170.573 53.8685 2

18 -13 -5 504.159 59.2072 8

-18 13 5 833.269 184.061 4

-5 18 -5 407.866 66.0301 3

-5 18 -5 313.031 62.1195 2

13 5 5 801.361 98.3529 9

5 -18 5 769.275 133.377 10

-18 13 5 322.832 79.4964 10

-5 18 -5 407.029 309.33 4

-5 18 -2 1094.61 105.726 2

-5 18 -2 1048.74 104.657 3

-18 13 2 1119.91 240.352 4

-5 18 -2 1052.75 551.402 4

5 -18 2 1093.19 146.786 10

13 5 2 844.859 149.04 9

-5 18 -2 999.245 203.208 7

-5 18 1 507.065 240.592 4

13 5 -1 563.153 134.07 9

-5 18 1 259.707 81.0372 7

-13 -5 1 627.647 66.1407 5

5 -18 -4 8137.94 668.087 6

-18 13 -4 7487.70 840.618 4

-5 18 4 8878.85 792.379 4

-18 13 -7 1236.49 224.549 4

5 -18 -7 1604.66 146.147 6

-13 -5 7 1740.49 290.036 10

18 -13 7 1337.50 191.609 10

13 5 -7 1580.68 209.086 9

5 -18 -7 1564.02 145.146 8

-5 18 7 1347.57 218.841 4

-18 13 -7 1200.69 144.788 14

5 -18 -7 1897.96 148.075 1

-18 13 -7 1317.93 140.717 2

18 -13 10 4892.22 498.796 10

-5 18 10 4535.32 483.506 10

13 5 -10 5726.40 506.378 9

5 -18 -10 5859.70 450.098 8

13 5 -10 5517.18 532.32 7

-18 13 -10 4866.07 447.714 14

-13 -5 10 4884.76 548.756 10

-18 13 -10 5666.67 451.752 11

-18 13 -10 4785.44 459.523 2

13 5 -13 997.491 85.0459 2

5 -18 -13 844.137 91.4985 8

-18 13 -13 699.093 87.1632 11

-18 13 -13 740.961 88.2639 14

18 -13 13 402.583 105.469 10

-13 -5 13 445.423 122.392 10

-18 13 -13 542.136 105.904 2

13 5 -13 485.333 119.122 7

5 -18 -13 850.444 90.6694 6

5 -18 -16 20.2754 25.6821 8

13 5 -16 3.08146 21.3506 2

-18 13 -16-38.2986 32.6991 14

-18 13 -16 30.3977 22.2372 11

-6 18 -15 140.979 36.9286 3

-6 18 -15 127.135 40.8515 2

18 -12 -15 90.1885 31.0244 8

-6 18 -15 153.868 37.3859 14

-6 18 -12 54.7995 38.8743 14

-6 18 -12-21.5386 35.9151 11

-6 18 -12 11.4809 44.6645 2

6 -18 12 -44.342 82.4209 10

-6 18 -12 40.2789 48.1659 3

18 -12 -12 5.91089 35.96 8

-12 -6 -12 5.25721 57.6154 8

-6 18 -9 70.1784 48.4576 2

-12 -6 -9 21.8722 39.4797 6

-6 18 -9 26.9109 52.5982 14

-6 18 -9 32.0379 45.3392 11

-12 -6 -9 35.2989 75.6772 8

18 -12 -9 54.6394 39.5031 8

-18 12 9 18.6079 96.3851 10

6 -18 9 33.8169 94.3314 10

-18 12 6 1646.62 284.647 10

-6 18 -6 3723.71 635.771 7

18 -12 -6 2334.90 228.57 8

-6 18 -6 2946.05 456.386 4

-18 12 6 3248.04 339.781 4

-12 -6 -6 2758.56 233.22 1

-12 -6 -6 2850.77 229.775 6

-6 18 -6 2781.41 239.477 2

-6 18 -6 2898.23 245.756 3

6 -18 6 2500.46 296.187 10

-6 18 -3 6095.78 483.527 2

-6 18 -3 5599.57 1118.69 4

-18 12 3 5801.50 603.207 4

-6 18 -3 6411.42 642.302 7

12 6 3 5015.91 519.491 9

6 -18 3 5359.70 519.162 10

-6 18 -3 5894.22 482.565 3

-6 18 0 964.633 143.169 7

-6 18 0 1474.03 378.176 4

-18 12 0 875.623 292.55 4

12 6 0 1030.45 165.982 9

6 -18 -3 479.518 61.4662 6

-6 18 3 413.788 181.925 4

-18 12 -3 472.654 233.786 4

6 -18 -621084.00 1618.32 8

12 6 -623376.80 1705.66 9

-18 12 -619568.40 1740.37 4

6 -18 -620754.00 1617.03 1

6 -18 -620539.60 1618.56 6

-6 18 621721.90 1729.26 4

-18 12 -617834.40 1613.30 2

-12 -6 9 4074.23 509.408 10

12 6 -9 4624.09 455.439 9

12 6 -9 4468.88 483.556 7

-18 12 -9 4958.88 405.22 11

6 -18 -9 4506.31 395.066 6

-18 12 -9 4416.42 403.283 2

-18 12 -9 5313.64 406.924 14

-6 18 9 4064.72 434.661 10

18 -12 9 4294.38 452.765 10

12 6 -12 364.215 111.871 7

12 6 -12 254.892 54.402 2

18 -12 12 234.326 86.6748 10

-12 -6 12 207.641 108.984 10

-18 12 -12 491.457 68.3958 14

-18 12 -12 166.373 83.6815 2

-18 12 -12 447.986 65.9979 11

-18 12 -15 32.1308 36.3585 14

12 6 -15 43.6143 26.5941 2

-18 12 -15-2.07321 27.3879 11

-7 18 -16 197.415 36.5439 3

-7 18 -16 172.361 38.9755 14

18 -11 -16 246.286 33.6234 8

-7 18 -13 1027.91 106.275 2

-7 18 -13 927.29 105.606 3

18 -11 -13 950.423 95.3206 8

-11 -7 -13 1102.62 112.646 8

-7 18 -13 817.944 96.2093 14

-7 18 -13 883.988 93.3635 11

7 -18 13 523.104 129.506 10

11 7 13 600.134 145.194 10

-7 18 -10 2730.97 255.397 14

18 -11 -10 2527.24 245.293 8

-11 -7 -10 3816.90 281.015 8

-7 18 -10 2730.22 253.349 11

-11 -7 -10 3178.42 252.431 6

-7 18 -10 3529.23 513.479 7

-18 11 10 1903.04 304.668 10

7 -18 10 2585.05 331.256 10

-7 18 -10 2863.81 257.945 2

-11 -7 -7 170.398 42.0086 6

18 -11 -7 31.2919 33.2288 8

-7 18 -7 205.974 355.518 7

-7 18 -7 67.4602 42.3243 13

7 -18 7 85.3108 95.6568 10

-18 11 7 45.732 129.861 10

-7 18 -7 122.511 47.5686 2

-7 18 -7 83.936 132.934 4

-7 18 -412156.80 921.753 3

-18 11 412518.60 1040.17 4

-7 18 -411355.20 921.038 2

11 7 413013.80 964.353 9

7 -18 412523.90 968.058 10

-18 11 4 6908.12 937.714 10

-7 18 -417353.10 1208.75 7

-7 18 -416579.50 1721.57 4

-7 18 -1 131.267 89.1278 7

-18 11 1 155.828 187.547 4

11 7 1 571.483 117.933 9

11 7 -2 9396.13 851.278 9

-7 18 2 9239.86 926.467 4

-11 -7 210376.40 876.751 10

-18 11 -2 8470.94 959.231 4

7 -18 -513938.60 1151.81 6

11 7 -515230.10 1239.73 9

-7 18 514715.40 1269.08 4

-18 11 -514886.00 1301.04 4

-18 11 -8 228.274 60.1884 14

11 7 -8 224.689 45.632 2

-18 11 -8 168.259 49.1846 2

11 7 -8 189.551 114.06 7

-7 18 8 150.889 72.5675 10

7 -18 -8 298.803 56.7648 6

11 7 -8 126.535 80.3452 9

11 7 -11 274.53 116.957 7

7 -18 -11 287.093 63.3944 6

18 -11 11 159.609 91.5885 10

-11 -7 11 108.573 117.315 10

-18 11 -11 322.818 65.0816 11

-18 11 -11 331.867 62.6849 14

11 7 -11 220.422 51.4887 2

-18 11 -11 219.927 79.7376 2

-18 11 -14 519.272 67.0665 11

-18 11 -14 566.794 69.708 14

11 7 -14 477.929 62.3296 2

11 7 -17 14.4572 17.9845 2

-8 18 -17 23.2987 25.7673 14

-10 -8 -14 513.695 71.2118 8

18 -10 -14 446.101 60.8752 8

-8 18 -14 408.484 65.9327 3

-8 18 -14 477.878 58.0739 11

-8 18 -14 635.072 69.1746 14

-18 10 11 127.947 84.245 10

8 -18 11 184.529 100.069 10

-10 -8 -11 253.075 54.3457 6

18 -10 -11 268.838 50.4598 8

-10 -8 -11 309.04 69.963 8

-8 18 -11 234.694 61.6626 14

-8 18 -11 222.863 74.1054 3

-8 18 -11 263.212 56.3894 11

-8 18 -11 228.981 66.3518 2

8 -18 813967.40 1302.51 10

-10 -8 -815693.00 1227.95 6

-18 10 813950.30 1357.40 10

-8 18 -814794.70 1234.78 2

-10 -8 -819346.80 1256.21 8

18 -10 -813436.90 1222.09 8

-8 18 -816293.00 1248.52 3

-8 18 -815305.20 1236.73 11

-8 18 -815865.60 1233.54 14

-8 18 -811560.80 1308.38 4

10 8 5 2784.40 268.31 9

-8 18 -5 2466.47 237.697 2

-8 18 -5 2508.84 549.633 4

-18 10 5 3056.13 341.409 4

-18 10 5 2508.86 303.874 10

8 -18 5 3291.65 296.61 10

-10 -8 -5 2656.06 229.899 6

-18 10 2 2629.99 358.563 4

10 8 2 1925.11 259.583 9

-8 18 -2 1666.00 277.074 7

8 -18 2 1838.28 234.845 10

-8 18 -2 3156.67 221.761 2

-8 18 -2 2574.63 218.327 3

10 8 -1 4621.56 463.011 9

-8 18 1 4528.88 584.642 4

-10 -8 4 4087.07 408.059 10

-18 10 -4 2614.24 418.367 4

8 -18 -4 3294.50 282.896 6

-8 18 4 4051.39 427.929 4

10 8 -4 3097.32 359.777 9

8 -18 -7 5955.51 504.679 6

-8 18 7 6196.00 603.495 4

-18 10 -7 5478.22 498.664 2

10 8 -7 6906.98 501.89 2

-8 18 7 5456.48 539.694 10

10 8 -7 6428.98 575.794 9

10 8 -10 181.122 87.6989 9

8 -18 -10 255.429 64.5869 8

-18 10 -10 220.436 60.0749 11

-18 10 -10 210.459 63.4995 14

8 -18 -10 297.913 66.806 6

10 8 -10 308.815 54.7173 2

-18 10 -10 180.845 68.4554 2

10 8 -10 181.734 126.191 7

18 -10 10 180.848 105.515 10

-8 18 10 166.363 76.0654 10

8 -18 -13 825.656 91.6826 8

10 8 -13 646.425 138.545 7

-18 10 -13 695.87 88.3885 14

18 -10 13 426.596 116.844 10

-10 -8 13 447.122 124.056 10

10 8 -13 1066.23 89.1378 2

-18 10 -13 783.894 89.3973 11

-18 10 -13 516.047 107.381 2

10 8 -16 327.862 39.5736 2

8 -18 -16 258.747 41.5328 8

-18 10 -16 336.487 39.3464 11

-9 18 -15 62.2106 34.3758 3

18 -9 -15 77.9641 29.1744 8

-9 18 -15 140.414 29.3736 11

-9 -9 -12 179.361 47.4996 6

-9 18 -12 193.086 49.0336 11

-9 -9 -12 283.153 65.7475 8

18 -9 -12 219.592 46.2278 8

-9 18 -12 254.161 58.3209 14

-9 18 -12 212.281 68.0119 3

9 -18 12 98.1314 85.0322 10

-18 9 9 5597.57 634.198 10

-9 -9 -9 5790.92 528.394 1

-9 18 -9 7107.56 538.914 14

18 -9 -9 5866.63 521.633 8

-9 -9 -9 8000.63 548.713 8

-9 18 -9 6133.46 549.827 3

-9 -9 -9 6309.82 528.414 6

9 -18 9 5587.15 597.072 10

-9 18 -9 6516.30 536.783 11

-9 -9 -6 2494.91 217.948 1

-18 9 6 2431.42 334.748 10

9 -18 6 2196.36 267.796 10

-9 -9 -6 2435.60 214.575 6

-9 18 -6 2793.91 454.369 4

-18 9 3 5236.77 500.64 4

-9 18 -3 5869.72 1312.63 4

9 -18 3 3779.52 375.727 10

-18 9 3 2650.66 380.104 10

9 9 3 4405.56 404.59 9

-9 18 -3 4538.26 353.822 3

-9 18 -3 4507.40 354.518 2

-9 18 -3 3987.51 456.231 7

-18 9 026444.20 2509.98 4

9 9 028139.90 2408.93 9

-9 -9 315231.30 1381.04 10

-9 18 317831.10 1455.83 4

-18 9 -315499.40 1473.03 4

-9 -9 6 669.04 180.276 10

-9 18 6 591.303 101.756 10

9 9 -6 565.093 66.6659 2

-9 18 6 423.502 162.57 4

9 -18 -6 617.258 74.6663 6

9 -18 -6 624.989 72.3849 8

9 9 -6 507.784 122.445 9

9 9 -9 490.633 116.377 9

9 -18 -9 466.025 77.0192 8

-18 9 -9 593.72 82.5162 14

-18 9 -9 518.513 83.2079 11

9 9 -9 517.399 143.292 7

9 -18 -9 466.428 75.135 6

-9 18 9 534.909 100.441 10

-9 -9 9 494.949 160.699 10

-18 9 -9 393.573 70.3544 2

9 9 -9 490.377 68.3225 2

9 -18 -12 39.3356 50.9453 8

9 9 -12 51.059 82.8492 7

-18 9 -12 18.5578 47.8683 11

-18 9 -12 128.371 52.8414 14

18 -9 12 148.971 94.2353 10

9 -18 -12 102.925 54.7476 6

-18 9 -12 59.0334 73.5021 2

9 9 -12 69.6902 43.8358 2

-18 9 -15 106.105 40.8692 14

9 -18 -15 223.059 41.419 8

9 9 -15 210.845 38.0415 2

-18 9 -15 194.47 37.9891 11

9 -18 -18 7.24585 13.9537 8

-10 18 -16 108.657 37.32 14

-10 18 -16 167.546 36.4114 2

-10 18 -16 122.388 23.6113 11

-8 -10 -13 354.716 60.8936 8

18 -8 -13 335.15 49.9491 8

-10 18 -13 465.763 62.0728 14

-10 18 -13 206.084 60.0607 3

-10 18 -13 281.992 52.4301 11

10 -18 13 262.753 94.1703 10

8 10 13 279.602 103.089 10

-18 8 10 342.356 135.472 10

10 -18 10 345.725 122.343 10

-10 18 -10 477.957 82.254 14

-8 -10 -10 602.044 76.4807 6

-8 -10 -10 634.632 82.8334 1

-10 18 -10 511.763 99.2406 3

-10 18 -10 482.087 78.1864 11

18 -8 -10 438.848 63.4695 8

-8 -10 -10 614.407 86.1294 8

-10 18 -7 6750.77 533.12 11

10 -18 7 5975.94 582.659 10

-8 -10 -7 5755.22 516.453 1

-18 8 7 6035.33 669.22 10

-10 18 -7 7846.36 979.491 7

-10 18 -7 6041.81 530.813 3

-8 -10 -7 6977.76 533.314 8

-10 18 -7 4904.71 638.104 4

-10 18 -7 6376.35 521.78 13

-10 18 -7 6567.05 522.723 14

-8 -10 -7 5962.32 515.305 6

-10 18 -448846.00 3709.23 7

-8 -10 -444646.40 3542.71 6

-18 8 452228.20 3686.39 4

-10 18 -452968.20 4275.96 4

10 -18 442353.30 3578.16 10

8 10 445295.20 3591.19 9

-18 8 437708.50 3604.80 10

-18 8 1 213.763 208.912 4

8 10 1 24.3246 90.8818 9

-18 8 -252451.90 4345.22 4

8 10 -248367.80 4231.20 9

-10 18 253852.40 4324.57 4

-8 -10 254141.10 4237.45 10

-10 18 5 6932.83 662.198 4

-10 18 5 6245.03 560.483 10

10 -18 -5 6477.75 528.713 6

10 -18 -5 6161.42 526.851 8

8 10 -5 6923.90 627.525 9

-8 -10 5 7074.89 645.453 10

8 10 -8 832.79 158.988 9

10 -18 -8 924.389 99.6641 8

8 10 -8 911.373 95.017 2

10 -18 -8 887.083 102.93 6

-10 18 8 771.215 124.15 10

-8 -10 8 930.175 198.954 10

-18 8 -8 809.796 107.75 14

8 10 -8 1223.19 202.627 7

10 -18 -11 774.673 97.4868 8

10 -18 -11 881.22 101.509 6

8 10 -11 749.585 170.074 7

-18 8 -11 815.292 97.1469 14

-18 8 -11 822.418 100.061 11

18 -8 11 718.696 146.20 10

-8 -10 11 574.645 166.091 10

8 10 -11 1033.65 97.5895 2

-18 8 -11 516.371 107.02 2

-18 8 -14 93.7362 42.077 14

-18 8 -14 99.3482 39.307 11

8 10 -14 159.433 39.6751 2

10 -18 -14 105.546 40.3525 8

8 10 -17 103.457 23.8487 2

10 -18 -17 92.8716 24.2193 8

-11 18 -17 36.5992 27.2966 14

-11 18 -17 97.7893 26.3125 2

18 -7 -17 81.9845 20.739 2

-11 18 -14-13.5742 44.787 2

-11 18 -14 136.999 48.7048 14

-11 18 -14 20.935 30.4643 11

-11 18 -14 23.4872 42.7702 3

18 -7 -14 30.1083 29.0469 8

-7 -11 -14 34.8968 40.8338 8

-11 18 -11 198.226 55.2745 11

-18 7 11 78.2808 94.3214 10

11 -18 11 197.731 91.3845 10

18 -7 -11 228.215 94.2665 9

-11 18 -11 104.156 77.8821 3

18 -7 -11 206.10 42.8905 8

-7 -11 -11 179.192 61.6091 8

-11 18 -11 155.34 64.6816 14

-11 18 -11 206.163 65.4221 2

-7 -11 -11 222.061 54.1369 6

-11 18 -8 1410.19 137.69 11

-11 18 -8 1774.58 137.159 14

-18 7 8 1359.15 259.36 10

11 -18 8 1237.12 188.069 10

-7 -11 -8 780.527 114.716 1

-7 -11 -8 1458.75 135.509 8

-11 18 -8 1122.32 131.253 2

-11 18 -8 916.275 190.53 4

-11 18 -8 1117.60 139.815 3

-7 -11 -8 1099.89 116.34 6

-11 18 -5 1031.61 113.295 3

-11 18 -5 792.478 373.159 4

-7 -11 -5 1177.15 108.392 6

11 -18 5 1012.28 147.272 10

-18 7 5 932.971 202.351 10

7 11 5 1155.04 151.04 9

-11 18 -2 843.375 699.903 4

7 11 2 459.926 121.783 9

-18 7 2 423.277 215.723 4

-7 -11 1 7147.79 671.668 10

7 11 -1 6674.97 678.914 9

-11 18 4 2387.21 349.028 4

7 11 -4 2642.82 320.944 9

11 -18 -4 2395.33 217.645 6

-7 -11 4 3290.13 332.043 10

-7 -11 7 6812.37 634.435 10

7 11 -7 5999.96 583.058 9

11 -18 -7 5468.69 501.977 8

7 11 -7 7087.10 504.502 2

11 -18 -7 5978.93 506.506 6

-11 18 7 5146.52 533.988 10

-11 18 7 5765.65 594.268 4

7 11 -10 113.472 77.4369 9

11 -18 -10 176.823 60.4165 8

7 11 -10 99.0678 105.57 7

-18 7 -10 97.9666 53.5695 14

-18 7 -10 123.558 54.4577 11

-7 -11 10 117.959 121.266 10

7 11 -10 292.173 50.3582 2

11 -18 -10 258.174 57.7483 6

-18 7 -13 1183.22 127.72 14

-18 7 -13 1482.18 133.646 11

7 11 -13 1544.77 131.771 2

11 -18 -13 1246.70 131.60 8

7 11 -13 1158.44 200.569 7

18 -7 13 1149.90 181.565 10

-7 -11 13 975.457 181.477 10

11 -18 -16 14.9037 26.0209 8

7 11 -16 31.887 24.8184 2

-6 -12 -15 116.854 37.8282 8

-12 18 -15 84.9968 37.3525 2

-12 18 -15 220.637 47.4469 14

-12 18 -15 114.517 28.8146 11

-18 6 12 208.615 90.5115 10

12 -18 12 206.735 85.3983 10

-6 -12 -12 254.671 53.1227 6

-12 18 -12 225.331 67.3898 2

-12 18 -12 137.127 58.6016 14

-6 -12 -12 296.982 62.9293 8

18 -6 -12 230.221 43.6895 8

-12 18 -12 205.717 52.3449 11

-12 18 -12 209.038 76.7707 3

-18 6 9 3732.61 502.256 10

-12 18 -9 4793.90 410.307 2

-6 -12 -9 5164.59 406.207 8

-12 18 -9 4646.49 419.602 3

-12 18 -9 4996.49 406.266 14

-12 18 -9 4824.59 406.09 11

12 -18 9 3723.53 451.422 10

-6 -12 -9 4593.19 395.779 1

-6 -12 -9 4339.11 393.47 6

-6 -12 -6 231.249 55.7874 8

-12 18 -6 133.024 169.039 4

-12 18 -6 179.508 49.8593 3

-18 6 6 395.426 172.001 10

12 -18 6 244.134 80.7395 10

-6 -12 -6 110.98 39.446 6

-6 -12 -6 57.5177 39.4523 1

-12 18 -6 124.619 46.6527 2

-6 -12 -3 48.7267 36.7602 6

-12 18 -3 405.535 629.868 4

6 12 3 10.4902 72.1739 9

12 -18 3 94.4946 43.4131 10

-18 6 3-13.4061 88.9838 10

-12 18 -3 26.1353 34.5972 2

-12 18 -3 86.3241 37.506 3

-18 6 010257.50 1049.56 4

6 12 0 9433.28 907.051 9

-12 18 3 540.40 223.262 4

-6 -12 3 626.21 154.529 10

-6 -12 6 6075.61 583.685 10

12 -18 -6 5684.86 474.061 8

6 12 -6 6043.96 575.285 9

6 12 -6 6144.15 582.065 7

6 12 -6 6037.40 473.149 2

-18 6 -6 5364.41 472.458 1

-12 18 6 5898.95 589.25 4

12 -18 -6 5530.60 476.505 6

6 12 -9 1274.26 203.513 9

12 -18 -9 1668.38 157.618 8

-18 6 -9 1157.39 150.223 14

12 -18 -9 1974.51 163.599 6

6 12 -9 1168.79 233.307 7

-18 6 -9 1912.58 167.134 11

-6 -12 9 1257.69 243.755 10

6 12 -9 1843.29 155.843 2

6 12 -12 263.832 54.4091 2

12 -18 -12 178.487 57.8241 8

18 -6 12 192.054 94.9074 10

-6 -12 12 207.073 107.249 10

12 -18 -12 176.122 59.1509 6

6 12 -12 246.203 125.424 7

-18 6 -12 248.957 59.8971 11

6 12 -12 240.933 52.6598 14

-18 6 -12 311.415 57.9459 14

12 -18 -15 214.928 43.3036 8

6 12 -15 264.418 42.8616 2

-13 18 -16 304.068 43.944 14

-13 18 -16 292.894 43.5436 2

-13 18 -16 210.99 32.0283 11

18 -5 -16 270.593 36.3867 2

-5 -13 -13 122.946 40.2247 6

-13 18 -13 236.787 53.7033 14

-13 18 -13 91.3311 61.2552 3

-13 18 -13 151.055 41.4529 11

5 13 13 76.6305 76.52 10

-18 5 13 135.894 70.6612 10

13 -18 13 69.0125 66.9418 10

-13 18 -13 80.7124 48.8518 2

-5 -13 -13 109.977 48.9039 8

18 -5 -13 84.6905 32.2091 8

-5 -13 -10-10.2913 45.6354 6

-13 18 -10-39.6146 66.3014 2

-5 -13 -10 37.6792 54.0675 1

-13 18 -10 18.087 54.8086 11

-18 5 10 19.6477 107.654 10

13 -18 10 13.8439 82.3498 10

18 -5 -10-13.2902 76.6239 9

-13 18 -10 57.6271 60.8991 14

-5 -13 -10 12.9298 50.4971 8

-13 18 -10-15.2551 77.7893 3

18 -5 -10 91.2412 76.538 7

-13 18 -7 2334.26 216.299 13

-5 -13 -7 2295.82 213.539 1

-13 18 -7 2688.32 234.358 11

13 -18 7 2250.47 267.856 10

-18 5 7 3044.05 408.043 10

5 13 7 2064.46 260.322 4

-5 -13 -7 2659.15 224.582 8

-13 18 -7 2592.38 225.877 2

-5 -13 -7 2148.98 211.132 6

-13 18 -7 2342.00 224.465 3

-13 18 -7 2171.36 329.833 4

-13 18 -7 2626.49 221.101 14

5 13 4 785.125 147.51 9

-13 18 -4 1363.07 116.441 2

-18 5 4 1592.73 223.865 10

13 -18 4 805.995 126.962 10

-13 18 -4 1336.37 116.589 3

-13 18 -4 530.553 410.169 4

-5 -13 -4 1010.46 108.239 6

5 13 116223.50 1389.01 9

-5 -13 2 3819.43 393.964 10

-13 18 2 3232.11 499.498 4

13 -18 -5 7689.31 551.576 8

5 13 -5 7338.87 684.642 9

5 13 -5 6715.09 548.334 2

-13 18 5 8526.85 706.357 4

-18 5 -5 5377.47 545.209 1

-5 -13 5 8306.55 664.013 10

5 13 -5 8173.68 681.773 7

13 -18 -5 6880.27 553.051 6

13 -18 -828871.20 2539.44 8

13 -18 -832111.10 2542.94 6

5 13 -833392.90 2540.43 2

-18 5 -832550.00 2550.33 11

-5 -13 832322.80 2664.20 10

5 13 -831558.00 2614.23 4

5 13 -830797.70 2623.58 9

5 13 -1112369.30 924.333 2

5 13 -1111575.00 1039.93 7

-5 -13 11 8920.05 996.945 10

-18 5 -1112319.30 928.874 11

13 -18 -1111340.90 923.725 6

13 -18 -1110662.50 923.30 8

-18 5 -11 8956.40 916.532 14

5 13 -1113535.20 921.845 14

13 -18 -14 1811.07 167.218 8

5 13 -14 2079.99 167.918 2

-18 5 -14 2084.66 168.392 11

5 13 -17 14.7748 20.921 3

13 -18 -17 2.6144 19.6619 8

5 13 -17-7.99447 20.4174 2

-14 18 -17 116.904 26.3912 2

18 -4 -17 112.631 21.2829 2

-14 18 -17 98.3102 16.5935 11

-14 18 -17 85.4899 27.9453 14

-14 18 -14-13.4825 46.0668 3

-14 18 -14 78.731 46.7516 14

-14 18 -14-7.26879 43.8863 2

-4 -14 -14 3.01369 36.7903 8

-14 18 -14-12.2615 31.7152 11

-18 4 11 85.5413 93.0168 10

14 -18 11 117.104 87.3011 10

18 -4 -11 233.807 89.1359 9

-14 18 -11 132.98 68.5228 2

-14 18 -11 165.174 64.9466 14

-4 -14 -11 227.631 61.0242 8

-14 18 -11 82.0405 83.8741 3

-4 -14 -11 196.203 53.539 6

-14 18 -11 174.753 55.1174 11

14 -18 8 1971.99 271.287 10

-4 -14 -8 2560.14 222.431 6

-18 4 8 2493.48 375.201 10

-14 18 -8 2512.67 236.453 2

-4 -14 -8 2949.76 233.755 8

-14 18 -8 2552.44 237.892 3

-14 18 -8 2572.08 238.407 11

-4 -14 -8 2478.04 221.441 1

-14 18 -8 2501.80 229.539 14

-4 -14 -5 2639.87 213.95 8

-4 -14 -5 2378.38 209.132 6

-14 18 -5 1866.82 433.829 4

-14 18 -5 2261.26 211.912 3

-18 4 5 2904.52 357.844 10

-14 18 -5 2241.55 212.777 2

14 -18 5 2277.50 247.021 10

-14 18 -2 3138.00 808.191 4

4 14 2 3609.40 376.907 9

-4 -14 -2 3607.62 311.07 6

-14 18 -2 3713.11 311.211 2

-14 18 -2 3736.91 312.789 3

-14 18 127858.00 2312.32 4

4 14 -124586.80 2156.97 9

-14 18 4 5916.75 610.065 4

18 -4 4 5265.17 488.381 9

-4 -14 4 6565.25 554.618 10

4 14 -4 6590.14 588.472 7

4 14 -4 5645.54 609.241 9

14 -18 -4 5275.86 456.088 6

-4 -14 7 428.832 151.728 10

4 14 -7 388.139 133.691 9

14 -18 -7 448.908 60.7372 8

4 14 -7 453.335 168.609 7

-14 18 7 319.028 126.788 4

4 14 -7 429.858 124.921 4

14 -18 -7 398.653 64.3025 6

4 14 -7 278.438 51.6456 2

-18 4 -7 203.614 51.15 1

14 -18 -10 573.171 77.5701 8

14 -18 -10 567.281 75.8393 6

4 14 -10 341.934 138.87 7

4 14 -10 559.274 69.6144 14

-18 4 -10 343.714 67.1165 14

4 14 -10 650.53 78.0961 2

-18 4 -10 572.795 80.1931 11

-4 -14 10 231.03 133.979 10

14 -18 -13 208.525 50.1394 8

4 14 -13 323.197 117.176 7

18 -4 13 236.28 93.4958 10

-4 -14 13 41.3316 75.793 10

4 14 -13 259.794 46.6845 14

-18 4 -13 85.7958 46.3037 14

4 14 -13 265.709 51.8059 2

-18 4 -13 249.188 52.7042 11

14 -18 -16-10.4331 24.3568 8

4 14 -16-35.9874 26.5416 2

18 -3 -15 103.125 27.397 2

-15 18 -15 72.3186 36.2183 2

-3 -15 -15 85.8868 35.3809 8

-15 18 -15 102.90 31.1076 11

-3 -15 -12 214.411 56.772 8

-15 18 -12 137.736 76.5972 3

-18 3 12 164.846 94.3654 10

15 -18 12 118.827 79.3227 10

3 15 12 218.527 87.6206 10

-3 -15 -12 197.903 48.6515 6

-15 18 -12 238.169 55.3004 11

-15 18 -12 181.769 69.6839 2

-18 3 9 322.848 150.62 10

15 -18 9 143.251 94.1614 10

18 -3 -9 529.389 127.529 9

-3 -15 -9 421.244 73.9588 8

-15 18 -9 313.472 82.2539 3

-3 -15 -9 457.393 65.9463 1

-15 18 -9 255.335 74.3201 2

-15 18 -9 291.315 74.101 11

-3 -15 -9 509.553 69.701 6

-18 3 6 5088.86 529.408 10

3 15 6 3770.56 405.359 4

-3 -15 -6 4199.67 344.774 8

-15 18 -6 4068.47 344.013 3

-3 -15 -6 4282.48 339.471 1

-3 -15 -6 4026.38 338.509 6

-15 18 -6 4170.56 345.491 2

-15 18 -6 3517.45 492.494 4

-3 -15 -3 1723.65 153.374 6

3 15 3 1444.58 208.226 9

-15 18 -3 1573.59 151.088 2

-15 18 -3 1708.62 154.798 3

-18 3 3 1798.28 253.121 10

-15 18 -3 1927.80 593.972 4

3 15 0 8666.49 798.265 7

3 15 0 8299.49 778.656 9

-18 3 0 8089.07 683.834 5

-15 18 0 8675.85 687.543 3

-15 18 0 9572.26 1016.88 4

-3 -15 3 4318.53 392.162 10

-15 18 3 4166.78 481.644 4

3 15 -3 4047.22 443.049 7

-18 3 -3 3354.16 295.933 5

3 15 -3 3831.05 456.135 9

-3 -15 6 849.602 171.487 10

3 15 -6 697.742 194.90 9

15 -18 -6 612.056 80.1893 8

3 15 -6 780.142 81.9803 2

3 15 -6 608.882 173.229 4

-15 18 6 444.243 158.951 4

-18 3 -6 841.976 84.3338 1

3 15 -6 632.395 188.001 7

15 -18 -6 681.766 85.5961 6

15 -18 -9 324.24 65.5751 8

3 15 -9 549.804 68.8107 2

-18 3 -9 246.887 66.3538 11

3 15 -9 295.226 112.715 9

-3 -15 9 198.294 134.468 10

15 -18 -9 364.26 62.3042 6

15 -18 -12 800.93 86.3428 8

3 15 -12 933.938 85.9544 2

3 15 -12 882.427 187.16 7

-3 -15 12 331.538 122.80 10

18 -3 12 566.573 137.785 10

-18 3 -12 722.476 85.9462 11

-18 3 -12 311.563 71.1992 14

3 15 -12 742.219 77.2228 14

15 -18 -15 327.452 45.6692 8

3 15 -15 271.907 45.2338 2

-16 18 -16 5.15909 18.9837 11

18 -2 -16 1.30776 17.8136 2

-16 18 -16 6.30456 26.3845 2

-2 -16 -16 6.94049 23.9047 8

-2 -16 -13 43.8714 41.9158 8

-16 18 -13-48.5569 75.4026 3

-16 18 -13 29.1173 56.7231 2

-16 18 -13 68.6647 42.1416 11

-2 -16 -13 68.3461 39.1465 6

-2 -16 -10 727.319 91.5736 6

-16 18 -10 749.19 112.727 2

16 -18 10 580.563 130.587 10

-18 2 10 513.435 163.166 10

-2 -16 -10 786.128 100.679 8

18 -2 -10 871.321 144.805 7

-16 18 -10 849.415 103.518 11

-16 18 -10 543.459 118.864 3

16 -18 7 2720.84 360.072 10

-18 2 7 4239.78 510.082 10

2 16 7 3795.81 388.733 4

-2 -16 -7 4216.50 331.735 8

-16 18 -7 3704.66 331.085 2

-16 18 -7 3665.85 328.364 3

-16 18 -7 3049.49 424.356 4

-16 18 -7 3644.39 322.702 13

-2 -16 -7 3893.18 323.311 6

-2 -16 -7 4027.91 322.667 1

-16 18 -4 466.327 60.7773 2

-16 18 -4 431.678 59.9218 3

-18 2 4 195.68 143.544 10

-16 18 -4 254.592 326.384 4

-2 -16 -4 430.97 61.3614 6

-2 -16 -4 263.397 52.6632 8

-16 18 -1 8642.93 729.167 3

2 16 1 9342.38 816.408 9

2 16 110102.40 811.944 7

-16 18 2 554.537 251.149 4

-2 -16 2 1139.49 171.683 10

2 16 -2 1269.62 248.487 7

-2 -16 5 2877.55 319.024 10

16 -18 -5 2163.31 225.816 8

2 16 -5 2219.22 368.247 9

2 16 -5 3047.97 232.49 2

-16 18 5 2762.74 352.876 4

-18 2 -5 3251.15 232.974 1

16 -18 -5 2362.68 229.937 6

2 16 -5 2366.25 358.111 7

2 16 -5 2273.61 347.082 4

2 16 -8 6119.35 675.70 9

16 -18 -8 7093.37 589.373 8

16 -18 -8 6905.27 589.215 6

-2 -16 8 7071.97 697.871 10

-18 2 -8 8238.75 603.325 11

2 16 -8 6183.00 670.313 4

-18 2 -8 6665.32 590.025 1

2 16 -8 7403.50 590.287 2

2 16 -11 4667.12 350.792 14

-18 2 -11 3139.23 345.39 14

2 16 -11 4579.64 357.484 2

-2 -16 11 2832.62 416.069 10

-18 2 -11 4970.16 361.876 11

16 -18 -11 4230.40 354.936 8

2 16 -11 4576.13 484.857 7

16 -18 -14 33.2022 35.228 8

2 16 -14 32.1412 35.5607 2

2 16 -14 8.65088 30.9997 14

2 16 -17 -5.5821 20.2902 3

2 16 -17-6.38463 19.531 2

16 -18 -17-9.97127 17.1417 8

-17 18 -17 10.9417 12.8917 11

-1 -17 -14 226.888 45.654 8

18 -1 -14 186.366 35.0435 2

-17 18 -14 173.971 41.219 11

-17 18 -14 173.734 51.7114 2

-1 -17 -14 201.584 41.2917 6

-1 -17 -11 2582.70 220.897 6

-17 18 -11 2506.75 226.469 11

-18 1 11 1927.41 294.951 10

18 -1 -11 2635.20 277.295 9

-1 -17 -11 2604.59 224.662 8

-17 18 -11 2258.61 242.685 2

18 -1 -11 1910.65 263.262 7

-17 18 -11 2163.54 252.999 3

17 -18 8 327.842 101.539 10

-18 1 8 352.265 187.204 10

18 -1 -8 329.073 123.179 9

-1 -17 -8 579.911 80.8747 8

-17 18 -8 223.989 120.485 4

-17 18 -8 669.675 86.8806 3

-17 18 -8 357.657 73.5683 14

-1 -17 -8 671.697 74.8228 1

-17 18 -8 646.87 88.749 2

-1 -17 -8 680.648 76.6799 6

-17 18 -8 467.861 89.4702 11

-1 -17 -536694.40 2903.77 8

-17 18 -535409.30 2903.78 2

-17 18 -535366.50 2904.87 3

-1 -17 -536470.60 2900.51 1

-18 1 545555.80 3089.16 10

1 17 536006.40 2992.22 4

-1 -17 -536475.70 2902.75 6

-17 18 -532115.40 3113.06 4

-17 18 -2 3020.13 247.091 2

-17 18 -2 2589.43 612.242 4

1 17 2 2356.68 273.832 7

-17 18 -2 2990.98 250.495 3

1 17 2 2565.27 320.069 9

1 17 -121968.50 1559.15 7

1 17 -121433.40 1537.48 9

-17 18 121492.70 1658.81 4

-18 1 -116230.40 1380.19 5

1 17 -4 2447.57 382.003 9

18 -1 4 2075.31 230.044 9

17 -18 -4 2218.90 197.268 8

1 17 -4 2565.51 372.278 7

1 17 -4 2451.71 201.961 2

17 -18 -4 2159.96 199.739 6

-17 18 4 2479.49 344.53 4

-1 -17 4 2248.79 272.315 10

1 17 -4 2446.96 363.32 4

1 17 -7 998.014 215.591 4

17 -18 -7 1389.17 128.052 6

-1 -17 7 1699.48 235.214 10

1 17 -7 1458.56 250.465 9

17 -18 -7 1193.70 125.906 8

-17 18 7 887.338 193.68 4

1 17 -7 1410.21 260.117 7

-18 1 -7 1150.04 125.287 1

1 17 -7 1292.00 127.18 2

17 -18 -10 33.6722 40.8479 6

17 -18 -10 10.5355 42.5989 8

-18 1 -10-25.1273 52.7832 11

1 17 -10 36.3435 45.5051 2

1 17 -10 36.5825 36.4471 11

-1 -17 10-18.8506 101.437 10

1 17 -10 35.2426 39.3384 14

1 17 -10 95.1917 130.401 7

17 -18 -13 321.351 55.5823 8

1 17 -13 375.333 59.1124 2

1 17 -13 325.676 51.1971 14

1 17 -16 22.3619 25.1945 3

1 17 -16 20.8959 23.7539 2

17 -18 -16 10.489 21.4854 8

0 -18 -15 433.329 55.0766 8

-18 18 -15 350.504 57.3244 2

-18 18 -15 492.085 57.6781 14

-18 18 -15 431.319 52.7778 11

0 18 12 327.546 97.8031 10

-18 0 12 485.648 130.748 10

-18 18 -12 677.749 88.2812 14

0 -18 -12 757.59 86.8848 8

-18 18 -12 725.327 89.4627 11

0 -18 -12 806.921 86.2444 6

-18 18 -12 458.264 117.434 3

-18 18 -12 615.916 109.118 2

18 0 -9 447.984 125.405 9

0 -18 -9 288.257 64.9936 8

-18 0 9 343.013 174.268 10

-18 18 -9 277.955 69.1888 3

-18 18 -9 255.092 69.5878 11

0 -18 -9 374.514 59.342 1

-18 18 -9 198.744 73.6336 2

0 -18 -9 315.66 63.3086 6

-18 18 -9 188.183 64.0455 14

-18 18 -6 468.166 60.4204 14

0 -18 -6 411.436 60.3771 8

-18 18 -6 315.773 55.0203 3

-18 0 6 539.828 215.682 10

-18 18 -6 28.3344 184.981 4

-18 18 -6 213.582 78.9762 11

0 -18 -6 229.254 47.9369 1

-18 18 -6 356.498 56.9308 2

0 18 6 198.38 114.596 4

0 18 3 2207.54 303.124 4

-18 18 -3 1972.54 165.527 3

-18 0 3 2629.71 304.752 10

-18 18 -3 1104.26 447.024 4

-18 18 -3 1571.93 159.026 2

-18 18 0 101.231 41.1472 3

-18 18 0 209.957 336.883 4

0 18 0 180.101 132.105 9

-18 0 0 126.27 40.5124 5

0 -18 0 63.1032 39.9773 6

0 18 0 123.77 107.758 7

0 -18 3 169.573 100.134 10

0 18 -3 59.6244 180.481 9

18 0 3 111.829 79.5065 9

0 18 -3 178.599 42.534 3

0 18 -3 330.475 204.417 7

0 18 -3 208.655 39.8146 2

-18 18 3 74.4745 176.719 4

0 18 -3 106.224 220.375 4

-18 0 -3 134.791 37.8509 5

18 -18 -631078.80 2589.55 6

0 18 -641914.60 2828.84 7

0 -18 634700.20 2685.76 10

0 18 -628465.10 2748.88 9

18 -18 -631368.20 2588.90 8

-18 0 -632061.70 2590.61 1

-18 0 -633706.70 2599.41 11

0 18 -632177.60 2592.06 2

-18 18 635476.40 2711.53 4

0 18 -635712.10 2760.07 4

18 -18 -9 7113.07 652.359 8

18 -18 -9 7462.59 652.974 6

0 18 -9 8256.41 657.10 2

-18 0 -9 9173.59 666.877 11

0 -18 9 6367.43 738.722 10

0 18 -9 8304.59 655.137 14

-18 0 -9 8076.39 659.167 1

18 -18 -12-3.10734 40.5708 8

0 18 -12 5.00951 35.7495 14

0 18 -12 37.4355 45.2645 2

0 18 -12-86.1582 113.722 7

0 -18 12-15.3913 81.2616 10

0 18 -15 65.9114 29.2731 2

18 -18 -15 69.04 28.8997 8

-1 19 -17 9.19651 17.8376 2

-1 19 -17 29.9573 18.7443 3

-1 19 -14 213.613 44.9408 2

-1 19 -14 205.49 38.3608 14

19 -18 -14 207.167 39.3133 8

-1 19 -11 77.5335 37.7956 11

19 -18 -11 22.6893 41.2008 8

-1 19 -11-23.8761 118.596 7

1 -19 11-34.1642 93.6349 10

-1 19 -11 40.1671 46.5465 2

-1 19 -11 32.5775 37.9227 14

19 -18 -8 2175.15 201.003 6

-18 -1 -8 2848.12 210.124 1

-1 19 -8 1671.58 330.993 7

1 -19 8 2053.71 288.38 10

-1 19 -8 1283.31 279.113 9

19 -18 -8 1857.02 200.123 8

-1 19 -8 2730.25 209.014 2

-18 -1 -8 2283.39 215.172 11

-1 19 -8 2032.90 204.53 14

1 -19 5 1224.24 201.031 10

-1 19 -5 1061.38 302.95 9

19 -18 -5 1074.05 128.522 8

-19 18 5 968.90 228.05 4

-1 19 -5 1677.93 143.471 3

-1 19 -5 1619.58 140.525 2

19 -18 -5 1226.21 131.468 6

-1 19 -5 1333.37 308.728 7

-18 -1 -5 2078.08 142.903 1

-1 19 -5 1513.08 323.432 4

-1 19 -2 1652.40 157.999 2

-18 -1 -2 1951.73 160.634 5

18 1 2 1804.50 220.787 9

1 -19 2 1669.60 224.886 10

-1 19 -2 1060.07 380.894 4

-19 18 2 1829.28 333.988 4

-1 19 -2 1896.98 334.943 7

-1 19 -2 1714.81 159.692 3

-1 19 1-16.3842 118.755 9

18 1 -1 58.5068 111.294 9

1 -19 -1-8.02851 37.7974 6

-1 19 1 9.40553 63.8649 7

-19 18 -1 24.4069 35.0779 3

-1 19 1-66.4361 228.152 4

1 -19 -4 3725.95 287.639 8

-18 -1 4 4290.69 443.724 10

-19 18 -4 2939.80 283.129 2

-19 18 -4 3555.99 559.063 4

-19 18 -4 3393.66 288.543 3

-1 19 4 3424.72 407.503 4

-19 18 -7 4902.38 395.081 11

-19 18 -7 4520.65 367.062 13

-19 18 -7 4659.57 371.658 14

18 1 -7 5345.76 462.63 9

-18 -1 7 5329.06 575.812 10

-1 19 7 3894.01 437.017 4

1 -19 -7 4428.21 370.011 8

1 -19 -7 4148.43 361.707 1

-19 18 -7 3447.56 472.497 4

-19 18 -7 3783.41 365.816 3

-19 18 -7 4054.23 368.964 2

1 -19 -10 1833.38 167.206 6

-18 -1 10 1844.49 280.567 10

18 1 -10 1750.75 218.438 9

1 -19 -10 1638.47 167.23 8

18 1 -10 1528.17 223.715 7

-19 18 -10 1943.71 178.003 11

-19 18 -10 1409.36 181.431 3

-19 18 -10 1682.96 169.417 14

-19 18 -10 1570.07 186.733 2

-19 18 -13 444.634 86.0683 2

1 -19 -13 555.133 69.7888 8

1 -19 -13 393.908 64.9633 6

-19 18 -13 478.464 68.554 11

-19 18 -13 582.641 72.0652 14

-19 18 -16 98.7679 23.68 11

-19 18 -16 104.342 31.3295 14

1 -19 -16 144.596 27.7694 8

19 -17 -15 178.928 33.7745 8

-2 19 -15 239.721 36.6503 14

-2 19 -15 238.525 39.5747 2

-2 19 -15 150.454 37.1248 3

19 -17 -12 1440.80 141.77 8

-2 19 -12 1664.87 149.614 2

2 -19 12 1269.38 204.145 10

-2 19 -12 1579.14 140.093 14

-2 19 -12 1396.81 138.238 11

-2 19 -12 1016.09 222.022 7

2 -19 9 456.685 139.163 10

-2 19 -9 479.11 75.7859 14

19 -17 -9 652.606 75.8336 8

-2 19 -9 592.097 80.2414 2

-17 -2 -9 522.401 79.6793 1

-17 -2 -9 639.719 89.1586 11

-2 19 -9 427.877 71.4664 11

19 -17 -6 2380.61 233.165 6

2 -19 6 3212.61 329.541 10

-2 19 -6 2414.11 416.104 9

19 -17 -6 2783.19 236.247 8

-2 19 -6 3683.73 493.219 7

-2 19 -6 2679.93 243.657 3

-17 -2 -6 2618.93 236.277 1

-19 17 6 2738.89 340.483 4

-2 19 -6 2250.47 397.127 4

-2 19 -6 3233.20 244.36 13

-2 19 -6 2541.60 238.922 2

-2 19 -318365.30 1365.63 7

-2 19 -313283.70 1094.93 2

-19 17 314165.40 1252.44 4

2 -19 314677.30 1167.36 10

-2 19 -315591.00 1322.41 9

17 2 312456.40 1136.93 9

-2 19 -310541.30 1339.80 4

-2 19 -313546.80 1096.61 3

2 -19 0 7675.83 618.591 6

-19 17 0 8721.75 910.745 4

-2 19 0 7876.65 732.721 9

17 2 0 6219.02 677.516 9

-2 19 0 9439.57 931.758 4

-2 19 0 7307.55 685.69 7

17 2 -3 4291.30 394.072 9

-17 -2 3 4132.28 420.259 10

-19 17 -3 3104.11 280.849 2

-19 17 -3 3290.64 285.016 3

2 -19 -3 3432.18 285.747 6

-2 19 3 4058.21 435.023 4

17 2 -612053.70 973.084 9

-2 19 6 9951.93 960.203 4

2 -19 -611787.20 877.925 8

-19 17 -610430.10 875.344 2

-19 17 -6 9396.01 1017.28 4

2 -19 -610154.40 871.336 1

-19 17 -611836.80 906.075 11

-19 17 -610998.10 878.394 14

-19 17 -6 9915.79 874.604 3

-17 -2 9 3225.38 448.176 10

17 2 -9 4220.36 389.049 9

-19 17 -9 3613.08 321.809 3

-19 17 -9 2906.68 313.699 14

-19 17 -9 3608.43 325.577 11

-19 17 -9 3960.91 331.325 2

2 -19 -9 3912.28 318.613 8

2 -19 -12 6.52184 45.3031 6

17 2 -12-12.5907 67.7435 7

2 -19 -12 18.0288 43.5434 8

-19 17 -12 31.0134 49.8276 14

17 2 -12 2.37324 31.3779 2

-19 17 -12-3.76584 45.3088 11

-19 17 -12-6.93721 71.832 2

-17 -2 12 31.9222 79.2608 10

2 -19 -15 543.058 61.6215 8

-19 17 -15 460.075 59.2874 11

-19 17 -15 620.842 64.1156 14

19 -16 -16 102.255 24.8429 8

-3 19 -16 161.204 30.8905 3

-3 19 -16 135.41 30.1762 2

19 -16 -13 10.6489 33.6051 8

-3 19 -13 25.8505 43.8059 2

-3 19 -13 38.045 29.4827 11

-3 19 -13 30.553 33.7018 14

3 -19 10 1259.94 214.295 10

-3 19 -10 1057.28 252.032 7

19 -16 -10 1180.61 128.074 8

-3 19 -10 1511.22 139.987 2

-3 19 -10 1363.94 133.771 14

-3 19 -10 1285.51 131.457 11

19 -16 -7 1437.57 146.169 6

3 -19 7 1274.61 213.492 10

-16 -3 -7 1930.95 167.305 11

19 -16 -7 1404.52 148.11 8

-3 19 -7 1450.18 153.119 2

-16 -3 -7 1542.04 150.059 1

-19 16 7 1697.55 242.662 4

-3 19 -7 2314.44 407.138 7

-3 19 -7 1890.75 157.568 14

-3 19 -7 1616.58 288.612 4

-3 19 -412602.00 940.296 3

-19 16 410987.90 1054.90 4

3 -19 411697.40 998.884 10

-3 19 -412349.70 939.196 2

-3 19 -413151.50 1208.34 9

16 3 410263.40 966.474 9

19 -16 -410486.00 929.862 8

-3 19 -412141.40 1258.84 4

-3 19 -113962.10 1115.51 7

16 3 112911.10 1073.24 9

-3 19 -112071.00 1004.98 3

-19 16 113273.90 1216.70 4

3 -19 -2 468.258 68.4311 6

-3 19 2 470.217 225.349 4

16 3 -2 686.733 151.064 9

-16 -3 2 577.943 70.6371 5

-19 16 -2 532.811 66.5137 3

-19 16 -2 180.918 295.465 4

16 3 -5 9069.29 733.295 9

-16 -3 5 9504.57 798.931 10

3 -19 -5 8029.07 631.841 8

-19 16 -5 7030.14 629.532 3

3 -19 -5 7547.49 627.60 1

-19 16 -5 7343.26 628.839 2

-19 16 -5 7005.98 800.874 4

3 -19 -5 8359.82 633.464 6

-3 19 5 7810.59 739.688 4

19 -16 8 1472.99 212.948 10

-16 -3 8 2203.26 339.699 10

16 3 -8 1971.04 243.473 9

3 -19 -8 1836.60 172.604 8

-19 16 -8 1403.22 233.837 4

-19 16 -8 1538.41 167.634 3

-19 16 -8 1845.14 175.33 14

3 -19 -8 2057.86 170.953 1

-19 16 -8 1857.35 176.948 2

16 3 -11 2805.56 208.957 2

-19 16 -11 2167.21 239.536 2

3 -19 -11 2541.05 218.436 8

16 3 -11 1898.82 267.121 7

-19 16 -11 2491.62 221.445 11

3 -19 -11 2559.04 216.00 6

-3 19 11 1627.10 241.741 10

19 -16 11 1586.17 242.144 10

-16 -3 11 1852.40 291.506 10

-19 16 -11 2234.98 217.748 14

-19 16 -14 517.257 63.9931 14

-19 16 -14 395.458 57.8267 11

16 3 -14 444.323 53.4082 2

3 -19 -14 524.826 61.4109 6

-19 16 -14 379.522 73.2991 2

3 -19 -14 398.194 59.6744 8

3 -19 -17 51.8927 19.9695 8

-19 16 -17 110.71 18.0811 11

-4 19 -17-13.6971 20.505 3

-4 19 -17 9.70761 19.7426 2

19 -15 -14 498.84 60.3673 8

-4 19 -14 434.308 64.0921 3

-4 19 -14 429.287 53.213 11

-4 19 -14 464.362 65.2228 2

19 -15 -11 1233.57 126.144 8

-4 19 -11 1263.85 132.664 2

4 -19 11 1120.77 198.224 10

-4 19 -11 1369.76 267.073 7

-4 19 -11 1113.20 124.804 11

-4 19 -11 1515.69 129.532 14

4 -19 8 1358.11 211.65 10

-4 19 -8 1300.36 127.441 14

-4 19 -8 1182.68 123.952 2

-4 19 -8 1158.09 319.161 7

-15 -4 -8 1011.25 117.509 1

-4 19 -8 1442.11 235.98 4

19 -15 -8 1074.94 117.49 8

-4 19 -5 4661.89 377.206 3

15 4 5 4961.19 403.724 9

-4 19 -5 4803.03 630.465 9

19 -15 -5 4472.05 367.827 8

-19 15 5 4475.18 480.093 4

19 -15 -5 3883.42 366.664 6

-4 19 -5 5630.45 684.329 4

4 -19 5 5396.44 450.115 10

-4 19 -5 4145.32 372.667 2

-4 19 -224328.90 1977.01 2

-19 15 225762.70 2141.07 4

4 -19 225432.80 2030.85 10

-4 19 -226373.50 2121.07 7

-4 19 -224868.50 1977.93 3

-4 19 -224201.00 2128.14 9

15 4 222309.90 2025.69 9

-4 19 -229760.20 2482.46 4

-4 19 113850.50 1294.44 7

-15 -4 116684.10 1264.78 5

-4 19 118441.30 1511.55 4

4 -19 -115449.30 1262.88 6

15 4 -114116.80 1331.97 9

4 -19 -4 49.3929 38.9345 6

-19 15 -4 59.7674 35.2569 3

-19 15 -4-205.738 235.637 4

4 -19 -4 210.275 36.1453 1

-19 15 -4 109.154 32.5733 2

-15 -4 4 258.297 163.475 10

19 -15 4-8.96223 57.7225 10

-4 19 4 19.3125 128.274 4

4 -19 -7 120.567 43.0535 6

-19 15 -7 40.1584 61.6502 11

-4 19 7 176.449 116.524 4

19 -15 7 46.4088 66.2574 10

-15 -4 7 79.431 155.278 10

15 4 -7 27.6558 85.0619 9

4 -19 -7 104.688 46.6027 8

-19 15 -7 100.837 47.3659 14

-19 15 -7 85.2639 39.9244 2

4 -19 -7 144.707 42.979 1

-19 15 -7-41.0956 110.826 4

-19 15 -7 117.781 36.2231 3

19 -15 10 356.874 100.173 10

-15 -4 10 435.717 155.349 10

-4 19 10 162.331 85.2187 10

15 4 -10 593.342 123.126 9

4 -19 -10 499.731 75.1496 8

-19 15 -10 372.347 74.8434 11

-19 15 -10 559.462 95.2809 2

15 4 -10 410.557 121.938 7

-19 15 -10 244.938 71.6609 14

15 4 -10 725.945 67.7748 2

4 -19 -13 330.305 58.8529 6

-19 15 -13 318.799 59.8076 11

-19 15 -13 366.831 82.4346 2

4 -19 -13 289.836 59.433 8

15 4 -13 359.32 51.0587 2

-19 15 -13 459.711 61.4957 14

-19 15 -16 112.251 25.3269 11

-19 15 -16 44.7658 30.2689 14

4 -19 -16 129.35 29.1448 8

15 4 -16 131.319 25.0156 2

-5 19 -15 317.087 50.389 3

-5 19 -15 468.067 53.8464 2

-5 19 -15 359.587 48.6711 14

19 -14 -15 330.323 46.0893 8

-5 19 -12 2791.88 235.888 2

19 -14 -12 2650.00 229.017 8

-5 19 -12 2376.37 227.342 11

-5 19 -12 2481.84 227.111 14

-5 19 -12 2425.08 235.169 3

5 -19 12 2214.01 297.725 10

-5 19 -9 715.754 89.3561 2

19 -14 -9 565.822 75.843 8

-5 19 -9 457.423 79.9873 14

-5 19 -9 554.975 80.4639 11

5 -19 9 394.042 141.141 10

19 -14 -6 419.545 64.2108 8

-5 19 -6 937.497 269.433 4

-5 19 -6 833.909 357.282 7

-5 19 -6 556.65 80.4855 3

-19 14 6 556.903 155.977 4

-5 19 -6 591.878 73.4433 2

5 -19 6 648.133 134.64 10

-14 -5 -6 526.581 68.8463 1

-5 19 -3 2987.92 275.273 2

-19 14 3 3621.80 416.834 4

-5 19 -3 3576.92 807.036 4

-5 19 -3 3100.24 275.648 3

14 5 3 3791.56 331.032 9

5 -19 3 3469.48 330.117 10

-5 19 -3 4621.44 497.739 7

-19 14 0 2969.35 485.03 4

5 -19 0 3343.54 289.64 6

-14 -5 0 3504.02 291.213 5

14 5 0 3084.48 355.844 9

-5 19 0 3647.16 346.74 7

-5 19 3 260.749 182.83 4

14 5 -3-14.4959 109.351 9

-19 14 -3 36.3513 241.045 4

5 -19 -3 52.0041 39.0272 6

-19 14 -6 41.8063 32.2337 2

5 -19 -6 22.7992 33.0757 1

5 -19 -6 101.737 42.5865 6

-14 -5 6 70.8693 142.65 10

19 -14 6 34.5561 69.5945 10

14 5 -6 42.035 88.2886 9

5 -19 -6 68.2476 38.5933 8

-5 19 6-34.9312 103.92 4

-19 14 -6-70.4812 137.635 4

-19 14 -6 2.5694 30.0972 3

-19 14 -9 123.426 60.9959 11

19 -14 9 61.62 78.0186 10

-14 -5 9 216.489 143.294 10

14 5 -9 89.2931 82.8593 9

5 -19 -9 139.135 53.9642 8

-19 14 -9 43.9052 55.6777 14

14 5 -9 16.6492 88.2546 7

14 5 -9 94.7775 38.8789 2

5 -19 -9 114.992 53.595 6

-19 14 -9 117.963 52.247 2

14 5 -12 -57.035 79.5068 7

5 -19 -12 10.784 46.1898 8

-19 14 -12-43.9033 76.8928 2

19 -14 12 -11.373 68.6654 10

-14 -5 12 48.5289 88.6304 10

14 5 -12-21.1554 34.4185 2

-19 14 -12-3.77682 44.2444 11

-19 14 -12 86.9153 50.8716 14

14 5 -15 123.201 29.2344 2

-19 14 -15 68.7557 31.9398 11

-19 14 -15 166.614 40.2476 14

5 -19 -15 101.199 34.0876 8

-6 19 -16 60.6613 29.0472 2

-6 19 -16-30.1286 28.9913 14

-6 19 -16 37.6101 24.467 3

19 -13 -16 16.1175 19.6719 8

19 -13 -13 23.5775 34.1047 8

-6 19 -13 85.4277 38.9476 14

-6 19 -13-3.43854 47.5287 2

-6 19 -13 71.0349 43.8005 3

-6 19 -13 67.9563 34.3025 11

-6 19 -10 8987.39 776.776 14

-6 19 -10 9340.25 780.964 2

19 -13 -10 8968.96 771.328 8

-6 19 -10 8782.17 776.661 11

-6 19 -10 9600.43 1005.40 7

-13 -6 -1010259.90 781.516 1

6 -19 10 7824.53 849.372 10

-13 -6 -7 989.805 100.451 6

6 -19 7 814.138 169.151 10

-13 -6 -7 1051.24 104.32 1

19 -13 -7 781.228 97.9406 8

-6 19 -7 1101.14 111.922 2

-19 13 7 961.504 187.597 4

-6 19 -7 868.24 114.16 3

-6 19 -7 1054.74 107.418 14

-6 19 -7 970.176 107.04 13

-6 19 -7 1154.35 255.027 4

-6 19 -7 1275.39 424.10 7

-6 19 -415826.60 1239.32 2

-19 13 416463.30 1359.15 4

6 -19 413750.90 1278.43 10

13 6 413598.90 1268.75 9

-6 19 -419380.50 1882.14 4

-6 19 -415484.60 1239.32 3

-6 19 -418434.80 1511.84 7

-19 13 139757.10 3275.21 4

13 6 133466.10 3143.46 9

-6 19 -140646.70 3090.02 2

-6 19 -139098.30 3089.88 3

-6 19 -135436.50 3156.46 7

-13 -6 -137818.40 3088.50 5

6 -19 -215856.70 1297.55 6

-19 13 -216369.70 1540.06 4

13 6 -215399.00 1376.39 9

-6 19 218116.00 1477.64 4

-13 -6 216282.20 1299.61 5

6 -19 -589938.80 7205.07 6

-6 19 595248.30 7324.63 4

6 -19 -589530.60 7204.60 8

13 6 -594869.00 7293.31 9

-19 13 -586213.40 7361.97 4

13 6 -8 338.512 112.832 9

19 -13 8 261.826 99.4365 10

-13 -6 8 397.646 172.313 10

-19 13 -8 274.509 55.6496 2

6 -19 -8 366.229 62.9068 6

-19 13 -8 207.346 64.0797 14

6 -19 -8 329.423 62.4433 8

-19 13 -11 2207.28 233.165 14

19 -13 11 2172.20 277.33 10

-13 -6 11 2194.00 314.49 10

-19 13 -11 2150.57 252.836 2

13 6 -11 2367.93 304.892 7

-19 13 -11 2723.78 239.263 11

13 6 -14 789.724 78.8225 2

-19 13 -14 664.058 81.7215 11

-19 13 -14 752.614 83.4332 14

6 -19 -17 123.64 25.4055 8

-7 19 -17 38.249 24.0093 2

-7 19 -14 54.706 28.1346 11

-7 19 -14 92.8813 39.5569 14

-7 19 -14 58.0454 38.3497 3

19 -12 -14 72.0911 33.3105 8

-7 19 -14 9.22717 40.5474 2

7 -19 11-63.2052 91.2232 10

-12 -7 -11 56.9126 38.0404 6

-7 19 -11 116.169 51.5986 2

-7 19 -11-14.0589 46.5721 14

-7 19 -11 94.4647 62.3824 3

-7 19 -11 75.6087 43.2644 11

19 -12 -11 98.5419 38.9662 8

-12 -7 -11 72.161 60.8806 8

-19 12 8-22.8438 115.918 10

7 -19 8 31.2175 91.4775 10

-7 19 -8 3.71609 47.8498 14

19 -12 -8 95.0251 36.3924 8

-7 19 -8 24.6458 47.4626 2

-12 -7 -8 6.15396 39.0133 6

-7 19 -8 77.303 48.8105 11

-7 19 -8-21.4855 94.4362 4

-7 19 -8-122.979 354.501 7

12 7 5 444.254 80.2339 9

-7 19 -5 299.505 59.5414 3

-19 12 5 489.846 149.777 4

-7 19 -5 269.043 56.3682 2

-12 -7 -5 379.411 49.519 6

-7 19 -5 519.978 269.53 7

-19 12 5 380.056 89.1394 10

7 -19 5 355.772 96.6743 10

-7 19 -2 816.836 89.5193 3

-19 12 2 822.831 228.085 4

-7 19 -2 773.508 171.176 7

7 -19 2 1028.79 128.153 10

-7 19 -2 1176.29 838.705 4

-7 19 -2 864.713 91.446 2

12 7 2 794.812 141.262 9

-12 -7 1 277.308 49.6953 5

-7 19 1 522.617 247.484 4

12 7 -1 591.504 129.999 9

7 -19 -418208.70 1485.11 6

-19 12 -419561.70 1681.43 4

-7 19 419718.10 1622.42 4

-7 19 7 7207.12 692.362 4

7 -19 -7 7251.40 593.145 6

-19 12 -7 7694.50 609.84 11

-19 12 -7 6651.97 587.923 2

12 7 -7 7440.63 666.583 9

-19 12 -7 7369.37 599.424 14

12 7 -10 2768.25 305.149 9

-12 -7 10 2068.12 339.704 10

12 7 -10 2413.56 330.822 7

-19 12 -10 3076.75 258.763 11

7 -19 -10 2942.07 253.038 6

19 -12 10 2300.10 296.51 10

-19 12 -10 2802.46 255.612 14

12 7 -10 3020.99 246.914 2

-19 12 -10 2486.14 261.317 2

-19 12 -13 407.954 59.0478 11

-19 12 -13 93.5094 72.4448 2

12 7 -13 259.888 49.2476 2

-19 12 -13 499.16 64.0676 14

-12 -7 13 209.203 93.4984 10

12 7 -16 161.743 29.002 2

-19 12 -16 223.553 30.4291 11

19 -11 -15 134.82 31.1704 8

-8 19 -15 228.637 42.1439 14

-8 19 -15 148.639 39.3792 3

-8 19 -15 156.508 28.9687 11

-11 -8 -12 1202.57 110.09 6

-8 19 -12 966.861 108.352 11

8 -19 12 698.403 156.823 10

-8 19 -12 961.854 117.326 2

-11 -8 -12 1445.24 132.121 8

-8 19 -12 985.057 120.729 3

-8 19 -12 1042.08 111.593 14

19 -11 -12 1001.55 105.348 8

-11 -8 -9 32.9421 41.0166 6

-8 19 -9 216.247 60.9562 14

-11 -8 -9 25.2206 70.3922 8

19 -11 -9 110.405 38.9299 8

-8 19 -9 85.8103 51.9437 11

-8 19 -9 74.7918 54.2033 2

-8 19 -9 97.9863 73.7673 3

-19 11 9 248.424 132.339 10

8 -19 9 129.24 106.046 10

-8 19 -6 456.439 71.699 2

-11 -8 -6 504.224 60.7743 6

-8 19 -6 836.79 368.25 7

-19 11 6 723.641 157.199 10

8 -19 6 518.036 114.649 10

-8 19 -6 124.745 223.046 4

-19 11 6 477.537 155.94 4

-8 19 -3 9992.61 825.021 3

-8 19 -3 9551.37 824.901 2

8 -19 310454.30 860.50 10

11 8 310903.60 877.846 9

-19 11 311093.30 964.229 4

-8 19 -312001.70 973.27 7

-19 11 012294.90 1238.85 4

-11 -8 013060.20 1046.45 5

-8 19 012965.80 1047.33 3

11 8 011609.50 1110.98 9

-19 11 -338132.30 3405.36 4

8 -19 -337976.20 3212.43 6

-11 -8 345258.20 3330.45 10

-8 19 343304.90 3374.53 4

-11 -8 341826.80 3214.90 5

8 -19 -6 3894.48 327.892 6

11 8 -6 3822.90 401.007 9

-8 19 6 3738.08 436.668 4

-19 11 -6 3594.55 455.347 4

8 -19 -9-7.19846 47.662 6

-19 11 -9 32.9606 52.8101 11

11 8 -9 74.2154 38.8595 2

-19 11 -9 33.8674 42.137 2

-8 19 9 94.6841 59.7003 10

19 -11 9-40.4492 81.4524 10

11 8 -9 63.4785 77.8199 9

-19 11 -9-13.3717 53.0974 14

11 8 -9 71.659 103.317 7

19 -11 12 349.435 102.387 10

-19 11 -12 614.809 76.1562 14

11 8 -12 568.943 69.3232 2

8 -19 -12 525.233 78.7443 6

-19 11 -12 250.369 87.3002 2

-19 11 -12 590.885 78.3818 11

11 8 -12 433.023 126.385 7

-19 11 -15 131.687 34.5491 11

11 8 -15 155.273 31.8203 2

-9 19 -16 91.6296 33.6702 14

19 -10 -13 115.414 35.707 8

-10 -9 -13 105.292 44.464 8

-9 19 -13 115.114 47.2077 14

-9 19 -13 94.135 49.871 3

9 -19 13 37.4713 63.776 10

10 9 13 -45.094 89.704 10

-9 19 -13 98.6537 36.4037 11

-19 10 10-17.5255 101.065 10

9 -19 10-31.5776 93.9451 10

-9 19 -10 32.6604 54.3548 14

19 -10 -10 29.5948 35.1023 8

-10 -9 -10 42.2558 61.4396 8

-10 -9 -10 57.3803 44.9601 6

-10 -9 -10 34.8391 53.5318 1

-9 19 -10 45.9439 45.4244 11

-9 19 -10 6.4544 71.0848 3

-9 19 -7 71.3188 361.771 7

-9 19 -7 44.1701 53.0836 3

-9 19 -7 44.5404 42.3112 14

-9 19 -7 24.3427 47.0003 2

-10 -9 -7 27.342 37.6578 1

-10 -9 -7 23.6413 64.2086 8

-9 19 -7-143.238 201.189 4

-19 10 7 25.6254 147.742 10

9 -19 7 39.9977 83.9908 10

-10 -9 -7 2.69916 34.5017 6

-10 -9 -4 2038.88 173.913 6

-9 19 -4 2082.40 333.696 7

10 9 4 2152.81 225.012 9

-9 19 -4 2059.36 181.153 3

9 -19 4 1972.31 214.943 10

-19 10 4 1172.49 207.854 10

-9 19 -4 2468.13 867.738 4

-19 10 4 2176.55 299.977 4

-9 19 -4 1978.58 180.801 2

-10 -9 -1 404.25 54.2925 5

-9 19 -1 275.269 50.622 2

10 9 1 622.61 125.91 9

-9 19 -1 385.849 55.1603 3

-19 10 1 539.238 228.63 4

-10 -9 2-31.9303 146.039 10

-9 19 2 29.2319 36.6755 3

-19 10 -2-161.904 219.887 4

-9 19 2-54.4169 205.129 4

10 9 -2 29.7417 111.091 9

-10 -9 2 79.9076 35.5255 5

-10 -9 5 4830.88 460.981 10

-19 10 -5 4213.24 485.514 4

9 -19 -5 3658.78 323.543 6

-9 19 5 4559.46 471.784 4

9 -19 -5 3667.28 322.953 8

10 9 -5 4607.40 422.892 9

-9 19 8 2400.59 261.224 10

-10 -9 8 2788.89 363.194 10

10 9 -8 2466.86 291.216 9

10 9 -8 2862.36 332.355 7

-19 10 -8 2717.60 239.865 14

9 -19 -8 2580.01 229.815 6

10 9 -8 2485.88 224.261 2

9 -19 -8 2474.43 229.008 8

9 -19 -11 95.6428 50.6877 6

9 -19 -11 33.0835 48.5274 8

10 9 -11 36.0299 102.561 7

-19 10 -11 170.821 55.9959 14

19 -10 11 81.4466 85.9647 10

10 9 -11 178.677 45.9932 2

-19 10 -11 72.3331 66.6197 2

-19 10 -11 144.654 55.3668 11

-19 10 -14 76.8766 42.1379 14

-19 10 -14-2.66797 35.0805 11

10 9 -14 2.35878 29.9456 2

9 -19 -14 19.4209 35.2115 8

9 -19 -17 22.065 20.7242 8

10 9 -17 10.2138 17.4703 2

-10 19 -17-6.27277 21.8454 2

19 -9 -17 24.4042 16.9491 2

-10 19 -17 -39.229 26.5321 14

-10 19 -14 402.861 65.252 3

-10 19 -14 571.765 66.1765 14

-9 -10 -14 408.58 62.8476 8

19 -9 -14 430.768 54.1759 8

-10 19 -14 379.04 53.1399 11

-19 9 11 135.454 87.4608 10

10 -19 11 176.733 105.123 10

-9 -10 -11 95.8532 46.4658 6

-9 -10 -11 50.6417 56.8696 8

19 -9 -11 143.383 40.184 8

-10 19 -11 272.009 65.0876 14

-10 19 -11 190.261 53.5624 11

-10 19 -11 95.4123 70.5647 3

10 -19 8 3205.88 367.998 10

-9 -10 -8 3366.49 296.925 6

-19 9 8 2876.10 427.036 10

-10 19 -8 3031.50 315.227 3

-9 -10 -8 4440.55 322.171 8

-10 19 -8 2746.75 382.029 4

-10 19 -8 3720.86 311.616 11

-9 -10 -8 3231.16 297.062 1

-10 19 -8 3611.22 304.978 14

9 10 5 1507.69 194.513 9

-19 9 5 1456.78 234.228 10

10 -19 5 1752.18 204.624 10

-9 -10 -5 1854.77 158.875 6

-10 19 -5 1837.49 372.393 7

-10 19 -2 475.648 62.2222 2

9 10 2 308.256 111.309 9

10 -19 2 408.612 73.7565 10

-10 19 -2 520.707 64.2096 3

-19 9 2 408.454 192.994 4

-10 19 1-35.5878 258.17 4

-10 19 1 142.93 40.2118 3

9 10 -1 118.605 105.801 9

-9 -10 4 7015.99 590.406 10

-19 9 -4 5397.67 635.311 4

10 -19 -4 5145.85 463.106 6

9 10 -4 5684.17 562.225 9

-10 19 4 5269.36 605.924 4

-9 -10 4 6015.20 465.482 5

9 10 -7 182.498 107.359 9

10 -19 -7 329.89 56.0349 8

9 10 -7 330.222 49.2129 2

-19 9 -7 234.587 63.0817 11

-10 19 7 107.738 109.301 4

10 -19 -7 307.761 57.2123 6

-9 -10 7 324.268 166.118 10

9 10 -10 201.498 89.0521 9

10 -19 -10 225.091 60.0583 8

-19 9 -10 214.297 60.3462 11

-19 9 -10 193.578 60.3096 14

10 -19 -10 241.562 58.5513 6

-19 9 -10 124.711 52.2793 2

9 10 -10 294.799 50.1935 2

-9 -10 10 202.217 141.445 10

19 -9 10 115.916 85.1836 10

9 10 -10 228.757 122.816 7

9 10 -13 30.8084 78.4435 7

10 -19 -13 131.126 47.1068 8

10 -19 -13 63.2433 43.5346 6

9 10 -13 51.7018 34.8533 2

-19 9 -13 181.783 50.4639 14

-19 9 -13 71.4765 44.019 11

19 -9 13 97.3982 74.8986 10

9 10 -16-3.22091 22.506 2

10 -19 -16 26.7802 26.4118 8

-11 19 -15 1526.31 148.101 11

-11 19 -15 1750.23 158.006 2

-11 19 -15 2057.99 156.932 14

-11 19 -15 1664.85 157.897 3

-11 19 -12 2825.49 304.967 3

-11 19 -12 2976.32 299.808 2

-8 -11 -12 4023.81 304.247 8

19 -8 -12 3637.50 287.771 8

-11 19 -12 3220.88 293.16 11

-8 -11 -12 3564.95 293.172 6

11 -19 12 2252.16 331.989 10

-11 19 -12 3608.24 297.109 14

-8 -11 -9 5977.03 543.316 1

-11 19 -9 7012.96 552.785 14

-8 -11 -9 7725.40 559.131 8

-11 19 -9 6529.56 570.072 3

-11 19 -9 6920.49 554.649 11

11 -19 9 5805.01 610.002 10

-19 8 9 5101.47 647.041 10

-8 -11 -9 6132.48 542.293 6

-8 -11 -6 332.531 67.9706 8

-8 -11 -6 168.587 47.2403 1

-11 19 -6 290.68 58.5613 3

-11 19 -6 421.884 221.902 4

-8 -11 -6 226.241 44.0796 6

-19 8 6 191.615 149.983 10

11 -19 6 224.796 87.1826 10

-8 -11 -324015.00 1914.79 6

11 -19 322116.00 1933.71 10

8 11 323699.20 1972.75 9

-11 19 021661.20 1751.44 3

-19 8 021206.20 1947.18 4

8 11 020957.40 1829.54 9

-19 8 -332616.90 2341.94 4

-8 -11 334488.90 2221.29 10

-11 19 329842.30 2284.78 4

-8 -11 6 2317.38 292.154 10

8 11 -6 1929.37 249.475 9

11 -19 -6 1975.49 168.888 8

8 11 -6 1846.45 164.17 2

-11 19 6 1918.08 289.913 4

-19 8 -6 1536.60 163.074 1

11 -19 -6 1849.09 169.263 6

8 11 -6 2418.04 278.423 7

-8 -11 6 1857.26 164.876 5

-19 8 -9 2832.57 261.578 11

8 11 -9 3079.08 324.074 9

8 11 -9 3469.79 254.672 2

11 -19 -9 2732.95 254.699 8

8 11 -9 3043.21 360.832 7

-8 -11 9 2593.12 360.877 10

11 -19 -9 3019.61 257.497 6

-19 8 -9 2223.49 252.351 14

8 11 -12 5855.10 403.661 2

11 -19 -12 4546.95 402.937 8

8 11 -12 5202.10 505.416 7

-19 8 -12 5029.19 405.532 11

11 -19 -12 4946.74 402.675 6

-19 8 -12 4048.67 397.366 14

-8 -11 12 3318.75 460.07 10

19 -8 12 4074.00 458.673 10

-19 8 -15 199.111 37.5797 11

11 -19 -15 175.245 38.5856 8

8 11 -15 133.864 32.9356 2

-12 19 -16 8.07183 17.3895 11

-12 19 -16 57.0316 29.0429 2

-12 19 -16-5.12101 31.8396 14

7 12 13-27.0339 69.9908 10

-7 -12 -13-8.29696 37.7953 6

-12 19 -13 92.5146 49.9957 14

-7 -12 -13 21.2098 42.7036 8

19 -7 -13 8.87064 27.869 8

-12 19 -13 5.84175 34.6385 11

-12 19 -13 25.5185 48.1607 3

-12 19 -13 40.0247 45.416 2

-12 19 -10 7197.21 649.987 3

-12 19 -10 7544.79 630.093 11

12 -19 10 5472.70 672.757 10

-7 -12 -10 7676.97 627.173 1

-19 7 10 5976.02 708.256 10

-12 19 -10 7824.62 639.113 2

-7 -12 -10 8425.90 633.54 8

19 -7 -10 5913.90 655.68 7

-7 -12 -10 8037.90 624.005 6

-12 19 -10 7919.71 631.779 14

-12 19 -7 419.752 76.379 11

-19 7 7 443.103 201.225 10

12 -19 7 627.213 120.236 10

-12 19 -7 366.961 65.7605 13

-7 -12 -7 468.694 77.9994 8

-7 -12 -7 421.412 62.2886 1

-12 19 -7 538.259 74.3116 2

-12 19 -7 362.68 153.307 4

-12 19 -7 421.653 68.6014 14

-7 -12 -7 359.881 58.7675 6

-12 19 -7 570.757 81.7617 3

-12 19 -4 681.655 86.7948 3

12 -19 4 757.659 108.802 10

-19 7 4 758.308 163.219 10

-7 -12 -4 919.243 85.4546 6

7 12 4 968.339 137.695 9

-12 19 -4 658.694 86.7268 2

-7 -12 -112989.90 1079.78 6

-12 19 -113758.20 1082.97 3

-19 7 113829.70 1290.49 4

7 12 113428.20 1156.77 9

7 12 -2 441.92 160.577 9

-7 -12 2 963.462 177.975 10

-12 19 2 769.042 264.506 4

7 12 -5 407.388 55.3463 2

-7 -12 5 795.701 169.192 10

-12 19 5 600.01 196.011 4

7 12 -5 909.115 172.824 9

12 -19 -5 584.757 63.3829 8

-7 -12 5 445.208 58.2597 5

12 -19 -5 383.324 60.9605 6

-7 -12 8-63.8835 151.162 10

7 12 -8-14.6357 83.0054 9

12 -19 -8 20.25 43.511 8

7 12 -8-66.9744 80.8741 4

12 -19 -8 59.7095 46.7187 6

-19 7 -8-8.25907 47.8089 14

7 12 -8 17.807 37.1354 2

12 -19 -11 517.215 79.5717 8

-19 7 -11 582.057 78.2319 14

-7 -12 11 504.063 148.262 10

19 -7 11 376.96 122.443 10

-19 7 -11 502.11 82.112 11

7 12 -11 578.412 76.2523 2

7 12 -11 680.518 162.952 7

12 -19 -11 530.325 79.4105 6

-19 7 -14 79.2479 37.1256 11

7 12 -14 111.789 36.0032 2

12 -19 -14 43.5541 36.4162 8

12 -19 -17 7.6148 19.0288 8

7 12 -17 33.5995 18.0434 2

-13 19 -17 53.93 22.1759 2

-13 19 -17-33.4636 26.7707 14

-13 19 -14 100.499 48.1465 14

-13 19 -14 45.1047 42.4187 3

-13 19 -14 75.3933 32.5938 11

-13 19 -14 51.5143 38.232 2

-6 -13 -14 94.2047 40.8423 8

-19 6 11 116.359 94.7322 10

13 -19 11 266.54 92.007 10

19 -6 -11 264.327 90.1937 9

-13 19 -11-7.70417 77.0803 3

-6 -13 -11 108.011 53.3276 8

-13 19 -11 117.81 63.3698 14

-13 19 -11 32.1893 62.0611 2

-6 -13 -11 113.309 48.47 6

-13 19 -11 168.165 56.4728 11

-13 19 -8 106.077 50.6587 14

-6 -13 -8 79.6275 42.7728 1

-13 19 -8 122.78 58.7801 2

-13 19 -8 90.3577 57.879 3

-13 19 -8 72.9962 76.0276 4

-13 19 -8 92.14 58.6397 11

-19 6 8 174.567 147.812 10

13 -19 8 222.552 95.2411 10

-6 -13 -8 97.0363 42.6888 6

-6 -13 -8 120.208 55.0597 8

-6 -13 -5 6696.79 512.107 8

-13 19 -5 5935.60 509.997 2

-19 6 5 6197.97 628.303 10

13 -19 5 5932.29 541.913 10

-6 -13 -5 6031.62 504.732 6

-13 19 -5 5896.35 510.323 3

-13 19 -5 5732.03 793.547 4

-6 -13 -2 4863.22 407.775 6

6 13 2 3402.67 454.189 9

-13 19 -2 6045.53 1157.71 4

-13 19 -2 5369.87 410.827 2

-13 19 155988.50 4562.55 4

6 13 -149479.60 4389.54 9

-13 19 154187.90 4293.23 3

13 -19 -414487.70 1187.52 6

-13 19 415919.10 1352.07 4

-6 -13 416173.20 1289.73 10

6 13 -413714.90 1304.97 9

13 -19 -7 499.722 73.0862 6

-6 -13 7 916.011 188.708 10

6 13 -7 498.158 139.058 9

13 -19 -7 608.56 72.6605 8

-13 19 7 541.708 157.62 4

6 13 -7 602.014 164.387 7

6 13 -7 506.11 66.2031 2

-19 6 -7 595.71 85.0359 11

-19 6 -7 348.059 65.5627 1

6 13 -7 429.996 124.524 4

13 -19 -10 61.9071 46.9217 8

6 13 -10-81.5004 124.974 7

13 -19 -10 64.1669 50.2016 6

-6 -13 10 39.4128 113.786 10

-19 6 -10 94.2194 52.8542 11

6 13 -10 86.8449 42.6946 2

-19 6 -10 144.545 49.235 14

-19 6 -13 58.2207 44.8913 14

-6 -13 13 55.1811 73.2045 10

6 13 -13 51.9269 39.7455 2

13 -19 -13 112.019 46.377 8

-19 6 -13 185.961 47.7495 11

13 -19 -16 10.297 24.3757 8

6 13 -16 8.34948 23.145 2

-5 -14 -15 2.32327 30.3926 8

-14 19 -15 16.5278 32.8246 2

-14 19 -15 3.57076 23.8936 11

-19 5 12 57.6127 76.3875 10

-5 -14 -12 165.931 47.8611 6

-14 19 -12 36.5751 71.4196 3

-14 19 -12 55.9301 53.289 14

-14 19 -12 79.5502 46.2707 11

-14 19 -12 81.6145 58.0683 2

-5 -14 -12 98.8918 46.8266 8

-14 19 -9 53.8312 66.5069 3

-14 19 -9-34.6176 54.0062 14

-5 -14 -9 4.31224 47.3148 8

-14 19 -9-14.9295 56.2621 11

-19 5 9-25.2429 135.834 10

14 -19 9-13.3439 78.1959 10

-5 -14 -9 56.438 44.4877 6

-5 -14 -9 164.771 51.0712 1

-14 19 -9 33.7002 58.7849 2

-5 -14 -6 1373.91 124.134 6

-5 -14 -6 1503.19 126.167 1

-14 19 -6 1305.82 295.001 4

-14 19 -6 1266.29 131.742 3

-14 19 -6 1049.68 124.519 14

-14 19 -6 1251.31 131.366 2

14 -19 6 941.274 154.757 10

-19 5 6 1239.25 271.151 10

-5 -14 -6 1272.67 130.522 8

-14 19 -3 3871.70 328.15 2

-14 19 -3 4914.80 956.742 4

-19 5 3 4132.59 424.22 10

-5 -14 -3 3771.87 326.691 6

-14 19 -3 4117.98 330.264 3

5 14 3 3186.09 375.653 9

-19 5 0 3803.70 325.681 5

5 14 0 4247.60 430.727 9

-19 5 -3 76.1069 30.9488 5

-5 -14 3 118.782 120.237 10
[truncated: 843,551 more chars]
