# Supplementary material for: Kinetic trapping of 2,4,6-tris(4-pyridyl)benzene and ZnI2 into M12L8 poly-[n]-catenanes using solution and solid-state processes
Source: Sci Rep. 2023 Apr 5;13:5605. doi: 10.1038/s41598-023-32661-x (PMC10076325; doi:10.1038/s41598-023-32661-x)

checkCIF/PLATON report

Structure factors have been supplied for datablock(s) TPB_ZnI2_NB_RT

THIS REPORT IS FOR GUIDANCE ONLY. IF USED AS PART OF A REVIEW PROCEDURE FOR PUBLICATION, IT SHOULD NOT REPLACE THE EXPERTISE OF AN EXPERIENCED CRYSTALLOGRAPHIC REFEREE.

No syntax errors found.   CIF dictionary   Interpreting this report

Datablock: TPB_ZnI2_NB_RT

Bond precision:	C-C = 0.0218 A		Wavelength=1.54184
Cell:	a=38.6805(7)	b=38.6805(7)	c=16.0202(3)
	alpha=90	beta=90	gamma=120
Temperature:	303 K		

	Calculated	Reported
Volume	20757.9(8)	20757.9(8)
Space group	R -3	R -3 :H
Hall group	-R 3	-R 3
Moiety formula	C168 H120 I24 N24 Zn12, 6(C6 H5 N O2)	?
Sum formula	C204 H150 I24 N30 O12 Zn12	C204 H150 I24 N30 O12 Zn12
Mr	7043.82	7043.57
Dx,g cm-3	1.690	1.690
Z	3	3
Mu (mm-1)	22.539	22.539
F000	9936.0	9936.0
F000'	9884.88	
h,k,lmax	48,48,20	42,48,19
Nref	9673	9032
Tmin,Tmax	0.172,0.324	0.032,0.130
Tmin'	0.070	

Correction method= # Reported T Limits: Tmin=0.032 Tmax=0.130
AbsCorr = CYLINDER

Data completeness= 0.934   Theta(max)= 76.440

R(reflections)= 0.0753(6532)   wR2(reflections)= 0.2561(9032)

S = 1.052   Npar= 386

The following ALERTS were generated. Each ALERT has the format
test-name_ALERT_alert-type_alert-level.
Click on the hyperlinks for more details of the test.

Alert level A

```
PLAT602_ALERT_2_A Solvent Accessible VOID(S) in Structure .......          ! Check
```

Author Response: The central area of M12L8 contains disordered solvent.

Alert level B

```
PLAT342_ALERT_3_B Low Bond Precision on  C-C Bonds ...............    0.02181 Ang.
```

Alert level C

```
ABSTY02_ALERT_1_C  An _exptl_absorpt_correction_type has been given without
            a literature citation. This should be contained in the
            _exptl_absorpt_process_details field.
            Absorption correction given as cylinder
PLAT084_ALERT_3_C High wR2 Value (i.e. > 0.25) ..................       0.26 Report
PLAT094_ALERT_2_C Ratio of Maximum / Minimum Residual Density ....       2.48 Report
PLAT202_ALERT_3_C Isotropic non-H Atoms in Anion/Solvent .........          9 Check
             O2      O3      N9      C1      C2      C6              etc.
PLAT234_ALERT_4_C Large Hirshfeld Difference N31      --C3       .       0.24 Ang.
PLAT234_ALERT_4_C Large Hirshfeld Difference N31      --C4S      .       0.24 Ang.
PLAT234_ALERT_4_C Large Hirshfeld Difference C32      --C35      .       0.18 Ang.
PLAT241_ALERT_2_C High   'MainMol' Ueq as Compared to Neighbors of         C3 Check
PLAT241_ALERT_2_C High   'MainMol' Ueq as Compared to Neighbors of        C18 Check
PLAT241_ALERT_2_C High   'MainMol' Ueq as Compared to Neighbors of        C35 Check
PLAT242_ALERT_2_C Low    'MainMol' Ueq as Compared to Neighbors of        Zn5 Check
PLAT242_ALERT_2_C Low    'MainMol' Ueq as Compared to Neighbors of        N25 Check
PLAT242_ALERT_2_C Low    'MainMol' Ueq as Compared to Neighbors of        N31 Check
PLAT242_ALERT_2_C Low    'MainMol' Ueq as Compared to Neighbors of        C32 Check
PLAT243_ALERT_4_C High   'Solvent' Ueq as Compared to Neighbors of        C13 Check
PLAT243_ALERT_4_C High   'Solvent' Ueq as Compared to Neighbors of        C19 Check
PLAT244_ALERT_4_C Low    'Solvent' Ueq as Compared to Neighbors of         N9 Check
PLAT244_ALERT_4_C Low    'Solvent' Ueq as Compared to Neighbors of         C1 Check
PLAT244_ALERT_4_C Low    'Solvent' Ueq as Compared to Neighbors of         C2 Check
PLAT260_ALERT_2_C Large Average Ueq of Residue Including        I1      0.106 Check
PLAT334_ALERT_2_C Small <C-C> Benzene Dist.   C33      -C34_a    .       1.37 Ang.
PLAT369_ALERT_2_C Long   C(sp2)-C(sp2) Bond  C7       - C16      .       1.53 Ang.
PLAT906_ALERT_3_C Large K Value in the Analysis of Variance ......      2.595 Check
PLAT911_ALERT_3_C Missing FCF Refl Between Thmin & STh/L=    0.600         34 Report
PLAT918_ALERT_3_C Reflection(s) with I(obs) much Smaller I(calc) .          1 Check
PLAT971_ALERT_2_C Check Calcd Resid. Dens.  0.83Ang From I1              2.44 eA-3
PLAT971_ALERT_2_C Check Calcd Resid. Dens.  0.62Ang From I1              1.62 eA-3
PLAT971_ALERT_2_C Check Calcd Resid. Dens.  0.29Ang From I3              1.51 eA-3
PLAT973_ALERT_2_C Check Calcd Positive Resid. Density on       Zn5       1.29 eA-3
PLAT976_ALERT_2_C Check Calcd Resid. Dens.  0.43Ang From O3      .      -0.44 eA-3
PLAT977_ALERT_2_C Check Negative Difference Density on H19       .      -0.32 eA-3
```

Alert level G

```
PLAT002_ALERT_2_G Number of Distance or Angle Restraints on AtSite         11 Note
PLAT003_ALERT_2_G Number of Uiso or Uij Restrained non-H Atoms ...         14 Report
PLAT172_ALERT_4_G The CIF-Embedded .res File Contains DFIX Records         12 Report
PLAT173_ALERT_4_G The CIF-Embedded .res File Contains DANG Records          2 Report
PLAT174_ALERT_4_G The CIF-Embedded .res File Contains FLAT Records          1 Report
PLAT178_ALERT_4_G The CIF-Embedded .res File Contains SIMU Records          2 Report
PLAT232_ALERT_2_G Hirshfeld Test Diff (M-X)  I2       --Zn6      .        7.1 s.u.
PLAT301_ALERT_3_G Main Residue  Disorder ..............(Resd  1  )         5% Note
PLAT367_ALERT_2_G Long?  C(sp?)-C(sp?) Bond  C32      - C34      .       1.51 Ang.
PLAT411_ALERT_2_G Short Inter H...H Contact  H35      ..H5S      .       1.45 Ang.
                                   -1/3+y,1/3-x+y,4/3-z  =     14_456 Check
PLAT432_ALERT_2_G Short Inter X...Y Contact  C35      ..C5S      .       3.07 Ang.
                                   -1/3+y,1/3-x+y,4/3-z  =     14_456 Check
PLAT794_ALERT_5_G Tentative Bond Valency for Zn5       (II)      .       2.00 Info
PLAT860_ALERT_3_G Number of Least-Squares Restraints ............          92 Note
PLAT883_ALERT_1_G No Info/Value for _atom_sites_solution_primary .     Please Do !
PLAT910_ALERT_3_G Missing # of FCF Reflection(s) Below Theta(Min).          1 Note
PLAT912_ALERT_4_G Missing # of FCF Reflections Above STh/L=  0.600        591 Note
PLAT941_ALERT_3_G Average HKL Measurement Multiplicity ...........        4.7 Low
PLAT950_ALERT_5_G Calculated (ThMax) and CIF-Reported Hmax Differ           6 Units
PLAT965_ALERT_2_G The SHELXL WEIGHT Optimisation has not Converged     Please Check
PLAT978_ALERT_2_G Number C-C Bonds with Positive Residual Density.          1 Info
```

---

1 **ALERT level A** = Most likely a serious problem - resolve or explain
1 **ALERT level B** = A potentially serious problem, consider carefully
31 **ALERT level C** = Check. Ensure it is not caused by an omission or oversight
20 **ALERT level G** = General information/check it is not something unexpected

2 ALERT type 1 CIF construction/syntax error, inconsistent or missing data
26 ALERT type 2 Indicator that the structure model may be wrong or deficient
10 ALERT type 3 Indicator that the structure quality may be low
13 ALERT type 4 Improvement, methodology, query or suggestion
2 ALERT type 5 Informative message, check

It is advisable to attempt to resolve as many as possible of the alerts in all categories. Often the minor alerts point to easily fixed oversights, errors and omissions in your CIF or refinement strategy, so attention to these fine details can be worthwhile. In order to resolve some of the more serious problems it may be necessary to carry out additional measurements or structure refinements. However, the purpose of your study may justify the reported deviations and the more serious of these should normally be commented upon in the discussion or experimental section of a paper or in the "special_details" fields of the CIF. checkCIF was carefully designed to identify outliers and unusual parameters, but every test has its limitations and alerts that are not important in a particular case may appear. Conversely, the absence of alerts does not guarantee there are no aspects of the results needing attention. It is up to the individual to critically assess their own results and, if necessary, seek expert advice.

Publication of your CIF in IUCr journals

A basic structural check has been run on your CIF. These basic checks will be run on all CIFs submitted for publication in IUCr journals (*Acta Crystallographica*, *Journal of Applied Crystallography*, *Journal of Synchrotron Radiation*); however, if you intend to submit to *Acta Crystallographica Section C* or *E* or *IUCrData*, you should make sure that full publication checks are run on the final version of your CIF prior to submission.

Publication of your CIF in other journals

Please refer to the *Notes for Authors* of the relevant journal for any special instructions relating to CIF submission.

PLATON version of 18/05/2022; check.def file version of 17/05/2022

Datablock TPB_ZnI2_NB_RT - ellipsoid plot

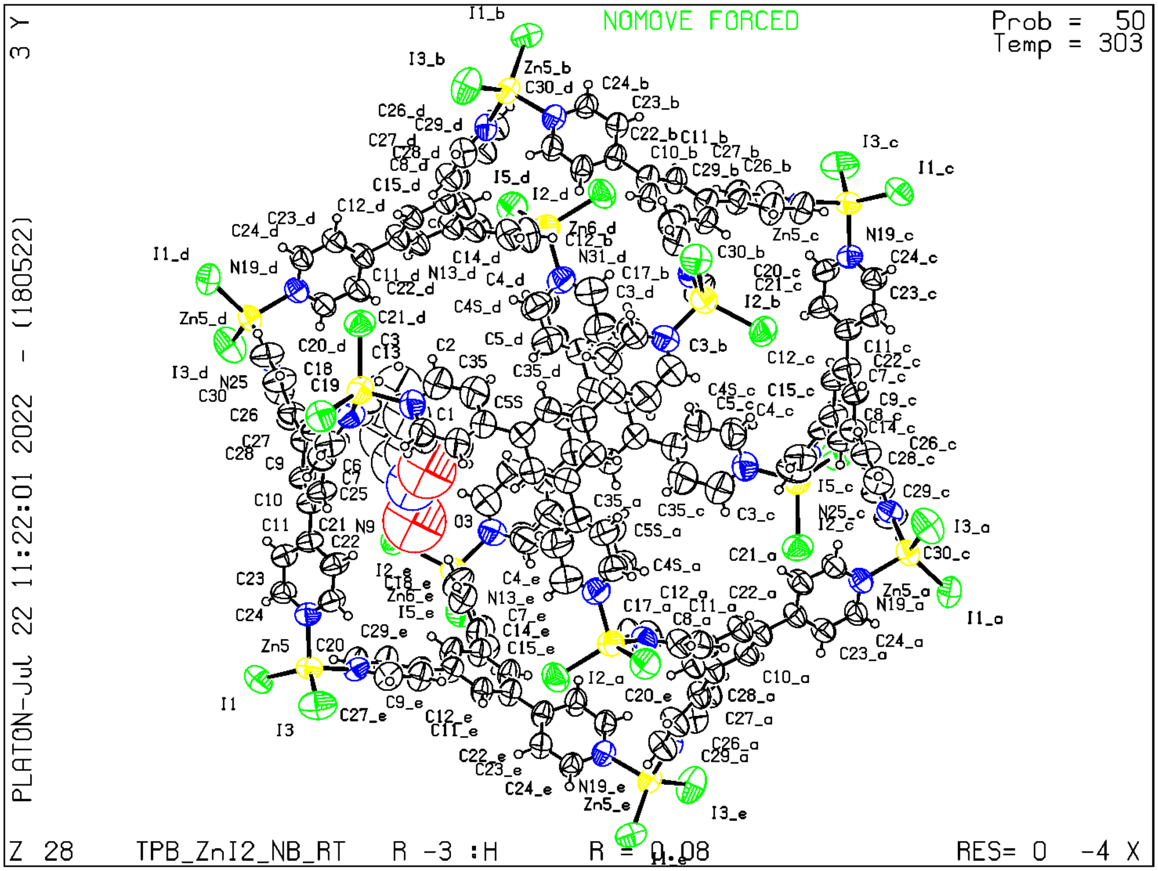

Supplement: Supplementary file 2 — Supplementary Information 2. [file 41598_2023_32661_MOESM2_ESM.pdf]
